# Intensive Distribution of G_2_-Quaduplexes in the Pseudorabies Virus Genome and Their Sensitivity to Cations and G-Quadruplex Ligands

**DOI:** 10.3390/molecules24040774

**Published:** 2019-02-21

**Authors:** Hui Deng, Bowen Gong, Zhiquan Yang, Zhen Li, Huan Zhou, Yashu Zhang, Xiaohui Niu, Sisi Liu, Dengguo Wei

**Affiliations:** 1 Laboratory of Medicinal Biophysical Chemistry, College of Science, Huazhong Agricultural University, Wuhan 430070, China; denghui0923@163.com (H.D.); night@webmail.hzau.edu.cn (B.G.); lizhen5a@163.com (Z.L.); zhouhuan@webmail.hzau.edu.cn (H.Z.); 2 Hubei Key Laboratory of Agricultural Bioinformatics, College of Informatics, Huazhong Agricultural University, Wuhan 430070, China; yang_zq@foxmail.com (Z.Y.); niuxiaoh@126.com (X.N.); 3 Laboratory of Medicinal Biophysical Chemistry, College of Plant Science and Technology, Huazhong Agricultural University, Wuhan 430070, China; yashu@webmail.hzau.edu.cn

**Keywords:** alphaherpesviruses, pseudorabies virus, genome, G-quadruplex, G-quadruplex ligand, nucleic acids conformation, regulatory element

## Abstract

Guanine-rich sequences in the genomes of herpesviruses can fold into G-quadruplexes. Compared with the widely-studied G_3_-quadruplexes, the dynamic G_2_-quadruplexes are more sensitive to the cell microenvironment, but they attract less attention. Pseudorabies virus (PRV) is the model species for the study of the latency and reactivation of herpesvirus in the nervous system. A total of 1722 G_2_-PQSs and 205 G_3_-PQSs without overlap were identified in the PRV genome. Twelve G_2_-PQSs from the CDS region exhibited high conservation in the genomes of the *Varicellovirus* genus. Eleven G_2_-PQSs were 100% conserved in the repeated region of the annotated PRV genomes. There were 212 non-redundant G_2_-PQSs in the 3′ UTR and 19 non-redundant G_2_-PQSs in the 5′ UTR, which would mediate gene expression in the post-transcription and translation processes. The majority of examined G_2_-PQSs formed parallel structures and exhibited different sensitivities to cations and small molecules in vitro. Two G_2_-PQSs, respectively, from 3′ UTR of *UL5* (encoding helicase motif) and *UL9* (encoding sequence-specific ori-binding protein) exhibited diverse regulatory activities with/without specific ligands in vivo. The G-quadruplex ligand, NMM, exhibited a potential for reducing the virulence of the PRV Ea strain. The systematic analysis of the distribution of G_2_-PQSs in the PRV genomes could guide further studies of the G-quadruplexes’ functions in the life cycle of herpesviruses.

## 1. Introduction

**Figure 1:**
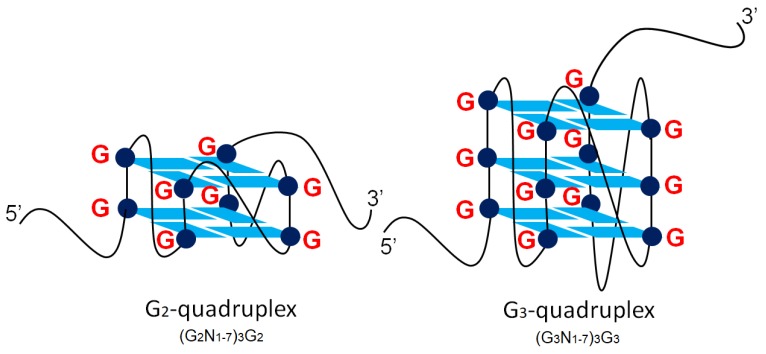
Schematic diagram of the G_2_-quadruplex and the G_3_-quadruplex.

The life-long latency of herpesviruses poses potential threats to the host at any time, and the reason for the wide existence of the GC-rich sequences in herpesvirus genomes remains unknown [1]. Guanine-rich sequences have been discovered to form special DNA or RNA secondary structures called G-quadruplexes. They are composed of π-stacked G-quartets via hydrogen-bonded structure in DNA or RNA [2]. Each G-quartet contains four guanines held by eight hydrogen bonds, and coordinated with a central monovalent cation, such as K^+^ and Na^+^. The G_3_-putative quadruplex sequences (G_3_-PQSs) in the form of (G_3+_N_1-7_)_3_G_3+_ form three or more G-quartets in the structures (Figure 1), and they exhibited good thermal stability under near-physiological conditions [3]. The function of these G_3_-quadruplex sequences has been well-studied in the genomes of humans [3], maize [4] and human viruses [5,6]. Recently, some studies have reported on the regulatory function of G_2_-quadruplexes (Figure 1), with two G-quartets in the viral mRNA translation of Epstein–Barr virus [7] and in the polyamine biosynthesis pathway of eukaryotic cells [8]. However, G_2_-putative quadruplex sequences (G_2_-PQSs) in the form of (G_2+_N_1-7_)_3_G_2+_ have not attracted wide attention, due to their relatively low stability, even though they exhibit certain superiority in sensitivity. The systematic analysis of G_2_-PQSs in the genomes of herpesviruses could explore more functions of G-rich sequences in herpesviruses, compared with that of G_3_-PQSs.

Pseudorabies virus (PRV) represents a good model for the study of G_2_-quadruplex functions in herpesvirus genomes. This virus is a herpesvirus of the *Varicellovirus* genus in the *Alphaherpesvirinae* subfamily that is included in the family *Herpesviridae*. The *Alphaherpesvirinae* chooses the nervous system for latency [9]. PRV causes neuronal and lethal infection in many animal species, yet posing little or no danger to humans [10,11,12,13,14]. PRV has been used as a model species for studying the cycle of infection, latency, and reactivation, which are critical processes for the survival of alphaherpesviruses [10]. Vaccination is the most effective approach to preventing virus infection. However, herpesviruses can establish latency in the host after the first infection, and they can reactivate to cause serious diseases in their host. Failure of vaccination will result in a big threat to humans and animals. For example, the Bartha-K61 vaccine has been used worldwide, and it played an important role in the eradication of pseudorabies virus in many countries. Nevertheless, it had failed to protect piglets from being infected by several virulent PRV strains in China, resulting in PRV re-outbreak in 2011 [15,16,17,18,19,20,21,22,23]. In order to prevent and cure herpesvirus infections successfully, it could be helpful to reveal the latency-reactivation mechanism, based on the characteristics of herpesvirus genomes and their feature in host cells. The Human gammaherpesvirus 4 (Epstein–Barr virus, EBV) was assumed to modulate immune evasion with a G_2_-quadruplex forming in the coding sequence (CDS) of Epstein–Barr virus-encoded nuclear antigen 1 (EBNA1) [7]. The PRV may respond to the defense of the host cells, through the formation and resolution of G-quadruplexes to regulate latency and reactivation. This study is aimed to provide clues for vaccine and drug development at the nucleic acid level.

In this work, a systematic analysis will be carried out to locate the distribution of G_2_/G_3_-quadruplex sequences in the PRV genome. The conservation of the putative G-quadruplex-forming sequences will be evaluated in the PRV strains and in the *Varicellovirus* genus. The evolutionary differences in the G-quadruplexes between non-human infectious herpesvirus and human herpesviruses will be discussed further. G_2_-PQSs structure types and their sensitivities to different cations and ligands will be examined, for their roles in regulating gene expression. The study of a classic G-quadruplex ligand, *N*-methyl mesoporphyrinIX (NMM), exhibited potential for inhibiting the virulence of the PRV Ea strain. This study will further investigate G_2_-quadruplexes working as sensors in response to small molecules, proteins, and physiological cation conditions in the specific microenvironment, to reveal the latency-reactivation mechanism of herpesviruses.

## 2. Results

### 2.1. Bioinformatic Analysis

#### 2.1.1. Genome-Wide Analysis of G_3_-PQSs and G_2_-PQSs Distributions in the PRV Genome

**Figure 2:**
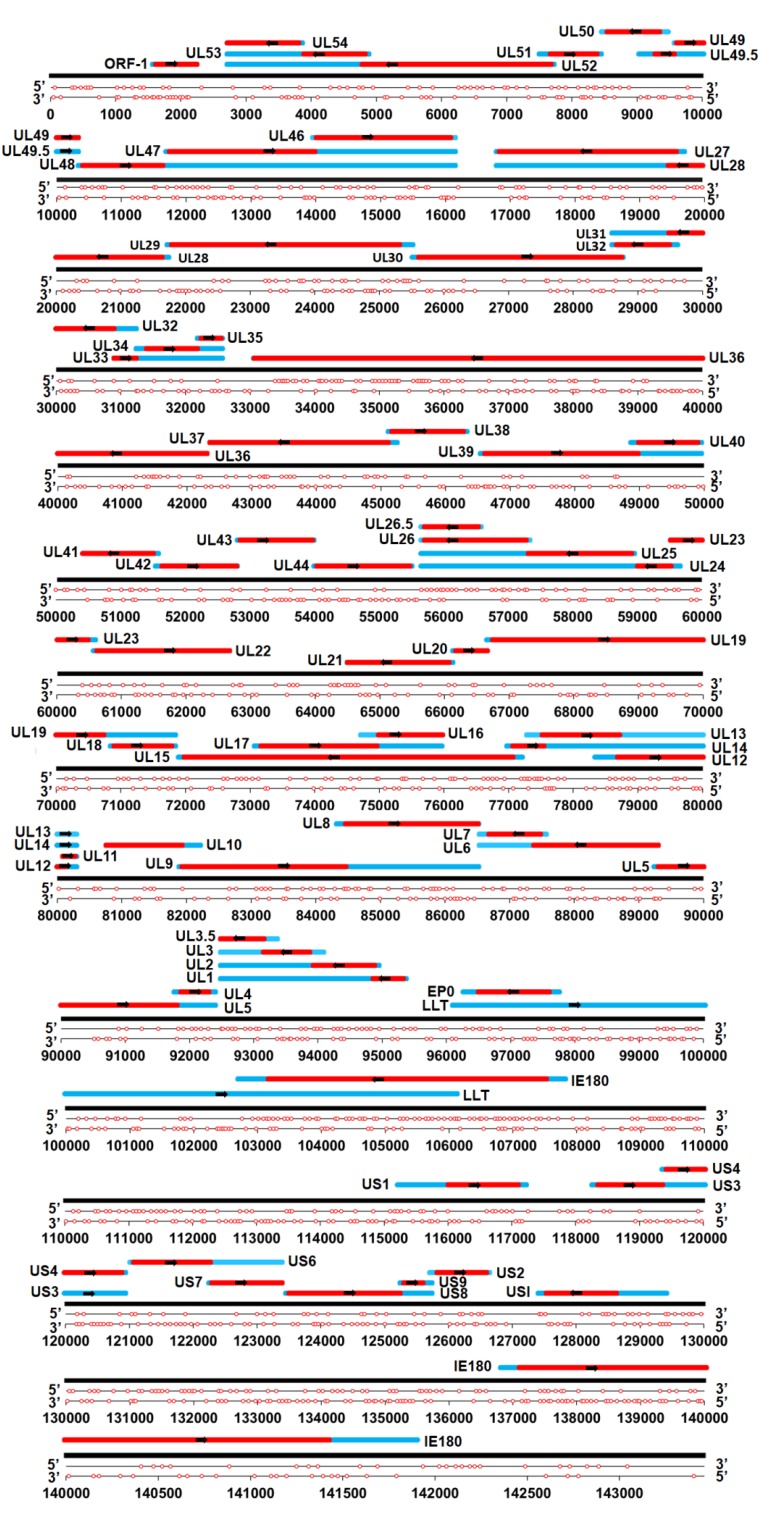
Distribution of G_2_-putative quadruplex sequences (G_2_-PQSs) in the double-strand genome of pseudorabies virus (PRV). One red circle indicates one G_2_-PQS. The red bars indicate the coding sequence (CDS) region. The blue bars indicate the untranslated region.

The putative G_3_-quadruplex sequences (G_3_-PQSs) in the PRV genome were predicted with the Quadparser program [3] in the form of the GnNm sequence (where *n* ≥ 3 and 1 ≤ *m* ≤ 7). The putative G_2_-quadruplex sequences (G_2_-PQSs) were predicted with the same program, but in the modified sequence form (*n* ≥ 2). The genome size of PRV was 143,461 bp, and it encoded 69 proteins. The analysis of the PRV reference genome (NC_006151.1) indicated that 1722 G_2_-PQSs and 205 G_3_-PQSs were distributed in the PRV genome (Figure 2; File S1), with the density of the G_2_-PQSs being 12 PQS/kb.

As the formation of the G-quadruplex could affect either transcription or translation, our analysis of PQSs was based on double strands with the well-annotated regions, and the predicted regulatory regions in the PRV reference genome (File S2). The 3’ end untranslated regions (3′ UTRs) from 63 genes, and the 5’ end untranslated regions (5′ UTRs) from 61 genes were annotated in the PRV reference genome in The National Center for Biotechnology Information (NCBI) Genome database [24]. The promoters of the annotated PRV genes were predicted to be 1 kb upstream of the annotated transcription start site of each gene. G_2_-PQSs were higher in density in the CDS region and in the large latency transcript (LLT) than in the repeat region, while the G_3_-PQSs were densely distributed in the repeat region (Table 1). The density of the G_2_-PQSs in the 3′ UTR was higher than that in the 5′ UTR (Table 1). In the promoter regions, the G_2_-PQSs density was 5.34 PQS/kb, and it was more than eight-fold that of the G_3_-PQSs density (Table 1).

The PQS monomer was named the single G-quadruplex-forming sequence, and the sequences forming more than two possible simultaneous G-quadruplexes were defined as the PQS cluster. The G_3_-quadruplex cluster, forming a highly stable structure, was identified in the repetitive region of the Herpes simplex virus type 1 (HSV-1) genome [25]. This study found that 86.8% of G_3_-PQSs and 77% of G_2_-PQSs were monomers in the genome of PRV (File S1). 27 non-redundant G_3_-PQSs clusters were located in the regulatory regions, rather than in the coding region in the PRV genome. Sixty-nine G_2_-PQS clusters were distributed in the repeat regions, and 122 G_2_-PQS clusters were located in the coding region in the PRV genome (Table 1).

#### 2.1.2. Conserved G_2_-PQSs in the Coding Sequences of PRV Genes

##### G_2_-PQS Distribution in the Coding Regions of Genes Involved in the Replication Cycle of PRV

**Figure 3:**
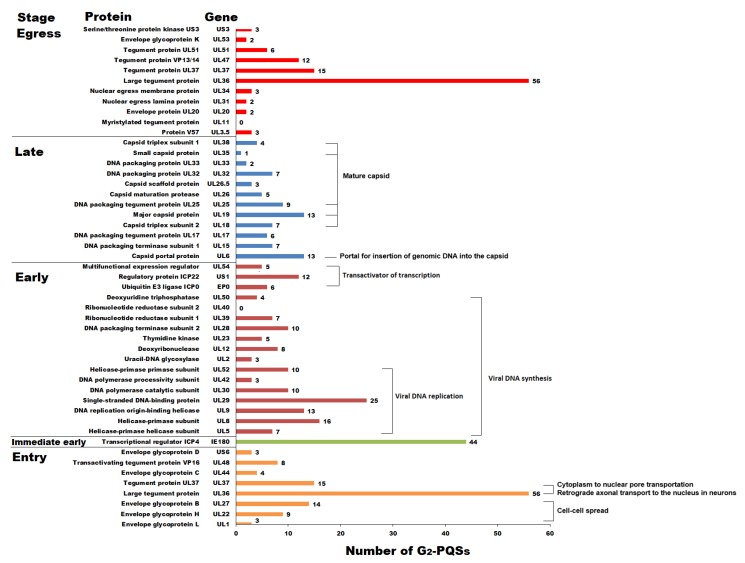
Number of G_2_-PQSs in the coding sequences of the genes that are involved in the PRV replication cycle.

The G_2_-quadruplex formation in the open reading frame (ORF) of Epstein–Barr virus-encoded nuclear antigen 1 (EBNA1) led to decreased mRNA translation in the Epstein–Barr virus (EBV), which suggested that the G-quadruplex in viral transcripts acts as a specific regulatory element to regulate translation level and immune evasion [7]. The PRV replication cycle contains five main processes, including entry, immediate early stage, early stage, late stage, and egress [10]. There were 481 G_2_-PQSs in the ORFs of the genes in above five stages (Figure 3). 112 G_2_-PQSs were found in the entry stage, and 30 of them were involved in important envelope glycoproteins recognizing host cells. After the entry stage, PRV is in the immediate early stage. It transcribes and expresses only one immediate early gene, *IE180*; this was different from human herpesviruses, which transcribe three to five immediate early genes. *IE180* is required for the effective transcription of early viral genes [10]. There were two copies of *IE180* in the PRV genome, and 22 G_2_-PQSs were located in the coding sequence of each copy. The genes in the early stage contained 144 G_2_-PQSs, which was twice as many as the total number of G_2_-PQSs in the late stage (*n* = 77). The early-stage proteins had functions in transactivation and viral DNA synthesis, while the late-stage proteins were mainly responsible for DNA packaging and capsid maturation. A total of 104 G_2_-PQSs were identified in the egress stage, and most of them were present in the tegument proteins, which are important for virion formation before cell-to-cell movement (Figure 3).

##### G_2_-PQSs Involved in the Processes of DNA Replication and Encapsidation

Eighty-four G_2_-PQSs were identified in the genes involved in the viral DNA replication process (Figure 3), including *UL5*, *UL8*, *UL9*, *UL29*, *UL30*, *UL42*, and *UL52*. In total, 39% of these G_2_-PQSs were in the coding region of the helicase–primerase complex formed by *UL5*, *UL8*, and *UL52*. Twenty-five G_2_-PQSs were found in the gene *UL29*, encoding the single-stranded DNA-binding protein ICP8. The DNA replication origin-binding helicase *UL9* contained 13 G_2_-PQSs in its coding sequence, and the DNA polymerase complex *UL30*/*UL42* contained 13 G_2_-PQSs in total.

In the late stage, *UL38*, *UL35*, *UL25*, *UL19*, *UL18*, and *UL6* encoded the proteins of the mature capsid constituents; these are required for the capsid assembly in the nucleus of the cell, and two scaffolding proteins (*UL26* and *UL26.5*) have been reported to have participated in capsid formation [10]. The above late proteins accounted for 61% of the G_2_-PQSs in the late stage (Figure 3).

##### G_2_-PQSs Involved in the Processes of Virus Entry and Egress

Both *UL36* and *UL37* were the tegument protein genes required in both the entry and egress stages [10]. The VP1/2 protein (*UL36*) is the important component of the inner layer of tegument proteins, and it was reported to be associated with the capsid during PRV transport across cytoplasm into the nuclear pore [27]. The production of VP1/2 protein is cut off by truncating the translation of *UL36* gene, resulting in the failed transportation of the virus particle. Fifty-six G_2_-PQSs were observed in the CDS region of *UL36*, accounting for 50% of the total number of G_2_-PQSs in the stages it involved (Figure 3). The viral glycoproteins gC (*UL44*), gD (*US6*), gB (*UL27*), gH (*UL22*), and gL (*UL1*) mediated a cascade required by PRV virions to enter specific cells, and totally there were 29 G_2_-PQSs in the ORFs of glycoproteins (Figure 3).

##### Conservation and Potential Function of G_2_-PQSs in CDS Region in the Varicellovirus Genus 

Since coding sequences had higher conservation than regulatory regions among different viruses in the same genus, the inter-species analysis of the conservation of the G_2_-PQSs in the coding region was performed within 11 *Varicellovirus* species including PRV. The conservation of the 494 G_2_-PQSs in the CDS region was analyzed. The analysis indicated that 55.3% of G_2_-PQSs exhibited the conservation score higher than 0.2 (Figure S1). Twelve of these 494 G_2_-PQSs were found with the conservation score higher than 0.7 (Table 2). Five G_2_-PQSs of them were derived from the immediate early gene *IE180*, and two G_2_-PQSs were derived from the gene *UL30* encoding the DNA polymerase catalytic subunit. One G_2_-PQS derived from the gene *UL13* encoding protein-serine/threonine kinase was 100% conserved in the *Varicellovirus* genus (Table 2). The other G_2_-PQSs with their conservation scored as 1.0 were from the gene *UL33* associated with *UL28* and *UL15* to contribute to DNA cleavage and PRV package. The DNA cleavage and encapsidation related gene *UL17* and major capsid protein gene *UL19* contained one conserved G_2_-PQS in their coding sequences. Two tegument protein genes *UL16* and *UL47* also contained one conserved G_2_-PQS, respectively (Table 2).

#### 2.1.3. Distribution Analysis of G_2_-PQSs in Regulatory Regions in PRV Genomes

Promoter, untranslated region, and intergenic region, which were important regulatory regions, had multiple functions in gene regulation. G_3_-PQSs were reported to be mainly located in the regulatory regions in the genomes of human herpesviruses [5], while less report on G_2_-PQSs was available. Systematic analysis of G_2_-PQS could be conducive to the exploration of their regulatory function. In this study, PRV was found to have higher density of G_2_-PQS than G_3_-PQS in the repeat regions (Table 1).

##### Dozens of Conserved G_2_-PQSs in the Repeat Regions Related to Genome Recombination

Terminal repeat (TR) region is important for genome replication in some herpesviruses. The G-quadruplex in the TR region of gammaherpesvirus Kaposi sarcoma-associated herpesvirus (KSHV) altered the latent DNA replication and episomal persistence [28]. Furthermore, the stabilization of HSV-1 G-quadruplexes in the repeat region inhibited DNA polymerase processing and viral DNA replication [25]. The PRV genome was similar to the HSV-1 genome which was characterized by two unique regions (UL and US), and the US region was flanked by the internal and terminal repeat sequences (IRS and TRS, respectively). During PRV infection, the recombination between the inverted repeats produced two possible isomers of the genome with the U_S_ region in opposite orientation. Both isomers were infectious and were in equimolar amount after infection, and the PRV genome was circularized upon entry into the host nucleus through blunt end ligation independent of any viral protein synthesis (reviewed in [10]).

**Figure 4:**
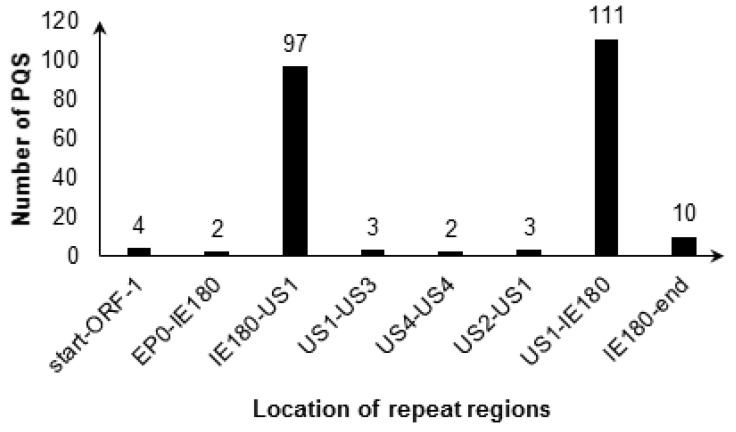
Distribution of G_2_-PQS in repeat regions in PRV genome.

**Figure 5:**
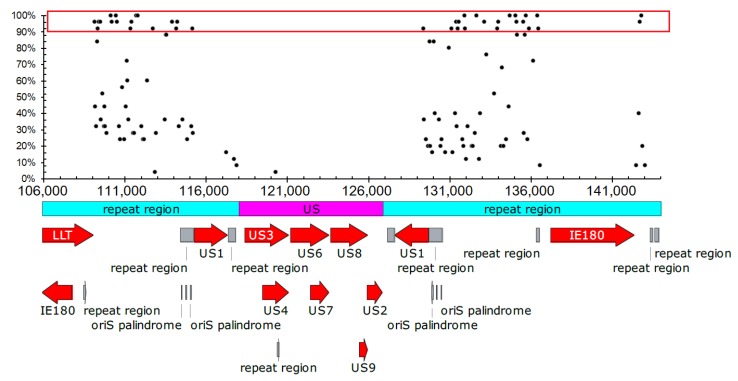
Conservation percentage of putative G-quadruplex sequences in the repeat region. Red block indicated the G_2_-PQSs with conservation percentage more than 90%.

4 G_2_-PQSs were located in 0–656 bp region, and 208 G_2_-PQSs were located in the two regions between genes *IE180* and *US1* (Figure 4). The conservation percentage of 117 G_2_-PQSs on the sense strand of PRV genome ranged from 4% to 100% (Figure 5). There were 38 G_2_-PQSs in the repeated region between *IE180* and *US1* with conservation percentage more than 90% (Figure 5), including 11 G_2_-PQSs with 100% conservation rate (Table S1). These conserved GC-rich sequences might form G-quadruplexes in the inverted repeat region, mediating the genome recombination after infection. 14 G_2_-PQSs existing between *IE180* and *ORF1* may be switches of PRV replication in circular genome.

##### G_2_-PQSs in the Untranslated Regions of PRV Genes

The untranslated regions (UTR) are important regulatory region for gene expression. G-quadruplex-forming sequences in the 5′ UTR acting as translational repressor have been reported in several human genes [29,30]. The 3′ UTR is related to mRNA stability, alternative splicing, polyadenylation, and localization. G-quadruplexes in the 3′ UTR of CaMKIIa (Ca^2+^/calmodulin-dependent protein kinase II) and PDS-95 (post-synaptic density protein 95) mRNAs are responsible for the transport of those mRNA in neurites in vivo [31].

**Figure 6:**
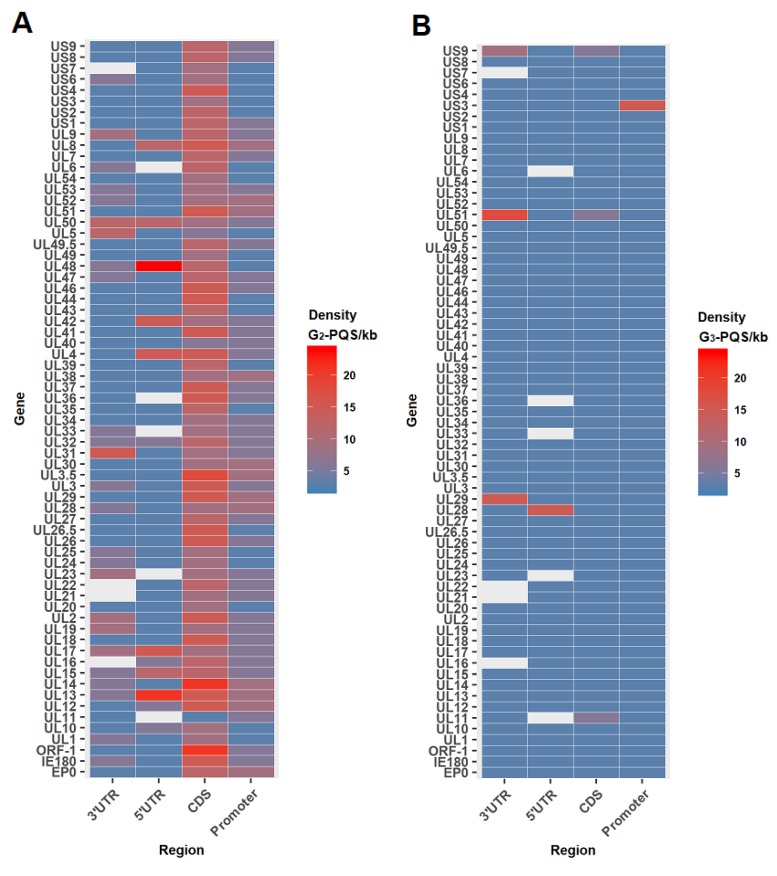
Heat maps of PQS density in coding sequence and regulatory regions of each PRV gene. (**A**) Heat map of G_2_-PQSs; (**B**) Heat map of G_3_-PQSs.

**Figure 7:**
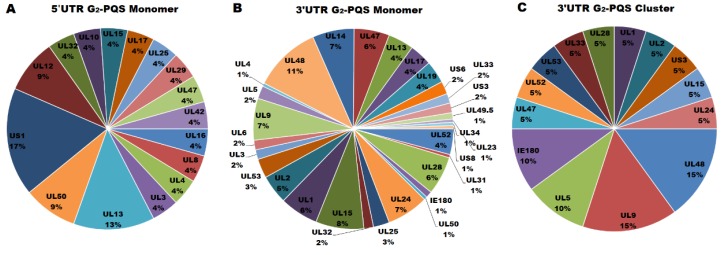
Quantitative distribution of G_2_-PQSs in the untranslated regions of annotated genes in PRV genome. (**A**) G_2_-PQS monomers in the 5′ UTR of the PRV genes. (**B**) G_2_-PQS monomers in the 3′ UTR of the PRV genes. (**C**) G_2_-PQS clusters in the 3′ UTR of the PRV genes.

Nineteen non-redundant G_2_-PQSs and five G_3_-PQSs were found in the 5′ UTR of the annotated PRV genes (Figure S2). Among all the genes in PRV genome, the gene *UL13* contained four G_2_-PQSs in its 212-bp 5′ UTR with the highest density of G_2_-PQS in the 5′ UTR regions, and the gene *UL28* contained one G_3_-PQS in its 72-bp 5′ UTR with the highest density of G_3_-PQS in the 5′ UTR (Figure 6). The transactivator gene *US1* contained four G_2_-PQSs in its 5′ UTR, accounting for 17% of G_2_-PQS monomers found in 5′ UTR region in the whole genome (Figure 7A). *UL13* had three G_2_-PQS monomers in its 5′ UTR, accounting for 13% of G_2_-PQS monomers in 5′ UTR in the PRV genome (Figure 7A). In the early protein genes, the deoxyribonuclease gene *UL12* and dUTPase gene *UL50* had two G_2_-PQS monomers in their 5′ UTR, respectively (Figure 7A). One G_2_-PQS cluster was found in the gene *UL13*, which was the unique G_2_-PQS cluster in the all annotated 5′ UTR of PRV genes (File S2).

The G_2_-PQSs in the 3′ UTR of annotated PRV genes were more than ten folds of those found in the 5′ UTR of the annotated PRV genes. There existed 212 non-redundant G_2_-PQSs and 8 G_3_-PQSs in the 3′ UTR of PRV genes (Figure S2). These results suggested that G_2_-quaduplexes might regulate gene expression frequently by effecting 3′ UTR secondary structure. The genes *UL51* and *UL29* had the highest density of G_3_-PQSs in their 3′ UTR regions (Figure 6).

More than 70 G_2_-PQS monomers were found in the 3′ UTR of the genes related to viral genome replication, envelopment, and packaging processes (Figure S2). *UL48* encoded the VP16 protein exerting multiple functions in the PRV, including transactivation in gene regulation and secondary envelopment in viral egress [10]. 21 G_2_-PQS monomers were found in the 3′ UTR of gene *UL48*, accounting for 11% of all the G_2_-PQS monomers in the 3′ UTR in the PRV genome (Figure 7B). The late stage gene *UL15* encoding DNA packaging terminase subunit 1 had 15 G_2_-PQS monomers in its 3′ UTR. The *UL9* encoded the sequence-specific ori-binding protein OBP which formed the ATP-dependent helicase motif. OBP was essential for the replication of the viral DNA [10]. *UL9* had 13 G_2_-PQS monomers in its 3′ UTR. The tegument protein coding gene *UL14* and type III membrane protein coding gene *UL24* had the same number of G_2_-PQSs in their 3′ UTR (Figure S2). The G_2_-PQS monomers from *UL9*, *UL14* and *UL24* accounted for 21% of the entire number of G_2_-PQS monomers in the 3′ UTR (Figure 7B).

Another important characteristic was that G_2_-PQS clusters existed in the 3′ UTR of PRV gene densely. The gene *UL48* had 3 G_2_-PQS clusters in its 3′ UTR. The DNA replication related gene group had far more G_2_-PQS clusters than other functional gene groups. The *UL9/UL5/UL52* group had 6 G_2_-PQS clusters in the 3′ UTR region (Figure 7C). The proteins coded by above three genes included the sequence-specific ori-binding protein, helicase motif, and primase subunit. The G_2_-PQSs from 3′ UTR of *UL5* and *UL9* were validated in further experiments. The identification of the functional G_2_-PQSs in the untranslated regions of genes in transactivation, and viral DNA synthesis processes will provide the elements for post-transcriptional regulation of virus genes.

##### A Wide Distribution of G_2_-PQSs in the Large Latency Transcript 

**Figure 8:**
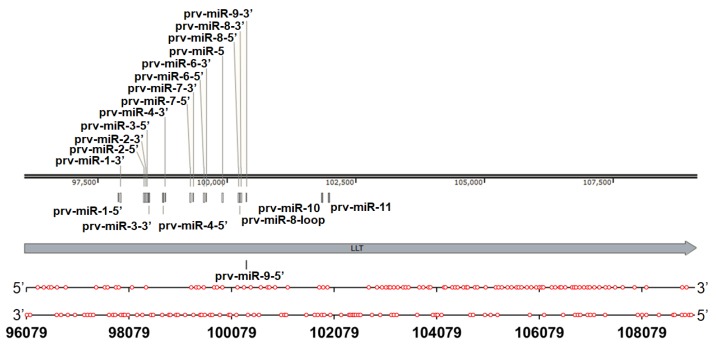
Distribution of G_2_-PQSs in the double-strands of the PRV large latency transcript region. One red circle indicates one G_2_-PQS.

The large latency transcript was the only gene reported to be transcribed during PRV latency [10]. It overlapped with the oppositely transcribed *IE180* gene and *EP0* gene, and it was one of the spliced transcripts in the PRV genome [9]. Sixty-seven G_2_-PQSs and 72 G_2_-PQSs were predicted, respectively, in the positive strand and in the negative strand of the LLT (Figure 8). Forty G_2_-PQSs were found to be 100% conserved among the annotated PRV genomes (Table S2). Thirty G_2_-PQSs in the negative strand were overlapped with the G_2_-PQSs in the *EP0* and *IE180* transcripts, suggesting that these overlapped G_2_-PQSs could be potential dual-functional regulatory elements. Twenty-seven G_3_-PQSs were found in the LLT transcription region (Figure S3), and only one G_3_-PQS was overlapped with *EP0* transcript, while the *IE180* transcript did not have any overlapped G_3_-PQS. The LLT intron had a microRNA cluster that was involved in PRV replication and affecting virulence [32,33]. Fifteen G_3_-PQSs and 35 G_2_-PQSs were found to be located in the intron of LLT. Thus, it could be speculated that these PQSs in the intron might be involved in the transcription regulation of microRNAs during PRV latency.

##### Density of the G_2_-PQSs in the Promoter Regions

The G_3_-quadruplexes in the promoter regions of Human immunodeficiency virus 1 (HIV-1) and human herpesviruses were reported to negatively regulate gene expression [5,34,35,36]. Since the promoters of PRV genes were not annotated in the reference genome, we predicted the promoters to be 1 kb upstream of the transcription start site of each gene. The gene *UL14* coding tegument protein and the gene *UL28*, coding DNA packaging terminase subunit 2, were found to contain nine G_2_-PQSs in their promoters, respectively, which was the largest number among all of the PRV genes. Viral DNA replication-related genes *UL29*/*UL30*/*UL8* had eight G_2_-PQSs in each promoter (Figure 6).

#### 2.1.4. Comparison of PQS Distribution among Three Herpesvirus Genomes

Inter-species comparison was performed among PRV, HHV-1 (Herpes simplex virus type 1, HSV-1) and HHV-3 (Varicella-zoster virus, VZV). These three viruses were neurotropic alphaherpesviruses. PRV and HHV-3 belonged to the same genus called *Varicellovirus*. These herpesviruses always caused serious diseases after reactivation from latency. Trigeminal ganglia (TG) was the common latency site for these three herpesviruses, and the dorsal root ganglia (DRG) was another latency site for HHV-3 [10,25,37]. It was difficult to control the switch between latency and reactivation of these herpesviruses. An analysis of the common features and differences in the PQS distribution among these herpesviruses will provide an insight into latency modulation.

G_3_-PQSs with the highest density among three herpesviruses were located in the repeat regions (Table 1) related to genome recombination during infection. Though they were in different genuses, PRV and HHV-1 shared similar features in the distribution and high density of G_2_-PQSs, which were different from HHV-3. G_2_-PQSs with the highest density in PRV and HHV-1 were located in the coding regions. The density of G_2_-PQSs in the coding regions of PRV and HHV-1 genes was found to be five-fold as much as that of HHV-3 (Table 1). In the PRV and HHV-1 genomes, the number of the G_2_-PQS monomers in the 3′ UTR regions was 8–12 fold of that of the 5′ UTR region. The latency-associated transcripts in HHV-1 and PRV were much longer than that in HHV-3. In PRV, the densities of G_2_-PQSs and G_3_-PQSs were 10.66 G_2_-PQS/kb and 2.07 G_3_-PQS/kb in its LLT, respectively. In HHV-1, it was 9.91 G_2_-PQS/kb and 3.39 G_3_-PQS/kb in its LAT, while in HHV-3, no G_3_-PQS was observed in its VLT, and the density of G_2_-PQS was half that of PRV (Table 1). Compared with HHV-1, the PRV had more G_3_-PQS monomers distributed in the repeat region, but less G_3_-PQS monomers in the CDS, UTR, promoter, and LLT regions (Table 1).

#### 2.1.5. Summary of Bioinformatics Analysis

In herpesvirus genomes, G_3_-PQSs were especially abundant in the repeat regions, while G_2_-PQSs were distributed genome-wide. The non-coding region had many more G_2_-PQSs than G_3_-PQSs, and G_2_-PQSs showed a higher density in the CDS region and LLT than in the repeat region. The genes involved in transactivation, genomic DNA replication, and the virus maturation processes had rich and conserved G_2_-PQSs in their mRNA sequences. In the *Varicellovirus* genus, some G_2_-PQSs in the coding sequences of the immediate early protein ICP4, DNA cleavage and packaging protein (*UL33*), and serine/threonine kinase (*UL13*) exhibited conservations of 100%. The highly conserved G_2_-PQSs provide universal target sites to control *Varicellovirus* by disturbing the translation of the above proteins. The density of G_2_-PQS in 3′ UTR was higher than that in the 5′ UTR, and more G_2_-PQS clusters were found in the 3′ UTR of PRV genes than those in the 5′ UTR. The 38 G_2_-PQSs in the repeated region between *IE180* and *US1* exhibited conservations of higher than 90% among all the annotated PRV genomes, indicating that the conserved elements might be involved in PRV genome recombination. The LLT overlapped with the oppositely transcribed *IE180* gene and the *EP0* gene. The PQSs in the overlapped regions might be potential dual-functional regulatory elements.

### 2.2. Experimental Validation

The genome-wide analysis of the G_2_-PQS distribution indicated that one-third of the PRV G_2_-PQSs were distributed in the non-coding regions of PRV genome, such as the regions of UTR, IRS, TRS, and LLT (File S1). Further study is needed to determine whether these PQSs could serve as *cis*-regulatory elements in gene expression.

#### 2.2.1. Parallel G-Quadruplexes formed by G_2_-PQSs In Vitro

**Figure 9:**
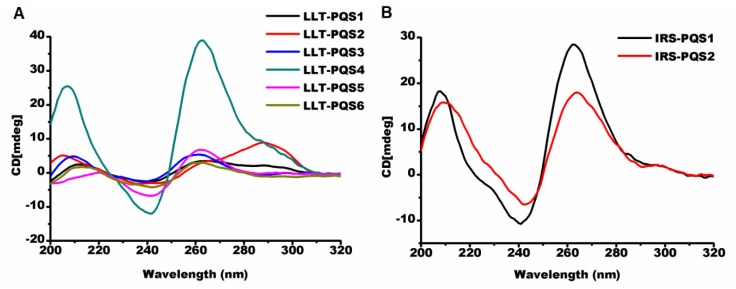

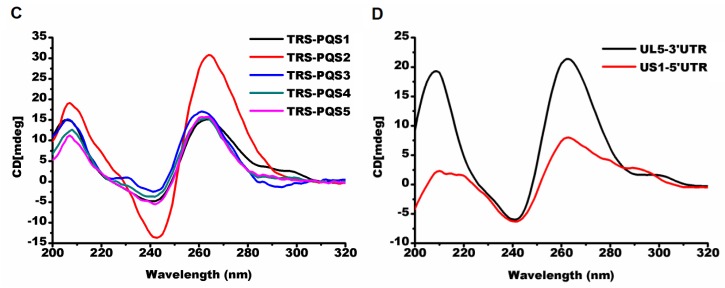
Circular dichroism (CD) spectra of G-quadruplex structures formed by selected oligonucleotides. (**A**) G_2_-PQS from the large latency-associated transcript (LLT) region. The LLT-PQS1 and LLT-PQS2 are located between the start site of LLT and the Prv-miR-1-5′; the LLT-PQS3, LLT-PQS4, LLT-PQS5, and LLT-PQS6 are located between Prv-miR-11-1 and the end of the LLT. (**B**) G_2_-PQS from the internal repeat sequence (IRS) region. The IRS-PQS1 and IRS-PQS2 are located between *IE180* and *US1*. (**C**) G_2_-PQS from the terminal repeat sequence (TRS) region. TRS-PQS1, TRS-PQS2, TRS-PQS3, and TRS-PQS4 are located in the sequence complementary to *US1* CDS; TRS-PQS5 is located between *IE180* and *US1*. (**D**) G_2_-PQS from the untranslated region (UTR). The UL5-3UTR is from the 3′ UTR of the gene *UL5*; US1-5UTR is from the 5′ UTR of the gene *US1*.

Circular dichroism (CD) spectroscopy is widely used to distinguish parallel, anti-parallel or hybrid G-quadruplex structures [38]. A parallel G-quadruplex showed a negative peak near 240 nm, and a positive peak near 260 nm. An anti-parallel G-quadruplex exhibited a negative peak near 260 nm and a positive peak around 290 nm. The hybrid G-quadruplex always displayed one negative peak at 240 nm and two positive peaks at 260 nm and 290 nm. Of the 15 G_2_-PQS oligonucleotides (details in Table S3) selected from the regulatory regions of the PRV genome, 13 G_2_-PQS folded into parallel G-quadruplexes in a buffer containing 100 mM potassium. LLT-PQS1 and LLT-PQS2, between the start of LLT and the Prv-miR-1-5′, formed hybrid G-quadruplexes, while other LLT-PQSs between Prv-miR-11-1 and the end of LLT formed parallel G-quadruplexes (Figure 9A). The three G_2_-PQSs were located in the repeat region between *IE180* and *US1*, and the five G_2_-PQSs were located in the repeat region complementary to *US1* CDS, and all of these eight G_2_-PQSs mentioned above formed parallel G-quadruplexes (Figure 9B,C). One G_2_-PQS from the 3′ UTR of *UL5*, and another from the 5′ UTR of *US1* exhibited typical parallel G-quadruplex peaks (Figure 9D).

#### 2.2.2. G_2_-quadruplexes in the 3′ UTR Affects Gene Expression In Vivo with Varying Sensitivities 

The formation of a G-quadruplex in the 3′ UTR was reported to result in gene expression decrease, alternative polyadenylation [39], and retrotransposition [40].This study found that there were more G_2_-PQSs in the 3′ UTR than in the 5′ UTR, in PRV (Table 1). In this study, G_2_-PQSs from the 3′ UTR were inserted into the dual luciferase vector, to check their functions.

**Figure 10:**
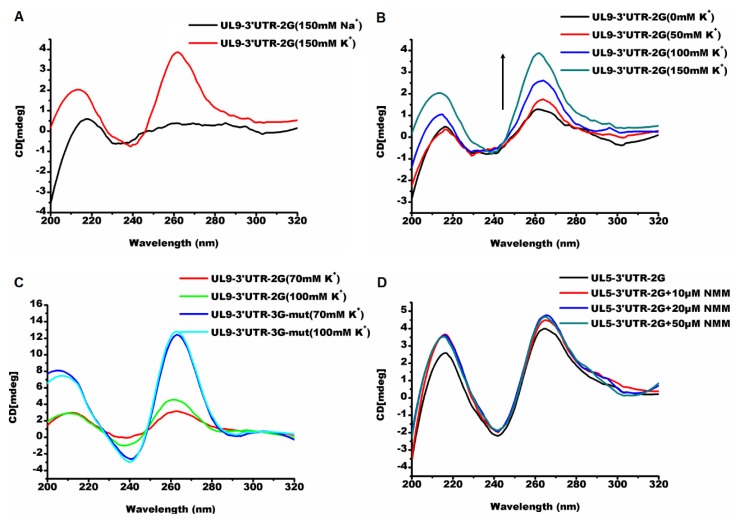
G_2_-PQSs from UL5-3′UTR and UL9-3′UTR are sensitive to cations and ligands. (**A**) CD spectroscopy of the G_2_-PQS (UL9-3′UTR-2G) in the presence of 150 mM NaCl or KCl. (**B**) CD spectroscopy of the G_2_-PQS (UL9-3′UTR-2G) with increasing KCl concentration (0–150 mM). (**C**) CD spectroscopy of G_2_-PQS (UL9-3′UTR-2G) and the G_3_-PQS mutant (UL9-3′UTR-3G-mut) with increasing KCl concentration (70–100 mM). (**D**) CD spectroscopy of the G_2_-PQS (UL5-3′UTR-2G) with increasing NMM concentration (10–50 μM).

**Figure 11:**
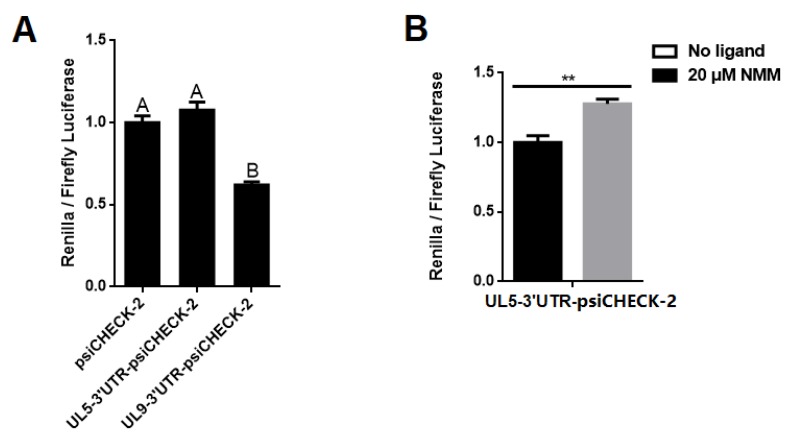
G_2_-PQSs from UL5-3′UTR- and UL9-3′UTR-regulated gene expression. (**A**) Dual-luciferase assay of G_2_-PQS from *UL5* and *UL9* in gene expression regulation. Different uppercase letters indicate the significant differences among the different constructs (*p* < 0.05, Tukey’s honestly significant difference (HSD) test) (**B**) Dual-luciferase assay of G_2_-PQS from *UL5* in gene expression regulation with the ligand NMM. ** *p* < 0.01, Student *t*-test.

In the Circular dichroism (CD) experiment, the PQS UL9-3′UTR-2G exhibited no G-quadruplex signal in 100 mM sodium solution, while it folded into a parallel G-quadruplex in the buffer containing 100 mM potassium (Figure 10A). The stability of the G-quadruplex increased with the addition of the potassium cation. (Figure 10B). UL9-3′UTR-2G showed a higher sensitivity to the increase of the potassium than the 3G mutant oligonucleotide obtained by replacing “GG” with “GGG” (Figure 10C). The insertion of PQS UL9-3′UTR-2G into the 3′ UTR region of the renilla luciferase gene in the psiCHECK-2 vector resulted in a 38% decrease in relative luciferase activity (Figure 11A). The PQS UL5-3′UTR-2G folded into a parallel G-quadruplex in a buffer with 100 mM K^+^ (Figure 10D), and the addition of the G-quadruplex ligand NMM stabilized the structure. The insertion of PQS UL5-3′UTR into the 3′ UTR region of renilla luciferase gene in the psiCHECK-2 vector made no difference in the protein expression (Figure 11A). The addition of NMM at 20 µM increased the relative luciferase activity (*p* < 0.01) (Figure 11B, Figures S5). The addition of other typical G-quadruplex ligands such as PDS and BRACO-19 made no significant difference in structural stability (Figures S5, S6A, S6B) and relative luciferase activity (Figures S5, S7A, S7B). The above results suggested that the G-quadruplex formed from G_2_-PQSs in 3′ UTR affected the protein expression, and that G_2_-PQSs were more sensitive to different cations and ligands than G_3_-PQSs. These features made the G_2_-quadruplex suitable as sensitive switches for gene expression regulation in response to different environmental factors, such as various proteins, small molecules, and signal cations.

#### 2.2.3. The G-Quadruplex Ligand Decreases the Virulence of PRV

G-quadruplex ligands were reported to interfere with biological processes related to tumor growth, by binding, stabilizing, converting, or unwinding G-quadruplex structures [41,42,43]. NMM at a series of concentrations (150 nM, 100 nM, 50 nM) was employed to treat the PRV-infected PK15 cells for 24 h. The plaque assay indicated that the NMM had the potential for reducing the virus titer at 24 h post-treatment (Figure S8).

## 3. Discussion

A high density of G_2_-PQSs was distributed widely in the genomes of PRV, HHV-1, and HHV-3, and they could provide sensitive regulatory switches in response to environmental factors in gene expression regulation. Compared to the G_3_-quadruplexes, the G_2_-quadruplexes exhibited less thermal stability and higher sensitivity to loop size and compositions, and they presented multiple conformations in different solutions [44]. As they were sensitive to the microenvironment in the cells, the G_2_-quadruplexes could act as the receptors of specific proteins or metabolites in the cells, to identify the cells that are suitable for PRV latency. In this study, PQS UL9-3′UTR-2G exhibited a parallel structure in potassium solution, while it could not fold into a G-quadruplex in a sodium cation solution (Figure 9A). The insertion of PQS UL9-3′UTR-2G into the psiCHECK-2 vector led to the decrease in reporter gene expression, while the insertion of PQS UL5-3′UTR-2G resulted in an increase in gene expression only with the addition of the ligand NMM (Figure 11, Figure S4). The G-quadruplex structural sensitivity enabled the virus to be easily mediated by physiological cations, different small molecules, or proteins. These findings are conducive to revealing the latency–reactivation mechanism of PRV in specific tissues under certain conditions.

Highly conserved G_2_-PQSs were discovered in the coding regions of genes related to virus replication and maturation, in the *Varicellovirus* genus. The formation of the RNA G_2_-quadruplex in the open reading frame of EBNA1 of EBV resulted in a downregulation of the expression level of the maintenance protein, facilitating virus escape from immune recognition [7]. The conserved G_2_-PQSs in the *Varicellovirus* genus were located in the unique immediate early protein (*IE180*), viral DNA replication protein (*UL30*), DNA cleavage and package proteins (*UL33*/*UL17*), and tegument proteins (*UL16*/*UL47*). *IE180* had five conserved G_2_-PQSs among all the *Varicellovirus* (Table 2). *IE180* was found to be a unique immediate early gene of PRV and the first viral gene transcribed during PRV infection. *IE180* is reported to mediate latency and reactivation by regulating early genes, and it activates *US4*, *UL12* (alkaline exonuclease), *UL22* (type I membrane protein), *UL23* (thymidine kinase), and *UL41* (RNAse) [10]. Based on previous reports, we speculated that the formation of the G-quadruplex in the CDS region of *IE180* would result in a reduction of immediate early protein levels, disturbing downstream gene expression in the life-cycle of PRV. Therefore, it could be further inferred that the G_2_-quadruplex might have a significant regulatory effect on the *Varicellovirus* genus, especially for the immediate early protein ICP4, the homolog of immediate early protein from PRV.

A high density of G_2_-PQSs in the untranslated regions of the PRV genes could provide cis-elements to regulate the post-transcription and translation processes. Though the G_3_-quadruplexes in 3′ UTR and 5′ UTR were widely reported to have regulated human gene translation [5,29,30,31,45], few reports on the G-quadruplexes in the untranslated regions of human herpesvirus genes are available. In this study, many G_2_-PQSs were found in the untranslated regions of herpesvirus genes (Figure 7). Various G-quadruplexes formed by those G_2_-PQSs regulated gene expression diversely, either under physiological conditions or with a stabilizing ligand (Figure 11). The data suggested that the formation of these G_2_-quadruplexes might result in the truncated 3′ UTR, and in turn, disturb 3′-end polyadenylation, finally leading to unstable virus mRNA. The G-quadruplex in the 3′ UTR was also reported to have played a role in regulating microRNA binding in humans [46,47], suggesting that the G-quadruplex might be involved in the interaction between virus/host microRNAs and virus genes.

A large number of G_2_-PQSs and G_3_-PQSs in the repeat regions of the herpesvirus genomes might play an important role in genomic integration or recombination during herpesvirus latency. Some conserved G_3_-PQSs in herpesviruses were reported to have played important role in virus integration, latent DNA replication, and episomal persistence. Several herpesviruses, like Marek’s Disease virus (MDV) [48], gallid herpesvirus 2 (GaHV-2) [49], and human herpesvirus 6 (HHV6) [50], were found to establish latent infections, with viral genomes integrated into telomere repeat tracts in host chromosomes through homologous recombination. In this study, the pseudorabies virus had 205 G_3_-PQSs in the genome, with 43.9% located in the repeat region (File S1). A high density of conserved G_2_-PQSs existed in the repeat regions between the two diverging transcripts *IE180* and *US1*, which were close to the origin of replication (OriS) (Figure 5). Telomeres were reported to form G-quadruplex clusters with a repeated sequence of (GGGTTA)n [51]. This study found that the PRV genome had imperfect telomeric repeats, with the unit sequence 5′-GGGGTGGAGACGGTGGAGGGAGAGGGGAGTGGG-3′ repeated 12 times. Thus, these G_2_-PQSs were speculated to be related to genomic recombination and integration during latency. 

## 4. Methods and Materials

### 4.1. Virus Sequences

The complete genome sequence of the Suid herpesvirus 1 (Pseudorabies virus, PRV) (NC_006151.1) was retrieved from NCBI Genome database [24]. It was annotated into 12 features, including 69 CDS regions, 70 genes, four introns, 119 misc. feature regions, one misc. RNA, 20 polyA sites, 14 protein binding sites, 117 regulatory region, 28 repeat regions, seven stem loop regions, one sequence tagged sites (STS), and one variation. The UTR was determined through alignment between the CDS sequence and the gene sequence. The sequences included in the gene sequences and located upstream of the CDS sequence were designated as 5′ UTRs, and the sequences downstream of the CDS were defined as 3′ UTRs. The putative G-quadruplex sequences (PQS) were searched in the standard genome sequence of PRV. The number of PQSs in the CDS, 3′ UTR, 5′ UTR and repeat regions was counted, respectively.

The standard genome sequences of the *Varicellovirus* genus retrieved from the NCBI Genome database [24] for conservation analysis were Human herpesvirus 3 (NC_001348.1), Equid herpesvirus 1 (NC_001491.2), Equid herpesvirus 3 strain AR/2007/C3A (NC_024771.1), Equid herpesvirus 4 (NC_001844.1), Equid herpesvirus 8 (NC_017826.1), Equid herpesvirus 9 (NC_011644.1), Bovine herpesvirus 1 (NC_001847.1), Bovine herpesvirus 5 (NC_005261.2), Suid herpesvirus 1 (NC_006151.1), Cercopithecine herpesvirus 9 (NC_002686.2), and Felid herpesvirus 1 (NC_013590.2).

### 4.2. Identification of Putative G-Quadruplex Sequences in the PRV Genome

The putative G_3_-quadruplex sequences were searched by Quadparser software [3]. The formula of the putative G_2_-quadruplex sequence was defined as G_2+_N_1-7_G_2+_N_1-7_G_2+_N_1-7_G_2+_, where G is guanine, and N is any nucleotide including G. With the same software, the putative I-motif sequences were searched with the formula C_2+_N_1-7_C_2+_N_1-7_C_2+_N_1-7_C_2+_, where C is cytosine, and N is any nucleotide including C. The Quadparser coded the sequence in the format *x*:*y*:*z*, where *x* stands for the number of guanine tracts or C-runs, *y* stands for the number of locations of putative G4 or I-motif formations, and *z* stands for the number of possible simultaneous G4- or I-motif structures.

The number of I-motif sequences in the sense strand was the same as the number of G_2_-PQSs in its complementary strand. The sum of the G_2_-PQSs and I-motif sequences in the sense strand was the total number of G_2_-PQSs in the double-stranded genome of PRV. The density of G_2_-PQS in the PRV standard genome was calculated by dividing the total number of G_2_-PQSs in the double-stranded genome of PRV by the genome size. If *z* = 1, the G_2_-PQS was counted as a PQS monomer. If *z* ≥ 2, the G_2_-PQS was counted as a PQS cluster. 

### 4.3. Comparison of the Distribution of G_2_-PQS and G_3_-PQS between PRV and Two Herpesviruses

The G_3_-PQS preferred to be located in the repeat regions in human herpesvirus genomes and some repeated G_3_-PQS clusters among the analyzed genomes were reported to be conserved [5]. In order to determine the distribution features of PQSs in PRV genome, we compared PRV, Human herpesvirus 1 (HHV-1), also known as Herpes simplex virus type 1 (HSV-1), and Human herpesvirus 3 (HHV-3), also known as Varicella-zoster virus (VZV). The reference genome sequences of PRV (NC_006151.1), HHV-1 (NC_001806.2) and HHV-3 (NC_001348.1) were downloaded from NCBI [24] and saved as FASTA files. The genome features of viruses above were analyzed with the software BEDTools (https://github.com/arq5x/bedtools2) [52] and the length of CDS region, 3′ UTR region, 5′ UTR region, repeat region, and latency associate transcript was recorded. The promoter region of each gene was predicted as 1kb upstream of the transcription start site of each gene. The PQSs in CDS, latency associate transcript, and repeat regions were counted in both positive strand and negative strand, and the PQSs in the untranslated regions and promoters were counted in terms of the transcription direction of each PRV gene. The number of G_3_-PQS monomer, G_3_-PQS cluster, G_2_-PQS monomer and G_2_-PQS cluster was counted with the Quadparser software. Quadparser was modified as described in last section. The density of PQSs was calculated by dividing the total number of PQSs located in each region or gene by the length of the corresponding region or gene. 

### 4.4. Conservation of Putative G-Quadruplex Sequences and I-Motif Sequences in the PRV CDS Region in the Varicellovirus Genus

The reference genome sequences of 11 virus species from the *Varicellovirus* genus were downloaded from NCBI. All the PQS and I-motif sequences in the genome of viruses from *Varicellovirus* genus were searched with Quadparser software and output into file g4_cds.txt (File S3). The protein sequences in each genome were listed in file common_protein.txt (File S4). Following data preparation, CDS and amino acid sequences of all proteins in the eleven virus species were used for multiple sequence alignment with MAFFT software (https://www.ebi.ac.uk/Tools/msa/mafft/). The identity of the above nucleotide sequences and amino acid sequences of each protein was calculated with infoalign program of the EMBOSS package (http://emboss.sourceforge.net/apps/release/6.6/emboss/apps/infoalign.html). The PQS sequences (GG**GG**GG**GG) and I-motif sequences (CC**CC**CC**CC) from the multiple alignment result of nucleotide sequences were identified and counted, and then the ratio of the common PQSs and I-motif sequences to the nucleotide sequences of all the proteins was calculated and output as conservation score of each PQS and I-motif sequence. The conservation of PQS in PRV CDS in the *Varicellovirus* genus was analyzed. 

### 4.5. Percentage Conservation of Putative G-Quadruplex Sequences in the Repeat Regions and LLTs of the PRV Genomes

Twenty-five PRV complete genome sequences from different strains were downloaded from the NCBI Genome database [24]. These sequences were Suid herpesvirus 1 (NC_006151.1, KU056477.1, BK001744.1), Suid alphaherpesvirus 1 isolate Ea (Hubei) (KX423960.1), Suid alphaherpesvirus 1 isolate LA (KU552118.1), Suid herpesvirus 1 strain NIA3 (KU900059.1), Suid herpesvirus 1 isolate DL14/08 (KU360259.1), Suid herpesvirus 1 strain Ea (KU315430.1), Suid herpesvirus 1 strain ADV32751/Italy2014 (KU198433.1), Suid herpesvirus 1 strain Kolchis (KT983811.1), Suid herpesvirus 1 isolate HLJ8 (KT824771.1), Suid herpesvirus 1 strain HN1201 (KP722022.1), Suid herpesvirus 1 strain HNB (KM189914.3), Suid herpesvirus 1 strain Fa (KM189913.1), Suid herpesvirus 1 strain Kaplan (KJ717942.1, JF797218.1, JQ809328.1), Suid herpesvirus 1 strain HNX (KM189912.1), Suid herpesvirus 1 isolate SC (KT809429.1), Suid herpesvirus 1 strain JS-2012 (KP257591.1), Suid herpesvirus 1 strain HeN1 (KP098534.1), Suid herpesvirus 1 isolate ZJ01 (KM061380.1), Suid herpesvirus 1 strain TJ (KJ789182.1), Suid herpesvirus 1 strain Becker (JF797219.1), and Suid herpesvirus 1 strain Bartha (JF797217.1). All of the putative G-quadruplex sequences located in the repeat regions or LLT of the reference genome sequence (Accession Number: NC_006151.1) were searched from the other 24 genome sequences, and the percentage conservation was calculated through dividing the number of genome sequences containing the same putative G-quadruplex sequences by the total number of genome sequences. 

### 4.6. Oligonucleotide Folding Conditions

All oligonucleotides purchased from Sangon Biotech, Shanghai, China were salt-free, purified, and dissolved in ddH_2_O to a concentration of 100 μM. Oligonucleotide sequences of PQS in regulatory region were selected from the PQSs predicted by Quadparser (Table S3). G_2_-PQSs from the *UL5* 3′ UTR, *UL9* 3′ UTR, and mutant sequences (Table S4) were folded under the same conditions, as follows. Oligonucleotides were diluted to 10 μM in 10 mM phosphate buffer at pH 7.0 supplemented with 100 mM KCl, then they were heated to 95 °C for 5 min in a 1.5 mL Eppendorf tube in water bath, and subsequently slowly cooled for ~8 h to room temperature, then used for spectra or stored at 4 °C.

Under the induced folding conditions for G_2_-PQS from *UL9* 3′ UTR, the G_2_-PQS was diluted to 10 μM in 10mM Tris-HCl buffer at pH 7.0, supplemented with an increasing concentration of KCl (50 mM, 100 mM, and 150 mM). The G_2_-PQS in 10mM sodium phosphate buffer at pH 7.0 supplemented with 100 mM NaCl was tested. These samples were placed at room temperature for 30 min, and then applied to CD spectra.

Under the induced folding condition for G_2_-PQS from *UL5* 3′ UTR, the G_2_-PQS was diluted to 10 μM in 10 mM phosphate buffer at pH 7.0, supplemented with 100 mM KCl, and then incubated with different ligands at room temperature for 30 min before CD spectroscopy.

### 4.7. Circular Dichroism Spectroscopy

CD spectra of the 10 μM folded oligonucleotide samples were collected at 25 °C on a JASCO 1500 CD spectrometer by using a quartz cuvette with 1 mm optical path. Data within a 200–320 nm range were collected using two scans at 100 nm/min with 1 s settling time and 1 nm bandwidth. The buffer baseline was recorded with the same parameters, and it was subtracted from the sample spectra before plotting. 

### 4.8. Plasmid Construction

The plasmids UL5-3′UTR-psiCHECK-2 and UL9-3′UTR-psiCHECK-2 were constructed by using specific oligonucleotides (Table S5), with the ClonExpress II One Step Cloning Kit (Vazyme, Nanjing, China) according to the manufacturer’s instructions. 

### 4.9. Cell Culture 

Cell lines, human embryonic kidney (HEK) 293T, porcine kidney cell (PK-15), and bovine kidney cells (MDBK) were provided by the State Key Laboratory of Agricultural Microbiology, College of Veterinary Medicine, Huazhong Agricultural University in China. The above cell lines were cultured in Dulbecco’s modified Eagle medium (DMEM) containing 10% fetal bovine serum (FBS), 0.044 M NaHCO_3_, and 0.025 M HEPES. Cells were grown at 37 °C in a humidified atmosphere with 5% CO_2_. 

### 4.10. Cytotoxicity Assay

The cytotoxicity of the G-quadruplex ligands, *N*-methyl mesoporphyrin IX (NMM) [53] (J&K Scientific, Beijing, China), BRACO-19 [54] (Sigma-Aldrich, Saint Louis, MO, USA), and pyridostatin (PDS) [55] (J&K Scientific, Beijing, China) to HEK293T was determined by the MTT assay, which is dependent on the measurement of mitochondrial dehydrogenase enzyme activity of viable cells. MTT, 3-(4,5-dimethylthiazol-2-yl)-2,5-diphenyltetrazolium bromide, is a yellow tetrazole, and it can be reduced to a purple formazan in living cells. The HEK293T cells, at a density of 1 × 10^4^ cells per well were seeded into a 96-well microplate. When the cell confluence reached 90%, the growth medium was replaced with 100 μL of DMEM containing 2% FBS and G-quadruplex ligands at a series of final concentrations (Figure S5), and the cells were incubated at 37 °C for 24 h. Then, 50 μL of MTT solution was added into each well, and the cells were incubated at 37 °C for another 4 h. After incubation, the supernatant was removed and 150 μL of dimethyl sulfoxide (DMSO) was added into each well to dissolve the formazan. The relative cell viability was analyzed by measuring the absorbance of formazan at 570 nm on the Synergy^TM^ HTX microplate reader (BioTek, Winooski, VT, USA). The cytostatic concentration, which will be applied in the subsequent dual luciferase assays, was required to maintain a cell viability of more than 90%. 

### 4.11. Transfection and Dual Luciferase Assays

The HEK293T cells were seeded in the 24-well plates at the concentration of 1 × 10^5^ cells/well. The cells were transfected with 0.8 μg of psiCHECK-2 reporter plasmids and Lipofectamine 2000 (Thermo Fisher Scientific, Carlsbad, CA, USA) according to the manufacturer’s instructions. NMM at 20 μM, BRACO-19 at 10 μM, or PDS at 10 μM was respectively added to the cells, with the medium being replaced at 6 h after transfection. The activities of firefly and renilla luciferase were measured 24 h after addition of the above G-quadruplex ligands, using the Dual-Luciferase Reporter Assay Kit (Promega, Madison, WI, USA) on a GloMax 20/20 luminometer (Promega, Madison, WI, USA).

### 4.12. Plaque Assay

The antiviral activity of NMM to PRV Ea strain was examined through plaque assay. The PK15 cells at the density of 1.2 × 10^6^ cells per well were seeded in the 6-well plate. When cell confluence reached 80–90%, the cells were placed in 4 °C for 1 h, and then the PRV Ea strain virus solution (MOI = 5) was added into each well. Then, the cells were placed back to 4 °C for 2 h, and the plate with cells was shacked once every 15 min during the incubation. After the incubation at 4 °C, the virus solution was removed and replaced with 2 mL of DMEM supplemented with NMM at different final concentrations (150 nM, 100 nM, 50 nM). Afterwards, the cells were incubated at 37 °C for 24 h. After incubation, the infectious viral particles were isolated from each well and prepared for plaque assay. 

MDBK cells at the density of 2 × 10^5^ cells per well were seeded in the 12-well plate. When the cell confluence reached 90%, the cells were used for the plaque assay. Exactly 200 μL of virus solution was added into each well. Afterwards, the cells were incubated at 37 °C for 2 h. Then, the infected cells were covered by 4% sodium carboxymethylcellulose (CMC-Na) supplemented with 3% FBS and 1% penicillin–streptomycin solution. After incubation at 37 °C for another 48 h, the infected cells were fixed and stained with the crystal violet solution (0.35%, *w/v* in ethanol) at room temperature. After 15 min, the crystal violet solution was removed, and the plate was washed with tap water. Then, the plate was put in the dry oven for a few minutes. Finally, the viral titer was determined by the plaque assay.

### 4.13. Statistical Analysis

A Student’s *t*-test was used to determine the significant differences in luciferase activity between the ligand treatment and control of each construct. One-way analysis of variance (ANOVA) with Tukey’s multiple comparison was applied to determining the significant difference among various treatments dual luciferase assay.

## 5. Conclusions

In summary, the systematic analysis of the distribution of G_2_-PQS in the PRV genomes provides a clear guide for elaborated studies of their functions, related to the establishment of latency and its reactivation in herpesviruses. We analyzed the putative G_2_-quadruplex and G_3_-quadruplex sequences in the PRV genome systematically, and then compared it with typical human herpesviruses in the same subfamily, then evaluated the structures and functions of G_2_-quadruplexes. G_2_-quadruplex sequences in the form of both monomers and clusters were found to be distributed in the entire PRV genome, especially in the CDS, LLT, and repeat regions. Extremely conserved G_2_-quadruplex sequences existed in the CDS of the genes related to viral genome replication and maturation processes. G_2_-quadruplex sequences tended to be located in the repeat regions close to the origin of replication site, which may contribute to genome replication and recombination. Most G_2_-quadruplexes from the regulatory regions formed parallel-type G-quadruplex. There were more G_2_-quadruplex sequences in the 3′ UTR regions than in the 5′ UTR regions. These G_2_-quadruplexes showed different sensitivities to physiological cations and small molecules. Thus, it could be inferred that the G_2_-quadruplex could act as a switch to control the expression of genes involved in virus latency establishment, viral genome replication cascade, and virus cell-to-cell movement. The G-quadruplex ligand, NMM, exhibited the potential for inhibition of the proliferation of PRV in its host cells. These massive and sensitive G_2_-quadruplexes could serve as a class of receptors in response to intracellular environments, guiding herpesviruses to choose specific cells or conditions for latency or reactivation.

## Figures and Tables

**Table 1:** Number of putative G-quadruplex sequences in three herpesvirus genomes.

Herpesviruses	Region †	Length (bp)	Putative G_2_-quadruplex Sequences				Putative G_3_-quadruplex Sequences			
			Number	Monomer	Cluster	Density	Number	Monomer	Cluster	Density
						(PQS/kb)				(PQS/kb)
Pseudorabies virus (PRV, NC_006151.1)	coding sequence (CDS)	105,363	1126	1004	122	10.69	51	51	0	0.48
	3’ end untranslated region (3′ UTR)	35,688	166	149	17	4.65	6	6	0	0.17
	5’ end untranslated region (5′ UTR)	6760	19	18	1	2.81	5	3	2	0.74
	Promoter	56,324	301	276	25	5.34	36	32	4	0.64
	large latency transcript (LLT)	13,040	139	109	30	10.66	27	25	2	2.07
	Repeat region	36,234	338	269	69	9.33	131	109	22	3.62
Human alphaherpesvirus 1 (Herpes simplex virus type 1, HSV-1, NC_001806.2)	CDS	121,089	1351	1248	103	11.16	109	104	5	0.90
	3′ UTR	49,322	235	223	12	4.76	27	27	0	0.55
	5′ UTR	3434	18	18	0	5.24	3	1	2	0.87
	Promoter	64,966	346	317	29	5.33	64	56	8	0.99
	latency-associated transcript (LAT)	15,944	158	146	12	9.91	54	44	10	3.39
	Repeat region	31,875	338	307	31	10.60	120	87	33	3.76
Human alphaherpesvirus 3 (Varicella-zoster virus, VZV, NC_001348.1)	CDS	111,496	296	282	14	2.65	10	10	0	0.09
	3′ UTR	29,758	37	36	1	1.24	1	1	0	0.03
	5′ UTR *	496	0	0	0	0	0	0	0	0
	Promoter	60,900	71	69	2	1.17	5	3	2	0.08
	VZV latency-associated transcript (VLT)	2417	13	12	1	5.38	0	0	0	0
	Repeat region	15,514	119	111	8	7.67	11	9	2	0.71

†: The CDS, 3′ UTR, 5′ UTR and repeat regions were analyzed according to the annotation information in the reference genomes. The promoter regions were predicted as 1kb upstream of the transcription start site of each annotated gene. LLT: large latency transcript; LAT: latency-associated transcript; VLT: VZV latency-associated transcript [26]. *: The annotated 5′ UTR of the genes from the Human alphaherpesvirus 3 were analyzed for the PQSs.

**Table 2:** G_2_-PQS with high conservation score in the coding sequence of *Varicellovirus* genes.

Gene Common Name	Function	Putative G_2_-quadruplex Sequence (5′-3′)	Score	Nucleotide Identity
*IE180*	Transactivator, homolog of ICP4	GGGCCGGGAACTGGACCGGG	1.000	0.579
		GGACTGGCCCGCGGACGGCCCGGCCGTGGGGG	0.900	0.579
		GGCTCGGCGCGGCGCGGCGCCGG	0.800	0.579
		GGCCAACGTGGCCGCGGCCCGG	0.700	0.579
		GGGCCCCGGTCCCGGGCCGGCTCCGGGCCCCGG	0.700	0.579
*UL30*	DNA replication	GGACGACGGCGGCGGCTACCAGGGCGCCAAGG	0.727	0.678
		GGTGTACGGGTTCACGGGCGTGGCCAACGGG	0.727	0.678
*UL33*	DNA cleavage and packaging	GGGGAGGCGCTGCGGGCGCGG	1.000	0.664
*UL17*	DNA cleavage and encapsidation	GGGGCGGCCGGCGCGGGCCCCGG	0.778	0.589
*UL16*	Tegument protein	GGTCCTGGCCCCCGGCGCGTGGTGGGCGCGCGG	0.800	0.580
*UL47*	Tegument protein	GGGGACGAGGAGGAGGAGGAGGAGGAGGAGGAGAGCGAGGGGGGCGCGTGGTCCGACGGGG	0.700	0.486
*UL13*	Protein-serine/threonine kinase	GGCCGTCGGGGCCGGATCGTACGG	1.000	0.525

## Supplementary Materials

The following are available online.
