# Supplementary material for: Intensive Distribution of G2-Quaduplexes in the Pseudorabies Virus Genome and Their Sensitivity to Cations and G-Quadruplex Ligands
Source: Molecules. 2019 Feb 21;24(4):774. doi: 10.3390/molecules24040774 (PMC6412908; doi:10.3390/molecules24040774)
Supplement: Supplementary file 1 [file molecules-24-00774-s001.zip › Supporting information/File S3.pdf]

gi|9625875|ref|NC_001348.1| Human herpesvirus 3, complete genome

191 220 173 562 CDS product membrane protein UL56 191-220 6:03:01 CCCGCCGACCTGGGACCCCGCCGGTCACCC

254 288 173 562 CDS product membrane protein UL56 254-288 7:04:01 CCCCCATCCCCTACCTACCCACATACGCCGAGGCC

515 546 173 562 CDS product membrane protein UL56 515-546 5:02:01 CCATTGTTGCCGTTTTTCCCGAGGAACCTCCC

872 907 589 915 CDS product membrane protein V1 872-907 6:03:01 CCCGAACACCTTCCGGTACCCCATACTCCGATACCC

1708 1736 1185 1850 CDS product myristylated tegument protein CIRC 1708-1736 5:02:01 CCGACCCACTCTCCCAACCCTAACAGCCC

1974 1991 1908 2447 CDS product nuclear protein UL55 1974-1991 4:01:01 CCGGTCCAACACTCCGCC

3526 3548 2783 4141 CDS product multifunctional expression regulator 3526-3548 4:01:01 GGTCACGCTGGCTCCCGCGGTGG

5118 5138 4252 5274 CDS product envelope glycoprotein K 5118-5138 5:02:01 CCCCCATACAACCGGTCCACC

5472 5492 5326 8577 CDS product helicase-primase primase subunit 5472-5492 4:01:01 CCGGGGAGCCGTTCTTCCTCC

5947 5960 5326 8577 CDS product helicase-primase primase subunit 5947-5960 4:01:01 CCAACCGCCGTCCC

7125 7150 5326 8577 CDS product helicase-primase primase subunit 7125-7150 5:02:01 GGGTGGATTTGAGGGAATAGTGGAGG

8692 8720 8607 9386 CDS product tegument protein UL51 8692-8720 7:04:01 CCCCCAAGGAGCCGCCCTGCTCCGCCTCC

8989 9002 8607 9386 CDS product tegument protein UL51 8989-9002 4:01:01 GGTTGGCGCGGTGG

12579 12598 12160 13392 CDS product transactivating tegument protein VP16 12579-12598 4:01:01 CCTAGTCCACCAAAGCAACC

13320 13367 12160 13392 CDS product transactivating tegument protein VP16 13320-13367 10:07:02 CCTCCGTCCCCATCCGAGATCCTGCCGGGGGACCCACCACGCCCACCC

14341 14366 13590 16049 CDS product tegument protein VP13/14 14341-14366 4:01:01 GGTTATGGAGCCTGCGGTATCAAAGG
14812 14833 13590 16049 CDS product tegument protein VP13/14 14812-14833 4:01:01 GGGCCAAGGAACGGCCATTTGG
15095 15121 13590 16049 CDS product tegument protein VP13/14 15095-15121 4:01:01 GGTTTTTTTGGGGCGTTGGCATATTGG
15944 15967 13590 16049 CDS product tegument protein VP13/14 15944-15967 5:02:01 CCCATTCCCTCCGTGGACCTAGCC
16396 16423 16214 18199 CDS product tegument protein VP11/12 16396-16423 6:03:01 GGCGAGGCTTTGGAGACGGCGGTAAAGG
16621 16639 16214 18199 CDS product tegument protein VP11/12 16621-16639 4:01:01 GGGGATGGACTTTGGTGGG
16751 16784 16214 18199 CDS product tegument protein VP11/12 16751-16784 5:02:01 CCGTAGTTGCCAATAAACCAATATCCAGACATCC
16882 16907 16214 18199 CDS product tegument protein VP11/12 16882-16907 4:01:01 CCAGATGATCCTACAAACCCCAGCCC
16938 16999 16214 18199 CDS product tegument protein VP11/12 16938-16999 9:06:02 GGCTTACACGGCTACTGGCTACGGGTGGATGTTATGGTTCTTGGACGTTGTGGACGCCAGGG
17091 17117 16214 18199 CDS product tegument protein VP11/12 17091-17117 4:01:01 GGGTCCTGCGGTCTCAGCGGGCACAGG
17186 17219 16214 18199 CDS product tegument protein VP11/12 17186-17219 6:03:01 GGCGAAAGGAAGAGTGGCGGGATGGTTTAAATGG
17318 17330 16214 18199 CDS product tegument protein VP11/12 17318-17330 4:01:01 GGGGGGATGGGGG
17635 17656 16214 18199 CDS product tegument protein VP11/12 17635-17656 4:01:01 GGGACAGGTATAGGGAATTTGG
17835 17855 16214 18199 CDS product tegument protein VP11/12 17835-17855 4:01:01 GGACATAGGCCATACGGACGG
19051 19070 18441 19346 CDS product thymidylate synthase 19051-19070 4:01:01 GGGGGATATGGGCCTTGGGG

19218 19238 18441 19346 CDS product thymidylate synthase 19218-19238 4:01:01 CCCCAAAACCTTTTCCTTGCC
19310 19322 18441 19346 CDS product thymidylate synthase 19310-19322 4:01:01 CCACACCCCCCCC
19903 19919 19431 21113 CDS product envelope glycoprotein C 19903-19919 5:02:01 GGATGGCTGGGGGTGGG
20016 20043 19431 21113 CDS product envelope glycoprotein C 20016-20043 5:02:01 CCGATGTTACCAAACCTTGAAGTCCACC
20680 21015 19431 21113 CDS product envelope glycoprotein C 20680-21015 56:53:14 GGGTGTTGGCTGCGGGATCGGGCTTTCGGGAAGCGGCCGAGGTGGGCGCGACGGCGGGATCGGGCTTTCGGGTAGCGGCCGAGGTGGGCGCGACGGCGGGATCGGGCTTTCGGGAAGCGGCCGAGGTGGGCGCGACGGCGGGATCGGGCTTTCGGGTAGCGGCCGAGGTGGGCGCGACGGCGGGATCGGGCTTTCGGGAAGCGGCCGAGGTGGGCGCGACGGCGGGATCGGGCTTTCGGGAAGCGGCCGAGGTGGGCGCGACGGCGGGATCGGGCTTTCGGGAAGCGGCCGAGGTGGGCGCGACGGCGGGATCGGGCTTTCGGGTAGCGGCCGAGG
21954 21971 21258 22478 CDS product envelope protein UL43 21954-21971 4:01:01 CCTCCCAACCCAGTTCCC
22667 22682 22568 23794 CDS product DNA polymerase processivity subunit 22667-22682 4:01:01 CCGATTCCCTCCTGCC
23497 23512 22568 23794 CDS product DNA polymerase processivity subunit 23497-23512 4:01:01 CCGTCCCTCCGTTGCC
25242 25265 24149 25516 CDS product tegument host shutoff protein 25242-25265 5:02:01 CCTTCTGCCACCCTCATCCTCCCC
26063 26083 25573 26493 CDS product ribonucleotide reductase subunit 2 26063-26083 4:01:01 CCCGTACCTTTTCTTCCAACC
27390 27407 26518 28845 CDS product ribonucleotide reductase subunit 1 27390-27407 4:01:01 CCCAGCCGCCAGGACCCC
28617 28647 26518 28845 CDS product ribonucleotide reductase subunit 1 28617-28647 5:02:01 GGAAACGGATCGTAGGTTAATACGGCAATGG
28744 28763 26518 28845 CDS product ribonucleotide reductase subunit 1 28744-28763 4:01:01 GGTGTGGTTGATTCGGGCGG
29054 29078 29024 30475 CDS product capsid triplex subunit 1 29054-29078 5:02:01 CCCCCCCACACAACCCCCTCTAACC

29099 29113 29024 30475 CDS product capsid triplex subunit 1 29099-29113 4:01:01 CCTCCGAACCGTTCC
29631 29651 29024 30475 CDS product capsid triplex subunit 1 29631-29651 4:01:01 GGGGACCTTTGGCCACGGCGG
29757 29782 29024 30475 CDS product capsid triplex subunit 1 29757-29782 4:01:01 CCGAAAACCTTATGAGCCGCGTTTCC
29905 29935 29024 30475 CDS product capsid triplex subunit 1 29905-29935 6:03:01 CCCCCCACCTTCTCCAACGGTCCAAGGAGCC
31826 31847 30759 33875 CDS product tegument protein UL37 31826-31847 4:01:01 GGCATTTGGGAAAAGGTTCAGG
32732 32754 30759 33875 CDS product tegument protein UL37 32732-32754 4:01:01 CCTTTTACCCGTACACACCCCCC
32826 32874 30759 33875 CDS product tegument protein UL37 32826-32874 8:05:02 CCGTTGACCTGCTTCCCCAGCACGTCCGTCCTTTGGTTCCTGACCGTCC
34145 34167 34083 42374 CDS product large tegument protein 34145-34167 4:01:01 GGTGCCCTGGGACCGGCGTCAGG
34592 34613 34083 42374 CDS product large tegument protein 34592-34613 4:01:01 GGCCATGGGACTATAGGACAGG
35602 35622 34083 42374 CDS product large tegument protein 35602-35622 4:01:01 GGGATTTGTGGTGGAAAATGG
36170 36192 34083 42374 CDS product large tegument protein 36170-36192 4:01:01 CCAGAAGTTCCCCAGGAACCCCC
37006 37031 34083 42374 CDS product large tegument protein 37006-37031 4:01:01 GGTAACCGGTTTTGTGGGACTTAGGG
38044 38062 34083 42374 CDS product large tegument protein 38044-38062 4:01:01 CCACAATCCATATACCCCC
38882 38911 34083 42374 CDS product large tegument protein 38882-38911 5:02:01 GGTACGTTGGACATTCAGGCGGTTGACTGG
39106 39128 34083 42374 CDS product large tegument protein 39106-39128 5:02:01 GGGGGATATGTTGGCATCGGGGG
39160 39185 34083 42374 CDS product large tegument protein 39160-39185 4:01:01 GGCACTCCAGGCATCGGCGTCTGTGG
39610 39626 34083 42374 CDS product large tegument protein 39610-39626 4:01:01 GGGGGAGGCGTCTGTGG
40488 40498 34083 42374 CDS product large tegument protein 40488-40498 4:01:01 CCCCCCCCCCC
41218 41239 34083 42374 CDS product large tegument protein 41218-41239 4:01:01 GGATTATGTGGTCTTTTGGGGG

41342 41360 34083 42374 CDS product large tegument protein 41342-41360 4:01:01 CCCCAAGGCCCCCCAAACC

41444 41526 34083 42374 CDS product large tegument protein 41444-41526 12:09:03
CCCGTCCGGCCCGCGCAGCCCGCGCAGCCCGCGCAGCCCGCGCAGCCCGCGCAGACCGTCCAGCCCGCGCAGCCCATAGAACC

41940 41960 34083 42374 CDS product large tegument protein 41940-41960 4:01:01 CCGATGCCCCCCAAACGGACC

42501 42527 42431 43138 CDS product small capsid protein 42501-42527 6:03:01 GGGGGGTATTTTGGGTTGGGGGCGGGG

42546 42577 42431 43138 CDS product small capsid protein 42546-42577 6:03:01
GGGCCGCGGGCGGTGATCCGGTTGTTGGCTGG

42671 42689 42431 43138 CDS product small capsid protein 42671-42689 4:01:01 GGTGGGGGGTGTTGCGGGG

42793 42813 42431 43138 CDS product small capsid protein 42793-42813 5:02:01 GGATGGATTTGGGGGTTGTGG

42943 42965 42431 43138 CDS product small capsid protein 42943-42965 4:01:01 GGCACTGGCCGCGGCTCCCACGG

42999 43025 42431 43138 CDS product small capsid protein 42999-43025 4:01:01 GGTGACCAGGTTGCAAAGGTCCACTGG

43579 43592 43212 44021 CDS product nuclear egress membrane protein 43579-43592 4:01:01 GGAGAGGTCGGCGG

43876 43894 43212 44021 CDS product nuclear egress membrane protein 43876-43894 4:01:01 GGGCGGGGGGGCGTTGTGG

44729 44751 44506 46263 CDS product DNA packaging protein UL32 44729-44751 5:02:01 GGCCTCTTCGGCGGTAGGTGTGG

45263 45280 44506 46263 CDS product DNA packaging protein UL32 45263-45280 4:01:01 GGACGGACTGACTGGAGG

46152 46175 44506 46263 CDS product DNA packaging protein UL32 46152-46175 5:02:01 CCACGCAAACCAACCGCCAATGCC

46310 46335 46190 47128 CDS product nuclear egress lamina protein 46310-46335 5:02:01
CCAGAACGCCATGCCGCCGTCAAACC

47612 47623 47052 50636 CDS product DNA polymerase catalytic subunit 47612-47623 4:01:01 GGCGGATGGTGG

47709 47725 47052 50636 CDS product DNA polymerase catalytic subunit 47709-47725 4:01:01
CCAAACCCGGCCATCCC

48482 48499 47052 50636 CDS product DNA polymerase catalytic subunit 48482-48499 4:01:01
GGTGGCATAATCGGATGG

51041 51055 50857 54471 CDS product single-stranded DNA-binding protein 51041-51055 4:01:01 GGCGGTGGTTTCTGG
51971 51993 50857 54471 CDS product single-stranded DNA-binding protein 51971-51993 4:01:01 GGCCGGGGTCATTGGTGCGATGG
52028 52043 50857 54471 CDS product single-stranded DNA-binding protein 52028-52043 4:01:01 GGTGGAGGATAGCGGG
52058 52072 50857 54471 CDS product single-stranded DNA-binding protein 52058-52072 4:01:01 GGATGGGGGACCGGG
53061 53080 50857 54471 CDS product single-stranded DNA-binding protein 53061-53080 4:01:01 CCTGTATCCGCCCCAAATCC
53213 53236 50857 54471 CDS product single-stranded DNA-binding protein 53213-53236 4:01:01 GGCAGCCCGGGCAAGGCTTGTAGG
53611 53636 50857 54471 CDS product single-stranded DNA-binding protein 53611-53636 4:01:01 CCAGGCGCCCGCGTGACCCATCATCC
53855 53884 50857 54471 CDS product single-stranded DNA-binding protein 53855-53884 6:03:01 GGCAGGGAATTGGAGCGGTTTAAACGGGGG
53998 54012 50857 54471 CDS product single-stranded DNA-binding protein 53998-54012 4:01:01 CCACCCTATCCGACC
56456 56470 54651 56963 CDS product DNA packaging terminase subunit 2 56456-56470 4:01:01 CCCCCTAACACCGCC
56925 56951 54651 56963 CDS product DNA packaging terminase subunit 2 56925-56951 5:02:01 GGCTGGCTCCGGATTCAACGGACCCGG
57885 57900 56819 59614 CDS product envelope glycoprotein B 57885-57900 4:01:01 GGTTGGTTGGAACTGG
58149 58164 56819 59614 CDS product envelope glycoprotein B 58149-58164 4:01:01 CCAGACCTACCTTGCC
58678 58706 56819 59614 CDS product envelope glycoprotein B 58678-58706 6:03:01 GGGTCCGGGACGGTGGAGGGCCAGCTTGG
59073 59126 56819 59614 CDS product envelope glycoprotein B 59073-59126 8:05:02 GGGACTTGGGACCGCGGGCCAGGCCGTTGGACATGTGGTTCTTGGGGCCACGGG

59179 59224 56819 59614 CDS product envelope glycoprotein B 59179-59224 8:05:02 GGGGCATTGGCCGTGGGATTATTGGTTTTGGCGGGACTGGTAGCGG

59984 60008 59766 60197 CDS product protein V32 59984-60008 4:01:01 CCTTTTCGCCTTAACCGCGACAGCC

60944 60961 60321 61229 CDS product capsid scaffold protein 60944-60961 4:01:01 GGGTGGTGGATATCCCGG

60957 60989 60321 61229 CDS product capsid scaffold protein 60957-60989 5:02:01 CCCGGAAACCCGTACGGTCCCCCGAAGTGTCCC

61017 61039 60321 61229 CDS product capsid scaffold protein 61017-61039 6:03:01 GGTGGGAAGGCAGGCGGGTGTGG

61328 61350 60321 62138 CDS product capsid maturation protease 61328-61350 4:01:01 GGGCCCGCCGGGGTGGAATGTGG

62359 62375 62171 63910 CDS product DNA packaging tegument protein UL25 62359-62375 4:01:01 GGCGGCTATGGGTAGGG

62939 62953 62171 63910 CDS product DNA packaging tegument protein UL25 62939-62953 4:01:01 GGTGCAGGCGGTAGG

63419 63442 62171 63910 CDS product DNA packaging tegument protein UL25 63419-63442 4:01:01 CCCCTTGCCCTTCCCAAGTACACC

63597 63625 62171 63910 CDS product DNA packaging tegument protein UL25 63597-63625 5:02:01 CCGTCTCCAGGGAATCCAAAACCTTGGCC

64856 64876 64807 65832 CDS product thymidine kinase 64856-64876 4:01:01 GGACGGGGCGTATGGAATTGG

65188 65207 64807 65832 CDS product thymidine kinase 65188-65207 4:01:01 CCGACCGACACCCAATCGCC

65289 65309 64807 65832 CDS product thymidine kinase 65289-65309 4:01:01 CCCGCTGAACCCCCCGGGACC

66528 66541 66074 68599 CDS product envelope glycoprotein H 66528-66541 4:01:01 CCCACCAAACCCCC

66958 66980 66074 68599 CDS product envelope glycoprotein H 66958-66980 5:02:01 CCACCGGGTCCGGATCCGGGGCC

67098 67119 66074 68599 CDS product envelope glycoprotein H 67098-67119 4:01:01 GGCCCACACGGAGGCTCTGCGG

68354 68375 66074 68599 CDS product envelope glycoprotein H 68354-68375 4:01:01 CCACAATTCCACCCTTCAATCC

68500 68544 66074 68599 CDS product envelope glycoprotein H 68500-68544 8:05:02

GGAGGGGCGTTTCTGGCGGTAGTGGGGTTTGGTATTATCGGATGG

69030 69047 68668 70293 CDS product tegument protein UL21 69030-69047 4:01:01 GGGAAGCTGGGGGAGTGG

69068 69094 68668 70293 CDS product tegument protein UL21 69068-69094 4:01:01 CCAACGTGCCAATAAAGCCCACTTCCC

69317 69336 68668 70293 CDS product tegument protein UL21 69317-69336 4:01:01 CCCCCCGTTCGCCATATACC

69831 69854 68668 70293 CDS product tegument protein UL21 69831-69854 5:02:01 GGTATTAATGGCGGTGGGTGTTGG

69890 69900 68668 70293 CDS product tegument protein UL21 69890-69900 4:01:01 CCCCCGCCACC

69992 70008 68668 70293 CDS product tegument protein UL21 69992-70008 5:02:01 CCACCGCCCCCGATCCC

71273 71292 70687 71355 CDS product envelope protein UL20 71273-71292 4:01:01 GGACTGTTTGGTGGACACGG

72382 72406 71540 75730 CDS product major capsid protein 72382-72406 5:02:01 GGACGGCAGGTTGACGGTGTGTTGG

72613 72639 71540 75730 CDS product major capsid protein 72613-72639 5:02:01 CCGTCTATACCCCCACAATCCAACTCC

73062 73087 71540 75730 CDS product major capsid protein 73062-73087 4:01:01 GGCCGAGGGTACAGAGGACACATTGG

73098 73120 71540 75730 CDS product major capsid protein 73098-73120 4:01:01 GGGGAGGTTTATGGAAACGTGGG

73257 73296 71540 75730 CDS product major capsid protein 73257-73296 8:05:02

CCTTCCCGGACCGCAGCGTCCCCCGGAAGCCATGCCAACC

73832 73851 71540 75730 CDS product major capsid protein 73832-73851 4:01:01 CCGCCCGAGACCGACTCCCC

73984 74005 71540 75730 CDS product major capsid protein 73984-74005 4:01:01 CCCATTACTCCACACCATGACC

74156 74186 71540 75730 CDS product major capsid protein 74156-74186 4:01:01

CCCCAACTACCCCAGAAGATCCAAGACACCC

74534 74554 71540 75730 CDS product major capsid protein 74534-74554 4:01:01 GGCGGGTTTTGGCAAAAATGG

74557 74570 71540 75730 CDS product major capsid protein 74557-74570 4:01:01 CCACCAATCCCTCC

74604 74620 71540 75730 CDS product major capsid protein 74604-74620 4:01:01 CCAACCCGTTGCCTACC

75070 75087 71540 75730 CDS product major capsid protein 75070-75087 4:01:01 CCCACCGAACCCCTACCC

75161 75181 71540 75730 CDS product major capsid protein 75161-75181 4:01:01 CCATGCCGGTGTCCACTGACC

75477 75493 71540 75730 CDS product major capsid protein 75477-75493 4:01:01 GGAGGCAAAGGCTGTGG
76253 76274 75847 76797 CDS product capsid triplex subunit 2 76253-76274 5:02:01 CCCTTTAACCATCCCCCAGACC
76426 76443 75847 76797 CDS product capsid triplex subunit 2 76426-76443 4:01:01 GGGACGGATCGGATGCGG
77633 77653 76851 82593 CDS product DNA packaging terminase subunit 1 77633-77653 4:01:01 CCGGTCAAACCCCACACCCCC
78920 78938 78170 80200 CDS product DNA packaging tegument protein UL17 78920-78938 4:01:01 CCAGGCACCCCGTCCGACC
79091 79106 78170 80200 CDS product DNA packaging tegument protein UL17 79091-79106 4:01:01 CCGCCAGCATCCCACC
79478 79506 78170 80200 CDS product DNA packaging tegument protein UL17 79478-79506 6:03:01 CCCCCTTACCCGCCTTTAAACCAGGCGCC
79895 79917 78170 80200 CDS product DNA packaging tegument protein UL17 79895-79917 4:01:01 CCAAGGTCCCCGTTCCCGTAGCC
80262 80276 76851 82593 CDS product DNA packaging terminase subunit 1 80262-80276 4:01:01 CCCGTTTCCACCCCC
80477 80494 80360 81451 CDS product tegument protein UL16 80477-80494 4:01:01 CCATCCTGACCGATCCCC
80851 80873 80360 81451 CDS product tegument protein UL16 80851-80873 4:01:01 CCCCCGCGGCTTCCGAAAAATCC
80977 80999 80360 81451 CDS product tegument protein UL16 80977-80999 4:01:01 GGATGGCATGCTCGGATACTTGG
81137 81157 80360 81451 CDS product tegument protein UL16 81137-81157 4:01:01 GGAGAAAGGCCGGTCAGCGGG
83185 83197 82719 83318 CDS product tegument protein UL14 83185-83197 4:01:01 CCCCCCAACCTCC
83573 83598 83168 84700 CDS product tegument serine/threonine protein kinase 83573-83598 4:01:01 GGTTTGCGGGACGGGGAACGTATGGG
84668 84689 83168 84700 CDS product tegument serine/threonine protein kinase 84668-84689 4:01:01 GGCACGATCGGGATTGGATAGG
84701 84713 84667 86322 CDS product deoxyribonuclease 84701-84713 4:01:01 CCCCCAGCCAGCC

| | | | | | |
|---|---|---|---|---|---|
| 84961 | 84985 | 84667 | 86322 | CDS product deoxyribonuclease | 84961-84985 4:01:01 CCACACAGCCTCAGGAGCCGCGTCC |
| 85191 | 85216 | 84667 | 86322 | CDS product deoxyribonuclease | 85191-85216 5:02:01 GGTGATGCGGCGGCAATGGCGTTTGG |
| 85737 | 85755 | 84667 | 86322 | CDS product deoxyribonuclease | 85737-85755 4:01:01 GGATTTAAAGGTGGGAGGG |
| 86246 | 86265 | 86226 | 86471 | CDS product myristylated tegument protein | 86246-86265 4:01:01 GGTCGAGGAGGAATCTGTGG |
| 87065 | 87090 | 86575 | 87882 | CDS product envelope glycoprotein M | 87065-87090 4:01:01 CCACGGACACCGATGACCACAGTCCC |
| 87515 | 87540 | 86575 | 87882 | CDS product envelope glycoprotein M | 87515-87540 5:02:01 CCGCCATGGCCATTGCCGTTAAAGCC |
| 87601 | 87629 | 86575 | 87882 | CDS product envelope glycoprotein M | 87601-87629 6:03:01 CCACCTCTATTTCCGGACCATACCCCCCC |
| 87952 | 87972 | 87881 | 90388 | CDS product DNA replication origin-binding helicase | 87952-87972 4:01:01 GGAGGGGATCTGGTTTCGTGG |
| 88939 | 88957 | 87881 | 90388 | CDS product DNA replication origin-binding helicase | 88939-88957 4:01:01 GGGCCGGATATGGTATCGG |
| 89289 | 89309 | 87881 | 90388 | CDS product DNA replication origin-binding helicase | 89289-89309 4:01:01 GGTTGTATTGGATGGCATGGG |
| 90339 | 90367 | 87881 | 90388 | CDS product DNA replication origin-binding helicase | 90339-90367 5:02:01 GGGATCGTGGCCGCGGATAAACGGGGCGG |
| 91051 | 91067 | 90493 | 92808 | CDS product helicase-primase subunit | 91051-91067 4:01:01 CCTACGCCCGCCTGACC |
| 91348 | 91377 | 90493 | 92808 | CDS product helicase-primase subunit | 91348-91377 5:02:01 GGTACGCGGCGGCGTTTGGAAAACCCGGGG |
| 91532 | 91550 | 90493 | 92808 | CDS product helicase-primase subunit | 91532-91550 4:01:01 GGAAACGGATGGGTTGTGG |
| 91602 | 91615 | 90493 | 92808 | CDS product helicase-primase subunit | 91602-91615 4:01:01 CCCCCCTTACCTCC |
| 92148 | 92169 | 90493 | 92808 | CDS product helicase-primase subunit | 92148-92169 4:01:01 CCCGCCATGGAACCGGTACTCC |
| 92156 | 92179 | 90493 | 92808 | CDS product helicase-primase subunit | 92156-92179 4:01:01 GGAACCGGTACTCCGGGTCGAGGG |

92592 92611 90493 92808 CDS product helicase-primase subunit 92592-92611 4:01:01 CCCAACCCACATGGACCTCC

93017 93043 92855 93850 CDS product tegument protein UL7 93017-93043 6:03:01 GGACAGGGGGGGGTTCCGTGGGGCAGG

93309 93331 92855 93850 CDS product tegument protein UL7 93309-93331 4:01:01 CCCCGGAGGGCCCAATGCCTTCC

93420 93437 92855 93850 CDS product tegument protein UL7 93420-93437 4:01:01 CCCCCACCGTATACACCC

93626 93643 92855 93850 CDS product tegument protein UL7 93626-93643 4:01:01 CCGGTTACCTCCCGCGCC

93776 93807 92855 93850 CDS product tegument protein UL7 93776-93807 6:03:01 CCGGACATCCCATCGGCCATCCCCGCGCCTCC

94732 94751 93675 95984 CDS product capsid portal protein 94732-94751 5:02:01 CCACCGATCCACTTCCTTCC

95020 95040 93675 95984 CDS product capsid portal protein 95020-95040 4:01:01 CCAACACCTCCCCAGAAAGCC

95233 95249 93675 95984 CDS product capsid portal protein 95233-95249 4:01:01 CCGCCGCTCCTCTGGCC

95406 95425 93675 95984 CDS product capsid portal protein 95406-95425 4:01:01 CCGACCCTTCCATTTAAACC

95792 95807 93675 95984 CDS product capsid portal protein 95792-95807 4:01:01 CCAGCCATCTTCCTCC

96570 96590 95996 98641 CDS product helicase-primase helicase subunit 96570-96590 5:02:01 GGGGGACGACGGTCGCGGAGG

97767 97788 95996 98641 CDS product helicase-primase helicase subunit 97767-97788 4:01:01 GGTTAATGGGGCGGGTGCCGGG

97838 97854 95996 98641 CDS product helicase-primase helicase subunit 97838-97854 4:01:01 CCAAAGCCCCCGAGTCC

97865 97883 95996 98641 CDS product helicase-primase helicase subunit 97865-97883 4:01:01 CCTCCATACCCGAAGACCC

98367 98393 95996 98641 CDS product helicase-primase helicase subunit 98367-98393 5:02:01 GGCAATGGCCACCACGGTGGACTATGG

98751 98771 98712 99302 CDS product nuclear protein UL4 98751-98771 4:01:01 GGTTACATGGGGTTAGGCGGG

99159 99182 98712 99302 CDS product nuclear protein UL4 99159-99182 5:02:01 CCACACCAACACCAACCCCGCACC

99206 99241 98712 99302 CDS product nuclear protein UL4 99206-99241 6:03:01 CCTGAAACCAACTGTCCTTCTTCACCACAACCGGCC

99989 100003 99607 100272 CDS product nuclear protein UL3 99989-100003 4:01:01 GGCGGATGGTGTCGG

100243 100256 99607 100272 CDS product nuclear protein UL3 100243-100256 4:01:01 GGTACGGAAGGAGG

100386 100408 100302 101219 CDS product uracil-DNA glycosylase 100386-100408 4:01:01 GGAACACGGGACAATGGCGACGG

100534 100549 100302 101219 CDS product uracil-DNA glycosylase 100534-100549 5:02:01 CCAGCCGCCCCCAGCC

100612 100642 100302 101219 CDS product uracil-DNA glycosylase 100612-100642 6:03:01 CCCCCTGCCTTGCCCAACTCTCCAGGCATCC

101107 101130 100302 101219 CDS product uracil-DNA glycosylase 101107-101130 4:01:01 GGGTTTTTTTGGTAACCGGATGGG

101462 101496 101170 101649 CDS product envelope glycoprotein L 101462-101496 5:02:01 CCGGGGGTGCCGCATAGTCCCACGCCTCATATACC

103174 103193 103082 104485 CDS product ubiquitin E3 ligase ICP0 103174-103193 6:03:01 GGAGGTGGAGGGGGTATTGG

103420 103446 103082 104485 CDS product ubiquitin E3 ligase ICP0 103420-103446 5:02:01 GGTCTCCCCGGTTGAGGTGGTTTCTGG

103492 103526 103082 104485 CDS product ubiquitin E3 ligase ICP0 103492-103526 7:04:01 GGTCCAGGTTTGCATGGTTAGGCGGGTTGGATCGG

103848 103862 103082 104485 CDS product ubiquitin E3 ligase ICP0 103848-103862 4:01:01 CCTGCCCCCCGGTCC

103889 103909 103082 104485 CDS product ubiquitin E3 ligase ICP0 103889-103909 4:01:01 CCATACACCACCGGGGTCGCC

104147 104163 103082 104485 CDS product ubiquitin E3 ligase ICP0 104147-104163 4:01:01 GGTCCTGGGCTTGGTGG

104306 104342 103082 104485 CDS product ubiquitin E3 ligase ICP0 104306-104342 7:04:01 GGACACCGGCAGAGAGGACACTGGACGCTGGTGGAGG

105224 105243 105201 109133 CDS product transcriptional regulator ICP4 105224-105243 5:02:01
CCTCCCCCCGCGCCCTCCCC
105263 105279 105201 109133 CDS product transcriptional regulator ICP4 105263-105279 4:01:01
GGAGGACGAGGACGAGG
105289 105302 105201 109133 CDS product transcriptional regulator ICP4 105289-105302 4:01:01 CCACCTTGACCGCC
105374 105417 105201 109133 CDS product transcriptional regulator ICP4 105374-105417 7:04:01
CCCCGGTGCGTCCCCGTCGTCCCCGCCCCCCTCCTCGCTGTCCC
105431 105455 105201 109133 CDS product transcriptional regulator ICP4 105431-105455 5:02:01
CCCCACCTCCCAATCGTCCAGCTCC
105515 105538 105201 109133 CDS product transcriptional regulator ICP4 105515-105538 5:02:01
CCGTTCCGAGCCCCCGTGGTGTCC
105548 105603 105201 109133 CDS product transcriptional regulator ICP4 105548-105603 9:06:02
CCGTGTTCCGTCGCTCCCCTCCAACACCGTCTCCGCGGCCCCAAAACCGGGCGGCC
105614 105630 105201 109133 CDS product transcriptional regulator ICP4 105614-105630 4:01:01
GGGAATCGGGGGGAGGG
105635 105650 105201 109133 CDS product transcriptional regulator ICP4 105635-105650 4:01:01 CCGAGCCTCGTCCGCC
105664 105680 105201 109133 CDS product transcriptional regulator ICP4 105664-105680 5:02:01
CCACCGACCGACCGGCC
105699 105718 105201 109133 CDS product transcriptional regulator ICP4 105699-105718 4:01:01
GGTTCTGCGGCAGGGTCGGG
105768 105803 105201 109133 CDS product transcriptional regulator ICP4 105768-105803 8:05:02
CCGGCCTCCGGCCCGGCGGCCCCCGGTCTCCGTCCC
105770 105793 105201 109133 CDS product transcriptional regulator ICP4 105770-105793 5:02:01

GGCCTCCGGCCCGGCGGCCCCCGG
105967 106023 105201 109133 CDS product transcriptional regulator ICP4 105967-106023 8:05:02
GGTATTCACGGGGCGACAGGGGGACGCGGGTCTTGGGGCCCGCGCGGGTACACACGG
106179 106223 105201 109133 CDS product transcriptional regulator ICP4 106179-106223 7:04:01
GGCCATCGGACGACGGCCTGGGCGTCCGGTCGCGCCGGGGCCCGG
106195 106221 105201 109133 CDS product transcriptional regulator ICP4 106195-106221 4:01:01
CCTGGGCGTCCGGTCGCGCCGGGGCCC
106254 106269 105201 109133 CDS product transcriptional regulator ICP4 106254-106269 4:01:01 CCATCCCTGGGCCACC
106279 106308 105201 109133 CDS product transcriptional regulator ICP4 106279-106308 6:03:01
CCACCGCGTCCAACACCAGCAACCGGCGCC
106343 106368 105201 109133 CDS product transcriptional regulator ICP4 106343-106368 5:02:01
GGCCCCGGCAAAGGCCAGGTCTCGGG
106411 106437 105201 109133 CDS product transcriptional regulator ICP4 106411-106437 5:02:01
CCGGCGGGCCGGTCCAGTTCCCGGCCC
106452 106484 105201 109133 CDS product transcriptional regulator ICP4 106452-106484 5:02:01
GGCAGGCACAACCGGTTACTCAGGGCTGCCAGG
106628 106647 105201 109133 CDS product transcriptional regulator ICP4 106628-106647 4:01:01
CCGCATCCAGGCGCACCTCC
106692 106707 105201 109133 CDS product transcriptional regulator ICP4 106692-106707 4:01:01 CCTCCGCCCGGACGCC
106752 106778 105201 109133 CDS product transcriptional regulator ICP4 106752-106778 4:01:01
GGGCGCCAGGCCGTGGGAAACAATGGG
106792 106809 105201 109133 CDS product transcriptional regulator ICP4 106792-106809 4:01:01
GGGCGATGGTTTCGGGGG

106819 106844 105201 109133 CDS product transcriptional regulator ICP4 106819-106844 4:01:01
CCTTGCGAGCCTGGTCCGACGGGACC
106831 106867 105201 109133 CDS product transcriptional regulator ICP4 106831-106867 5:02:01
GGTCCGACGGGACCGGGGTATGCAGGGCCCCCCGGGG
106941 106968 105201 109133 CDS product transcriptional regulator ICP4 106941-106968 5:02:01
GGGCCCGAGGTGATGGTCACGGGCTCGG
106972 106994 105201 109133 CDS product transcriptional regulator ICP4 106972-106994 4:01:01
CCCGCCTCTTGGCCTTGGAAACC
107138 107161 105201 109133 CDS product transcriptional regulator ICP4 107138-107161 5:02:01
GGCGGCCGACGGCTGAGGGTCGGG
107171 107202 105201 109133 CDS product transcriptional regulator ICP4 107171-107202 5:02:01
GGCCGGGGTTGTTGGAGAAGGGTGACCGCGGG
107324 107342 105201 109133 CDS product transcriptional regulator ICP4 107324-107342 4:01:01
GGCCGCGGCCGCGTGGGGG
107347 107369 105201 109133 CDS product transcriptional regulator ICP4 107347-107369 5:02:01
CCCAGAGGGCCTCCCCCGTGGCC
107644 107663 105201 109133 CDS product transcriptional regulator ICP4 107644-107663 4:01:01
CCTCATCCTCCCAGTGACCC
107674 107697 105201 109133 CDS product transcriptional regulator ICP4 107674-107697 4:01:01
CCCCGGACGGTCCAAACCGCACCC
107766 107799 105201 109133 CDS product transcriptional regulator ICP4 107766-107799 6:03:01
GGGGGAATTATCGGGGTTCTGGATCGCGGCAGGG
108268 108281 105201 109133 CDS product transcriptional regulator ICP4 108268-108281 4:01:01 CCGCCTTTGCCCCC

108327 108349 105201 109133 CDS product transcriptional regulator ICP4 108327-108349 4:01:01 GGGCCGGGTACAGGTCCACCGGG
108342 108359 105201 109133 CDS product transcriptional regulator ICP4 108342-108359 4:01:01 CCACCGGGTTTCCGCGCC
108621 108666 105201 109133 CDS product transcriptional regulator ICP4 108621-108666 8:05:02 GGAGACGCGGGGGATACAGGGGAGAAGGCCTGCGGGAACGGAGGGG
108834 108854 105201 109133 CDS product transcriptional regulator ICP4 108834-108854 4:01:01 CCCATTAGATCCTGACCGTCC
108955 108982 105201 109133 CDS product transcriptional regulator ICP4 108955-108982 5:02:01 CCCGTCCCACCATGACCCCGTTCTCTCC
109039 109073 105201 109133 CDS product transcriptional regulator ICP4 109039-109073 6:03:01 CCGCCGCGGCCGCGTCCAACAGGTCCATTAACTCC
111244 111272 110581 111417 CDS product regulatory protein ICP22 111244-111272 4:01:01 CCCCGTCCCCAAAGAGACCCCAGCGTGCC
111763 111777 111565 112107 CDS product virion protein US10 111763-111777 4:01:01 GGGGGAGGGCAAAGG
111825 111846 111565 112107 CDS product virion protein US10 111825-111846 5:02:01 CCACCCTCGGTTCCGCCGATCC
112603 112619 112332 112640 CDS product membrane protein US9 112603-112619 4:01:01 CCACGGCCTCACCCTCC
113748 113766 113037 114218 CDS product serine/threonine protein kinase US3 113748-113766 4:01:01 GGTATTATGGCTGGGCTGG
114179 114198 113037 114218 CDS product serine/threonine protein kinase US3 114179-114198 4:01:01 CCCGATCCATATCCAAATCC
115101 115131 114496 115560 CDS product envelope glycoprotein I 115101-115131 5:02:01 CCCGCGACACCCAAAGGGTCCGGTACCTCCC

115830 115853 115808 117679 CDS product envelope glycoprotein E 115830-115853 5:02:01 GGTGGGGGTATTGATGGGGTTCGG
116175 116198 115808 117679 CDS product envelope glycoprotein E 116175-116198 4:01:01 GGAGGATCTTGGGGACGATACGGG
116496 116518 115808 117679 CDS product envelope glycoprotein E 116496-116518 5:02:01 GGTGGTGGATGTGGATTGCGCGG
117334 117356 115808 117679 CDS product envelope glycoprotein E 117334-117356 5:02:01 CCGCCAACGGCCGGTCAGCCACC
117579 117601 115808 117679 CDS product envelope glycoprotein E 117579-117601 4:01:01 GGACTCGGAATCTACGGATACGG
117625 117641 115808 117679 CDS product envelope glycoprotein E 117625-117641 4:01:01 GGAGGGAGTCACGGGGG
118049 118070 117790 118332 CDS product virion protein US10 118049-118070 5:02:01 GGATCGGCGGAACCGAGGGTGG
118118 118132 117790 118332 CDS product virion protein US10 118118-118132 4:01:01 CCTTTGCCCTCCCCC
118623 118651 118480 119316 CDS product regulatory protein ICP22 118623-118651 4:01:01
GGCACGCTGGGGTCTCTTTGGGGACGGGG
120822 120856 120764 124696 CDS product transcriptional regulator ICP4 120822-120856 6:03:01
GGAGTTAATGGACCTGTTGGACGCGGCCGCGGCGG
120913 120940 120764 124696 CDS product transcriptional regulator ICP4 120913-120940 5:02:01
GGAGAGAACGGGGTCATGGTGGGACGGG
121041 121061 120764 124696 CDS product transcriptional regulator ICP4 121041-121061 4:01:01
GGACGGTCAGGATCTAATGGG
121229 121274 120764 124696 CDS product transcriptional regulator ICP4 121229-121274 8:05:02
CCCCTCCGTTCCCGCAGGCCTTCTCCCCTGTATCCCCCGCGTCTCC
121536 121553 120764 124696 CDS product transcriptional regulator ICP4 121536-121553 4:01:01
GGCGCGGAAACCCGGTGG
121546 121568 120764 124696 CDS product transcriptional regulator ICP4 121546-121568 4:01:01
CCCGGTGGACCTGTACCCGGCCC
121614 121627 120764 124696 CDS product transcriptional regulator ICP4 121614-121627 4:01:01 GGGGGCAAAGGCGG

122096 122129 120764 124696 CDS product transcriptional regulator ICP4 122096-122129 6:03:01
CCCTGCCGCGATCCAGAACCCCGATAATTCCCCC
122198 122221 120764 124696 CDS product transcriptional regulator ICP4 122198-122221 4:01:01
GGGTGCGGTTTGGACCGTCCGGGG
122232 122251 120764 124696 CDS product transcriptional regulator ICP4 122232-122251 4:01:01
GGGTCACTGGGAGGATGAGG
122526 122548 120764 124696 CDS product transcriptional regulator ICP4 122526-122548 5:02:01
GGCCACGGGGGAGGCCCTCTGGG
122553 122571 120764 124696 CDS product transcriptional regulator ICP4 122553-122571 4:01:01
CCCCCACGCGGCCGCGGCC
122693 122724 120764 124696 CDS product transcriptional regulator ICP4 122693-122724 5:02:01
CCCGCGGTCACCCTTCTCCAACAACCCCGGCC
122734 122757 120764 124696 CDS product transcriptional regulator ICP4 122734-122757 5:02:01
CCCGACCCTCAGCCGTCGGCCGCC
122901 122923 120764 124696 CDS product transcriptional regulator ICP4 122901-122923 4:01:01
GGTTTCCAAGGCCAAGAGGCGGG
122927 122954 120764 124696 CDS product transcriptional regulator ICP4 122927-122954 5:02:01
CCGAGCCCGTGACCATCACCTCGGGCCC
123028 123064 120764 124696 CDS product transcriptional regulator ICP4 123028-123064 5:02:01
CCCCGGGGGGCCCTGCATACCCCGGTCCCGTCGGACC
123051 123076 120764 124696 CDS product transcriptional regulator ICP4 123051-123076 4:01:01
GGTCCCGTCGGACCAGGCTCGCAAGG
123086 123103 120764 124696 CDS product transcriptional regulator ICP4 123086-123103 4:01:01

CCCCCGAAACCATCGCCC
123117 123143 120764 124696 CDS product transcriptional regulator ICP4 123117-123143 4:01:01
CCCATTGTTTCCCACGGCCTGGCGCCC
123188 123203 120764 124696 CDS product transcriptional regulator ICP4 123188-123203 4:01:01 GGCGTCCGGGCGGAGG
123248 123267 120764 124696 CDS product transcriptional regulator ICP4 123248-123267 4:01:01
GGAGGTGCGCCTGGATGCGG
123411 123443 120764 124696 CDS product transcriptional regulator ICP4 123411-123443 5:02:01
CCTGGCAGCCCTGAGTAACCGGTTGTGCCTGCC
123458 123484 120764 124696 CDS product transcriptional regulator ICP4 123458-123484 5:02:01
GGGCCGGGAACTGGACCGGCCCGCCGG
123527 123552 120764 124696 CDS product transcriptional regulator ICP4 123527-123552 5:02:01
CCCGAGACCTGGCCTTTGCCGGGGCC
123587 123616 120764 124696 CDS product transcriptional regulator ICP4 123587-123616 6:03:01
GGCGCCGGTTGCTGGTGTTGGACGCGGTGG
123626 123641 120764 124696 CDS product transcriptional regulator ICP4 123626-123641 4:01:01 GGTGGCCCAGGGATGG
123672 123716 120764 124696 CDS product transcriptional regulator ICP4 123672-123716 7:04:01
CCGGGCCCCGGCGCGACCGGACGCCCAGGCCGTCGTCCGATGGCC
123674 123700 120764 124696 CDS product transcriptional regulator ICP4 123674-123700 4:01:01
GGGCCCCGGCGCGACCGGACGCCCAGG
123872 123928 120764 124696 CDS product transcriptional regulator ICP4 123872-123928 8:05:02
CCGTGTGTACCCGCGCGGGCCCCAAGACCCGCGTCCCCCTGTCGCCCCGTGAATACC
124092 124127 120764 124696 CDS product transcriptional regulator ICP4 124092-124127 8:05:02
GGGACGGAGACCGGGGGCCGCCGGGCCGGAGGCCGG

124102 124125 120764 124696 CDS product transcriptional regulator ICP4 124102-124125 5:02:01
CCGGGGGCCGCCGGGCCGGAGGCC
124177 124196 120764 124696 CDS product transcriptional regulator ICP4 124177-124196 4:01:01
CCCGACCCTGCCGCAGAACC
124215 124231 120764 124696 CDS product transcriptional regulator ICP4 124215-124231 5:02:01
GGCCGGTCGGTCGGTGG
124245 124260 120764 124696 CDS product transcriptional regulator ICP4 124245-124260 4:01:01 GGCGGACGAGGCTCGG
124265 124281 120764 124696 CDS product transcriptional regulator ICP4 124265-124281 4:01:01
CCCTCCCCCCGATTCCC
124292 124347 120764 124696 CDS product transcriptional regulator ICP4 124292-124347 9:06:02
GGCCGCCCGGTTTTGGGGCCGCGGAGACGGTGTTGGAGGGGAGCGACGGAACACGG
124357 124380 120764 124696 CDS product transcriptional regulator ICP4 124357-124380 5:02:01
GGACACCACGGGGGCTCGGAACGG
124440 124464 120764 124696 CDS product transcriptional regulator ICP4 124440-124464 5:02:01
GGAGCTGGACGATTGGGAGGTGGGG
124478 124521 120764 124696 CDS product transcriptional regulator ICP4 124478-124521 7:04:01
GGGACAGCGAGGAGGGGGGCGGGGACGACGGGGACGCACCGGGG
124593 124606 120764 124696 CDS product transcriptional regulator ICP4 124593-124606 4:01:01 GGCGGTCAAGGTGG
124616 124632 120764 124696 CDS product transcriptional regulator ICP4 124616-124632 4:01:01
CCTCGTCCTCGTCCTCC
124652 124671 120764 124696 CDS product transcriptional regulator ICP4 124652-124671 5:02:01
GGGGAGGGCGCGGGGGGAGG

gi|50313241|ref|NC_001491.2| Equid herpesvirus 1, complete genome

1323 1365 1298 1906 CDS product membrane protein UL56 1323-1365 9:06:02 CCGCGCCTCCTCTGTCTCCATCTCCATGTGCCCACCGCCGCCC

1506 1533 1298 1906 CDS product membrane protein UL56 1506-1533 5:02:01 CCTGCCTTCTACCGGCTCCCTGAGATCC

1605 1639 1298 1906 CDS product membrane protein UL56 1605-1639 6:03:01 GGAACGCGGGGCAGAGCGGCCGCGGGCGTGGACGG

1676 1695 1298 1906 CDS product membrane protein UL56 1676-1695 4:01:01 CCCTGTGCCAGGCGCCACCC

1766 1782 1298 1906 CDS product membrane protein UL56 1766-1782 4:01:01 CCGATCCCCACCAGACC

1955 1969 1945 2562 CDS product membrane protein V1 1955-1969 4:01:01 CCTTTCCAAACCCCC

2348 2364 1945 2562 CDS product membrane protein V1 2348-2364 4:01:01 CCGGACCCTGCCAGGCC

2397 2415 1945 2562 CDS product membrane protein V1 2397-2415 4:01:01 GGTGGAGGGGCGCGTCTGG

2977 2995 2841 3614 CDS product myristylated tegument protein CIRC 2977-2995 4:01:01 GGACGTCGGGCGGCCCGGG

3118 3144 2841 3614 CDS product myristylated tegument protein CIRC 3118-3144 5:02:01 GGTCTTTGGATACGAGGACGGTACCGG

3300 3316 2841 3614 CDS product myristylated tegument protein CIRC 3300-3316 4:01:01 CCGAGGAGGCCACCACC

3337 3372 2841 3614 CDS product myristylated tegument protein CIRC 3337-3372 6:03:01 CCTACTGGGCCAGACCCAGAGCTCCACGCACCGTCC

3413 3442 2841 3614 CDS product myristylated tegument protein CIRC 3413-3442 5:02:01 CCACCCACGAAACCGAGTAGCCAAAGGCCC

3438 3456 2841 3614 CDS product myristylated tegument protein CIRC 3438-3456 4:01:01 GGCCCAGGGGCAAGGGGGG

| | | | | | | | |
|---|---|---|---|---|---|---|---|
| 3460 | 3473 | 2841 | 3614 | CDS product myristylated tegument protein CIRC | 3460-3473 | 4:01:01 | CCCTCCAGCCCGCC |
| 4057 | 4073 | 3647 | 4249 | CDS product nuclear protein UL55 | 4057-4073 | 5:02:01 | GGCCGGCTGGGAGGTGG |
| 4168 | 4202 | 3647 | 4249 | CDS product nuclear protein UL55 | 4168-4202 | 6:03:01 | CCCGCCGTGGTTCCTCCCGTGGGAGCCTATATCCC |
| 4499 | 4522 | 4462 | 5874 | CDS product multifunctional expression regulator | 4499-4522 | 4:01:01 | GGTAAACCGGAACGAGGGTGTAGG |
| 5096 | 5122 | 4462 | 5874 | CDS product multifunctional expression regulator | 5096-5122 | 5:02:01 | CCGATCCGAAGCCGAGCACCGGAGCCC |
| 5389 | 5405 | 4462 | 5874 | CDS product multifunctional expression regulator | 5389-5405 | 4:01:01 | CCTGCGCCGCCATGTCC |
| 5513 | 5534 | 4462 | 5874 | CDS product multifunctional expression regulator | 5513-5534 | 4:01:01 | CCGGCTCCGGCTTTTCCCAGCC |
| 5589 | 5608 | 4462 | 5874 | CDS product multifunctional expression regulator | 5589-5608 | 4:01:01 | CCCATGGCCAAACGCCGGCC |
| 6171 | 6190 | 6011 | 7042 | CDS product envelope glycoprotein K | 6171-6190 | 5:02:01 | GGGTGGCGGGAGTCGGGGGG |
| 6258 | 6272 | 6011 | 7042 | CDS product envelope glycoprotein K | 6258-6272 | 4:01:01 | CCACCACAAACCCCC |
| 6973 | 7000 | 6011 | 7042 | CDS product envelope glycoprotein K | 6973-7000 | 5:02:01 | GGTAAACGCGGCATAGGCGGTGATAAGG |
| 7372 | 7388 | 7056 | 10301 | CDS product helicase-primase primase subunit | 7372-7388 | 4:01:01 | CCACCTCACCGAGTACC |
| 7566 | 7578 | 7056 | 10301 | CDS product helicase-primase primase subunit | 7566-7578 | 4:01:01 | GGATGGCGGGGGG |
| 7870 | 7891 | 7056 | 10301 | CDS product helicase-primase primase subunit | 7870-7891 | 6:03:01 | GGAGGTAAGGGGGTGGCACAGG |
| 8814 | 8848 | 7056 | 10301 | CDS product helicase-primase primase subunit | 8814-8848 | 6:03:01 | |

GGTGGCGTGGAATGGACACTCTGGGGTTTGTTGGG
8867 8882 7056 10301 CDS product helicase-primase primase subunit 8867-8882 4:01:01 CCAGCCGCGGCCAGCC
9452 9469 7056 10301 CDS product helicase-primase primase subunit 9452-9469 4:01:01
GGGGTTAGGTGCGGCCGG
9508 9531 7056 10301 CDS product helicase-primase primase subunit 9508-9531 4:01:01
GGTCGTAGGTGCCCCCGGATCTGG
9689 9711 7056 10301 CDS product helicase-primase primase subunit 9689-9711 5:02:01
GGAGGTCAAACCGGAGCGGCCGG
9922 9942 7056 10301 CDS product helicase-primase primase subunit 9922-9942 4:01:01
CCAACGCCCGGGCTCCGGTCC
10002 10015 7056 10301 CDS product helicase-primase primase subunit 10002-10015 4:01:01 CCTCCCACCATACC
10194 10213 7056 10301 CDS product helicase-primase primase subunit 10194-10213 4:01:01
CCCTCCGAGCTCTCCTGTCC
10385 10412 10300 11037 CDS product tegument protein UL51 10385-10412 5:02:01 CCCAGCTACCATGCTCCGCCTACAGTCC
10471 10494 10300 11037 CDS product tegument protein UL51 10471-10494 4:01:01 CCTCTGCCGACAACACCCGGCGCC
10576 10598 10300 11037 CDS product tegument protein UL51 10576-10598 4:01:01 CCGACAACCCGAACCTGAACGCC
10642 10665 10300 11037 CDS product tegument protein UL51 10642-10665 4:01:01 CCTGCCTCGCGGCCCTCATGCACC
10780 10809 10300 11037 CDS product tegument protein UL51 10780-10809 5:02:01
CCCTCGGCCTGGATCCCCAGGCAACCGTCC
10920 10932 10300 11037 CDS product tegument protein UL51 10920-10932 4:01:01 CCTCTCCTCCCCC
11141 11164 11135 12115 CDS product deoxyuridine triphosphatase 11141-11164 5:02:01 CCGGTTGAGCCAAACCCTCCATCC
11251 11272 11135 12115 CDS product deoxyuridine triphosphatase 11251-11272 4:01:01 GGGGGAGTACGTGGGAAAGGGG
11477 11495 11135 12115 CDS product deoxyuridine triphosphatase 11477-11495 4:01:01 GGCGTGACGGCGGGGTTGG

12130 12171 12084 12386 CDS product envelope glycoprotein N 12130-12171 8:05:02 GGTGGTGCTTGGTCTGGCCAGAGGGGCTGGTGGCGACCCAGG

12321 12340 12084 12386 CDS product envelope glycoprotein N 12321-12340 4:01:01 CCTGCCAGGCATACCGCGCC

12885 12906 12549 13463 CDS product tegument protein VP22 12885-12906 5:02:01 CCTCCAATGGCCGCCAAAGCCC

12947 12975 12549 13463 CDS product tegument protein VP22 12947-12975 6:03:01 CCACCCCCACGTGTTCCAACGCGACCACC

13343 13368 12549 13463 CDS product tegument protein VP22 13343-13368 4:01:01 GGGTATGCGGCAGCTGGACCCAACGG

13715 13739 13595 14944 CDS product transactivating tegument protein VP16 13715-13739 4:01:01 CCATCGAGTCCGTTTATTCCATTCC

13762 13785 13595 14944 CDS product transactivating tegument protein VP16 13762-13785 6:03:01 CCACCGCCCAAGGCCGCCAGCCCC

13802 13832 13595 14944 CDS product transactivating tegument protein VP16 13802-13832 5:02:01 GGCTACAAGGCGAGCTGGGTTTTCCGGAGGG

13959 13995 13595 14944 CDS product transactivating tegument protein VP16 13959-13995 6:03:01 CCTCGATAGCCTGCCCACTCCCAGCCACTATAGCCCC

14026 14047 13595 14944 CDS product transactivating tegument protein VP16 14026-14047 4:01:01 CCCTTCCCAGAGGTTCCCGCCC

14330 14350 13595 14944 CDS product transactivating tegument protein VP16 14330-14350 4:01:01 CCTGGCGCCTTCACGCCAGCC

14763 14775 13595 14944 CDS product transactivating tegument protein VP16 14763-14775 4:01:01 CCCGCCCTCCCCC

14788 14814 13595 14944 CDS product transactivating tegument protein VP16 14788-14814 5:02:01 CCAGGCGACCCAGTCCCGCCTCTTACC

15594 15620 15317 17932 CDS product tegument protein VP13/14 15594-15620 4:01:01
CCCCGCACAGCCACAAGCACCCAGACC
15666 15683 15317 17932 CDS product tegument protein VP13/14 15666-15683 4:01:01 GGAAGAGGAGGAAGAGGG
15802 15819 15317 17932 CDS product tegument protein VP13/14 15802-15819 4:01:01 CCCACGTCCGCCGAATCC
15838 15860 15317 17932 CDS product tegument protein VP13/14 15838-15860 5:02:01 GGTGAAATGGTGGCCGGTGCGGG
16182 16198 15317 17932 CDS product tegument protein VP13/14 16182-16198 4:01:01 GGGGGACTACATGGAGG
16289 16304 15317 17932 CDS product tegument protein VP13/14 16289-16304 4:01:01 CCAACCACACCAACCC
16356 16373 15317 17932 CDS product tegument protein VP13/14 16356-16373 4:01:01 CCTGGCCCGCACACCCCC
16775 16798 15317 17932 CDS product tegument protein VP13/14 16775-16798 4:01:01 GGGAGAGGCGCAGGGAAACTGGGG
17097 17110 15317 17932 CDS product tegument protein VP13/14 17097-17110 4:01:01 GGTGGCAATGGAGG
17440 17459 15317 17932 CDS product tegument protein VP13/14 17440-17459 4:01:01 GGGGTGTTTTTGGGGGTGGG
17460 17488 15317 17932 CDS product tegument protein VP13/14 17460-17488 5:02:01
CCTAATCCTTCAGCGCCTGGCGGGCCACC
17674 17690 15317 17932 CDS product tegument protein VP13/14 17674-17690 4:01:01 CCTCGCCCCGGCCCTCC
17776 17806 15317 17932 CDS product tegument protein VP13/14 17776-17806 6:03:01
GGCGCCGGCCTGGGACGCATGGTGGACATGG
17852 17862 15317 17932 CDS product tegument protein VP13/14 17852-17862 4:01:01 CCACCTCCTCC
18084 18096 18083 20326 CDS product tegument protein VP11/12 18084-18096 4:01:01 GGACGGAGGGGGG
18156 18171 18083 20326 CDS product tegument protein VP11/12 18156-18171 4:01:01 CCTGCTGCCAACCCCC
18478 18493 18083 20326 CDS product tegument protein VP11/12 18478-18493 4:01:01 GGTGCGGTGGGTCAGG
18920 18933 18083 20326 CDS product tegument protein VP11/12 18920-18933 4:01:01 CCACCGCCGACGCC
19097 19114 18083 20326 CDS product tegument protein VP11/12 19097-19114 4:01:01 GGCTCGATGGTGGCGTGG
19399 19428 18083 20326 CDS product tegument protein VP11/12 19399-19428 6:03:01

CCTCCCCAAGGGCCGCTACCGGCACCCCCC

| | | | | | |
|---|---|---|---|---|---|
| 19495 | 19508 | 18083 | 20326 | CDS product tegument protein VP11/12 | 19495-19508 4:01:01 CCAACCTCCCCTCC |
| 19523 | 19538 | 18083 | 20326 | CDS product tegument protein VP11/12 | 19523-19538 4:01:01 CCCCCAGGACCAACCC |
| 19722 | 19743 | 18083 | 20326 | CDS product tegument protein VP11/12 | 19722-19743 4:01:01 CCCCTCAACGCCATCGCCAACC |
| 19760 | 19779 | 18083 | 20326 | CDS product tegument protein VP11/12 | 19760-19779 4:01:01 CCAATCCACTCCTTGGGCCC |
| 19800 | 19817 | 18083 | 20326 | CDS product tegument protein VP11/12 | 19800-19817 4:01:01 CCCCCAACCCGTTGATCC |
| 19875 | 19890 | 18083 | 20326 | CDS product tegument protein VP11/12 | 19875-19890 4:01:01 GGTCGAGGAGGTTTGG |
| 19926 | 19948 | 18083 | 20326 | CDS product tegument protein VP11/12 | 19926-19948 4:01:01 CCCCAGCCGTGCCGGGCGTTCCC |
| 19978 | 19992 | 18083 | 20326 | CDS product tegument protein VP11/12 | 19978-19992 4:01:01 CCATCGTCCCCCGCC |
| 20002 | 20018 | 18083 | 20326 | CDS product tegument protein VP11/12 | 20002-20018 4:01:01 CCACCCGCCAATTCTCC |
| 20178 | 20195 | 18083 | 20326 | CDS product tegument protein VP11/12 | 20178-20195 4:01:01 CCCCGGCCCCACCCAACC |
| 21518 | 21539 | 21445 | 22851 | CDS product envelope glycoprotein C | 21518-21539 4:01:01 CCAGCGCCAGTGCCGCGGCCCC |
| 21760 | 21777 | 21445 | 22851 | CDS product envelope glycoprotein C | 21760-21777 4:01:01 GGAACCCCTGGTAGGTGG |
| 21971 | 21987 | 21445 | 22851 | CDS product envelope glycoprotein C | 21971-21987 4:01:01 CCTCCTCCGGATCAACC |
| 22163 | 22182 | 21445 | 22851 | CDS product envelope glycoprotein C | 22163-22182 4:01:01 GGCTCGGGTGAACGGAAAGG |
| 22273 | 22285 | 21445 | 22851 | CDS product envelope glycoprotein C | 22273-22285 4:01:01 CCGGCCGTCCGCC |
| 22620 | 22645 | 21445 | 22851 | CDS product envelope glycoprotein C | 22620-22645 4:01:01 GGTGCAGGTGATTGTGGTTTCATGGG |
| 23153 | 23168 | 23029 | 24234 | CDS product envelope protein UL43 | 23153-23168 4:01:01 CCCCCAGTATACCCCC |
| 23243 | 23263 | 23029 | 24234 | CDS product envelope protein UL43 | 23243-23263 4:01:01 GGGCCGTGGAGTGGATGCCGG |
| 24114 | 24134 | 23029 | 24234 | CDS product envelope protein UL43 | 24114-24134 4:01:01 GGAGGCCGCCCAGGTACATGG |
| 24678 | 24694 | 24479 | 25696 | CDS product DNA polymerase processivity subunit | 24678-24694 4:01:01 GGCACGGCTGGGTCGGG |
| 25396 | 25406 | 24479 | 25696 | CDS product DNA polymerase processivity subunit | 25396-25406 4:01:01 GGCGGGGGGGG |

26778 26795 26262 27755 CDS product tegument host shutoff protein 26778-26795 4:01:01 CCCCGCGCCTTTCCTACC
26966 26987 26262 27755 CDS product tegument host shutoff protein 26966-26987 5:02:01 CCGCTGTTTCCGCCAACCCTCC
27357 27384 26262 27755 CDS product tegument host shutoff protein 27357-27384 6:03:01 GGCCTCGGCGGTCTGCGGAGGCCTCAGG
27464 27479 26262 27755 CDS product tegument host shutoff protein 27464-27479 4:01:01 CCCCCCAAAACCCGCC
27565 27585 26262 27755 CDS product tegument host shutoff protein 27565-27585 4:01:01 CCTACCCATCACCCAGGAGCC
27974 28005 27894 28859 CDS product ribonucleotide reductase subunit 2 27974-28005 6:03:01 GGCGAGAGGAAAGCTCGGGGATGGTTTGGGGG
28291 28311 27894 28859 CDS product ribonucleotide reductase subunit 2 28291-28311 4:01:01 GGTTGTGGGTACGGAGATAGG
28399 28429 27894 28859 CDS product ribonucleotide reductase subunit 2 28399-28429 6:03:01 CCTTTGCCTGTAGCCAGGCAACCTTCCGTCC
28702 28725 27894 28859 CDS product ribonucleotide reductase subunit 2 28702-28725 5:02:01 GGTCATCGGAAATGGGAAGGTCGG
30538 30559 28904 31276 CDS product ribonucleotide reductase subunit 1 30538-30559 4:01:01 CCCGGCCTCTTCCATTAGAGCC
30669 30685 28904 31276 CDS product ribonucleotide reductase subunit 1 30669-30685 4:01:01 CCTGTCCGGCCAGCGCC
30712 30733 28904 31276 CDS product ribonucleotide reductase subunit 1 30712-30733 4:01:01 CCAGCCTACTTCCCCGCTGACC
30744 30760 28904 31276 CDS product ribonucleotide reductase subunit 1 30744-30760 4:01:01

CCATAGCCGGTTCCTCC

31151 31169 28904 31276 CDS product ribonucleotide reductase subunit 1 31151-31169 4:01:01 GGGCGGGGAGGCAGCGTGG

31535 31555 31519 32916 CDS product capsid triplex subunit 1 31535-31555 4:01:01 CCCAATCCTGTGGGTCCCACC

31610 31650 31519 32916 CDS product capsid triplex subunit 1 31610-31650 7:04:01 GGCGTGTGGTTCTGGGGGAGTCTGAGGCTATGGCCCCCAGG

31796 31829 31519 32916 CDS product capsid triplex subunit 1 31796-31829 7:04:01 GGAGCGGGAGGGCGGCGTTTGGGTGCGGCTCTGG

31835 31850 31519 32916 CDS product capsid triplex subunit 1 31835-31850 4:01:01 CCGCTCCCTCCGTTCC

31931 31966 31519 32916 CDS product capsid triplex subunit 1 31931-31966 6:03:01 CCGCCACGTACACCCTCACTCCCTCCCGGCGTAACC

32432 32451 31519 32916 CDS product capsid triplex subunit 1 32432-32451 4:01:01 CCCAAGCCTCGTCCAACTCC

32496 32537 31519 32916 CDS product capsid triplex subunit 1 32496-32537 9:06:02 GGCATCGAGGTGGTGGCGGAGGCGGATTATCGGAAGTCCGGG

32709 32721 31519 32916 CDS product capsid triplex subunit 1 32709-32721 4:01:01 GGGGGTGGCCTGG

33583 33609 33292 36354 CDS product tegument protein UL37 33583-33609 7:04:01 GGACGGACAGGGAAATGGAGGCGGTGG

33933 33957 33292 36354 CDS product tegument protein UL37 33933-33957 4:01:01 GGCCTAATGGTCAAGGGCTACAAGG

34012 34037 33292 36354 CDS product tegument protein UL37 34012-34037 4:01:01 CCTATTTTCCCAAAGGAACCGCGACC

34366 34403 33292 36354 CDS product tegument protein UL37 34366-34403 6:03:01 CCGAGCACGCCAGTAACACCTGGGAGACCATCCAGGCC

34467 34491 33292 36354 CDS product tegument protein UL37 34467-34491 5:02:01 GGAATTTTGGAGCGCGTGGTGGTGG

34706 34728 33292 36354 CDS product tegument protein UL37 34706-34728 5:02:01 GGGTCTGGCGGACAAGGGAGTGG

34831 34856 33292 36354 CDS product tegument protein UL37 34831-34856 4:01:01 CCACTGGGCCCGGAACCCATAGCGCC

35170 35191 33292 36354 CDS product tegument protein UL37 35170-35191 4:01:01 GGGCCAGGGACTACGGCGCCGG
35272 35304 33292 36354 CDS product tegument protein UL37 35272-35304 6:03:01
CCCCTACCCCAACCATGGCCCATGCCAACAACC
35379 35408 33292 36354 CDS product tegument protein UL37 35379-35408 5:02:01
CCAGTGCCCGAACGACCCTCCGTTAAAACC
35846 35881 33292 36354 CDS product tegument protein UL37 35846-35881 7:04:01
GGCCAGGGCCCAGAGGGGGTTGGCAATAAGGGCCGG
35892 35911 33292 36354 CDS product tegument protein UL37 35892-35911 4:01:01 GGGGGTTCTGAGGCTCCCGG
36607 36641 36588 46853 CDS product large tegument protein 36607-36641 6:03:01
GGCGAATAAGGCGGGGGGCGCTCAGGCCGATGTGG
36797 36819 36588 46853 CDS product large tegument protein 36797-36819 5:02:01 GGGCGGGCCTGGACTGTGGCCGG
37049 37067 36588 46853 CDS product large tegument protein 37049-37067 4:01:01 GGGGCTATTGGTCTGGCGG
37393 37420 36588 46853 CDS product large tegument protein 37393-37420 6:03:01 GGCTGTGGGTGCGGTTTCGGAGGCGTGG
37462 37478 36588 46853 CDS product large tegument protein 37462-37478 4:01:01 GGACATGGAGGGTGTGG
37559 37579 36588 46853 CDS product large tegument protein 37559-37579 5:02:01 CCCCCGTGGACGCCTCCCACC
37857 37871 36588 46853 CDS product large tegument protein 37857-37871 4:01:01 GGCTCTCGGGGGAGG
38077 38095 36588 46853 CDS product large tegument protein 38077-38095 4:01:01 GGTGGAAAATGGCGCGCGG
38449 38472 36588 46853 CDS product large tegument protein 38449-38472 4:01:01 CCTATTTGCCCGAGAACCGAAACC
38689 38716 36588 46853 CDS product large tegument protein 38689-38716 5:02:01 CCTATCAGCCGTCCCTACCCGCGTAGCC
38917 38937 36588 46853 CDS product large tegument protein 38917-38937 4:01:01 GGCCTCGGCGGGTATCCAGGG
38932 38946 36588 46853 CDS product large tegument protein 38932-38946 4:01:01 CCAGGGACCTCCGCC
39641 39665 36588 46853 CDS product large tegument protein 39641-39665 6:03:01 CCAAACTCCACCACCTCATCCGCCC
40042 40055 36588 46853 CDS product large tegument protein 40042-40055 4:01:01 GGGGGTTCAGGAGG

| | | | | | | | |
|---|---|---|---|---|---|---|---|
| 40068 | 40084 | 36588 | 46853 | CDS product large tegument protein | 40068-40084 | 4:01:01 | CCGCGATAGCCGCCGCC |
| 40110 | 40126 | 36588 | 46853 | CDS product large tegument protein | 40110-40126 | 5:02:01 | CCGCCGCCACTGCCGCC |
| 40131 | 40148 | 36588 | 46853 | CDS product large tegument protein | 40131-40148 | 4:01:01 | GGGAGCTGGCGAGGCTGG |
| 40438 | 40454 | 36588 | 46853 | CDS product large tegument protein | 40438-40454 | 4:01:01 | GGAACAGGCCCTGGCGG |
| 41344 | 41365 | 36588 | 46853 | CDS product large tegument protein | 41344-41365 | 4:01:01 | GGATATTAAGGCCGTGGAGTGG |
| 41526 | 41542 | 36588 | 46853 | CDS product large tegument protein | 41526-41542 | 4:01:01 | GGGACGAGGCATGGGGG |
| 41983 | 42001 | 36588 | 46853 | CDS product large tegument protein | 41983-42001 | 4:01:01 | CCGCCCTCACCTGGAAGCC |
| 42321 | 42338 | 36588 | 46853 | CDS product large tegument protein | 42321-42338 | 4:01:01 | GGTTTGGGCTGGAAAAGG |
| 42908 | 42929 | 36588 | 46853 | CDS product large tegument protein | 42908-42929 | 5:02:01 | GGCGAAAGCGGACGGGCGGCGG |
| 42942 | 42979 | 36588 | 46853 | CDS product large tegument protein | 42942-42979 | 9:06:02 | CCTCCGCCATTCCCGGAAACCCCCTGGCCACCTTTACC |
| 43368 | 43388 | 36588 | 46853 | CDS product large tegument protein | 43368-43388 | 4:01:01 | CCAGACCCGGGGCGACCGACC |
| 43688 | 43710 | 36588 | 46853 | CDS product large tegument protein | 43688-43710 | 4:01:01 | CCTCCAATGAACCCGGATGACCC |
| 43966 | 44000 | 36588 | 46853 | CDS product large tegument protein | 43966-44000 | 7:04:01 | GGCGTGGCAAGAGTGGTTGGAGGATGGGTTCGCGG |
| 44022 | 44044 | 36588 | 46853 | CDS product large tegument protein | 44022-44044 | 5:02:01 | CCAACGCCATGCCGGCGCCTCCC |
| 44145 | 44167 | 36588 | 46853 | CDS product large tegument protein | 44145-44167 | 4:01:01 | CCGCCATCATGTCCGGAAAGCCC |
| 44199 | 44260 | 36588 | 46853 | CDS product large tegument protein | 44199-44260 | 13:10:03 | CCGTTCCACCTTCCGTACCTGCTCCTCCCACTCTTCCACCCGCTCCCCCTCTGCCCCAATCC |
| 44543 | 44581 | 36588 | 46853 | CDS product large tegument protein | 44543-44581 | 8:05:02 | CCAGTTCCACCCACCGATAAGCCGTCAACCACCACTCCC |
| 44741 | 44760 | 36588 | 46853 | CDS product large tegument protein | 44741-44760 | 5:02:01 | CCCGGAGCCAAACCACCCCC |
| 44934 | 44992 | 36588 | 46853 | CDS product large tegument protein | 44934-44992 | 11:08:02 | |

CCCAATCAACCAAGGAGCCCCCAAAGCCTGCCGTAGAGACCCCCGCCGCGGCCCCGGCC
45138 45164 36588 46853 CDS product large tegument protein 45138-45164 6:03:01 CCCCCGCCGCGGCCCCGGCCAAAGACC
45187 45271 36588 46853 CDS product large tegument protein 45187-45271 15:12:03
CCCAAAGCCGGCCAAGGACCAGGCCAAGGACCAGGCCAAGGACCAGGCCAAGGACCAGGCCAAGGACCAGGCCAAGGACCAGGCC
45196 45269 36588 46853 CDS product large tegument protein 45196-45269 13:10:03
GGCCAAGGACCAGGCCAAGGACCAGGCCAAGGACCAGGCCAAGGACCAGGCCAAGGACCAGGCCAAGGACCAGG
45565 45595 36588 46853 CDS product large tegument protein 45565-45595 5:02:01
CCCATTGCCCGATTCTCCCACCGACGACCCC
45935 45948 36588 46853 CDS product large tegument protein 45935-45948 4:01:01 CCGACCGCCATTCC
46097 46149 36588 46853 CDS product large tegument protein 46097-46149 11:08:02
CCTCAGCCGCCACCGGCAAACCAGACGCCACCGCCTCAAGAACCCCCAGCACC
47519 47538 47403 48230 CDS product nuclear egress membrane protein 47519-47538 4:01:01 GGAGATGCTGGCGAGGCTGG
47921 47935 47403 48230 CDS product nuclear egress membrane protein 47921-47935 4:01:01 GGGGGCGTTGGGGGG
48098 48115 47403 48230 CDS product nuclear egress membrane protein 48098-48115 4:01:01 GGCTGGTAGGTGTTTGGG
48819 48835 48763 50625 CDS product DNA packaging protein UL32 48819-48835 4:01:01 GGAGGCTGGCAGCCGGG
49341 49369 48763 50625 CDS product DNA packaging protein UL32 49341-49369 4:01:01
CCCCGCTGCGCCGCGTCCGGGACGGCCCC
49359 49404 48763 50625 CDS product DNA packaging protein UL32 49359-49404 10:07:02
GGGACGGCCCCGGCGGCAACGGCCATGGCCGGCCTCGGTATGGCGG
49521 49570 48763 50625 CDS product DNA packaging protein UL32 49521-49570 11:08:02
GGCAGACATGGAGCGGGCAGGCCGGCGGATGGGGGCGGTGGGGTGTTTGG
49888 49911 48763 50625 CDS product DNA packaging protein UL32 49888-49911 4:01:01 GGGCCTGCGTGGAGGAGTATTGGG
49985 50004 48763 50625 CDS product DNA packaging protein UL32 49985-50004 4:01:01 GGACACGGCCACGGGGCCGG

50217 50243 48763 50625 CDS product DNA packaging protein UL32 50217-50243 6:03:01 GGCGACTGCGGCGTGGGTGGCGGCGGG

50379 50401 48763 50625 CDS product DNA packaging protein UL32 50379-50401 5:02:01 CCCATAGGCCCCCCTCCAGAACC

51009 51043 50618 51598 CDS product nuclear egress lamina protein 51009-51043 7:04:01 CCCAACCTGCACCGTTTCCGGCGAGCCTCGCCTCC

51140 51171 50618 51598 CDS product nuclear egress lamina protein 51140-51171 6:03:01 CCGCCCAGGGGGACCAGGCCGGCGTTGCCGCC

51147 51161 50618 51598 CDS product nuclear egress lamina protein 51147-51161 4:01:01 GGGGGACCAGGCCGG

51372 51389 50618 51598 CDS product nuclear egress lamina protein 51372-51389 4:01:01 CCTACTGGCCGCCTGCCC

51800 51831 51522 55184 CDS product DNA polymerase catalytic subunit 51800-51831 6:03:01 GGAAACATAGGTCGGGTCTTCGGCGAGGTCGG

51852 51894 51522 55184 CDS product DNA polymerase catalytic subunit 51852-51894 8:05:02 GGTTGGGGGCGTGCGGTCTTGGTTACCACGGGGTTTTGGGCGG

52022 52037 51522 55184 CDS product DNA polymerase catalytic subunit 52022-52037 4:01:01 GGTGAGGTGCGGCAGG

52213 52238 51522 55184 CDS product DNA polymerase catalytic subunit 52213-52238 5:02:01 CCTCTGCTGCCGCCGTGGCCACATCC

52462 52481 51522 55184 CDS product DNA polymerase catalytic subunit 52462-52481 5:02:01 CCCCCAGCTCCGATACCCCC

52544 52565 51522 55184 CDS product DNA polymerase catalytic subunit 52544-52565 4:01:01 GGAGTAAGGCTTGTGGTTTCGG

52704 52733 51522 55184 CDS product DNA polymerase catalytic subunit 52704-52733 6:03:01 CCGTTTGCCACCCCCGTGAATCCGTAAACC

52790 52805 51522 55184 CDS product DNA polymerase catalytic subunit 52790-52805 4:01:01 GGGGGTGCTGGTGGGG

53080 53131 51522 55184 CDS product DNA polymerase catalytic subunit 53080-53131 9:06:02
CCTTTGCTCCCTGGTAGCCCACGGCCCTCCCGACGCCGGGCTTCCCGCCTCC
53164 53182 51522 55184 CDS product DNA polymerase catalytic subunit 53164-53182 4:01:01
CCGGGGTACCGTCCACACC
53395 53411 51522 55184 CDS product DNA polymerase catalytic subunit 53395-53411 4:01:01
CCACCGCCGATAGTTCC
53442 53459 51522 55184 CDS product DNA polymerase catalytic subunit 53442-53459 4:01:01
CCCACCAGCCTAGAGTCC
53475 53504 51522 55184 CDS product DNA polymerase catalytic subunit 53475-53504 5:02:01
CCTATTACGCCCCTCCGGTCAGGCCCTCCC
54014 54041 51522 55184 CDS product DNA polymerase catalytic subunit 54014-54041 4:01:01
GGAGATCTGGATGACCAGGTCCTCTTGG
54138 54160 51522 55184 CDS product DNA polymerase catalytic subunit 54138-54160 4:01:01
GGTATCGGCTCCAGGTTATCGGG
54232 54251 51522 55184 CDS product DNA polymerase catalytic subunit 54232-54251 4:01:01
GGGTGCCGGGTCGCAGGCGG
54367 54381 51522 55184 CDS product DNA polymerase catalytic subunit 54367-54381 4:01:01 GGCGGCTGCTGGAGG
54480 54508 51522 55184 CDS product DNA polymerase catalytic subunit 54480-54508 8:05:02
CCGCCCCACCATCCCCCGCCGCTGCCGCC
54519 54542 51522 55184 CDS product DNA polymerase catalytic subunit 54519-54542 5:02:01
CCGCCGTTGCCATTTCCCAGGGCC
54622 54638 51522 55184 CDS product DNA polymerase catalytic subunit 54622-54638 5:02:01
CCACCTCCGCCTTTGCC

54831 54889 51522 55184 CDS product DNA polymerase catalytic subunit 54831-54889 10:07:02
CCATCGCCCCGAAAATCCTGGCCGTTCCAAACCCGAATCCTGCGAGGCCAGCAACCTCC
54979 54990 51522 55184 CDS product DNA polymerase catalytic subunit 54979-54990 4:01:01 GGTCGGCGGGGG
55665 55682 55453 59082 CDS product single-stranded DNA-binding protein 55665-55682 4:01:01
GGACTGGGTGGTACTGGG
55737 55753 55453 59082 CDS product single-stranded DNA-binding protein 55737-55753 4:01:01
GGAGGCTCGGTCATCGG
55768 55782 55453 59082 CDS product single-stranded DNA-binding protein 55768-55782 4:01:01 CCCCCAACCTCACCC
55889 55907 55453 59082 CDS product single-stranded DNA-binding protein 55889-55907 4:01:01
CCTGTCTCCGGAGACCACC
56177 56193 55453 59082 CDS product single-stranded DNA-binding protein 56177-56193 4:01:01
GGGTCCGGCGGCTGTGG
56229 56250 55453 59082 CDS product single-stranded DNA-binding protein 56229-56250 4:01:01
GGAGCGGCCCACCTGGCCTTGG
56315 56338 55453 59082 CDS product single-stranded DNA-binding protein 56315-56338 4:01:01
GGGAAACGCCGGCAAGGGTTCGGG
56571 56589 55453 59082 CDS product single-stranded DNA-binding protein 56571-56589 4:01:01
GGACTGGTAGGAGCCATGG
56634 56659 55453 59082 CDS product single-stranded DNA-binding protein 56634-56659 6:03:01
GGGGGCCCGGCAGACGGCAAGGATGG
56667 56702 55453 59082 CDS product single-stranded DNA-binding protein 56667-56702 8:05:02
CCTTCCTACCACCGCTTCTACCTAATAGCCGCCCCC
57262 57284 55453 59082 CDS product single-stranded DNA-binding protein 57262-57284 5:02:01

CCAGCCCCTGCTCAGCCCCCACC
57708 57728 55453 59082 CDS product single-stranded DNA-binding protein 57708-57728 5:02:01
CCCGCCTCTGAGCCGCCCACC
57838 57869 55453 59082 CDS product single-stranded DNA-binding protein 57838-57869 5:02:01
CCAGAGCCAGGGTGGCCGGCATGGCCAGCGCC
57847 57861 55453 59082 CDS product single-stranded DNA-binding protein 57847-57861 4:01:01 GGGTGGCCGGCATGG
57944 57968 55453 59082 CDS product single-stranded DNA-binding protein 57944-57968 4:01:01
CCTCTTTCCCCGGGGACACCCCCCC
58003 58027 55453 59082 CDS product single-stranded DNA-binding protein 58003-58027 4:01:01
CCCTGCTCCAGCGCAACCAGATGCC
58475 58504 55453 59082 CDS product single-stranded DNA-binding protein 58475-58504 6:03:01
GGCAGGCAACTGGAACGGTCTCAACGGTGG
58586 58608 55453 59082 CDS product single-stranded DNA-binding protein 58586-58608 6:03:01
GGCCGGGGGATTTGGCACGGGGG
58815 58848 55453 59082 CDS product single-stranded DNA-binding protein 58815-58848 6:03:01
GGAGAGTGGAGCGTGGAGGCTGCCCAGGAGCTGG
58962 58977 55453 59082 CDS product single-stranded DNA-binding protein 58962-58977 4:01:01 GGTGGCCTGGCCATGG
59790 59815 59243 61570 CDS product DNA packaging terminase subunit 2 59790-59815 4:01:01
GGGCAGCGGCATAGTGGTACCCCCGG
59826 59844 59243 61570 CDS product DNA packaging terminase subunit 2 59826-59844 4:01:01
CCCCACCCATCCCTGCTCC
60008 60051 59243 61570 CDS product DNA packaging terminase subunit 2 60008-60051 8:05:02
GGCGCGCGGCGGCGGAAGCGGCTCTCAGGGCCCTCGAGGCCAGG

60565 60583 59243 61570 CDS product DNA packaging terminase subunit 2 60565-60583 4:01:01 GGGGCCGGCGAGTTGGGGG
61468 61492 59243 61570 CDS product DNA packaging terminase subunit 2 61468-61492 5:02:01 GGGGCAATTGGCGCGGAGACGGTGG
61591 61603 61432 64374 CDS product envelope glycoprotein B 61591-61603 4:01:01 CCACCCCCCACCC
61684 61708 61432 64374 CDS product envelope glycoprotein B 61684-61708 6:03:01 CCGTACCCACCACGCCAAGCCCCCC
61846 61871 61432 64374 CDS product envelope glycoprotein B 61846-61871 7:04:01 CCTGCCCACCGCCTACCGGATCCACC
62068 62088 61432 64374 CDS product envelope glycoprotein B 62068-62088 5:02:01 GGGTTCCGGTTTCGGTGGAGG
62226 62252 61432 64374 CDS product envelope glycoprotein B 62226-62252 5:02:01 CCGGGGGCCAGAGCCTGGCAGACCACC
62273 62291 61432 64374 CDS product envelope glycoprotein B 62273-62291 4:01:01 GGGGTGGATGCCATGGAGG
62327 62342 61432 64374 CDS product envelope glycoprotein B 62327-62342 5:02:01 GGAGGTGGAGGCGCGG
62344 62378 61432 64374 CDS product envelope glycoprotein B 62344-62378 6:03:01 CCGTCTACCCCTACGACTCCTTCGCCCTGTCCACC
63009 63027 61432 64374 CDS product envelope glycoprotein B 63009-63027 4:01:01 CCAGAACCTCAGCCAACCC
63080 63094 61432 64374 CDS product envelope glycoprotein B 63080-63094 4:01:01 GGAGGCAACGGCAGG
63097 63119 61432 64374 CDS product envelope glycoprotein B 63097-63119 4:01:01 CCGATTCTTCCAACGTCACCGCC
63290 63315 61432 64374 CDS product envelope glycoprotein B 63290-63315 4:01:01 CCCGAGCGCCATAGTCTCCGCAACCC
63727 63748 61432 64374 CDS product envelope glycoprotein B 63727-63748 4:01:01 GGGCTGAGCTGGAGGACACCGG
63875 63904 61432 64374 CDS product envelope glycoprotein B 63875-63904 6:03:01 GGGCCTGGGTAAAGTGGGGGAGGCCGTGGG
64214 64236 61432 64374 CDS product envelope glycoprotein B 64214-64236 4:01:01 GGTTTCGGCCCTGGAAAAGCAGG
64599 64645 64578 65060 CDS product protein V32 64599-64645 7:04:01 CCACAGCTACCGGTGATGCCCACGCCGAGGCCGCGGTTTCCCCAGCC

64991 65010 64578 65060 CDS product protein V32 64991-65010 4:01:01 CCCGACACGCCCACCGCCCC
65211 65228 65153 66142 CDS product capsid scaffold protein 65211-65228 4:01:01 GGCCGGTGGACGCACTGG
65372 65397 65153 66142 CDS product capsid scaffold protein 65372-65397 4:01:01 GGGGCTTCCCCGGCTGGCACCTGTGG
65412 65445 65153 66142 CDS product capsid scaffold protein 65412-65445 5:02:01
GGGTTGGCTGAGAGGGGATTCCCGGTAGCTGCGG
65504 65523 65153 66142 CDS product capsid scaffold protein 65504-65523 5:02:01 GGAGGAACGGCTGGTATGGG
65540 65567 65153 66142 CDS product capsid scaffold protein 65540-65567 5:02:01 GGTGCGGCTTGGGGCTGCGCGGTCACGG
65613 65629 65153 66142 CDS product capsid scaffold protein 65613-65629 4:01:01 CCGCCCCCATTAGATCC
65635 65654 65153 66142 CDS product capsid scaffold protein 65635-65654 5:02:01 GGTGGTGGACGGGCGCCCGG
66385 66407 65153 67093 CDS product capsid maturation protease 66385-66407 4:01:01 CCCCTCTATGCCAGCCTCTCTCC
66502 66520 65153 67093 CDS product capsid maturation protease 66502-66520 4:01:01 CCACGTGGCCGCGTCCCCC
66693 66713 65153 67093 CDS product capsid maturation protease 66693-66713 4:01:01 CCTCGTCCGGCCCAAGGCGCC
66903 66921 65153 67093 CDS product capsid maturation protease 66903-66921 4:01:01 CCTCCCCAACCACACACCC
67680 67695 67212 68975 CDS product DNA packaging tegument protein UL25 67680-67695 4:01:01 CCCCCGCTCGCCCCCC
67946 67968 67212 68975 CDS product DNA packaging tegument protein UL25 67946-67968 4:01:01
GGGTCCGACGGCGGTGCTCGTGG
68061 68071 67212 68975 CDS product DNA packaging tegument protein UL25 68061-68071 4:01:01 CCCCCTCCTCC
68120 68153 67212 68975 CDS product DNA packaging tegument protein UL25 68120-68153 6:03:01
CCCGACCTTTCCGAGCGTTCCGTCTCCGAGTGCC
68234 68255 67212 68975 CDS product DNA packaging tegument protein UL25 68234-68255 4:01:01
GGCGCGATGGGCTAAATGGAGG
68392 68405 67212 68975 CDS product DNA packaging tegument protein UL25 68392-68405 4:01:01 CCACCGGCCGCTCC
68435 68468 67212 68975 CDS product DNA packaging tegument protein UL25 68435-68468 6:03:01

CCAGGTGCCGAATACCACCCCGTTTGAGCCGCCC

68617 68634 67212 68975 CDS product DNA packaging tegument protein UL25 68617-68634 4:01:01 GGGGCGTGGCGGCGTCGG

68771 68796 67212 68975 CDS product DNA packaging tegument protein UL25 68771-68796 5:02:01 GGCCGCTGCGGCGGCTCTATGGCGGG

69273 69295 69079 69897 CDS product nuclear protein UL24 69273-69295 4:01:01 GGCTTTGGCCTTGGTTCCTTTGG

69954 69971 69910 70968 CDS product thymidine kinase 69954-69971 4:01:01 GGGGCGCCGGTCAGGCGG

70211 70242 69910 70968 CDS product thymidine kinase 70211-70242 6:03:01 CCAGAGCCGCTTTACCACGCCCTACCTTATCC

70322 70343 69910 70968 CDS product thymidine kinase 70322-70343 5:02:01 CCGCCACCCAGTCGCCTCTGCC

70402 70429 69910 70968 CDS product thymidine kinase 70402-70429 5:02:01 CCATGGTTGCCACCCTACCCAGGGAACC

70590 70617 69910 70968 CDS product thymidine kinase 70590-70617 5:02:01 GGGAGAGTATGGCGCGACGGCTGGGGGG

71266 71282 71192 73738 CDS product envelope glycoprotein H 71266-71282 4:01:01 CCCGTCCCAGCCACTCC

71479 71537 71192 73738 CDS product envelope glycoprotein H 71479-71537 9:06:02 CCTCCGCAACACCAACCCTGTGTCGCCAACGGGCCGGAACCTGGGGACCCACGCGGGCC

72140 72157 71192 73738 CDS product envelope glycoprotein H 72140-72157 4:01:01 GGATCTCCAGGGAGGTGG

72381 72400 71192 73738 CDS product envelope glycoprotein H 72381-72400 5:02:01 CCTCCGCCTCATTGTCCCCC

72609 72627 71192 73738 CDS product envelope glycoprotein H 72609-72627 4:01:01 GGTGGCCTTAGCGGACCGG

72751 72772 71192 73738 CDS product envelope glycoprotein H 72751-72772 4:01:01 CCCGAGCCAGGCGCGCCCGCCC

73577 73599 71192 73738 CDS product envelope glycoprotein H 73577-73599 4:01:01 CCGCCTTCGCGTCCTATTCGTCC

74804 74825 74632 76224 CDS product tegument protein UL21 74804-74825 5:02:01 CCTGCGCCGCTCCTCCGATTCC

74988 75004 74632 76224 CDS product tegument protein UL21 74988-75004 4:01:01 GGGATCGGTGGGTTGGG

75075 75101 74632 76224 CDS product tegument protein UL21 75075-75101 5:02:01 GGCCACCAGGTGTGGGTCTGGGAGGGG

75453 75477 74632 76224 CDS product tegument protein UL21 75453-75477 4:01:01 GGGCTTTGGAATCAGGCGCACCTGG

75673 75690 74632 76224 CDS product tegument protein UL21 75673-75690 4:01:01 GGGGGCAACTCGGTTAGG
75910 75933 74632 76224 CDS product tegument protein UL21 75910-75933 4:01:01 CCAAAGGCCTGGGCGCCCTGGGCC
76095 76115 74632 76224 CDS product tegument protein UL21 76095-76115 4:01:01 GGCCGTGGCATGGGGTCTAGG
76997 77026 76793 77512 CDS product envelope protein UL20 76997-77026 4:01:01
CCCACGTCGTCCTGTTTGCCATTTCTGCCC
78103 78129 77703 81833 CDS product major capsid protein 78103-78129 5:02:01 GGCAGAGGCCCTGGGCCTTATCGGGGG
78465 78478 77703 81833 CDS product major capsid protein 78465-78478 4:01:01 CCCCCTCCGTCGCC
78853 78876 77703 81833 CDS product major capsid protein 78853-78876 4:01:01 CCAGGCGACCAACGTTCCGTACCC
78929 78941 77703 81833 CDS product major capsid protein 78929-78941 4:01:01 CCGGCCACCGACC
78971 79015 77703 81833 CDS product major capsid protein 78971-79015 9:06:02
CCAACCCCCGGACAGCCAGACCCTCGCACCTACCCACCCCAAACC
79173 79186 77703 81833 CDS product major capsid protein 79173-79186 4:01:01 CCCTACCCCCAGCC
79444 79461 77703 81833 CDS product major capsid protein 79444-79461 5:02:01 CCCCCTCCCCCTATGTCC
79815 79840 77703 81833 CDS product major capsid protein 79815-79840 4:01:01 CCGAGTACACCGTTCCCGGAGAAGCC
79886 79902 77703 81833 CDS product major capsid protein 79886-79902 4:01:01 CCGGCCCTGCTACCGCC
79935 79985 77703 81833 CDS product major capsid protein 79935-79985 9:06:02
GGGCCGACATGGGCAGGGCCAGGGCTCAGGAGCTATGGGTGGATGGGGTGG
80030 80051 77703 81833 CDS product major capsid protein 80030-80051 4:01:01 GGAAACACCGGCGGCCATTTGG
80089 80112 77703 81833 CDS product major capsid protein 80089-80112 4:01:01 CCCGATTGTACCTCACCACGACCC
80245 80255 77703 81833 CDS product major capsid protein 80245-80255 4:01:01 GGGGGCGGAGG
80262 80316 77703 81833 CDS product major capsid protein 80262-80316 10:07:02
CCCCAACCAGCCCCAGCGACCCGCGCCATCCGCTGAACCCACGCCACCTAGTGCC
80346 80372 77703 81833 CDS product major capsid protein 80346-80372 4:01:01 CCAGAGTGGCCGTCGACACCGACGCCC

80454 80464 77703 81833 CDS product major capsid protein 80454-80464 4:01:01 CCGCCACCGCC
80953 80966 77703 81833 CDS product major capsid protein 80953-80966 4:01:01 GGCGGTTGGGGGGG
81001 81015 77703 81833 CDS product major capsid protein 81001-81015 4:01:01 GGACTTGGGGGTGGG
81021 81040 77703 81833 CDS product major capsid protein 81021-81040 5:02:01 CCGCCGCCTACGCAGCCGCC
81169 81192 77703 81833 CDS product major capsid protein 81169-81192 4:01:01 CCTTGGGCCCCAGGGCCCAAGGCC
81234 81250 77703 81833 CDS product major capsid protein 81234-81250 4:01:01 CCCACGGCCAGGCCGCC
81401 81432 77703 81833 CDS product major capsid protein 81401-81432 7:04:01 CCGGCCTACCCGAGCCGCGCCACCGTTAACCC
81647 81665 77703 81833 CDS product major capsid protein 81647-81665 4:01:01 GGCTCGACGGACCTGGTGG
82111 82142 82083 83027 CDS product capsid triplex subunit 2 82111-82142 5:02:01 CCTACTGCCCAGTGACCTATCTCCCGCTGACC
82664 82682 82083 83027 CDS product capsid triplex subunit 2 82664-82682 4:01:01 GGCGTGGCGCGGTCTCTGG
82739 82763 82083 83027 CDS product capsid triplex subunit 2 82739-82763 4:01:01 CCAAACCTGCTCACCCTGGGAACCC
82819 82851 82083 83027 CDS product capsid triplex subunit 2 82819-82851 6:03:01 CCAGCTCGTCCACCAGCAGCCCGTGCCCCAACC
82922 82939 82083 83027 CDS product capsid triplex subunit 2 82922-82939 4:01:01 GGTGACGTGGTCGGTGGG
83180 83196 83148 88917 CDS product DNA packaging terminase subunit 1 83180-83196 5:02:01 GGTTGGCGGGAGGCTGG
83209 83225 83148 88917 CDS product DNA packaging terminase subunit 1 83209-83225 4:01:01 CCATCACCAGGGCCACC
83776 83795 83148 88917 CDS product DNA packaging terminase subunit 1 83776-83795 4:01:01 CCACACCGGTTCCTGAAGCC

84414 84446 83148 88917 CDS product DNA packaging terminase subunit 1 84414-84446 6:03:01
CCGGCTACCCTGTTCCATACCTCCCGGCGCACC
84755 84777 84480 86600 CDS product DNA packaging tegument protein UL17 84755-84777 4:01:01
CCGACAGTTCCAGCGGACCACCC
85021 85045 84480 86600 CDS product DNA packaging tegument protein UL17 85021-85045 4:01:01
CCACCCCACAGTTTCCCAGAGCGCC
85481 85504 84480 86600 CDS product DNA packaging tegument protein UL17 85481-85504 4:01:01
CCCCCTCAGGGACCCAGGAGCCCC
85566 85583 84480 86600 CDS product DNA packaging tegument protein UL17 85566-85583 4:01:01
CCAACCCGCTCACCCCCC
85603 85618 84480 86600 CDS product DNA packaging tegument protein UL17 85603-85618 4:01:01 CCACGCCGCCGTGGCC
85734 85748 84480 86600 CDS product DNA packaging tegument protein UL17 85734-85748 4:01:01 GGGGGGGTCGGAGGG
85767 85786 84480 86600 CDS product DNA packaging tegument protein UL17 85767-85786 4:01:01
CCGGGTCCATCAACCCCCCC
85795 85817 84480 86600 CDS product DNA packaging tegument protein UL17 85795-85817 5:02:01
GGCGTGTGTGGCGGCCGGTGTGG
85901 85915 84480 86600 CDS product DNA packaging tegument protein UL17 85901-85915 4:01:01 CCGCCGCCCGTGGCC
86251 86264 84480 86600 CDS product DNA packaging tegument protein UL17 86251-86264 5:02:01 GGCGGGGGGGCGG
86665 86695 86620 87732 CDS product tegument protein UL16 86665-86695 6:03:01
CCGCGTCGTCCCCCAACCGCCAACTAACCCC
86826 86864 86620 87732 CDS product tegument protein UL16 86826-86864 8:05:02
CCTCCCCCGCCCAAGCTCACCCACACCGCCAAGTGCGCC
86985 87017 86620 87732 CDS product tegument protein UL16 86985-87017 5:02:01

CCCTTTCCCGTAGGCACCCAGAGATTCCGCGCC
87048 87067 86620 87732 CDS product tegument protein UL16 87048-87067 4:01:01 CCGTCCGACATCCCTGACCC
87170 87188 86620 87732 CDS product tegument protein UL16 87170-87188 4:01:01 GGCTCCTGGCGTCTGGTGG
87251 87278 86620 87732 CDS product tegument protein UL16 87251-87278 5:02:01 GGCTGGATGGAAAGCCAGGGGTCTGGGG
87554 87577 86620 87732 CDS product tegument protein UL16 87554-87577 4:01:01 GGTTGTGGGGATTGGGCCGCACGG
87603 87618 86620 87732 CDS product tegument protein UL16 87603-87618 4:01:01 GGATGGAAACTGGTGG
88443 88473 83148 88917 CDS product DNA packaging terminase subunit 1 88443-88473 5:02:01
GGCTACTTGGTGAAAGGCGTCTGAGGAGCGG
88568 88608 83148 88917 CDS product DNA packaging terminase subunit 1 88568-88608 6:03:01
CCTGTCGCACCTTGGGGTCCACATAAGCCTCCACGGAGGCC
88732 88763 83148 88917 CDS product DNA packaging terminase subunit 1 88732-88763 6:03:01
GGAACGACGGTCTGGTGGCGCTGGGGAGTAGG
88892 88910 83148 88917 CDS product DNA packaging terminase subunit 1 88892-88910 4:01:01
CCCTGCCCAGCACCCTACC
88957 88978 88947 89900 CDS product tegument protein UL14 88957-88978 4:01:01 GGCACGGTCTAGGCGCCAGAGG
89336 89355 88947 89900 CDS product tegument protein UL14 89336-89355 6:03:01 GGAGGAGGCGGTGAGGTCGG
89452 89484 88947 89900 CDS product tegument protein UL14 89452-89484 5:02:01
CCCTCATTCTCCCCGCTCAACCTGTCCCGCCCC
90052 90076 89369 91153 CDS product tegument serine/threonine protein kinase 90052-90076 5:02:01
GGAGGCGCCGGTAGCTATGGAGAGG
90312 90325 89369 91153 CDS product tegument serine/threonine protein kinase 90312-90325 4:01:01
CCCTCCCTCCGTCC
90882 90906 89369 91153 CDS product tegument serine/threonine protein kinase 90882-90906 6:03:01

CCCTGCCCTATTTCCCACCACCCCC

91051 91065 89369 91153 CDS product tegument serine/threonine protein kinase 91051-91065 4:01:01 CCGTCCGCCTTTACC

91472 91510 91135 92832 CDS product deoxyribonuclease 91472-91510 6:03:01 GGTAGGAGAGATGGAGCTAGAGGGGATCGTGGAACGCGG

91544 91561 91135 92832 CDS product deoxyribonuclease 91544-91561 4:01:01 GGCTGGCTTTGTGGCCGG

91889 91906 91135 92832 CDS product deoxyribonuclease 91889-91906 4:01:01 GGTGGATCCTGGATGCGG

92428 92460 91135 92832 CDS product deoxyribonuclease 92428-92460 5:02:01 GGGACACAGGAGAGTTGGCGCTGTCGGTTCCGG

92521 92538 91135 92832 CDS product deoxyribonuclease 92521-92538 4:01:01 CCGGCCATTTTCCCGACC

92702 92730 91135 92832 CDS product deoxyribonuclease 92702-92730 4:01:01 GGTGATTGTGGACCGCGAGGGTTGCTGGG

92903 92929 92784 93008 CDS product myristylated tegument protein 92903-92929 7:04:01 CCCCCACCCCCACCTCTCCCGAAACCC

93452 93468 93120 94472 CDS product envelope glycoprotein M 93452-93468 4:01:01 GGCTTTGGATGCGGTGG

93477 93503 93120 94472 CDS product envelope glycoprotein M 93477-93503 5:02:01 GGGGCCTGGACGGAAACCATGGCATGG

93775 93794 93120 94472 CDS product envelope glycoprotein M 93775-93794 4:01:01 CCGCGCGTCCGGGTCCGGCC

93925 93941 93120 94472 CDS product envelope glycoprotein M 93925-93941 4:01:01 CCGCCCCCAGGACTGCC

94031 94062 93120 94472 CDS product envelope glycoprotein M 94031-94062 5:02:01 GGTCGTTGGCCGGGTGCACCCGGCCACCGCGG

94040 94058 93120 94472 CDS product envelope glycoprotein M 94040-94058 4:01:01 CCGGGTGCACCCGGCCACC

94217 94238 93120 94472 CDS product envelope glycoprotein M 94217-94238 4:01:01 CCGCATCCACACTCCATCGACC

94496 94513 94390 97053 CDS product DNA replication origin-binding helicase 94496-94513 4:01:01 GGGCGGCAGAGCGGAAGG

94578 94599 94390 97053 CDS product DNA replication origin-binding helicase 94578-94599 4:01:01
GGGGGCGACCTGGAAGAGTGGG
94659 94679 94390 97053 CDS product DNA replication origin-binding helicase 94659-94679 5:02:01
CCTCCGCCCAACAACCCGTCC
95120 95146 94390 97053 CDS product DNA replication origin-binding helicase 95120-95146 5:02:01
GGTGGATTTCTTGGCTTCGGCGCGCGG
95283 95304 94390 97053 CDS product DNA replication origin-binding helicase 95283-95304 4:01:01
GGAACCAAGGTTATGGAGACGG
95765 95778 94390 97053 CDS product DNA replication origin-binding helicase 95765-95778 4:01:01
GGGTGGGTGGCCGG
96257 96288 94390 97053 CDS product DNA replication origin-binding helicase 96257-96288 6:03:01
CCTGGGCGCCTGCCTGCGCCTCCCAGCGGCCC
96474 96498 94390 97053 CDS product DNA replication origin-binding helicase 96474-96498 5:02:01
GGGGGGTCCGGGCTGGACATGTCGG
97012 97032 94390 97053 CDS product DNA replication origin-binding helicase 97012-97032 4:01:01
GGCCACAGGTTTACGGAGCGG
97317 97342 97069 99324 CDS product helicase-primase subunit 97317-97342 4:01:01
GGGTGCTGGCCCCTGGCTCCCCTGGG
97371 97386 97069 99324 CDS product helicase-primase subunit 97371-97386 4:01:01 GGTGGCATAACGGCGG
97468 97481 97069 99324 CDS product helicase-primase subunit 97468-97481 4:01:01 CCCTCCGACCCGCC
97490 97504 97069 99324 CDS product helicase-primase subunit 97490-97504 4:01:01 GGGTGAAGGCGGTGG
97544 97570 97069 99324 CDS product helicase-primase subunit 97544-97570 5:02:01
GGTGGAGGTAGACTTGGACCCAGCGGG

98112 98131 97069 99324 CDS product helicase-primase subunit 98112-98131 4:01:01 GGGCTGTGGCCGGCTCTAGG
98153 98225 97069 99324 CDS product helicase-primase subunit 98153-98225 13:10:03
GGCTCCATGGGACCCGGAAGACAGGTGGCCCGGTGAATCGGAGGCAAAACGGGTAGAGGGGGCGGTACACAGG
98235 98265 97069 99324 CDS product helicase-primase subunit 98235-98265 6:03:01
GGTACCGAGGATGATTGGGGGGCTGGGCGGG
98471 98504 97069 99324 CDS product helicase-primase subunit 98471-98504 5:02:01
CCCCGTAGCCTATAGGTCCATCATAGCCCTATCC
98577 98596 97069 99324 CDS product helicase-primase subunit 98577-98596 4:01:01 GGCTTTTGGGGGGCAGCCGG
99558 99583 99421 100332 CDS product tegument protein UL7 99558-99583 5:02:01 GGGGCAGGGGGTGATGGGTCTTTTGG
99695 99715 99421 100332 CDS product tegument protein UL7 99695-99715 4:01:01 GGTCAAAGGGGTTGTGGTTGG
100067 100082 99421 100332 CDS product tegument protein UL7 100067-100082 4:01:01 CCGAGCCCTCCTGTCC
100143 100169 99421 100332 CDS product tegument protein UL7 100143-100169 6:03:01
GGTCGTGAAGGAGGGGGGTAGGCCTGG
100803 100827 100130 102391 CDS product capsid portal protein 100803-100827 5:02:01
CCTCGTGCCCGAGTCCTCCCGCGCC
100882 100897 100130 102391 CDS product capsid portal protein 100882-100897 4:01:01 CCCGTCTGCCGCCGCC
101024 101046 100130 102391 CDS product capsid portal protein 101024-101046 4:01:01 CCCTCCAACATCCCGTTAATGCC
101307 101362 100130 102391 CDS product capsid portal protein 101307-101362 10:07:02
CCACGGCCTTGGAGGCCCACCCAGGGCCCTGGGCCTCGTCCTTTTTCCTGCCCACC
101605 101627 100130 102391 CDS product capsid portal protein 101605-101627 4:01:01 GGCCACGCTGGCGGTGCACCTGG
101623 101653 100130 102391 CDS product capsid portal protein 101623-101653 7:04:01
CCTGGCCAGCTCCACCACGGCCTCCAACCCC
101759 101780 100130 102391 CDS product capsid portal protein 101759-101780 5:02:01 GGGTGGACGGTCGCGCCGGGGG

101839 101873 100130 102391 CDS product capsid portal protein 101839-101873 6:03:01
GGCAAAGCTGGCGGTGCCGTCGGGTTCGCTGGCGG
102900 102915 102375 105020 CDS product helicase-primase helicase subunit 102900-102915 4:01:01
CCTCGAGCCCGCCTCC
103831 103859 102375 105020 CDS product helicase-primase helicase subunit 103831-103859 5:02:01
GGAACACGGAGTAGTGGTGGCCAGAACGG
104078 104120 102375 105020 CDS product helicase-primase helicase subunit 104078-104120 8:05:02
GGGCTGGACGAGGGGAGGGTCCGGGAGGCGTACAGGCGCATGG
104722 104762 102375 105020 CDS product helicase-primase helicase subunit 104722-104762 7:04:01
GGAGTCGGTGGACGACGTGGAGCTGGACATGGCCACCGCGG
105341 105353 105070 105747 CDS product nuclear protein UL4 105341-105353 4:01:01 CCCCCTGACCACC
105356 105375 105070 105747 CDS product nuclear protein UL4 105356-105375 5:02:01 GGACGGGTCGGTACTGGTGG
105547 105581 105070 105747 CDS product nuclear protein UL4 105547-105581 7:04:01
CCGTATGTTCCACCGCTGCCATAACCGACGCCTCC
105684 105711 105070 105747 CDS product nuclear protein UL4 105684-105711 4:01:01 GGTACCAACGGAGCCTCGGCGTTGCTGG
105714 105734 105070 105747 CDS product nuclear protein UL4 105714-105734 4:01:01 CCGTGTTTTCCCTGCCTTTCC
105986 106004 105877 106416 CDS product protein V57 105986-106004 4:01:01 CCTCGCCGCGGCTGCCGCC
106136 106205 105877 106416 CDS product protein V57 106136-106205 11:08:02
CCATGGCCAGGGCCCCGAGCGTTCCAGAAGGCCTCTCCGAGAGGGCCAGCTGTCCGTCGCCACCCGCCCC
106323 106347 105877 106416 CDS product protein V57 106323-106347 6:03:01 GGCGGCAAGCTTGGCGGCGGCCCGG
106768 106787 106478 107116 CDS product nuclear protein UL3 106768-106787 4:01:01 GGAGAACGAGGAGGTTTTGG
106945 106965 106478 107116 CDS product nuclear protein UL3 106945-106965 4:01:01 GGATTCCACGGAAAGGTCGGG
106976 106994 106478 107116 CDS product nuclear protein UL3 106976-106994 4:01:01 GGGGTTGGGGTGGAAGTGG

107014 107025 106478 107116 CDS product nuclear protein UL3 107014-107025 4:01:01 GGGGGCTGGTGG
107077 107088 106478 107116 CDS product nuclear protein UL3 107077-107088 4:01:01 CCACCCCCACCC
107350 107374 107206 108144 CDS product uracil-DNA glycosylase 107350-107374 4:01:01 GGGGAGCAGGACTTCTGGGCGTGGG
107429 107454 107206 108144 CDS product uracil-DNA glycosylase 107429-107454 6:03:01 CCGCCCTGACCAGCCTGTGCCACCCC
107518 107546 107206 108144 CDS product uracil-DNA glycosylase 107518-107546 6:03:01 CCCTGTTCCGCCCACCTTTCCAAAAACCC
107660 107685 107206 108144 CDS product uracil-DNA glycosylase 107660-107685 5:02:01 GGCCCGGCGCGTGGTACGGATCCTGG
110391 110421 110387 111985 CDS product ubiquitin E3 ligase ICP0 110391-110421 4:01:01 GGTTTCTCCGGTATCTTTGGCAGTTGTGGGG
110528 110544 110387 111985 CDS product ubiquitin E3 ligase ICP0 110528-110544 4:01:01 GGGTAGGGCACCGGGGG
110635 110656 110387 111985 CDS product ubiquitin E3 ligase ICP0 110635-110656 4:01:01 CCCACCAAATAGCCCTGCTCCC
110849 110874 110387 111985 CDS product ubiquitin E3 ligase ICP0 110849-110874 5:02:01 GGTGCAACGGGTTGGTCCCGGGGGGG
110981 111001 110387 111985 CDS product ubiquitin E3 ligase ICP0 110981-111001 4:01:01 GGCTGATCGGTGGTATTCAGG
111082 111106 110387 111985 CDS product ubiquitin E3 ligase ICP0 111082-111106 5:02:01 GGTGCGGGAGTTGGTTCTGGGCTGG
111153 111177 110387 111985 CDS product ubiquitin E3 ligase ICP0 111153-111177 5:02:01 CCGGCCTTCTCCCCCGTCTGCGCCC

111181 111195 110387 111985 CDS product ubiquitin E3 ligase ICP0 111181-111195 4:01:01 GGTGGGTGGCCCCGG

111281 111301 110387 111985 CDS product ubiquitin E3 ligase ICP0 111281-111301 4:01:01 GGGTCGGTTAGGTTGTTGGGG

111589 111610 110387 111985 CDS product ubiquitin E3 ligase ICP0 111589-111610 5:02:01 CCTTTCGACCAGCCAGTCCACC

114148 114181 114128 118591 CDS product transcriptional regulator ICP4 114148-114181 7:04:01 CCAGTCTCCTCCGTCCTCGTCGTCCGACACCACC

114193 114238 114128 118591 CDS product transcriptional regulator ICP4 114193-114238 8:05:02 GGGAGCGCGGCGGAGGGCCTGGGCCACGCCGGGGCTCGGGCCGGGG

114246 114270 114128 118591 CDS product transcriptional regulator ICP4 114246-114270 5:02:01 CCACCAGCTCCACATCGCCGGCCCC

114350 114374 114128 118591 CDS product transcriptional regulator ICP4 114350-114374 4:01:01 CCGAACCCGGGCTCCCAGTCGACCC

114377 114408 114128 118591 CDS product transcriptional regulator ICP4 114377-114408 6:03:01 GGGGCTCGGCGGCGGGGAGCCTCGGTGATGGG

114417 114438 114128 118591 CDS product transcriptional regulator ICP4 114417-114438 4:01:01 GGGGCTCGGCCTCGGCGTCGGG

114492 114525 114128 118591 CDS product transcriptional regulator ICP4 114492-114525 6:03:01 CCGAGGGCTCCACGGCCCCAGCGCCGCGCCGGCC

114522 114544 114128 118591 CDS product transcriptional regulator ICP4 114522-114544 4:01:01 GGCCGCAGGCGAGGTACACGGGG

114570 114586 114128 118591 CDS product transcriptional regulator ICP4 114570-114586 4:01:01 GGTTGGCCGCGCGGTGG

114614 114644 114128 118591 CDS product transcriptional regulator ICP4 114614-114644 5:02:01
GGGTCGTGGAACCCGAGGCCCTCGGCCTGGG
114625 114663 114128 118591 CDS product transcriptional regulator ICP4 114625-114663 6:03:01
CCCGAGGCCCTCGGCCTGGGCCCGCATGTCCTTGCAGCC
114673 114691 114128 118591 CDS product transcriptional regulator ICP4 114673-114691 4:01:01
GGGCAGGACGCGCTGGCGG
114751 114769 114128 118591 CDS product transcriptional regulator ICP4 114751-114769 4:01:01
GGCCACGTTGGAGGAGCGG
114849 114894 114128 118591 CDS product transcriptional regulator ICP4 114849-114894 8:05:02
GGGTGCTGGAGGTGAGGACCGCGCGGCTGAGGTGGCGCTCCCGGGG
114968 114985 114128 118591 CDS product transcriptional regulator ICP4 114968-114985 4:01:01
CCGTCCTGGGGCCAATCC
115024 115048 114128 118591 CDS product transcriptional regulator ICP4 115024-115048 5:02:01
GGCGGAGCCCAGGCGCAGGCAGAGG
115063 115082 114128 118591 CDS product transcriptional regulator ICP4 115063-115082 4:01:01
GGTGAAGGCCAGGTCCCCGG
115116 115151 114128 118591 CDS product transcriptional regulator ICP4 115116-115151 6:03:01
CCGAGACGTCCGGGGCCCCGGTCCAGTTGCCGGCCC
115147 115190 114128 118591 CDS product transcriptional regulator ICP4 115147-115190 7:04:01
GGCCCAGGCGTGGGACCGCTTGGTGAGGATGCGGTTCCCCAGGG
115183 115216 114128 118591 CDS product transcriptional regulator ICP4 115183-115216 6:03:01
CCCCAGGGCCGCCAGCAGCGCCGAGAGTCCCCCC
115255 115270 114128 118591 CDS product transcriptional regulator ICP4 115255-115270 4:01:01 GGGGCGGGAGGCCGGG

115266 115294 114128 118591 CDS product transcriptional regulator ICP4 115266-115294 5:02:01
CCGGGAGTCCGCCCAGCAGGTCCTCGTCC
115314 115330 114128 118591 CDS product transcriptional regulator ICP4 115314-115330 4:01:01
CCACCACCTTCACGTCC
115379 115398 114128 118591 CDS product transcriptional regulator ICP4 115379-115398 4:01:01
CCGCTGGCCGCCAGCTCCCC
115409 115428 114128 118591 CDS product transcriptional regulator ICP4 115409-115428 4:01:01
CCGTCCCGGGCCGGGGGGCC
115437 115472 114128 118591 CDS product transcriptional regulator ICP4 115437-115472 6:03:01
GGGCCGCGATGGTGGCCAGGGCCTGGGGATCGAAGG
115481 115507 114128 118591 CDS product transcriptional regulator ICP4 115481-115507 4:01:01
GGGCGCCAGGCCTCGGGGAACAGCTGG
115620 115679 114128 118591 CDS product transcriptional regulator ICP4 115620-115679 11:08:02
GGTCGGGGCCCTCGGCTGGCATGGGTCCCAGGGCCCGGGGAGCCTGGTGGCCCGGGGTGG
115646 115685 114128 118591 CDS product transcriptional regulator ICP4 115646-115685 6:03:01
CCCAGGGCCCGGGGAGCCTGGTGGCCCGGGGTGGCCACCC
115693 115705 114128 118591 CDS product transcriptional regulator ICP4 115693-115705 4:01:01 GGGGGCCGGAGGG
115737 115796 114128 118591 CDS product transcriptional regulator ICP4 115737-115796 11:08:02
GGGCGGACGAGGAGAAGGAGGCCGAGGCTCCGGCCTGGGCCGCCGGCTCCAGGGGCTCGG
115758 115786 114128 118591 CDS product transcriptional regulator ICP4 115758-115786 6:03:01
CCGAGGCTCCGGCCTGGGCCGCCGGCTCC
115801 115830 114128 118591 CDS product transcriptional regulator ICP4 115801-115830 4:01:01
CCGCTTGCCGCTCTTGCCCCTGGGGCGCCC

115822 115843 114128 118591 CDS product transcriptional regulator ICP4 115822-115843 4:01:01
GGGGCGCCCGTGGATGGCACGG
115875 115898 114128 118591 CDS product transcriptional regulator ICP4 115875-115898 6:03:01
GGCTGAGGTGGGCCGGGGAGGCGG
115962 115985 114128 118591 CDS product transcriptional regulator ICP4 115962-115985 6:03:01
GGGTGGCCCGGCAGCAGGTGGCGG
115992 116007 114128 118591 CDS product transcriptional regulator ICP4 115992-116007 4:01:01 CCCCGGCGCCTCCGCC
116013 116047 114128 118591 CDS product transcriptional regulator ICP4 116013-116047 6:03:01
GGGAGTGCTGGGGGGACTGGGAGTGGGACGAGGGG
116068 116104 114128 118591 CDS product transcriptional regulator ICP4 116068-116104 7:04:01
GGGGACGGTGGGCCACAGGGGCGGCAGGGTCTGAAGG
116107 116127 114128 118591 CDS product transcriptional regulator ICP4 116107-116127 4:01:01
CCCCTCCGCGGCCGCGGAGCC
116116 116136 114128 118591 CDS product transcriptional regulator ICP4 116116-116136 4:01:01
GGCCGCGGAGCCGGAGAAGGG
116146 116171 114128 118591 CDS product transcriptional regulator ICP4 116146-116171 4:01:01
GGGCGAGGACGATGAGGGCTGCTGGG
116180 116201 114128 118591 CDS product transcriptional regulator ICP4 116180-116201 4:01:01
GGTGGGGCCAGCAGGGACACGG
116202 116224 114128 118591 CDS product transcriptional regulator ICP4 116202-116224 6:03:01
CCTCCCCCAACATCCCCCCGACC
116245 116291 114128 118591 CDS product transcriptional regulator ICP4 116245-116291 8:05:02
GGCCTGGGTGACGGTCCAGGCCGAGGCCCGGGCCCGGGCCCCCTCGG

116260 116287 114128 118591 CDS product transcriptional regulator ICP4 116260-116287 6:03:01
CCAGGCCGAGGCCCGGGCCCGGGCCCCC
116304 116332 114128 118591 CDS product transcriptional regulator ICP4 116304-116332 6:03:01
CCAGCGGCGCCACGGTCCGGGCCACCACC
116365 116386 114128 118591 CDS product transcriptional regulator ICP4 116365-116386 5:02:01
GGAGCCCAGGCGGTGGGTAGGG
116394 116426 114128 118591 CDS product transcriptional regulator ICP4 116394-116426 5:02:01
CCCCGAGGAGCCTGGCCCGCTCGACCAGGTCCC
116448 116467 114128 118591 CDS product transcriptional regulator ICP4 116448-116467 5:02:01
CCGCCGTCTCCAGCCCGGCC
116488 116509 114128 118591 CDS product transcriptional regulator ICP4 116488-116509 5:02:01
GGGAGGGCAGGCGGGAGAGGGG
116553 116567 114128 118591 CDS product transcriptional regulator ICP4 116553-116567 4:01:01 GGAGGCGCAGGAGGG
116626 116672 114128 118591 CDS product transcriptional regulator ICP4 116626-116672 9:06:02
GGCGGACGGCCTGGGCGCCCTGGTCCCCGGAGCGGCCGCGGCCGCGG
116635 116677 114128 118591 CDS product transcriptional regulator ICP4 116635-116677 6:03:01
CCTGGGCGCCCTGGTCCCCGGAGCGGCCGCGGCCGCGGCAGCC
116724 116754 114128 118591 CDS product transcriptional regulator ICP4 116724-116754 5:02:01
GGACGCGGACCGAGGGCGCCGGGGCCTCGGG
116763 116810 114128 118591 CDS product transcriptional regulator ICP4 116763-116810 10:07:02
GGCTGGCCGCGGCAGCGGCGGCCTGGGCCGGGTAGCCGGCCACGGCGG
116784 116803 114128 118591 CDS product transcriptional regulator ICP4 116784-116803 4:01:01
CCTGGGCCGGGTAGCCGGCC

116820 116833 114128 118591 CDS product transcriptional regulator ICP4 116820-116833 4:01:01 CCGGCCTCCCCGCC
116863 116884 114128 118591 CDS product transcriptional regulator ICP4 116863-116884 5:02:01 GGCGCGCCGGAGGCTCTGGAGG
116913 116936 114128 118591 CDS product transcriptional regulator ICP4 116913-116936 5:02:01 GGCGGCTCATGGCCACCGAGGCGG
116965 116985 114128 118591 CDS product transcriptional regulator ICP4 116965-116985 4:01:01 CCCGGCCGCCATGGCGTCCCC
116991 117005 114128 118591 CDS product transcriptional regulator ICP4 116991-117005 4:01:01 GGGGCAGGGGGTTGG
117258 117291 114128 118591 CDS product transcriptional regulator ICP4 117258-117291 6:03:01 CCCACAGCCCGTCCCGCGAGTCCCCGGTCCCCCC
117321 117342 114128 118591 CDS product transcriptional regulator ICP4 117321-117342 4:01:01 CCGACCCGGGCCAGGGGTCCCC
117328 117350 114128 118591 CDS product transcriptional regulator ICP4 117328-117350 4:01:01 GGGCCAGGGGTCCCCGGACGGGG
117393 117406 114128 118591 CDS product transcriptional regulator ICP4 117393-117406 4:01:01 CCGCCGCCGGGGCC
117420 117436 114128 118591 CDS product transcriptional regulator ICP4 117420-117436 4:01:01 CCGCCGCCCCGGTAGCC
117479 117533 114128 118591 CDS product transcriptional regulator ICP4 117479-117533 9:06:02 GGGGCCTCCTCGGCCGGTGCCTGGGTCTGGGTCTGGGGTATGGGTCTGGGGTTGG
117483 117499 114128 118591 CDS product transcriptional regulator ICP4 117483-117499 4:01:01 CCTCCTCGGCCGGTGCC
117572 117615 114128 118591 CDS product transcriptional regulator ICP4 117572-117615 8:05:02 GGACGCTGGTCCTTGGGGAGCCGGTGAGGGCTCCGGCCCGGCGG

117710 117721 114128 118591 CDS product transcriptional regulator ICP4 117710-117721 4:01:01 GGAGGGAGGAGG
117731 117747 114128 118591 CDS product transcriptional regulator ICP4 117731-117747 4:01:01 GGGCGGCTGGGAGCGGG
117758 117799 114128 118591 CDS product transcriptional regulator ICP4 117758-117799 8:05:02 GGAGCCGGAGCGGGTGCGCCGGGGGAAGCGGCGCCGGAGCGG
117809 117832 114128 118591 CDS product transcriptional regulator ICP4 117809-117832 5:02:01 GGGGTGGCGGCCCCAGCGGCCGGG
117819 117843 114128 118591 CDS product transcriptional regulator ICP4 117819-117843 4:01:01 CCCCAGCGGCCGGGCGATCCCCTCC
117844 117887 114128 118591 CDS product transcriptional regulator ICP4 117844-117887 8:05:02 GGAGGACGGCCCGGGAGAGCCGGCGGCCGACGGGGTTTTCGCGG
117918 117930 114128 118591 CDS product transcriptional regulator ICP4 117918-117930 4:01:01 CCTCCTCGCCGCC
117978 118009 114128 118591 CDS product transcriptional regulator ICP4 117978-118009 6:03:01 CCCCGGCACCATCCGCCTGGTCGTCCTCGTCC
118051 118090 114128 118591 CDS product transcriptional regulator ICP4 118051-118090 7:04:01 CCGGACCCGGGGTGCCGGGGACCCTCCGCCCGGGGAGGCC
118082 118133 114128 118591 CDS product transcriptional regulator ICP4 118082-118133 9:06:02 GGGGAGGCCGAGGATGGAAACTCGGGCTGCGGGGACCCCGGGCAGGTCTCGG
118152 118176 114128 118591 CDS product transcriptional regulator ICP4 118152-118176 5:02:01 CCGGGTCGGCCGCGTCCCCACCGCC
118177 118201 114128 118591 CDS product transcriptional regulator ICP4 118177-118201 4:01:01 GGGTGATGAGGAGCCCGTGGCCCGG
118190 118219 114128 118591 CDS product transcriptional regulator ICP4 118190-118219 5:02:01

CCCGTGGCCCGGCGACCGTTCCCCGGGGCC
118214 118231 114128 118591 CDS product transcriptional regulator ICP4 118214-118231 4:01:01
GGGGCCACGGAGGAGTGG
118259 118295 114128 118591 CDS product transcriptional regulator ICP4 118259-118295 7:04:01
GGAGCCGGGCTGGGTGCCGGGGACGCCGGCTGGGCGG
118316 118333 114128 118591 CDS product transcriptional regulator ICP4 118316-118333 4:01:01
CCGCCGCTGCCGGCGGCC
118342 118359 114128 118591 CDS product transcriptional regulator ICP4 118342-118359 4:01:01
CCGCTTCGCCTTCCCTCC
118362 118390 114128 118591 CDS product transcriptional regulator ICP4 118362-118390 6:03:01
GGGGCTCGGGAGTCGGAGACGGCGGAGGG
118458 118478 114128 118591 CDS product transcriptional regulator ICP4 118458-118478 5:02:01
GGTCGGGCGCGGCGGGCTGGG
118492 118519 114128 118591 CDS product transcriptional regulator ICP4 118492-118519 5:02:01
GGCCGCGCTGGCTGCGCGGATGAGGGGG
121430 121473 121368 122249 CDS product regulatory protein ICP22 121430-121473 11:08:02
CCGTCCACCAGCCCCCTCATCCCGTCCCTGACCCCCTCGCCCCC
121483 121502 121368 122249 CDS product regulatory protein ICP22 121483-121502 4:01:01 CCCGTCCCCACGCTCCAGCC
121533 121566 121368 122249 CDS product regulatory protein ICP22 121533-121566 6:03:01
GGCTCCCCGGCGGCTCGGACCATCCGGAATACGG
121571 121614 121368 122249 CDS product regulatory protein ICP22 121571-121614 7:04:01
CCGCTATCCCCGCGGGCCCTGCGCCCGTACCTGGCCCGGGGGCC
121603 121619 121368 122249 CDS product regulatory protein ICP22 121603-121619 4:01:01 GGCCCGGGGGCCAGGGG

121695 121717 121368 122249 CDS product regulatory protein ICP22 121695-121717 5:02:01
CCACCTCCTCGATCCACGTGACC
121894 121925 121368 122249 CDS product regulatory protein ICP22 121894-121925 6:03:01
CCTGGAGCCGCCGAGCGACCCCCCGAATACCC
122065 122078 121368 122249 CDS product regulatory protein ICP22 122065-122078 4:01:01 GGACGTGGGGGAGG
122136 122171 121368 122249 CDS product regulatory protein ICP22 122136-122171 8:05:02
CCGATGCCCCCACCAACAACCACCACCCTACAACCC
122194 122212 121368 122249 CDS product regulatory protein ICP22 122194-122212 4:01:01 CCGCAAGCGCCAACCCCCC
122912 122959 122862 123572 CDS product virion protein US10 122912-122959 10:07:02
GGCGAGGAGTCCGGAGCGGGGACGGGGACGGGGGCGGGCGCGGACGGG
123049 123062 122862 123572 CDS product virion protein US10 123049-123062 4:01:01 GGCGGAGGCAGCGG
123081 123105 122862 123572 CDS product virion protein US10 123081-123105 5:02:01 CCGGGCCGCCCGCGGAGGCCTGGCC
123150 123170 122862 123572 CDS product virion protein US10 123150-123170 4:01:01 CCAGCCCCATGCCCGTCTTCC
123214 123236 122862 123572 CDS product virion protein US10 123214-123236 5:02:01 GGATCTCGGCGCGGCGCCGGTGG
123448 123466 122862 123572 CDS product virion protein US10 123448-123466 5:02:01 CCCCCGCCACGCAGCCGCC
123475 123532 122862 123572 CDS product virion protein US10 123475-123532 9:06:02
CCTGCGCGCCCGCGGCCGCAAGCTCCCTCTCCAGCTGCCCTCGGCCCATCGGACCGCC
124381 124402 124376 125194 CDS product virion protein V67 124381-124402 4:01:01 GGCCACGGGGGTGTCTTCGGGG
124425 124474 124376 125194 CDS product virion protein V67 124425-124474 8:05:02
GGCTGCGGGGCTGCCTGGCCCCTCTGGGGTGGGGGTCAGGGGAGCTCTGG
124618 124640 124376 125194 CDS product virion protein V67 124618-124640 5:02:01 GGCGATGGCGGGGGGAACCTTGG
124681 124697 124376 125194 CDS product virion protein V67 124681-124697 4:01:01 GGCATCGGCGGCGTCGG
124719 124738 124376 125194 CDS product virion protein V67 124719-124738 4:01:01 CCGCGGGTTCCTCCTCGCCC

124754 124781 124376 125194 CDS product virion protein V67 124754-124781 6:03:01 GGGTAGAGGAACGGCAGGCGGACGAAGG

124822 124838 124376 125194 CDS product virion protein V67 124822-124838 4:01:01 GGGCAGCGGCAGGGCGG

124851 124876 124376 125194 CDS product virion protein V67 124851-124876 7:04:01 CCTCTCCGGCCACCACCCCCTCGACC

125050 125093 124376 125194 CDS product virion protein V67 125050-125093 9:06:02 GGAGTTGGCGGCCCCCCCGGCCGCGGCTGCGGCGAAGGTGGGGG

125103 125128 124376 125194 CDS product virion protein V67 125103-125128 6:03:01 GGTGGGGCGGGTTGGCATTGCGGCGG

125155 125171 124376 125194 CDS product virion protein V67 125155-125171 5:02:01 GGTGCCGGCGGTGGCGG

125260 125290 125019 126275 CDS product virion protein US2 125260-125290 7:04:01 GGGCTAGGCGGTGGTGGGTGTAAAGGCGAGG

125430 125505 125019 126275 CDS product virion protein US2 125430-125505 14:11:03 GGCGGGCGGCGGGCGCCCGCGGGTCCCCGGGGCGGGGCGGCGTCGCGGCGGCGGCCGTGGACCGAGCGGGCGCGGG

125536 125559 125019 126275 CDS product virion protein US2 125536-125559 7:04:01 CCCCCCCCTCCGACGGCCGCCGCC

125562 125584 125019 126275 CDS product virion protein US2 125562-125584 4:01:01 GGCAGCGGCCGCCCCGGGGCGGG

126483 126511 126411 127559 CDS product serine/threonine protein kinase US3 126483-126511 7:04:01 CCACGCCTCCGCTACCACCTCCCACACCC

126533 126551 126411 127559 CDS product serine/threonine protein kinase US3 126533-126551 4:01:01 GGTGCGGCCGCGGACGAGG

126853 126872 126411 127559 CDS product serine/threonine protein kinase US3 126853-126872 4:01:01 GGACACCTTGGTATCGGGGG

127111 127137 126411 127559 CDS product serine/threonine protein kinase US3 127111-127137 5:02:01 GGAACCCGCGGACCTGGGCCTGGCTGG

127787 127817 127681 128916 CDS product envelope glycoprotein G 127787-127817 6:03:01 CCCACCAAACACCCAGCCCGAACGCCCACCC

128281  128300  127681  128916  CDS product envelope glycoprotein G  128281-128300  4:01:01 CCAGCCCACACCCGCGTGCC
128492  128506  127681  128916  CDS product envelope glycoprotein G  128492-128506  4:01:01 GGTGGGTGCGGTTGG
129252  129272  129097  131490  CDS product envelope glycoprotein J  129252-129272  5:02:01 CCCACCACGAGTCCACCTACC
129285  129313  129097  131490  CDS product envelope glycoprotein J  129285-129313  5:02:01
CCCCCCACATCAACCCACACATCCTCCCC
129379  129404  129097  131490  CDS product envelope glycoprotein J  129379-129404  4:01:01
CCAGCACAACCTCTATTCCAACATCC
129418  129437  129097  131490  CDS product envelope glycoprotein J  129418-129437  4:01:01 CCACCACAACAACCCCAACC
129466  129494  129097  131490  CDS product envelope glycoprotein J  129466-129494  4:01:01
CCGCGGCTCCCACAACGGCCGCTACAACC
129607  129626  129097  131490  CDS product envelope glycoprotein J  129607-129626  4:01:01 CCACCACTGTTCCAACAACC
129772  129869  129097  131490  CDS product envelope glycoprotein J  129772-129869  19:16:04
CCTCTGCAACCACCGCGGCTACCACCACCGCGGCTACCACCACCGCGGCTACCACCACCGCGGCTACCACCACCGCGGCTACCACCACCGCGGCTACC
129916  129959  129097  131490  CDS product envelope glycoprotein J  129916-129959  8:05:02
CCACGTCCACCCCCTCAGCTTCCACTGCCACATCTGCCACTCCC
130312  130331  129097  131490  CDS product envelope glycoprotein J  130312-130331  4:01:01 CCGACTCTTCCACCGTGCCC
130450  130486  129097  131490  CDS product envelope glycoprotein J  130450-130486  6:03:01
CCGGAAATACCACCCCATCACCTTCCCCGCGTACCCC
130553  130565  129097  131490  CDS product envelope glycoprotein J  130553-130565  4:01:01 CCACACCACCGCC
130596  130618  129097  131490  CDS product envelope glycoprotein J  130596-130618  4:01:01 GGTCGCGCAGGGGGTCGTCGGGG
130740  130763  129097  131490  CDS product envelope glycoprotein J  130740-130763  5:02:01 CCTCAGCTCCCGCCAGACCGACCC
131053  131085  129097  131490  CDS product envelope glycoprotein J  131053-131085  6:03:01
CCACCAAATCAACCACAATACCGTGTCCGGGCC

131689 131717 131583 132791 CDS product envelope glycoprotein D 131689-131717 5:02:01 GGTTCGAGGACGCCAGGATAGGCCAAAGG
132413 132435 131583 132791 CDS product envelope glycoprotein D 132413-132435 5:02:01 CCCGTGCCTCCGGATAACCACCC
132481 132519 131583 132791 CDS product envelope glycoprotein D 132481-132519 6:03:01 CCCGAAACCAGGCCAGGCGGACCCCAAACCCAATCAGCC
133413 133431 132899 134173 CDS product envelope glycoprotein I 133413-133431 4:01:01 CCCCGATCCAATGCCCACC
133614 133630 132899 134173 CDS product envelope glycoprotein I 133614-133630 4:01:01 GGTTATTGGTCAGGAGG
133631 133655 132899 134173 CDS product envelope glycoprotein I 133631-133655 4:01:01 CCCTGTTATGCCATTGGTTCCAGCC
133975 134010 132899 134173 CDS product envelope glycoprotein I 133975-134010 7:04:01 GGACAGGGGGGCGCATCGGCGGCCGAACGGAGACGG
134074 134090 132899 134173 CDS product envelope glycoprotein I 134074-134090 5:02:01 CCTCCAACCCCCAAACC
134783 134819 134406 136058 CDS product envelope glycoprotein E 134783-134819 6:03:01 CCAATCCTTGCCGAAACCCGCAGCGTCCTACAAATCC
134915 134934 134406 136058 CDS product envelope glycoprotein E 134915-134934 4:01:01 CCGCCCAAACAACCGCAACC
134952 134993 134406 136058 CDS product envelope glycoprotein E 134952-134993 7:04:01 CCCCGCCACCCGTAACCGTTCCTCAGGTTCCCGTAAAGACCC
135181 135207 134406 136058 CDS product envelope glycoprotein E 135181-135207 5:02:01 CCCGACAGCCATGGCCTGCCTGCACCC
135437 135454 134406 136058 CDS product envelope glycoprotein E 135437-135454 4:01:01 GGACATCCGGAGGCGTGG
135859 135899 134406 136058 CDS product envelope glycoprotein E 135859-135899 9:06:02 CCCACCACCTCCCAAACCAGCTCCACAGCTCCCACCATACC
135929 135952 134406 136058 CDS product envelope glycoprotein E 135929-135952 5:02:01 GGCAGGTCCGGATTCAAGGTTTGG

136186 136212 136055 136447 CDS product membrane protein US8A 136186-136212 5:02:01 CCCAGATCCGGCTCAACCTCCGATTCC

136784 136797 136783 137442 CDS product membrane protein US9 136784-136797 4:01:01 GGAGAAGGCGGAGG

136942 136955 136783 137442 CDS product membrane protein US9 136942-136955 4:01:01 CCGCTACCTCCACC

137066 137083 136783 137442 CDS product membrane protein US9 137066-137083 4:01:01 CCCGTCTCCCCCATCACC

137324 137352 136783 137442 CDS product membrane protein US9 137324-137352 6:03:01 GGCCCGCAGGCACAGGCGGCGGCGCGTGG

137987 138003 137966 138784 CDS product virion protein V67 137987-138003 5:02:01 CCGCCACCGCCGGCACC

138030 138055 137966 138784 CDS product virion protein V67 138030-138055 6:03:01 CCGCCGCAATGCCAACCCGCCCCACC

138065 138108 137966 138784 CDS product virion protein V67 138065-138108 9:06:02 CCCCCACCTTCGCCGCAGCCGCGGCCGGGGGGGCCGCCAACTCC

138282 138307 137966 138784 CDS product virion protein V67 138282-138307 7:04:01 GGTCGAGGGGGTGGTGGCCGGAGAGG

138320 138336 137966 138784 CDS product virion protein V67 138320-138336 4:01:01 CCGCCCTGCCGCTGCCC

138377 138404 137966 138784 CDS product virion protein V67 138377-138404 6:03:01 CCTTCGTCCGCCTGCCGTTCCTCTACCC

138420 138439 137966 138784 CDS product virion protein V67 138420-138439 4:01:01 GGGCGAGGAGGAACCCGCGG

138461 138477 137966 138784 CDS product virion protein V67 138461-138477 4:01:01 CCGACGCCGCCGATGCC

138518 138540 137966 138784 CDS product virion protein V67 138518-138540 5:02:01 CCAAGGTTCCCCCCGCCATCGCC

138684 138733 137966 138784 CDS product virion protein V67 138684-138733 8:05:02 CCAGAGCTCCCCTGACCCCCACCCCAGAGGGGCCAGGCAGCCCCGCAGCC

138756 138777 137966 138784 CDS product virion protein V67 138756-138777 4:01:01 CCCCGAAGACACCCCCGTGGCC

139626 139683 139588 140298 CDS product virion protein US10 139626-139683 9:06:02 GGCGGTCCGATGGGCCGAGGGCAGCTGGAGAGGGAGCTTGCGGCCGCGGGCGCGCAGG

139692 139710 139588 140298 CDS product virion protein US10 139692-139710 5:02:01 GGCGGCTGCGTGGCGGGGG

139922 139944 139588 140298 CDS product virion protein US10 139922-139944 5:02:01 CCACCGGCGCCGCGCCGAGATCC
139988 140008 139588 140298 CDS product virion protein US10 139988-140008 4:01:01 GGAAGACGGGCATGGGGCTGG
140053 140077 139588 140298 CDS product virion protein US10 140053-140077 5:02:01 GGCCAGGCCTCCGCGGGCGGCCCGG
140096 140109 139588 140298 CDS product virion protein US10 140096-140109 4:01:01 CCGCTGCCTCCGCC
140199 140246 139588 140298 CDS product virion protein US10 140199-140246 10:07:02 CCCGTCCGCGCCCGCCCCCGTCCCCGTCCCCGCTCCGGACTCCTCGCC
140946 140964 140911 141792 CDS product regulatory protein ICP22 140946-140964 4:01:01 GGGGGGTTGGCGCTTGCGG
140987 141022 140911 141792 CDS product regulatory protein ICP22 140987-141022 8:05:02 GGGTTGTAGGGTGGTGGTTGTTGGTGGGGGCATCGG
141080 141093 140911 141792 CDS product regulatory protein ICP22 141080-141093 4:01:01 CCTCCCCCACGTCC
141233 141264 140911 141792 CDS product regulatory protein ICP22 141233-141264 6:03:01 GGGTATTCGGGGGGTCGCTCGGCGGCTCCAGG
141441 141463 140911 141792 CDS product regulatory protein ICP22 141441-141463 5:02:01 GGTCACGTGGATCGAGGAGGTGG
141539 141555 140911 141792 CDS product regulatory protein ICP22 141539-141555 4:01:01 CCCCTGGCCCCCGGGCC
141544 141587 140911 141792 CDS product regulatory protein ICP22 141544-141587 7:04:01 GGCCCCCGGGCCAGGTACGGGCGCAGGGCCCGCGGGGATAGCGG
141592 141625 140911 141792 CDS product regulatory protein ICP22 141592-141625 6:03:01 CCGTATTCCGGATGGTCCGAGCCGCCGGGGAGCC
141656 141675 140911 141792 CDS product regulatory protein ICP22 141656-141675 4:01:01 GGCTGGAGCGTGGGGACGGG
141685 141728 140911 141792 CDS product regulatory protein ICP22 141685-141728 11:08:02 GGGGGCGAGGGGGTCAGGGACGGGATGAGGGGGCTGGTGGACGG
144639 144666 144569 149032 CDS product transcriptional regulator ICP4 144639-144666 5:02:01

CCCCCTCATCCGCGCAGCCAGCGCGGCC

144680 144700 144569 149032 CDS product transcriptional regulator ICP4 144680-144700 5:02:01
CCCAGCCCGCCGCGCCCGACC

144768 144796 144569 149032 CDS product transcriptional regulator ICP4 144768-144796 6:03:01
CCCTCCGCCGTCTCCGACTCCCGAGCCCC

144799 144816 144569 149032 CDS product transcriptional regulator ICP4 144799-144816 4:01:01
GGAGGGAAGGCGAAGCGG

144825 144842 144569 149032 CDS product transcriptional regulator ICP4 144825-144842 4:01:01
GGCCGCCGGCAGCGGCGG

144863 144899 144569 149032 CDS product transcriptional regulator ICP4 144863-144899 7:04:01
CCGCCCAGCCGGCGTCCCCGGCACCCAGCCCGGCTCC

144927 144944 144569 149032 CDS product transcriptional regulator ICP4 144927-144944 4:01:01
CCACTCCTCCGTGGCCCC

144939 144968 144569 149032 CDS product transcriptional regulator ICP4 144939-144968 5:02:01
GGCCCCGGGGAACGGTCGCCGGGCCACGGG

144957 144981 144569 149032 CDS product transcriptional regulator ICP4 144957-144981 4:01:01
CCGGGCCACGGGCTCCTCATCACCC

144982 145006 144569 149032 CDS product transcriptional regulator ICP4 144982-145006 5:02:01
GGCGGTGGGGACGCGGCCGACCCGG

145025 145076 144569 149032 CDS product transcriptional regulator ICP4 145025-145076 9:06:02
CCGAGACCTGCCCGGGGTCCCCGCAGCCCGAGTTTCCATCCTCGGCCTCCCC

145068 145107 144569 149032 CDS product transcriptional regulator ICP4 145068-145107 7:04:01
GGCCTCCCCGGGCGGAGGGTCCCCGGCACCCCGGGTCCGG

145149 145180 144569 149032 CDS product transcriptional regulator ICP4 145149-145180 6:03:01 GGACGAGGACGACCAGGCGGATGGTGCCGGGG
145228 145240 144569 149032 CDS product transcriptional regulator ICP4 145228-145240 4:01:01 GGCGGCGAGGAGG
145271 145314 144569 149032 CDS product transcriptional regulator ICP4 145271-145314 8:05:02 CCGCGAAAACCCCGTCGGCCGCCGGCTCTCCCGGGCCGTCCTCC
145315 145339 144569 149032 CDS product transcriptional regulator ICP4 145315-145339 4:01:01 GGAGGGGATCGCCCGGCCGCTGGGG
145326 145349 144569 149032 CDS product transcriptional regulator ICP4 145326-145349 5:02:01 CCCGGCCGCTGGGGCCGCCACCCC
145359 145400 144569 149032 CDS product transcriptional regulator ICP4 145359-145400 8:05:02 CCGCTCCGGCGCCGCTTCCCCCGGCGCACCCGCTCCGGCTCC
145411 145427 144569 149032 CDS product transcriptional regulator ICP4 145411-145427 4:01:01 CCCGCTCCCAGCCGCCC
145437 145448 144569 149032 CDS product transcriptional regulator ICP4 145437-145448 4:01:01 CCTCCTCCCTCC
145543 145586 144569 149032 CDS product transcriptional regulator ICP4 145543-145586 8:05:02 CCGCCGGGCCGGAGCCCTCACCGGCTCCCCAAGGACCAGCGTCC
145625 145679 144569 149032 CDS product transcriptional regulator ICP4 145625-145679 9:06:02 CCAACCCCAGACCCATACCCCAGACCCAGACCCAGGCACCGGCCGAGGAGGCCCC
145659 145675 144569 149032 CDS product transcriptional regulator ICP4 145659-145675 4:01:01 GGCACCGGCCGAGGAGG
145722 145738 144569 149032 CDS product transcriptional regulator ICP4 145722-145738 4:01:01 GGCTACCGGGGCGGCGG
145752 145765 144569 149032 CDS product transcriptional regulator ICP4 145752-145765 4:01:01 GGCCCCGGCGGCGG

145808 145830 144569 149032 CDS product transcriptional regulator ICP4 145808-145830 4:01:01
CCCCGTCCGGGGACCCCTGGCCC
145816 145837 144569 149032 CDS product transcriptional regulator ICP4 145816-145837 4:01:01
GGGGACCCCTGGCCCGGGTCGG
145867 145900 144569 149032 CDS product transcriptional regulator ICP4 145867-145900 6:03:01
GGGGGGACCGGGGACTCGCGGGACGGGCTGTGGG
146153 146167 144569 149032 CDS product transcriptional regulator ICP4 146153-146167 4:01:01 CCAACCCCCTGCCCC
146173 146193 144569 149032 CDS product transcriptional regulator ICP4 146173-146193 4:01:01
GGGGACGCCATGGCGGCCGGG
146222 146245 144569 149032 CDS product transcriptional regulator ICP4 146222-146245 5:02:01
CCGCCTCGGTGGCCATGAGCCGCC
146274 146295 144569 149032 CDS product transcriptional regulator ICP4 146274-146295 5:02:01
CCTCCAGAGCCTCCGGCGCGCC
146325 146338 144569 149032 CDS product transcriptional regulator ICP4 146325-146338 4:01:01 GGCGGGGAGGCCGG
146348 146395 144569 149032 CDS product transcriptional regulator ICP4 146348-146395 10:07:02
CCGCCGTGGCCGGCTACCCGGCCCAGGCCGCCGCTGCCGCGGCCAGCC
146355 146374 144569 149032 CDS product transcriptional regulator ICP4 146355-146374 4:01:01
GGCCGGCTACCCGGCCCAGG
146404 146434 144569 149032 CDS product transcriptional regulator ICP4 146404-146434 5:02:01
CCCGAGGCCCCGGCGCCCTCGGTCCGCGTCC
146481 146523 144569 149032 CDS product transcriptional regulator ICP4 146481-146523 6:03:01
GGCTGCCGCGGCCGCGGCCGCTCCGGGGACCAGGGCGCCCAGG
146486 146532 144569 149032 CDS product transcriptional regulator ICP4 146486-146532 9:06:02

CCGCGGCCGCGGCCGCTCCGGGGACCAGGGCGCCCAGGCCGTCCGCC

146591 146605 144569 149032 CDS product transcriptional regulator ICP4 146591-146605 4:01:01 CCCTCCTGCGCCTCC

146649 146670 144569 149032 CDS product transcriptional regulator ICP4 146649-146670 5:02:01
CCCCTCTCCCGCCTGCCCTCCC

146691 146710 144569 149032 CDS product transcriptional regulator ICP4 146691-146710 5:02:01
GGCCGGGCTGGAGACGGCGG

146732 146764 144569 149032 CDS product transcriptional regulator ICP4 146732-146764 5:02:01
GGGACCTGGTCGAGCGGGCCAGGCTCCTCGGGG

146772 146793 144569 149032 CDS product transcriptional regulator ICP4 146772-146793 5:02:01
CCCTACCCACCGCCTGGGCTCC

146826 146854 144569 149032 CDS product transcriptional regulator ICP4 146826-146854 6:03:01
GGTGGTGGCCCGGACCGTGGCGCCGCTGG

146867 146913 144569 149032 CDS product transcriptional regulator ICP4 146867-146913 8:05:02
CCGAGGGGGCCCGGGCCCGGGCCTCGGCCTGGACCGTCACCCAGGCC

146871 146898 144569 149032 CDS product transcriptional regulator ICP4 146871-146898 6:03:01
GGGGGCCCGGGCCCGGGCCTCGGCCTGG

146934 146956 144569 149032 CDS product transcriptional regulator ICP4 146934-146956 6:03:01
GGTCGGGGGGATGTTGGGGGAGG

146957 146978 144569 149032 CDS product transcriptional regulator ICP4 146957-146978 4:01:01
CCGTGTCCCTGCTGGCCCCACC

146987 147012 144569 149032 CDS product transcriptional regulator ICP4 146987-147012 4:01:01
CCCAGCAGCCCTCATCGTCCTCGCCC

147022 147042 144569 149032 CDS product transcriptional regulator ICP4 147022-147042 4:01:01

CCCTTCTCCGGCTCCGCGGCC
147031 147051 144569 149032 CDS product transcriptional regulator ICP4 147031-147051 4:01:01 GGCTCCGCGGCCGCGGAGGGG
147054 147090 144569 149032 CDS product transcriptional regulator ICP4 147054-147090 7:04:01 CCTTCAGACCCTGCCGCCCCTGTGGCCCACCGTCCCC
147111 147145 144569 149032 CDS product transcriptional regulator ICP4 147111-147145 6:03:01 CCCCTCGTCCCACTCCCAGTCCCCCCAGCACTCCC
147151 147166 144569 149032 CDS product transcriptional regulator ICP4 147151-147166 4:01:01 GGCGGAGGCGCCGGGG
147173 147196 144569 149032 CDS product transcriptional regulator ICP4 147173-147196 6:03:01 CCGCCACCTGCTGCCGGGCCACCC
147260 147283 144569 149032 CDS product transcriptional regulator ICP4 147260-147283 6:03:01 CCGCCTCCCCGGCCCACCTCAGCC
147315 147336 144569 149032 CDS product transcriptional regulator ICP4 147315-147336 4:01:01 CCGTGCCATCCACGGGCGCCCC
147328 147357 144569 149032 CDS product transcriptional regulator ICP4 147328-147357 4:01:01 GGGCGCCCCAGGGGCAAGAGCGGCAAGCGG
147362 147421 144569 149032 CDS product transcriptional regulator ICP4 147362-147421 11:08:02 CCGAGCCCCTGGAGCCGGCGGCCCAGGCCGGAGCCTCGGCCTCCTTCTCCTCGTCCGCCC
147372 147400 144569 149032 CDS product transcriptional regulator ICP4 147372-147400 6:03:01 GGAGCCGGCGGCCCAGGCCGGAGCCTCGG
147453 147465 144569 149032 CDS product transcriptional regulator ICP4 147453-147465 4:01:01 CCCTCCGGCCCCC
147473 147512 144569 149032 CDS product transcriptional regulator ICP4 147473-147512 6:03:01 GGGTGGCCACCCCGGGCCACCAGGCTCCCCGGGCCCTGGG

147479 147538 144569 149032 CDS product transcriptional regulator ICP4 147479-147538 11:08:02
CCACCCCGGGCCACCAGGCTCCCCGGGCCCTGGGACCCATGCCAGCCGAGGGCCCCGACC
147651 147677 144569 149032 CDS product transcriptional regulator ICP4 147651-147677 4:01:01
CCAGCTGTTCCCCGAGGCCTGGCGCCC
147686 147721 144569 149032 CDS product transcriptional regulator ICP4 147686-147721 6:03:01
CCTTCGATCCCCAGGCCCTGGCCACCATCGCGGCCC
147730 147749 144569 149032 CDS product transcriptional regulator ICP4 147730-147749 4:01:01
GGCCCCCCGGCCCGGGACGG
147760 147779 144569 149032 CDS product transcriptional regulator ICP4 147760-147779 4:01:01
GGGGAGCTGGCGGCCAGCGG
147828 147844 144569 149032 CDS product transcriptional regulator ICP4 147828-147844 4:01:01
GGACGTGAAGGTGGTGG
147864 147892 144569 149032 CDS product transcriptional regulator ICP4 147864-147892 5:02:01
GGACGAGGACCTGCTGGGCGGACTCCCGG
147888 147903 144569 149032 CDS product transcriptional regulator ICP4 147888-147903 4:01:01 CCCGGCCTCCCGCCCC
147942 147975 144569 149032 CDS product transcriptional regulator ICP4 147942-147975 6:03:01
GGGGGGACTCTCGGCGCTGCTGGCGGCCCTGGGG
147968 148011 144569 149032 CDS product transcriptional regulator ICP4 147968-148011 7:04:01
CCCTGGGGAACCGCATCCTCACCAAGCGGTCCCACGCCTGGGCC
148007 148042 144569 149032 CDS product transcriptional regulator ICP4 148007-148042 6:03:01
GGGCCGGCAACTGGACCGGGGCCCCGGACGTCTCGG
148076 148095 144569 149032 CDS product transcriptional regulator ICP4 148076-148095 4:01:01
CCGGGGACCTGGCCTTCACC

148110 148134 144569 149032 CDS product transcriptional regulator ICP4 148110-148134 5:02:01
CCTCTGCCTGCGCCTGGGCTCCGCC
148173 148190 144569 149032 CDS product transcriptional regulator ICP4 148173-148190 4:01:01
GGATTGGCCCCAGGACGG
148264 148309 144569 149032 CDS product transcriptional regulator ICP4 148264-148309 8:05:02
CCCCGGGAGCGCCACCTCAGCCGCGCGGTCCTCACCTCCAGCACCC
148389 148407 144569 149032 CDS product transcriptional regulator ICP4 148389-148407 4:01:01
CCGCTCCTCCAACGTGGCC
148467 148485 144569 149032 CDS product transcriptional regulator ICP4 148467-148485 4:01:01
CCGCCAGCGCGTCCTGCCC
148495 148533 144569 149032 CDS product transcriptional regulator ICP4 148495-148533 6:03:01
GGCTGCAAGGACATGCGGGCCCAGGCCGAGGGCCTCGGG
148514 148544 144569 149032 CDS product transcriptional regulator ICP4 148514-148544 5:02:01
CCCAGGCCGAGGGCCTCGGGTTCCACGACCC
148572 148588 144569 149032 CDS product transcriptional regulator ICP4 148572-148588 4:01:01
CCACCGCGCGGCCAACC
148614 148636 144569 149032 CDS product transcriptional regulator ICP4 148614-148636 4:01:01
CCCCGTGTACCTCGCCTGCGGCC
148633 148666 144569 149032 CDS product transcriptional regulator ICP4 148633-148666 6:03:01
GGCCGGCGCGGCGCTGGGGCCGTGGAGCCCTCGG
148720 148741 144569 149032 CDS product transcriptional regulator ICP4 148720-148741 4:01:01
CCCGACGCCGAGGCCGAGCCCC
148750 148781 144569 149032 CDS product transcriptional regulator ICP4 148750-148781 6:03:01

CCCATCACCGAGGCTCCCCGCCGCCGAGCCCC

148784 148808 144569 149032 CDS product transcriptional regulator ICP4 148784-148808 4:01:01 GGGTCGACTGGGAGCCCGGGTTCGG

148888 148912 144569 149032 CDS product transcriptional regulator ICP4 148888-148912 5:02:01 GGGGCCGGCGATGTGGAGCTGGTGG

148920 148965 144569 149032 CDS product transcriptional regulator ICP4 148920-148965 8:05:02 CCCCGGCCCGAGCCCCGGCGTGGCCCAGGCCCTCCGCCGCGCTCCC

148977 149010 144569 149032 CDS product transcriptional regulator ICP4 148977-149010 7:04:01 GGTGGTGTCGGACGACGAGGACGGAGGAGACTGG

gi|9629732|ref|NC_001844.1| Equid herpesvirus 4, complete genome

2928 2954 2457 3227 CDS product myristylated tegument protein CIRC 2928-2954 6:03:01 CCACCAACCACGCTTCCACCGAAAACC

3145 3174 2457 3227 CDS product myristylated tegument protein CIRC 3145-3174 5:02:01 CCCACCCAGCGAGCCAAACTGCCTTACACC

3672 3688 3262 3864 CDS product nuclear protein UL55 3672-3688 5:02:01 GGCGGGTTGGGAGGTGG

4268 4303 4081 5484 CDS product multifunctional expression regulator 4268-4303 6:03:01 CCGGAGACACCGCCTCGGCGCCACTCCCAACGGCCC

4589 4620 4081 5484 CDS product multifunctional expression regulator 4589-4620 5:02:01 CCAAGTCCCGTAGGGACCGCGCCGCTTCTGCC

4863 4879 4081 5484 CDS product multifunctional expression regulator 4863-4879 4:01:01 GGCGTTGGCGGCTGCGG

4992 5009 4081 5484 CDS product multifunctional expression regulator 4992-5009 4:01:01

CCACCCGCCAGACGGTCC
5785 5797 5618 6649 CDS product envelope glycoprotein K 5785-5797 4:01:01 GGGGGCTGGGGGG
5902 5936 5618 6649 CDS product envelope glycoprotein K 5902-5936 5:02:01
GGGGTATAGCTGGGAGCAGGGAGTATGGACTAAGG
7276 7302 6658 9900 CDS product helicase-primase primase subunit 7276-7302 5:02:01
CCGCCATAAACCTCCCCAGAGTCGCCC
7457 7470 6658 9900 CDS product helicase-primase primase subunit 7457-7470 4:01:01 CCTCCGGTCCCGCC
7480 7493 6658 9900 CDS product helicase-primase primase subunit 7480-7493 4:01:01 GGTGGTGGTACCGG
8437 8458 6658 9900 CDS product helicase-primase primase subunit 8437-8458 4:01:01
GGTTTGGTAGATGGAATTAAGG
8466 8481 6658 9900 CDS product helicase-primase primase subunit 8466-8481 4:01:01 CCAGCCATGTCCAGCC
9096 9115 6658 9900 CDS product helicase-primase primase subunit 9096-9115 4:01:01
GGTGGCTTGTAGGTCGTAGG
9987 10018 9899 10633 CDS product tegument protein UL51 9987-10018 7:04:01
CCCAGCCACCATGCTCCGCCTACAGTCCGCCC
10728 10748 10722 11702 CDS product deoxyuridine triphosphatase 10728-10748 4:01:01 CCTGTTGAACCAAAGCCCCCC
10835 10860 10722 11702 CDS product deoxyuridine triphosphatase 10835-10860 6:03:01
GGTGGGCGAATAGGTAGGAAAGGGGG
11912 11931 11671 11973 CDS product envelope glycoprotein N 11912-11931 4:01:01 CCAGGCATACCGCGCCTTCC
12464 12506 12128 13042 CDS product tegument protein VP22 12464-12506 7:04:01
CCTCCCATGGCCGTTTGAGCCCCACAAAAACCACACCCCACCC
12671 12692 12128 13042 CDS product tegument protein VP22 12671-12692 4:01:01 CCCCCAAAACGCCAAAGACGCC
13543 13558 13173 14519 CDS product transactivating tegument protein VP16 13543-13558 4:01:01

CCTGCCCATACCCACC
14338 14389 13173 14519 CDS product transactivating tegument protein VP16 14338-14389 10:07:02
CCCACCATCCCCCAGCGCCGTTTTACCAGGGGATCCAGTTCCACCGCTTACC
15166 15187 14890 17484 CDS product tegument protein VP13/14 15166-15187 4:01:01 CCCCCGCGCAGCCACAAGCACC
15800 15820 14890 17484 CDS product tegument protein VP13/14 15800-15820 4:01:01 CCACCCGGTTGCCATGAACCC
16400 16414 14890 17484 CDS product tegument protein VP13/14 16400-16414 4:01:01 CCAACCGCTTCCCCC
16649 16662 14890 17484 CDS product tegument protein VP13/14 16649-16662 4:01:01 GGTGGCAATGGAGG
16961 16972 14890 17484 CDS product tegument protein VP13/14 16961-16972 4:01:01 GGTGGATGGGGG
17226 17242 14890 17484 CDS product tegument protein VP13/14 17226-17242 4:01:01 CCACGCCCTGGACCCCC
17328 17358 14890 17484 CDS product tegument protein VP13/14 17328-17358 6:03:01
GGCGCCGGTTTGGGGCGCATGGTGGACATGG
17651 17677 17633 19864 CDS product tegument protein VP11/12 17651-17677 4:01:01
CCTCATGGGCCCGCGTTTCCAAAAACC
18290 18310 17633 19864 CDS product tegument protein VP11/12 18290-18310 4:01:01 GGTGGCTATTGTGGTTTATGG
18647 18664 17633 19864 CDS product tegument protein VP11/12 18647-18664 5:02:01 GGTTGGACGGCGGCGTGG
18755 18786 17633 19864 CDS product tegument protein VP11/12 18755-18786 5:02:01
GGGCAAACATGGAAAAGGGAACGGTTATGTGG
18945 18974 17633 19864 CDS product tegument protein VP11/12 18945-18974 6:03:01
CCCGCCCCAGGGACCTCTTCCTGCGCCACC
19268 19287 17633 19864 CDS product tegument protein VP11/12 19268-19287 5:02:01 CCTCCACGCCTTCGCCAACC
19422 19444 17633 19864 CDS product tegument protein VP11/12 19422-19444 4:01:01 GGAGGAAATTTGGCAATTGCGGG
20087 20105 20018 20698 CDS product membrane protein UL45 20087-20105 4:01:01 GGGCTGAGTGGGCGGGCGG
20554 20583 20018 20698 CDS product membrane protein UL45 20554-20583 6:03:01

GGTTGGTTGGGGTAGAGTCGGCAGCGGTGG
21447 21459 20939 22396 CDS product envelope glycoprotein C 21447-21459 4:01:01 GGAGGCTGGGGGG
21662 21687 20939 22396 CDS product envelope glycoprotein C 21662-21687 4:01:01 GGGTGAACGGAGAGGTCCATTAGGGG
22282 22301 20939 22396 CDS product envelope glycoprotein C 22282-22301 5:02:01 GGTTGGTGTGGCTGGACTGG
22648 22668 22575 23786 CDS product envelope protein UL43 22648-22668 4:01:01 GGGTTGCGGCCAAACGGCTGG
24016 24041 24002 25222 CDS product DNA polymerase processivity subunit 24016-24041 4:01:01
GGGAGCTTCGGGACAGGGAATTTTGG
24522 24537 24002 25222 CDS product DNA polymerase processivity subunit 24522-24537 4:01:01 CCACCTGCCTATCTCC
24853 24874 24002 25222 CDS product DNA polymerase processivity subunit 24853-24874 5:02:01
GGTTGGCTGGGCCTTGGTCCGG
24882 24904 24002 25222 CDS product DNA polymerase processivity subunit 24882-24904 4:01:01
CCAATAAACCCCTGCGTCCGTCC
26009 26033 25765 27255 CDS product tegument host shutoff protein 26009-26033 4:01:01
GGACAGAGGTATATACGGTGATGGG
26164 26185 25765 27255 CDS product tegument host shutoff protein 26164-26185 5:02:01
CCAACGGTTCCACCACCCAGCC
26469 26487 25765 27255 CDS product tegument host shutoff protein 26469-26487 4:01:01 CCATTGTTTCCCCCTACCC
27173 27188 25765 27255 CDS product tegument host shutoff protein 27173-27188 4:01:01 CCACATTCCCACCCCC
27447 27471 27362 28324 CDS product ribonucleotide reductase subunit 2 27447-27471 6:03:01
GGGGGAAGCTTGGTGGGGGTTTGGG
27494 27517 27362 28324 CDS product ribonucleotide reductase subunit 2 27494-27517 5:02:01
CCAATTTCCCCCAAAAGCCTGTCC
27561 27584 27362 28324 CDS product ribonucleotide reductase subunit 2 27561-27584 5:02:01

GGTGGGTGCTTTTGGGGGCATGGG

29166 29191 28363 30732 CDS product ribonucleotide reductase subunit 1 29166-29191 4:01:01 CCCCTGAATTCCAAGTCCCAGCGACC

29329 29341 28363 30732 CDS product ribonucleotide reductase subunit 1 29329-29341 4:01:01 GGAGGTGGAAGGG

29574 29592 28363 30732 CDS product ribonucleotide reductase subunit 1 29574-29592 4:01:01 CCCAAATCCACACCGCTCC

29677 29698 28363 30732 CDS product ribonucleotide reductase subunit 1 29677-29698 4:01:01 CCGCTTTCTCCATCCACGTACC

29998 30018 28363 30732 CDS product ribonucleotide reductase subunit 1 29998-30018 4:01:01 CCAACCTCTTCCATAAGAGCC

30171 30192 28363 30732 CDS product ribonucleotide reductase subunit 1 30171-30192 5:02:01 CCAGCCAACCTCACCGCTAACC

31058 31084 30967 32355 CDS product capsid triplex subunit 1 31058-31084 5:02:01 GGCGAATGGTTCTGGGGGAGTCCGAGG

31203 31234 30967 32355 CDS product capsid triplex subunit 1 31203-31234 6:03:01 GGGTGGGCATCTAGGCTCGCACGGCCGGTTGG

31264 31294 30967 32355 CDS product capsid triplex subunit 1 31264-31294 5:02:01 GGGTGTGGATCTGGTGCGTCGGCTCCCTCGG

31944 31965 30967 32355 CDS product capsid triplex subunit 1 31944-31965 4:01:01 GGCATCAAGGTGGTTTCGTAGG

32860 32882 32712 35777 CDS product tegument protein UL37 32860-32882 4:01:01 GGTATCTGTGGAAGAGGCAGCGG

33003 33029 32712 35777 CDS product tegument protein UL37 33003-33029 4:01:01 GGACCGACCGGGAAACGGAAGCCGTGG

33625 33641 32712 35777 CDS product tegument protein UL37 33625-33641 4:01:01 GGCGGGAATGGTAGCGG

34127 34148 32712 35777 CDS product tegument protein UL37 34127-34148 4:01:01 GGTTTGGCAGACAAGGGCGTGG

34799 34828 32712 35777 CDS product tegument protein UL37 34799-34828 5:02:01
CCTTTACCGGAACGGCCAACCGTTAGAACC
35150 35174 32712 35777 CDS product tegument protein UL37 35150-35174 4:01:01 GGAGACATGGCACTGGCCACAGAGG
35311 35331 32712 35777 CDS product tegument protein UL37 35311-35331 4:01:01 GGGGGGCTCCGAGGCACCCGG
36019 36047 36006 46610 CDS product large tegument protein 36019-36047 5:02:01
CCCACCTGTTCCAACGGCCGGTGGGGCCC
36034 36069 36006 46610 CDS product large tegument protein 36034-36069 7:04:01
GGCCGGTGGGGCCCAGGCTGATGTGGTGGTTATAGG
36974 36994 36006 46610 CDS product large tegument protein 36974-36994 5:02:01 CCCCCGTGGACTCCCCCCACC
37116 37140 36006 46610 CDS product large tegument protein 37116-37140 5:02:01 GGGCCGAGGTATTGGATGATGGGGG
37260 37281 36006 46610 CDS product large tegument protein 37260-37281 4:01:01 GGAGGATTTTCAGGATTTCTGG
37318 37343 36006 46610 CDS product large tegument protein 37318-37343 5:02:01 GGGGGTCCTAGGCGTGGAGATAGAGG
37483 37503 36006 46610 CDS product large tegument protein 37483-37503 4:01:01 GGCGTTTTTGGTGGAAAATGG
37571 37585 36006 46610 CDS product large tegument protein 37571-37585 4:01:01 CCAGACCAATCCGCC
38104 38123 36006 46610 CDS product large tegument protein 38104-38123 4:01:01 CCTTGCCTCAGTCCCTAACC
38899 38930 36006 46610 CDS product large tegument protein 38899-38930 5:02:01
GGCTCCTGGAGCGTTGGAACAGCTGGCGAAGG
39082 39098 36006 46610 CDS product large tegument protein 39082-39098 4:01:01 GGCTGGCGTAGGACCGG
40299 40313 36006 46610 CDS product large tegument protein 40299-40313 4:01:01 GGGCTTTGGAGGAGG
40759 40780 36006 46610 CDS product large tegument protein 40759-40780 4:01:01 GGACGTTAAGGCCGTGGAGTGG
40933 40956 36006 46610 CDS product large tegument protein 40933-40956 4:01:01 GGTTAGCTGGGATGAGGCATGGGG
41376 41416 36006 46610 CDS product large tegument protein 41376-41416 8:05:02
CCGGAATAGCCGCTGCCGGTAGCCGCCCCCACCTGGAAGCC

41649 41676 36006 46610 CDS product large tegument protein 41649-41676 4:01:01 CCATGCGCCCCGCTATGACCCGCGAGCC
42309 42338 36006 46610 CDS product large tegument protein 42309-42338 5:02:01
GGATGCGGTCCCCTGGTGAGAACGGGCGGG
42819 42841 36006 46610 CDS product large tegument protein 42819-42841 5:02:01 CCGTTTCCTCCGACCTGAGCTCC
42907 42929 36006 46610 CDS product large tegument protein 42907-42929 5:02:01 GGAACGGCTGTCTGGGTTGGTGG
43378 43404 36006 46610 CDS product large tegument protein 43378-43404 5:02:01 GGCGTGGCAAGAGTGGCTAGAGGATGG
43505 43522 36006 46610 CDS product large tegument protein 43505-43522 4:01:01 CCTCCTCTATTGCCACCC
43646 43662 36006 46610 CDS product large tegument protein 43646-43662 4:01:01 CCAACACCTCCCCTCCC
43799 43819 36006 46610 CDS product large tegument protein 43799-43819 4:01:01 CCCCAAACCCCTGCACCCCCC
44123 44136 36006 46610 CDS product large tegument protein 44123-44136 4:01:01 CCACCTCCTGATCC
44154 44738 36006 46610 CDS product large tegument protein 44154-44738 88:85:22
CCGCTCCGTCCAAACCCGCAGCGGCCCCGGCTCCGTCCAAACCCGCAGCGGCCCCGGCTCCGTCCAAACCCGCAGCGGCCCCGGCTCCGTCCAAACCCGCAGCGGCCCCGGCTCCGTCCAAACCCGCA
GCGGCCCCGGCTCCGTCCAAACCCGCAGCGGCCCCGGCTCCGTCCAAACCCGCAGCGGCCCCGGCTCCGTCCAAACCCGCAGCGGCCCCGGCTCCGTCCAAACCCGCAGCGGCCCCGGCTCCGTCCAAACCC
GCAGCGGCCCCGGCTCCGTCCAAACCCGCAGCGGCCCCGGCTCCGTCCAAACCCGCAGCGGCCCCGGCTCCGTCCAAACCCGCAGCGGCCCCGGCTCCGTCCAAACCCGCAGCGGCCCCGGCTCCGTCCAAA
CCCGCAGCGGCCCCGGCTCCGTCCAAACCCGCAGCGGCCCCGGCTCCGTCCAAACCCGCAGCGGCCCCGGCTCCGTCCAAACCCGCAGCGGCCCCGGCTCCGTCCAAACCCGCAGCGGCCCCGGCTCCGTCC
AAACCCGCAGCGGCCCCGGCTCCGTCCAAACCCGCAGCGGCCCCGGCTCCGTCCAAACCCC
44972 44992 36006 46610 CDS product large tegument protein 44972-44992 4:01:01 CCCTTACCAAATACCTCTCCC
45261 45288 36006 46610 CDS product large tegument protein 45261-45288 5:02:01 CCGCCAACGTACCTCTCCCGGACTCACC
45323 45343 36006 46610 CDS product large tegument protein 45323-45343 4:01:01 CCATTGCCCGATTCACCCACC
45852 45873 36006 46610 CDS product large tegument protein 45852-45873 4:01:01 CCAAACCGCTACCAAAGTCTCC
47665 47685 47156 47980 CDS product nuclear egress membrane protein 47665-47685 4:01:01
GGCCATTGGGGCGTTTGGTGG
48562 48579 48515 50365 CDS product DNA packaging protein UL32 48562-48579 4:01:01 GGTATAACAGGAGGCTGG

48691 48713 48515 50365 CDS product DNA packaging protein UL32 48691-48713 4:01:01 CCCACGCCCAAACCTCAACACCC
49099 49117 48515 50365 CDS product DNA packaging protein UL32 49099-49117 4:01:01 GGAGTTGGATCTGGGACGG
49131 49156 48515 50365 CDS product DNA packaging protein UL32 49131-49156 4:01:01 GGCGTTAACGGGGCCTGGTATGATGG
49993 50014 48515 50365 CDS product DNA packaging protein UL32 49993-50014 4:01:01 GGAGACGCGGGTAGGTTTATGG
50119 50141 48515 50365 CDS product DNA packaging protein UL32 50119-50141 4:01:01 CCACTGGGGCCTCCCCAGGAACC
51950 51960 51262 54924 CDS product DNA polymerase catalytic subunit 51950-51960 4:01:01 CCTCCGCCGCC
52441 52463 51262 54924 CDS product DNA polymerase catalytic subunit 52441-52463 4:01:01 CCGTTTGCCACACCGGTGAATCC
52817 52875 51262 54924 CDS product DNA polymerase catalytic subunit 52817-52875 10:07:02 CCTTTGCTCCTTGGTATCCCACAATCCTACCTGTGCCGGGTTTTCCGCCCCCTGTTTCC
53970 53988 51262 54924 CDS product DNA polymerase catalytic subunit 53970-53988 5:02:01 GGTTCCGGGCCGGAGGCGG
54217 54245 51262 54924 CDS product DNA polymerase catalytic subunit 54217-54245 7:04:01 CCTCCCCACCAGCCGCCGCTGTTTCCACC
54589 54623 51262 54924 CDS product DNA polymerase catalytic subunit 54589-54623 6:03:01 CCGTTCCAAACCCGAATCCTGCGAGGCCAGCAACC
55390 55407 55178 58804 CDS product single-stranded DNA-binding protein 55390-55407 4:01:01 GGCCTTGGCGGCGCTGGG
55521 55538 55178 58804 CDS product single-stranded DNA-binding protein 55521-55538 4:01:01 GGCAAGACGGAGGTTTGG
55546 55562 55178 58804 CDS product single-stranded DNA-binding protein 55546-55562 4:01:01 CCATTTTCCTCCCCACC

56040 56064 55178 58804 CDS product single-stranded DNA-binding protein 56040-56064 4:01:01
GGGAACTTCTGGTAAAGGTTCTGGG
56079 56094 55178 58804 CDS product single-stranded DNA-binding protein 56079-56094 4:01:01 GGGGGACGGTAGCGGG
56296 56314 55178 58804 CDS product single-stranded DNA-binding protein 56296-56314 4:01:01
GGCCTGGTTGGCGCCATGG
56387 56413 55178 58804 CDS product single-stranded DNA-binding protein 56387-56413 5:02:01
CCAATCCTTCTTACCACCGCTTTTACC
56480 56502 55178 58804 CDS product single-stranded DNA-binding protein 56480-56502 4:01:01
CCGCAGACCAGCCAGCTGCTCCC
56987 57006 55178 58804 CDS product single-stranded DNA-binding protein 56987-57006 4:01:01
CCGGCACCTGCTCGGCCTCC
57280 57297 55178 58804 CDS product single-stranded DNA-binding protein 57280-57297 4:01:01
GGTCAGTCGGTGGAGGGG
57466 57493 55178 58804 CDS product single-stranded DNA-binding protein 57466-57493 5:02:01
GGGGACATGGCTAGGGTTAGCATGGAGG
57631 57649 55178 58804 CDS product single-stranded DNA-binding protein 57631-57649 4:01:01
GGTGCGGTTGGCTTTTTGG
57704 57715 55178 58804 CDS product single-stranded DNA-binding protein 57704-57715 4:01:01 CCCCCAACCCCC
57956 57978 55178 58804 CDS product single-stranded DNA-binding protein 57956-57978 4:01:01
CCAGACGACCTCGTGATCCGGCC
57975 57993 55178 58804 CDS product single-stranded DNA-binding protein 57975-57993 4:01:01
GGCCGCGGTATTGGCCTGG
58200 58229 55178 58804 CDS product single-stranded DNA-binding protein 58200-58229 6:03:01

GGCAGGCAACTGGAATGGCCTCAACGGCGG

58311 58333 55178 58804 CDS product single-stranded DNA-binding protein 58311-58333 6:03:01 GGCTGGCGGATTTGGTATGGGGG

58540 58567 55178 58804 CDS product single-stranded DNA-binding protein 58540-58567 5:02:01 GGGGAGTGGAGCGTGGAGGCCGCCCAGG

60624 60641 58964 61285 CDS product DNA packaging terminase subunit 2 60624-60641 4:01:01 GGCGGTGAGTGGCGCCGG

60887 60918 58964 61285 CDS product DNA packaging terminase subunit 2 60887-60918 5:02:01 GGTTTATGGTTACTGCGGCTAAAGGTGATTGG

61299 61313 61147 64074 CDS product envelope glycoprotein B 61299-61313 4:01:01 CCAGCTACCTCCCCC

61422 61464 61147 64074 CDS product envelope glycoprotein B 61422-61464 6:03:01 CCCAGTACTCCCGCGTCAACCCAGTCCGCTAAAACCGTTGACC

61552 61564 61147 64074 CDS product envelope glycoprotein B 61552-61564 4:01:01 CCTGCCCGCCGCC

61980 61997 61147 64074 CDS product envelope glycoprotein B 61980-61997 4:01:01 GGATGGATGCCATGGAGG

62709 62739 61147 64074 CDS product envelope glycoprotein B 62709-62739 6:03:01 CCAGAACCTACTCCAACCCAAGAGAGCCTCC

63576 63605 61147 64074 CDS product envelope glycoprotein B 63576-63605 6:03:01 GGCCTTGGTAAGGTGGGAGAGGCAGTTGGG

63618 63633 61147 64074 CDS product envelope glycoprotein B 63618-63633 4:01:01 GGAGCGGCTGGCGCGG

63696 63723 61147 64074 CDS product envelope glycoprotein B 63696-63723 4:01:01 GGCCTGTTGGTAATTGCGGGCTTAGTGG

64317 64339 64268 64750 CDS product protein V32 64317-64339 4:01:01 GGCTGCATCGGCTACCGCGGCGG

64607 64626 64268 64750 CDS product protein V32 64607-64626 4:01:01 CCAGGGACCCCCATGTGTCC

64677 64697 64268 64750 CDS product protein V32 64677-64697 4:01:01 CCCAGACACACCCACCGCCCC

65060 65076 64827 65822 CDS product capsid scaffold protein 65060-65076 4:01:01 GGCGGGCACCGGTGTGG
65182 65203 64827 65822 CDS product capsid scaffold protein 65182-65203 4:01:01 GGGGAACGCCCGGTGGGACGGG
65970 65995 64827 66770 CDS product capsid maturation protease 65970-65995 5:02:01
CCGCAGGTGCCAACCAGCTCCGCCCC
66063 66084 64827 66770 CDS product capsid maturation protease 66063-66084 4:01:01 CCCTCTATGCCGGCCTCGCGCC
66489 66515 64827 66770 CDS product capsid maturation protease 66489-66515 5:02:01
CCTGCCGCAGTTGCCAAAACCGCACCC
66580 66598 64827 66770 CDS product capsid maturation protease 66580-66598 4:01:01 CCTCCCCTACCACACACCC
67734 67744 66885 68648 CDS product DNA packaging tegument protein UL25 67734-67744 4:01:01 CCTCCTCCCCC
67795 67817 66885 68648 CDS product DNA packaging tegument protein UL25 67795-67817 5:02:01
CCACCTTCCCGATGGCTCCATCC
67949 67964 66885 68648 CDS product DNA packaging tegument protein UL25 67949-67964 4:01:01 GGCGGAATATGGACGG
68101 68141 66885 68648 CDS product DNA packaging tegument protein UL25 68101-68141 7:04:01
CCCTGTACCAGGTGCCAAAAACCACTCCGTTTGACCCTCCC
68356 68378 66885 68648 CDS product DNA packaging tegument protein UL25 68356-68378 4:01:01
CCACTTTAGCCACTTGCCGCTCC
69157 69172 68749 69567 CDS product nuclear protein UL24 69157-69172 4:01:01 GGAGGCAAGGTTTTGG
69883 69917 69582 70640 CDS product thymidine kinase 69883-69917 6:03:01
CCAAAGCCGCTTTACCACGCCCTACCTGATACTCC
69994 70015 69582 70640 CDS product thymidine kinase 69994-70015 5:02:01 CCGCCACCCGGTCGCCTCTACC
70262 70287 69582 70640 CDS product thymidine kinase 70262-70287 4:01:01 GGGCGAGTTTGGCGCGACGGTTGGGG
71166 71183 70858 73425 CDS product envelope glycoprotein H 71166-71183 4:01:01 CCTCCCAAACACCAACCC
71199 71224 70858 73425 CDS product envelope glycoprotein H 71199-71224 4:01:01 CCAGAACCCGGTGACCCACGCGGACC

71370 71388 70858 73425 CDS product envelope glycoprotein H
71717 71728 70858 73425 CDS product envelope glycoprotein H
72685 72710 70858 73425 CDS product envelope glycoprotein H
72723 72739 70858 73425 CDS product envelope glycoprotein H
74379 74398 74243 75832 CDS product tegument protein UL21
74421 74436 74243 75832 CDS product tegument protein UL21
74680 74712 74243 75832 CDS product tegument protein UL21
GGCATCGGCCACCAGGTGGGGGTCTGGTAACGG
74946 74971 74243 75832 CDS product tegument protein UL21
75224 75241 74243 75832 CDS product tegument protein UL21
75518 75550 74243 75832 CDS product tegument protein UL21
CCAAAAGCCTGGGTACCCTGGACCAATACTTCC
77494 77514 77301 81428 CDS product major capsid protein
77707 77727 77301 81428 CDS product major capsid protein
78181 78206 77301 81428 CDS product major capsid protein
78569 78613 77301 81428 CDS product major capsid protein
CCAACCCCAGGACAGCCAGATCCCCGAACCTACCCACCCCAGACC
78715 78731 77301 81428 CDS product major capsid protein
79120 79143 77301 81428 CDS product major capsid protein
79430 79452 77301 81428 CDS product major capsid protein
79554 79580 77301 81428 CDS product major capsid protein
79860 79904 77301 81428 CDS product major capsid protein
CCCCCACCAGCCCCAGCGACCCGCGCCACCCGCTCAATCCGCGCC

71370-71388 4:01:01 GGCAACCCGGTTGGAGTGG
71717-71728 4:01:01 CCGCCTTCCCCC
72685-72710 5:02:01 CCGTTATCCCACCTCGCCTGTACACC
72723-72739 4:01:01 GGCTTGGAGGACGACGG
74379-74398 4:01:01 CCACCGGCAAGCCGCTTCCC
74421-74436 4:01:01 CCGCCCCCCCGATACC
74680-74712 7:04:01
74946-74971 5:02:01 CCAAGCCCGGCCATGGTTCCTCAACC
75224-75241 4:01:01 GGGGGCCTGGATACGGGG
75518-75550 5:02:01
77494-77514 4:01:01 GGTGCGGTTTTTGGAGCTCGG
77707-77727 4:01:01 GGCTTTGGGTCTGATAGGAGG
78181-78206 5:02:01 GGAGGATATGGCCGCGGACGTTCCGG
78569-78613 8:05:02
78715-78731 4:01:01 GGTGGCCATCAGGAGGG
79120-79143 5:02:01 GGCTACGGTTTTGGCGGTACGTGG
79430-79452 4:01:01 GGGGAAGCAGTGGGTGGACAGGG
79554-79580 5:02:01 GGGCACAGGATCTATGGGTGGATGGGG
79860-79904 9:06:02

80754 80787 77301 81428 CDS product major capsid protein 80754-80787 6:03:01 CCGGAAACCGCCTAGGACCCCAAGGTCCCCGCCC

80996 81027 77301 81428 CDS product major capsid protein 80996-81027 5:02:01 CCCGCTTACCCCAACCGTGCTACCGTTAACCC

81696 81716 81661 82605 CDS product capsid triplex subunit 2 81696-81716 4:01:01 CCAGGAGACCTGTCTCCTTCC

82235 82260 81661 82605 CDS product capsid triplex subunit 2 82235-82260 5:02:01 GGTCAACGGAGTGGCGAGGTCGCTGG

82397 82429 81661 82605 CDS product capsid triplex subunit 2 82397-82429 6:03:01 CCAGCTTATCCACCAGCAGCCCGTACCCCAGCC

82732 82747 82703 88469 CDS product DNA packaging terminase subunit 1 82732-82747 5:02:01 GGTGGTAGGTGGAAGG

83331 83350 82703 88469 CDS product DNA packaging terminase subunit 1 83331-83350 4:01:01 CCACGCCGGTTCCTGAAGCC

84312 84336 84037 86157 CDS product DNA packaging tegument protein UL17 84312-84336 5:02:01 CCTACTATTCCTTCAGACCACCCCC

84743 84759 84037 86157 CDS product DNA packaging tegument protein UL17 84743-84759 4:01:01 GGCGCGGCTTGAGGAGG

85291 85305 84037 86157 CDS product DNA packaging tegument protein UL17 85291-85305 4:01:01 GGGGGGGTAGGAGGG

85458 85472 84037 86157 CDS product DNA packaging tegument protein UL17 85458-85472 4:01:01 CCACCGCCTGTGGCC

86218 86235 86176 87285 CDS product tegument protein UL16 86218-86235 4:01:01 CCTCGTTGTCCCCCAACC

86601 86624 86176 87285 CDS product tegument protein UL16 86601-86624 5:02:01 CCGTCCGACGTGCCCAACCCTGCC

86723 86748 86176 87285 CDS product tegument protein UL16 86723-86748 5:02:01 GGCACCGGGTGTTTGGTGGTATAAGG

86808 86830 86176 87285 CDS product tegument protein UL16 86808-86830 5:02:01 GGGTGGAAGGCCAGAGGTTTGGG

86996 87006 86176 87285 CDS product tegument protein UL16 86996-87006 4:01:01 GGTGGAGGGGG

87156 87171 86176 87285 CDS product tegument protein UL16 87156-87171 4:01:01 GGATGGAAACTGGTGG
87724 87735 82703 88469 CDS product DNA packaging terminase subunit 1 87724-87735 4:01:01 GGCGGATGGTGG
88888 88936 88499 89464 CDS product tegument protein UL14 88888-88936 9:06:02 GGTGGCAGCGGTGAAGCAGGCGAGTCGGAGGAATGGCTCGGTCACGAGG
89341 89369 88499 89464 CDS product tegument protein UL14 89341-89369 6:03:01 GGCCCGGCCAGTCAGGAATGGCTGGGTGG
89616 89635 88921 90717 CDS product tegument serine/threonine protein kinase 89616-89635 4:01:01 GGAGGTGCCGGAAGTTATGG
91036 91059 90699 92396 CDS product deoxyribonuclease 91036-91059 4:01:01 GGTGGGACAAATGGAGATTGAGGG
91428 91443 90699 92396 CDS product deoxyribonuclease 91428-91443 4:01:01 CCGAAGCCGCCACCCC
91550 91571 90699 92396 CDS product deoxyribonuclease 91550-91571 4:01:01 GGTGTTGTGGGGGCATCTTTGG
92266 92294 90699 92396 CDS product deoxyribonuclease 92266-92294 4:01:01 GGTGATTGTGGACCGCGAGGGTTGCTGGG
92467 92486 92348 92575 CDS product myristylated tegument protein 92467-92486 5:02:01 CCCCCCGCCCCCCCGAGGCC
93336 93355 92681 94033 CDS product envelope glycoprotein M 93336-93355 4:01:01 CCGCGCGTCCGTGGCCCGCC
94139 94160 93951 96614 CDS product DNA replication origin-binding helicase 94139-94160 4:01:01 GGGGGAGACTTGGAAGAGTGGG
95557 95575 93951 96614 CDS product DNA replication origin-binding helicase 95557-95575 4:01:01 CCAATTTCCCCTGACCGCC
96035 96050 93951 96614 CDS product DNA replication origin-binding helicase 96035-96050 4:01:01 GGGGGGTCCGGGCTGG
96627 96653 96626 98881 CDS product helicase-primase subunit 96627-96653 5:02:01 GGGGGAAAACGTGGAATGGTTTAATGG
96867 96899 96626 98881 CDS product helicase-primase subunit 96867-96899 5:02:01

GGTACCTGGGTGTTGGCCGTTGGCCCCTCTGGG
98134 98153 96626 98881 CDS product helicase-primase subunit 98134-98153 4:01:01 GGCTTTTGGGGGGCAGCTGG
99078 99100 98942 99850 CDS product tegument protein UL7 99078-99100 5:02:01 GGGGGCAGGGTTTATCGGGTTGG
99199 99232 98942 99850 CDS product tegument protein UL7 99199-99232 5:02:01 GGGGAGCACTCGGGTCTGGTCAAACGGGTTGTGG
99661 99687 98942 99850 CDS product tegument protein UL7 99661-99687 5:02:01 GGTCGTGAAGGAAGGGGGCAGCCCGGG
99780 99796 98942 99850 CDS product tegument protein UL7 99780-99796 4:01:01 CCTCCACCATTTCGTCC
100472 100488 99648 101891 CDS product capsid portal protein 100472-100488 4:01:01 CCCGCCGTTTCCAGCCC
101126 101156 99648 101891 CDS product capsid portal protein 101126-101156 6:03:01 CCTCGCCAGTTCAACCACAGCCTCCAACCCC
101952 101966 101875 104517 CDS product helicase-primase helicase subunit 101952-101966 4:01:01 CCGTCTACCTCCTCC
104775 104794 104567 105250 CDS product nuclear protein UL4 104775-104794 4:01:01 GGTGCTGGATTGTGGCGTGG
104822 104850 104567 105250 CDS product nuclear protein UL4 104822-104850 5:02:01 CCGATAGCACCAGAGTTCCACTAACCACC
105012 105030 104567 105250 CDS product nuclear protein UL4 105012-105030 4:01:01 CCATGCCCTGACCTGTACC
106466 106487 105971 106606 CDS product nuclear protein UL3 106466-106487 5:02:01 GGGGTTGTGGGAGCGGTGGTGG
106557 106578 105971 106606 CDS product nuclear protein UL3 106557-106578 5:02:01 CCTCGACCGGCCATCCCCACCC
107008 107036 106696 107640 CDS product uracil-DNA glycosylase 107008-107036 4:01:01 CCCTGACTGGCCCATTTATCCAAAAATCC
107154 107175 106696 107640 CDS product uracil-DNA glycosylase 107154-107175 4:01:01 GGGTGCATGGTACGGATCCTGG
107171 107196 106696 107640 CDS product uracil-DNA glycosylase 107171-107196 4:01:01 CCTGGCCCACGATAACCACACGAACC

107528 107552 106696 107640 CDS product uracil-DNA glycosylase 107528-107552 5:02:01
GGCTGGCTTTGCTGGTGGGTATTGG
110229 110260 110103 111713 CDS product ubiquitin E3 ligase ICP0 110229-110260 6:03:01
GGGGACAACGGGTCTGGGTATGGCACAGGTGG
110770 110788 110103 111713 CDS product ubiquitin E3 ligase ICP0 110770-110788 4:01:01 CCCCCACGGCTTCCCGACC
110858 110904 110103 111713 CDS product ubiquitin E3 ligase ICP0 110858-110904 7:04:01
CCTTCTACCCCGTCCCCCCCGTCTTCCCCGTCTTCCACGTCTACCCC
110912 110954 110103 111713 CDS product ubiquitin E3 ligase ICP0 110912-110954 6:03:01
GGGTGCGTTAGGCCTTGGGTGCACGGGTCTGGGGTTTTCTGGG
113124 113147 113094 117422 CDS product transcriptional regulator ICP4 113124-113147 6:03:01
CCGTCCTCCTCGTCCGATACCACC
113212 113233 113094 117422 CDS product transcriptional regulator ICP4 113212-113233 5:02:01
CCAGCTCCACGTCCCCCGGTCC
113313 113337 113094 117422 CDS product transcriptional regulator ICP4 113313-113337 4:01:01
CCGAACCCCGGCTCCCACTCAACCC
113340 113356 113094 117422 CDS product transcriptional regulator ICP4 113340-113356 4:01:01
GGGGCTCGGCGGCGAGG
113456 113475 113094 117422 CDS product transcriptional regulator ICP4 113456-113475 4:01:01
GGCGGGCTCCACGGCCCCGG
113464 113488 113094 117422 CDS product transcriptional regulator ICP4 113464-113488 5:02:01
CCACGGCCCCGGCTCCGCGCCGTCC
113524 113556 113094 117422 CDS product transcriptional regulator ICP4 113524-113556 6:03:01
GGCCCCATCGGTTGGCCGCGCGGTGGCTCTGGG

113664 113682 113094 117422 CDS product transcriptional regulator ICP4 113664-113682 4:01:01
GGTGGCAGTGGAACGCGGG
113691 113730 113094 117422 CDS product transcriptional regulator ICP4 113691-113730 7:04:01
CCGGCCCGGGTGTCCACCGTGTAGGCCACGTTGGCCGCCC
113812 113857 113094 117422 CDS product transcriptional regulator ICP4 113812-113857 7:04:01
GGGTGCTGGAAGTGAGGACCGCGCGGCTGAGGTGGCGCTCCCGGGG
113911 113929 113094 117422 CDS product transcriptional regulator ICP4 113911-113929 4:01:01
GGTACTGGCTGATGGCGGG
113931 113948 113094 117422 CDS product transcriptional regulator ICP4 113931-113948 4:01:01
CCGTCCTGGGGCCAGTCC
113987 114005 113094 117422 CDS product transcriptional regulator ICP4 113987-114005 4:01:01
GGCGGAGCCCAGGCGAAGG
114047 114078 113094 117422 CDS product transcriptional regulator ICP4 114047-114078 5:02:01
GGAGAGGAGCAGGACTCCCTGGGCGTTTAGGG
114095 114114 113094 117422 CDS product transcriptional regulator ICP4 114095-114114 4:01:01
CCCAGTCCAGTTGCCAGCCC
114139 114179 113094 117422 CDS product transcriptional regulator ICP4 114139-114179 7:04:01
CCCGGTTCCCCAGGGCCGCCAGCAGCGCCGAGAGCCCCCCC
114218 114233 113094 117422 CDS product transcriptional regulator ICP4 114218-114233 4:01:01 GGGTCGGGTGGTTGGG
114237 114257 113094 117422 CDS product transcriptional regulator ICP4 114237-114257 4:01:01
CCACCCAGCAGGTCCTCGTCC
114320 114348 113094 117422 CDS product transcriptional regulator ICP4 114320-114348 6:03:01
GGCAGCCCGGCGGCGGAGCGGTCCGCTGG

114372 114391 113094 117422 CDS product transcriptional regulator ICP4 114372-114391 4:01:01
CCCTCCCTGGCCGGAGGCCC
114400 114473 113094 117422 CDS product transcriptional regulator ICP4 114400-114473 11:08:02
GGGCTGCGATGGTAGCCAGGGCCTGGGGGTCGAAGGTAAGCGCGGGGCGCCAGGCCTCGGGGAACAGCGGGTGG
114530 114545 113094 117422 CDS product transcriptional regulator ICP4 114530-114545 4:01:01 GGGGGGTGGCGTGTGG
114580 114607 113094 117422 CDS product transcriptional regulator ICP4 114580-114607 6:03:01
GGCGGTGTGGCCCCTCGGGCGGCATGGG
114687 114725 113094 117422 CDS product transcriptional regulator ICP4 114687-114725 8:05:02
CCTCCGCGGGACCCCGGGAGCTCCCCCGCCGCCGGCTCC
114740 114769 113094 117422 CDS product transcriptional regulator ICP4 114740-114769 4:01:01
CCGCTTCCCGCTCTTGCCCCTGGGGCGCCC
114776 114808 113094 117422 CDS product transcriptional regulator ICP4 114776-114808 6:03:01
GGGACGGTCGTTCGGGGAGGCGTAGGGTGCCGG
114805 114821 113094 117422 CDS product transcriptional regulator ICP4 114805-114821 5:02:01
CCGGTCCGCCCCCTCCC
114844 114876 113094 117422 CDS product transcriptional regulator ICP4 114844-114876 6:03:01
GGCCGAGGATAGCCTGGGAGGCAGCCGGTGGGG
114896 114937 113094 117422 CDS product transcriptional regulator ICP4 114896-114937 7:04:01
GGGGCGAGCCTGGGTCTGGGTGGCCCGGGAGCAGGTTGTCGG
115002 115049 113094 117422 CDS product transcriptional regulator ICP4 115002-115049 8:05:02
GGCTTCCCGGGAACAGTGGGCCACAAGGCGGGGATGCGGGAGGTCTGG
115058 115071 113094 117422 CDS product transcriptional regulator ICP4 115058-115071 4:01:01 GGAGGAGGAGAGGG
115137 115166 113094 117422 CDS product transcriptional regulator ICP4 115137-115166 6:03:01

CCCACGGCTTCCCCCAACATGCCCCCGGCC
115187 115224 113094 117422 CDS product transcriptional regulator ICP4 115187-115224 7:04:01
GGCCTGGGTGATGGTCCAGGCCGAGGCCCGGGCCCGGG
115202 115232 113094 117422 CDS product transcriptional regulator ICP4 115202-115232 6:03:01
CCAGGCCGAGGCCCGGGCCCGGGCTCCCTCC
115267 115289 113094 117422 CDS product transcriptional regulator ICP4 115267-115289 4:01:01
CCACAACCAGAACCGCGCGGACC
115307 115328 113094 117422 CDS product transcriptional regulator ICP4 115307-115328 5:02:01
GGGGCCCAGGCGGTGGATAGGG
115430 115451 113094 117422 CDS product transcriptional regulator ICP4 115430-115451 5:02:01
GGGAGGGCAGGCGGGAGAGGGG
115568 115605 113094 117422 CDS product transcriptional regulator ICP4 115568-115605 8:05:02
GGCGGTAGGCTTGGGGGTTCTGGTACCAGGACCCGCGG
115593 115610 113094 117422 CDS product transcriptional regulator ICP4 115593-115610 4:01:01
CCAGGACCCGCGGCCGCC
115619 115644 113094 117422 CDS product transcriptional regulator ICP4 115619-115644 4:01:01
CCGTGGCCCGAGGGCCGCGCACACCC
115676 115690 113094 117422 CDS product transcriptional regulator ICP4 115676-115690 4:01:01 GGCCGGGGGATCCGG
115729 115745 113094 117422 CDS product transcriptional regulator ICP4 115729-115745 4:01:01
CCGTGACCCCGGCCGCC
115840 115869 113094 117422 CDS product transcriptional regulator ICP4 115840-115869 5:02:01
GGTCGTAGCGGCGGCTCATGGCCACCGAGG
115901 115921 113094 117422 CDS product transcriptional regulator ICP4 115901-115921 4:01:01

CCCGGCCGCCATGGCGTCCCC
115996 116022 113094 117422 CDS product transcriptional regulator ICP4 115996-116022 5:02:01
GGCAGAACTGGGCCAGGTTCTGGTCGG
116136 116157 113094 117422 CDS product transcriptional regulator ICP4 116136-116157 5:02:01
GGTACGGCGGTCTGGGCTTCGG
116194 116227 113094 117422 CDS product transcriptional regulator ICP4 116194-116227 6:03:01
CCCAGAGCCCGTCCCGCGAGTCCCCGGTTCCCCC
116264 116284 113094 117422 CDS product transcriptional regulator ICP4 116264-116284 4:01:01
GGGCCATGGGTCTCCGGATGG
116370 116426 113094 117422 CDS product transcriptional regulator ICP4 116370-116426 11:08:02
GGGAGGGCCTCTTCGGCCCCGGTTGTGGCGGCGGTCTGGGGTATGGGTCTTGGGTGG
116557 116570 113094 117422 CDS product transcriptional regulator ICP4 116557-116570 4:01:01 CCCTAACCCCCTCC
116595 116637 113094 117422 CDS product transcriptional regulator ICP4 116595-116637 6:03:01
GGGGCGAGGGGCCCGCCCGGGTTACTCGGGGTGGGAGCGCCGG
116742 116797 113094 117422 CDS product transcriptional regulator ICP4 116742-116797 11:08:02
GGGGACCCGGTGGCCGGTTGGGTTTTTGCGGCGCTCGGCGAGGGATGGGGGCGAGG
116930 116954 113094 117422 CDS product transcriptional regulator ICP4 116930-116954 5:02:01
CCGGCCCCTTGGCGCCGGCCTCCCC
116957 117009 113094 117422 CDS product transcriptional regulator ICP4 116957-117009 10:07:02
GGGGGAGGCCGAGGGTGGAAACTCGGCCCCGGGAGACCCCGGGCAGGTCTCGG
117039 117083 113094 117422 CDS product transcriptional regulator ICP4 117039-117083 8:05:02
CCTCCGCCCGATGATCCGGTGTCCCTGCGACCGGCCCCTGTAGCC
117166 117190 113094 117422 CDS product transcriptional regulator ICP4 117166-117190 5:02:01

CCGGAGACCGCTTTGCCTTCCCGCC
117193 117221 113094 117422 CDS product transcriptional regulator ICP4 117193-117221 7:04:01
GGGGCTCGGGGGCCGGGGAAGGCGGCGGG
117256 117280 113094 117422 CDS product transcriptional regulator ICP4 117256-117280 4:01:01
CCACCCCAAACATCCCCTGGCTTCC
117294 117311 113094 117422 CDS product transcriptional regulator ICP4 117294-117311 4:01:01
GGGGCGGCCGGGGTATGG
117323 117347 113094 117422 CDS product transcriptional regulator ICP4 117323-117347 4:01:01
GGCCGCGCTGGCTGCGCGGATGAGG
120131 120166 120069 120923 CDS product regulatory protein ICP22 120131-120166 8:05:02
CCGTCCACCAGCCCCATCATACCCTCCCTGTCCCCC
120181 120200 120069 120923 CDS product regulatory protein ICP22 120181-120200 4:01:01 CCCATCCCCACGCTCCAGCC
120599 120623 120069 120923 CDS product regulatory protein ICP22 120599-120623 5:02:01
CCGCCCAGCAAGCCACCCAAAACCC
120670 120687 120069 120923 CDS product regulatory protein ICP22 120670-120687 4:01:01 GGAGGAAGAGGCTAGCGG
120823 120851 120069 120923 CDS product regulatory protein ICP22 120823-120851 6:03:01
CCCCACCAACCCTCCCACCACACGTGCCC
123126 123138 122631 123491 CDS product virion protein V67 123126-123138 4:01:01 GGAGGTGGTAGGG
123669 123688 123585 124559 CDS product virion protein US2 123669-123688 4:01:01 GGAGATAGGGTGCTAGGTGG
125395 125435 124695 125849 CDS product serine/threonine protein kinase US3 125395-125435 7:04:01
GGTTGTGGACCCCATGGACCTTGGTTTGGCTGGTACCGTGG
126059 126083 125970 127277 CDS product envelope glycoprotein G 126059-126083 4:01:01
CCCCGCAAAACCGGTCCCATGCCCC

127602 127642 127455 129707 CDS product envelope glycoprotein J 127602-127642 7:04:01
CCCCCACCACGGGCCCCACTACCACATCTTCCCAAACATCC
128583 128595 127455 129707 CDS product envelope glycoprotein J 128583-128595 4:01:01 CCACCAAACCCCC
128961 128982 127455 129707 CDS product envelope glycoprotein J 128961-128982 5:02:01 CCCCACCCCCACAAGGCCAACC
129282 129302 127455 129707 CDS product envelope glycoprotein J 129282-129302 4:01:01 CCACAATACCATGTCCAGGCC
129897 129913 129798 131006 CDS product envelope glycoprotein D 129897-129913 4:01:01 GGCGTGTGGTTCGGGGG
129929 129947 129798 131006 CDS product envelope glycoprotein D 129929-129947 4:01:01 CCCGAGTTTCCACCACCCC
130718 130734 129798 131006 CDS product envelope glycoprotein D 130718-130734 4:01:01 CCAAAACCCAATCCACC
131507 131529 131111 132373 CDS product envelope glycoprotein I 131507-131529 4:01:01 CCCGGCCACAGCCCGCAGATTCC
131626 131643 131111 132373 CDS product envelope glycoprotein I 131626-131643 4:01:01 CCAGAGCCAGTTCCAACC
131755 131772 131111 132373 CDS product envelope glycoprotein I 131755-131772 4:01:01 CCGACTCCTCGCCCAGCC
131956 131985 131111 132373 CDS product envelope glycoprotein I 131956-131985 6:03:01
CCACCCCCCAAACCGCAAACCACTTCTTCC
132203 132222 131111 132373 CDS product envelope glycoprotein I 132203-132222 4:01:01 GGAGCCGGTTGGCGTGTGGG
132269 132290 131111 132373 CDS product envelope glycoprotein I 132269-132290 5:02:01 CCTCGCCACCATCCCACAAGCC
133362 133389 132593 134239 CDS product envelope glycoprotein E 133362-133389 5:02:01
CCCAACCGCTATGGCCTGCCTGCACCCC
133618 133635 132593 134239 CDS product envelope glycoprotein E 133618-133635 4:01:01 GGACATCCGGAGGCGTGG
134041 134058 132593 134239 CDS product envelope glycoprotein E 134041-134058 5:02:01 CCACAACCTCCACCAGCC
134107 134130 132593 134239 CDS product envelope glycoprotein E 134107-134130 5:02:01 GGCCGGTCGGGTTTCAAGGTCTGG
134173 134192 132593 134239 CDS product envelope glycoprotein E 134173-134192 4:01:01 CCAACCCCACCCGTCGGACC
134470 134492 134273 134605 CDS product membrane protein US8A 134470-134492 5:02:01 CCACCATCTCCAGAACCCGAGCC
135041 135054 134911 135573 CDS product membrane protein US9 135041-135054 4:01:01 GGCAGAGGCGGTGG

135065 135087 134911 135573 CDS product membrane protein US9 135065-135087 4:01:01 GGAGGTAGCTACGGCTGCTTTGG
135206 135224 134911 135573 CDS product membrane protein US9 135206-135224 4:01:01 CCCCCCGTCCCCAGAGGCC
135221 135246 134911 135573 CDS product membrane protein US9 135221-135246 5:02:01
GGCCGTAGGTATTGAGGATGTGGTGG
135418 135435 134911 135573 CDS product membrane protein US9 135418-135435 4:01:01 CCGCCAGCCTGTTCATCC
135439 135473 134911 135573 CDS product membrane protein US9 135439-135473 8:05:02
GGGTCGGCCGGCGGCAGGCGCGGCGACACAGGCGG
135968 136035 135917 136603 CDS product virion protein US10 135968-136035 13:10:03
GGGGGCAGGGGGAGCGGGAGCTTTCGGCCTCGGGTACGCAGGTACGCGGCGGCGGCGGCGTGTCGGGG
136044 136079 135917 136603 CDS product virion protein US10 136044-136079 7:04:01
CCGGATCGGCCCCCGCCATGTCCAGCAGCGCCACCC
136253 136276 135917 136603 CDS product virion protein US10 136253-136276 4:01:01 GGCGCGGCGCCTAGGTCTACCAGG
136272 136288 135917 136603 CDS product virion protein US10 136272-136288 4:01:01 CCAGGTACCTCCCCACC
136525 136551 135917 136603 CDS product virion protein US10 136525-136551 6:03:01 CCCCTCACCGCCTCCGCAGTCTCCCCC
136562 136576 135917 136603 CDS product virion protein US10 136562-136576 4:01:01 CCCCCGTCTACCGCC
137197 137225 137127 137981 CDS product regulatory protein ICP22 137197-137225 6:03:01
GGGCACGTGTGGTGGGAGGGTTGGTGGGG
137361 137378 137127 137981 CDS product regulatory protein ICP22 137361-137378 4:01:01 CCGCTAGCCTCTTCCTCC
137425 137449 137127 137981 CDS product regulatory protein ICP22 137425-137449 5:02:01
GGGTTTTGGGTGGCTTGCTGGGCGG
137848 137867 137127 137981 CDS product regulatory protein ICP22 137848-137867 4:01:01 GGCTGGAGCGTGGGGATGGG
137882 137917 137127 137981 CDS product regulatory protein ICP22 137882-137917 8:05:02
GGGGGACAGGGAGGGTATGATGGGGCTGGTGGACGG

140701 140725 140628 144956 CDS product transcriptional regulator ICP4 140701-140725 4:01:01
CCTCATCCGCGCAGCCAGCGCGGCC
140737 140754 140628 144956 CDS product transcriptional regulator ICP4 140737-140754 4:01:01
CCATACCCCGGCCGCCCC
140768 140792 140628 144956 CDS product transcriptional regulator ICP4 140768-140792 4:01:01
GGAAGCCAGGGGATGTTTGGGGTGG
140827 140855 140628 144956 CDS product transcriptional regulator ICP4 140827-140855 7:04:01
CCCGCCGCCTTCCCCGGCCCCCGAGCCCC
140858 140882 140628 144956 CDS product transcriptional regulator ICP4 140858-140882 5:02:01
GGCGGGAAGGCAAAGCGGTCTCCGG
140965 141009 140628 144956 CDS product transcriptional regulator ICP4 140965-141009 8:05:02
GGCTACAGGGGCCGGTCGCAGGGACACCGGATCATCGGGCGGAGG
141039 141091 140628 144956 CDS product transcriptional regulator ICP4 141039-141091 10:07:02
CCGAGACCTGCCCGGGGTCTCCCGGGGCCGAGTTTCCACCCTCGGCCTCCCCC
141094 141118 140628 144956 CDS product transcriptional regulator ICP4 141094-141118 5:02:01
GGGGAGGCCGGCGCCAAGGGGCCGG
141251 141306 140628 144956 CDS product transcriptional regulator ICP4 141251-141306 11:08:02
CCTCGCCCCCATCCCTCGCCGAGCGCCGCAAAAACCCAACCGGCCACCGGGTCCCC
141411 141453 140628 144956 CDS product transcriptional regulator ICP4 141411-141453 6:03:01
CCGGCGCTCCCACCCCGAGTAACCCGGGCGGGCCCCTCGCCCC
141478 141491 140628 144956 CDS product transcriptional regulator ICP4 141478-141491 4:01:01 GGAGGGGGTTAGGG
141622 141678 140628 144956 CDS product transcriptional regulator ICP4 141622-141678 11:08:02
CCACCCAAGACCCATACCCCAGACCGCCGCCACAACCGGGGCCGAAGAGGCCCTCCC

141764 141784 140628 144956 CDS product transcriptional regulator ICP4 141764-141784 4:01:01
CCATCCGGAGACCCATGGCCC
141821 141854 140628 144956 CDS product transcriptional regulator ICP4 141821-141854 6:03:01
GGGGGAACCGGGGACTCGCGGGACGGGCTCTGGG
141891 141912 140628 144956 CDS product transcriptional regulator ICP4 141891-141912 5:02:01
CCGAAGCCCAGACCGCCGTACC
142026 142052 140628 144956 CDS product transcriptional regulator ICP4 142026-142052 5:02:01
CCGACCAGAACCTGGCCCAGTTCTGCC
142127 142147 140628 144956 CDS product transcriptional regulator ICP4 142127-142147 4:01:01
GGGGACGCCATGGCGGCCGGG
142179 142208 140628 144956 CDS product transcriptional regulator ICP4 142179-142208 5:02:01
CCTCGGTGGCCATGAGCCGCCGCTACGACC
142303 142319 140628 144956 CDS product transcriptional regulator ICP4 142303-142319 4:01:01
GGCGGCCGGGGTCACGG
142358 142372 140628 144956 CDS product transcriptional regulator ICP4 142358-142372 4:01:01 CCGGATCCCCCGGCC
142404 142429 140628 144956 CDS product transcriptional regulator ICP4 142404-142429 4:01:01
GGGTGTGCGCGGCCCTCGGGCCACGG
142438 142455 140628 144956 CDS product transcriptional regulator ICP4 142438-142455 4:01:01
GGCGGCCGCGGGTCCTGG
142443 142480 140628 144956 CDS product transcriptional regulator ICP4 142443-142480 8:05:02
CCGCGGGTCCTGGTACCAGAACCCCCAAGCCTACCGCC
142597 142618 140628 144956 CDS product transcriptional regulator ICP4 142597-142618 5:02:01
CCCCTCTCCCGCCTGCCCTCCC

142720 142741 140628 144956 CDS product transcriptional regulator ICP4 142720-142741 5:02:01
CCCTATCCACCGCCTGGGCCCC
142759 142781 140628 144956 CDS product transcriptional regulator ICP4 142759-142781 4:01:01
GGTCCGCGCGGTTCTGGTTGTGG
142816 142846 140628 144956 CDS product transcriptional regulator ICP4 142816-142846 6:03:01
GGAGGGAGCCCGGGCCCGGGCCTCGGCCTGG
142824 142861 140628 144956 CDS product transcriptional regulator ICP4 142824-142861 7:04:01
CCCGGGCCCGGGCCTCGGCCTGGACCATCACCCAGGCC
142882 142911 140628 144956 CDS product transcriptional regulator ICP4 142882-142911 6:03:01
GGCCGGGGGCATGTTGGGGGAAGCCGTGGG
142977 142990 140628 144956 CDS product transcriptional regulator ICP4 142977-142990 4:01:01 CCCTCTCCTCCTCC
142999 143046 140628 144956 CDS product transcriptional regulator ICP4 142999-143046 8:05:02
CCAGACCTCCCGCATCCCCGCCTTGTGGCCCACTGTTCCCGGGAAGCC
143111 143152 140628 144956 CDS product transcriptional regulator ICP4 143111-143152 7:04:01
CCGACAACCTGCTCCCGGGCCACCCAGACCCAGGCTCGCCCC
143172 143204 140628 144956 CDS product transcriptional regulator ICP4 143172-143204 6:03:01
CCCCACCGGCTGCCTCCCAGGCTATCCTCGGCC
143227 143243 140628 144956 CDS product transcriptional regulator ICP4 143227-143243 5:02:01
GGGAGGGGGCGGACCGG
143240 143272 140628 144956 CDS product transcriptional regulator ICP4 143240-143272 6:03:01
CCGGCACCCTACGCCTCCCCGAACGACCGTCCC
143279 143308 140628 144956 CDS product transcriptional regulator ICP4 143279-143308 4:01:01
GGGCGCCCCAGGGGCAAGAGCGGGAAGCGG

143323 143361 140628 144956 CDS product transcriptional regulator ICP4 143323-143361 8:05:02
GGAGCCGGCGGCGGGGGAGCTCCCGGGGTCCCGCGGAGG
143441 143468 140628 144956 CDS product transcriptional regulator ICP4 143441-143468 6:03:01
CCCATGCCGCCCGAGGGGCCACACCGCC
143503 143518 140628 144956 CDS product transcriptional regulator ICP4 143503-143518 4:01:01 CCACACGCCACCCCCC
143575 143648 140628 144956 CDS product transcriptional regulator ICP4 143575-143648 11:08:02
CCACCCGCTGTTCCCCGAGGCCTGGCGCCCCGCGCTTACCTTCGACCCCCAGGCCCTGGCTACCATCGCAGCCC
143657 143676 140628 144956 CDS product transcriptional regulator ICP4 143657-143676 4:01:01
GGGCCTCCGGCCAGGGAGGG
143700 143728 140628 144956 CDS product transcriptional regulator ICP4 143700-143728 6:03:01
CCAGCGGACCGCTCCGCCGCCGGGCTGCC
143791 143811 140628 144956 CDS product transcriptional regulator ICP4 143791-143811 4:01:01
GGACGAGGACCTGCTGGGTGG
143815 143830 140628 144956 CDS product transcriptional regulator ICP4 143815-143830 4:01:01 CCCAACCACCCGACCC
143869 143909 140628 144956 CDS product transcriptional regulator ICP4 143869-143909 7:04:01
GGGGGGGCTCTCGGCGCTGCTGGCGGCCCTGGGGAACCGGG
143934 143953 140628 144956 CDS product transcriptional regulator ICP4 143934-143953 4:01:01
GGGCTGGCAACTGGACTGGG
143970 144001 140628 144956 CDS product transcriptional regulator ICP4 143970-144001 5:02:01
CCCTAAACGCCCAGGGAGTCCTGCTCCTCTCC
144043 144061 140628 144956 CDS product transcriptional regulator ICP4 144043-144061 4:01:01
CCTTCGCCTGGGCTCCGCC
144100 144117 140628 144956 CDS product transcriptional regulator ICP4 144100-144117 4:01:01

GGACTGGCCCCAGGACGG

144119 144137 140628 144956 CDS product transcriptional regulator ICP4 144119-144137 4:01:01

CCCGCCATCAGCCAGTACC

144191 144236 140628 144956 CDS product transcriptional regulator ICP4 144191-144236 7:04:01

CCCCGGGAGCGCCACCTCAGCCGCGCGGTCCTCACTTCCAGCACCC

144318 144357 140628 144956 CDS product transcriptional regulator ICP4 144318-144357 7:04:01

GGGCGGCCAACGTGGCCTACACGGTGGACACCCGGGCCGG

144366 144384 140628 144956 CDS product transcriptional regulator ICP4 144366-144384 4:01:01

CCCGCGTTCCACTGCCACC

144492 144524 140628 144956 CDS product transcriptional regulator ICP4 144492-144524 6:03:01

CCCAGAGCCACCGCGCGGCCAACCGATGGGGCC

144560 144584 140628 144956 CDS product transcriptional regulator ICP4 144560-144584 5:02:01

GGACGGCGCGGAGCCGGGGCCGTGG

144573 144592 140628 144956 CDS product transcriptional regulator ICP4 144573-144592 4:01:01

CCGGGGCCGTGGAGCCCGCC

144692 144708 140628 144956 CDS product transcriptional regulator ICP4 144692-144708 4:01:01

CCTCGCCGCCGAGCCCC

144711 144735 140628 144956 CDS product transcriptional regulator ICP4 144711-144735 4:01:01

GGGTTGAGTGGGAGCCGGGGTTCGG

144815 144836 140628 144956 CDS product transcriptional regulator ICP4 144815-144836 5:02:01

GGACCGGGGGACGTGGAGCTGG

144901 144924 140628 144956 CDS product transcriptional regulator ICP4 144901-144924 6:03:01

GGTGGTATCGGACGAGGAGGACGG

gi|9629818|ref|NC_001847.1| Bovine herpesvirus 1, complete genome
492 530 486 1229 CDS product myristylated tegument protein CIRC 492-530 9:06:02
CCCGCGCCTCCGCGCCTGCTGCCGGCCCGCCCCCAGCCC
619 656 486 1229 CDS product myristylated tegument protein CIRC 619-656 6:03:01
CCCCGTGTACCACCACATGCGCCGCGGCCGCACGGCCC
682 707 486 1229 CDS product myristylated tegument protein CIRC 682-707 5:02:01 CCGCGCCTATGCCATCTTGCCCTGCC
758 784 486 1229 CDS product myristylated tegument protein CIRC 758-784 6:03:01 CCCACCACTGGCCTGCACCGCCTCGCC
888 902 486 1229 CDS product myristylated tegument protein CIRC 888-902 4:01:01 CCACCCCCGAGATCC
922 964 486 1229 CDS product myristylated tegument protein CIRC 922-964 9:06:02
CCCGGCCTTCCCACCGCCGCCCTCGGCCCGCGCGCGCCGCTCC
988 1016 486 1229 CDS product myristylated tegument protein CIRC 988-1016 6:03:01
CCGGCCCGCACGCACCTTCTGCCCCCACC
1918 1949 1658 2860 CDS product multifunctional expression regulator 1918-1949 6:03:01
GGGGCAGGTGGCGAGGCTTAGCCGGCGCGCGG
2223 2242 1658 2860 CDS product multifunctional expression regulator 2223-2242 4:01:01
GGTAGAGGTCGCGGCCGTGG
2260 2280 1658 2860 CDS product multifunctional expression regulator 2260-2280 4:01:01
GGTGACGCGGCGGCCCTCGGG
2486 2496 1658 2860 CDS product multifunctional expression regulator 2486-2496 4:01:01 CCCCCGCCGCC
2584 2603 1658 2860 CDS product multifunctional expression regulator 2584-2603 4:01:01
CCTGCGCGGCCGCCGCGGCC
2624 2640 1658 2860 CDS product multifunctional expression regulator 2624-2640 4:01:01

GGAGGTGCTTCGGCGGG
2672 2691 1658 2860 CDS product multifunctional expression regulator 2672-2691 5:02:01 GGCGGCTCGGCCGGCATCGG
2697 2710 1658 2860 CDS product multifunctional expression regulator 2697-2710 4:01:01 CCTCCGCGCCCTCC
3189 3218 3040 4038 CDS product envelope glycoprotein K 3189-3218 6:03:01 CCCGCTCTCGCCGCCGTCGCCGCCATCGCC
3229 3241 3040 4038 CDS product envelope glycoprotein K 3229-3241 4:01:01 CCTCCGCGCCGCC
3317 3345 3040 4038 CDS product envelope glycoprotein K 3317-3345 5:02:01 GGTACAGCGGGTAGGCGGCCGCGCAGGGG
3355 3391 3040 4038 CDS product envelope glycoprotein K 3355-3391 7:04:01 CCGACGCTGCCGGCCGCCGCAATCCGCGCGCCGAGCC
3449 3462 3040 4038 CDS product envelope glycoprotein K 3449-3462 4:01:01 GGGGGTCGAGGCGG
3672 3696 3040 4038 CDS product envelope glycoprotein K 3672-3696 5:02:01 CCATGCCAGCAGGCCGCCGCGAGCC
3807 3829 3040 4038 CDS product envelope glycoprotein K 3807-3829 4:01:01 GGCGTGCCGGCACGGGGCGTCGG
4039 4063 4013 7237 CDS product helicase-primase primase subunit 4039-4063 4:01:01 CCGCGCCGCGCGCCCCGAGAGATCC
4066 4087 4013 7237 CDS product helicase-primase primase subunit 4066-4087 4:01:01 GGTGAACACGGTACCGAGGCGG
4276 4294 4013 7237 CDS product helicase-primase primase subunit 4276-4294 4:01:01 GGCTTTGGCGGCCACGAGG
4400 4422 4013 7237 CDS product helicase-primase primase subunit 4400-4422 4:01:01 CCACGGTCGCCGCCGTCGACGCC

4468 4487 4013 7237 CDS product helicase-primase primase subunit 4468-4487 4:01:01
GGCCGCGCGGTTGACGGCGG
4558 4616 4013 7237 CDS product helicase-primase primase subunit 4558-4616 11:08:02
GGGTGGCGCGGCGTGGAAGTGGAAGTGGTGCGGGTCGCGGTGCGCGGCGACGAAGGCGG
4770 4792 4013 7237 CDS product helicase-primase primase subunit 4770-4792 4:01:01
GGCTCATGGCCTCGGCGAAGGGG
4932 4963 4013 7237 CDS product helicase-primase primase subunit 4932-4963 7:04:01
CCGCCGCGCTTCCCGCGCCCTCCGCGCCGCCC
5070 5091 4013 7237 CDS product helicase-primase primase subunit 5070-5091 5:02:01
GGTCGGGCGCGGCCCAGGACGG
5158 5194 4013 7237 CDS product helicase-primase primase subunit 5158-5194 6:03:01
GGGGTGTGCGGGCCAGGCATCGGCGGCGATTTCGGGG
5253 5281 4013 7237 CDS product helicase-primase primase subunit 5253-5281 6:03:01
GGTCTCCGCGGGGGAGGGGGCGCTTGAGG
5308 5324 4013 7237 CDS product helicase-primase primase subunit 5308-5324 4:01:01
GGTTACGGCGAGGCGGG
5358 5414 4013 7237 CDS product helicase-primase primase subunit 5358-5414 11:08:02
GGTCGCGCGGCGCGGCCGGGCCCAGGCTCCGGCGGTCCGAGCGGCCGGCGGCACAGG
5373 5403 4013 7237 CDS product helicase-primase primase subunit 5373-5403 5:02:01
CCGGGCCCAGGCTCCGGCGGTCCGAGCGGCC
5485 5498 4013 7237 CDS product helicase-primase primase subunit 5485-5498 4:01:01 GGCGGCGGCAGCGG
5499 5518 4013 7237 CDS product helicase-primase primase subunit 5499-5518 5:02:01
CCGCCGCAACCTCGACCGCC

5616 5639 4013 7237 CDS product helicase-primase primase subunit 5616-5639 4:01:01
GGTAGACGGGCGTGGGCGCCGCGG
5634 5665 4013 7237 CDS product helicase-primase primase subunit 5634-5665 5:02:01
CCGCGGTCCCGCGCGCGCCAAAGAGCCCGTCC
5759 5775 4013 7237 CDS product helicase-primase primase subunit 5759-5775 4:01:01
CCGCCGTCCGGCGCGCC
6151 6188 4013 7237 CDS product helicase-primase primase subunit 6151-6188 8:05:02
GGGGGGCTCGGTGGCGGTGACGGCGCGCAGGTGCTCGG
6297 6320 4013 7237 CDS product helicase-primase primase subunit 6297-6320 5:02:01
GGCAGCGGACCTCGGCGAGGTCGG
6445 6481 4013 7237 CDS product helicase-primase primase subunit 6445-6481 7:04:01
GGGGTTAGGCGGCGCGGCGACGGCGTACGTGGCCAGG
6493 6524 4013 7237 CDS product helicase-primase primase subunit 6493-6524 5:02:01
GGCCTGCAGGTCGTAGGTAGCGGCGTCGCCGG
6682 6706 4013 7237 CDS product helicase-primase primase subunit 6682-6706 6:03:01
GGCTAGGCCGCGGCGGCCGGCCGGG
6766 6783 4013 7237 CDS product helicase-primase primase subunit 6766-6783 4:01:01
GGTGCGGCCGGTGCGAGG
6786 6805 4013 7237 CDS product helicase-primase primase subunit 6786-6805 4:01:01
CCAGCTGCTCCGTGGCCACC
6957 6980 4013 7237 CDS product helicase-primase primase subunit 6957-6980 5:02:01
GGGCCAGGCGCGGGGTCGCGGCGG
6986 7003 4013 7237 CDS product helicase-primase primase subunit 6986-7003 4:01:01

CCGCCCGGCGGGCCGCCC

7027 7051 4013 7237 CDS product helicase-primase primase subunit 7027-7051 5:02:01 CCGCGCATCCGCCAGCGCCAGCGCC

7124 7147 4013 7237 CDS product helicase-primase primase subunit 7124-7147 5:02:01 CCACGGCCGGGCTCCTCCTGGCCC

7186 7204 4013 7237 CDS product helicase-primase primase subunit 7186-7204 4:01:01 GGTGGCGTACAGGATTTGG

7248 7281 7236 7967 CDS product tegument protein UL51 7248-7281 8:05:02 GGCTGGTCGGGTGGCTGTGCGGGCGCGGCGGGGG

7399 7435 7236 7967 CDS product tegument protein UL51 7399-7435 7:04:01 GGAGGACGTGGCGCGCTCGGCAGACGGCACGCGGCGG

7584 7597 7236 7967 CDS product tegument protein UL51 7584-7597 4:01:01 CCTGCCTGGCCGCC

7618 7661 7236 7967 CDS product tegument protein UL51 7618-7661 8:05:02 GGTGGGCGCTGTGGAGGGCACGACGGACAGCATGGTGGACCAGG

7726 7769 7236 7967 CDS product tegument protein UL51 7726-7769 9:06:02 GGGCATTCAGGCGCAGGGCGCGGGCGCGGAGGCGGCGGCCAGGG

7780 7802 7236 7967 CDS product tegument protein UL51 7780-7802 4:01:01 GGCCGTTGGTGCCGAGGCTCGGG

7803 7875 7236 7967 CDS product tegument protein UL51 7803-7875 13:10:03 CCCCGCCGCCCCTGCCGCGGCAGCCGGCCGCCTCGGCGCCGGTCGCCGTGTCTCCGGCACCGCCGGTGGCGCC

7821 7844 7236 7967 CDS product tegument protein UL51 7821-7844 4:01:01 GGCAGCCGGCCGCCTCGGCGCCGG

7887 7907 7236 7967 CDS product tegument protein UL51 7887-7907 5:02:01 CCGCCACAAAACCACCTAGCC

7926 7945 7236 7967 CDS product tegument protein UL51 7926-7945 5:02:01 CCGCCGCTTCTTCCCCCGCC

8050 8076 8045 9022 CDS product deoxyuridine triphosphatase 8050-8076 4:01:01

CCCGGTGGACCCAAAGCCGCGCGGCCC

8130 8154 8045 9022 CDS product deoxyuridine triphosphatase 8130-8154 5:02:01 GGCGCGGGGCCCCAGGGGCGGGCGG

8165 8205 8045 9022 CDS product deoxyuridine triphosphatase 8165-8205 7:04:01 GGGCTAGTTGGGAAGGGCTCGCGGTCGTTGAGGCCGGACGG

8304 8338 8045 9022 CDS product deoxyuridine triphosphatase 8304-8338 7:04:01 GGCAGGGCTCCCCGGGGGGCCAGGCTGTCGGCAGG

8506 8529 8045 9022 CDS product deoxyuridine triphosphatase 8506-8529 4:01:01 GGGGGCGAACGTGGCAAAGAAGGG

8575 8600 8045 9022 CDS product deoxyuridine triphosphatase 8575-8600 5:02:01 GGCGATGAGGCGAGGCAGCGTGGTGG

8639 8664 8045 9022 CDS product deoxyuridine triphosphatase 8639-8664 5:02:01 GGGGGAGCGGCCGCTGCGGACTCGGG

8719 8739 8045 9022 CDS product deoxyuridine triphosphatase 8719-8739 5:02:01 GGCCACGGCTGGTGGGCGGGG

8925 8947 8045 9022 CDS product deoxyuridine triphosphatase 8925-8947 6:03:01 CCAACCGCCAGGGGGCCGCCTCC

9057 9090 8970 9260 CDS product envelope glycoprotein N 9057-9090 6:03:01 GGCGCGAGGGGGCAATGGACTTTTGGAGCGCAGG

9238 9255 8970 9260 CDS product envelope glycoprotein N 9238-9255 4:01:01 GGAGTCGCGGGGGCGGGG

9576 9599 9384 10160 CDS product tegument protein VP22 9576-9599 6:03:01 CCGTCCAGCCCGCCGCCCCGCGGCC

9638 9659 9384 10160 CDS product tegument protein VP22 9638-9659 6:03:01 CCCGCCGCCGCGCCGGCCCGCC

9670 9690 9384 10160 CDS product tegument protein VP22 9670-9690 4:01:01 CCGGGCGTCCTCGCGCCCGCC

9699 9727 9384 10160 CDS product tegument protein VP22 9699-9727 8:05:02 CCGCCGACCCGCCCGTCCTCCGGCCAGCC

9734 9755 9384 10160 CDS product tegument protein VP22 9734-9755 4:01:01 GGGTCCTCCGGCGGCGCCGGGG

9762 9793 9384 10160 CDS product tegument protein VP22 9762-9793 6:03:01
CCGTCGGTCCACCTCGACCTCGCGCGCCCCCC
10077 10099 9384 10160 CDS product tegument protein VP22 10077-10099 4:01:01 CCGCGCGGGCCCAGCGCCCCGCC
10448 10474 10344 11792 CDS product transactivating tegument protein VP16 10448-10474 6:03:01
CCGCCCCCGCGCCCGGCTCCCCTGGCC
10533 10556 10344 11792 CDS product transactivating tegument protein VP16 10533-10556 5:02:01
GGGCCATGGAGCGGTGGAACGAGG
10654 10679 10344 11792 CDS product transactivating tegument protein VP16 10654-10679 4:01:01
GGTGCCTGGGGACGCGGAGCGCTTGG
10699 10723 10344 11792 CDS product transactivating tegument protein VP16 10699-10723 6:03:01
CCAGCCGCTTCCCGCACCGCCGGCC
10720 10763 10344 11792 CDS product transactivating tegument protein VP16 10720-10763 8:05:02
GGCCTCGGAGGAGGGCCTCCCGGAGTATGTGGCCGGCGTACAGG
10858 10898 10344 11792 CDS product transactivating tegument protein VP16 10858-10898 8:05:02
GGCGGCGCGTGGCCGAGGCGCGGCGGGCGCGGGCGCCCAGG
10911 10930 10344 11792 CDS product transactivating tegument protein VP16 10911-10930 5:02:01
GGCAGCTGGTGGCGGCGCGG
10942 10964 10344 11792 CDS product transactivating tegument protein VP16 10942-10964 5:02:01
GGCGAGCCGGCTGGCGCGGCTGG
11088 11104 10344 11792 CDS product transactivating tegument protein VP16 11088-11104 4:01:01
CCTGCCTGTTCCACCCC
11120 11170 10344 11792 CDS product transactivating tegument protein VP16 11120-11170 10:07:02
GGCGTCGTGGCGCTGGAGGACGGCTTCTTGGACGCGGCGGAGCTGCGGCGG

11206 11252 10344 11792 CDS product transactivating tegument protein VP16 11206-11252 8:05:02
GGTCCGCGCGGGGCTGGTCGAGGTTGAAGTGGGGCCTCTGGTGGAGG

11335 11363 10344 11792 CDS product transactivating tegument protein VP16 11335-11363 7:04:01
GGCCGGCGGGCGGCTGGCGCCGGAGCGGG

11519 11557 10344 11792 CDS product transactivating tegument protein VP16 11519-11557 7:04:01
CCTTCAGCCAAGGGCGCCGCCCCGGCCGAGTTTGCCGCC

11628 11670 10344 11792 CDS product transactivating tegument protein VP16 11628-11670 7:04:01
CCTTGGCCTTAGCCGAGCCCGCGGCAGCCCTGGCCCCGGCCCC

11682 11730 10344 11792 CDS product transactivating tegument protein VP16 11682-11730 8:05:02
CCCCAGCCGAGCCCGCGGCGGCCGTCGCCGGGCCAAGCCCGGCAAACCC

11986 12009 11963 14182 CDS product tegument protein VP13/14 11986-12009 5:02:01 CCTGAGCGCCGCCGCGCCGTCTCC

12018 12049 11963 14182 CDS product tegument protein VP13/14 12018-12049 5:02:01
CCGCACGCACCCGTTCCAGCGCCCCTCTGCCC

12100 12140 11963 14182 CDS product tegument protein VP13/14 12100-12140 7:04:01
CCGCGGGTCCGGCGCCCGCGGCCGTACTTCCAGCGGCCCCC

12502 12515 11963 14182 CDS product tegument protein VP13/14 12502-12515 5:02:01 GGCGGCGGGGGCGG

12645 12688 11963 14182 CDS product tegument protein VP13/14 12645-12688 10:07:02
GGAGGCGGCCGCTGCGGCTGCGGGGGAGAGGAGCGTGGTGGAGG

12740 12762 11963 14182 CDS product tegument protein VP13/14 12740-12762 5:02:01 GGATTTTGGAGGGCCGGGTGCGG

12842 12863 11963 14182 CDS product tegument protein VP13/14 12842-12863 4:01:01 GGCGCGCTTGGGAGATGGCCGG

12893 12910 11963 14182 CDS product tegument protein VP13/14 12893-12910 4:01:01 GGAGCACGGTGGACCCGG

12948 12975 11963 14182 CDS product tegument protein VP13/14 12948-12975 5:02:01
GGGCACCCGGCCCCGGCTAGTGTGGCGG

13010 13025 11963 14182 CDS product tegument protein VP13/14 13010-13025 5:02:01 GGCTGGGGGTGGGGGG
13129 13147 11963 14182 CDS product tegument protein VP13/14 13129-13147 4:01:01 GGCCGGGGCGCGCGGGCGG
13150 13169 11963 14182 CDS product tegument protein VP13/14 13150-13169 5:02:01 CCGCGCCGCCTGCCGCGGCC
13166 13186 11963 14182 CDS product tegument protein VP13/14 13166-13186 5:02:01 GGCCGGCGGTTGGGCTGCTGG
13587 13603 11963 14182 CDS product tegument protein VP13/14 13587-13603 4:01:01 GGAGACGGTGGACTCGG
13604 13621 11963 14182 CDS product tegument protein VP13/14 13604-13621 4:01:01 CCACCTTCCAAGAGGCCC
13819 13842 11963 14182 CDS product tegument protein VP13/14 13819-13842 5:02:01 GGCGCAGCGGTGTACGGGGGCCGG
13908 13942 11963 14182 CDS product tegument protein VP13/14 13908-13942 6:03:01
GGTGCGCCCGGTGTTCCTGGTGGAGTTCCGGGAGG
13998 14020 11963 14182 CDS product tegument protein VP13/14 13998-14020 5:02:01 GGCCGGCAAGCGGCGGGTCATGG
14074 14094 11963 14182 CDS product tegument protein VP13/14 14074-14094 4:01:01 GGTTCGCGGCCGGTGCTTGGG
14103 14125 11963 14182 CDS product tegument protein VP13/14 14103-14125 5:02:01 GGACATCGCGGAGGCGCTGGCGG
14392 14414 14314 16560 CDS product tegument protein VP11/12 14392-14414 4:01:01 CCACGGCCGCGGCCGTGGTGGCC
14396 14424 14314 16560 CDS product tegument protein VP11/12 14396-14424 6:03:01
GGCCGCGGCCGTGGTGGCCTTGCAGGAGG
14507 14532 14314 16560 CDS product tegument protein VP11/12 14507-14532 5:02:01
GGAGGCGCTGGTTCTGTCGGACTCGG
14584 14603 14314 16560 CDS product tegument protein VP11/12 14584-14603 4:01:01 GGGTGCTGGCGGGCGCGCGG
14691 14721 14314 16560 CDS product tegument protein VP11/12 14691-14721 7:04:01
GGCGCGGCCGGCGGCGCGGCGATGGAGCAGG
15039 15058 14314 16560 CDS product tegument protein VP11/12 15039-15058 4:01:01 CCGCGCGAGCCGCGGCCGCC
15134 15156 14314 16560 CDS product tegument protein VP11/12 15134-15156 5:02:01 GGCCCTGACGGCGGCGGTGCTGG
15259 15282 14314 16560 CDS product tegument protein VP11/12 15259-15282 5:02:01 CCGTACACCACCACCTGCAGTACC

15402 15419 14314 16560 CDS product tegument protein VP11/12 15402-15419 4:01:01 CCGACCATGACTTCCGCC

15424 15455 14314 16560 CDS product tegument protein VP11/12 15424-15455 6:03:01 GGGCGGCCCTGGAGCGCGGCACGGCGAGCTGG

15535 15567 14314 16560 CDS product tegument protein VP11/12 15535-15567 6:03:01 GGCTCGGCGGGAGCCGCGGGCTGCTGGCGCCGG

15579 15604 14314 16560 CDS product tegument protein VP11/12 15579-15604 5:02:01 CCGTCGGGCCGCGGCCGCCGCTGCCC

15616 15661 14314 16560 CDS product tegument protein VP11/12 15616-15661 10:07:02 CCGCGCGCCCCTTGCCCAGCCTGCCGGCCGCGGCCGCCGCCCCTCC

15662 15688 14314 16560 CDS product tegument protein VP11/12 15662-15688 5:02:01 GGGCTCGGGCTCGGGCTCGGGCTCGGG

15817 15831 14314 16560 CDS product tegument protein VP11/12 15817-15831 5:02:01 GGCGGGAGGCGGCGG

15929 15968 14314 16560 CDS product tegument protein VP11/12 15929-15968 7:04:01 CCCCATGCCTGCCCCGCCGCCCCGCGGTGGCCGCGGCGCC

15990 16028 14314 16560 CDS product tegument protein VP11/12 15990-16028 7:04:01 CCCATGCCTGCCCCGCCGCCCCGCGGCGGCCGCGGCGCC

16049 16088 14314 16560 CDS product tegument protein VP11/12 16049-16088 7:04:01 CCCCATGCCTGCCCCGCCGCCCCGCGGCGGCCGCGGCGCC

16145 16164 14314 16560 CDS product tegument protein VP11/12 16145-16164 4:01:01 GGACGAGGGCAAGGACGAGG

16177 16208 14314 16560 CDS product tegument protein VP11/12 16177-16208 5:02:01 CCGGGGCGACCGCCCCGTTGGCCGCGGACCCC

16244 16314 14314 16560 CDS product tegument protein VP11/12 16244-16314 12:09:03 CCTCCGCGTCCCGGCCTCGGCCCCGGCCCCGGCCCCGGCCCCGTGCACCGAGCGCCCGGGTAATCCAACCC

16256 16281 14314 16560 CDS product tegument protein VP11/12 16256-16281 5:02:01
GGCCTCGGCCCCGGCCCCGGCCCCGG
16334 16368 14314 16560 CDS product tegument protein VP11/12 16334-16368 5:02:01
CCCTCGGCTCCGCGCCCTAGACGACCGGCACGGCC
16360 16390 14314 16560 CDS product tegument protein VP11/12 16360-16390 7:04:01
GGCACGGCCTGGAGGCGCTGGCGGCTGCCGG
16387 16416 14314 16560 CDS product tegument protein VP11/12 16387-16416 5:02:01
CCGGTGCCGCTCACACCGCGCGCCACAACC
16528 16545 14314 16560 CDS product tegument protein VP11/12 16528-16545 4:01:01 GGCCTGAGGTGGAGAGGG
16820 16853 16683 18209 CDS product envelope glycoprotein C 16820-16853 6:03:01
GGCGGTCGCGGAGAACTCGGGCAGCGGTGCCGGG
17078 17098 16683 18209 CDS product envelope glycoprotein C 17078-17098 5:02:01 GGGCGGGCGGTAAACGGCCGG
17164 17185 16683 18209 CDS product envelope glycoprotein C 17164-17185 6:03:01 GGTTGGGCGGGTGGGCGGTTGG
18446 18472 18388 19524 CDS product envelope protein UL43 18446-18472 5:02:01 CCTTGGCCACGTGCGCCGCGGCCAGCC
18481 18502 18388 19524 CDS product envelope protein UL43 18481-18502 4:01:01 CCGCGCCCGCAGCGCCGGACCC
18512 18540 18388 19524 CDS product envelope protein UL43 18512-18540 5:02:01
CCACCAGCGTGCCCGCGGCTGCCGAGGCC
18556 18569 18388 19524 CDS product envelope protein UL43 18556-18569 4:01:01 CCGCCGTCCGCGCC
18726 18749 18388 19524 CDS product envelope protein UL43 18726-18749 5:02:01 CCAGCGCCCACCGCCCTCGAGCCC
18760 18783 18388 19524 CDS product envelope protein UL43 18760-18783 5:02:01 CCCGGGGCCCAGAGCGCCGCCGCC
18786 18799 18388 19524 CDS product envelope protein UL43 18786-18799 4:01:01 GGCTAAGGCGGCGG
18908 18934 18388 19524 CDS product envelope protein UL43 18908-18934 5:02:01 CCAGAGTCCGCAGCGCCGCGTACCGCC
18959 18978 18388 19524 CDS product envelope protein UL43 18959-18978 4:01:01 CCAGCGCCGCCGCGGCGTCC

18992 19061 18388 19524 CDS product envelope protein UL43 18992-19061 11:08:02
CCAGCGCGGCCGCGGCGCCCGCGGCCGCCAGCGCCTCGGGCCGCGCGCCCCAGCGCCCGGCCGAAAGGCC
19209 19237 18388 19524 CDS product envelope protein UL43 19209-19237 6:03:01
GGCGGGGGCGGGGTCTGTGGCCCAGACGG
19397 19418 18388 19524 CDS product envelope protein UL43 19397-19418 5:02:01 CCAGGGCCGCCGCCATCAAGCC
19429 19449 18388 19524 CDS product envelope protein UL43 19429-19449 4:01:01 CCCATGGCCACCAGCGCGCCC
19487 19507 18388 19524 CDS product envelope protein UL43 19487-19507 4:01:01 CCCCCGCGCAGCCGCTCACCC
19609 19635 19597 20823 CDS product DNA polymerase processivity subunit 19609-19635 6:03:01
CCCCCCTCCCCGCCCCGATCCGGGCCC
19642 19670 19597 20823 CDS product DNA polymerase processivity subunit 19642-19670 5:02:01
GGGCCCGGGGCGCCGGCGTCGGCGCGCGG
19750 19769 19597 20823 CDS product DNA polymerase processivity subunit 19750-19769 5:02:01
GGTTGGCCGGACGCGGCGGG
19779 19801 19597 20823 CDS product DNA polymerase processivity subunit 19779-19801 5:02:01
GGGAAGGCGGGGTGCGGGCTCGG
19821 19848 19597 20823 CDS product DNA polymerase processivity subunit 19821-19848 9:06:02
GGCGGAGGCGGTGGCGGTGGCGGGGCGG
19902 19923 19597 20823 CDS product DNA polymerase processivity subunit 19902-19923 5:02:01
GGCGGCGCTGGCGGGCGCGAGG
19945 19962 19597 20823 CDS product DNA polymerase processivity subunit 19945-19962 5:02:01
CCGCCGACCTGCCGCACC
20054 20070 19597 20823 CDS product DNA polymerase processivity subunit 20054-20070 4:01:01
CCCTGCGGCCGCCGCCC

20184 20214 19597 20823 CDS product DNA polymerase processivity subunit 20184-20214 5:02:01 GGACTCGGCGCTCAGGCAACGGGCGCGCCGG

20540 20560 19597 20823 CDS product DNA polymerase processivity subunit 20540-20560 5:02:01 GGGCGGGGGCCCACTGGAAGG

20667 20692 19597 20823 CDS product DNA polymerase processivity subunit 20667-20692 4:01:01 GGCGCCAAAGGGGCTCAGCAGGGCGG

20757 20779 19597 20823 CDS product DNA polymerase processivity subunit 20757-20779 5:02:01 GGACAGACCGGCGGTGAGGCGGG

21125 21140 21068 22447 CDS product tegument host shutoff protein 21125-21140 4:01:01 CCATCACCACGCCGCC

21312 21336 21068 22447 CDS product tegument host shutoff protein 21312-21336 4:01:01 GGACCGCGGCATCCACGGGGACCGG

21315 21342 21068 22447 CDS product tegument host shutoff protein 21315-21342 4:01:01 CCGCGGCATCCACGGGGACCGGCGCGCC

21388 21411 21068 22447 CDS product tegument host shutoff protein 21388-21411 5:02:01 GGCGGCTCGGGCCGCCTCGGGCGG

21435 21452 21068 22447 CDS product tegument host shutoff protein 21435-21452 4:01:01 GGAGGACGAGGTGCTGGG

21504 21520 21068 22447 CDS product tegument host shutoff protein 21504-21520 4:01:01 GGAGGCAACGGCGAAGG

21540 21556 21068 22447 CDS product tegument host shutoff protein 21540-21556 4:01:01 GGCGGGGGCCGCGCGGG

21876 21903 21068 22447 CDS product tegument host shutoff protein 21876-21903 6:03:01 GGTGCAGCAGGTGGTGCGGGGGCTGCGG

21911 21933 21068 22447 CDS product tegument host shutoff protein 21911-21933 4:01:01 CCGAGGCCGAGCCCGCGACTACC

21976 22005 21068 22447 CDS product tegument host shutoff protein 21976-22005 5:02:01

GGTCGTCCGGGCGCTGGGCCGCGGCGCCGG
21994 22014 21068 22447 CDS product tegument host shutoff protein 21994-22014 4:01:01
CCGCGGCGCCGGTTGCCGCCC
22356 22377 21068 22447 CDS product tegument host shutoff protein 22356-22377 5:02:01
CCCCACTCCACCCCCATTTGCC
22397 22431 21068 22447 CDS product tegument host shutoff protein 22397-22431 5:02:01
GGGACGAGGCCCCCGCGGGGCCGGGGTCGCGACGG
22642 22674 22563 23507 CDS product ribonucleotide reductase subunit 2 22642-22674 6:03:01
GGGCCAAAGGAAAATCGGTCCCAGGAGGCGGGG
22837 22849 22563 23507 CDS product ribonucleotide reductase subunit 2 22837-22849 4:01:01 GGGCCGGCGGCGG
22915 22937 22563 23507 CDS product ribonucleotide reductase subunit 2 22915-22937 4:01:01
GGCTGATGAGGTCGTTGGTTTGG
22954 22974 22563 23507 CDS product ribonucleotide reductase subunit 2 22954-22974 4:01:01
GGTTGTGGGTGCGCAGGTAGG
23041 23117 22563 23507 CDS product ribonucleotide reductase subunit 2 23041-23117 13:10:03
CCACCGACTCTGCCGCGGCCACGCGCCGCTCGAGCCAGTCCACCTTGCGCCGGACCGCCGGGTCGCCGAGGGCGCCC
23284 23303 22563 23507 CDS product ribonucleotide reductase subunit 2 23284-23303 4:01:01
GGTCATCGGCGGCCGAGAGG
23340 23360 22563 23507 CDS product ribonucleotide reductase subunit 2 23340-23360 4:01:01
CCGCTGAGCCGCGCCACGTCC
23485 23504 22563 23507 CDS product ribonucleotide reductase subunit 2 23485-23504 5:02:01
CCGCGTCCGCCGCCTCGGCC
23730 23768 23526 25889 CDS product ribonucleotide reductase subunit 1 23730-23768 6:03:01

GGGGCGCGGTCGGCGCAGAGGTCGATCAGGCGCTCTTGG
23795 23819 23526 25889 CDS product ribonucleotide reductase subunit 1 23795-23819 4:01:01
GGCCAGCGGATGCCCGGGCGCGAGG
23997 24017 23526 25889 CDS product ribonucleotide reductase subunit 1 23997-24017 4:01:01
CCCTCGCTGCCCTCCGTCACC
24026 24045 23526 25889 CDS product ribonucleotide reductase subunit 1 24026-24045 4:01:01
GGACACGGTCGGCATAAGGG
24218 24255 23526 25889 CDS product ribonucleotide reductase subunit 1 24218-24255 7:04:01
GGCGCTGGTGGCCATGACGGCCAGGAGCAGGCGCTCGG
24428 24459 23526 25889 CDS product ribonucleotide reductase subunit 1 24428-24459 6:03:01
GGCGGCCGTGGCGGCCGCCGTGGACAGAGCGG
24760 24783 23526 25889 CDS product ribonucleotide reductase subunit 1 24760-24783 4:01:01
CCGTAAAGGCCGGGCCGTGCAGCC
24835 24860 23526 25889 CDS product ribonucleotide reductase subunit 1 24835-24860 5:02:01
GGCCTTCGAGGTGGGCGAGGTAGCGG
25168 25181 23526 25889 CDS product ribonucleotide reductase subunit 1 25168-25181 4:01:01 CCTCCACGGCCTCC
25352 25377 23526 25889 CDS product ribonucleotide reductase subunit 1 25352-25377 4:01:01
CCCAAAGGCCGGCTCCCGCATCGTCC
25382 25395 23526 25889 CDS product ribonucleotide reductase subunit 1 25382-25395 4:01:01 GGCGGCGGTCGCGG
25769 25791 23526 25889 CDS product ribonucleotide reductase subunit 1 25769-25791 7:04:01
GGCGGCGGGCGGCGGCGGCACGG
25794 25817 23526 25889 CDS product ribonucleotide reductase subunit 1 25794-25817 4:01:01
CCGCAGCCCAGCTGCGCCCATTCC

26244 26275 26231 27655 CDS product capsid triplex subunit 1 26244-26275 6:03:01 CCACCCAGTCCCCGGTTCCCCACACCAAGCCC
26470 26491 26231 27655 CDS product capsid triplex subunit 1 26470-26491 4:01:01 GGGGCAGCGGTAGGCCGCGCGG
26552 26564 26231 27655 CDS product capsid triplex subunit 1 26552-26564 4:01:01 CCCGCCGTCCCCC
26885 26905 26231 27655 CDS product capsid triplex subunit 1 26885-26905 4:01:01 CCGCCCAGGCACCCGAGTGCC
26912 26948 26231 27655 CDS product capsid triplex subunit 1 26912-26948 6:03:01 GGGAACGCGCGGCAGCGGATCATGGCCTGCAGGCAGG
26979 27004 26231 27655 CDS product capsid triplex subunit 1 26979-27004 5:02:01 GGCGGTCGCCGGAGTTGGAAATGAGG
27046 27059 26231 27655 CDS product capsid triplex subunit 1 27046-27059 4:01:01 GGCGGCGCAGGCGG
27322 27348 26231 27655 CDS product capsid triplex subunit 1 27322-27348 6:03:01 GGGCGAGAGGACGGCGGTGCCGGCGGG
27458 27499 26231 27655 CDS product capsid triplex subunit 1 27458-27499 7:04:01 CCCCGGGGTGCCCGCCCGCGAGCGCCGCCAACCGCAGGCGCC
27517 27539 26231 27655 CDS product capsid triplex subunit 1 27517-27539 5:02:01 GGCTTCGGCGGCAGAGGCCTCGG
27534 27547 26231 27655 CDS product capsid triplex subunit 1 27534-27547 4:01:01 CCTCGGCCGCCGCC
27550 27562 26231 27655 CDS product capsid triplex subunit 1 27550-27562 4:01:01 GGCGGCGGCGGGG
27575 27592 26231 27655 CDS product capsid triplex subunit 1 27575-27592 5:02:01 GGCGGCGCGGCGGCTGGG
27913 27939 27871 30945 CDS product tegument protein UL37 27913-27939 6:03:01 GGTTGGATGGCGAGGTCGCCGGCCCGG
27950 27972 27871 30945 CDS product tegument protein UL37 27950-27972 5:02:01 GGAGGCCAGGGCGGCCGTCTCGG
27977 27995 27871 30945 CDS product tegument protein UL37 27977-27995 4:01:01 CCTCCTCGCGTCCGGGCCC
28160 28179 27871 30945 CDS product tegument protein UL37 28160-28179 4:01:01 GGCCGGCGTTCGGGACCTGG
28199 28221 27871 30945 CDS product tegument protein UL37 28199-28221 4:01:01 GGCGCCGTTGGGTTACCTGGCGG

| | | | | | | |
|---|---|---|---|---|---|---|
| 28214 | 28238 | 27871 | 30945 | CDS product tegument protein UL37 | 28214-28238 4:01:01 | CCTGGCGGCCGCGCGCCTGCGCGCC |
| 28241 | 28257 | 27871 | 30945 | CDS product tegument protein UL37 | 28241-28257 4:01:01 | GGCGGCGTTCGGCCCGG |
| 28274 | 28287 | 27871 | 30945 | CDS product tegument protein UL37 | 28274-28287 4:01:01 | GGTGGTGGAGTGGG |
| 28319 | 28345 | 27871 | 30945 | CDS product tegument protein UL37 | 28319-28345 4:01:01 | GGACGCGGCGTGCGTGGGTGTGTTAGG |
| 28346 | 28369 | 27871 | 30945 | CDS product tegument protein UL37 | 28346-28369 4:01:01 | CCCCGACCCTGCGTGCCGCTCGCC |
| 28513 | 28534 | 27871 | 30945 | CDS product tegument protein UL37 | 28513-28534 4:01:01 | CCGCGGACGCCGCGGCGCCGCC |
| 28598 | 28632 | 27871 | 30945 | CDS product tegument protein UL37 | 28598-28632 6:03:01 | GGACACGGCGCCGGGGCTGGCTGAGGCGTTTGCGG |
| 28721 | 28740 | 27871 | 30945 | CDS product tegument protein UL37 | 28721-28740 4:01:01 | GGCGGCGCTGGCCTACCGGG |
| 28854 | 28868 | 27871 | 30945 | CDS product tegument protein UL37 | 28854-28868 4:01:01 | GGCGCGGTGGTGCGG |
| 28963 | 28979 | 27871 | 30945 | CDS product tegument protein UL37 | 28963-28979 4:01:01 | CCACGCCAGACCAAGCC |
| 28982 | 29003 | 27871 | 30945 | CDS product tegument protein UL37 | 28982-29003 4:01:01 | GGAGACGCTGGCCGCGGCGGGG |
| 29093 | 29122 | 27871 | 30945 | CDS product tegument protein UL37 | 29093-29122 8:05:02 | GGCGGTGGGCTGCGTGGCGGTGGCGGGCGG |
| 29321 | 29349 | 27871 | 30945 | CDS product tegument protein UL37 | 29321-29349 6:03:01 | GGCGCTGGCGGCAGAGGCCGCGGCCGCGG |
| 29484 | 29513 | 27871 | 30945 | CDS product tegument protein UL37 | 29484-29513 6:03:01 | CCGCCCGCGGCCGAAGCGCCGCCCGCGGCC |
| 29531 | 29550 | 27871 | 30945 | CDS product tegument protein UL37 | 29531-29550 4:01:01 | GGCCGCGGGCGGCGCTTCGG |
| 29627 | 29657 | 27871 | 30945 | CDS product tegument protein UL37 | 29627-29657 6:03:01 | CCCGGCCTTTGCGCCCCACTTCGCCGCCGCC |
| 29666 | 29685 | 27871 | 30945 | CDS product tegument protein UL37 | 29666-29685 4:01:01 | GGACGTGCTGGTGGCTGCGG |
| 29754 | 29769 | 27871 | 30945 | CDS product tegument protein UL37 | 29754-29769 4:01:01 | GGCGCGGGAGCGGTGG |

| | | | | | | | |
|---|---|---|---|---|---|---|---|
| 29801 | 29822 | 27871 | 30945 | CDS product tegument protein UL37 | 29801-29822 | 5:02:01 | GGCCCTGGCGGCCGGGCTCTGG |
| 29945 | 29965 | 27871 | 30945 | CDS product tegument protein UL37 | 29945-29965 | 4:01:01 | CCCGCCTGTGCCCGCGCGCCC |
| 30093 | 30112 | 27871 | 30945 | CDS product tegument protein UL37 | 30093-30112 | 5:02:01 | GGCGCGGTGGCGCGGCTGGG |
| 30197 | 30238 | 27871 | 30945 | CDS product tegument protein UL37 | 30197-30238 | 7:04:01 | GGGTGCGTGGCGCTCGGCGGACCTGGCCGACGCGGTGCGCGG |
| 30247 | 30269 | 27871 | 30945 | CDS product tegument protein UL37 | 30247-30269 | 4:01:01 | CCGAAGCCGCGCGCGCCCGGGCC |
| 30363 | 30375 | 27871 | 30945 | CDS product tegument protein UL37 | 30363-30375 | 4:01:01 | GGCGGCGGGCTGG |
| 30398 | 30451 | 27871 | 30945 | CDS product tegument protein UL37 | 30398-30451 | 9:06:02 | GGCGGGCCACTCGGCGCTGGTGCGGGCGCAGACGGCGCTGGCGCTGGCCGCCGG |
| 30461 | 30482 | 27871 | 30945 | CDS product tegument protein UL37 | 30461-30482 | 5:02:01 | GGCGGGCGCGGAGGCGCCGGGG |
| 30592 | 30624 | 27871 | 30945 | CDS product tegument protein UL37 | 30592-30624 | 6:03:01 | GGCTCCGCGGCGTCTGGGACGAGGTCCAGGAGG |
| 30686 | 30753 | 27871 | 30945 | CDS product tegument protein UL37 | 30686-30753 | 11:08:02 | GGCGGTGCTGGCGCTGTTGGAGGGCTACCCGGAGGTTCGGGGAGACGAGGGCAGCCCGGCGCTCCTGG |
| 30764 | 30784 | 27871 | 30945 | CDS product tegument protein UL37 | 30764-30784 | 4:01:01 | GGACGTGGCCGACTGGGCGGG |
| 30832 | 30848 | 27871 | 30945 | CDS product tegument protein UL37 | 30832-30848 | 4:01:01 | CCGCCGCGTCTGCCGCC |
| 30851 | 30875 | 27871 | 30945 | CDS product tegument protein UL37 | 30851-30875 | 5:02:01 | GGCAAACACGGCGGCGGGGCCGTGG |
| 30919 | 30939 | 27871 | 30945 | CDS product tegument protein UL37 | 30919-30939 | 4:01:01 | CCAGTCGCCCGGCCGCAGCCC |
| 31125 | 31150 | 31040 | 40651 | CDS product large tegument protein | 31125-31150 | 5:02:01 | GGCGCTGGGGCCGGGCAGCGCGGTGG |
| 31181 | 31199 | 31040 | 40651 | CDS product large tegument protein | 31181-31199 | 4:01:01 | GGCTGGCCTTCGCGGGCGG |
| 31305 | 31327 | 31040 | 40651 | CDS product large tegument protein | 31305-31327 | 5:02:01 | CCTGCCAAACCGCATCACCGACC |
| 31329 | 31347 | 31040 | 40651 | CDS product large tegument protein | 31329-31347 | 4:01:01 | GGCGGGCGCGGGGAGCGGG |
| 31479 | 31511 | 31040 | 40651 | CDS product large tegument protein | 31479-31511 | 5:02:01 | |

GGCGCTGGTGATCGTGGGCGCCATGGGCGTCGG
31803 31839 31040 40651 CDS product large tegument protein 31803-31839 6:03:01
CCCGCGCGCGCCGGCCAACACGGCCTGCCTTACGGCC
31836 31861 31040 40651 CDS product large tegument protein 31836-31861 7:04:01 GGCCGTGGCGGTGGGCGGCGCGGGGG
31950 31983 31040 40651 CDS product large tegument protein 31950-31983 6:03:01
GGCCGCGGCCGCGGCTCGCGCGGCGGGTAAGCGG
31997 32016 31040 40651 CDS product large tegument protein 31997-32016 5:02:01 GGCGGCGGCGGGCCCCGTGG
32009 32041 31040 40651 CDS product large tegument protein 32009-32041 5:02:01
CCCCGTGGACGCCGCCCTCGAGCCGCGAGGACC
32088 32152 31040 40651 CDS product large tegument protein 32088-32152 13:10:03
GGTCCGGGGCGCGGAGACGGCGGGGGCCGGGGAAGATTGGGGGGAGGGGGGAAACGCGGCTGCGG
32153 32165 31040 40651 CDS product large tegument protein 32153-32165 4:01:01 CCCGCCCGCCGCC
32177 32196 31040 40651 CDS product large tegument protein 32177-32196 4:01:01 CCGCCGCGCCCGCGCACGCC
32202 32222 31040 40651 CDS product large tegument protein 32202-32222 4:01:01 GGCGTGCGCGGCGGAGCTTGG
32232 32254 31040 40651 CDS product large tegument protein 32232-32254 5:02:01 GGCGGAGGTTACCGCGGCCTTGG
32243 32263 31040 40651 CDS product large tegument protein 32243-32263 4:01:01 CCGCGGCCTTGGACGCCGTCC
32441 32459 31040 40651 CDS product large tegument protein 32441-32459 4:01:01 CCCCGTCGGCCGTCCAGCC
32467 32482 31040 40651 CDS product large tegument protein 32467-32482 4:01:01 GGGGCGGTGGCCCAGG
32527 32548 31040 40651 CDS product large tegument protein 32527-32548 4:01:01 GGGATGGTGCTCGAGGGGCTGG
32592 32611 31040 40651 CDS product large tegument protein 32592-32611 4:01:01 GGCGCTGGGCGACCTGGCGG
32676 32717 31040 40651 CDS product large tegument protein 32676-32717 9:06:02
GGACGAGCTGGAGGGCGAGGTGGACGGCGCCGGCGGCGCGGG
32763 32779 31040 40651 CDS product large tegument protein 32763-32779 4:01:01 CCTCTCGGCCTCCGGCC

32769 32784 31040 40651 CDS product large tegument protein 32769-32784 4:01:01 GGCCTCCGGCCGGCGG
32870 32889 31040 40651 CDS product large tegument protein 32870-32889 4:01:01 CCCGCGTGGCCGCCGAGACC
32913 32929 31040 40651 CDS product large tegument protein 32913-32929 4:01:01 GGCGCTGGAGGGCACGG
32949 32975 31040 40651 CDS product large tegument protein 32949-32975 5:02:01 CCTGGACGCCGTCCCGCCCGACGACCC
32982 33016 31040 40651 CDS product large tegument protein 32982-33016 6:03:01
GGTGCCCGCGGACAGCGCGGTGGCCGACTTGGCGG
33059 33075 31040 40651 CDS product large tegument protein 33059-33075 5:02:01 CCGCCGCCCTCTCCGCC
33146 33160 31040 40651 CDS product large tegument protein 33146-33160 4:01:01 CCATCCTGGCCGACC
33240 33263 31040 40651 CDS product large tegument protein 33240-33263 6:03:01 GGCGGCGCGGGTGGCTCGGCTTGG
33266 33303 31040 40651 CDS product large tegument protein 33266-33303 7:04:01
CCGCGCCGGGCGCCGCGGCGCCTCCGCTGCCGCGGTCC
33367 33385 31040 40651 CDS product large tegument protein 33367-33385 4:01:01 GGCCTCGCGGCCTGGGTGG
33399 33413 31040 40651 CDS product large tegument protein 33399-33413 4:01:01 GGCGCAGGCGGAAGG
33469 33508 31040 40651 CDS product large tegument protein 33469-33508 6:03:01
GGGCGCTCGGCGTCGCGGGCGTCGCTGGAGGCGAGCATGG
33531 33554 31040 40651 CDS product large tegument protein 33531-33554 7:04:01 GGCGGCGGCGCGGGCGGCGGCGGG
33564 33581 31040 40651 CDS product large tegument protein 33564-33581 4:01:01 GGAGGCGCCGGGCGCGGG
33648 33673 31040 40651 CDS product large tegument protein 33648-33673 5:02:01 GGACGCCCTGGCGACGGGCGCGGCGG
33699 33766 31040 40651 CDS product large tegument protein 33699-33766 11:08:02
GGCCGCGGCCCGCGCGGGGCTAGAGGCGCAGGCGCTCGCGGCGCGGCGGCGCGTGGCCGAGCTGGGGG
33840 33861 31040 40651 CDS product large tegument protein 33840-33861 4:01:01 GGCGCCCGGCGCGCTGGAGCGG
34008 34034 31040 40651 CDS product large tegument protein 34008-34034 5:02:01 GGCGCCGGGCGCGCTGGCCGGGCTGGG
34068 34090 31040 40651 CDS product large tegument protein 34068-34090 4:01:01 GGGCCAGGCGATGGGCCCCGCGG

34107 34162 31040 40651 CDS product large tegument protein 34107-34162 9:06:02
GGGGCACGCGGAGCGCGTGGCGGCCGCCGTGGCTCGCGCGGCGGCGGAGCCTGCGG
34156 34182 31040 40651 CDS product large tegument protein 34156-34182 5:02:01 CCTGCGGCGCCTGCCGCGGTGGCCGCC
34203 34229 31040 40651 CDS product large tegument protein 34203-34229 6:03:01 GGCGGCTGAGGCGCTGCGGGCCGGGGG
34306 34322 31040 40651 CDS product large tegument protein 34306-34322 4:01:01 GGCGCGCGGCGGCTGGG
34440 34465 31040 40651 CDS product large tegument protein 34440-34465 6:03:01 GGCAGACGCGGCGCTGGGGGCGGCGG
34478 34534 31040 40651 CDS product large tegument protein 34478-34534 12:09:03
GGGCCGCGGACGAGGCCGCGGTGGCCGCCGGCGGCGAGGATGCGGACGGCGGCGAGG
34545 34565 31040 40651 CDS product large tegument protein 34545-34565 4:01:01 GGCCGACGCGGACGGCGCCGG
34629 34650 31040 40651 CDS product large tegument protein 34629-34650 5:02:01 GGCCCGGGCGCTGGCGCGGCGG
34665 34686 31040 40651 CDS product large tegument protein 34665-34686 4:01:01 CCGGCGCCTGCGCGCCGAGGCC
34718 34741 31040 40651 CDS product large tegument protein 34718-34741 5:02:01 GGCAGGAGGAGCGCTGGCGCGAGG
34844 34879 31040 40651 CDS product large tegument protein 34844-34879 7:04:01
GGCCGCTGGCGCGGCAGGCCGACCGGGCGCTGGAGG
35157 35173 31040 40651 CDS product large tegument protein 35157-35173 4:01:01 GGAGGGCGACCTGGCGG
35213 35236 31040 40651 CDS product large tegument protein 35213-35236 5:02:01 GGGGGCACGCGGCCTTCGAGGAGG
35277 35299 31040 40651 CDS product large tegument protein 35277-35299 6:03:01 GGCGGCCGGCGGGCGCGCGGCGG
35496 35551 31040 40651 CDS product large tegument protein 35496-35551 10:07:02
GGCCGCGGCGCGAGAGGCGCTAGAGGCGGCAAAGGCGGCGCGCGCGGAGGCCACGG
35572 35596 31040 40651 CDS product large tegument protein 35572-35596 5:02:01 GGCGAGGTCGTGGCTGCCGAGGCGG
35618 35641 31040 40651 CDS product large tegument protein 35618-35641 4:01:01 CCGGGCTGGCCAACCTCAAGAACC
35688 35706 31040 40651 CDS product large tegument protein 35688-35706 4:01:01 CCGCGCCGCCTCGGCGGCC
35736 35784 31040 40651 CDS product large tegument protein 35736-35784 11:08:02

GGCGGCCGTGGAGGAGGCGCCGGAGCTGGACGTGGCCGCGGTGGAGTGG

35915 35959 31040 40651 CDS product large tegument protein 35915-35959 10:07:02 GGCGGCAGCTGGAGTCGGCGGCGGCGGCGTGGGACGGCGCGTGGG

35979 35994 31040 40651 CDS product large tegument protein 35979-35994 4:01:01 CCGCCGCGCGGCCGCC

36042 36066 31040 40651 CDS product large tegument protein 36042-36066 5:02:01 GGCGGCCGCGGGCGTGGTGCTGGGG

36112 36143 31040 40651 CDS product large tegument protein 36112-36143 6:03:01 GGCGCGCTGGACGCGCGGCTGGCGGAGCGCGG

36158 36180 31040 40651 CDS product large tegument protein 36158-36180 4:01:01 CCTTCCACGAAGCCGCGCGCGCC

36219 36235 31040 40651 CDS product large tegument protein 36219-36235 4:01:01 GGCGGTGCGCGGCGAGG

36287 36310 31040 40651 CDS product large tegument protein 36287-36310 5:02:01 CCGAGCTCCCGGCCTGGGCCGCCC

36526 36548 31040 40651 CDS product large tegument protein 36526-36548 4:01:01 GGGCGCGTGGCGGCCCTTCTGGG

36607 36627 31040 40651 CDS product large tegument protein 36607-36627 4:01:01 CCGCGCGCCTACCTGGACGCC

36739 36760 31040 40651 CDS product large tegument protein 36739-36760 6:03:01 GGCGTGGTGGAGGCGGCTTCGG

36875 36886 31040 40651 CDS product large tegument protein 36875-36886 4:01:01 GGGGGCTGGCGG

36987 37029 31040 40651 CDS product large tegument protein 36987-37029 7:04:01 GGCCGAGCGGGGGCTGCGGCCAGCGCGGGCGCCGGCGAGCGGG

37227 37252 31040 40651 CDS product large tegument protein 37227-37252 4:01:01 GGGAGAGCCCGGTGCGAGGCGCCCGG

37261 37289 31040 40651 CDS product large tegument protein 37261-37289 7:04:01 GGCGGCGGCGCGGACGACCCGGCGGACGG

37301 37314 31040 40651 CDS product large tegument protein 37301-37314 4:01:01 CCATCCGCGCCGCC

37329 37344 31040 40651 CDS product large tegument protein 37329-37344 4:01:01 CCGCCTTTCCCCGTCC

37590 37650 31040 40651 CDS product large tegument protein 37590-37650 12:09:03 GGGCGGCCCTTCGGAAGACGCGGCGGCGCTGGCCGCTGCGGCTGCGGCGGCCGGGGGCCGG

37764 37798 31040 40651 CDS product large tegument protein 37764-37798 5:02:01
GGGCGCGCCGGTTGTCGTGGCCATGGAGAACCCGG
37871 37887 31040 40651 CDS product large tegument protein 37871-37887 4:01:01 CCGACGCGCCTGCCGCC
37910 37927 31040 40651 CDS product large tegument protein 37910-37927 4:01:01 GGGGGGAGCGGCTGCTGG
38081 38093 31040 40651 CDS product large tegument protein 38081-38093 4:01:01 CCGCCGCGCCGCC
38173 38198 31040 40651 CDS product large tegument protein 38173-38198 6:03:01 CCCCCCGCGGCCAGCGCCGCCGACCC
38274 38298 31040 40651 CDS product large tegument protein 38274-38298 5:02:01 CCCCCCCGTGTTCCAGCACGCCACC
38321 38345 31040 40651 CDS product large tegument protein 38321-38345 7:04:01 GGGCGGCGGCAGGTGGCGGCAGCGG
38418 38446 31040 40651 CDS product large tegument protein 38418-38446 6:03:01
GGCACCGGCACTGGCACCGGCTTCGGCGG
38505 38517 31040 40651 CDS product large tegument protein 38505-38517 4:01:01 CCCGCCGGCCCCC
38532 38544 31040 40651 CDS product large tegument protein 38532-38544 4:01:01 CCCGCCGGCCCCC
38559 38571 31040 40651 CDS product large tegument protein 38559-38571 4:01:01 CCCGCCGGCCCCC
38617 38636 31040 40651 CDS product large tegument protein 38617-38636 4:01:01 GGAGCCGCGGAGGCTCCTGG
38684 38702 31040 40651 CDS product large tegument protein 38684-38702 4:01:01 CCATGCCCGCCGCGTTGCC
38712 38906 31040 40651 CDS product large tegument protein 38712-38906 44:41:11
CCCGCCGCCCGCAAGCCTGGTCTCCGCAGCGCCTCCAGTGCCTCCGGGGCCGCGCCTGCCGCCGGCCCCGCCATTGCCGCCGCCGGCCCCGCCATTGCCGCCGCCGGCCCCGCCATTGCCACCGCCGG
CCCCGCCATTGCCGCCGCCGGCCCCGCCATTGCCGCCGCCGGCCCCGCCATTGCCGCCGCCGGCCCC
38915 39020 31040 40651 CDS product large tegument protein 38915-39020 20:17:05
CCCCGGTTCCGGCCCCGCCATTGCCGCCGCCGGCCCTGACTCCGGCCCTGACTCCGGCCCCGACTCCGGCCCCGACTCCGGCCCCGCCATTGCCGCTGCCGGCCCC
39027 39058 31040 40651 CDS product large tegument protein 39027-39058 6:03:01
GGTTTTGGTTCCGGCCCCGGTTCCGGCTCCGG
39037 39131 31040 40651 CDS product large tegument protein 39037-39131 18:15:04

CCGGCCCCGGTTCCGGCTCCGGCCCCGATTCCGGCTCCGGCCCCGACTCCGGCTCCGGCCCCGACTCCGGCTCCGCCATTGCCGCCGCCGGCTCC

39244 39263 31040 40651 CDS product large tegument protein 39244-39263 4:01:01 CCCGCCAGCGTTCCGGCGCC

39325 39338 31040 40651 CDS product large tegument protein 39325-39338 4:01:01 CCGTCCAGACCGCC

39349 39392 31040 40651 CDS product large tegument protein 39349-39392 8:05:02 CCCGTGCCTGGCTTGCCCCTGCCGCCGAGCCGGGTCCAAGCACC

39403 39570 31040 40651 CDS product large tegument protein 39403-39570 32:29:08 CCCGCGCCGCCCCCCGCGCCAGAGCGGCCCGCGCCGCCCCCCGCGCCAGAGCGGCCCGCGCCGCCCCCCGCGCCAGAGCGGCCCGCGCCGCCCCCCGCGCCAGAGCGGCCCGCGCCGCCCCCCGCGCCAGAGCGGCCCGCGCCGCCCCCCGCGCCAGAGCGGCCCGCC

39618 39631 31040 40651 CDS product large tegument protein 39618-39631 4:01:01 GGTGGGCCAGGTGG

39673 39693 31040 40651 CDS product large tegument protein 39673-39693 4:01:01 CCTGGCGCGCCCGCCAGGCCC

39725 39744 31040 40651 CDS product large tegument protein 39725-39744 4:01:01 CCCGGGCCGTTGACCCTACC

39829 39847 31040 40651 CDS product large tegument protein 39829-39847 4:01:01 CCGCCTGGCCCCGCTCCCC

39856 39889 31040 40651 CDS product large tegument protein 39856-39889 7:04:01 CCCCCTGCGCCTTCGGCCCTGCCGGCGCCGCGCC

39916 40052 31040 40651 CDS product large tegument protein 39916-40052 25:22:06 CCGGCGCCCCCGATCGCGCCCCCGATCGCGCCCCCGATCGCGCCCCCGATCGCGCCCCCGATCGCGCCCCCGATCGCGCCCCCGATCGCGCCCCCGATCGCGCCCCCGATCGCGCCCCCCGCCATGCCGGCACCCCC

40082 40118 31040 40651 CDS product large tegument protein 40082-40118 8:05:02 CCGCGCCCCCAATCGCGCCCCCCGCCACGCCAGCGCC

40129 40148 31040 40651 CDS product large tegument protein 40129-40148 4:01:01 CCCCTTGCCGCGCCGGTGCC

40143 40165 31040 40651 CDS product large tegument protein 40143-40165 5:02:01 GGTGCCTCGGTTGGTAGGTGCGG

40324 40345 31040 40651 CDS product large tegument protein 40324-40345 5:02:01 CCCGCGCCCCCCAAGGTCCCCC

40358 40378 31040 40651 CDS product large tegument protein 40358-40378 4:01:01 CCGACGCCACCGATAGCTCCC

40380 40408 31040 40651 CDS product large tegument protein 40380-40408 6:03:01
GGACGGAAGCGGGCCGGAAACGGAAGAGG
40419 40442 31040 40651 CDS product large tegument protein 40419-40442 5:02:01 GGAAGCGGGGGGCGACGGCCCCGG
40831 40850 40812 41186 CDS product small capsid protein 40831-40850 5:02:01 CCGCCGCGCCAGCGTCCTCC
40879 40890 40812 41186 CDS product small capsid protein 40879-40890 4:01:01 GGGGGAGGAAGG
41030 41051 40812 41186 CDS product small capsid protein 41030-41051 4:01:01 CCGGGCCGCGCTCACCTGCTCC
41128 41162 40812 41186 CDS product small capsid protein 41128-41162 6:03:01
GGGTGTCGGGGGTGATGGTAGCCGGCGCACTAGGG
41357 41371 41231 42013 CDS product nuclear egress membrane protein 41357-41371 4:01:01 CCGGCGCCGCCGGCC
41548 41575 41231 42013 CDS product nuclear egress membrane protein 41548-41575 4:01:01
GGACATCATGGCGCGAAAGGGCCGTGGG
41766 41792 41231 42013 CDS product nuclear egress membrane protein 41766-41792 4:01:01
CCGTCTGCGCCCAGCTGGCCATTAGCC
41863 41892 41231 42013 CDS product nuclear egress membrane protein 41863-41892 6:03:01
GGGCGGGCGGCGCGGGTCCCAGGGCTGGGG
41966 41985 41231 42013 CDS product nuclear egress membrane protein 41966-41985 4:01:01 CCCTCGCCCTCGCCCTCGCC
42185 42198 42124 42450 CDS product DNA packaging protein UL33 42185-42198 4:01:01 CCTTCCTCGCCTCC
42221 42239 42124 42450 CDS product DNA packaging protein UL33 42221-42239 5:02:01 CCGCCCGCCCGCCCGAGCC
42291 42305 42124 42450 CDS product DNA packaging protein UL33 42291-42305 4:01:01 GGCGTCGGTGGTGGG
42389 42420 42124 42450 CDS product DNA packaging protein UL33 42389-42420 5:02:01
CCAGCTCGGCCACGTCCTCGGTGGCCAGCGCC
42469 42488 42431 44236 CDS product DNA packaging protein UL32 42469-42488 5:02:01 GGCGCCGGTGGCGGCGATGG
42628 42659 42431 44236 CDS product DNA packaging protein UL32 42628-42659 7:04:01

GGGGACGAGGACGGGGGTGGTGACAGGGACGG
42669 42680 42431 44236 CDS product DNA packaging protein UL32 42669-42680 4:01:01 GGAGGGGGGCGG
42713 42747 42431 44236 CDS product DNA packaging protein UL32 42713-42747 7:04:01
CCGCCGCCGCGCTGGCCATCGACCGGCCCTGCGCC
42793 42816 42431 44236 CDS product DNA packaging protein UL32 42793-42816 4:01:01 CCCCCCTGGGTGGCCGACTACGCC
42844 42861 42431 44236 CDS product DNA packaging protein UL32 42844-42861 4:01:01 CCGCCCTGTGCCGTGGCC
42891 42927 42431 44236 CDS product DNA packaging protein UL32 42891-42927 6:03:01
CCTGATGGACCGGCACTTCCTAGCCGCGCACCGCGCC
43011 43028 42431 44236 CDS product DNA packaging protein UL32 43011-43028 4:01:01 GGACGGCGGCGTTCCCGG
43088 43108 42431 44236 CDS product DNA packaging protein UL32 43088-43108 4:01:01 CCAACTACTCCTTCCTCATCC
43113 43165 42431 44236 CDS product DNA packaging protein UL32 43113-43165 11:08:02
GGCAACGCGGGCGCTGCTGGCTCGGCGGCGGAGGCGCCCGGAGGCGCCGGCGG
43276 43316 42431 44236 CDS product DNA packaging protein UL32 43276-43316 10:07:02
GGTGGCGGTGGGGGCGCGCGGGCGGCGCGGCGCCCGGGGGG
43412 43428 42431 44236 CDS product DNA packaging protein UL32 43412-43428 4:01:01 CCGCGGCCAGCGCCGCC
43535 43548 42431 44236 CDS product DNA packaging protein UL32 43535-43548 4:01:01 CCCGCCGCGCCGCC
43644 43669 42431 44236 CDS product DNA packaging protein UL32 43644-43669 5:02:01
GGCGCTGGGGCCGGTGCTGGCCACGG
43846 43871 42431 44236 CDS product DNA packaging protein UL32 43846-43871 5:02:01
GGCGCGCCGGTCGAGGGCGACGGCGG
44115 44139 42431 44236 CDS product DNA packaging protein UL32 44115-44139 5:02:01
CCAGCGGTTCCCCCCAAAGCCGACC
44306 44327 44229 45314 CDS product nuclear egress lamina protein 44306-44327 4:01:01

GGCGACTGGGCAACGGCCCCGG

44322 44357 44229 45314 CDS product nuclear egress lamina protein 44322-44357 6:03:01 CCCCGGCCTCGACACCAGCCCCGGCCCCGGGGCGCC

44439 44465 44229 45314 CDS product nuclear egress lamina protein 44439-44465 5:02:01 CCCGCTCCCGGGGCCCGGCCACGCGCC

44488 44504 44229 45314 CDS product nuclear egress lamina protein 44488-44504 4:01:01 GGGGGCGCGGCGCGCGG

44707 44733 44229 45314 CDS product nuclear egress lamina protein 44707-44733 5:02:01 CCCCGCCTGCACCGTGACCGGCGAGCC

44803 44825 44229 45314 CDS product nuclear egress lamina protein 44803-44825 6:03:01 CCGGGCCTTCCTGGCCTCCATCC

44834 44862 44229 45314 CDS product nuclear egress lamina protein 44834-44862 7:04:01 GGCGGGACGGACGACGGCGGCTTTGGCGG

44868 44890 44229 45314 CDS product nuclear egress lamina protein 44868-44890 5:02:01 CCCCCGGCCCCGGCGGCCCCACC

45042 45074 44229 45314 CDS product nuclear egress lamina protein 45042-45074 8:05:02 CCATCCACCTGCACCACCGCCTGCTGGACCACC

45139 45162 44229 45314 CDS product nuclear egress lamina protein 45139-45162 6:03:01 GGTGCGCCGGGACGGGGGTGGGGG

45190 45212 44229 45314 CDS product nuclear egress lamina protein 45190-45212 5:02:01 GGTGCCGGCGGTGAGCGCGGGGG

45428 45466 45238 48978 CDS product DNA polymerase catalytic subunit 45428-45466 7:04:01 GGGCCTTGAAGGTGGCGCCGACGGTGCCTAGGAGGTGGG

45503 45534 45238 48978 CDS product DNA polymerase catalytic subunit 45503-45534 5:02:01

CCCGCGCATACCCCGGGTCCTCGGCCATGTCC

45570 45586 45238 48978 CDS product DNA polymerase catalytic subunit 45570-45586 4:01:01 GGGCGGCGCGGACGGGG

45741 45762 45238 48978 CDS product DNA polymerase catalytic subunit 45741-45762 5:02:01 GGCGTAGGCCTCCGGTGGGCGG

45891 45919 45238 48978 CDS product DNA polymerase catalytic subunit 45891-45919 6:03:01 GGGCGGCACGGCCGAGGCCTCGGCCGCGG

46033 46049 45238 48978 CDS product DNA polymerase catalytic subunit 46033-46049 4:01:01 CCGCCGCCGACAAGGCC

46130 46161 45238 48978 CDS product DNA polymerase catalytic subunit 46130-46161 6:03:01 CCGCCGAAACCCGGCGCGCCATGCCGTCGCCC

46208 46230 45238 48978 CDS product DNA polymerase catalytic subunit 46208-46230 4:01:01 CCGAGTCCGTGTCGCCGTAAACC

46241 46258 45238 48978 CDS product DNA polymerase catalytic subunit 46241-46258 5:02:01 GGTAGCGGCCGGCGGCGG

46259 46275 45238 48978 CDS product DNA polymerase catalytic subunit 46259-46275 5:02:01 CCGCCGCCGGGCCGGCC

46488 46503 45238 48978 CDS product DNA polymerase catalytic subunit 46488-46503 4:01:01 GGGCGCGGCGGTGGGG

46772 46796 45238 48978 CDS product DNA polymerase catalytic subunit 46772-46796 5:02:01 CCAAAACCTTGGCCCCCTGGTACCC

46824 46859 45238 48978 CDS product DNA polymerase catalytic subunit 46824-46859 7:04:01 CCGGTCCGCCGCGCCCGCGCGCCCCTCGTTCCCCCC

46870 46915 45238 48978 CDS product DNA polymerase catalytic subunit 46870-46915 9:06:02

CCCCCCTCGTCCTCCTCTTCCTCCTCGCCGCGAGCCGCGTCGGCCC

| | | | | | | |
|---|---|---|---|---|---|---|
| 46931 | 46976 | 45238 | 48978 | CDS product DNA polymerase catalytic subunit | 46931-46976 | 9:06:02 |
| CCCCCCGTCCCCGCCCGGCGCCCCGCGCCGCGCCGGCCTCGGCTCC | | | | | | |
| 46965 | 47000 | 45238 | 48978 | CDS product DNA polymerase catalytic subunit | 46965-47000 | 6:03:01 |
| GGCCTCGGCTCCGGCGAGGCGCTTTTGGGTGTCCGG | | | | | | |
| 47084 | 47121 | 45238 | 48978 | CDS product DNA polymerase catalytic subunit | 47084-47121 | 8:05:02 |
| CCGTGATTCCGGCCAGCCGCGCCACCGCCGACAGCTCC | | | | | | |
| 47185 | 47214 | 45238 | 48978 | CDS product DNA polymerase catalytic subunit | 47185-47214 | 5:02:01 |
| CCGATGACGCCGCGCCCCTCGGGCCCGGCC | | | | | | |
| 47234 | 47280 | 45238 | 48978 | CDS product DNA polymerase catalytic subunit | 47234-47280 | 9:06:02 |
| CCTTGTAGTCCAGGTCCACCTTCCGCTCCCGCAGGGCCTCCTCGGCC | | | | | | |
| 47601 | 47623 | 45238 | 48978 | CDS product DNA polymerase catalytic subunit | 47601-47623 | 5:02:01 |
| GGCGGGCGCGGGGAGCCCGGCGG | | | | | | |
| 47639 | 47662 | 45238 | 48978 | CDS product DNA polymerase catalytic subunit | 47639-47662 | 6:03:01 |
| GGAAGGCCGGCGGCAGGTCGCAGG | | | | | | |
| 47732 | 47766 | 45238 | 48978 | CDS product DNA polymerase catalytic subunit | 47732-47766 | 6:03:01 |
| GGACGACGAGGTCCTCGGCGTTGGCGGCCGCCGGG | | | | | | |
| 47759 | 47784 | 45238 | 48978 | CDS product DNA polymerase catalytic subunit | 47759-47784 | 5:02:01 |
| CCGCCGGGAACGCCAGCCCGTCGCCC | | | | | | |
| 48143 | 48165 | 45238 | 48978 | CDS product DNA polymerase catalytic subunit | 48143-48165 | 5:02:01 |
| CCGCTGCCTCGACCACCGCGACC | | | | | | |
| 48190 | 48208 | 45238 | 48978 | CDS product DNA polymerase catalytic subunit | 48190-48208 | 4:01:01 |
| CCCAGGTAACCGCCGGGCC | | | | | | |

48219 48232 45238 48978 CDS product DNA polymerase catalytic subunit 48219-48232 4:01:01 GGCCCGGCCGGCGG
48236 48264 45238 48978 CDS product DNA polymerase catalytic subunit 48236-48264 5:02:01 CCTGCTGTGCCGCGCCGGCGGCCAGGGCC
48252 48268 45238 48978 CDS product DNA polymerase catalytic subunit 48252-48268 4:01:01 GGCGGCCAGGGCCGAGG
48324 48340 45238 48978 CDS product DNA polymerase catalytic subunit 48324-48340 4:01:01 GGCTAGGTCGACGGCGG
48397 48414 45238 48978 CDS product DNA polymerase catalytic subunit 48397-48414 4:01:01 CCGTCCTCCGACATGCCC
48428 48450 45238 48978 CDS product DNA polymerase catalytic subunit 48428-48450 5:02:01 CCGTGGCGCCGCCGGGCCGGGCC
48432 48466 45238 48978 CDS product DNA polymerase catalytic subunit 48432-48466 6:03:01 GGCGCCGCCGGGCCGGGCCTCGGCGGCAAAGCGGG
48701 48743 45238 48978 CDS product DNA polymerase catalytic subunit 48701-48743 9:06:02 CCGCCGCCCCCGCAGCCTCGTCGTCCGTGCCGCCGTCGGCGCC
48738 48764 45238 48978 CDS product DNA polymerase catalytic subunit 48738-48764 4:01:01 GGCGCCGTCGGCGTCCAGGCAGCGCGG
48776 48793 45238 48978 CDS product DNA polymerase catalytic subunit 48776-48793 5:02:01 GGAAGGACGAGGCGGCGG
48809 48837 45238 48978 CDS product DNA polymerase catalytic subunit 48809-48837 7:04:01 CCGCGCGCCCGCCGCCGCGGCCCGCCGCC
48891 48924 45238 48978 CDS product DNA polymerase catalytic subunit 48891-48924 6:03:01 GGGGGCGCCTCGGCCGCGCGGCGCGAGGTACGGG

| | | | | | | |
|---|---|---|---|---|---|---|
| 49234 | 49253 | 49233 | 52844 | CDS product single-stranded DNA-binding protein | 49234-49253 | 4:01:01 |
| GGACGCGGCGGCCAAGACGG | | | | | | |
| 49445 | 49469 | 49233 | 52844 | CDS product single-stranded DNA-binding protein | 49445-49469 | 5:02:01 |
| GGCCTGGGTGGCGCGGGGCTGACGG | | | | | | |
| 49481 | 49497 | 49233 | 52844 | CDS product single-stranded DNA-binding protein | 49481-49497 | 4:01:01 |
| CCCAGCCACTACCACCC | | | | | | |
| 49528 | 49556 | 49233 | 52844 | CDS product single-stranded DNA-binding protein | 49528-49556 | 5:02:01 |
| CCTGCGCGCCAGCACGGCCGCGCCAAACC | | | | | | |
| 49708 | 49727 | 49233 | 52844 | CDS product single-stranded DNA-binding protein | 49708-49727 | 4:01:01 |
| GGAGGCTTTCAAGGAGACGG | | | | | | |
| 49780 | 49796 | 49233 | 52844 | CDS product single-stranded DNA-binding protein | 49780-49796 | 4:01:01 |
| GGGCGCCGGCGAGGCGG | | | | | | |
| 49957 | 49973 | 49233 | 52844 | CDS product single-stranded DNA-binding protein | 49957-49973 | 4:01:01 |
| GGGCCCGGCCGCGGTGG | | | | | | |
| 50095 | 50116 | 49233 | 52844 | CDS product single-stranded DNA-binding protein | 50095-50116 | 5:02:01 |
| GGGGCGCGGTGGCGGCCGCGGG | | | | | | |
| 50126 | 50150 | 49233 | 52844 | CDS product single-stranded DNA-binding protein | 50126-50150 | 5:02:01 |
| GGCGCGGACCCGGGCGCGGCCAAGG | | | | | | |
| 50178 | 50207 | 49233 | 52844 | CDS product single-stranded DNA-binding protein | 50178-50207 | 7:04:01 |
| GGCTGGCCTCGGTGATGGCGGCGGACACGG | | | | | | |
| 50398 | 50420 | 49233 | 52844 | CDS product single-stranded DNA-binding protein | 50398-50420 | 6:03:01 |
| GGAGGTGGACGACGGCGGCGCGG | | | | | | |
| 50439 | 50496 | 49233 | 52844 | CDS product single-stranded DNA-binding protein | 50439-50496 | 11:08:02 |

CCGCCGCCGGCTTCCACCGCTTCTACCAGATCGCCGCCCCCTACGCTGCCGGGAACCC

50519 50548 49233 52844 CDS product single-stranded DNA-binding protein 50519-50548 6:03:01

CCGCTGCCGCAGACCGGCGCCGGTCCGGCC

50584 50608 49233 52844 CDS product single-stranded DNA-binding protein 50584-50608 4:01:01

GGACCACCTGGCGCTGGCGTGCGGG

50790 50817 49233 52844 CDS product single-stranded DNA-binding protein 50790-50817 5:02:01

GGCTGCGGCACCGGCTGCCCCGGTTTGG

51258 51278 49233 52844 CDS product single-stranded DNA-binding protein 51258-51278 4:01:01

GGCGCTCGGTGCTGGCCGTGG

51320 51337 49233 52844 CDS product single-stranded DNA-binding protein 51320-51337 4:01:01

GGGCAGCCGGTGGACGGG

51439 51455 49233 52844 CDS product single-stranded DNA-binding protein 51439-51455 4:01:01

GGAGGCCGCGGTCGCGG

51520 51542 49233 52844 CDS product single-stranded DNA-binding protein 51520-51542 4:01:01

GGTAAGCCTGGAGGTCTTCAAGG

51731 51758 49233 52844 CDS product single-stranded DNA-binding protein 51731-51758 6:03:01

CCGCCCGGGAGCCCGACGCCCAACCCCC

51974 52006 49233 52844 CDS product single-stranded DNA-binding protein 51974-52006 5:02:01

GGGCTCACGGCGATCGTGGCCGGGGCGCGACGG

52061 52081 49233 52844 CDS product single-stranded DNA-binding protein 52061-52081 4:01:01

CCCGACGCCGCGGCGGCCGCC

52072 52088 49233 52844 CDS product single-stranded DNA-binding protein 52072-52088 4:01:01

GGCGGCCGCCGAGGAGG

52099 52128 49233 52844 CDS product single-stranded DNA-binding protein 52099-52128 7:04:01
GGCGGAGGCCCGCCCGGAGGTGTGGGCGGG
52131 52148 49233 52844 CDS product single-stranded DNA-binding protein 52131-52148 4:01:01
CCTTCGCCGCCAGCAACC
52159 52191 49233 52844 CDS product single-stranded DNA-binding protein 52159-52191 5:02:01
GGTCATGGCCACGCGGCCCGCGGTAGTGCTGGG
52243 52272 49233 52844 CDS product single-stranded DNA-binding protein 52243-52272 6:03:01
GGCGGGCAACTGGAGCGGCCTCAACGGGGG
52421 52448 49233 52844 CDS product single-stranded DNA-binding protein 52421-52448 4:01:01
GGCAGCAACGGCGCGCTGGCGCAGACGG
52489 52529 49233 52844 CDS product single-stranded DNA-binding protein 52489-52529 8:05:02
GGAGCACATGGACCTGGACGACTGGGCGGCCCTGGTGGAGG
52552 52610 49233 52844 CDS product single-stranded DNA-binding protein 52552-52610 11:08:02
GGTGGAGCTGACGGAGCGCGCGGCCGCGCGGCCCGGGGGATGGTCGCCGGAGGGCGCGG
52630 52652 49233 52844 CDS product single-stranded DNA-binding protein 52630-52652 5:02:01
GGAGCTCGAGGCGGCGGCCGAGG
52724 52747 49233 52844 CDS product single-stranded DNA-binding protein 52724-52747 5:02:01
GGCGGCCCCGTGGCCGGGGCCGGG
52729 52778 49233 52844 CDS product single-stranded DNA-binding protein 52729-52778 9:06:02
CCCCGTGGCCGGGGCCGGGCCCGCCGCGCCTGGAGCCAAGCGCCCCGACC
53095 53116 53058 55538 CDS product DNA packaging terminase subunit 2 53095-53116 4:01:01
CCTCGGCTCCGAGCCTGGCCCC
53153 53165 53058 55538 CDS product DNA packaging terminase subunit 2 53153-53165 4:01:01 GGCGGGGCGGCGG

53257 53272 53058 55538 CDS product DNA packaging terminase subunit 2 53257-53272 4:01:01 CCGCCACCTCGACGCC
53607 53629 53058 55538 CDS product DNA packaging terminase subunit 2 53607-53629 4:01:01 CCTCAACCGACGCCGCGCTCGCC
53658 53684 53058 55538 CDS product DNA packaging terminase subunit 2 53658-53684 7:04:01 GGGCCACGGTGGCGGGCGGCGGGGTGG
53770 53783 53058 55538 CDS product DNA packaging terminase subunit 2 53770-53783 4:01:01 GGGCCGGCGGCTGG
53880 53924 53058 55538 CDS product DNA packaging terminase subunit 2 53880-53924 11:08:02 GGGCGCGGCTGGCGGCGGCAGCGGCGGCGCTGGCGGCGCTGGCGG
53938 53950 53058 55538 CDS product DNA packaging terminase subunit 2 53938-53950 4:01:01 GGGGGCGGGCGGG
53978 53997 53058 55538 CDS product DNA packaging terminase subunit 2 53978-53997 5:02:01 GGAGGCGACGGCGACGGCGG
54037 54057 53058 55538 CDS product DNA packaging terminase subunit 2 54037-54057 4:01:01 CCACGTCTTCCGCCCGGCGCC
54120 54155 53058 55538 CDS product DNA packaging terminase subunit 2 54120-54155 8:05:02 CCGCCGCCAGCGCCGTCGCCGCCTTCGCCGGCAACC
54199 54215 53058 55538 CDS product DNA packaging terminase subunit 2 54199-54215 4:01:01 GGCCGCGGCGGAGATGG
54259 54287 53058 55538 CDS product DNA packaging terminase subunit 2 54259-54287 5:02:01 GGACAAGCTGGCGCAGCTGGACGGCGTGG
54385 54407 53058 55538 CDS product DNA packaging terminase subunit 2 54385-54407 4:01:01 CCGGCGCCTCACGCGCCCGGACC
54441 54465 53058 55538 CDS product DNA packaging terminase subunit 2 54441-54465 6:03:01 GGCGGGCGGCCGCGGGCGAGGCGGG

54475 54495 53058 55538 CDS product DNA packaging terminase subunit 2 54475-54495 5:02:01
GGTTGGCCTCGGCGGCCCGGG

54526 54542 53058 55538 CDS product DNA packaging terminase subunit 2 54526-54542 5:02:01
GGAGGAGGGCGGGACGG

54551 54659 53058 55538 CDS product DNA packaging terminase subunit 2 54551-54659 23:20:05
GGCGGCGCCGGGGCCGGGGTGGGGGGCGGTGGGGCGGGGCCGTCCGGGGGCGCAGACGGCGGGGGCGGCGCGGAGGACGACGGCCCCGCGGGCTGGGCGGCGCTGGCGG

54850 54865 53058 55538 CDS product DNA packaging terminase subunit 2 54850-54865 4:01:01 CCGCGCCTTCCGCGCC

54871 54893 53058 55538 CDS product DNA packaging terminase subunit 2 54871-54893 5:02:01
GGCGAGCGCGGGCACGGTGGTGG

54898 54916 53058 55538 CDS product DNA packaging terminase subunit 2 54898-54916 4:01:01
CCGCGCCTCGGACGCCACC

55138 55163 53058 55538 CDS product DNA packaging terminase subunit 2 55138-55163 7:04:01
GGCGGCCGCGGGGGGCGGGTGGGCGG

55321 55351 53058 55538 CDS product DNA packaging terminase subunit 2 55321-55351 6:03:01
GGACCGAACGGCGCCGGGCCCGGACGGGCGG

55425 55448 53058 55538 CDS product DNA packaging terminase subunit 2 55425-55448 5:02:01
GGGCGCCGGAGACGGTCGGCGAGG

55609 55677 55394 58192 CDS product envelope glycoprotein B 55609-55677 13:10:03
CCCCCGGCGCCCCCGCCCGAAGAGGCCGCGAGCCCGGCGCCCCCCGCGAGCCCCAGCCCCCCCGGCCCC

55776 55794 55394 58192 CDS product envelope glycoprotein B 55776-55794 4:01:01 CCCGCCGCCCTCGGGCGCC

55788 55806 55394 58192 CDS product envelope glycoprotein B 55788-55806 4:01:01 GGGCGCCACGGTGGTCCGG

56074 56089 55394 58192 CDS product envelope glycoprotein B 56074-56089 4:01:01 GGGCGCAAGGTGGTGG

56190 56208 55394 58192 CDS product envelope glycoprotein B 56190-56208 4:01:01 GGCGCTGGGCTCGGCGGGG

56244 56266 55394 58192 CDS product envelope glycoprotein B 56244-56266 4:01:01 GGAAGAAGTGGAGGCGCGCTCGG
56523 56542 55394 58192 CDS product envelope glycoprotein B 56523-56542 4:01:01 GGCCAAGTGGCGCGAGGCGG
56864 56888 55394 58192 CDS product envelope glycoprotein B 56864-56888 5:02:01 CCGCCGCGGCGCCCAAGCCGGGCCC
56901 56930 55394 58192 CDS product envelope glycoprotein B 56901-56930 5:02:01
CCGCGCCGCGCCGTCTGCGCCCGGCGGCCC
56923 56977 55394 58192 CDS product envelope glycoprotein B 56923-56977 10:07:02
GGCGGCCCGGGCGCGGCCAACGGGCCCGCCGGCGACGGCGACGCCGGCGGGCGGG
57020 57040 55394 58192 CDS product envelope glycoprotein B 57020-57040 4:01:01 CCTACGACCACATCCAGGACC
57060 57082 55394 58192 CDS product envelope glycoprotein B 57060-57082 5:02:01 CCGCCTGGCCACGTCCTGGTGCC
57162 57174 55394 58192 CDS product envelope glycoprotein B 57162-57174 4:01:01 CCGCCGCGCCGCC
57282 57303 55394 58192 CDS product envelope glycoprotein B 57282-57303 5:02:01 CCGCCCGCCGGTCTCCTTTGCC
57324 57346 55394 58192 CDS product envelope glycoprotein B 57324-57346 5:02:01 GGTGGAGGGCCAGCTCGGCGAGG
57382 57397 55394 58192 CDS product envelope glycoprotein B 57382-57397 4:01:01 CCCTGCACCGCCAACC
57654 57676 55394 58192 CDS product envelope glycoprotein B 57654-57676 4:01:01 GGTCAAGACGGACGGCAATATGG
57708 57761 55394 58192 CDS product envelope glycoprotein B 57708-57761 10:07:02
GGGCCTGGGCGCCGTCGGGCAGGCGGTGGGCACGGTGGTGCTGGGCGCCGCGGG
57814 57838 55394 58192 CDS product envelope glycoprotein B 57814-57838 4:01:01 GGCGCGCTGGCCACGGGGCTGCTGG
57884 57903 55394 58192 CDS product envelope glycoprotein B 57884-57903 4:01:01 CCCGCCTCCGCAGCAACCCC
57969 57985 55394 58192 CDS product envelope glycoprotein B 57969-57985 4:01:01 GGGCGAGGAAGAGGAGG
58732 58768 58678 59604 CDS product capsid scaffold protein 58732-58768 9:06:02
GGCGGTTGGGCGGGCGGCAGGCTGGCCATGGCGCTGG
58772 58808 58678 59604 CDS product capsid scaffold protein 58772-58808 7:04:01
CCACCGTCGCGGCCACCCCGGGCTCCTCCGGCGGCCC

58782 58805 58678 59604 CDS product capsid scaffold protein 58782-58805 4:01:01 GGCCACCCCGGGCTCCTCCGGCGG
58815 58838 58678 59604 CDS product capsid scaffold protein 58815-58838 5:02:01 GGCGGCGGCCGCGACGGTCGCTGG
58868 58887 58678 59604 CDS product capsid scaffold protein 58868-58887 4:01:01 CCTGCGCCGGCGCCTGGGCC
58876 58914 58678 59604 CDS product capsid scaffold protein 58876-58914 7:04:01
GGCGCCTGGGCCGGTGCGAGGGTCGGCACTTGGGCCGGG
58910 58927 58678 59604 CDS product capsid scaffold protein 58910-58927 4:01:01 CCGGGCCCGCACCTACCC
58998 59020 58678 59604 CDS product capsid scaffold protein 58998-59020 4:01:01 GGCAGGACCGTAGGGGTAGCCGG
59052 59074 58678 59604 CDS product capsid scaffold protein 59052-59074 5:02:01 GGACACGGCGGAGGCGAGCTTGG
59089 59141 58678 59604 CDS product capsid scaffold protein 59089-59141 13:10:03
GGTGGCGGGTGCGGCGGCGGAGGAGAGTGGTGGTGAGGCGGCAGCTCGGCTGG
59179 59195 58678 59604 CDS product capsid scaffold protein 59179-59195 5:02:01 CCGCCGCCGCTCCGGCC
59207 59232 58678 59604 CDS product capsid scaffold protein 59207-59232 4:01:01 CCCAGTTGTACCGGCGCCGCTTGGCC
59229 59253 58678 59604 CDS product capsid scaffold protein 59229-59253 7:04:01 GGCCAGGGGGCGGCGGTCGAGGGGG
59254 59279 58678 59604 CDS product capsid scaffold protein 59254-59279 5:02:01 CCGTCGTAGCCCGAGCCGTCCGCGCC
59297 59323 58678 59604 CDS product capsid scaffold protein 59297-59323 5:02:01 GGCCGTCGGCGATGGCGCCGGCCAGGG
59368 59392 58678 59604 CDS product capsid scaffold protein 59368-59392 5:02:01 GGGTGCCAGGGCGCGGCGCCGGCGG
59386 59406 58678 59604 CDS product capsid scaffold protein 59386-59406 4:01:01 CCGGCGGCGCCGTACCAGCCC
59407 59447 58678 59604 CDS product capsid scaffold protein 59407-59447 11:08:02
GGCATCGGGGGCGGAGCGGCGGCGGCAGCGGCGGCGGCCGG
59458 59479 58678 59604 CDS product capsid scaffold protein 59458-59479 6:03:01 GGAGCCGGCGGAGGCGCGGCGG
59604 59652 58678 60543 CDS product capsid maturation protease 59604-59652 13:10:03
GGCAGGCGGTGGGGGCGGCGGGGGCGGTGCCGGCGGAAGCTCTGGCTGG
59906 59935 58678 60543 CDS product capsid maturation protease 59906-59935 5:02:01

GGGGCACCGGCCAGGCGCGGTCGCCGAGGG
59958 59971 58678 60543 CDS product capsid maturation protease 59958-59971 4:01:01 GGCGAGGAGGGCGG
60355 60374 58678 60543 CDS product capsid maturation protease 60355-60374 5:02:01 GGCAGCGGGGCGGCGGGCGG
60780 60793 60602 62398 CDS product DNA packaging tegument protein UL25 60780-60793 4:01:01 CCGCCACGACCTCC
60799 60823 60602 62398 CDS product DNA packaging tegument protein UL25 60799-60823 6:03:01 GGGCGCGGGCGCGGCGCGGTGGCGG
61052 61083 60602 62398 CDS product DNA packaging tegument protein UL25 61052-61083 5:02:01 CCCACGGCGCCGTCGAGGCCGCCATCGGCGCC
61107 61128 60602 62398 CDS product DNA packaging tegument protein UL25 61107-61128 4:01:01 CCGCCCCGGGCACCAGCGTGCC
61182 61205 60602 62398 CDS product DNA packaging tegument protein UL25 61182-61205 5:02:01 GGCGGCGCACCAGGAGCAAGGCGG
61239 61258 60602 62398 CDS product DNA packaging tegument protein UL25 61239-61258 4:01:01 GGTCCTCGGCGAGGAAAAGG
61347 61368 60602 62398 CDS product DNA packaging tegument protein UL25 61347-61368 5:02:01 CCGCGCCGCCGCCTAGCGCGCC
61448 61462 60602 62398 CDS product DNA packaging tegument protein UL25 61448-61462 4:01:01 CCCGCGCCGCCGCCC
61540 61570 60602 62398 CDS product DNA packaging tegument protein UL25 61540-61570 5:02:01 GGCGCGGGCCAGGGCGTCCAGGTACGCCGGG
61618 61639 60602 62398 CDS product DNA packaging tegument protein UL25 61618-61639 4:01:01 GGCAACGGCACGGTGCGCGAGG
61716 61732 60602 62398 CDS product DNA packaging tegument protein UL25 61716-61732 5:02:01 CCGCCACCGCGGCCGCC

61844 61860 60602 62398 CDS product DNA packaging tegument protein UL25 61844-61860 5:02:01
CCCGCCGACCCCCCGCC
61890 61907 60602 62398 CDS product DNA packaging tegument protein UL25 61890-61907 5:02:01
GGTCGGCGGCCAGGGTGG
61976 61999 60602 62398 CDS product DNA packaging tegument protein UL25 61976-61999 7:04:01
CCCGCGCCGCCGCCCCCCTCCTCC
62008 62027 60602 62398 CDS product DNA packaging tegument protein UL25 62008-62027 4:01:01
GGCGGCGCGGCCCGCGCGGG
62018 62083 60602 62398 CDS product DNA packaging tegument protein UL25 62018-62083 15:12:03
CCCGCGCGGGCCGACTCCGCCGCCGCGTCCGCCTCCTCCGCCCGGCGCGCCGCCTCCTCCAGGTCC
62100 62116 60602 62398 CDS product DNA packaging tegument protein UL25 62100-62116 5:02:01
CCTCCGCCACCCGGGCC
62181 62213 60602 62398 CDS product DNA packaging tegument protein UL25 62181-62213 6:03:01
CCGCGGCCGCTGCCGCGGCCTGGTTCCGCGCCC
62386 62412 62380 63261 CDS product nuclear protein UL24 62386-62412 5:02:01 CCCGAGCGCCATGGCCCCGATGCCGCC
62434 62454 62380 63261 CDS product nuclear protein UL24 62434-62454 4:01:01 CCCAGGCACGCCGCGCCGCCC
62488 62532 62380 63261 CDS product nuclear protein UL24 62488-62532 8:05:02
CCCGCGTGCCTGCTGCCGCCCACCGGGCGGCCGCTCTCCCCGGCC
62539 62575 62380 63261 CDS product nuclear protein UL24 62539-62575 8:05:02
GGGCCGGGGTCGCCGGCGGGCGGCCGCGGGGTGGCGG
62576 62601 62380 63261 CDS product nuclear protein UL24 62576-62601 5:02:01 CCACAGCCGCCCTTTTGCCCGTAGCC
62616 62635 62380 63261 CDS product nuclear protein UL24 62616-62635 4:01:01 CCCCTTCTGCCGCCGCGGCC
62830 62863 62380 63261 CDS product nuclear protein UL24 62830-62863 6:03:01

GGCGCTAGGACCACCGGGTCTGGGCCCGGGGGGG

62993 63019 62380 63261 CDS product nuclear protein UL24 62993-63019 5:02:01 CCCGGGCGTGCCCTGCCTCCGCGACCC

63101 63123 62380 63261 CDS product nuclear protein UL24 63101-63123 4:01:01 CCTCCGGGGCGCCAAACTTGCCC

63203 63231 62380 63261 CDS product nuclear protein UL24 63203-63231 5:02:01

GGCTGTGGCACCGGATCCCGGCGCGCAGG

63303 63323 63260 64339 CDS product thymidine kinase 63303-63323 4:01:01 GGACGGCGCGCACGGGCTGGG

63493 63508 63260 64339 CDS product thymidine kinase 63493-63508 4:01:01 GGCGCCGGCGGGCCGG

63525 63541 63260 64339 CDS product thymidine kinase 63525-63541 4:01:01 GGACGCGGCGGGCCTGG

63613 63629 63260 64339 CDS product thymidine kinase 63613-63629 4:01:01 CCGCCTGGGCCGGCGCC

63619 63635 63260 64339 CDS product thymidine kinase 63619-63635 4:01:01 GGGCCGGCGCCGGGCGG

63657 63709 63260 64339 CDS product thymidine kinase 63657-63709 9:06:02

CCGCCACCCCGTGGCCGCGTGCCTCTGCTACCCCTTCGCCCGCTACTGCCTCC

63746 63778 63260 64339 CDS product thymidine kinase 63746-63778 7:04:01

CCGCCATGCCCCCGGAAGCGCCCGGGGCCAACC

63791 63804 63260 64339 CDS product thymidine kinase 63791-63804 4:01:01 CCCTCCCCCCGGCC

63822 63842 63260 64339 CDS product thymidine kinase 63822-63842 5:02:01 GGCGGCGCGGGCCAGGCCCGG

63924 63967 63260 64339 CDS product thymidine kinase 63924-63967 8:05:02

GGGGGGCGCATGGCGCGACGGCTGGGACGCGCTGGAGTGGGCGG

64083 64097 63260 64339 CDS product thymidine kinase 64083-64097 4:01:01 GGGCGGCGGGACGGG

64118 64139 63260 64339 CDS product thymidine kinase 64118-64139 4:01:01 GGGCGCTGGACGCCCTGGCCGG

64130 64153 63260 64339 CDS product thymidine kinase 64130-64153 6:03:01 CCCTGGCCGGCCGCCTCGCCGCCC

64245 64259 63260 64339 CDS product thymidine kinase 64245-64259 4:01:01 GGACGGGGCGGCGGG

64302 64331 63260 64339 CDS product thymidine kinase 64302-64331 7:04:01 GGCGGGGGAGGCCACGGCGGGCCCTAGGGG

| | | | | | | | |
|---|---|---|---|---|---|---|---|
| 64506 | 64529 | 64455 | 66983 | CDS product envelope glycoprotein H | 64506-64529 | 6:03:01 | CCGCGGGCGCCCCCGCCGCCGCCC |
| 64532 | 64545 | 64455 | 66983 | CDS product envelope glycoprotein H | 64532-64545 | 4:01:01 | GGCGGCGCGGGGGG |
| 64590 | 64616 | 64455 | 66983 | CDS product envelope glycoprotein H | 64590-64616 | 6:03:01 | GGGAAATGGTGGTCGGAGCCGGGCCGG |
| 64632 | 64650 | 64455 | 66983 | CDS product envelope glycoprotein H | 64632-64650 | 4:01:01 | CCATCCGCTGCCTCGGGCC |
| 64900 | 64919 | 64455 | 66983 | CDS product envelope glycoprotein H | 64900-64919 | 5:02:01 | CCTGGTGCCGAGCCGCCCCC |
| 64962 | 64983 | 64455 | 66983 | CDS product envelope glycoprotein H | 64962-64983 | 5:02:01 | CCGCGCGCCCGCCGCCCGCCCC |
| 65002 | 65015 | 64455 | 66983 | CDS product envelope glycoprotein H | 65002-65015 | 4:01:01 | GGCGGGGCGGGTGG |
| 65182 | 65205 | 64455 | 66983 | CDS product envelope glycoprotein H | 65182-65205 | 6:03:01 | GGCTCGGCTGGCGGTGGGGCTCGG |
| 65318 | 65343 | 64455 | 66983 | CDS product envelope glycoprotein H | 65318-65343 | 6:03:01 | CCGCCACCCGGGCCCGCGGCCGGTCC |
| 65366 | 65387 | 64455 | 66983 | CDS product envelope glycoprotein H | 65366-65387 | 4:01:01 | GGCTACGGGAACTCGCGGCTGG |
| 65508 | 65564 | 64455 | 66983 | CDS product envelope glycoprotein H | 65508-65564 | 9:06:02 | CCGCGCGCCGCGGACCGAGCGCCGGCCCCGCGCCCAACGCTGCCTACCACGCGTACC |
| 65565 | 65583 | 64455 | 66983 | CDS product envelope glycoprotein H | 65565-65583 | 4:01:01 | GGGTCGCGGCGCGGCTGGG |
| 65608 | 65625 | 64455 | 66983 | CDS product envelope glycoprotein H | 65608-65625 | 4:01:01 | GGGGGCGCTCGCGGACGG |
| 65889 | 65915 | 64455 | 66983 | CDS product envelope glycoprotein H | 65889-65915 | 5:02:01 | GGCTGCTGGCCCACGGCGTGGTGTCGG |
| 66069 | 66091 | 64455 | 66983 | CDS product envelope glycoprotein H | 66069-66091 | 4:01:01 | CCGCCGGGCACGCCACGGCGGCC |
| 66085 | 66110 | 64455 | 66983 | CDS product envelope glycoprotein H | 66085-66110 | 6:03:01 | GGCGGCCGCGCTGGACCTGGAGGAGG |
| 66125 | 66150 | 64455 | 66983 | CDS product envelope glycoprotein H | 66125-66150 | 6:03:01 | GGCGGCGGCGCCGGGGGCGACGCGGG |
| 66252 | 66278 | 64455 | 66983 | CDS product envelope glycoprotein H | 66252-66278 | 5:02:01 | GGGCCGCGCTGGACGCCTGGCTGGAGG |
| 66284 | 66305 | 64455 | 66983 | CDS product envelope glycoprotein H | 66284-66305 | 5:02:01 | CCCGCGGCCGCCGCGCCGAACC |
| 66313 | 66356 | 64455 | 66983 | CDS product envelope glycoprotein H | 66313-66356 | 8:05:02 | GGCGGCGCTCGGCATGCTGGGCCGGGGAGGCCTCTTCGGCCCGG |
| 66401 | 66417 | 64455 | 66983 | CDS product envelope glycoprotein H | 66401-66417 | 4:01:01 | GGCGGGTGGGGCGCGGG |

66568 66587 64455 66983 CDS product envelope glycoprotein H 66568-66587 5:02:01 GGCCAGCGGCGCGGTGGAGG
66592 66612 64455 66983 CDS product envelope glycoprotein H 66592-66612 4:01:01 CCGCCTTGCGGTCCCCGGCCC
66870 66900 64455 66983 CDS product envelope glycoprotein H 66870-66900 6:03:01
GGGCGTCGGTAGGCGGGGCGCTGGTCGCGGG
67145 67179 67133 68857 CDS product tegument protein UL21 67145-67179 6:03:01
CCGGCTCCAGCTTCCCCTTCTCCCTCCCCTTCGCC
67226 67244 67133 68857 CDS product tegument protein UL21 67226-67244 5:02:01 CCGGCCGCCCCAAGCCGCC
67376 67395 67133 68857 CDS product tegument protein UL21 67376-67395 4:01:01 CCAGCGCCGGCAGCCCCGCC
67580 67612 67133 68857 CDS product tegument protein UL21 67580-67612 8:05:02
CCGCCGCCGTCTCCGCCGGCGCCGCCTTTTTCC
67701 67720 67133 68857 CDS product tegument protein UL21 67701-67720 4:01:01 CCGCGTCCGCGACCGTGCCC
67728 67760 67133 68857 CDS product tegument protein UL21 67728-67760 7:04:01
GGGGCGCGGCGGCGCGGTGCGGATAATTGGCGG
67883 67911 67133 68857 CDS product tegument protein UL21 67883-67911 4:01:01
CCGACAAAGCCGCGGCGTCCGCGCGGCCC
67920 67951 67133 68857 CDS product tegument protein UL21 67920-67951 6:03:01
CCGTCGCCGCGGCCACCAAGTCCCCGCGCGCC
68071 68095 67133 68857 CDS product tegument protein UL21 68071-68095 4:01:01 GGCCCAGGCATTGTAGGTCCGCGGG
68217 68247 67133 68857 CDS product tegument protein UL21 68217-68247 6:03:01
CCCCTTCTCCGGCGCCGCGCCGCAGCCCGCC
68281 68304 67133 68857 CDS product tegument protein UL21 68281-68304 5:02:01 GGGGGCGCACGGGCCGCGGCGGGG
68526 68550 67133 68857 CDS product tegument protein UL21 68526-68550 5:02:01 CCGCGCCCGCGGCCGGGGCCCCGCC
68727 68741 67133 68857 CDS product tegument protein UL21 68727-68741 4:01:01 CCGCCTCCCCGCCCC

69033 69050 68969 69664 CDS product envelope protein UL20 69033-69050 5:02:01 GGAAGAGGGGGCGGCCGG
69060 69088 68969 69664 CDS product envelope protein UL20 69060-69088 5:02:01 GGGAGACAGCGGCTCGGACGACGAGGAGG
69117 69136 68969 69664 CDS product envelope protein UL20 69117-69136 4:01:01 GGCGTACGGGGGCGACGTGG
69195 69217 68969 69664 CDS product envelope protein UL20 69195-69217 4:01:01 GGCGTACGTGGTCTTTGGCGCGG
69335 69353 68969 69664 CDS product envelope protein UL20 69335-69353 5:02:01 GGCTGGCGGCGGCCGCGGG
69366 69384 68969 69664 CDS product envelope protein UL20 69366-69384 4:01:01 GGGCGTGCGGGCGGACCGG
69394 69487 68969 69664 CDS product envelope protein UL20 69394-69487 16:13:04 GGGCGCGGGCCTCGGGCGCTGCTGGCGGCGCTGGTGCTGGCGCGCGCGGCGGTCTTTGCGGCGGTGGCGCTGCCGGCGGCCTTTGCGGGCCCGG
69510 69527 68969 69664 CDS product envelope protein UL20 69510-69527 6:03:01 GGCGGTGGCGGACGGCGG
69537 69549 68969 69664 CDS product envelope protein UL20 69537-69549 4:01:01 GGCGGCGGCGGGG
69558 69583 68969 69664 CDS product envelope protein UL20 69558-69583 6:03:01 GGCCGGGCTGGCGGCGTACACGGCGG
69754 69771 69737 73894 CDS product major capsid protein 69754-69771 4:01:01 CCTCCGCGCGCGCCCGCC
69777 69794 69737 73894 CDS product major capsid protein 69777-69794 4:01:01 GGGCGGGCTGGTCTCCGG
69945 69964 69737 73894 CDS product major capsid protein 69945-69964 4:01:01 GGAGCTCGGGCTCTCGGTGG
70057 70077 69737 73894 CDS product major capsid protein 70057-70077 4:01:01 CCACATCCGGTGGACCAGCCC
70482 70516 69737 73894 CDS product major capsid protein 70482-70516 6:03:01 GGACCTCGTGGGCTGCACGGCCGCGTCGGTGGCGG
70546 70583 69737 73894 CDS product major capsid protein 70546-70583 7:04:01 GGGCGCCCGGTGGATGGCGTGCTGGTGACGACGGCGGG
70595 70636 69737 73894 CDS product major capsid protein 70595-70636 8:05:02 GGCTGCTGGGCGGCGTGCTGGCGCTGGCCGACTCGGAGGCGG
70746 70783 69737 73894 CDS product major capsid protein 70746-70783 7:04:01

GGGGCTGGAGGGGACCGCGGGCCCGGGGCTGGCCGTGG

70821 70848 69737 73894 CDS product major capsid protein 70821-70848 6:03:01 GGCGGACCTGGTGGCCGTTGGGGACCGG

71002 71024 69737 73894 CDS product major capsid protein 71002-71024 5:02:01 CCGCCGCCCGGGCACCCCGACCC

71130 71190 69737 73894 CDS product major capsid protein 71130-71190 12:09:03

GGACGTGGACCCGGTGCTGGCGGCGCTCGGCCGGCGCGGCCAGAGGCGAGAGGCGGAGTGG

71221 71250 69737 73894 CDS product major capsid protein 71221-71250 5:02:01

GGCGAGCAGGAGCTCGCGGACCAGGTGCGG

71322 71342 69737 73894 CDS product major capsid protein 71322-71342 4:01:01 GGAACAGATGGTGGCGCCCGG

71413 71432 69737 73894 CDS product major capsid protein 71413-71432 5:02:01 CCAGGGCCACCCAACCCACC

71562 71585 69737 73894 CDS product major capsid protein 71562-71585 6:03:01 GGCAACGGTGGCGGCGGTGCGCGG

71793 71824 69737 73894 CDS product major capsid protein 71793-71824 5:02:01

GGACTGCACGGCGGTCTACAAGGACCTGCTGG

71928 71945 69737 73894 CDS product major capsid protein 71928-71945 4:01:01 CCCGGTGCTTCCGCCGCC

71984 72001 69737 73894 CDS product major capsid protein 71984-72001 4:01:01 GGCGGACCCGGCGCGCGG

72112 72136 69737 73894 CDS product major capsid protein 72112-72136 5:02:01 GGCGGCGGCCCCGGGGCGTACCCGG

72258 72280 69737 73894 CDS product major capsid protein 72258-72280 4:01:01 GGTGCAGTCGGTCGTGGTGCCGG

72301 72363 69737 73894 CDS product major capsid protein 72301-72363 10:07:02

CCGCCCGCGGGCCCCGAGCACCCGCGCCACCCGCTGCACCCGACGAACCTGGTGGCCAACTCC

72393 72406 69737 73894 CDS product major capsid protein 72393-72406 4:01:01 GGCGGTGGACGCGG

72431 72456 69737 73894 CDS product major capsid protein 72431-72456 4:01:01 CCGTGACCAACATGGCCGAGCGCACC

72459 72479 69737 73894 CDS product major capsid protein 72459-72479 6:03:01 GGTGGTGGTGGTGGACGCGGG

72635 72649 69737 73894 CDS product major capsid protein 72635-72649 4:01:01 CCTGCCCGGACCACC

72993 73011 69737 73894 CDS product major capsid protein 72993-73011 5:02:01 GGCGGTCGGCGGCGTCGGG

73041 73066 69737 73894 CDS product major capsid protein 73041-73066 5:02:01 GGACCTCGGCGTGGGCTACACGGCGG

73067 73083 69737 73894 CDS product major capsid protein 73067-73083 4:01:01 CCTACGCCGCCGCGGCC

73155 73175 69737 73894 CDS product major capsid protein 73155-73175 4:01:01 GGTGAACGGGGACGCGGACGG

73240 73259 69737 73894 CDS product major capsid protein 73240-73259 4:01:01 CCCATTATGCCCGCCGCGCC

73260 73288 69737 73894 CDS product major capsid protein 73260-73288 6:03:01 GGCGGGCACGGCGCGCGGGCAGGCTGCGG

73335 73354 69737 73894 CDS product major capsid protein 73335-73354 4:01:01 CCGCTCGCCGTGCAACCCCC

73357 73375 69737 73894 CDS product major capsid protein 73357-73375 4:01:01 GGGCGCTCGGCAGGCGCGG

73422 73476 69737 73894 CDS product major capsid protein 73422-73476 10:07:02 CCATGGCCAGGGCGACCCGGCCCACCCGCACCGCGCCACCGTTAACCCGTGGGCC

73575 73594 69737 73894 CDS product major capsid protein 73575-73594 5:02:01 GGCGGAGGTGTCGGCCAAGG

73620 73634 69737 73894 CDS product major capsid protein 73620-73634 4:01:01 GGAGGTGGGCGCGGG

73773 73788 69737 73894 CDS product major capsid protein 73773-73788 4:01:01 CCGCCTCGCCGCGCCC

73789 73829 69737 73894 CDS product major capsid protein 73789-73829 9:06:02 GGCGGCGCGGCCGCGGGCGTCGAGGGCCGGCTGGAGGAGGG

73845 73865 69737 73894 CDS product major capsid protein 73845-73865 4:01:01 CCTGATCCGCGACGCCTCGCC

74044 74057 73983 74933 CDS product capsid triplex subunit 2 74044-74057 4:01:01 CCTAGCCGCCCTCC

74113 74129 73983 74933 CDS product capsid triplex subunit 2 74113-74129 4:01:01 GGCGGACGTGGCGCTGG

74178 74203 73983 74933 CDS product capsid triplex subunit 2 74178-74203 5:02:01 CCGCGTACCGCTGCCGGTTCCCGGCC

74368 74384 73983 74933 CDS product capsid triplex subunit 2 74368-74384 4:01:01 GGCCTCGGCGGGCGCGG

74431 74480 73983 74933 CDS product capsid triplex subunit 2 74431-74480 9:06:02 GGTGGCGCGCGTGGTGGCGCGCGCGGTAGAGGCGCTGGGGGACCGCGCGG

74665 74684 73983 74933 CDS product capsid triplex subunit 2 74665-74684 4:01:01 GGGCCTGCAGGACGGCGTGG
74740 74757 73983 74933 CDS product capsid triplex subunit 2 74740-74757 4:01:01 CCTGGAGCCGGCCGAGCC
74760 74776 73983 74933 CDS product capsid triplex subunit 2 74760-74776 5:02:01 GGCAGGACGGCGGCCGG
74792 74809 73983 74933 CDS product capsid triplex subunit 2 74792-74809 4:01:01 GGGGCGCTGGCGGCCTGG
74818 74844 73983 74933 CDS product capsid triplex subunit 2 74818-74844 5:02:01 GGCGACCCGGCTGGGAGACGCGGTCGG
75086 75133 75077 80655 CDS product DNA packaging terminase subunit 1 75086-75133 10:07:02 GGCGGGCGGGGGGCCAGCGGGCAGAAGGCGCCGTCGGTGGGCGGGAGG
75197 75225 75077 80655 CDS product DNA packaging terminase subunit 1 75197-75225 5:02:01 CCCGAGAAGGCCCGGGCCCCGCCGGGGCC
75205 75244 75077 80655 CDS product DNA packaging terminase subunit 1 75205-75244 7:04:01 GGCCCGGGCCCCGCCGGGGCCGGCGGTGCTCTCGGTTAGG
75288 75320 75077 80655 CDS product DNA packaging terminase subunit 1 75288-75320 6:03:01 GGCGCACGGTCAGCGAGGCCAGGTCCTGGGAGG
75348 75368 75077 80655 CDS product DNA packaging terminase subunit 1 75348-75368 4:01:01 GGATGAAGTGGTCAAAGGCGG
75418 75433 75077 80655 CDS product DNA packaging terminase subunit 1 75418-75433 4:01:01 GGGCGGGCGGCAGTGG
75448 75476 75077 80655 CDS product DNA packaging terminase subunit 1 75448-75476 6:03:01 GGCGGGCATGGGCACGGCACCGGACGCGG
75561 75587 75077 80655 CDS product DNA packaging terminase subunit 1 75561-75587 5:02:01 GGCCGTCGAGGCGGCGCCGGTGCACGG
75672 75709 75077 80655 CDS product DNA packaging terminase subunit 1 75672-75709 7:04:01 CCAGGCCCAGCACCAGCCAGCTGCCGCGATGCCGCCCC

75715 75740 75077 80655 CDS product DNA packaging terminase subunit 1 75715-75740 5:02:01
GGCGATGCCGGAGCCGGAGGCGCGGG
75778 75808 75077 80655 CDS product DNA packaging terminase subunit 1 75778-75808 6:03:01
CCGCTCGGGCCACGGGCCGCGGGCCGCCGCC
75849 75877 75077 80655 CDS product DNA packaging terminase subunit 1 75849-75877 5:02:01
GGGTGAACACGGCCGCGGGCTCGGCGCGG
75885 75897 75077 80655 CDS product DNA packaging terminase subunit 1 75885-75897 4:01:01 CCTCGCCGCCGCC
75922 75941 75077 80655 CDS product DNA packaging terminase subunit 1 75922-75941 5:02:01
GGGCAGGAAGGTCTCGGCGG
76013 76027 75077 80655 CDS product DNA packaging terminase subunit 1 76013-76027 4:01:01 CCGCCGTGCGCCGCC
76117 76139 75077 80655 CDS product DNA packaging terminase subunit 1 76117-76139 4:01:01
GGCCTTGCCGGTGTTGGTCGAGG
76191 76207 75077 80655 CDS product DNA packaging terminase subunit 1 76191-76207 4:01:01
GGACGGCCTCGGGGCGG
76307 76330 75077 80655 CDS product DNA packaging terminase subunit 1 76307-76330 5:02:01
CCGACCTCGTCCCGACCCGACGCC
76386 76406 76332 78437 CDS product DNA packaging tegument protein UL17 76386-76406 4:01:01
CCGCGGGCACCGTCGTCCACC
76431 76452 76332 78437 CDS product DNA packaging tegument protein UL17 76431-76452 4:01:01
GGGCCGCCGGGGCGGACCCCGG
76465 76499 76332 78437 CDS product DNA packaging tegument protein UL17 76465-76499 9:06:02
GGCCTCGGGGGGGGCGCCGGGGGCCGCGGCGGGGG
76637 76662 76332 78437 CDS product DNA packaging tegument protein UL17 76637-76662 5:02:01

CCCACGCAGCCGGCGGCCCGGCCCCC
76713 76732 76332 78437 CDS product DNA packaging tegument protein UL17 76713-76732 4:01:01
CCCTGCCCGTCGCCCTGCCC
76774 76800 76332 78437 CDS product DNA packaging tegument protein UL17 76774-76800 5:02:01
GGCGCTGCGGCTGGAAATGGCCGACGG
76863 76886 76332 78437 CDS product DNA packaging tegument protein UL17 76863-76886 5:02:01
CCGACGTCCCGCGCCTGGCCGCCC
76918 76954 76332 78437 CDS product DNA packaging tegument protein UL17 76918-76954 9:06:02
CCATGCCGGCCTGCCGGCCGCCGCCGTCGCCGCGGCC
76994 77008 76332 78437 CDS product DNA packaging tegument protein UL17 76994-77008 4:01:01 CCGCCGCTGCCGCCC
77020 77041 76332 78437 CDS product DNA packaging tegument protein UL17 77020-77041 4:01:01
CCGCCCGGAGCGCCTGCTGGCC
77059 77078 76332 78437 CDS product DNA packaging tegument protein UL17 77059-77078 4:01:01
CCCCGCCGCGGTCGCCCGCC
77223 77275 76332 78437 CDS product DNA packaging tegument protein UL17 77223-77275 13:10:03
CCGCCGCCGCCTGCGCCGCCACCGCGCCGGTCCTGCCTCCCGACGCCGCGACC
77293 77346 76332 78437 CDS product DNA packaging tegument protein UL17 77293-77346 9:06:02
GGAGCCGCGGGCCGCCGGGCCCGGACTTGGCGCGGTCGCGCGGGCGCTGGCGGG
77297 77314 76332 78437 CDS product DNA packaging tegument protein UL17 77297-77314 4:01:01
CCGCGGGCCGCCGGGCCC
77536 77578 76332 78437 CDS product DNA packaging tegument protein UL17 77536-77578 8:05:02
GGCGGGCTGGGGCGGGCGCCGGGCGCCCGGCCCGGCCCGCCGG
77554 77581 76332 78437 CDS product DNA packaging tegument protein UL17 77554-77581 6:03:01

CCGGGCGCCCGGCCCGGCCCGCCGGCCC

77692 77715 76332 78437 CDS product DNA packaging tegument protein UL17 77692-77715 5:02:01 GGCGGCGCCGCTGGGCGAGGACGG

77823 77849 76332 78437 CDS product DNA packaging tegument protein UL17 77823-77849 5:02:01 CCCGCCCGGACCTACCCCAGCGGTTCC

77876 77899 76332 78437 CDS product DNA packaging tegument protein UL17 77876-77899 5:02:01 GGATGTCTGGAGGACGTGGCGCGG

77938 77957 76332 78437 CDS product DNA packaging tegument protein UL17 77938-77957 4:01:01 GGGGGCGCTCTCGGCCCGGG

77983 77993 76332 78437 CDS product DNA packaging tegument protein UL17 77983-77993 4:01:01 GGCGGCGGTGG

78013 78036 76332 78437 CDS product DNA packaging tegument protein UL17 78013-78036 4:01:01 CCCCGGCGGCCGGGGCGTCCTGCC

78107 78125 76332 78437 CDS product DNA packaging tegument protein UL17 78107-78125 4:01:01 GGCGGGGAGGTCATAAAGG

78145 78180 76332 78437 CDS product DNA packaging tegument protein UL17 78145-78180 7:04:01 GGCCACGACGGCGCAGGAGTCCGGGCGGCGGCCGGG

78324 78350 76332 78437 CDS product DNA packaging tegument protein UL17 78324-78350 4:01:01 CCCTGGAGGCCGACGTCGCCGCGCACC

78376 78396 76332 78437 CDS product DNA packaging tegument protein UL17 78376-78396 5:02:01 GGCGGTGCTCGGGCGGCTGGG

78397 78432 76332 78437 CDS product DNA packaging tegument protein UL17 78397-78432 8:05:02 CCGCCCCCTCCCCGCGCCCCCAGGCGCGCCGGCCCC

78826 78859 78471 79490 CDS product tegument protein UL16 78826-78859 7:04:01

CCCCGCGCCGGCCGACCTCCCCGACCCGAGCGCC
78955 78973 78471 79490 CDS product tegument protein UL16 78955-78973 4:01:01 GGCGCCGGGCGTGTGGTGG
79061 79078 78471 79490 CDS product tegument protein UL16 79061-79078 4:01:01 GGGGGCGTGCTCGGGCGG
79174 79193 78471 79490 CDS product tegument protein UL16 79174-79193 4:01:01 CCCGTGCGCCGCGCCGTGCC
79343 79390 78471 79490 CDS product tegument protein UL16 79343-79390 8:05:02
GGCGTCGGGCCGCGCGGCGAAGCGGTGCCGGTGAACGGCGCCGGCTGG
79729 79764 75077 80655 CDS product DNA packaging terminase subunit 1 79729-79764 7:04:01
CCGCCGAACCACTGCCGGAGCCGCGCCACGATCTCC
79773 79791 75077 80655 CDS product DNA packaging terminase subunit 1 79773-79791 4:01:01
GGGCTCGGTGGCTTTGCGG
79873 79912 75077 80655 CDS product DNA packaging terminase subunit 1 79873-79912 7:04:01
CCGTGCCGCCGTGGTACCAGGAACACCGTGGCCCGCTGCC
80106 80122 75077 80655 CDS product DNA packaging terminase subunit 1 80106-80122 5:02:01
GGCGCGGGGGGGGTCGG
80158 80176 75077 80655 CDS product DNA packaging terminase subunit 1 80158-80176 4:01:01
CCCACCAGCCGGCTGAACC
80399 80416 75077 80655 CDS product DNA packaging terminase subunit 1 80399-80416 4:01:01
GGCGAGAGGCCGTGGCGG
80477 80500 75077 80655 CDS product DNA packaging terminase subunit 1 80477-80500 5:02:01
GGTGGCGCTTGGGGGCCGCAACGG
80531 80577 75077 80655 CDS product DNA packaging terminase subunit 1 80531-80577 9:06:02
CCTCCCCGCGGGCGCCGCCGCCGCCGTCGGGCCGCGCTCCCCGTTCC
80719 80755 80718 81386 CDS product tegument protein UL14 80719-80755 6:03:01

GGCGACGGCGGCGCTCGCGGGAGACCCGGCGCCGGGG
80775 80792 80718 81386 CDS product tegument protein UL14 80775-80792 5:02:01 GGCGGCGGCTGCGGCTGG
80852 80873 80718 81386 CDS product tegument protein UL14 80852-80873 4:01:01 GGCGCGGACCGGGACGACCCGG
80920 80942 80718 81386 CDS product tegument protein UL14 80920-80942 5:02:01 GGGGGGGCAGATCCGGGCGGCGG
80994 81025 80718 81386 CDS product tegument protein UL14 80994-81025 6:03:01
CCGCCCAAGCGGCCGTGGCCGCCGTGCTGGCC
81091 81111 80718 81386 CDS product tegument protein UL14 81091-81111 4:01:01 GGACCAGGAGGAGCGCATGGG
81124 81170 80718 81386 CDS product tegument protein UL14 81124-81170 9:06:02
GGACTGCGGGGGCGACGTGGGCGTGGGCGGGGCCTGGCTGGACGGGG
81263 81278 80718 81386 CDS product tegument protein UL14 81263-81278 4:01:01 GGAGGGCTCGCGGCGG
81305 81337 80718 81386 CDS product tegument protein UL14 81305-81337 8:05:02
GGCTCGGCGGCAGCGGCGGCGGGCCAGGCTGGG
81365 81395 81301 82779 CDS product tegument serine/threonine protein kinase 81365-81395 7:04:01
CCGCCCCACAGCACCCCCTGACCCCCGCGCC
81408 81425 81301 82779 CDS product tegument serine/threonine protein kinase 81408-81425 4:01:01
CCCCCGCTCCGGCGCGCC
81463 81492 81301 82779 CDS product tegument serine/threonine protein kinase 81463-81492 7:04:01
CCGCCCTCGCCGCCTCGGGCCGCCGGATCC
81501 81529 81301 82779 CDS product tegument serine/threonine protein kinase 81501-81529 6:03:01
CCGCGCGCCGATCCGCCCCGCGAGCCGCC
81660 81677 81301 82779 CDS product tegument serine/threonine protein kinase 81660-81677 4:01:01
GGGGCCGGCGGCTACGGG
81697 81719 81301 82779 CDS product tegument serine/threonine protein kinase 81697-81719 4:01:01

CCGCCGGCGTGGCCGTAAAGACC

81808 81826 81301 82779 CDS product tegument serine/threonine protein kinase 81808-81826 4:01:01 CCGACGCCATCATCCGGCC

81878 81908 81301 82779 CDS product tegument serine/threonine protein kinase 81878-81908 7:04:01 GGACCTGGTGGCCTACGCGGAGGCGGCGGGG

81913 81929 81301 82779 CDS product tegument serine/threonine protein kinase 81913-81929 4:01:01 CCGTCCTGTCGCCCGCC

82205 82234 81301 82779 CDS product tegument serine/threonine protein kinase 82205-82234 6:03:01 CCTGCCGCCCGACGCCCGGATGCCAGACCC

82280 82305 81301 82779 CDS product tegument serine/threonine protein kinase 82280-82305 4:01:01 CCTCGACTTCCTTAACCGGCACGGCC

82316 82338 81301 82779 CDS product tegument serine/threonine protein kinase 82316-82338 4:01:01 CCCCGAGCCGCTGCCCGCGGACC

82476 82505 81301 82779 CDS product tegument serine/threonine protein kinase 82476-82505 5:02:01 GGGCTGGTCATCGGCATTCTGGCGCACCGG

82580 82608 81301 82779 CDS product tegument serine/threonine protein kinase 82580-82608 5:02:01 GGCCGTGCGGGCGGCCATCGGCAACGTGG

82613 82637 81301 82779 CDS product tegument serine/threonine protein kinase 82613-82637 4:01:01 CCGCGCGGCCTTCGACCGCACTACC

82887 82913 82776 84239 CDS product deoxyribonuclease 82887-82913 6:03:01 GGCCGAGGCGGCGGGCGAAGGCGACGG

82990 83008 82776 84239 CDS product deoxyribonuclease 82990-83008 4:01:01 GGCGCGCGGCGCCCGGGGG

83028 83050 82776 84239 CDS product deoxyribonuclease 83028-83050 5:02:01 CCGCGACCCGCGGCCAGGCCGCC

83061 83125 82776 84239 CDS product deoxyribonuclease 83061-83125 10:07:02

GGTGGCTGCTCCGGCGGAGCCTGGCCACGGCCTCGTCGGTCCGCTGGGGCGCCGCCGGCCCGCGG

83071 83092 82776 84239 CDS product deoxyribonuclease 83071-83092 4:01:01 CCGGCGGAGCCTGGCCACGGCC

83112 83127 82776 84239 CDS product deoxyribonuclease 83112-83127 4:01:01 CCGCCGGCCCGCGGCC

83236 83260 82776 84239 CDS product deoxyribonuclease 83236-83260 5:02:01 CCCGCCCGGCGACCTCCCAACGCCC

83264 83284 82776 84239 CDS product deoxyribonuclease 83264-83284 6:03:01 GGGCTGGCGGACGGCGGGGGG

83326 83339 82776 84239 CDS product deoxyribonuclease 83326-83339 4:01:01 GGCCGGGGCGGCGG

83350 83369 82776 84239 CDS product deoxyribonuclease 83350-83369 5:02:01 GGCGGGGGCGGGCGCAGAGG

83376 83397 82776 84239 CDS product deoxyribonuclease 83376-83397 4:01:01 CCTGCGGCCTCCTGATCGACCC

83468 83485 82776 84239 CDS product deoxyribonuclease 83468-83485 4:01:01 CCGCATCCCACCCAGACC

83604 83629 82776 84239 CDS product deoxyribonuclease 83604-83629 5:02:01 CCTTCCTGCGGTCCGTGCGCCGCCCC

83654 83681 82776 84239 CDS product deoxyribonuclease 83654-83681 4:01:01 GGCTGCCCCGGCGCCGCGGAAGCGCTGG

83691 83730 82776 84239 CDS product deoxyribonuclease 83691-83730 9:06:02

CCGACGCCTGGGCGCCGCCCCCGCCTGCCGGCGCCGCACC

83938 83965 82776 84239 CDS product deoxyribonuclease 83938-83965 4:01:01 CCTCGCGGGCCACTTCCCCGAGCGCGCC

84040 84053 82776 84239 CDS product deoxyribonuclease 84040-84053 4:01:01 GGGGCCGGGGGCGG

84171 84202 82776 84239 CDS product deoxyribonuclease 84171-84202 6:03:01 GGGCGGCCGTCAAGGAGTCATGGGACAGGCGG

84213 84229 84191 84460 CDS product myristylated tegument protein 84213-84229 4:01:01 CCGATGCCGCCGCAACC

84291 84327 84191 84460 CDS product myristylated tegument protein 84291-84327 6:03:01

CCTCGACGACCTGCAGGCCCTGACGGCCGGGGCCGCC

84306 84349 84191 84460 CDS product myristylated tegument protein 84306-84349 9:06:02

GGCCCTGACGGCCGGGGCCGCCGCGGGGGAGGAGGGCGGCGAGG

84414 84445 84191 84460 CDS product myristylated tegument protein 84414-84445 6:03:01

CCCCCCCGTAAAACCGTACCGGCCGCAGCGCC

84544 84574 84530 85846 CDS product envelope glycoprotein M 84544-84574 9:06:02 GGGGGGTGGGGAGCGGGGGGGGGGGCGGGGG
84585 84603 84530 85846 CDS product envelope glycoprotein M 84585-84603 4:01:01 GGGAGTGGGGGACCTCCGG
84614 84638 84530 85846 CDS product envelope glycoprotein M 84614-84638 6:03:01 GGTATGGTTGGCGGGCGGGGCGGGG
84813 84827 84530 85846 CDS product envelope glycoprotein M 84813-84827 4:01:01 GGTGGTGCAGGCAGG
84853 84878 84530 85846 CDS product envelope glycoprotein M 84853-84878 4:01:01 GGCCACGGCCAGCAGGGCGACGCCGG
84875 84896 84530 85846 CDS product envelope glycoprotein M 84875-84896 4:01:01 CCGGCCAGCGCCAGCTTTACCC
84984 85008 84530 85846 CDS product envelope glycoprotein M 84984-85008 5:02:01 CCACCAGCGTTCCAAAGGCCGGCCC
85120 85135 84530 85846 CDS product envelope glycoprotein M 85120-85135 4:01:01 CCCCAGCCCGGCCCCC
85179 85195 84530 85846 CDS product envelope glycoprotein M 85179-85195 4:01:01 CCAGCAGGCCGCCGGCC
85210 85246 84530 85846 CDS product envelope glycoprotein M 85210-85246 6:03:01 GGCGCGCCCGGGGCCCAGCAGGCGGTGGGCGCGCGGG
85285 85304 84530 85846 CDS product envelope glycoprotein M 85285-85304 4:01:01 GGCGCCGGGCAGCCCGGCGG
85324 85364 84530 85846 CDS product envelope glycoprotein M 85324-85364 9:06:02 GGCGGCCGGCGGCGCCAGGTGCGCGAGGTAGGTGGCCGCGG
85414 85432 84530 85846 CDS product envelope glycoprotein M 85414-85432 4:01:01 CCAGGCCGCCAGCGCGCCC
85488 85503 84530 85846 CDS product envelope glycoprotein M 85488-85503 4:01:01 CCGCCGCAAAACCCCC
85582 85602 84530 85846 CDS product envelope glycoprotein M 85582-85602 4:01:01 GGAGAAGGCCGCTGTGGTCGG
85634 85669 84530 85846 CDS product envelope glycoprotein M 85634-85669 6:03:01 CCGAGCTCCCGCTGCGCCCAGGCGCCGCCGTCGGCC
85819 85835 84530 85846 CDS product envelope glycoprotein M 85819-85835 4:01:01 GGCGGCAGGCTGCGCGG
85849 85877 85845 88424 CDS product DNA replication origin-binding helicase 85849-85877 5:02:01 GGCAACGCCGGGGGCAGCGGCCGATGCGG

85990 86005 85845 88424 CDS product DNA replication origin-binding helicase 85990-86005 4:01:01
CCGGCGCCACCCCGCC
86130 86156 85845 88424 CDS product DNA replication origin-binding helicase 86130-86156 6:03:01
GGTTGGGCGAGGCGCTGGGGGCCACGG
86278 86312 85845 88424 CDS product DNA replication origin-binding helicase 86278-86312 6:03:01
GGGCGCGGCCTACCGGCGGCTGCTGGTGCAAGTGG
86448 86492 85845 88424 CDS product DNA replication origin-binding helicase 86448-86492 7:04:01
GGCTGTTGCGGCGGTGCCCGCGGATTGTGGCGATGGACGCGACGG
86629 86661 85845 88424 CDS product DNA replication origin-binding helicase 86629-86661 7:04:01
GGCGGCGCGGCTTAAGGGGGACGCCGAGGACGG
87088 87109 85845 88424 CDS product DNA replication origin-binding helicase 87088-87109 6:03:01
GGTGGCTGAGGACGGCGGCTGG
87376 87397 85845 88424 CDS product DNA replication origin-binding helicase 87376-87397 4:01:01
CCGACGGCCCGCGGCCGCGGCC
87414 87432 85845 88424 CDS product DNA replication origin-binding helicase 87414-87432 4:01:01
CCGCCGGCATCCCGTCCCC
87433 87452 85845 88424 CDS product DNA replication origin-binding helicase 87433-87452 4:01:01
GGTGTCGGCCGAGGGCCTGG
87441 87475 85845 88424 CDS product DNA replication origin-binding helicase 87441-87475 7:04:01
CCGAGGGCCTGGCCGAGCACCCGGCCGTGGCCGCC
87608 87626 85845 88424 CDS product DNA replication origin-binding helicase 87608-87626 5:02:01
CCCGCCGCCGCCGAAAGCC
87674 87697 85845 88424 CDS product DNA replication origin-binding helicase 87674-87697 4:01:01

CCCGTGCTCACCGAGGCCGGCGCC
87706 87733 85845 88424 CDS product DNA replication origin-binding helicase 87706-87733 5:02:01 GGCGGCTTTGGCGCCCGGGCTGCGCTGG
87766 87804 85845 88424 CDS product DNA replication origin-binding helicase 87766-87804 7:04:01 GGCCCGGGCGCTGGGGCTGCTGCGGCGCCGCGGCGGCGG
87934 87993 85845 88424 CDS product DNA replication origin-binding helicase 87934-87993 9:06:02 GGCGGGCGTGATGGCCCGCGGGCCTGTGGTCGCTGTGGCCGCGCGGCTTTCTGGGCGGGG
88003 88028 85845 88424 CDS product DNA replication origin-binding helicase 88003-88028 5:02:01 GGGGCGGGGCGTGGGGCTGGGCGCGG
88062 88084 85845 88424 CDS product DNA replication origin-binding helicase 88062-88084 4:01:01 GGGAAGAGGTGTTCGGGGCCCGG
88309 88342 85845 88424 CDS product DNA replication origin-binding helicase 88309-88342 6:03:01 CCCGGAGCCCGGGCCCGACGCGCCCCCCGAAGCC
88681 88697 88485 90731 CDS product helicase-primase subunit 88681-88697 4:01:01 GGCCGCGCGGCTGGAGG
88710 88724 88485 90731 CDS product helicase-primase subunit 88710-88724 5:02:01 CCGCCGCCGCCCCCC
88875 88911 88485 90731 CDS product helicase-primase subunit 88875-88911 7:04:01 CCGCCGAGGAGCCCCCCGATGCCGCCGACGCGGCCCC
88933 88985 88485 90731 CDS product helicase-primase subunit 88933-88985 10:07:02 GGCGCTACGGCTGGCCGTAGAGGAAGACGCGGTGCGCGCGGCGGCGGCGGCGG
89046 89062 88485 90731 CDS product helicase-primase subunit 89046-89062 4:01:01 CCGACCTCTCCGCGCCC
89121 89139 88485 90731 CDS product helicase-primase subunit 89121-89139 4:01:01 CCGACATCGCCCAGCCCCC
89142 89165 88485 90731 CDS product helicase-primase subunit 89142-89165 5:02:01 GGAAGCGGGCCGGGCGCCTGGCGG
89277 89296 88485 90731 CDS product helicase-primase subunit 89277-89296 4:01:01 CCTTTTCGCCGATGGCCGCC

89339 89368 88485 90731 CDS product helicase-primase subunit 89339-89368 5:02:01 GGACCGGCGCAGGTCCTCGGCTTTCTGGGG
89448 89464 88485 90731 CDS product helicase-primase subunit 89448-89464 4:01:01 CCGCCGACGCCGGCGCC
89623 89655 88485 90731 CDS product helicase-primase subunit 89623-89655 5:02:01 GGGCGGCAGCGACGGGTCAGATTGGCCCGCGGG
89727 89750 88485 90731 CDS product helicase-primase subunit 89727-89750 4:01:01 CCTTTTTTCCCACCCTGTACGCCC
89965 90005 88485 90731 CDS product helicase-primase subunit 89965-90005 6:03:01 GGATGGCTTTTGGGGCGCCTTCGGGGACGCGGCGCCCGAGG
90294 90316 88485 90731 CDS product helicase-primase subunit 90294-90316 4:01:01 CCGTCGCCCAGCCCGAGCGCCCC
90333 90346 88485 90731 CDS product helicase-primase subunit 90333-90346 4:01:01 CCGCCGCCGCGGCC
90469 90490 88485 90731 CDS product helicase-primase subunit 90469-90490 4:01:01 GGACGCGGACCACGGCCCCCGG
90478 90492 88485 90731 CDS product helicase-primase subunit 90478-90492 4:01:01 CCACGGCCCCCGGCC
90505 90521 88485 90731 CDS product helicase-primase subunit 90505-90521 4:01:01 GGCGGGCCACGAGGCGG
90942 90965 90784 91683 CDS product tegument protein UL7 90942-90965 6:03:01 CCCGCCGCGCCGCGCCGCGCCGCC
91269 91321 90784 91683 CDS product tegument protein UL7 91269-91321 9:06:02 GGGGCGGCACAAGGCCAGCCGGTACGGCAGCACGGCAGCGGGCACGGTAGTGG
91341 91360 90784 91683 CDS product tegument protein UL7 91341-91360 4:01:01 GGCGGTGAGCACGGCGAAGG
91370 91384 90784 91683 CDS product tegument protein UL7 91370-91384 4:01:01 GGAAGGCCGGCATGG
91507 91530 90784 91683 CDS product tegument protein UL7 91507-91530 4:01:01 GGCGGCCCTGCTGGGATCTCGCGG
91716 91741 91640 93706 CDS product capsid portal protein 91716-91741 5:02:01 GGCTGCGGCTGCGGCTGCGGCTGCGG
91764 91789 91640 93706 CDS product capsid portal protein 91764-91789 5:02:01 GGCTGCGGCTGCGGCTGCGGCTGCGG
91926 91948 91640 93706 CDS product capsid portal protein 91926-91948 6:03:01 CCTCCTCCGACCTGCACGCCTCC
92003 92024 91640 93706 CDS product capsid portal protein 92003-92024 4:01:01 GGCGCCAGGATCAAGGAGATGG

92043 92059 91640 93706 CDS product capsid portal protein 92043-92059 4:01:01 CCGACACCTCCTGGCCC

92103 92131 91640 93706 CDS product capsid portal protein 92103-92131 5:02:01 CCTGCTCCCACAGCCGCGAGAGCCGCTCC

92227 92255 91640 93706 CDS product capsid portal protein 92227-92255 6:03:01 CCCCAATTCCGCCGTCCGCGGGGCCCGCC

92265 92293 91640 93706 CDS product capsid portal protein 92265-92293 7:04:01 CCCGGGCCGGCCGGCGGCCCGCCCCCCCC

92268 92281 91640 93706 CDS product capsid portal protein 92268-92281 4:01:01 GGGCCGGCCGGCGG

92300 92319 91640 93706 CDS product capsid portal protein 92300-92319 4:01:01 GGCGTCGGCCGCTTGGGCGG

92377 92393 91640 93706 CDS product capsid portal protein 92377-92393 4:01:01 GGCCAGGTCGCGGTTGG

92502 92541 91640 93706 CDS product capsid portal protein 92502-92541 9:06:02 CCGCCCCGCCGCCCGCGCGGCCGCCCGCCGCGCCAAATCC

92694 92712 91640 93706 CDS product capsid portal protein 92694-92712 4:01:01 CCGCGTCCTCGCCCTCACC

92728 92745 91640 93706 CDS product capsid portal protein 92728-92745 4:01:01 GGACGGCGCGCCCGGCGG

92770 92785 91640 93706 CDS product capsid portal protein 92770-92785 4:01:01 CCGCCCCACCAGCACC

92847 92867 91640 93706 CDS product capsid portal protein 92847-92867 4:01:01 CCAGGACCTCGCCGTGCAGCC

93063 93086 91640 93706 CDS product capsid portal protein 93063-93086 5:02:01 CCTCGCATTCCGCCAGCACCCGCC

93124 93156 91640 93706 CDS product capsid portal protein 93124-93156 6:03:01 GGGAAGCGGCCCCCGGCGCCTGGACGGTGCCGG

93153 93176 91640 93706 CDS product capsid portal protein 93153-93176 4:01:01 CCGGCACCGCGAGGCCGATGCGCC

93219 93242 91640 93706 CDS product capsid portal protein 93219-93242 4:01:01 GGGCGAAGCTGGCCGGGCTGCCGG

93231 93250 91640 93706 CDS product capsid portal protein 93231-93250 4:01:01 CCGGGCTGCCGGCGCCCCCC

93333 93369 91640 93706 CDS product capsid portal protein 93333-93369 7:04:01

CCGCGAGCCGCACCACCTCTGCCTCGCCGCGCGGGCC

93424 93442 91640 93706 CDS product capsid portal protein 93424-93442 4:01:01 CCAATCCCGCGCCACGTCC

93607 93640 91640 93706 CDS product capsid portal protein 93607-93640 5:02:01 CCGCACCCACTCGCCCGGCGGCCCCGGAGCGCCC

93623 93643 91640 93706 CDS product capsid portal protein 93623-93643 4:01:01 GGCGGCCCCGGAGCGCCCGGG

93831 93874 93756 96272 CDS product helicase-primase helicase subunit 93831-93874 9:06:02 GGATCCGGCAGCTGGAGGCCGCGCGGCTGCCGGCGGCGGCCCGG

93892 93918 93756 96272 CDS product helicase-primase helicase subunit 93892-93918 4:01:01 CCGCGCGGCCGCGGCGGCCGAGTCCCC

94080 94097 93756 96272 CDS product helicase-primase helicase subunit 94080-94097 5:02:01 CCGCCGCCTACCACAGCC

94185 94202 93756 96272 CDS product helicase-primase helicase subunit 94185-94202 4:01:01 CCAGCCCGCCCAGCATCC

94260 94277 93756 96272 CDS product helicase-primase helicase subunit 94260-94277 4:01:01 GGCGGCTGCTGGGCGCGG

94312 94330 93756 96272 CDS product helicase-primase helicase subunit 94312-94330 4:01:01 GGCGGAGCTCGCGGCGGGG

94420 94438 93756 96272 CDS product helicase-primase helicase subunit 94420-94438 4:01:01 GGCCGGGCTGCTGGGCCGG

94621 94645 93756 96272 CDS product helicase-primase helicase subunit 94621-94645 5:02:01 CCTCACCTGCCTTATCACCAACCCC

94888 94904 93756 96272 CDS product helicase-primase helicase subunit 94888-94904 4:01:01 CCGCCTCCACGCGCACC

94990 95010 93756 96272 CDS product helicase-primase helicase subunit 94990-95010 5:02:01
CCGCACCGCCACGGGCCAGCC
95002 95049 93756 96272 CDS product helicase-primase helicase subunit 95002-95049 8:05:02
GGGCCAGCCGGGGCTGGGGGTGGAAAAATGGCTGCAGGCCAACTCGGG
95074 95097 93756 96272 CDS product helicase-primase helicase subunit 95074-95097 4:01:01
CCGCGACCAGGACGCCCAGTCACC
95360 95378 93756 96272 CDS product helicase-primase helicase subunit 95360-95378 4:01:01
CCCGGCCTGGACCCGGGCC
95363 95384 93756 96272 CDS product helicase-primase helicase subunit 95363-95384 4:01:01
GGCCTGGACCCGGGCCGCGTGG
95440 95474 93756 96272 CDS product helicase-primase helicase subunit 95440-95474 8:05:02
GGACGACGCGGTCGACTGGCGGGGGCTGGCGGCGG
95486 95507 93756 96272 CDS product helicase-primase helicase subunit 95486-95507 4:01:01
GGCTCTGAGGCCGGCGCCGGGG
95581 95600 93756 96272 CDS product helicase-primase helicase subunit 95581-95600 4:01:01
CCAGCGCCCTACGTCCGCCC
95762 95780 93756 96272 CDS product helicase-primase helicase subunit 95762-95780 4:01:01
GGCGGGCTGGTCTCCATGG
95842 95863 93756 96272 CDS product helicase-primase helicase subunit 95842-95863 5:02:01
GGAGGAGCCCGGCCGGCGCCGG
95849 95869 93756 96272 CDS product helicase-primase helicase subunit 95849-95869 4:01:01
CCCGGCCGGCGCCGGCTCCCC
95974 95993 93756 96272 CDS product helicase-primase helicase subunit 95974-95993 4:01:01

GGAGGCGCTGGACGACAAGG
96042 96074 93756 96272 CDS product helicase-primase helicase subunit 96042-96074 6:03:01
CCATGACCATCGCCCGCTCCCAGGGCCTGAGCC
96156 96183 93756 96272 CDS product helicase-primase helicase subunit 96156-96183 4:01:01
CCCGATTTCTCCGAATGAACCTCAACCC
96294 96318 96293 96850 CDS product nuclear protein UL4 96294-96318 4:01:01 GGCGCTCGCGGCCCCCGGGCCCGGG
96362 96382 96293 96850 CDS product nuclear protein UL4 96362-96382 4:01:01 CCCCCTTGTGGGCCTTGCCCC
96414 96448 96293 96850 CDS product nuclear protein UL4 96414-96448 8:05:02
GGGGGATCGCTCGGTGTCGGTGGGCGGCGGGCTGG
96739 96759 96293 96850 CDS product nuclear protein UL4 96739-96759 5:02:01 CCGGCGACCGCGCCGCCCCCC
96812 96834 96293 96850 CDS product nuclear protein UL4 96812-96834 4:01:01 CCCGCCCCTCGGGCTCCCGCCCC
96918 96973 96914 97294 CDS product protein V57 96918-96973 12:09:03
GGAACGTGCGGTAATTGGGGGGGAAAGGGCGCGTCGGGGGCGGCGGTGGGCGGCGG
97026 97057 96914 97294 CDS product protein V57 97026-97057 6:03:01 GGCGCGCGGCCCGGGCCTCCGCGGGCGGCAGG
97164 97183 96914 97294 CDS product protein V57 97164-97183 4:01:01 CCCCGACCGCGGCGGCCGCC
97201 97214 96914 97294 CDS product protein V57 97201-97214 4:01:01 GGCGGCGCGGTTGG
97236 97263 96914 97294 CDS product protein V57 97236-97263 7:04:01 CCGCCACCGTGGCCACCCGGGCCTCCCC
97548 97573 97297 97911 CDS product nuclear protein UL3 97548-97573 5:02:01 CCGCGCGCCGCAGCCGCGCCAGGGCC
97756 97788 97297 97911 CDS product nuclear protein UL3 97756-97788 7:04:01
CCCAGACCCGCCGCTGCCAGGCCGGGCCCGGCC
97821 97852 97297 97911 CDS product nuclear protein UL3 97821-97852 8:05:02 GGCGGGGGCAGAAGCGGAAGCGGCGGCGGCGG
97863 97878 97297 97911 CDS product nuclear protein UL3 97863-97878 5:02:01 CCAGCCGCCGGCCGCC
98089 98114 97958 98863 CDS product uracil DNA glycosylase 98089-98114 5:02:01 CCGCTTCCCGGCCGCCCCAAAGGCCC

| | | | | | | | |
|---|---|---|---|---|---|---|---|
| 98175 | 98206 | 97958 | 98863 | CDS product uracil DNA glycosylase | 98175-98206 | 7:04:01 | CCAGTACCGCGTGCACCAGCCGCCCCCAGCCC |
| 98355 | 98373 | 97958 | 98863 | CDS product uracil DNA glycosylase | 98355-98373 | 5:02:01 | GGAGGCTGGGTGGGACCGG |
| 98692 | 98717 | 97958 | 98863 | CDS product uracil DNA glycosylase | 98692-98717 | 5:02:01 | GGGGCGCCGGCGCTTGGGCGGCGCGG |
| 98758 | 98855 | 97958 | 98863 | CDS product uracil DNA glycosylase | 98758-98855 | 17:14:04 | CCGAAGCCGGGGCCGAAGCCGGGGCCGAAGCCGGGGCCGAAGCCGGGGCCGAAGCCGGGGCCGAAGCCGGGGCCGAAGCCGGGGCCGGGGCCGGGGCC |
| 98838 | 98859 | 97958 | 98863 | CDS product uracil DNA glycosylase | 98838-98859 | 4:01:01 | GGGGCCGGGGCCGGGGCCGGGG |
| 98878 | 98900 | 98866 | 99342 | CDS product envelope glycoprotein L | 98878-98900 | 4:01:01 | CCGTCGCCAGGGCCGCAGGGACC |
| 98909 | 98925 | 98866 | 99342 | CDS product envelope glycoprotein L | 98909-98925 | 4:01:01 | GGTATCGGGGCGGCAGG |
| 98938 | 98954 | 98866 | 99342 | CDS product envelope glycoprotein L | 98938-98954 | 5:02:01 | GGCGGCGGTGGCAGCGG |
| 99160 | 99180 | 98866 | 99342 | CDS product envelope glycoprotein L | 99160-99180 | 5:02:01 | CCCGCCAGGCCCGGGGCCGCC |
| 101124 | 101145 | 100670 | 102700 | CDS product ubiquitin E3 ligase ICP0 | 101124-101145 | 5:02:01 | CCGCCGCCGCGGCCGGGGTCCC |
| 101156 | 101191 | 100670 | 102700 | CDS product ubiquitin E3 ligase ICP0 | 101156-101191 | 7:04:01 | CCCGCCGAGGCCGGCCCCGGGGCCGCCGCGGGGGCC |
| 101164 | 101211 | 100670 | 102700 | CDS product ubiquitin E3 ligase ICP0 | 101164-101211 | 10:07:02 | GGCCGGCCCCGGGGCCGCCGCGGGGGCCGGGTCGGCGGGGCGGGCGGG |
| 101239 | 101290 | 100670 | 102700 | CDS product ubiquitin E3 ligase ICP0 | 101239-101290 | 10:07:02 | GGAGATGGGCGCGGGGGCTGGGGCCGGGGCCGGGGCCGGGGTCGGGGCGCGG |
| 101335 | 101401 | 100670 | 102700 | CDS product ubiquitin E3 ligase ICP0 | 101335-101401 | 12:09:03 | CCCAGCCTCCCCGAGCAGCCTGGCCCGGCGCTCCCCTCGTCCCTCCTCACTGCCCGCCCCCCAGCCC |
| 101411 | 101443 | 100670 | 102700 | CDS product ubiquitin E3 ligase ICP0 | 101411-101443 | 6:03:01 | CCCGCCGCGACTCCCAAGCCCGCTCCCCTCCCC |

101498 101518 100670 102700 CDS product ubiquitin E3 ligase ICP0 101498-101518 4:01:01
GGCGCCGCCGGCAATCGGGGG

101658 101705 100670 102700 CDS product ubiquitin E3 ligase ICP0 101658-101705 9:06:02
CCGCAGGCCGCCGAGCCCGGGCCGCGCCAGACGCCCCCCGCGTGCGCC

101715 101743 100670 102700 CDS product ubiquitin E3 ligase ICP0 101715-101743 6:03:01
CCTCCGCGTCCGTCTCCGGCTCCGTTTCC

101755 101771 100670 102700 CDS product ubiquitin E3 ligase ICP0 101755-101771 5:02:01 GGCGGTCAGGTCGGAGG

101817 101842 100670 102700 CDS product ubiquitin E3 ligase ICP0 101817-101842 6:03:01
CCTCCGAGCCCTCCGAGTCCGAGTCC

101859 101874 100670 102700 CDS product ubiquitin E3 ligase ICP0 101859-101874 4:01:01 CCACCCCACGGCCGCC

102095 102131 100670 102700 CDS product ubiquitin E3 ligase ICP0 102095-102131 8:05:02
CCCACCAGAGCGCCCTCGACCCACTCCACCACCCGCC

102144 102160 100670 102700 CDS product ubiquitin E3 ligase ICP0 102144-102160 4:01:01 CCCCCGGCCCATGCTCC

102161 102190 100670 102700 CDS product ubiquitin E3 ligase ICP0 102161-102190 5:02:01
GGCGTGTTCGGCAGCAAGGGCAGGCGCGGG

102216 102391 100670 102700 CDS product ubiquitin E3 ligase ICP0 102216-102391 36:33:09
CCTGCGCCGCGTCCGCGCCTCGCCCCGCGCGCCCCCCGGCCGCTCCGGCCGCCCTGCCCGCCCCGGCGCCTCTGGCCTCCCCGGCCTCCTCCGCCCCCCCGGCCCCGCCGCCGGCCTCGCTCCCGTCC
GCGGCCCCGGCTTCCCCGCCGGCCCCCGAGGCCTCGCCGTCGGCCGCC

102438 102481 100670 102700 CDS product ubiquitin E3 ligase ICP0 102438-102481 10:07:02
CCGCGTCCGGCTCCAACGCGCCGTCCGCCCCCGGCCCTCCCCCC

102568 102581 100670 102700 CDS product ubiquitin E3 ligase ICP0 102568-102581 4:01:01 CCCCTCCAGCCACC

102582 102618 100670 102700 CDS product ubiquitin E3 ligase ICP0 102582-102618 7:04:01
GGCGGATGCAGGCCAGGCAGAAGGCGTGCAGGCAGGG

102677 102693 100670 102700 CDS product ubiquitin E3 ligase ICP0 102677-102693 4:01:01 GGGGCAGCGGCGGGCGG
103683 103708 103673 107704 CDS product transcriptional regulator ICP4 103683-103708 6:03:01
CCGGCCCGCCGCCGGCGGCGCCGGCC
103744 103778 103673 107704 CDS product transcriptional regulator ICP4 103744-103778 9:06:02
GGACGAGGACGAGGACGAGGAGGAGGAGGAGGAGG
103804 103870 103673 107704 CDS product transcriptional regulator ICP4 103804-103870 14:11:03
CCCGCGCCGGGGCCGCCGCGGCCGCCGGCCGCCGCCCGCCGGCGCCGGGAAGCCCGAGCCCGCGCCC
103886 103904 103673 107704 CDS product transcriptional regulator ICP4 103886-103904 4:01:01
GGCACCCGGGCCGGCGGGG
103932 103948 103673 107704 CDS product transcriptional regulator ICP4 103932-103948 5:02:01
CCACCAGGCCGCCGCCC
103977 104023 103673 107704 CDS product transcriptional regulator ICP4 103977-104023 8:05:02
CCCGCGGGCCCTGGTCCGCGTCCCAGTCCACGCCGGGCGCCGCGGCC
104011 104031 103673 107704 CDS product transcriptional regulator ICP4 104011-104031 4:01:01
GGGCGCCGCGGCCGCGGGCGG
104034 104053 103673 107704 CDS product transcriptional regulator ICP4 104034-104053 5:02:01
CCGCCGCGGCCTCCAGCACC
104057 104090 103673 107704 CDS product transcriptional regulator ICP4 104057-104090 6:03:01
GGCGGCGCCTCGGCGCGCGGCTCCGGCAGCGCGG
104103 104116 103673 107704 CDS product transcriptional regulator ICP4 104103-104116 4:01:01 CCACCGCCGCGGCC
104149 104174 103673 107704 CDS product transcriptional regulator ICP4 104149-104174 5:02:01
CCGCCCGCAGGCCAGGTACACCGGCC
104158 104181 103673 107704 CDS product transcriptional regulator ICP4 104158-104181 4:01:01

GGCCAGGTACACCGGCCGCAGCGG
104199 104219 103673 107704 CDS product transcriptional regulator ICP4 104199-104219 6:03:01
GGTTGGCGGCGCGGTGGCTGG
104220 104292 103673 107704 CDS product transcriptional regulator ICP4 104220-104292 12:09:03
CCGCCTCGCCCTCCACGAAGTCCGGCTCCCCGAGCCCCAGCGCCGCGCCCTGCGCGGCCATGTCCTTGCGCCC
104330 104369 103673 107704 CDS product transcriptional regulator ICP4 104330-104369 7:04:01
GGCGGCACGGGCACCGCGGTGCGCGGGCCCAGGCGCGTGG
104577 104595 103673 107704 CDS product transcriptional regulator ICP4 104577-104595 4:01:01
CCTGCCCGACGGCCGGGCC
104641 104663 103673 107704 CDS product transcriptional regulator ICP4 104641-104663 6:03:01
CCGCCGCCGCGCCGCGGCCAGCC
104751 104777 103673 107704 CDS product transcriptional regulator ICP4 104751-104777 5:02:01
CCGGCGCGCCCGTCCAGCGCCCGGCCC
104847 104872 103673 107704 CDS product transcriptional regulator ICP4 104847-104872 5:02:01
GGCGCGGCGGCCACTCGGGCCGCCGG
104895 104910 103673 107704 CDS product transcriptional regulator ICP4 104895-104910 4:01:01 CCGCCAGCGCCTCCCC
104971 104987 103673 107704 CDS product transcriptional regulator ICP4 104971-104987 4:01:01
CCAGGCCACGCGCCGCC
105013 105032 103673 107704 CDS product transcriptional regulator ICP4 105013-105032 4:01:01
GGGGCCCGCGCGGCGGCGGG
105074 105103 103673 107704 CDS product transcriptional regulator ICP4 105074-105103 5:02:01
GGGCGCCAGGGCTCGGGGAAGAGCGGGTGG
105105 105124 103673 107704 CDS product transcriptional regulator ICP4 105105-105124 4:01:01

CCGCGAGCCGCGCCGCGACC
105154 105175 103673 107704 CDS product transcriptional regulator ICP4 105154-105175 4:01:01
GGCGCTGGGCGCGGGCGTGTGG
105184 105199 103673 107704 CDS product transcriptional regulator ICP4 105184-105199 4:01:01 GGGCGGCACGCGGCGG
105211 105237 103673 107704 CDS product transcriptional regulator ICP4 105211-105237 6:03:01
GGCGGCGGGGCCGCCGGGCGGCATGGG
105249 105264 103673 107704 CDS product transcriptional regulator ICP4 105249-105264 4:01:01 GGGCGGGCAGCGGCGG
105267 105286 103673 107704 CDS product transcriptional regulator ICP4 105267-105286 5:02:01
CCCGCCGCGCCGGCCCGGCC
105278 105302 103673 107704 CDS product transcriptional regulator ICP4 105278-105302 6:03:01
GGCCCGGCCGCGTCGGCGGCGGCGG
105333 105457 103673 107704 CDS product transcriptional regulator ICP4 105333-105457 26:23:06
CCTCCCCCGAGGGGCCCCCCGCGGCGGCCGGCAGGGCCGCCGCCTCGCCGTCCCCGTCCCCGTCCCCTTCTTCCTCCTCCTCCTCCTCCTCCTCCTCCTCGGCCCTTCTTCCTTCCCTCGGACCC
105355 105368 103673 107704 CDS product transcriptional regulator ICP4 105355-105368 4:01:01 GGCGGCCGGCAGGG
105467 105673 103673 107704 CDS product transcriptional regulator ICP4 105467-105673 44:41:11
CCCGCCGCCGGGCCGGGGCGGCCCTCTTCCTCCTCTTCCTCCTCTTCCTCCTCTTCCTCCTCTTCCTCCTTTTCCTCCTCTTCCTCCTCTTCCGCCTCTTCGTCCTCCCCGTCCTCCCCGTCCTCCGC
CTCGGACCCGTCCTCCGGGTCCGCCCGCGGCGCCCCGGCCCTCATGTCCTCCACCTCCACTTCCACCGGCCCCCGGCCC
105662 105695 103673 107704 CDS product transcriptional regulator ICP4 105662-105695 7:04:01
GGCCCCCGGCCCGGCGCGGCGGCGCCGGCGCCGG
105686 105721 103673 107704 CDS product transcriptional regulator ICP4 105686-105721 8:05:02
CCGGCGCCGGCGCCCCCGCCGGCGGCCTCCAGGGCC
105732 105757 103673 107704 CDS product transcriptional regulator ICP4 105732-105757 7:04:01
CCGCCAGCCGCGCCGGCACCTCCGCC

105840 105871 103673 107704 CDS product transcriptional regulator ICP4 105840-105871 7:04:01
CCACGGTCCGCGCCGCCAGCGCGGCCGCCTCC
105984 106006 103673 107704 CDS product transcriptional regulator ICP4 105984-106006 4:01:01
CCGGGTGCGCCGCCAGCGCGTCC
106007 106036 103673 107704 CDS product transcriptional regulator ICP4 106007-106036 6:03:01
GGCGCGCAGGCCGCGGCCGGCAGGCCGCGG
106017 106074 103673 107704 CDS product transcriptional regulator ICP4 106017-106074 9:06:02
CCGCGGCCGGCAGGCCGCGGCCCGCCGCGGCCGAGAGCACCGGGAGCCCGGCGGCGCC
106058 106088 103673 107704 CDS product transcriptional regulator ICP4 106058-106088 7:04:01
GGGAGCCCGGCGGCGCCGGCGGCGCGGCGGG
106089 106102 103673 107704 CDS product transcriptional regulator ICP4 106089-106102 4:01:01 CCGCCTCCAGCGCC
106107 106126 103673 107704 CDS product transcriptional regulator ICP4 106107-106126 4:01:01
GGCAGGCGACGGCGCAGCGG
106155 106174 103673 107704 CDS product transcriptional regulator ICP4 106155-106174 4:01:01
CCGCCCGCGCCGTGCTCGCC
106175 106191 103673 107704 CDS product transcriptional regulator ICP4 106175-106191 4:01:01
GGCGGCAGGGGCGCCGG
106244 106276 103673 107704 CDS product transcriptional regulator ICP4 106244-106276 7:04:01
CCGCTGCCGCCGGCGCCGGCCTCCGGGTAGGCC
106255 106283 103673 107704 CDS product transcriptional regulator ICP4 106255-106283 6:03:01
GGCGCCGGCCTCCGGGTAGGCCATGGGGG
106338 106358 103673 107704 CDS product transcriptional regulator ICP4 106338-106358 4:01:01
GGCGGCTCATGGCCACGCAGG

106566 106582 103673 107704 CDS product transcriptional regulator ICP4 106566-106582 4:01:01
CCAGGTCCACCAGCGCC
106594 106612 103673 107704 CDS product transcriptional regulator ICP4 106594-106612 4:01:01
CCCCGCGTCCCCCATCTCC
106626 106645 103673 107704 CDS product transcriptional regulator ICP4 106626-106645 4:01:01
CCGCGGGCCCCGCGGCCGCC
106728 106743 103673 107704 CDS product transcriptional regulator ICP4 106728-106743 5:02:01 GGCCGGGCGGCGGCGG
106751 106767 103673 107704 CDS product transcriptional regulator ICP4 106751-106767 4:01:01
CCGGGCCACGCCTCGCC
106860 106878 103673 107704 CDS product transcriptional regulator ICP4 106860-106878 5:02:01
GGCGCCGCGGCGGGGGGGG
106889 106943 103673 107704 CDS product transcriptional regulator ICP4 106889-106943 10:07:02
GGCGGCGAGGGCGCCGGGGGCCCGGGCGCGCGGCCCCGCGGGGCGCCGGGCCCGG
106934 106951 103673 107704 CDS product transcriptional regulator ICP4 106934-106951 4:01:01
CCGGGCCCGGCGCCCGCC
106958 106982 103673 107704 CDS product transcriptional regulator ICP4 106958-106982 4:01:01
GGGGCCGCTTTGGTTCTGGTCCCGG
106978 107014 103673 107704 CDS product transcriptional regulator ICP4 106978-107014 7:04:01
CCCGGCCCCAGTCCCGGCCTCAGCCTCGGCCTCGGCC
107035 107070 103673 107704 CDS product transcriptional regulator ICP4 107035-107070 7:04:01
CCCCTCCCGGGCCTCGCCCCCATCGTCCTCGGGCCC
107085 107109 103673 107704 CDS product transcriptional regulator ICP4 107085-107109 4:01:01
CCTCGGGCCCGCTGTCCTCGGACCC

107124 107200 103673 107704 CDS product transcriptional regulator ICP4 107124-107200 13:10:03
CCTCGGGCCCAACGTCGCCGGCCTCGGTCCTGGCCCCGAGGGCACCGCCGCCCCCCGCCTCAGTCCCCGCTTCGGCC
107143 107165 103673 107704 CDS product transcriptional regulator ICP4 107143-107165 4:01:01
GGCCTCGGTCCTGGCCCCGAGGG
107211 107221 103673 107704 CDS product transcriptional regulator ICP4 107211-107221 4:01:01 CCGCCCCCGCC
107224 107264 103673 107704 CDS product transcriptional regulator ICP4 107224-107264 7:04:01
GGCTTCGATGGCGGCGGCTATGACGGCCGCTTCGGCCGCGG
107250 107302 103673 107704 CDS product transcriptional regulator ICP4 107250-107302 12:09:03
CCGCTTCGGCCGCGGCCGCCTCCGCCCCGGCCGCGGCCGCCTCCACCGCGACC
107334 107365 103673 107704 CDS product transcriptional regulator ICP4 107334-107365 8:05:02
CCGCGTCCTCCGCGCCCTCCGCCCCCTCGGCC
107376 107437 103673 107704 CDS product transcriptional regulator ICP4 107376-107437 10:07:02
CCGCGACGTCCGCGTCCTCGGCGCCCCCCGCGTCCCTGGCGCCGGCGTCCTCGCCGATCTCC
107446 107465 103673 107704 CDS product transcriptional regulator ICP4 107446-107465 4:01:01
GGCCTCGGCGCCCTCGGTGG
107448 107491 103673 107704 CDS product transcriptional regulator ICP4 107448-107491 7:04:01
CCTCGGCGCCCTCGGTGGCCCCCGCGTCTCCGGCGCCGTCGTCC
107502 107528 103673 107704 CDS product transcriptional regulator ICP4 107502-107528 7:04:01
CCGCGTCCTCCCCGCCCACCATCCGCC
107536 107563 103673 107704 CDS product transcriptional regulator ICP4 107536-107563 5:02:01
GGCCAGCTCGGCGCGGGCGGCCCGCCGG
107604 107641 103673 107704 CDS product transcriptional regulator ICP4 107604-107641 7:04:01
CCGGGTCTTCCTCCGAGCCCCCCGTCGGGCCGAGGTCC

112804 112830 112768 113670 CDS product regulatory protein ICP22 112804-112830 6:03:01
CCTGCCGCCTCTGCCGCTCGCCCGACC
112871 112895 112768 113670 CDS product regulatory protein ICP22 112871-112895 4:01:01
CCGCGGCCCGGGCGCCTTCTGCCCC
113088 113112 112768 113670 CDS product regulatory protein ICP22 113088-113112 4:01:01
CCCGAGCAGGCCGGCCCGCTGCGCC
113125 113144 112768 113670 CDS product regulatory protein ICP22 113125-113144 6:03:01 CCGCCGCCGCCGCCGGGCCC
113153 113185 112768 113670 CDS product regulatory protein ICP22 113153-113185 6:03:01
CCCGCTGTCGCCCCCGGCGCGCCTGCCGGGCCC
113243 113292 112768 113670 CDS product regulatory protein ICP22 113243-113292 13:10:03
GGAGGACGAGGAGGCGGACGAGGAGGGGGAGGAGAACGGCGGCGAGGGGG
113293 113372 112768 113670 CDS product regulatory protein ICP22 113293-113372 17:14:04
CCGCCGCCGAAAGCCCCCCCCGGTGCCGCCCGCGAGCCGCGGTCCTCTCCTCCGCCTCGCCCGCGGCCTCGGCCGTCTCC
113385 113408 112768 113670 CDS product regulatory protein ICP22 113385-113408 6:03:01
CCCACCGCGTCCTCTTCCACCTCC
113462 113519 112768 113670 CDS product regulatory protein ICP22 113462-113519 10:07:02
CCCCGGGCCCGGGGACCCGCGCCCGGCCGGCGCCGGGCCCGGCGCCGGCGGCCCCCCC
113486 113512 112768 113670 CDS product regulatory protein ICP22 113486-113512 6:03:01
GGCCGGCGCCGGGCCCGGCGCCGGCGG
113531 113549 112768 113670 CDS product regulatory protein ICP22 113531-113549 4:01:01 CCGGCGCCGGCCCGCGCCC
113563 113602 112768 113670 CDS product regulatory protein ICP22 113563-113602 8:05:02
CCTCCGGCTCCTCGCCGGGCTCCTCGCCATACCCGTCGCC
113637 113658 112768 113670 CDS product regulatory protein ICP22 113637-113658 5:02:01

CCCGCGCCGGCCAAGCGCCGCC
114547 114566 114213 114944 CDS product virion protein V67 114547-114566 4:01:01 CCGCCAGCGGGGCCTCGTCC
114642 114658 114213 114944 CDS product virion protein V67 114642-114658 4:01:01 GGTCGGCGCGGCGCGGG
114730 114754 114213 114944 CDS product virion protein V67 114730-114754 4:01:01 GGGGCTTGGACGAGGGCACCACCGG
114824 114862 114213 114944 CDS product virion protein V67 114824-114862 8:05:02
CCAGCCACCAAGCCCCCCCTCGCATCCGGGACCGGTGCC
114851 114879 114213 114944 CDS product virion protein V67 114851-114879 6:03:01
GGGACCGGTGCCGGCGGTCGCGGTCGCGG
115118 115129 115001 115663 CDS product virion protein US2 115118-115129 4:01:01 CCACCGGCCACC
115136 115168 115001 115663 CDS product virion protein US2 115136-115168 5:02:01
GGGAGTGTCGGGGTATCGTAGGGCGGCCACGGG
115179 115213 115001 115663 CDS product virion protein US2 115179-115213 7:04:01
CCGGGCCGCGCCGCGGGAGCCGCCACGCCGCTCCC
115358 115384 115001 115663 CDS product virion protein US2 115358-115384 5:02:01 CCGTCGACCACGCTGACCCACTCCGCC
115385 115432 115001 115663 CDS product virion protein US2 115385-115432 9:06:02
GGTTGGCGGTGGCGGGCTAAGCCGGCGCCCCCGGGCGCGGGCCAAGGG
115530 115562 115001 115663 CDS product virion protein US2 115530-115562 5:02:01
CCGTCGTCCCCAGCCGGCTTGCCGTCAGCGCCC
115999 116021 115796 117202 CDS product serine/threonine protein kinase US3 115999-116021 6:03:01
CCGCCGGCCAGAACCGGTCCGCC
116014 116047 115796 117202 CDS product serine/threonine protein kinase US3 116014-116047 6:03:01
GGTCCGCCGGACGCGGACGGGATCGAAGGGGGGG
116473 116497 115796 117202 CDS product serine/threonine protein kinase US3 116473-116497 4:01:01

GGGGAGCTGGTGTGCGTGGTGCTGG
116562 116581 115796 117202 CDS product serine/threonine protein kinase US3 116562-116581 4:01:01
GGCGCTGGCGGTGACGCGGG
116595 116617 115796 117202 CDS product serine/threonine protein kinase US3 116595-116617 4:01:01
CCTCGCGTACCTGCACTCCCGCC
116682 116710 115796 117202 CDS product serine/threonine protein kinase US3 116682-116710 5:02:01
GGGCGACTTTGGCGCGGCACACGGGCCGG
116731 116749 115796 117202 CDS product serine/threonine protein kinase US3 116731-116749 4:01:01
GGCCTGGCCGGCACCCTGG
116871 116903 115796 117202 CDS product serine/threonine protein kinase US3 116871-116903 5:02:01
GGGCCCGCAGGGCGAGGACGCCGAGGCATCGGG
116989 117016 115796 117202 CDS product serine/threonine protein kinase US3 116989-117016 5:02:01
CCACCCAGCCCCACTGACCGGCTGACCC
117045 117073 115796 117202 CDS product serine/threonine protein kinase US3 117045-117073 5:02:01
CCGAGAGCCGCACAGCCCGTACCGCTGCC
117084 117118 115796 117202 CDS product serine/threonine protein kinase US3 117084-117118 7:04:01
CCGGCTGCCCTGCGACGCCGACCGCCTCCTACACC
117339 117381 117337 118671 CDS product envelope glycoprotein G 117339-117381 9:06:02
CCTGCCGCCCGGACCGGCACCTTGGCCGCCGTCGCCCTAATCC
117462 117500 117337 118671 CDS product envelope glycoprotein G 117462-117500 7:04:01
CCCTCCCGCCCGCTGGGGCCCGTCCTGAACCTAGCGGCC
117517 117537 117337 118671 CDS product envelope glycoprotein G 117517-117537 4:01:01 GGGTTTCGGTGCGCGCGGTGG
117560 117598 117337 118671 CDS product envelope glycoprotein G 117560-117598 8:05:02

GGCCCTCTTGGACATGGCGGAGACGGTGGTGCCCGGCGG

117591 117611 117337 118671 CDS product envelope glycoprotein G 117591-117611 4:01:01 CCCGGCGGACCGCGAGCCGCC

117803 117827 117337 118671 CDS product envelope glycoprotein G 117803-117827 4:01:01 CCCCGGCCTGTACGACCGCGGGACC

117918 117942 117337 118671 CDS product envelope glycoprotein G 117918-117942 5:02:01 GGGCTGGACCGAGGGCTCGGTGTGG

118232 118250 117337 118671 CDS product envelope glycoprotein G 118232-118250 4:01:01 CCGGACCGCCAGCGGGCCC

118406 118456 117337 118671 CDS product envelope glycoprotein G 118406-118456 9:06:02 CCAGGAGCCCGCTCGCCTCGAGCGCCGCCCCGATGCCCGCGCCACCGATCC

118475 118493 117337 118671 CDS product envelope glycoprotein G 118475-118493 4:01:01 CCTGCCCGATGACCCCGCC

118573 118595 117337 118671 CDS product envelope glycoprotein G 118573-118595 4:01:01 CCTGCCGGCTCGCCCGCGCAGCC

118612 118631 117337 118671 CDS product envelope glycoprotein G 118612-118631 4:01:01 CCCGCGCCGCCACGTTCGCC

118987 119007 118896 120149 CDS product envelope glycoprotein D 118987-119007 4:01:01 CCCGCCGGCGTACCCGATGCC

119040 119059 118896 120149 CDS product envelope glycoprotein D 119040-119059 4:01:01 CCGGGCCCATACCGTCGCCC

119299 119316 118896 120149 CDS product envelope glycoprotein D 119299-119316 4:01:01 CCGCTACCGCACACCCCC

119690 119704 118896 120149 CDS product envelope glycoprotein D 119690-119704 5:02:01 CCGCCGCCTGCCGCC

119715 119728 118896 120149 CDS product envelope glycoprotein D 119715-119728 4:01:01 CCCCCGCGCCACCC

119740 119823 118896 120149 CDS product envelope glycoprotein D 119740-119823 13:10:03 GGCCCGCGAGGATGAAGGGGAGACCGAGGACGGGGCAGCCGGGCGGGAGGGCAACGGCGGCCCCCCAGGACCCGAAGGCGACGG

119867 119902 118896 120149 CDS product envelope glycoprotein D 119867-119902 7:04:01 CCGAAACCCGGCCCCAGCCCCGACGCCGACCGCCCC

119935 119979 118896 120149 CDS product envelope glycoprotein D 119935-119979 10:07:02 CCCCCCGCCCGCCCCCGCTACGCCCGCGGCCCCCGACGCCGTGCC

119990 120011 118896 120149 CDS product envelope glycoprotein D 119990-120011 4:01:01 GGGATCGGCATTGCGGCTGCGG
120027 120040 118896 120149 CDS product envelope glycoprotein D 120027-120040 5:02:01 CCGCCGCCGCCGCC
120305 120319 120287 121435 CDS product envelope glycoprotein I 120305-120319 4:01:01 GGATGGTGGTGCTGG
120450 120471 120287 121435 CDS product envelope glycoprotein I 120450-120471 4:01:01 CCTGGAACACCAGCTCCCGGCC
120548 120566 120287 121435 CDS product envelope glycoprotein I 120548-120566 4:01:01 CCGCGTTCGCCTCCTGCCC
120584 120606 120287 121435 CDS product envelope glycoprotein I 120584-120606 4:01:01 CCTTTCGCTCCTGCCTGCACGCC
120671 120689 120287 121435 CDS product envelope glycoprotein I 120671-120689 4:01:01 CCATCGCCCATCCGCGCCC
120721 120743 120287 121435 CDS product envelope glycoprotein I 120721-120743 4:01:01 GGCATCTACGGCGGCACCGCGGG
120802 120840 120287 121435 CDS product envelope glycoprotein I 120802-120840 6:03:01 CCCGGAGACCCAGAGGCCGCGGCCGCGCACCCCGGCGCC
120921 120954 120287 121435 CDS product envelope glycoprotein I 120921-120954 7:04:01 CCCCGCAGGCGCCGCCACCACGCCCGGCCCCACC
121043 121101 120287 121435 CDS product envelope glycoprotein I 121043-121101 14:11:03 CCCTCACCACGCCCCCGGCCGGCCTGACCGCCAGCCCCGCCGCCAGCCCCTCCCGTGCC
121112 121125 120287 121435 CDS product envelope glycoprotein I 121112-121125 4:01:01 CCGCCCCGGCCGCC
121254 121268 120287 121435 CDS product envelope glycoprotein I 121254-121268 4:01:01 CCGCCGCCTGCTGCC
121357 121374 120287 121435 CDS product envelope glycoprotein I 121357-121374 4:01:01 CCGAGCGCCCCAGCCACC
121380 121402 120287 121435 CDS product envelope glycoprotein I 121380-121402 4:01:01 GGCACTGGGCTCCCGGCCAAAGG
121391 121419 120287 121435 CDS product envelope glycoprotein I 121391-121419 6:03:01 CCCGGCCAAAGGCGCCCCCCCTCGCCACC
121719 121734 121714 123441 CDS product envelope glycoprotein E 121719-121734 4:01:01 CCCACCGCGCCGCCCC
121789 121816 121714 123441 CDS product envelope glycoprotein E 121789-121816 5:02:01 CCGAGGCCAAGCCCGCGACCGAAACCCC

121817 121848 121714 123441 CDS product envelope glycoprotein E 121817-121848 5:02:01
GGGCTCGGCTTCGGTCGACACGGTCTTCACGG
121936 121977 121714 123441 CDS product envelope glycoprotein E 121936-121977 7:04:01
CCTGCTCGCCGCCCGTGCCGGAGCCCGTCTGCCTCGACGACC
122011 122045 121714 123441 CDS product envelope glycoprotein E 122011-122045 6:03:01
CCTGCCTGCGAACCGCCCGCGTGGCCCCGCTGGCC
122168 122179 121714 123441 CDS product envelope glycoprotein E 122168-122179 4:01:01 GGAGGACGGCGG
122200 122226 121714 123441 CDS product envelope glycoprotein E 122200-122226 4:01:01
GGCTCATCGGCGACGCCGGCGACGAGG
122252 122275 121714 123441 CDS product envelope glycoprotein E 122252-122275 4:01:01 GGTCGCGACGGCCGGCGCGCAGGG
122301 122337 121714 123441 CDS product envelope glycoprotein E 122301-122337 9:06:02
CCAGCGACCGGGCCCACCCCCGGCCCGCCGCCCCACC
122555 122588 121714 123441 CDS product envelope glycoprotein E 122555-122588 7:04:01
CCACCCCGAGGCACCGGCCTGCCTGCACCCCGCC
122613 122630 121714 123441 CDS product envelope glycoprotein E 122613-122630 4:01:01 CCGTACCGCTCCGAGACC
122657 122672 121714 123441 CDS product envelope glycoprotein E 122657-122672 4:01:01 CCGCCCGGACCCTGCC
122881 122950 121714 123441 CDS product envelope glycoprotein E 122881-122950 12:09:03
CCGACCACACGCGCCCCGAGGCCGCAGCCGCCGACGCTCCCGAGCCAGGCCCACCGCTCACCAGCGAGCC
122961 122978 121714 123441 CDS product envelope glycoprotein E 122961-122978 4:01:01 CCCACCGGGCCCGCGCCC
122980 123025 121714 123441 CDS product envelope glycoprotein E 122980-123025 8:05:02
GGCTTGTGGTGCTGGTGGGCGCGCTTGGACTCGCGGGACTGGTGGG
123089 123136 121714 123441 CDS product envelope glycoprotein E 123089-123136 7:04:01
CCTCAACCCCTTCGGGCCCGTATACACCAGCTTGCCGACCAACGAGCC

123247 123274 121714 123441 CDS product envelope glycoprotein E 123247-123274 5:02:01 CCTACGACCTCGCCGGCGCCCCAGAGCC
123610 123626 123548 123982 CDS product membrane protein US9 123610-123626 5:02:01 CCCCCTTCACCGCCGCC
123635 123655 123548 123982 CDS product membrane protein US9 123635-123655 4:01:01 CCATCGTGTCCATCCCCATCC
123698 123720 123548 123982 CDS product membrane protein US9 123698-123720 4:01:01 CCGGCACCGCGGCAGCCGCACCC
123843 123876 123548 123982 CDS product membrane protein US9 123843-123876 8:05:02 GGGGCGGCAGCAGCGGCGGCGGCATCGGCGGCGG
123910 123944 123548 123982 CDS product membrane protein US9 123910-123944 7:04:01 GGCCTCGGGGCCTGCGCGGCGGCGGCAGCGGCAGG
124558 124579 124548 125450 CDS product regulatory protein ICP22 124558-124579 5:02:01 GGCGGCGCTTGGCCGGCGCGGG
124614 124653 124548 125450 CDS product regulatory protein ICP22 124614-124653 8:05:02 GGCGACGGGTATGGCGAGGAGCCCGGCGAGGAGCCGGAGG
124667 124685 124548 125450 CDS product regulatory protein ICP22 124667-124685 4:01:01 GGGCGCGGGCCGGCGCCGG
124697 124754 124548 125450 CDS product regulatory protein ICP22 124697-124754 10:07:02 GGGGGGGCCGCCGGCGCCGGGCCCGGCGCCGGCCGGGCGCGGGTCCCCGGGCCCGGGG
124704 124730 124548 125450 CDS product regulatory protein ICP22 124704-124730 6:03:01 CCGCCGGCGCCGGGCCCGGCGCCGGCC
124808 124831 124548 125450 CDS product regulatory protein ICP22 124808-124831 6:03:01 GGAGGTGGAAGAGGACGCGGTGGG
124844 124923 124548 125450 CDS product regulatory protein ICP22 124844-124923 17:14:04 GGAGACGGCCGAGGCCGCGGGCGAGGCGGAGGAGAGGACCGCGGCTCGCGGGCGGCACCGGGGGGGGCTTTCGGCGGCGG
124924 124973 124548 125450 CDS product regulatory protein ICP22 124924-124973 13:10:03

CCCCCTCGCCGCCGTTCTCCTCCCCCTCCTCGTCCGCCTCCTCGTCCTCC
125031 125063 124548 125450 CDS product regulatory protein ICP22 125031-125063 6:03:01
GGGCCCGGCAGGCGCGCCGGGGGCGACAGCGGG
125072 125091 124548 125450 CDS product regulatory protein ICP22 125072-125091 6:03:01 GGGCCCGGCGGCGGCGGCGG
125104 125128 124548 125450 CDS product regulatory protein ICP22 125104-125128 4:01:01
GGCGCAGCGGGCCGGCCTGCTCGGG
125321 125345 124548 125450 CDS product regulatory protein ICP22 125321-125345 4:01:01
GGGGCAGAAGGCGCCCGGGCCGCGG
125386 125412 124548 125450 CDS product regulatory protein ICP22 125386-125412 6:03:01
GGTCGGGCGAGCGGCAGAGGCGGCAGG
130575 130612 130514 134545 CDS product transcriptional regulator ICP4 130575-130612 7:04:01
GGACCTCGGCCCGACGGGGGGCTCGGAGGAAGACCCGG
130653 130680 130514 134545 CDS product transcriptional regulator ICP4 130653-130680 5:02:01
CCGGCGGGCCGCCCGCGCCGAGCTGGCC
130688 130714 130514 134545 CDS product transcriptional regulator ICP4 130688-130714 7:04:01
GGCGGATGGTGGGCGGGGAGGACGCGG
130725 130768 130514 134545 CDS product transcriptional regulator ICP4 130725-130768 7:04:01
GGACGACGGCGCCGGAGACGCGGGGGCCACCGAGGGCGCCGAGG
130751 130770 130514 134545 CDS product transcriptional regulator ICP4 130751-130770 4:01:01
CCACCGAGGGCGCCGAGGCC
130779 130840 130514 134545 CDS product transcriptional regulator ICP4 130779-130840 10:07:02
GGAGATCGGCGAGGACGCCGGCGCCAGGGACGCGGGGGGCGCCGAGGACGCGGACGTCGCGG
130851 130882 130514 134545 CDS product transcriptional regulator ICP4 130851-130882 8:05:02

GGCCGAGGGGGCGGAGGGCGCGGAGGACGCGG
130914 130966 130514 134545 CDS product transcriptional regulator ICP4 130914-130966 12:09:03
GGTCGCGGTGGAGGCGGCCGCGGCCGGGGCGGAGGCGGCCGCGGCCGAAGCGG
130952 130992 130514 134545 CDS product transcriptional regulator ICP4 130952-130992 7:04:01
CCGCGGCCGAAGCGGCCGTCATAGCCGCCGCCATCGAAGCC
130995 131005 130514 134545 CDS product transcriptional regulator ICP4 130995-131005 4:01:01 GGCGGGGGCGG
131016 131092 130514 134545 CDS product transcriptional regulator ICP4 131016-131092 13:10:03
GGCCGAAGCGGGGACTGAGGCGGGGGGCGGCGGTGCCCTCGGGGCCAGGACCGAGGCCGGCGACGTTGGGCCCGAGG
131051 131073 130514 134545 CDS product transcriptional regulator ICP4 131051-131073 4:01:01
CCCTCGGGGCCAGGACCGAGGCC
131107 131131 130514 134545 CDS product transcriptional regulator ICP4 131107-131131 4:01:01
GGGTCCGAGGACAGCGGGCCCGAGG
131146 131181 130514 134545 CDS product transcriptional regulator ICP4 131146-131181 7:04:01
GGGCCCGAGGACGATGGGGGCGAGGCCCGGGAGGGG
131202 131238 130514 134545 CDS product transcriptional regulator ICP4 131202-131238 7:04:01
GGCCGAGGCCGAGGCTGAGGCCGGGACTGGGGCCGGG
131234 131258 130514 134545 CDS product transcriptional regulator ICP4 131234-131258 4:01:01
CCGGGACCAGAACCAAAGCGGCCCC
131265 131282 130514 134545 CDS product transcriptional regulator ICP4 131265-131282 4:01:01
GGCGGGCGCCGGGCCCGG
131273 131327 130514 134545 CDS product transcriptional regulator ICP4 131273-131327 10:07:02
CCGGGCCCGGCGCCCCGCGGGGCCGCGCGCCCGGGCCCCCGGCGCCCTCGCCGCC
131338 131356 130514 134545 CDS product transcriptional regulator ICP4 131338-131356 5:02:01

CCCCCCCCGCCGCGGCGCC
131449 131465 130514 134545 CDS product transcriptional regulator ICP4 131449-131465 4:01:01
GGCGAGGCGTGGCCCGG
131473 131488 130514 134545 CDS product transcriptional regulator ICP4 131473-131488 5:02:01 CCGCCGCCGCCCGGCC
131571 131590 130514 134545 CDS product transcriptional regulator ICP4 131571-131590 4:01:01
GGCGGCCGCGGGGCCCGCGG
131604 131622 130514 134545 CDS product transcriptional regulator ICP4 131604-131622 4:01:01
GGAGATGGGGGACGCGGGG
131634 131650 130514 134545 CDS product transcriptional regulator ICP4 131634-131650 4:01:01
GGCGCTGGTGGACCTGG
131858 131878 130514 134545 CDS product transcriptional regulator ICP4 131858-131878 4:01:01
CCTGCGTGGCCATGAGCCGCC
131933 131961 130514 134545 CDS product transcriptional regulator ICP4 131933-131961 6:03:01
CCCCCATGGCCTACCCGGAGGCCGGCGCC
131940 131972 130514 134545 CDS product transcriptional regulator ICP4 131940-131972 7:04:01
GGCCTACCCGGAGGCCGGCGCCGGCGGCAGCGG
132025 132041 130514 134545 CDS product transcriptional regulator ICP4 132025-132041 4:01:01
CCGGCGCCCCTGCCGCC
132042 132061 130514 134545 CDS product transcriptional regulator ICP4 132042-132061 4:01:01
GGCGAGCACGGCGCGGGCGG
132090 132109 130514 134545 CDS product transcriptional regulator ICP4 132090-132109 4:01:01
CCGCTGCGCCGTCGCCTGCC
132114 132127 130514 134545 CDS product transcriptional regulator ICP4 132114-132127 4:01:01 GGCGCTGGAGGCGG

132128 132158 130514 134545 CDS product transcriptional regulator ICP4 132128-132158 7:04:01
CCCGCCGCGCCGCCGGCGCCGCCGGGCTCCC
132142 132199 130514 134545 CDS product transcriptional regulator ICP4 132142-132199 9:06:02
GGCGCCGCCGGGCTCCCGGTGCTCTCGGCCGCGGCGGGCCGCGGCCTGCCGGCCGCGG
132180 132209 130514 134545 CDS product transcriptional regulator ICP4 132180-132209 6:03:01
CCGCGGCCTGCCGGCCGCGGCCTGCGCGCC
132210 132232 130514 134545 CDS product transcriptional regulator ICP4 132210-132232 4:01:01
GGACGCGCTGGCGGCGCACCCGG
132345 132376 130514 134545 CDS product transcriptional regulator ICP4 132345-132376 7:04:01
GGAGGCGGCCGCGCTGGCGGCGCGGACCGTGG
132459 132484 130514 134545 CDS product transcriptional regulator ICP4 132459-132484 7:04:01
GGCGGAGGTGCCGGCGCGGCTGGCGG
132495 132530 130514 134545 CDS product transcriptional regulator ICP4 132495-132530 8:05:02
GGCCCTGGAGGCCGCCGGCGGGGGCGCCGGCGCCGG
132521 132554 130514 134545 CDS product transcriptional regulator ICP4 132521-132554 7:04:01
CCGGCGCCGGCGCCGCCGCGCCGGGCCGGGGGCC
132543 132749 130514 134545 CDS product transcriptional regulator ICP4 132543-132749 44:41:11
GGGCCGGGGGCCGGTGGAAGTGGAGGTGGAGGACATGAGGGCCGGGGCGCCGCGGGCGGACCCGGAGGACGGGTCCGAGGCGGAGGACGGGGAGGACGGGGAGGACGAAGAGGCGGAAGAGGAGGAAG
AGGAGGAAAAGGAGGAAGAGGAGGAAGAGGAGGAAGAGGAGGAAGAGGAGGAAGAGGGCCGCCCCGGCCCGGCGGCGGG
132759 132883 130514 134545 CDS product transcriptional regulator ICP4 132759-132883 26:23:06
GGGTCCGAGGGAAGGAAGAAGGGCCGAGGAGGAGGAGGAGGAGGAGGAGGAAGAAGGGGACGGGGACGGGGACGGCGAGGCGGCGGCCCTGCCGGCCGCCGCGGGGGGCCCCTCGGGGGAGG
132848 132861 130514 134545 CDS product transcriptional regulator ICP4 132848-132861 4:01:01 CCCTGCCGGCCGCC
132914 132938 130514 134545 CDS product transcriptional regulator ICP4 132914-132938 6:03:01

CCGCCGCCGCCGACGCGGCCGGGCC
132930 132949 130514 134545 CDS product transcriptional regulator ICP4 132930-132949 5:02:01
GGCCGGGCCGGCGCGGCGGG
132952 132967 130514 134545 CDS product transcriptional regulator ICP4 132952-132967 4:01:01 CCGCCGCTGCCCGCCC
132979 133005 130514 134545 CDS product transcriptional regulator ICP4 132979-133005 6:03:01
CCCATGCCGCCCGGCGGCCCCGCCGCC
133017 133032 130514 134545 CDS product transcriptional regulator ICP4 133017-133032 4:01:01 CCGCCGCGTGCCGCCC
133041 133062 130514 134545 CDS product transcriptional regulator ICP4 133041-133062 4:01:01
CCACACGCCCGCGCCCAGCGCC
133092 133111 130514 134545 CDS product transcriptional regulator ICP4 133092-133111 4:01:01
GGTCGCGGCGCGGCTCGCGG
133113 133142 130514 134545 CDS product transcriptional regulator ICP4 133113-133142 5:02:01
CCACCCGCTCTTCCCCGAGCCCTGGCGCCC
133184 133203 130514 134545 CDS product transcriptional regulator ICP4 133184-133203 4:01:01
CCCGCCGCCGCGCGGGCCCC
133229 133245 130514 134545 CDS product transcriptional regulator ICP4 133229-133245 4:01:01
GGCGGCGCGTGGCCTGG
133306 133321 130514 134545 CDS product transcriptional regulator ICP4 133306-133321 4:01:01 GGGGAGGCGCTGGCGG
133344 133369 130514 134545 CDS product transcriptional regulator ICP4 133344-133369 5:02:01
CCGGCGGCCCGAGTGGCCGCCGCGCC
133439 133465 130514 134545 CDS product transcriptional regulator ICP4 133439-133465 5:02:01
GGGCCGGGCGCTGGACGGGCGCGCCGG
133553 133575 130514 134545 CDS product transcriptional regulator ICP4 133553-133575 6:03:01

GGCTGGCCGCGGCGCGGCGGCGG
133621 133639 130514 134545 CDS product transcriptional regulator ICP4 133621-133639 4:01:01
GGCCCGGCCGTCGGGCAGG
133847 133886 130514 134545 CDS product transcriptional regulator ICP4 133847-133886 7:04:01
CCACGCGCCTGGGCCCGCGCACCGCGGTGCCCGTGCCGCC
133924 133996 130514 134545 CDS product transcriptional regulator ICP4 133924-133996 12:09:03
GGGCGCAAGGACATGGCCGCGCAGGGCGCGGCGCTGGGGCTCGGGGAGCCGGACTTCGTGGAGGGCGAGGCGG
133997 134017 130514 134545 CDS product transcriptional regulator ICP4 133997-134017 6:03:01
CCAGCCACCGCGCCGCCAACC
134035 134058 130514 134545 CDS product transcriptional regulator ICP4 134035-134058 4:01:01
CCGCTGCGGCCGGTGTACCTGGCC
134042 134067 130514 134545 CDS product transcriptional regulator ICP4 134042-134067 5:02:01
GGCCGGTGTACCTGGCCTGCGGGCGG
134100 134113 130514 134545 CDS product transcriptional regulator ICP4 134100-134113 4:01:01 GGCCGCGGCGGTGG
134126 134159 130514 134545 CDS product transcriptional regulator ICP4 134126-134159 6:03:01
CCGCGCTGCCGGAGCCGCGCGCCGAGGCGCCGCC
134163 134182 130514 134545 CDS product transcriptional regulator ICP4 134163-134182 5:02:01
GGTGCTGGAGGCCGCGGCGG
134185 134205 130514 134545 CDS product transcriptional regulator ICP4 134185-134205 4:01:01
CCGCCCGCGGCCGCGGCGCCC
134193 134239 130514 134545 CDS product transcriptional regulator ICP4 134193-134239 8:05:02
GGCCGCGGCGCCCGGCGTGGACTGGGACGCGGACCAGGGCCCGCGGG
134268 134284 130514 134545 CDS product transcriptional regulator ICP4 134268-134284 5:02:01

GGGCGGCGGCCTGGTGG

134312 134330 130514 134545 CDS product transcriptional regulator ICP4 134312-134330 4:01:01
CCCCGCCGGCCCGGGTGCC

134346 134412 130514 134545 CDS product transcriptional regulator ICP4 134346-134412 14:11:03
GGGCGCGGGCTCGGGCTTCCCGGCGCCGGCGGGCGGCGGCCGGCGGCCGCGGCGGCCCCGGCGCGGG

134438 134472 130514 134545 CDS product transcriptional regulator ICP4 134438-134472 9:06:02
CCTCCTCCTCCTCCTCCTCGTCCTCGTCCTCGTCC

134508 134533 130514 134545 CDS product transcriptional regulator ICP4 134508-134533 6:03:01
GGCCGGCGCCGCCGGCGGCGGGCCGG

gi|126882977|ref|NC_002686.2| Cercopithecine herpesvirus 9, complete genome

960 973 784 1329 CDS product membrane protein UL56A 960-973 4:01:01 CCTCCCACACCCCC

994 1012 784 1329 CDS product membrane protein UL56A 994-1012 5:02:01 CCATTGCCTCCAATCCACC

1701 1728 1571 2452 CDS product multifunctional expression regulator-related protein 1701-1728
5:02:01 CCCCCAACACGCCGGCCTCACATGCCCC

4016 4033 3946 4497 CDS product nuclear protein UL55 4016-4033 4:01:01 CCTCCGCTGCACCTGCCC

7161 7178 6288 7301 CDS product envelope glycoprotein K 7161-7178 4:01:01 CCTCCCTGTACCGGAGCC

8167 8180 7306 10551 CDS product helicase-primase primase subunit 8167-8180 4:01:01 GGAGGCGGTACTGG

10629 10655 10562 11257 CDS product tegument protein UL51 10629-10655 4:01:01 GGTTCGTGGAAAACCGGGTCCATCTGG

11081 11102 10562 11257 CDS product tegument protein UL51 11081-11102 4:01:01 CCATGCTAACCCCTGCGCCTCC

14210 14235 14182 15402 CDS product transactivating tegument protein VP16 14210-14235 4:01:01
GGAACAGTGGCATAATAGGCATTTGG

16140 16168 15721 17649 CDS product tegument protein VP13/14 16140-16168 5:02:01

CCAATGTTACCTTCCTCGCCCAGGTTACC
16563 16602 15721 17649 CDS product tegument protein VP13/14 16563-16602 7:04:01 GGACATGGAAATGGCGTAGGAATAGGAGACGGCTGCTGGG
16707 16733 15721 17649 CDS product tegument protein VP13/14 16707-16733 4:01:01 GGCTTTTTAGGAGCACTGGCTTATTGG
16756 16772 15721 17649 CDS product tegument protein VP13/14 16756-16772 4:01:01 CCGAGCCTGCCACGGCC
18523 18543 17795 19762 CDS product tegument protein VP11/12 18523-18543 4:01:01 GGATATGGATGGATGTTATGG
18767 18790 17795 19762 CDS product tegument protein VP11/12 18767-18790 4:01:01 GGCGAGAAGGGTTAAGTGGAACGG
18884 18896 17795 19762 CDS product tegument protein VP11/12 18884-18896 4:01:01 GGGGGGACGGGGG
22193 22215 20864 22486 CDS product envelope glycoprotein C 22193-22215 5:02:01 GGTTCATGGGCCTGGGGGGTGGG
22991 23027 22639 23904 CDS product envelope protein UL43 22991-23027 6:03:01 CCATTACCAACTCCAATCCCAACATAACCAAAACACC
26914 26936 26827 27762 CDS product ribonucleotide reductase subunit 2 26914-26936 4:01:01 GGAAAATCGGCCTTCGGTGCTGG
31189 31210 30310 31713 CDS product capsid triplex subunit 1 31189-31210 5:02:01 CCACCACCTTCACCAACGGTCC
37097 37119 35204 43165 CDS product large tegument protein 37097-37119 5:02:01 CCAACACCACCCCTTCCCGCCCC
37648 37681 35204 43165 CDS product large tegument protein 37648-37681 7:04:01 GGATTAGGGACATGGCTGGCTTTGGTAGGAGAGG
39877 39887 35204 43165 CDS product large tegument protein 39877-39887 4:01:01 CCCCCACCACC
42193 42212 35204 43165 CDS product large tegument protein 42193-42212 4:01:01 CCTTTAGCCAATACCCCTCC
42505 42753 35204 43165 CDS product large tegument protein 42505-42753 34:31:08 CCCCCCTCTCAGCCCCCCTCTCAGCCCCCCTCTCAGCCCCCCTCTCAGCCCCCCTCTCAGCCCTCTCAGCCCTCTCAGCCCTCTCAGCCCTCTCAGCCCCCCTCTCAGCCCTCTCAGGCCTCTCAGCCCTCTCAGCCCTCTCAGGCCTCTCAGCCCCCCTCTCAGCCCCCCTCTCAGCCCTCTCAGCCCTCTCAGCCCTCTCAGCCCTCTCAGCCCTCTCAGCCCTCTCAGCCCTCTCAGCCCTCTCAGCCCAAAGCC

44672 44685 44597 45241 CDS product nuclear egress membrane protein 44672-44685 4:01:01 GGTGGGGGAAATGG

49045 49065 48395 51913 CDS product DNA polymerase catalytic subunit 49045-49065 4:01:01 GGGAGGAAGCGGTTTTCCTGG

50467 50486 48395 51913 CDS product DNA polymerase catalytic subunit 50467-50486 4:01:01 CCCACCTTTCCCAACATCCC

53280 53299 52099 55683 CDS product single-stranded DNA-binding protein 53280-53299 4:01:01 GGTAAAGATGGGGGTCCGGG

53868 53886 52099 55683 CDS product single-stranded DNA-binding protein 53868-53886 4:01:01 CCGTTTTCCTCCGAGGGCC

54835 54860 52099 55683 CDS product single-stranded DNA-binding protein 54835-54860 4:01:01 CCAAGCGACCACATAATCCATCTTCC

55086 55111 52099 55683 CDS product single-stranded DNA-binding protein 55086-55111 5:02:01 GGAAATTGGAGTGGCCTTAACGGTGG

55569 55585 52099 55683 CDS product single-stranded DNA-binding protein 55569-55585 4:01:01 CCACCTTCATACCAACC

57121 57143 55770 58052 CDS product DNA packaging terminase subunit 2 57121-57143 4:01:01 GGCATCCGAGGTTTATGGATGGG

60136 60169 57911 60661 CDS product envelope glycoprotein B 60136-60169 6:03:01 GGTTTGGGTACTGCGGGACAGGCCGTTGGTCAGG

60241 60265 57911 60661 CDS product envelope glycoprotein B 60241-60265 4:01:01 GGTGCATTGGCTGTTGGACTGTTGG

62385 62410 61291 63057 CDS product capsid maturation protease 62385-62410 5:02:01 CCCCGAAATACCGGCCTCCATTCTCC

62932 62956 61291 63057 CDS product capsid maturation protease 62932-62956 5:02:01

GGTAAGGGGTTTTTAGGCGGTAAGG

63859 63873 63088 64827 CDS product DNA packaging tegument protein UL25 63859-63873 4:01:01 GGAGCCGGAGGTAGG

64984 65003 64891 65637 CDS product nuclear protein UL24 64984-65003 4:01:01 GGATTTGGATTTAAGGTTGG

66480 66505 65663 66676 CDS product thymidine kinase 66480-66505 5:02:01 GGATTTATGGAAATGGGGTATGGGGG

67396 67423 66849 69407 CDS product envelope glycoprotein H 67396-67423 5:02:01 CCTGTTACCAACAAGACCCACCGCACCC

67518 67539 66849 69407 CDS product envelope glycoprotein H 67518-67539 4:01:01 CCACCTTAAAAACCCACGTTCC

67754 67776 66849 69407 CDS product envelope glycoprotein H 67754-67776 5:02:01 CCCCCTGGACCCGACCCGGGACC

69729 69753 69458 71059 CDS product tegument protein UL21 69729-69753 4:01:01 CCGGAACACCCAATAATCCAATTCC

70889 70917 69458 71059 CDS product tegument protein UL21 70889-70917 5:02:01
CCGGGTCCCACGCCACGTACCACATGTCC

73180 73199 72198 76376 CDS product major capsid protein 73180-73199 4:01:01 GGCTTTGGTTATGGGTAAGG

73506 73527 72198 76376 CDS product major capsid protein 73506-73527 4:01:01 CCCCTCCTGATAAACCGGATCC

73907 73926 72198 76376 CDS product major capsid protein 73907-73926 4:01:01 CCAGGTCCCGAAAGACCTCC

81047 81063 80862 81944 CDS product tegument protein UL16 81047-81063 4:01:01 CCATTTATTCCTCCGCC

81406 81422 80862 81944 CDS product tegument protein UL16 81406-81422 4:01:01 GGGAGTATGGTGGTCGG

81645 81661 80862 81944 CDS product tegument protein UL16 81645-81661 4:01:01 GGAGAAAGGCCGGGCGG

83651 83681 83181 83780 CDS product tegument protein UL14 83651-83681 6:03:01
CCCTCCCCGATTCCCCATGCTCCACCAACCC

85964 85987 85120 86649 CDS product deoxyribonuclease 85964-85987 4:01:01 CCGTGCCAAATACCTATTTACCCC

86319 86343 85120 86649 CDS product deoxyribonuclease 86319-86343 5:02:01 CCCGTTTTCGCCAATCCACGCCACC

86669 86688 86649 86897 CDS product myristylated tegument protein 86669-86688 4:01:01 GGCCGAGGAGGAATTTGTGG

87988 88027 86982 88274 CDS product envelope glycoprotein M 87988-88027 7:04:01
CCGGTGCCCCATATCCTTTATTTCCGGCCCACACACCCCC

88432 88454 88310 90760 CDS product DNA replication origin-binding helicase 88432-88454 4:01:01 CCTAATGATCCACAGTTCCGTCC
90585 90607 88310 90760 CDS product DNA replication origin-binding helicase 90585-90607 4:01:01 GGAAGCACGGTTTATGGTTGCGG
91971 91996 90877 93174 CDS product helicase-primase subunit 91971-91996 5:02:01 CCGTGGACCATTCATCCACCCTTACC
92966 92986 90877 93174 CDS product helicase-primase subunit 92966-92986 4:01:01 CCCCAGGGCCATCCCAAAGCC
93453 93478 93243 94157 CDS product tegument protein UL7 93453-93478 5:02:01 GGCGGTGAGGATTTATTGGTGAATGG
94276 94291 94048 96255 CDS product capsid portal protein 94276-94291 4:01:01 CCCCGCCGTTCCAACC
100493 100511 100404 101306 CDS product uracil DNA glycosylase 100493-100511 4:01:01 GGAGAGAGGAGAGGGGTGG
100744 100765 100404 101306 CDS product uracil DNA glycosylase 100744-100765 4:01:01 GGGTCGGTAAAGGCAAGTGGGG
102960 102981 102915 104426 CDS product ubiquitin E3 ligase ICP0 102960-102981 4:01:01 CCCTGTTTCCTACCGCGTCTCC
103727 103747 102915 104426 CDS product ubiquitin E3 ligase ICP0 103727-103747 4:01:01 CCCATATCCATTTGCCTGTCC
103776 103798 102915 104426 CDS product ubiquitin E3 ligase ICP0 103776-103798 4:01:01 GGTGGTTCCGTTGGAACACCAGG
104262 104294 102915 104426 CDS product ubiquitin E3 ligase ICP0 104262-104294 5:02:01 GGACACTGGGCGCTTGTGGAAGTCCAGGTTTGG
105826 105845 105265 109104 CDS product transcriptional regulator ICP4 105826-105845 4:01:01 GGCACGTCGGACGGTCTCGG
105847 105870 105265 109104 CDS product transcriptional regulator ICP4 105847-105870 4:01:01 CCCGCGCTGCCGGGCCTGCGGCCC

105858 105896 105265 109104 CDS product transcriptional regulator ICP4 105858-105896 6:03:01 GGGCCTGCGGCCCTCGGGGAGGTAGACGGGTCGCAGGGG
105956 105984 105265 109104 CDS product transcriptional regulator ICP4 105956-105984 7:04:01 CCGCCGCCCCCACGGCCAATCCCTGCGCC
106211 106230 105265 109104 CDS product transcriptional regulator ICP4 106211-106230 4:01:01 CCGCTCTTGCCAGACCGGCC
106370 106390 105265 109104 CDS product transcriptional regulator ICP4 106370-106390 5:02:01 GGCCGGCGGAGGCGAGCCTGG
106483 106501 105265 109104 CDS product transcriptional regulator ICP4 106483-106501 4:01:01 CCGCTCCAGTTACCGGCCC
106759 106776 105265 109104 CDS product transcriptional regulator ICP4 106759-106776 4:01:01 CCGCCGTGTCCCCTGGCC
106806 106827 105265 109104 CDS product transcriptional regulator ICP4 106806-106827 5:02:01 CCTTAACACCGGCCGCCACGCC
107104 107127 105265 109104 CDS product transcriptional regulator ICP4 107104-107127 5:02:01 CCCGCCAGTCCGCTCCTCTCACCC
107189 107209 105265 109104 CDS product transcriptional regulator ICP4 107189-107209 4:01:01 CCGGGGCCGCTACCGCGGCCC
107245 107260 105265 109104 CDS product transcriptional regulator ICP4 107245-107260 4:01:01 GGGTAGGCCATGGTGG
107331 107353 105265 109104 CDS product transcriptional regulator ICP4 107331-107353 6:03:01 GGCGGCGGCGGCGTGTGGAAGGG
107354 107376 105265 109104 CDS product transcriptional regulator ICP4 107354-107376 4:01:01 CCCACAGAGCCTCTCCACGTGCC

107681 107703 105265 109104 CDS product transcriptional regulator ICP4 107681-107703 5:02:01 CCCCCGACGGTCCAAACCTCACC
107969 108012 105265 109104 CDS product transcriptional regulator ICP4 107969-108012 7:04:01 CCTCTTCCTTGCCCGTACCCGTCGCCTGCTTGTCCCGTGTGTCC
108180 108234 105265 109104 CDS product transcriptional regulator ICP4 108180-108234 9:06:02 CCTGGGTCCGGGTTCGCCCCCGCGTGCGCCACAATCCCCGACCACACCGGTGTCC
108419 108434 105265 109104 CDS product transcriptional regulator ICP4 108419-108434 4:01:01 CCGCCGATGTCCTTCC
108547 108587 105265 109104 CDS product transcriptional regulator ICP4 108547-108587 7:04:01 GGGGACCTTGGTGTGCGTGGATCGGTAGGTTTCTGGGAAGG
108607 108635 105265 109104 CDS product transcriptional regulator ICP4 108607-108635 4:01:01 GGGGACCTCGGGGCCCGTGGGTCGCTTGG
108770 108786 105265 109104 CDS product transcriptional regulator ICP4 108770-108786 4:01:01 CCGGGGCCAAATCCACC
110558 110579 110490 111275 CDS product regulatory protein ICP22 110558-110579 4:01:01 GGAGCCCGGGAGTACGGCTCGG
110703 110726 110490 111275 CDS product regulatory protein ICP22 110703-110726 4:01:01 GGGCATCGGGACGGGTTACAAGGG
111512 111542 111497 112060 CDS product virion protein US10 111512-111542 5:02:01 CCGAACCCGTCAACCCGGGTTCCTGCTACCC
111755 111772 111497 112060 CDS product virion protein US10 111755-111772 4:01:01 CCTTCCCTGCCGTGCGCC
114041 114060 112890 114071 CDS product serine/threonine protein kinase US3 114041-114060 4:01:01 CCGGACCCATATCCCAACCC
116871 116890 115583 117397 CDS product envelope glycoprotein E 116871-116890 4:01:01 GGAACCGGGTTCCGGATTGG

117340  117370  115583  117397  CDS product envelope glycoprotein E  117340-117370  6:03:01 GGCGCGGAGTGCGGCGGGTCTGGATACACGG

117777  117794  117491  118054  CDS product virion protein US10  117777-117794  4:01:01 GGCGCACGGCAGGGAAGG

118007  118037  117491  118054  CDS product virion protein US10  118007-118037  5:02:01 GGGTAGCAGGAACCCGGGTTGACGGGTTCGG

118823  118846  118276  119061  CDS product regulatory protein ICP22  118823-118846  4:01:01 CCCTTGTAACCCGTCCCGATGCCC

118970  118991  118276  119061  CDS product regulatory protein ICP22  118970-118991  4:01:01 CCGAGCCGTACTCCCGGGCTCC

120763  120779  120447  124286  CDS product transcriptional regulator ICP4  120763-120779  4:01:01 GGTGGATTTGGCCCCGG

120914  120942  120447  124286  CDS product transcriptional regulator ICP4  120914-120942  4:01:01 CCAAGCGACCCACGGGCCCCGAGGTCCCC

120962  121002  120447  124286  CDS product transcriptional regulator ICP4  120962-121002  7:04:01 CCTTCCCAGAAACCTACCGATCCACGCACACCAAGGTCCCC

121115  121130  120447  124286  CDS product transcriptional regulator ICP4  121115-121130  4:01:01 GGAAGGACATCGGCGG

121315  121369  120447  124286  CDS product transcriptional regulator ICP4  121315-121369  9:06:02 GGACACCGGTGTGGTCGGGGATTGTGGCGCACGCGGGGGCGAACCCGGACCCAGG

121537  121580  120447  124286  CDS product transcriptional regulator ICP4  121537-121580  7:04:01 GGACACACGGGACAAGCAGGCGACGGGTACGGGCAAGGAAGAGG

121846  121868  120447  124286  CDS product transcriptional regulator ICP4  121846-121868  5:02:01 GGTGAGGTTTGGACCGTCGGGGG

122173  122195  120447  124286  CDS product transcriptional regulator ICP4  122173-122195  4:01:01

GGCACGTGGAGAGGCTCTGTGGG
122196 122218 120447 124286 CDS product transcriptional regulator ICP4 122196-122218 6:03:01 CCCTTCCACACGCCGCCGCCGCC
122289 122304 120447 124286 CDS product transcriptional regulator ICP4 122289-122304 4:01:01 CCACCATGGCCTACCC
122340 122360 120447 124286 CDS product transcriptional regulator ICP4 122340-122360 4:01:01 GGGCCGCGGTAGCGGCCCCGG
122422 122445 120447 124286 CDS product transcriptional regulator ICP4 122422-122445 5:02:01 GGGTGAGAGGAGCGGACTGGCGGG
122722 122743 120447 124286 CDS product transcriptional regulator ICP4 122722-122743 5:02:01 GGCGTGGCGGCCGGTGTTAAGG
122773 122790 120447 124286 CDS product transcriptional regulator ICP4 122773-122790 4:01:01 GGCCAGGGGACACGGCGG
123048 123066 120447 124286 CDS product transcriptional regulator ICP4 123048-123066 4:01:01 GGGCCGGTAACTGGAGCGG
123159 123179 120447 124286 CDS product transcriptional regulator ICP4 123159-123179 5:02:01 CCAGGCTCGCCTCCGCCGGCC
123319 123338 120447 124286 CDS product transcriptional regulator ICP4 123319-123338 4:01:01 GGCCGGTCTGGCAAGAGCGG
123565 123593 120447 124286 CDS product transcriptional regulator ICP4 123565-123593 7:04:01 GGCGCAGGGATTGGCCGTGGGGGCGGCGG
123653 123691 120447 124286 CDS product transcriptional regulator ICP4 123653-123691 6:03:01 CCCCTGCGACCCGTCTACCTCCCCGAGGGCCGCAGGCCC
123679 123702 120447 124286 CDS product transcriptional regulator ICP4 123679-123702 4:01:01

GGGCCGCAGGCCCGGCAGCGCGGG
123704 123723 120447 124286 CDS product transcriptional regulator ICP4 123704-123723 4:01:01
CCGAGACCGTCCGACGTGCC
123799 123818 120447 124286 CDS product transcriptional regulator ICP4 123799-123818 4:01:01
GGTAGCGGGGAGATCGGTGG
123879 123900 120447 124286 CDS product transcriptional regulator ICP4 123879-123900 4:01:01
GGCCACCGGGGTTCGGGAGCGG
123910 123927 120447 124286 CDS product transcriptional regulator ICP4 123910-123927 5:02:01
GGTGGAGATGGGGGATGG
124045 124059 120447 124286 CDS product transcriptional regulator ICP4 124045-124059 4:01:01 GGGCGCGGGTGGCGG
124108 124132 120447 124286 CDS product transcriptional regulator ICP4 124108-124132 4:01:01
CCGAGACCGCGGCGCCCGTCGCTCC
124193 124221 120447 124286 CDS product transcriptional regulator ICP4 124193-124221 5:02:01
GGGGTGTTTAGGACGGGCCCGGCGCACGG

gi|123318702|ref|NC_005261.2| Bovine herpesvirus 5, complete genome
732 778 598 1335 CDS product myristylated tegument protein CIRC 732-778 8:05:02
CCCATGTACCACCACATGCGCCGCGGCCGCGCGGCCCACACCACGCC
794 819 598 1335 CDS product myristylated tegument protein CIRC 794-819 6:03:01 CCGCGCCTACGCCATCCTGCCCTGCC
870 896 598 1335 CDS product myristylated tegument protein CIRC 870-896 6:03:01 CCCGCCACTGGCCTGCACCGCCTCGCC
1000 1014 598 1335 CDS product myristylated tegument protein CIRC 1000-1014 4:01:01 CCACCCCCGAGATCC
1025 1052 598 1335 CDS product myristylated tegument protein CIRC 1025-1052 6:03:01
CCAGGCGGACCAGGCCTTCCCGCCGCCC

1097 1152 598 1335 CDS product myristylated tegument protein CIRC 1097-1152 11:08:02
CCCGCCCACCCGCGCCTTCTGCCCCCACCAGCTGGCCGAGGGCTCCATCCTGGACC

1264 1295 598 1335 CDS product myristylated tegument protein CIRC 1264-1295 7:04:01
CCGACACCACGTCCGCCCCCAGTCCCGGCACC

1959 1977 1702 2913 CDS product multifunctional expression regulator 1959-1977 5:02:01
GGCTGGGCAGGTGGCGAGG

2082 2137 1702 2913 CDS product multifunctional expression regulator 2082-2137 9:06:02
GGCGCCGGCCGTGGCCACGATGGGGTCCTTGGTGCGCAGGGGCAGGTTCTTGGCGG

2216 2235 1702 2913 CDS product multifunctional expression regulator 2216-2235 4:01:01
CCCGCAGCGCCCGCGCCGCC

2267 2286 1702 2913 CDS product multifunctional expression regulator 2267-2286 4:01:01
GGTAGAGGTCGCGGCCGTGG

2304 2326 1702 2913 CDS product multifunctional expression regulator 2304-2326 5:02:01
GGTGACGCGGCGGCCCTCGGGGG

2331 2362 1702 2913 CDS product multifunctional expression regulator 2331-2362 6:03:01
CCGCTCCGGGCCAAAGTCCAGCACGGCCGCCC

2456 2497 1702 2913 CDS product multifunctional expression regulator 2456-2497 7:04:01
CCGTGGCCCTCTGCGCCGCCTCGGCCATGCTCCGCGCGAGCC

2519 2530 1702 2913 CDS product multifunctional expression regulator 2519-2530 4:01:01 GGGCGGAGGCGG

2567 2605 1702 2913 CDS product multifunctional expression regulator 2567-2605 9:06:02
CCGCCTCGCGCTCCGCCGCCGGGCCGCGAGCCCGCCGCC

2641 2656 1702 2913 CDS product multifunctional expression regulator 2641-2656 4:01:01
CCTCGCCGCCGCGGCC

2666 2705 1702 2913 CDS product multifunctional expression regulator 2666-2705 7:04:01
CCCGCCGCGCCAGGGGCGCCCCGGCGGCCCCGTCCTCGCC
2771 2784 1702 2913 CDS product multifunctional expression regulator 2771-2784 4:01:01
CCCGCTCCACCACC
2797 2816 1702 2913 CDS product multifunctional expression regulator 2797-2816 4:01:01
GGGGCCCGCGGGGGCGCGGG
2819 2835 1702 2913 CDS product multifunctional expression regulator 2819-2835 4:01:01
CCTTGTCCCCGTCCTCC
3274 3317 3135 4130 CDS product envelope glycoprotein K 3274-3317 8:05:02
CCTTCCTGATCCCGCTCTCGCCGTCGCCAGCGGCGCCGCCCCCC
3322 3341 3135 4130 CDS product envelope glycoprotein K 3322-3341 5:02:01 GGCGGCGCGGGGGCTTGCGG
3368 3386 3135 4130 CDS product envelope glycoprotein K 3368-3386 4:01:01 GGCGAAGGCGGCGACGTGG
3406 3433 3135 4130 CDS product envelope glycoprotein K 3406-3433 4:01:01 GGTACATCGGGTACGCGGCCGCGCAGGG
3442 3497 3135 4130 CDS product envelope glycoprotein K 3442-3497 11:08:02
CCACGCCGACGCTGCCGGCCGCCGCGACCCGCGCGCCGAGCCGCGCCAGGCCCTCC
3544 3567 3135 4130 CDS product envelope glycoprotein K 3544-3567 6:03:01 GGGGGTCGAGGCGGAAGTCGGCGG
3624 3642 3135 4130 CDS product envelope glycoprotein K 3624-3642 4:01:01 GGGCCGTGGCCGGCCATGG
3627 3644 3135 4130 CDS product envelope glycoprotein K 3627-3644 4:01:01 CCGTGGCCGGCCATGGCC
3683 3699 3135 4130 CDS product envelope glycoprotein K 3683-3699 4:01:01 GGGGCTGAGGAGGTCGG
3901 3918 3135 4130 CDS product envelope glycoprotein K 3901-3918 4:01:01 GGGCGTGGAGGCACGGGG
4108 4120 3135 4130 CDS product envelope glycoprotein K 4108-4120 4:01:01 CCGTCCGCCCCCC
4151 4179 4105 7362 CDS product helicase-primase primase subunit 4151-4179 5:02:01
GGTCCACGGTGAACACGGTCCCGAGGCGG

4380 4410 4105 7362 CDS product helicase-primase primase subunit 4380-4410 6:03:01
GGCGGCCACGAGGCGGGCGCCGGCGCCCTGG
4494 4513 4105 7362 CDS product helicase-primase primase subunit 4494-4513 4:01:01
GGCGCCGCGGTTGTCGGCGG
4562 4585 4105 7362 CDS product helicase-primase primase subunit 4562-4585 5:02:01
GGAAGGCCGCGCGGTTGACGGCGG
4685 4726 4105 7362 CDS product helicase-primase primase subunit 4685-4726 7:04:01
GGTGCGGGTCGCGGTGCGCGGCGACGAAGGCGGCCACGTCGG
4774 4796 4105 7362 CDS product helicase-primase primase subunit 4774-4796 4:01:01
CCGCCCGCGGCGCCCGGCTTGCC
4865 4893 4105 7362 CDS product helicase-primase primase subunit 4865-4893 5:02:01
GGTAGCGGCTCATGGCCTCGGCGAAGGGG
4944 4992 4105 7362 CDS product helicase-primase primase subunit 4944-4992 8:05:02
GGGGCCGCCGGCAATGAGGTAGGGCGCCGGGACGGGCACGGCCACGCGG
5024 5066 4105 7362 CDS product helicase-primase primase subunit 5024-5066 8:05:02
CCGCTAGGGCCGCCGCGCCCGCGTCCTCGGCGCCAGCGCCCCC
5068 5096 4105 7362 CDS product helicase-primase primase subunit 5068-5096 5:02:01
GGCGGCTCGCCGGGCCGGGCGTCCGCGGG
5193 5232 4105 7362 CDS product helicase-primase primase subunit 5193-5232 8:05:02
GGACGGCGAGGCGCTGCCGGCGCGGAGGGCGGCCGAGGGG
5256 5292 4105 7362 CDS product helicase-primase primase subunit 5256-5292 7:04:01
GGGGTGCGCGGGCCAGGCGTCGGCGGCGGCCTCGGGG
5351 5379 4105 7362 CDS product helicase-primase primase subunit 5351-5379 5:02:01

GGTCCCCGCGGGGGATGGGGCGCCTAAGG
5406 5422 4105 7362 CDS product helicase-primase primase subunit 5406-5422 4:01:01
GGTCACGGCGAGGCGGG
5464 5480 4105 7362 CDS product helicase-primase primase subunit 5464-5480 4:01:01
GGCGCGGGCGGGCCCGG
5497 5513 4105 7362 CDS product helicase-primase primase subunit 5497-5513 4:01:01
CCCCCGCCCGCGCTGCC
5534 5545 4105 7362 CDS product helicase-primase primase subunit 5534-5545 4:01:01 GGAGGAGGGCGG
5592 5608 4105 7362 CDS product helicase-primase primase subunit 5592-5608 4:01:01
GGCAGCGGCGACGGCGG
5609 5655 4105 7362 CDS product helicase-primase primase subunit 5609-5655 9:06:02
CCGCGACCTCGGCCGCCACCTCCCCGGGGTCCAGGGCGGCCACGTCC
5694 5711 4105 7362 CDS product helicase-primase primase subunit 5694-5711 4:01:01
GGCCTGGTGGCCGCGCGG
5723 5746 4105 7362 CDS product helicase-primase primase subunit 5723-5746 4:01:01
GGTAGACGGGCGCGGGCACCGCGG
5741 5772 4105 7362 CDS product helicase-primase primase subunit 5741-5772 5:02:01
CCGCGGTCCCGCGCGCGCCAAAAAGCCCGTCC
5875 5906 4105 7362 CDS product helicase-primase primase subunit 5875-5906 6:03:01
GGCGCGGCGGGCGCGGCGTCGGGGCCCAGCGG
5911 5937 4105 7362 CDS product helicase-primase primase subunit 5911-5937 6:03:01
CCCGCCGCGCCGGCCGGCGTGCCGTCC
6021 6063 4105 7362 CDS product helicase-primase primase subunit 6021-6063 7:04:01

GGTGCGCCCGGGGGGCCCGTCGGTCCTCGCGGCGGGCGCCAGG
6090 6124 4105 7362 CDS product helicase-primase primase subunit 6090-6124 6:03:01
GGCCTCGGCCGTCGCGGGGGGCAGCCGGGCGACGG
6209 6220 4105 7362 CDS product helicase-primase primase subunit 6209-6220 4:01:01 GGAGGCGGGAGG
6230 6253 4105 7362 CDS product helicase-primase primase subunit 6230-6253 5:02:01
GGCGCGGCGCAAGGGGGGCGCCGG
6264 6310 4105 7362 CDS product helicase-primase primase subunit 6264-6310 10:07:02
GGCAGCGGCGGGGGGCTCGGTGGCGGTGACGGCACGCAGGTGCTCGG
6359 6381 4105 7362 CDS product helicase-primase primase subunit 6359-6381 4:01:01
CCACGAACTCCGGGTCGGCCACC
6386 6402 4105 7362 CDS product helicase-primase primase subunit 6386-6402 4:01:01
GGCTGCGGCGGTCGTGG
6419 6438 4105 7362 CDS product helicase-primase primase subunit 6419-6438 4:01:01
GGCAGCGGACCTCGGCGAGG
6567 6603 4105 7362 CDS product helicase-primase primase subunit 6567-6603 7:04:01
GGGGTTGGGCGGCGCGGCGACGGCGTACGTGGCCAGG
6615 6660 4105 7362 CDS product helicase-primase primase subunit 6615-6660 8:05:02
GGCCTGCAGGTCGTAGGTGGCGGCGTCGCCGGCGCGCTCGGCCTGG
6729 6748 4105 7362 CDS product helicase-primase primase subunit 6729-6748 4:01:01
GGGGGTCGCGCCGGAGTTGG
6804 6828 4105 7362 CDS product helicase-primase primase subunit 6804-6828 5:02:01
GGCCAGGCCGCGGCGCCCGGCCGGG
6806 6825 4105 7362 CDS product helicase-primase primase subunit 6806-6825 4:01:01

CCAGGCCGCGGCGCCCGGCC

7065 7105 4105 7362 CDS product helicase-primase primase subunit 7065-7105 7:04:01

GGCGCAGGCAAAGACGGGCCAGGCGCGGGGCCGCGCGGCGG

7111 7128 4105 7362 CDS product helicase-primase primase subunit 7111-7128 4:01:01

CCGCCCGGCGGGCCGCCC

7249 7271 4105 7362 CDS product helicase-primase primase subunit 7249-7271 5:02:01

CCGCGGCCGGGCTCCTCCTGGCC

7373 7414 7361 8149 CDS product tegument protein UL51 7373-7414 9:06:02

GGCTGGTTGGGTGGCTGTGCGGGCGTGGCGGGGGCGCGCGGG

7476 7489 7361 8149 CDS product tegument protein UL51 7476-7489 5:02:01 GGAGGCGGTGGTGG

7490 7514 7361 8149 CDS product tegument protein UL51 7490-7514 4:01:01 CCGTCAGCTCCCTGCTGCCGGCGCC

7521 7560 7361 8149 CDS product tegument protein UL51 7521-7560 9:06:02

GGTGGAGGACGTGGTGCGCTCGGCGGACGGCGCGCGGCGG

7743 7786 7361 8149 CDS product tegument protein UL51 7743-7786 8:05:02

GGTGGGCGCCGTGGAGGGCACGACGGACAGCATGGTGGACCAGG

7851 7874 7361 8149 CDS product tegument protein UL51 7851-7874 4:01:01 GGGCATCCGGGCGCAGGGCGCGGG

7884 7905 7361 8149 CDS product tegument protein UL51 7884-7905 5:02:01 GGAGGCGAAGGCGAAGGCCGGG

7914 7939 7361 8149 CDS product tegument protein UL51 7914-7939 5:02:01 GGCGGTCGCCGGTGCTGAAGGCCGGG

7935 7996 7361 8149 CDS product tegument protein UL51 7935-7996 12:09:03

CCGGGCCCCGCCGCCCCTGCCGCTGCAGCCGGCGGGCCTGCCTCCGGCCCCGGCCCCGGCCC

8008 8051 7361 8149 CDS product tegument protein UL51 8008-8051 12:09:03

CCCCCGGTCCCGCCCCTGCCGCCGCCGCCGCCACCGTTGCCGCC

8105 8127 7361 8149 CDS product tegument protein UL51 8105-8127 6:03:01 CCGCCTCCTCCTCTACGCCCGCC

8292 8318 8287 9252 CDS product deoxyuridine triphosphatase 8292-8318 4:01:01
CCCGGTGGACCCGAAGCCGCGCGACCC
8343 8361 8287 9252 CDS product deoxyuridine triphosphatase 8343-8361 4:01:01 GGCGAGGTCGGCGACGCGG
8372 8448 8287 9252 CDS product deoxyuridine triphosphatase 8372-8448 15:12:03
GGCGCGGGGCGCCGGGGGCGGGCGGGGCGGCGCGGGGGCTTGTCGGGAAGGGCTCGCGGTCGTTGAGGCTGGGCGGG
8546 8580 8287 9252 CDS product deoxyuridine triphosphatase 8546-8580 7:04:01
GGCAGGGCTCCCCAGGGGGCCAGGCTGTCGGCAGG
8748 8771 8287 9252 CDS product deoxyuridine triphosphatase 8748-8771 4:01:01 GGGGGCGAACGTGGCAAAGAAGGG
8782 8805 8287 9252 CDS product deoxyuridine triphosphatase 8782-8805 7:04:01 CCCGCCTCCCACCCGGCCCCCGCC
8825 8842 8287 9252 CDS product deoxyuridine triphosphatase 8825-8842 4:01:01 GGCGGGGCACCGTGGCGG
8874 8896 8287 9252 CDS product deoxyuridine triphosphatase 8874-8896 4:01:01 CCCCGCCGCGGGCCCGGGCGCCC
8952 8972 8287 9252 CDS product deoxyuridine triphosphatase 8952-8972 5:02:01 GGCGGGCGGGCGGGGCCCGGG
9066 9086 8287 9252 CDS product deoxyuridine triphosphatase 9066-9086 4:01:01 CCGCCCCGGCGGTGCCTCTCC
9098 9126 8287 9252 CDS product deoxyuridine triphosphatase 9098-9126 5:02:01
CCGCCAGCGCAACCGGGCCCCAGAGCGCC
9170 9180 8287 9252 CDS product deoxyuridine triphosphatase 9170-9180 4:01:01 CCTCCGCCGCC
9287 9321 9203 9490 CDS product envelope glycoprotein N 9287-9321 5:02:01
GGCGCGAGGAAGCGATGGACTTCTGGAGCGCGGGG
9471 9485 9203 9490 CDS product envelope glycoprotein N 9471-9485 4:01:01 GGTGCGGGGGCGGGG
9625 9656 9624 10427 CDS product tegument protein VP22 9625-9656 5:02:01
GGCCCGGTTCCACAGGTCCTCGGACGACGAGG
9764 9790 9624 10427 CDS product tegument protein VP22 9764-9790 5:02:01 CCCGAGCCCAAGCCCAGGCCCGGGCCC
9780 9802 9624 10427 CDS product tegument protein VP22 9780-9802 4:01:01 GGCCCGGGCCCGACGGGCGCCGG

9810 9844 9624 10427 CDS product tegument protein VP22 9810-9844 6:03:01
CCCGCTCGTGCCCTGGCGCCGCGACCGCCCCGTCC
9872 9896 9624 10427 CDS product tegument protein VP22 9872-9896 6:03:01 CCCGCCGCCGCGGCGCCGGCCCGCC
9922 9970 9624 10427 CDS product tegument protein VP22 9922-9970 9:06:02
CCCGCCGCGGCCCGCTCCCGCCGCCAACGCTCCCGCTACCAGGCAGGCC
9981 9994 9624 10427 CDS product tegument protein VP22 9981-9994 4:01:01 CCTCCGGCGCCGCC
10005 10068 9624 10427 CDS product tegument protein VP22 10005-10068 14:11:03
CCGCCGCCGTCCCGCCCCGAACCCGCGCGCCCCCCGGCGCCGCCGCCGTCGCGTCCGGCCGGCC
10290 10309 9624 10427 CDS product tegument protein VP22 10290-10309 5:02:01 CCGGGCTCCTCGCCGCCGCC
10318 10357 9624 10427 CDS product tegument protein VP22 10318-10357 8:05:02
CCTAGCCACGCGGGCCCAGCGCCCCGCCGCTCCGGCCCCC
10351 10370 9624 10427 CDS product tegument protein VP22 10351-10370 4:01:01 GGCCCCCGCGGGCGGCAGGG
10371 10384 9624 10427 CDS product tegument protein VP22 10371-10384 4:01:01 CCCCACCGCCAGCC
10406 10420 9624 10427 CDS product tegument protein VP22 10406-10420 4:01:01 GGCGGGCGGGCCCGG
10689 10708 10620 12068 CDS product transactivating tegument protein VP16 10689-10708 4:01:01
CCCCTGACGGTCCGTCCCCC
10724 10750 10620 12068 CDS product transactivating tegument protein VP16 10724-10750 6:03:01
CCGCCCCCGCGCCCGGCGCCCCTGGCC
10809 10832 10620 12068 CDS product transactivating tegument protein VP16 10809-10832 5:02:01
GGGCCATGGAGCGGTGGAACGAGG
10927 10955 10620 12068 CDS product transactivating tegument protein VP16 10927-10955 5:02:01
GGCGGTGCCTGGGGACGCGGAGCGCTTGG
10975 10999 10620 12068 CDS product transactivating tegument protein VP16 10975-10999 6:03:01

CCAGCCGCTTCCCGCACCGCCGGCC
10996 11039 10620 12068 CDS product transactivating tegument protein VP16 10996-11039 8:05:02
GGCCTCGGAGGAGGGCCTCCCGGAGTATGTGGCCGGCGTACAGG
11134 11174 10620 12068 CDS product transactivating tegument protein VP16 11134-11174 8:05:02
GGCGGCGCGTGGCCGAGGCGCGGCGGGCGCGGGCGCCCAGG
11187 11206 10620 12068 CDS product transactivating tegument protein VP16 11187-11206 5:02:01
GGCAGCTGGTGGCGGCGCGG
11364 11380 10620 12068 CDS product transactivating tegument protein VP16 11364-11380 4:01:01
CCTGCCTGTTCCACCCC
11396 11446 10620 12068 CDS product transactivating tegument protein VP16 11396-11446 10:07:02
GGCGTCGTGGCGCTGGAGGACGGCTTCTTGGACGCGGCGGAGCTGCGGCGG
11482 11528 10620 12068 CDS product transactivating tegument protein VP16 11482-11528 8:05:02
GGTCCGCGCGGGGCTGGTCGAGGTTGAAGTGGGGCCTCTGGTGGAGG
11611 11639 10620 12068 CDS product transactivating tegument protein VP16 11611-11639 7:04:01
GGCCGGCGGGCGGCTGGCGCCGGAGCGGG
11795 11833 10620 12068 CDS product transactivating tegument protein VP16 11795-11833 7:04:01
CCTTCAGCCAAGGGCGCCGCCCCGGCCGAGTTTGCCGCC
11904 11946 10620 12068 CDS product transactivating tegument protein VP16 11904-11946 7:04:01
CCTTGGCCTTAGCCGAGCCCGCGGCAGCCCTGGCCCCGGCCCC
11958 12006 10620 12068 CDS product transactivating tegument protein VP16 11958-12006 8:05:02
CCCCAGCCGAGCCCGCGGCGGCCGTCGCCGGGCCAAGCCCGGCAAACCC
12262 12285 12239 14464 CDS product tegument protein VP13/14 12262-12285 5:02:01 CCTGAGCGCCGCCCGCGCCGCTCC
12294 12334 12239 14464 CDS product tegument protein VP13/14 12294-12334 6:03:01

CCGCACGCACCCGTTCCAGCGCCCCTCTGCCCGGCGGAGCC
12378 12419 12239 14464 CDS product tegument protein VP13/14 12378-12419 7:04:01
CCCGCGGGTCCGGCGCCCGCGGCCTGACTTCCAGCGGCCCCC
12614 12642 12239 14464 CDS product tegument protein VP13/14 12614-12642 5:02:01
CCCACCTGCGCGCCATAGAGGCCCTGCCC
12781 12807 12239 14464 CDS product tegument protein VP13/14 12781-12807 7:04:01
GGCGGCGGGGGCGGCCAGGAACCCGGG
12907 12922 12239 14464 CDS product tegument protein VP13/14 12907-12922 4:01:01 CCCCCGCTCCTCAGCC
12924 12967 12239 14464 CDS product tegument protein VP13/14 12924-12967 10:07:02
GGAGGCGGCCGCTGCGGCTGCGGGGGAGAGGAGCGTGGTGGAGG
13019 13041 12239 14464 CDS product tegument protein VP13/14 13019-13041 5:02:01 GGATTTTGGAGGGCCGGGTGCGG
13121 13142 12239 14464 CDS product tegument protein VP13/14 13121-13142 4:01:01 GGCGCGCCTGGGAGATGGCCGG
13172 13189 12239 14464 CDS product tegument protein VP13/14 13172-13189 4:01:01 GGAGCACGGTGGACCCGG
13227 13254 12239 14464 CDS product tegument protein VP13/14 13227-13254 6:03:01
GGGCACCCGGCCCCGGCTGGTGTGGCGG
13289 13304 12239 14464 CDS product tegument protein VP13/14 13289-13304 5:02:01 GGCTGGGGGTGGGGGG
13409 13427 12239 14464 CDS product tegument protein VP13/14 13409-13427 4:01:01 GGCCGGGGCGCGCGGGCGG
13449 13468 12239 14464 CDS product tegument protein VP13/14 13449-13468 5:02:01 GGCGGCGGTTGGGCTGCTGG
13869 13885 12239 14464 CDS product tegument protein VP13/14 13869-13885 4:01:01 GGAGACGGTGGACTCGG
13886 13903 12239 14464 CDS product tegument protein VP13/14 13886-13903 4:01:01 CCACCTTCCAGGAGGCCC
13933 13956 12239 14464 CDS product tegument protein VP13/14 13933-13956 5:02:01 GGGGAGGCCTACGTGGCGGTGCGG
14101 14124 12239 14464 CDS product tegument protein VP13/14 14101-14124 5:02:01 GGCGCAGCGGTGTACGGGGGCCGG
14190 14224 12239 14464 CDS product tegument protein VP13/14 14190-14224 6:03:01

GGTGCGCCCGGTGTCCCTGGTGGAGTTCTGGGAGG

14280 14302 12239 14464 CDS product tegument protein VP13/14 14280-14302 5:02:01 GGCCGGCAAGCGGCGGGTCATGG

14356 14376 12239 14464 CDS product tegument protein VP13/14 14356-14376 4:01:01 GGTTCGCGGCCGGTGCTTGGG

14385 14407 12239 14464 CDS product tegument protein VP13/14 14385-14407 5:02:01 GGACATCGCGGAGGCGCTGGCGG

14670 14707 14597 16801 CDS product tegument protein VP11/12 14670-14707 6:03:01
GGACGCCACGGCCGCGGCCGTAGTGGCCCTGCAGGAGG

14675 14698 14597 16801 CDS product tegument protein VP11/12 14675-14698 4:01:01 CCACGGCCGCGGCCGTAGTGGCCC

14790 14815 14597 16801 CDS product tegument protein VP11/12 14790-14815 5:02:01
GGAGGCGCTGGTTCTGTCGGACTCGG

14867 14886 14597 16801 CDS product tegument protein VP11/12 14867-14886 4:01:01 GGGTGCTGGCGGGCGCGCGG

14974 15004 14597 16801 CDS product tegument protein VP11/12 14974-15004 7:04:01
GGCGCGGCCGGCGGCGCGGCGATGGAGCAGG

15090 15112 14597 16801 CDS product tegument protein VP11/12 15090-15112 4:01:01 GGCGTACAAGGCCGCTGCGGCGG

15322 15341 14597 16801 CDS product tegument protein VP11/12 15322-15341 4:01:01 CCGCGCGAGCCGCGGCCGCC

15360 15377 14597 16801 CDS product tegument protein VP11/12 15360-15377 5:02:01 CCACCTGACCGCCGGCCC

15417 15439 14597 16801 CDS product tegument protein VP11/12 15417-15439 5:02:01 GGCCCTGACGGCGGCGGCGCTGG

15519 15541 14597 16801 CDS product tegument protein VP11/12 15519-15541 4:01:01 GGCCGCGGTCGAGCTGGCGTCGG

15542 15565 14597 16801 CDS product tegument protein VP11/12 15542-15565 5:02:01 CCGTGCACCACCACCTGCAGTACC

15676 15702 14597 16801 CDS product tegument protein VP11/12 15676-15702 6:03:01
CCGCTGATGCCAACCATGACCTCCGCC

15707 15738 14597 16801 CDS product tegument protein VP11/12 15707-15738 6:03:01
GGGCGGCGCTGGAGCGCGGCACGGCGAGCTGG

15818 15850 14597 16801 CDS product tegument protein VP11/12 15818-15850 6:03:01

GGCTCGGCGGGAGCCGCGGGCTGCTGGCGCCGG
15847 15923 14597 16801 CDS product tegument protein VP11/12 15847-15923 15:12:03
CCGGTGCGCCGCCGCGGCGGCCCGCAGAGCCCAGCCACGCGCCCCCTGCCCAGCCCGCCGGCCGCGGCCACCCCACC
16067 16081 14597 16801 CDS product tegument protein VP11/12 16067-16081 5:02:01 GGCGGGAGGCGGCGG
16115 16134 14597 16801 CDS product tegument protein VP11/12 16115-16134 4:01:01 CCTCGTCCCCGCCCTTCGCC
16179 16201 14597 16801 CDS product tegument protein VP11/12 16179-16201 5:02:01 CCCCATGCCCGCCCCGCCGCCCC
16242 16264 14597 16801 CDS product tegument protein VP11/12 16242-16264 5:02:01 CCCCATGCCCGCCCCGCCGCCCC
16375 16396 14597 16801 CDS product tegument protein VP11/12 16375-16396 4:01:01 GGAGCCGGAGCCGGGGACGGGG
16479 16545 14597 16801 CDS product tegument protein VP11/12 16479-16545 11:08:02
CCGGCCCGCGGCCCCGGGCCCGGCCCCGGCCCCGTGCACCGAGCGCCCGGGCAACCCTACCCGGTCC
16481 16507 14597 16801 CDS product tegument protein VP11/12 16481-16507 5:02:01
GGCCCGCGGCCCCGGGCCCGGCCCCGG
16560 16594 14597 16801 CDS product tegument protein VP11/12 16560-16594 6:03:01
CCCGCGCCTCCGCGCCCTAGAGGACCCGCACGGCC
16591 16616 14597 16801 CDS product tegument protein VP11/12 16591-16616 6:03:01
GGCCTGGAGGCGCTGGCGGCCGCCGG
16610 16642 14597 16801 CDS product tegument protein VP11/12 16610-16642 6:03:01
CCGCCGGCGCCGCGCACACCGCGCGCCACAACC
16664 16683 14597 16801 CDS product tegument protein VP11/12 16664-16683 4:01:01 CCCGCGCCAGCGGGTCCTCC
16741 16759 14597 16801 CDS product tegument protein VP11/12 16741-16759 5:02:01 CCTCCACCTTCCCGCGCCC
17081 17103 17022 18482 CDS product envelope glycoprotein C 17081-17103 4:01:01 GGCGCCGAGGCCGCAGGCGACGG
17159 17211 17022 18482 CDS product envelope glycoprotein C 17159-17211 9:06:02
GGCGGTCGCGGAGAACTCGGGCAGCGGCGCCGGGTAGCCGGTGGCAGTGCAGG

17414 17456 17022 18482 CDS product envelope glycoprotein C 17414-17456 8:05:02
GGGCGGGCGGTAGACGGCCGGCGTGGCAGCCGCGGCGAAGCGG
17500 17521 17022 18482 CDS product envelope glycoprotein C 17500-17521 6:03:01 GGTTGGGCGGGTGGGCGGTTGG
17638 17657 17022 18482 CDS product envelope glycoprotein C 17638-17657 4:01:01 GGCGCGTGGAGCGCGGCGGG
17666 17682 17022 18482 CDS product envelope glycoprotein C 17666-17682 4:01:01 GGCGGCGCGGCACACGG
17683 17702 17022 18482 CDS product envelope glycoprotein C 17683-17702 4:01:01 CCGCGTAGCCGGCGCCCTCC
17709 17741 17022 18482 CDS product envelope glycoprotein C 17709-17741 6:03:01
GGCCGGGGCTCGACGGAGACGGCGGGTGCGCGG
17813 17873 17022 18482 CDS product envelope glycoprotein C 17813-17873 11:08:02
GGCTGCGGCGGTCAGGGAGCGCACGGTCAGGGGCAGGCTGCCGGGGTCGGCGGGCGAGGGG
17945 17971 17022 18482 CDS product envelope glycoprotein C 17945-17971 5:02:01 GGGCGGGGCGCGCGGGAAGGGGTTGGG
18084 18108 17022 18482 CDS product envelope glycoprotein C 18084-18108 5:02:01 GGCTCGGCATACGGGGCGGCCACGG
18188 18205 17022 18482 CDS product envelope glycoprotein C 18188-18205 5:02:01 GGCGGGCGGCGGCGCCGG
18243 18277 17022 18482 CDS product envelope glycoprotein C 18243-18277 7:04:01
GGCGTGGCCGGGGGGCTGTGGGAAGGAGCTGTGGG
18317 18349 17022 18482 CDS product envelope glycoprotein C 18317-18349 7:04:01
GGCCGGGGTTGGGGAGGGCGGGCTTGGGGAGGG
18360 18384 17022 18482 CDS product envelope glycoprotein C 18360-18384 6:03:01 GGCGGGGGCAAGGGCGAGGGCGAGG
18385 18401 17022 18482 CDS product envelope glycoprotein C 18385-18401 4:01:01 CCTCGCCCTCCGCTTCC
18729 18755 18671 19813 CDS product envelope protein UL43 18729-18755 5:02:01 CCTTGGCCACGTGCGCCGCGGCCAGCC
18764 18785 18671 19813 CDS product envelope protein UL43 18764-18785 4:01:01 CCGCGCCCGCGGCGCCGGACCC
18795 18829 18671 19813 CDS product envelope protein UL43 18795-18829 6:03:01
CCACCAGCGCGCCCGCGGTGGCCGAGGCCAGGGCC

18811 18834 18671 19813 CDS product envelope protein UL43 18811-18834 5:02:01 GGTGGCCGAGGCCAGGGCCGCGGG
18839 18852 18671 19813 CDS product envelope protein UL43 18839-18852 4:01:01 CCGCCCTCCGCGCC
19009 19032 18671 19813 CDS product envelope protein UL43 19009-19032 5:02:01 CCAGCGCCCGCCGCCCTCAGCCCC
19043 19072 18671 19813 CDS product envelope protein UL43 19043-19072 5:02:01 CCCGGGGCCCAGAGCGCCGCCGCGGCGGCC
19066 19082 18671 19813 CDS product envelope protein UL43 19066-19082 4:01:01 GGCGGCCAGGGCAACGG
19156 19173 18671 19813 CDS product envelope protein UL43 19156-19173 4:01:01 GGGGTCGGCGGGGCAGGG
19191 19214 18671 19813 CDS product envelope protein UL43 19191-19214 4:01:01 CCAGGGTCCGCAGCGCCGCATACC
19252 19268 18671 19813 CDS product envelope protein UL43 19252-19268 4:01:01 GGCGGCGTCCGCGGCGG
19292 19344 18671 19813 CDS product envelope protein UL43 19292-19344 9:06:02 CCCGCGGCCGCCAGCGCCTCGGGCCGCGCGCCCCAGCGCCCGGCCGAGAGGCC
19450 19469 18671 19813 CDS product envelope protein UL43 19450-19469 4:01:01 GGCGTAGGCCGCGGCCGCGG
19458 19482 18671 19813 CDS product envelope protein UL43 19458-19482 4:01:01 CCGCGGCCGCGGCCGTCAGCGCCCC
19492 19520 18671 19813 CDS product envelope protein UL43 19492-19520 5:02:01 GGCAGGCGCGGGGCCCGCGGCCCAGACGG
19626 19642 18671 19813 CDS product envelope protein UL43 19626-19642 4:01:01 CCGCTGCCGCGGCCGCC
19686 19707 18671 19813 CDS product envelope protein UL43 19686-19707 5:02:01 CCAGCGCCGCCGCCGTCAGGCC
19760 19796 18671 19813 CDS product envelope protein UL43 19760-19796 6:03:01 CCGCAGCCGGCCTTCGTTCCCGCGCAGCCGCTCACCC
21602 21626 21358 22722 CDS product tegument host shutoff protein 21602-21626 4:01:01 GGACCGCGGCATCCACGGGGACCGG
21605 21632 21358 22722 CDS product tegument host shutoff protein 21605-21632 4:01:01 CCGCGGCATCCACGGGGACCGGCGCGCC

21678 21701 21358 22722 CDS product tegument host shutoff protein 21678-21701 5:02:01 GGCGGCTCAGGCCGCCTCGGGCGG
21725 21742 21358 22722 CDS product tegument host shutoff protein 21725-21742 4:01:01 GGAGGACGAGGTGCTGGG
21770 21784 21358 22722 CDS product tegument host shutoff protein 21770-21784 4:01:01 GGACGCGGCGGCCGG
21795 21816 21358 22722 CDS product tegument host shutoff protein 21795-21816 6:03:01 GGCGGGGAGGAGGCGGCGAGGG
21836 21852 21358 22722 CDS product tegument host shutoff protein 21836-21852 4:01:01 GGCGGGGGCCGCGCGGG
22136 22159 21358 22722 CDS product tegument host shutoff protein 22136-22159 5:02:01 CCACACGGACCTGCACCAGCCGCC
22172 22199 21358 22722 CDS product tegument host shutoff protein 22172-22199 6:03:01 GGTGCAGCAGGTGGTGCGGGGGCTGCGG
22204 22232 21358 22722 CDS product tegument host shutoff protein 22204-22232 6:03:01 CCGCCGCGGCCGAGCCCGCGACCGACACC
22235 22249 21358 22722 CDS product tegument host shutoff protein 22235-22249 4:01:01 GGCGGAGGAGTCCGG
22269 22289 21358 22722 CDS product tegument host shutoff protein 22269-22289 4:01:01 CCGCGGCCCCGGCTGCCGCCC
22631 22652 21358 22722 CDS product tegument host shutoff protein 22631-22652 5:02:01 CCCCACCCCGCCCCCCTTCGCC
22672 22710 21358 22722 CDS product tegument host shutoff protein 22672-22710 7:04:01 GGGACGAGGCCTCCGCGGGGCCGGGGCCGCGGCGGCCGG
22681 22716 21358 22722 CDS product tegument host shutoff protein 22681-22716 7:04:01 CCTCCGCGGGGCCGGGGCCGCGGCGGCCGGCCCGCC
22926 22958 22847 23794 CDS product ribonucleotide reductase subunit 2 22926-22958 6:03:01

GGGCCAGAGGAAAATCGGTCCCAGGAGGCGGGG

22970 22981 22847 23794 CDS product ribonucleotide reductase subunit 2 22970-22981 4:01:01 GGAGGCAGGTGG

23202 23224 22847 23794 CDS product ribonucleotide reductase subunit 2 23202-23224 4:01:01 GGCTGATGAGGTCGTTGGTTTGG

23241 23261 22847 23794 CDS product ribonucleotide reductase subunit 2 23241-23261 4:01:01 GGTTGTGGGTGCGCAGGTAGG

23328 23386 22847 23794 CDS product ribonucleotide reductase subunit 2 23328-23386 10:07:02 CCACCGACTCGGCCGCGGCCACGCGCCGCTCGAGCCAGTCCACCTTGCGCCGGACTGCC

23403 23428 22847 23794 CDS product ribonucleotide reductase subunit 2 23403-23428 5:02:01 CCTCCACGTAGCCCGCGCGCGCCACC

23571 23590 22847 23794 CDS product ribonucleotide reductase subunit 2 23571-23590 4:01:01 GGTCATCGGCGGCCGAGAGG

23627 23647 22847 23794 CDS product ribonucleotide reductase subunit 2 23627-23647 4:01:01 CCGCTGAGCCGCGCCACGTCC

24018 24056 23814 26216 CDS product ribonucleotide reductase subunit 1 24018-24056 6:03:01 GGGGCGCGGTCGGCGCAGAGGTCGATCAGGCGCTCCTGG

24083 24107 23814 26216 CDS product ribonucleotide reductase subunit 1 24083-24107 4:01:01 GGCCAGCGGGTGCCCGGGCGCGAGG

24285 24305 23814 26216 CDS product ribonucleotide reductase subunit 1 24285-24305 4:01:01 CCCTCGCTGCCCTCCGTCACC

24314 24333 23814 26216 CDS product ribonucleotide reductase subunit 1 24314-24333 4:01:01 GGACACGGTCGGCATGAGGG

24506 24543 23814 26216 CDS product ribonucleotide reductase subunit 1 24506-24543 7:04:01

GGCGCTGGTGGCCATGACGGCCAGGAGCAGGCGCTCGG
24716 24747 23814 26216 CDS product ribonucleotide reductase subunit 1 24716-24747 7:04:01 GGCGGCGGTGGCGGCCGCCGTGGACAGCGCGG
25006 25035 23814 26216 CDS product ribonucleotide reductase subunit 1 25006-25035 5:02:01 CCTCGACGCCCAGCCCCTCGCGCTCCAGCC
25072 25091 23814 26216 CDS product ribonucleotide reductase subunit 1 25072-25091 4:01:01 GGCTGAGGTGCGAGGCGCGG
25132 25148 23814 26216 CDS product ribonucleotide reductase subunit 1 25132-25148 4:01:01 GGTAGGCGAGGTAGCGG
25456 25469 23814 26216 CDS product ribonucleotide reductase subunit 1 25456-25469 4:01:01 CCTCCACGGCCTCC
25640 25665 23814 26216 CDS product ribonucleotide reductase subunit 1 25640-25665 4:01:01 CCCGAAGGCCGGCTCCCGCATCGTCC
25704 25726 23814 26216 CDS product ribonucleotide reductase subunit 1 25704-25726 4:01:01 CCCATGGACTCCAGCGCCCCGCC
26036 26079 23814 26216 CDS product ribonucleotide reductase subunit 1 26036-26079 12:09:03 GGCGCGGGAGGGCTGGGAGGCGGCGGCGGGCGGCGGCGGCACGG
26082 26105 23814 26216 CDS product ribonucleotide reductase subunit 1 26082-26105 5:02:01 CCGCAGCCCAGCCGCGCCCACTCC
26138 26151 23814 26216 CDS product ribonucleotide reductase subunit 1 26138-26151 4:01:01 GGCCGGGCAGGCGG
26594 26632 26581 28014 CDS product capsid triplex subunit 1 26594-26632 8:05:02 CCACCCAGTCCCCGGCCCCCCACACCAGGCCCTCGATCC
26744 26768 26581 28014 CDS product capsid triplex subunit 1 26744-26768 5:02:01 GGGTGGGCCGCCGGGCGGCGCTCGG

26820 26841 26581 28014 CDS product capsid triplex subunit 1 26820-26841 4:01:01 GGGGCAGCGGTAGGCCGCGCGG
26873 26914 26581 28014 CDS product capsid triplex subunit 1 26873-26914 11:08:02
CCGCGGCCCCGTCCGCCCCCGCCTCCTCGCCCGCCGTCCCCC
27114 27153 26581 28014 CDS product capsid triplex subunit 1 27114-27153 7:04:01
GGCCGGCACGTGGGCCGTGGAGCGCGGGTGTGCGGCCTGG
27234 27256 26581 28014 CDS product capsid triplex subunit 1 27234-27256 4:01:01 CCCGCCCAGGCACCCAAACGCCC
27257 27298 26581 28014 CDS product capsid triplex subunit 1 27257-27298 7:04:01
GGTGCGGGAACGCGTGGCAGCGGATCATGGCCTGCAGGCAGG
27416 27439 26581 28014 CDS product capsid triplex subunit 1 27416-27439 5:02:01 GGAAGGAAAAGGCCACGAGGGAGG
27440 27468 26581 28014 CDS product capsid triplex subunit 1 27440-27468 6:03:01
CCCGCAGCCCCCCGGGCGCCGCGCCGCCC
27528 27547 26581 28014 CDS product capsid triplex subunit 1 27528-27547 6:03:01 GGGGGGCGCGGTGGCGGCGG
27673 27698 26581 28014 CDS product capsid triplex subunit 1 27673-27698 6:03:01
GGCGAAAGGACGGCGGCGCCGGCGGG
27752 27772 26581 28014 CDS product capsid triplex subunit 1 27752-27772 4:01:01 GGATGGTCACGGTCGCGTCGG
27808 27855 26581 28014 CDS product capsid triplex subunit 1 27808-27855 9:06:02
CCCCGGGGCGCCCGCCCGCGGGCCCCGCTGCCCCCAGGAGCCGCCACC
27873 27901 26581 28014 CDS product capsid triplex subunit 1 27873-27901 6:03:01
GGCGGCCGCCGCGGCGGAAGCGGCGCCGG
27934 27951 26581 28014 CDS product capsid triplex subunit 1 27934-27951 4:01:01 GGCGACGCGGCGGCCGGG
28342 28359 28300 31464 CDS product tegument protein UL37 28342-28359 4:01:01 GGCTGGACGGCGACGCGG
28360 28376 28300 31464 CDS product tegument protein UL37 28360-28376 4:01:01 CCGCCGACCCCGCGGCC
28385 28398 28300 31464 CDS product tegument protein UL37 28385-28398 4:01:01 GGAGGCCAGGGCGG

28582	28616	28300	31464	CDS product tegument protein UL37		28582-28616 7:04:01
CCGCCGCCCTCATGGCCGGCGTCCGGGACCTGGCC
28595	28621	28300	31464	CDS product tegument protein UL37		28595-28621 5:02:01 GGCCGGCGTCCGGGACCTGGCCAACGG
28634	28664	28300	31464	CDS product tegument protein UL37		28634-28664 5:02:01
GGCGCCGCTGGGCTACCTGGCGGCCGCGCGG
28676	28692	28300	31464	CDS product tegument protein UL37		28676-28692 4:01:01 GGCGGCGTTCGGCCCGG
28709	28721	28300	31464	CDS product tegument protein UL37		28709-28721 4:01:01 GGTGGTGGAGTGG
28754	28780	28300	31464	CDS product tegument protein UL37		28754-28780 4:01:01 GGACGCGGCGTGCGTGGGCGTGCTGGG
28781	28804	28300	31464	CDS product tegument protein UL37		28781-28804 4:01:01 CCCCGACCCCGCGCCCCGCTCGCC
28915	28943	28300	31464	CDS product tegument protein UL37		28915-28943 5:02:01
GGAAGGTGCCCATGGCTGCCGTGGCGCGG
28948	28977	28300	31464	CDS product tegument protein UL37		28948-28977 5:02:01
CCGCGGACGCCGCGGCGCCGCCGCTGGCCC
29045	29067	28300	31464	CDS product tegument protein UL37		29045-29067 5:02:01 GGGGCTGGCGGAGGCCTTCGCGG
29093	29115	28300	31464	CDS product tegument protein UL37		29093-29115 4:01:01 GGCGCTGGTGCCCGTGGGCGAGG
29289	29337	28300	31464	CDS product tegument protein UL37		29289-29337 9:06:02
GGCGCGGTGGCGCGGCTGCTGGAGCGCGCGGGCGCGGACGCGGGCAGGG
29351	29397	28300	31464	CDS product tegument protein UL37		29351-29397 9:06:02
GGCGGCGGCCCTGGCGCGGGTCGCGGAGCACGCGGCGGCCGTCTGGG
29423	29456	28300	31464	CDS product tegument protein UL37		29423-29456 6:03:01
GGACCAGGCCGTGGAGACGCTGGCCGCGGCGGGG
29513	29535	28300	31464	CDS product tegument protein UL37		29513-29535 4:01:01 CCCGGAGCCGCGGCCGCGCGCCC
29552	29581	28300	31464	CDS product tegument protein UL37		29552-29581 8:05:02

GGCGGTGGGCTGCGTGGCGGTGGCGGGCGG

29780 29824 28300 31464 CDS product tegument protein UL37 29780-29824 7:04:01 GGCACTCGTGGCCGAGGCCGCGGCCGCGGACAAGCGGGCCGCGGG

29836 29892 28300 31464 CDS product tegument protein UL37 29836-29892 13:10:03 GGGCGGCCGGCGAGGGCGGCGGCGGCGACCGGGCAGAGGCGGCGGCGGCCGCTAGGG

29956 29969 28300 31464 CDS product tegument protein UL37 29956-29969 4:01:01 CCCCCCCCGCGGCC

30096 30146 28300 31464 CDS product tegument protein UL37 30096-30146 8:05:02 CCCGCTGCCGTCCTGCGGGACCCGGCCTTCGCGCCCTACTTCGCCGCTGCC

30229 30278 28300 31464 CDS product tegument protein UL37 30229-30278 9:06:02 GGGCGCGGGACTGCGGTGCCGGGGCGGTGGCCAACGTGGACGGCTACCGG

30290 30311 28300 31464 CDS product tegument protein UL37 30290-30311 5:02:01 GGCCCTGGCGGCCGGGCTCTGG

30392 30411 28300 31464 CDS product tegument protein UL37 30392-30411 5:02:01 GGCGGCCGTGGCCGCGGCGG

30434 30454 28300 31464 CDS product tegument protein UL37 30434-30454 4:01:01 CCCGCCCGTGCCCGCGCGCCC

30547 30581 28300 31464 CDS product tegument protein UL37 30547-30581 7:04:01 CCGCCTCCATGGCCGCGGCCGTGGGCGCCGTCGCC

30582 30603 28300 31464 CDS product tegument protein UL37 30582-30603 6:03:01 GGCGCGGTGGCGCGGCTGGGGG

30650 30670 28300 31464 CDS product tegument protein UL37 30650-30670 4:01:01 GGCGGTGTACGCGGCGCGTGG

30686 30727 28300 31464 CDS product tegument protein UL37 30686-30727 7:04:01 GGGCACGTGGCGCTCGGCGGACCTGGCCGACGCGGTGCGCGG

30812 30837 28300 31464 CDS product tegument protein UL37 30812-30837 4:01:01 GGCGCTGCAGGAGTGCGAGGCCGCGG

30832 30850 28300 31464 CDS product tegument protein UL37 30832-30850 4:01:01 CCGCGGACGCCCGCCCCCC

30852 30864 28300 31464 CDS product tegument protein UL37 30852-30864 4:01:01 GGCGGCGGGCTGG

30887 30940 28300 31464 CDS product tegument protein UL37 30887-30940 9:06:02

GGCGGGCCACTCGGCGCTGGTGCGGGCGCAGACGGCGCTGGCGCTGGCCGCCGG
30950 30971 28300 31464 CDS product tegument protein UL37 30950-30971 5:02:01 GGCGGGCGCGGAGGCGCCGGGG
31081 31113 28300 31464 CDS product tegument protein UL37 31081-31113 6:03:01
GGCTCCGCGGCGTCTGGGACGAGGTCCAGGAGG
31124 31140 28300 31464 CDS product tegument protein UL37 31124-31140 4:01:01 GGCGGCCCCGGGGCCGG
31172 31242 28300 31464 CDS product tegument protein UL37 31172-31242 11:08:02
GGAGGCGGTGCTGGCGCTGCTGGAGGGCTACTCGGAAGTCCAGGGCGACGAGGGGAGCCCGGCGCTCTTGG
31253 31285 28300 31464 CDS product tegument protein UL37 31253-31285 6:03:01
GGACGTGGCCGACTGGGCGGGCGTGGACCGCGG
31320 31339 28300 31464 CDS product tegument protein UL37 31320-31339 5:02:01 GGCGGCAACGGCGACGGCGG
31349 31382 28300 31464 CDS product tegument protein UL37 31349-31382 9:06:02
GGCGGCGGGGGCGGACGCGGCGGCGGGGCCGTGG
31649 31674 31564 41178 CDS product large tegument protein 31649-31674 5:02:01 GGCGCTAGGGCCGGGCAGCGCGGTGG
31705 31723 31564 41178 CDS product large tegument protein 31705-31723 4:01:01 GGCTGGCCTTCGCGGGCGG
31769 31785 31564 41178 CDS product large tegument protein 31769-31785 4:01:01 GGAGGGCGCGGCGTGGG
31829 31851 31564 41178 CDS product large tegument protein 31829-31851 5:02:01 CCTGCCCAACCGCATCGCCGACC
31852 31871 31564 41178 CDS product large tegument protein 31852-31871 4:01:01 GGGCGGACGCGGACAGCGGG
32003 32035 31564 41178 CDS product large tegument protein 32003-32035 5:02:01
GGCGCTGGTGATCGTGGGCGCCATGGGCGTGGG
32176 32193 31564 41178 CDS product large tegument protein 32176-32193 4:01:01 GGTGGGCGGGCGCGCTGG
32360 32397 31564 41178 CDS product large tegument protein 32360-32397 8:05:02
GGCCGTGGCGGTGGGCGGCGCCGGGGCGGAGTGCGAGG
32414 32446 31564 41178 CDS product large tegument protein 32414-32446 6:03:01

CCCGGGCTCGCCGGCGCTGCCGCCCGCCGAGCC

32474 32507 31564 41178 CDS product large tegument protein 32474-32507 6:03:01

GGCCGCGGCCGCGGCCCGCGCGGCGGGAAAGCGG

32521 32540 31564 41178 CDS product large tegument protein 32521-32540 5:02:01 GGCGGCGGCGGGCCCCGTGG

32533 32565 31564 41178 CDS product large tegument protein 32533-32565 5:02:01

CCCCGTGGACGCCGCCCTCGAGCCGCGAGGACC

32608 32634 31564 41178 CDS product large tegument protein 32608-32634 6:03:01 GGAAGGTCCGGGGCGCGGAGTCGGCGG

32643 32682 31564 41178 CDS product large tegument protein 32643-32682 9:06:02

GGGGAGGGCTGGGGAGGGGCGGGGGTGGGAGCGGGAGAGG

32686 32711 31564 41178 CDS product large tegument protein 32686-32711 6:03:01 CCGCCCTGGCGCCCCGACCGCCGCCC

32717 32736 31564 41178 CDS product large tegument protein 32717-32736 5:02:01 GGGCGCCGGGGCGGGGGAGG

32747 32767 31564 41178 CDS product large tegument protein 32747-32767 4:01:01 GGCCTGCGCGGCGGAGCTTGG

32792 32823 31564 41178 CDS product large tegument protein 32792-32823 6:03:01

GGCCCTGGAGGGCGTCCGGGCGCACGCGGCGG

32988 33004 31564 41178 CDS product large tegument protein 32988-33004 4:01:01 CCCTCGGCCGTCCAGCC

33012 33027 31564 41178 CDS product large tegument protein 33012-33027 4:01:01 GGGGCGGTGGCCCAGG

33072 33093 31564 41178 CDS product large tegument protein 33072-33093 4:01:01 GGGATGGTGCTCGAGGGGCTGG

33106 33117 31564 41178 CDS product large tegument protein 33106-33117 4:01:01 GGCTGGCGGAGG

33181 33197 31564 41178 CDS product large tegument protein 33181-33197 4:01:01 CCGCCGTCGCGGCCGCC

33221 33271 31564 41178 CDS product large tegument protein 33221-33271 10:07:02

GGACGAGCTGGAGGGCGAGGTGGACGGCGCCGCCGGCGGCGGCGACGCGGG

33317 33333 31564 41178 CDS product large tegument protein 33317-33333 4:01:01 CCTCTCGGCCTCCGGCC

33323 33338 31564 41178 CDS product large tegument protein 33323-33338 4:01:01 GGCCTCCGGCCGGCGG

33415 33443 31564 41178 CDS product large tegument protein 33415-33443 5:02:01
CCTGCGAGGCCCGCGCAGCCGCCGAGACC
33467 33483 31564 41178 CDS product large tegument protein 33467-33483 4:01:01 GGCGCTGGAGGGCACGG
33503 33529 31564 41178 CDS product large tegument protein 33503-33529 5:02:01 CCTGGACGCCGTCCCGCCCGACGACCC
33536 33570 31564 41178 CDS product large tegument protein 33536-33570 7:04:01
GGCGCCCGCGGACGGCGCGGTGGCCGACCTGGCGG
33613 33629 31564 41178 CDS product large tegument protein 33613-33629 5:02:01 CCGCCGCCCTGTCCGCC
33794 33817 31564 41178 CDS product large tegument protein 33794-33817 6:03:01 GGCGGCGCGGGTGGCCCGGCTTGG
33822 33850 31564 41178 CDS product large tegument protein 33822-33850 7:04:01
CCGCCCGGCGGCGCCGCCCCGCCGCCGCC
33890 33906 31564 41178 CDS product large tegument protein 33890-33906 4:01:01 GGCGGCGGGCGCAACGG
33921 33939 31564 41178 CDS product large tegument protein 33921-33939 4:01:01 GGCCTCGCGGCCTGGGTGG
33953 33967 31564 41178 CDS product large tegument protein 33953-33967 4:01:01 GGCGCAGGCGGAGGG
34023 34062 31564 41178 CDS product large tegument protein 34023-34062 6:03:01
GGGCGCTCGGCCTCGCGGGCGTCGCTGGAGGCTAGCATGG
34081 34112 31564 41178 CDS product large tegument protein 34081-34112 8:05:02
CCGCCGCCGCCTCCCGCGCTGCCGCCGCGGCC
34128 34191 31564 41178 CDS product large tegument protein 34128-34191 14:11:03
GGCGCGGGCGGCGGCGGCACGGGGCCCCTGGGCCCGGGGGCCGAGGAGGCGGCGGATGCCGCGG
34186 34211 31564 41178 CDS product large tegument protein 34186-34211 5:02:01 CCGCGGACGCCGCCGTCCGCGGCGCC
34232 34257 31564 41178 CDS product large tegument protein 34232-34257 5:02:01 GGACGCCCTGGCGACGGGCGCGGCGG
34283 34362 31564 41178 CDS product large tegument protein 34283-34362 15:12:03
GGCCGCGGCCCGCGCGGAGCTGGAGGCGCAGGCGGCCGCGGCGCGGCGGCGCGCGGCCGAGCTGGGGGAGCGGCGCGCGG

34424 34445 31564 41178 CDS product large tegument protein 34424-34445 4:01:01 GGCACCCGGCGCGCTGGAGCGG

34454 34479 31564 41178 CDS product large tegument protein 34454-34479 6:03:01 GGACGCGGGCGCGCGGACGGCGGAGG

34523 34544 31564 41178 CDS product large tegument protein 34523-34544 4:01:01 GGGCGTGCAGGACCGGCTGTGG

34568 34642 31564 41178 CDS product large tegument protein 34568-34642 13:10:03 GGCGCTGGAGAACCCGGGCGCGCTGGCGCCGGGGGCGCTGGCCGGGCTGGGCCCGGCCTTCGCGGCGGTGCTGGG

34652 34674 31564 41178 CDS product large tegument protein 34652-34674 4:01:01 GGGACAGGCGCTGGGCCCTGCGG

34730 34755 31564 41178 CDS product large tegument protein 34730-34755 5:02:01 GGCGGCGGAGCCCGCGGCGCCCGCGG

34740 34763 31564 41178 CDS product large tegument protein 34740-34763 5:02:01 CCCGCGGCGCCCGCGGCCGCCGCC

34790 34820 31564 41178 CDS product large tegument protein 34790-34820 6:03:01 GGCGGAGGCGCTGCGGGCCAGGGGCGCGGGG

34823 34842 31564 41178 CDS product large tegument protein 34823-34842 4:01:01 CCCGCCCGAGTTCGCCTTCC

34890 34906 31564 41178 CDS product large tegument protein 34890-34906 4:01:01 GGCGCGCGGCGGCTGGG

34919 34939 31564 41178 CDS product large tegument protein 34919-34939 4:01:01 GGCCGTGGGCGAGGCCTCGGG

34979 35070 31564 41178 CDS product large tegument protein 34979-35070 18:15:04 GGCGGCGGGCGCGGACGCGGCGGAGACGGCGGCAGCGACGGCCGCGGCGGACGCGGCGCTGGGCGCCGCGGAGGAGGTGCTGCGGGCCGCGG

35081 35124 31564 41178 CDS product large tegument protein 35081-35124 9:06:02 GGCCGAGGCCGCTGGCGGCGAGGACGCGGAAGGCGCGGGCGAGG

35135 35148 31564 41178 CDS product large tegument protein 35135-35148 4:01:01 GGACGGCGAGGCGG

35152 35170 31564 41178 CDS product large tegument protein 35152-35170 4:01:01 CCGCCGGGCGCCTGGGGCC

35157 35178 31564 41178 CDS product large tegument protein 35157-35178 4:01:01 GGGCGCCTGGGGCCGCAGGCGG

35204 35225 31564 41178 CDS product large tegument protein 35204-35225 5:02:01 GGCCCAGGCGCTGGCGCGGCGG

35240 35261 31564 41178 CDS product large tegument protein 35240-35261 4:01:01 CCGGCGCCTGCGCGCCGAGGCC

35293 35316 31564 41178 CDS product large tegument protein 35293-35316 5:02:01 GGCAGGAGGAGCGCTGGCGCGAGG

35419 35454 31564 41178 CDS product large tegument protein 35419-35454 7:04:01
GGCCGCTGGCGCGGCAGGCCGACCGGGCGCTGGAGG
35467 35487 31564 41178 CDS product large tegument protein 35467-35487 4:01:01 GGGCGACGGCGGCCATCGAGG
35684 35698 31564 41178 CDS product large tegument protein 35684-35698 4:01:01 GGCCTCGGGCGGCGG
35732 35748 31564 41178 CDS product large tegument protein 35732-35748 4:01:01 GGAGGGCGACCTGGCGG
35741 35765 31564 41178 CDS product large tegument protein 35741-35765 4:01:01 CCTGGCGGCCGTGCCCCGGCTCGCC
35788 35811 31564 41178 CDS product large tegument protein 35788-35811 5:02:01 GGGGGCACGCGGCTTTCGAGGAGG
35852 35874 31564 41178 CDS product large tegument protein 35852-35874 6:03:01 GGCGGCCGGCGGGCGCGCGGCGG
36014 36038 31564 41178 CDS product large tegument protein 36014-36038 4:01:01 CCACGCCCGCTTCGCCGGCTCGGCC
36077 36132 31564 41178 CDS product large tegument protein 36077-36132 10:07:02
GGCCGCGGCGCGAGAGGCGCTAGAGGCGGCAAAGGCGGCGCGCGCGGAGGCCACGG
36153 36177 31564 41178 CDS product large tegument protein 36153-36177 6:03:01 GGCGAGGTCGTGGCGGCCGAGGCGG
36199 36222 31564 41178 CDS product large tegument protein 36199-36222 4:01:01 CCGGGCTGGCCAACCTCAAGAACC
36269 36287 31564 41178 CDS product large tegument protein 36269-36287 4:01:01 CCGCGCCGCCTCGGCGGCC
36317 36365 31564 41178 CDS product large tegument protein 36317-36365 11:08:02
GGCGGCCGTGGAGGAGGCGCCGGAGCTGGACGTGGCCGCGGTGGAGTGG
36496 36540 31564 41178 CDS product large tegument protein 36496-36540 10:07:02
GGCGGCAGCTGGAGTCGGCGGCGGCGGCGTGGGACGGCGCGTGGG
36584 36612 31564 41178 CDS product large tegument protein 36584-36612 4:01:01
GGACCACGAGGGCGCCAAGGCGCGCGCGG
36623 36647 31564 41178 CDS product large tegument protein 36623-36647 5:02:01 GGCGGCCGCGGGCGTGGTGCTGGGG
36693 36724 31564 41178 CDS product large tegument protein 36693-36724 6:03:01
GGCGCGCTGGACGCGCGGCTGGCGGAGCGCGG

36739 36761 31564 41178 CDS product large tegument protein 36739-36761 4:01:01 CCTTCCACGAGACCGCGCGCGCC

36800 36816 31564 41178 CDS product large tegument protein 36800-36816 4:01:01 GGCGGTGCGCGGCGAGG

36878 36894 31564 41178 CDS product large tegument protein 36878-36894 4:01:01 GGCCTGGGCGGCGCAGG

36903 36924 31564 41178 CDS product large tegument protein 36903-36924 4:01:01 CCCTTCCGGGACCTGCTGGTCC

36987 37008 31564 41178 CDS product large tegument protein 36987-37008 5:02:01 GGCGCGGCCGCGGCTCGGGCGG

37107 37143 31564 41178 CDS product large tegument protein 37107-37143 7:04:01

GGCCGCGTGGCGGCGCTCCTGGGCGACGAGGCGGCGG

37310 37341 31564 41178 CDS product large tegument protein 37310-37341 8:05:02

GGACAACGGCGGCGTGGTGGAGGCGGCCTCGG

37510 37530 31564 41178 CDS product large tegument protein 37510-37530 5:02:01 CCGCCACGCTGCCCCGCCGCC

37568 37636 31564 41178 CDS product large tegument protein 37568-37636 14:11:03

GGCCGAGCGGGGGCTGCGGCCGGTGCGGGCGCCGCTGGGCGGCGGCAGCGGCGGCGGCGAGGGGCGCGG

37646 37657 31564 41178 CDS product large tegument protein 37646-37657 4:01:01 GGGCGGCGGCGG

37778 37818 31564 41178 CDS product large tegument protein 37778-37818 8:05:02

GGACGGTGTCCTGGCCGAGGCGCTCGCGGGCCGGGTGGCGG

37839 37897 31564 41178 CDS product large tegument protein 37839-37897 13:10:03

GGCGGCGGCGCGCGGCGGCCGGTGCCGCTGGGCGGCGGGGCGGACGACCCGGCGGACGG

37909 37922 31564 41178 CDS product large tegument protein 37909-37922 4:01:01 CCATCCGCGCCGCC

37931 37952 31564 41178 CDS product large tegument protein 37931-37952 5:02:01 CCCGGGCCGCCTCTCCCCGTCC

38114 38136 31564 41178 CDS product large tegument protein 38114-38136 4:01:01 GGTGGACTCGCTGGGCGCGCAGG

38152 38169 31564 41178 CDS product large tegument protein 38152-38169 4:01:01 CCTTCCTGGCGCGCCGCC

38198 38238 31564 41178 CDS product large tegument protein 38198-38238 8:05:02

GGGCGGGCCCTCGGAGGACGCGGCGGCGCTGGCCGCAGCGG

38230 38249 31564 41178 CDS product large tegument protein 38230-38249 4:01:01 CCGCAGCGGCCAGCGCCGCC
38372 38397 31564 41178 CDS product large tegument protein 38372-38397 4:01:01 GGGCGCGCCGGTCGTCGTGGCCATGG
38479 38508 31564 41178 CDS product large tegument protein 38479-38508 5:02:01
CCGACGCGCCTGCCGCGGTGTCCTCGGACC
38518 38535 31564 41178 CDS product large tegument protein 38518-38535 4:01:01 GGGGGGAGCGGCTGCTGG
38623 38637 31564 41178 CDS product large tegument protein 38623-38637 4:01:01 CCGCGCCGCCCTGCC
38689 38701 31564 41178 CDS product large tegument protein 38689-38701 4:01:01 CCGCCGCGCCGCC
38738 38754 31564 41178 CDS product large tegument protein 38738-38754 4:01:01 CCGGGACCTGCCGAGCC
38781 38806 31564 41178 CDS product large tegument protein 38781-38806 6:03:01 CCCCCCGCAGCCGACCCCGCCGACCC
38878 38920 31564 41178 CDS product large tegument protein 38878-38920 8:05:02
CCCACCCCCCCGTCTTCCGGCACGCCGCCGGCGACCCGTTTCC
38929 38953 31564 41178 CDS product large tegument protein 38929-38953 7:04:01 GGCCGGCGGCCGGCGGCGGCAGCGG
38957 39062 31564 41178 CDS product large tegument protein 38957-39062 18:15:04
CCGCGCGGCCAACGCCCGCGCGGCCGCGTGCCCCCAAGGCCGGACCCCGGCCCCAGCTCCGGCCGCGCCTCCGGCCCCGGCGGAGCCCGCCCTCGCCTGGGGGGCC
39055 39075 31564 41178 CDS product large tegument protein 39055-39075 5:02:01 GGGGGGCCGAGGAGTGGCTGG
39118 39137 31564 41178 CDS product large tegument protein 39118-39137 4:01:01 CCGCCTACTCGCCCGAGCCC
39195 39230 31564 41178 CDS product large tegument protein 39195-39230 7:04:01
CCTGCCCGTACGCCGGCACCTTACCCGCCCGAGCCC
39265 39284 31564 41178 CDS product large tegument protein 39265-39284 5:02:01 CCGCCTACCGGCCCGGGTCC
39274 39295 31564 41178 CDS product large tegument protein 39274-39295 4:01:01 GGCCCGGGTCCGCAGGGGACGG
39299 39328 31564 41178 CDS product large tegument protein 39299-39328 7:04:01
CCCACCAGAGTCCGCGCCCGCCCCCACGCC
39338 39368 31564 41178 CDS product large tegument protein 39338-39368 8:05:02

CCCGCCAGAGCCCGCGCCCACCCCCGCCCCC
39405 39419 31564 41178 CDS product large tegument protein 39405-39419 4:01:01 CCCGCCCCCGAGGCC
39449 39472 31564 41178 CDS product large tegument protein 39449-39472 5:02:01 CCGCCTGACGCGCCGCACCGGGCC
39509 39538 31564 41178 CDS product large tegument protein 39509-39538 6:03:01
CCCGCTGCCCGCCGCGCCCCCGGCGGCTCC
39548 39631 31564 41178 CDS product large tegument protein 39548-39631 17:14:04
CCCGCCGCCCGCGGCCTTGCCGCCGACGGCCGCCGGTGCCGCGGCGTCCCCAGCCTCGCCGCCCGCCGCGCCCCCAGATTCTCC
39641 39680 31564 41178 CDS product large tegument protein 39641-39680 9:06:02
CCCGCCGCCCGCGGTCCCGCCGCCCGCGGCCGCCAGTACC
39690 39722 31564 41178 CDS product large tegument protein 39690-39722 9:06:02
CCAGCCTCGCCGCCAGCGCCCCCGCCGCCTCCC
39733 39772 31564 41178 CDS product large tegument protein 39733-39772 9:06:02
CCCCGGGCACCGCTCCGCCGCCACCTCCCTCGTTGCCGCC
39801 39827 31564 41178 CDS product large tegument protein 39801-39827 5:02:01 CCCGGCGCCCAGACGGTCCGCCGGCCC
39815 39836 31564 41178 CDS product large tegument protein 39815-39836 4:01:01 GGTCCGCCGGCCCGGGGCTCGG
39869 39892 31564 41178 CDS product large tegument protein 39869-39892 6:03:01 CCCGCCGCCCCCGTTTTTGCCGCC
39899 39937 31564 41178 CDS product large tegument protein 39899-39937 8:05:02
GGGGGCGCCGGCGGACGGTCCGCCGGCCCGGGGCTCGGG
39946 40060 31564 41178 CDS product large tegument protein 39946-40060 23:20:05
CCGGCCGCGCCGCCGAGCCCGCCCCGCCCCTGCCAGCCGTTAGCCCGCCCGGGCCACCCGCCGTCTCCCCACCGCCCCGGCAGCCCCGCCCAAGCCCCGCGGACGCCGGCCCCCC
40046 40068 31564 41178 CDS product large tegument protein 40046-40068 4:01:01 GGACGCCGGCCCCCCAGGTGCGG
40071 40152 31564 41178 CDS product large tegument protein 40071-40152 18:15:04
CCCGCGCCCGCTCCGCCCGCTCCGCCCGCTCCGCCCGCTCCGCCCGCTCCGCCCGCTCCGCCCGCGCCACCGAGCCAACCCC

40153 40178 31564 41178 CDS product large tegument protein 40153-40178 5:02:01 GGGCGGACGGGCCGGCACCGCCGGGG
40164 40183 31564 41178 CDS product large tegument protein 40164-40183 5:02:01 CCGGCACCGCCGGGGCCGCC
40206 40237 31564 41178 CDS product large tegument protein 40206-40237 6:03:01 CCGGCGGCCCCGGCGCCCCCAAGGGCCCCCCC
40253 40283 31564 41178 CDS product large tegument protein 40253-40283 6:03:01 CCCGGCGCCGCCGAAGCCCAGACCCGCGCCC
40293 40313 31564 41178 CDS product large tegument protein 40293-40313 4:01:01 CCCGGCGCGCCCGCCAAGCCC
40391 40411 31564 41178 CDS product large tegument protein 40391-40411 4:01:01 GGGCCCCGGCCCCGCGGACGG
40448 40498 31564 41178 CDS product large tegument protein 40448-40498 11:08:02 CCCGCCACCCGACCCCGCGACCCAGCCGGCCGGGCCGGCGCCAGCGCCTCC
40505 40536 31564 41178 CDS product large tegument protein 40505-40536 6:03:01 GGCCGCGGCGCTTCCGGCCGCGGCCCGGCCGG
40518 40576 31564 41178 CDS product large tegument protein 40518-40576 12:09:03 CCGGCCGCGGCCCGGCCGGCGCCCGCGACCCAGCCGGCCGGGCCGGCGCCAGCGCCTCC
40583 40632 31564 41178 CDS product large tegument protein 40583-40632 8:05:02 GGCCGCGGCGCCTCCGGCCGCGGCCCGGCTGGCGCCCCCGGCCCAGCCGG
40585 40608 31564 41178 CDS product large tegument protein 40585-40608 5:02:01 CCGCGGCGCCTCCGGCCGCGGCCC
40617 40639 31564 41178 CDS product large tegument protein 40617-40639 6:03:01 CCCCCGGCCCAGCCGGCGCCTCC
40653 40669 31564 41178 CDS product large tegument protein 40653-40669 5:02:01 CCCGCCGCCCCCCGCCC
40673 40689 31564 41178 CDS product large tegument protein 40673-40689 4:01:01 GGATGTGGGCGGCCGGG
40685 40708 31564 41178 CDS product large tegument protein 40685-40708 4:01:01 CCGGGCGCGCCCGACGGCCCGCCC
40814 40832 31564 41178 CDS product large tegument protein 40814-40832 4:01:01 GGCGGACGCCGAGGCGCGG
40845 40889 31564 41178 CDS product large tegument protein 40845-40889 9:06:02

CCCGCGCCCCCCGAGCCCCCCGTCGACCCCGGCCTCGACACCGCC

40916 40947 31564 41178 CDS product large tegument protein 40916-40947 7:04:01 GGAGACGGAGACGGAGGCGGAAGAGACGGAGG

40956 40974 31564 41178 CDS product large tegument protein 40956-40974 4:01:01 GGCCGCGGCCCAGGCCGGG

41418 41436 41405 41782 CDS product small capsid protein 41418-41436 4:01:01 CCGAGGCCGAAGCCGCGCC

41664 41681 41405 41782 CDS product small capsid protein 41664-41681 4:01:01 GGAGCGGGCGGGACGCGG

41707 41758 41405 41782 CDS product small capsid protein 41707-41758 9:06:02 GGGCAGCAGGTCCCGTAGGGTGTCGGGGGTGATGGTGGCCGGCGCGCTGGGG

41866 41911 41827 42657 CDS product nuclear egress membrane protein 41866-41911 8:05:02 CCAAAGACTCCGCCCACAAGCGCCGCGAGGACCGCGAGCCCCCGCC

41948 41977 41827 42657 CDS product nuclear egress membrane protein 41948-41977 7:04:01 GGCTCCCGGCGCCCCCGGCGGCGGCGGCGG

42079 42116 41827 42657 CDS product nuclear egress membrane protein 42079-42116 7:04:01 CCCAGCCGCTCCTGGACCGCGCGGTCCAGCAGCCGGCC

42201 42219 41827 42657 CDS product nuclear egress membrane protein 42201-42219 4:01:01 GGCGCGGAATGGCCGCGGG

42410 42436 41827 42657 CDS product nuclear egress membrane protein 42410-42436 4:01:01 CCGTCTGCGCCCAGCTGGCCATCAGCC

42507 42536 41827 42657 CDS product nuclear egress membrane protein 42507-42536 6:03:01 GGGCGGGCGGCGCGGGTCCCAGGGCTGGGG

42596 42622 41827 42657 CDS product nuclear egress membrane protein 42596-42622 5:02:01 GGCTCCGGGAGGAGCCCTCGGCATCGG

42799 42812 42738 43070 CDS product DNA packaging protein UL33 42799-42812 4:01:01 CCTTCCTCGCCTCC

42839 42853 42738 43070 CDS product DNA packaging protein UL33 42839-42853 4:01:01 CCGCCCGCCCGGGCC

42865 42890 42738 43070 CDS product DNA packaging protein UL33 42865-42890 4:01:01 CCGAGGCCAGGCGCGCCGTGAAGGCC
42905 42919 42738 43070 CDS product DNA packaging protein UL33 42905-42919 4:01:01 GGCGTCGGTGGTGGG
42946 42968 42738 43070 CDS product DNA packaging protein UL33 42946-42968 4:01:01 CCACGTCCTCGAACCAGATCTCC
43009 43040 42738 43070 CDS product DNA packaging protein UL33 43009-43040 5:02:01 CCAGCTCGGCCACGTCCTCGGTGGCCAGCGCC
43089 43108 43051 44847 CDS product DNA packaging protein UL32 43089-43108 5:02:01 GGCGCCGGCGGCGGCGAGGG
43120 43138 43051 44847 CDS product DNA packaging protein UL32 43120-43138 4:01:01 GGGCGGAGGGCGCGTTCGG
43223 43242 43051 44847 CDS product DNA packaging protein UL32 43223-43242 4:01:01 GGAGGTGCCCGGCCCGCGGG
43256 43299 43051 44847 CDS product DNA packaging protein UL32 43256-43299 10:07:02 GGAGGGGGACGCGGACGCGGACGGGGAGGGGGGCGGCGCCGCGG
43324 43351 43051 44847 CDS product DNA packaging protein UL32 43324-43351 6:03:01 CCGCCGCCGCGCTGGCCATCGACCGGCC
43404 43427 43051 44847 CDS product DNA packaging protein UL32 43404-43427 4:01:01 CCCCCCTGGGTCGCCGACTACGCC
43455 43472 43051 44847 CDS product DNA packaging protein UL32 43455-43472 4:01:01 CCGCCCTGCGCCGTGGCC
43502 43538 43051 44847 CDS product DNA packaging protein UL32 43502-43538 6:03:01 CCTGATGGACCGGCACTTCCTGGCCGCGCACCGCGCC
43622 43648 43051 44847 CDS product DNA packaging protein UL32 43622-43648 7:04:01 GGACGGCGGCGTTCCCGGCGGCGGCGG
43659 43683 43051 44847 CDS product DNA packaging protein UL32 43659-43683 4:01:01 GGCGCCGCTGGGGGGGCGCGCCGGG
43730 43758 43051 44847 CDS product DNA packaging protein UL32 43730-43758 4:01:01 GGCGACGCGGGCGCTGTTGGCGTCGACGG

43769 43789 43051 44847 CDS product DNA packaging protein UL32 43769-43789 5:02:01 GGAGGCGGCGCCCGCGGACGG
43887 43936 43051 44847 CDS product DNA packaging protein UL32 43887-43936 12:09:03 GGCGGCTGCGGCGGTGGCGGCGGCGCGCGGGCGGCGCGGCGTCCCGGGGG
44022 44058 43051 44847 CDS product DNA packaging protein UL32 44022-44058 8:05:02 CCCGCCGGCGCCCCGCCGGCGCCGCCGCCCCGCGGACC
44054 44073 43051 44847 CDS product DNA packaging protein UL32 44054-44073 4:01:01 GGACCGGTGGGCGTACGCGG
44146 44166 43051 44847 CDS product DNA packaging protein UL32 44146-44166 5:02:01 CCCGCCGCACCGCCGTCGACC
44261 44280 43051 44847 CDS product DNA packaging protein UL32 44261-44280 4:01:01 GGGGCCGGTGCTGGCCACGG
44457 44482 43051 44847 CDS product DNA packaging protein UL32 44457-44482 5:02:01 GGCGCGCCGGTCGAAGGCGACGGCGG
44726 44750 43051 44847 CDS product DNA packaging protein UL32 44726-44750 5:02:01 CCAGCTCTTCCCCCCGAAGCCAACC
44851 44884 44840 45979 CDS product nuclear egress lamina protein 44851-44884 7:04:01 GGGAGGTGCGGCCGGGCCCGGGAGCTGGAGCTGG
44899 44942 44840 45979 CDS product nuclear egress lamina protein 44899-44942 7:04:01 CCCACGCCCGCGCGCGCCGGCGACCGGACCCCGACCCCGAGCCC
44943 44973 44840 45979 CDS product nuclear egress lamina protein 44943-44973 6:03:01 GGGCTCGGCCCGGTCCGAGCGGCGCGGGCGG
44984 45044 44840 45979 CDS product nuclear egress lamina protein 44984-45044 11:08:02 CCCCGCCCCGCTCCCGCTCCCGCTCCCGCTCCCGCTCCCGCTCCCGCTCGCCGTTCCTGCC
45048 45084 44840 45979 CDS product nuclear egress lamina protein 45048-45084 7:04:01 GGAGCGGTCCCGGTCCCGGTCCCGGTCCCGGTCCCGG
45056 45088 44840 45979 CDS product nuclear egress lamina protein 45056-45088 6:03:01

CCCGGTCCCGGTCCCGGTCCCGGTCCCGGTCCC
45141 45169 44840 45979 CDS product nuclear egress lamina protein 45141-45169 4:01:01
GGGCGCGCGGCGCGCGGGGCGCGCGCGGG
45195 45210 44840 45979 CDS product nuclear egress lamina protein 45195-45210 5:02:01 CCTGGCCTCCTCCCCC
45360 45386 44840 45979 CDS product nuclear egress lamina protein 45360-45386 5:02:01
CCCCGCCTGCACCGTGACCGGTGGCCC
45379 45412 44840 45979 CDS product nuclear egress lamina protein 45379-45412 6:03:01
GGTGGCCCGCGGATGGGCCGCGCGGACCGCGCGG
45459 45481 44840 45979 CDS product nuclear egress lamina protein 45459-45481 6:03:01
CCGGGCCTTCCTGGCCTCCATCC
45529 45548 44840 45979 CDS product nuclear egress lamina protein 45529-45548 5:02:01 GGCGGCGTCGGGCCCGGCGG
45569 45588 44840 45979 CDS product nuclear egress lamina protein 45569-45588 4:01:01 CCGAGGTCCTGGCCCAGCCC
45707 45739 44840 45979 CDS product nuclear egress lamina protein 45707-45739 8:05:02
CCATCCACCTGCACCACCGCCTGCTGGACCACC
45804 45827 44840 45979 CDS product nuclear egress lamina protein 45804-45827 6:03:01
GGTGCGCCGGGACGGGGGCGGGGG
45846 45877 44840 45979 CDS product nuclear egress lamina protein 45846-45877 6:03:01
GGACGCCGAGGTGCCGGCGGTGAGCGCGGGGG
46096 46134 45903 49655 CDS product DNA polymerase catalytic subunit 46096-46134 7:04:01
GGGCCTTGAAGGTGGCGCCGACGGTGCCCAGGAGGTGGG
46148 46170 45903 49655 CDS product DNA polymerase catalytic subunit 46148-46170 4:01:01
GGTGTTGAGGGGCACGGCGCGGG
46182 46208 45903 49655 CDS product DNA polymerase catalytic subunit 46182-46208 5:02:01

CCCGGGTCCTCGGCCATGTCCGAGACC
46213 46266 45903 49655 CDS product DNA polymerase catalytic subunit 46213-46266 11:08:02
GGCGCGCGGGGCGGGCGGCGGCCGCGGCGGCGCCGCTGGGAGGCGCGGGCGGGG
46421 46442 45903 49655 CDS product DNA polymerase catalytic subunit 46421-46442 5:02:01
GGCGTAGGCCTCGGGCGGGCGG
46485 46521 45903 49655 CDS product DNA polymerase catalytic subunit 46485-46521 6:03:01
GGGTCGGCGATCTTGGCGTGGGCCTCGGCCAGCACGG
46553 46570 45903 49655 CDS product DNA polymerase catalytic subunit 46553-46570 4:01:01
CCGCCCGGGCCACTCGCC
46571 46599 45903 49655 CDS product DNA polymerase catalytic subunit 46571-46599 6:03:01
GGGCGGCACGGCTGAGGCCTCGGCCGCGG
46810 46841 45903 49655 CDS product DNA polymerase catalytic subunit 46810-46841 7:04:01
CCGCCGAGACCTGCCGCGCCATGCCGTCGCCC
46888 46910 45903 49655 CDS product DNA polymerase catalytic subunit 46888-46910 4:01:01
CCGAGTCCGTGTCGCCGTAGACC
46932 46958 45903 49655 CDS product DNA polymerase catalytic subunit 46932-46958 5:02:01
CCGGCCGCCGCAGCGGCCTCGGCGGCC
46946 46964 45903 49655 CDS product DNA polymerase catalytic subunit 46946-46964 4:01:01
GGCCTCGGCGGCCTCGGGG
47045 47077 45903 49655 CDS product DNA polymerase catalytic subunit 47045-47077 6:03:01
GGTCGTCACGGTGGCCGCGACGGCCAGGCAGGG
47085 47114 45903 49655 CDS product DNA polymerase catalytic subunit 47085-47114 5:02:01
CCGTTGGCCACGCCGGTGAAGCCGTACACC

47162 47186 45903 49655 CDS product DNA polymerase catalytic subunit 47162-47186 5:02:01
GGCCTCCTCGGGCGCGGCGGCGGGG
47311 47347 45903 49655 CDS product DNA polymerase catalytic subunit 47311-47347 7:04:01
CCGCGCCGGGCGCCAGCCCCGCCGGCGGGGCCTCGCC
47455 47479 45903 49655 CDS product DNA polymerase catalytic subunit 47455-47479 4:01:01
CCAGGACCTTGGCGCCCTGATACCC
47512 47620 45903 49655 CDS product DNA polymerase catalytic subunit 47512-47620 23:20:05
CCGCCGCCCCCGCGCTCCCTGCGTCCTCCCCGTCCCCCCCCTCCGCCTCCCCGTCATCCTCCCCATCGCCCCCCTCCTCGCCCTCGCCGCGGGCCGCGTCGGCCCAGCC
47602 47630 45903 49655 CDS product DNA polymerase catalytic subunit 47602-47630 4:01:01
GGGCCGCGTCGGCCCAGCCGGCGAACGGG
47631 47654 45903 49655 CDS product DNA polymerase catalytic subunit 47631-47654 4:01:01
CCCCCGTCGCTGCCCTCGGCGCCC
47648 47684 45903 49655 CDS product DNA polymerase catalytic subunit 47648-47684 5:02:01
GGCGCCCGCGGGGCGCCGCTGGCTGTCCGGCAGCAGG
47698 47718 45903 49655 CDS product DNA polymerase catalytic subunit 47698-47718 4:01:01
GGGCCAGGCGCAGCAGGCAGG
47768 47798 45903 49655 CDS product DNA polymerase catalytic subunit 47768-47798 7:04:01
CCCGGCCAGCCGCGCCACCGCCGACAGCTCC
47828 47846 45903 49655 CDS product DNA polymerase catalytic subunit 47828-47846 4:01:01
CCCGACCAGCGCCGAGTCC
47862 47891 45903 49655 CDS product DNA polymerase catalytic subunit 47862-47891 6:03:01
CCGATGACGCCGCGGCCCTCCGGCCCGGCC
47911 47957 45903 49655 CDS product DNA polymerase catalytic subunit 47911-47957 9:06:02

CCTTGTAGTCCAGGTCCACCTTCCGCTCCCGCAGGGCCTCCTCGGCC

48278 48300 45903 49655 CDS product DNA polymerase catalytic subunit 48278-48300 5:02:01

GGCGGGCGCGGGGAGCCCGGCGG

48316 48339 45903 49655 CDS product DNA polymerase catalytic subunit 48316-48339 6:03:01

GGAAGGCCGGCGGCAGGTCGCAGG

48409 48443 45903 49655 CDS product DNA polymerase catalytic subunit 48409-48443 6:03:01

GGACGACGAGGTCCTCGGCGTTGGCGGCCGCCGGG

48436 48461 45903 49655 CDS product DNA polymerase catalytic subunit 48436-48461 5:02:01

CCGCCGGGAACGCCAGCCCGTCGCCC

48621 48652 45903 49655 CDS product DNA polymerase catalytic subunit 48621-48652 6:03:01

CCAGCGCGCCCGGGCCGCAGCCGGTACCAGCC

48823 48839 45903 49655 CDS product DNA polymerase catalytic subunit 48823-48839 4:01:01

CCGCCGCGCTAACCACC

48870 48891 45903 49655 CDS product DNA polymerase catalytic subunit 48870-48891 4:01:01

CCCAGGTAGCCGACGCCGGGCC

48900 48923 45903 49655 CDS product DNA polymerase catalytic subunit 48900-48923 7:04:01

CCGCCGCCCCCGCCGCCGGCGTCC

48932 48954 45903 49655 CDS product DNA polymerase catalytic subunit 48932-48954 5:02:01

GGCGCCGGCGGCCAGGGCCGAGG

48964 48992 45903 49655 CDS product DNA polymerase catalytic subunit 48964-48992 6:03:01

CCGCCACCATGGCGTCCACCAGCTGGCCC

49010 49026 45903 49655 CDS product DNA polymerase catalytic subunit 49010-49026 4:01:01

GGCCAGGTCAACGGCGG

49083 49100 45903 49655 CDS product DNA polymerase catalytic subunit 49083-49100 4:01:01
CCGTCCTCCGACATGCCC
49114 49136 45903 49655 CDS product DNA polymerase catalytic subunit 49114-49136 5:02:01
CCGTGGCGCCGCCGGGCCGGGCC
49118 49152 45903 49655 CDS product DNA polymerase catalytic subunit 49118-49152 6:03:01
GGCGCCGCCGGGCCGGGCCTCGGCGGCGAAGCGGG
49387 49415 45903 49655 CDS product DNA polymerase catalytic subunit 49387-49415 8:05:02
CCGCCAGCCTTGCAGCCTCCCCCTCCCCC
49471 49488 45903 49655 CDS product DNA polymerase catalytic subunit 49471-49488 5:02:01
GGAAGGAGGACACGGCGG
49515 49559 45903 49655 CDS product DNA polymerase catalytic subunit 49515-49559 8:05:02
CCGGCGCGGCCCGCCTCGCCGGCGCCGCCGGCGTCGCCCCGGCCC
49555 49575 45903 49655 CDS product DNA polymerase catalytic subunit 49555-49575 4:01:01
GGCCCGGCTGCGCGGCCGGGG
50121 50141 49926 53552 CDS product single-stranded DNA-binding protein 50121-50141 5:02:01
CCGGCACCAAGACCACCGGCC
50138 50162 49926 53552 CDS product single-stranded DNA-binding protein 50138-50162 5:02:01
GGCCTGGGCGGCGCGGGGCTGACGG
50174 50190 49926 53552 CDS product single-stranded DNA-binding protein 50174-50190 4:01:01
CCCAGCCACTACCACCC
50221 50249 49926 53552 CDS product single-stranded DNA-binding protein 50221-50249 5:02:01
CCTGCGCGCCAGCACGGCCGCGCCGAACC
50269 50292 49926 53552 CDS product single-stranded DNA-binding protein 50269-50292 5:02:01

GGCGCGGCGGCGCTTCGGCTTCGG

50368 50407 49926 53552 CDS product single-stranded DNA-binding protein 50368-50407 5:02:01

CCCCGAGACGGCCCTGCTCTTCCTCGCGACCACGGAGGCC

50401 50420 49926 53552 CDS product single-stranded DNA-binding protein 50401-50420 4:01:01

GGAGGCCTTCAAGGAGACGG

50650 50666 49926 53552 CDS product single-stranded DNA-binding protein 50650-50666 4:01:01

GGGCCCGGCCGCGGTGG

50686 50705 49926 53552 CDS product single-stranded DNA-binding protein 50686-50705 4:01:01

GGACGCGGTGGCGCGCGGGG

50795 50815 49926 53552 CDS product single-stranded DNA-binding protein 50795-50815 6:03:01

GGCGGCGGCGGTGGACGCGGG

50830 50849 49926 53552 CDS product single-stranded DNA-binding protein 50830-50849 4:01:01

GGACCAGGGCGCGGCCAAGG

50844 50860 49926 53552 CDS product single-stranded DNA-binding protein 50844-50860 4:01:01

CCAAGGCCGCCGCGCCC

50877 50906 49926 53552 CDS product single-stranded DNA-binding protein 50877-50906 6:03:01

GGCTGGCCTCGGTGATGGCGGCCGACACGG

50986 51024 49926 53552 CDS product single-stranded DNA-binding protein 50986-51024 7:04:01

GGGCGCGGCGGACGAGGCGGCGAAGCTGGACGCGCTGGG

51047 51065 49926 53552 CDS product single-stranded DNA-binding protein 51047-51065 4:01:01

GGGCTGGTGGGCGCCATGG

51097 51119 49926 53552 CDS product single-stranded DNA-binding protein 51097-51119 6:03:01

GGAGGTGGACGACGGCGGCGCGG

51138 51195 49926 53552 CDS product single-stranded DNA-binding protein 51138-51195 11:08:02
CCGCCGCCGGCTTCCACCGCTTCTACCAAATCGCCGCCCCCTACGTGGCCGGGAACCC
51237 51253 49926 53552 CDS product single-stranded DNA-binding protein 51237-51253 4:01:01
CCGGCCCGGCCGCGTCC
51283 51307 49926 53552 CDS product single-stranded DNA-binding protein 51283-51307 4:01:01
GGACCACCTGGCGATGGCGTGCGGG
51489 51516 49926 53552 CDS product single-stranded DNA-binding protein 51489-51516 5:02:01
GGCTGCGGCACCGGCTGCCCCGGTTCGG
51657 51673 49926 53552 CDS product single-stranded DNA-binding protein 51657-51673 4:01:01
CCACGTACCGCGCCGCC
51763 51797 49926 53552 CDS product single-stranded DNA-binding protein 51763-51797 7:04:01
CCACGCCAGCTTCCGCGGCGCCCTGGCCGCCATCC
51957 51989 49926 53552 CDS product single-stranded DNA-binding protein 51957-51989 6:03:01
GGCGCTCGGTGCTGGCCGTGGTGCAGGACTTGG
52138 52154 49926 53552 CDS product single-stranded DNA-binding protein 52138-52154 4:01:01
GGAGGCCGCGGTCGCGG
52219 52241 49926 53552 CDS product single-stranded DNA-binding protein 52219-52241 4:01:01
GGTGAGCCTGGAGGTCTTCCGGG
52430 52457 49926 53552 CDS product single-stranded DNA-binding protein 52430-52457 6:03:01
CCGCCCGGGAGCCCGACGCCTAACCCCC
52673 52705 49926 53552 CDS product single-stranded DNA-binding protein 52673-52705 6:03:01
GGGCTCACGGCGATCGTGGCCGGGGCGCGGCGG
52760 52780 49926 53552 CDS product single-stranded DNA-binding protein 52760-52780 4:01:01

CCCGACGCCTCGGCGGCCGCC
52771 52787 49926 53552 CDS product single-stranded DNA-binding protein 52771-52787 4:01:01
GGCGGCCGCCGAGGAGG
52798 52827 49926 53552 CDS product single-stranded DNA-binding protein 52798-52827 6:03:01
GGCCGAGGCCCGCCCGGAGGTGTGGGCGGG
52830 52847 49926 53552 CDS product single-stranded DNA-binding protein 52830-52847 5:02:01
CCTTCGCCGCCAGCCACC
52858 52890 49926 53552 CDS product single-stranded DNA-binding protein 52858-52890 6:03:01
GGTCATGGCCACGCGGCCCGCGGTGGTGCTGGG
52942 52971 49926 53552 CDS product single-stranded DNA-binding protein 52942-52971 6:03:01
GGCGGGCAACTGGAGCGGCCTCAACGGGGG
53053 53070 49926 53552 CDS product single-stranded DNA-binding protein 53053-53070 4:01:01
GGCCGCGGGCGCGGGCGG
53120 53147 49926 53552 CDS product single-stranded DNA-binding protein 53120-53147 4:01:01
GGCAGCAACGGCGCGCTGGCGCAGACGG
53188 53228 49926 53552 CDS product single-stranded DNA-binding protein 53188-53228 8:05:02
GGAGCACATGGACCTGGACGACTGGGCGGCCCTGGTGGAGG
53251 53309 49926 53552 CDS product single-stranded DNA-binding protein 53251-53309 11:08:02
GGTGGAGCTGACGGAGCGCGTGGCTGCGCGGCCCGGGGGCTGGTCGCCGGAGGGCGCGG
53329 53351 49926 53552 CDS product single-stranded DNA-binding protein 53329-53351 5:02:01
GGAGCTCGAGGCGGCGGCCGAGG
53412 53486 49926 53552 CDS product single-stranded DNA-binding protein 53412-53486 13:10:03
CCTACGCCTTCGGCGCCGCCGGGGCCGGGGCCGCCGGCGGCACCTGCGCCATGCCGGGGGCCAAGCGCCCCGACC

53423 53451 49926 53552 CDS product single-stranded DNA-binding protein 53423-53451 5:02:01
GGCGCCGCCGGGGCCGGGGCCGCCGGCGG
53503 53524 49926 53552 CDS product single-stranded DNA-binding protein 53503-53524 4:01:01
GGCGGCCGTTCCGGAAAAGCGG
53808 53835 53789 56239 CDS product DNA packaging terminase subunit 2 53808-53835 4:01:01
CCCCGTCGGTCCCGAGCCCGGCGGCCCC
53850 53893 53789 56239 CDS product DNA packaging terminase subunit 2 53850-53893 8:05:02
CCCGGCGTCCGGCCCGGCCCAGCCCAGCGGGGCCGCGGCCCGCC
53979 53994 53789 56239 CDS product DNA packaging terminase subunit 2 53979-53994 4:01:01 CCGCCACCTCGACGCC
54141 54161 53789 56239 CDS product DNA packaging terminase subunit 2 54141-54161 4:01:01
GGCGGCCGCGGGCGCCGCGGG
54321 54360 53789 56239 CDS product DNA packaging terminase subunit 2 54321-54360 6:03:01
CCTGCTCGCCTCGGCCGAGGCCGCGCTCGCCGCGCTCGCC
54380 54406 53789 56239 CDS product DNA packaging terminase subunit 2 54380-54406 7:04:01
GGGCCACGGTGGCGGCGGGCGGCGTGG
54570 54586 53789 56239 CDS product DNA packaging terminase subunit 2 54570-54586 4:01:01
CCAGTACCTGCCGCACC
54608 54673 53789 56239 CDS product DNA packaging terminase subunit 2 54608-54673 14:11:03
GGCTGGCGGCGGCGGCGGCGGCGCTGGAGGCGCTGGAAGCGCCGGCCGCCGCGGGGGCGCGCAGGG
54687 54739 53789 56239 CDS product DNA packaging terminase subunit 2 54687-54739 10:07:02
GGCGAGCGGCCCTCCCGGGGGCGCCGGCGACGGCGGCTCGGTGCGCGAGGCGG
54762 54782 53789 56239 CDS product DNA packaging terminase subunit 2 54762-54782 4:01:01
CCACGTCTTCCGCCCGGCGCC

54845 54867 53789 56239 CDS product DNA packaging terminase subunit 2 54845-54867 6:03:01
CCACCGCCAGCGCCGTCGCCGCC
54899 54940 53789 56239 CDS product DNA packaging terminase subunit 2 54899-54940 9:06:02
GGGAGGCGCGGCACCGGCTGCAGGTGGCCGCGGCGGAGCTGG
54988 55012 53789 56239 CDS product DNA packaging terminase subunit 2 54988-55012 6:03:01
GGGCTGGCGCGGCTGGACGGCGTGG
55026 55042 53789 56239 CDS product DNA packaging terminase subunit 2 55026-55042 4:01:01
GGGGAGCGCGGCGGTGG
55166 55229 53789 56239 CDS product DNA packaging terminase subunit 2 55166-55229 12:09:03
GGCGCGCGGCCGCGGGCGAGGAGGGGGGCGAGGATGGGGCCGACTCGGGCGAAGAGGGGGGTGG
55243 55357 53789 56239 CDS product DNA packaging terminase subunit 2 55243-55357 25:22:06
GGCGGGGACGGGGACGGGGCCGGGGGCGGCAACGGCGGCGCGGGGCCGGCCGGGGGCGCGGACGGCGGGGCCGGCGCGGGGGACGACGGCCCCGCGGGCTGGGCGGCGCTGGCGG
55548 55563 53789 56239 CDS product DNA packaging terminase subunit 2 55548-55563 4:01:01 CCGCGCCTTCCGCGCC
55573 55591 53789 56239 CDS product DNA packaging terminase subunit 2 55573-55591 5:02:01
GGCGCGGGCACGGTGGTGG
55596 55614 53789 56239 CDS product DNA packaging terminase subunit 2 55596-55614 4:01:01
CCGCGCCTCGGACGCCACC
55836 55861 53789 56239 CDS product DNA packaging terminase subunit 2 55836-55861 7:04:01
GGCGGCCGCAGGGGGCGGGTGGGTGG
56019 56049 53789 56239 CDS product DNA packaging terminase subunit 2 56019-56049 6:03:01
GGACCGAACGGCGCCGGGCCCGGACGGGCGG
56123 56146 53789 56239 CDS product DNA packaging terminase subunit 2 56123-56146 5:02:01
GGGCGCCGGATACGGTCGGCGAGG

56208 56221 53789 56239 CDS product DNA packaging terminase subunit 2 56208-56221 4:01:01 GGCGCCGGCGGCGG
56298 56317 56092 58935 CDS product envelope glycoprotein B 56298-56317 4:01:01 CCGACGACGCCGCCTGTGCC
56319 56346 56092 58935 CDS product envelope glycoprotein B 56319-56346 6:03:01 GGGGGAGCTGGGGAGGCCGGGAGCCCGG
56335 56399 56092 58935 CDS product envelope glycoprotein B 56335-56399 12:09:03
CCGGGAGCCCGGCGCCCCCAGCGAGCCCGGCGCCCCCCGCAAGCCCCGCCCCGCCGACGGCCCC
56492 56510 56092 58935 CDS product envelope glycoprotein B 56492-56510 4:01:01 CCCGCCGCCCTCGGGCGCC
56504 56522 56092 58935 CDS product envelope glycoprotein B 56504-56522 4:01:01 GGGCGCCACGGTGGTCCGG
56793 56808 56092 58935 CDS product envelope glycoprotein B 56793-56808 4:01:01 GGGCGCAAGGTGGTGG
56894 56927 56092 58935 CDS product envelope glycoprotein B 56894-56927 6:03:01
GGACGAGGTGTACACGGCGCTGGGCTCGGCGGGG
56963 56985 56092 58935 CDS product envelope glycoprotein B 56963-56985 6:03:01 GGAGGAGGTGGAGGCGCGCTCGG
57197 57213 56092 58935 CDS product envelope glycoprotein B 57197-57213 4:01:01 GGTGGCCTGGGACTGGG
57389 57405 56092 58935 CDS product envelope glycoprotein B 57389-57405 4:01:01 GGAGGCCGGGGCCGCGG
57601 57631 56092 58935 CDS product envelope glycoprotein B 57601-57631 6:03:01
CCGCCGCTGCCGCGGCGCCCAAGCCGGTACC
57644 57680 56092 58935 CDS product envelope glycoprotein B 57644-57680 9:06:02
CCGGTCCGCGTCCCCCACGCCCCCCGCGCCCGCCGCC
57684 57700 56092 58935 CDS product envelope glycoprotein B 57684-57700 4:01:01 GGCGACGGCGGCGACGG
57760 57780 56092 58935 CDS product envelope glycoprotein B 57760-57780 4:01:01 CCTACGACCACATCCAGGACC
57800 57822 56092 58935 CDS product envelope glycoprotein B 57800-57822 5:02:01 CCGCCTGGCCACGTCCTGGTGCC
57902 57914 56092 58935 CDS product envelope glycoprotein B 57902-57914 4:01:01 CCGCCGCGCCGCC
58022 58043 56092 58935 CDS product envelope glycoprotein B 58022-58043 5:02:01 CCGCCCGCCGGTCTCCTTCGCC
58064 58086 56092 58935 CDS product envelope glycoprotein B 58064-58086 5:02:01 GGTGGAGGGCCAGCTCGGCGAGG

58244 58282 56092 58935 CDS product envelope glycoprotein B 58244-58282 6:03:01
CCTGAACCTCACGGTCCTAGAGGACCGCGAGTTCCTGCC
58394 58416 56092 58935 CDS product envelope glycoprotein B 58394-58416 4:01:01 GGTCAAGACGGACGGCAACATGG
58448 58501 56092 58935 CDS product envelope glycoprotein B 58448-58501 8:05:02
GGGCCTGGGCGCCGTCGGGCAGGCAGTGGGCACGGTTGTGCTGGGCGCCGCGGG
58554 58578 56092 58935 CDS product envelope glycoprotein B 58554-58578 4:01:01 GGCGCGCTGGCCACGGGGCTGCTGG
58597 58614 56092 58935 CDS product envelope glycoprotein B 58597-58614 4:01:01 CCGCCTTCCTGGCGTACC
58624 58643 56092 58935 CDS product envelope glycoprotein B 58624-58643 4:01:01 CCCGCCTCCGCAGCAACCCC
58694 58728 56092 58935 CDS product envelope glycoprotein B 58694-58728 8:05:02
GGGCGCGGGCGCCCCGGGCGAGGAGGGGGAGGAGG
58876 58893 56092 58935 CDS product envelope glycoprotein B 58876-58893 4:01:01 GGCGGCGGGCGCCGCCGG
58887 58916 56092 58935 CDS product envelope glycoprotein B 58887-58916 5:02:01
CCGCCGGCGTACCAGCAGCTCCCGATGTCC
59545 59651 59444 60391 CDS product capsid scaffold protein 59545-59651 21:18:05
GGCAATGGCCCCGGGCTCCTCAGGTGGCCCGCCGGCAGCGGCGGGCGCCGGGGCGGCGGGGGTGGCGAGCGGTGGGGCCGGGGCCGGGGCCTGGGCCGGCGCGGGGG
59622 59641 59444 60391 CDS product capsid scaffold protein 59622-59641 4:01:01 CCGGGGCCGGGGCCTGGGCC
59661 59680 59444 60391 CDS product capsid scaffold protein 59661-59680 5:02:01 GGGCCGGCGCGGGGGCCGGG
59676 59692 59444 60391 CDS product capsid scaffold protein 59676-59692 4:01:01 CCGGGCCCGCGACCACC
59693 59708 59444 60391 CDS product capsid scaffold protein 59693-59708 4:01:01 GGCTGGGGCGGAGCGG
59738 59770 59444 60391 CDS product capsid scaffold protein 59738-59770 8:05:02
GGCAGGCACGGCACGGGCGCTGGGGGCAGGTGG
59779 59810 59444 60391 CDS product capsid scaffold protein 59779-59810 5:02:01
GGCCGCGAAGGCGGGGCCGTAGGGGTAGCCGG

59863 59931 59444 60391 CDS product capsid scaffold protein 59863-59931 14:11:03
GGAGAGGGCGTGTGGAGCCGGCTGTGGCGGCGAGGGGGGGCGGTGGTGCTGGGGCGGCAGCTCGGCGGG

59955 59975 59444 60391 CDS product capsid scaffold protein 59955-59975 4:01:01 CCTCGTCGTCCCCGCCGCCCC

60013 60037 59444 60391 CDS product capsid scaffold protein 60013-60037 7:04:01 GGCTGGGGGGCGGCGGTCGAGGGGG

60038 60070 59444 60391 CDS product capsid scaffold protein 60038-60070 6:03:01
CCGTCGTAGCCCGGCCCGTCCGCGCCCTGCGCC

60075 60107 59444 60391 CDS product capsid scaffold protein 60075-60107 6:03:01
GGCGCCGGCCGTCGGCGATGGCGCCGGCCAGGG

60151 60185 59444 60391 CDS product capsid scaffold protein 60151-60185 7:04:01
GGGGTGCCAGGGCGCGGCGGCGCCACTGGCGGCGG

60200 60266 59444 60391 CDS product capsid scaffold protein 60200-60266 16:13:04
GGCATCGGGGGCGGGGGCGCGGCGGCGATGGCGGGCGGCAGGAAGTACGGGGCGGGCGGGGGCGCGG

60340 60360 59444 60391 CDS product capsid scaffold protein 60340-60360 5:02:01 GGCCGGCAGGGGGTGCGCCGG

60391 60424 59444 61303 CDS product capsid maturation protease 60391-60424 8:05:02
GGCGGGGGGCGGCGCCGGCGGAAGCTCCGGCTGG

60507 60520 59444 61303 CDS product capsid maturation protease 60507-60520 4:01:01 CCTCCCCCCCGGCC

60665 60692 59444 61303 CDS product capsid maturation protease 60665-60692 5:02:01
GGCACCGGCCAGGCGCGGTCGCCGAGGG

60703 60728 59444 61303 CDS product capsid maturation protease 60703-60728 6:03:01
GGCCTCGGCGTCGGCGAGGAGGGCGG

60750 60782 59444 61303 CDS product capsid maturation protease 60750-60782 7:04:01
GGCGGAAGGGGGCCACACAGGCTTCGGGCGTGG

60851 60874 59444 61303 CDS product capsid maturation protease 60851-60874 5:02:01 CCGTCGGCCTCCTCGCCGGGCGCC

60868 60887 59444 61303 CDS product capsid maturation protease 60868-60887 4:01:01 GGGCGCCAGGCGGCGCGAGG
61111 61131 59444 61303 CDS product capsid maturation protease 61111-61131 5:02:01 GGGCAGCGGGGCGGCGGGCGG
61268 61284 59444 61303 CDS product capsid maturation protease 61268-61284 4:01:01 CCGGCGCCGCCGTCGCC
61586 61599 61408 63219 CDS product DNA packaging tegument protein UL25 61586-61599 4:01:01 CCGCCACGACCTCC
61605 61629 61408 63219 CDS product DNA packaging tegument protein UL25 61605-61629 6:03:01 GGGCGCCGGCGCGGCGCGGTGGCGG
61858 61904 61408 63219 CDS product DNA packaging tegument protein UL25 61858-61904 8:05:02 CCCACGGCGCCGTCGGGGCCGCCGTCGGCGCCGCGCGCCACCTCCCC
61913 61934 61408 63219 CDS product DNA packaging tegument protein UL25 61913-61934 4:01:01 CCGCGCCGGGCACCAGCGTGCC
62039 62064 61408 63219 CDS product DNA packaging tegument protein UL25 62039-62064 5:02:01 GGCTCTGGTCCTCGGCGAGGAAGAGG
62153 62174 61408 63219 CDS product DNA packaging tegument protein UL25 62153-62174 5:02:01 CCGCGCCGCCGCCCAGCGCCCC
62175 62190 61408 63219 CDS product DNA packaging tegument protein UL25 62175-62190 4:01:01 GGGGATGGCCGGCAGG
62254 62274 61408 63219 CDS product DNA packaging tegument protein UL25 62254-62274 5:02:01 CCCGCGCCGCCGCCCGGCGCC
62269 62295 61408 63219 CDS product DNA packaging tegument protein UL25 62269-62295 5:02:01 GGCGCCTGGAACTGGCCCCGTGGGAGG
62427 62451 61408 63219 CDS product DNA packaging tegument protein UL25 62427-62451 5:02:01 GGTGGCGCGGTGCGCGAGGTGCAGG
62522 62538 61408 63219 CDS product DNA packaging tegument protein UL25 62522-62538 5:02:01 CCGCCACCGCGGCCGCC

62650 62666 61408 63219 CDS product DNA packaging tegument protein UL25 62650-62666 5:02:01
CCCGCCGAGCCCCCGCC
62688 62713 61408 63219 CDS product DNA packaging tegument protein UL25 62688-62713 5:02:01
GGCCAGCAGGTCGGCCGCCAGGGTGG
62782 62831 61408 63219 CDS product DNA packaging tegument protein UL25 62782-62831 9:06:02
CCGAGCGCGCCCGCGCCGCAGTCCCCGTCCTCGCCCTCCCCCGCGCGGCC
62840 62904 61408 63219 CDS product DNA packaging tegument protein UL25 62840-62904 17:14:04
CCGCCGACTCCGCCGCCGCCGCCGCGTCCGCCTCCTCCGCCCGGCGCGCCGCCTCCTCCAGGTCC
62918 62937 61408 63219 CDS product DNA packaging tegument protein UL25 62918-62937 5:02:01
CCACCTCCGCCACGCGGGCC
63002 63034 61408 63219 CDS product DNA packaging tegument protein UL25 63002-63034 6:03:01
CCGCGGCCGCTGCCGCGGCCCGGTTCCGCGCCC
63207 63235 63201 64040 CDS product nuclear protein UL24 63207-63235 5:02:01
CCCAAGCGCCATGGCCCCGGCGCCGCCCC
63244 63271 63201 64040 CDS product nuclear protein UL24 63244-63271 5:02:01 CCGAGATCCCGCCCAGGCACGCCGCGCC
63282 63296 63201 64040 CDS product nuclear protein UL24 63282-63296 5:02:01 CCCCCACCCCCCGCC
63310 63332 63201 64040 CDS product nuclear protein UL24 63310-63332 5:02:01 CCTCGGCCCTCCCGCCCGCGGCC
63339 63376 63201 64040 CDS product nuclear protein UL24 63339-63376 9:06:02
GGGGACTGGGATGGGGACGGGGGCGGGGGCGGACGCGG
63377 63398 63201 64040 CDS product nuclear protein UL24 63377-63398 6:03:01 CCGCCCCTTCCGCCGCCGCGCC
63402 63423 63201 64040 CDS product nuclear protein UL24 63402-63423 4:01:01 GGTCTCGGTCTCGGTCTCCCGG
63449 63462 63201 64040 CDS product nuclear protein UL24 63449-63462 4:01:01 CCGCCCCGCCCGCC
63601 63635 63201 64040 CDS product nuclear protein UL24 63601-63635 7:04:01

CCAGCACCGGCGCCAGGACCACCGGGTCCGGGCCC

63609 63647 63201 64040 CDS product nuclear protein UL24 63609-63647 7:04:01

GGCGCCAGGACCACCGGGTCCGGGCCCGGGGGGGCGAGG

63880 63909 63201 64040 CDS product nuclear protein UL24 63880-63909 5:02:01

CCTCCGGGGCGCCGAACTCGCCCAAGAGCC

63910 63928 63201 64040 CDS product nuclear protein UL24 63910-63928 4:01:01 GGGCTAGGCGCGGGCGCGG

63984 64001 63201 64040 CDS product nuclear protein UL24 63984-64001 4:01:01 CCGTGGCACCGGACCCCC

64091 64112 64039 65109 CDS product thymidine kinase 64091-64112 4:01:01 GGACGGCGCGCACGGGCTGGGG

64141 64154 64039 65109 CDS product thymidine kinase 64141-64154 5:02:01 CCGCCGCCTCCGCC

64235 64266 64039 65109 CDS product thymidine kinase 64235-64266 6:03:01 CCTCGCCGCGTCCGCGCGCCGCGCCGCGGCCC

64262 64302 64039 65109 CDS product thymidine kinase 64262-64302 8:05:02

GGCCCAGGGGGGGCGCGCGGACGCGGACGCGGCCGGCCTGG

64374 64390 64039 65109 CDS product thymidine kinase 64374-64390 4:01:01 CCGCCTGAGCCGGCGCC

64424 64476 64039 65109 CDS product thymidine kinase 64424-64476 10:07:02

CCGGCACCCCCTGGCCGCCTGCCTGTGCTACCCCTTCGCCCGCTACTGCCTCC

64558 64571 64039 65109 CDS product thymidine kinase 64558-64571 4:01:01 CCCTCCCCCCGGCC

64603 64635 64039 65109 CDS product thymidine kinase 64603-64635 5:02:01

GGCCCGGGGACCGCGCGGACGCGGGCTTTCTGG

64686 64749 64039 65109 CDS product thymidine kinase 64686-64749 11:08:02

GGCGCGGGGGGCGCGTGGCGCGACGGCTGGGACGCGCTGGGGTGGGCGGACGCGGACGCGCTGG

64851 64863 64039 65109 CDS product thymidine kinase 64851-64863 4:01:01 GGCGGCGGGGCGG

64865 64883 64039 65109 CDS product thymidine kinase 64865-64883 4:01:01 CCTGCCCGCGGTCCACGCC

64897 64920 64039 65109 CDS product thymidine kinase 64897-64920 5:02:01 CCCTGGCCGACCGCCTTGCGGCCC

| | | | | | | | |
|---|---|---|---|---|---|---|---|
| 64989 | 65007 | 64039 | 65109 | CDS product thymidine kinase | 64989-65007 | 4:01:01 | CCCGCCATGCGGGCCGCCC |
| 65012 | 65026 | 64039 | 65109 | CDS product thymidine kinase | 65012-65026 | 4:01:01 | GGACGGGGCGGCGGG |
| 65036 | 65064 | 64039 | 65109 | CDS product thymidine kinase | 65036-65064 | 5:02:01 | GGCGGACCTGGCGCGCCGGTTCGCGCGGG |
| 65073 | 65096 | 64039 | 65109 | CDS product thymidine kinase | 65073-65096 | 4:01:01 | CCGGGGGGGCCCGAGGCGGCCCCC |
| 65075 | 65101 | 64039 | 65109 | CDS product thymidine kinase | 65075-65101 | 5:02:01 | GGGGGGGCCCGAGGCGGCCCCCAGGGG |
| 65277 | 65302 | 65226 | 67772 | CDS product envelope glycoprotein H | 65277-65302 | 5:02:01 | CCGCGCTCGCCGCGGGCGCCCCCGCC |
| 65326 | 65387 | 65226 | 67772 | CDS product envelope glycoprotein H | 65326-65387 | 12:09:03 | GGGCGGGGACGCGCGGTACGAGGTCGAGGAGTGGGAGATGGTGGTCGGGGGCGAGTCGGCGG |
| 65400 | 65418 | 65226 | 67772 | CDS product envelope glycoprotein H | 65400-65418 | 4:01:01 | CCATCCACTGCCTTGGGCC |
| 65563 | 65588 | 65226 | 67772 | CDS product envelope glycoprotein H | 65563-65588 | 4:01:01 | GGACGCCGGGATCCCGGACACCCCGG |
| 65668 | 65686 | 65226 | 67772 | CDS product envelope glycoprotein H | 65668-65686 | 4:01:01 | CCTGGTGCCGAGCCGGCCC |
| 65758 | 65790 | 65226 | 67772 | CDS product envelope glycoprotein H | 65758-65790 | 6:03:01 | CCTGCCGCGCGCTCCGCGCCCCGCGCCGACGCC |
| 65830 | 65843 | 65226 | 67772 | CDS product envelope glycoprotein H | 65830-65843 | 4:01:01 | GGCCGGCGGCGCGG |
| 65918 | 65947 | 65226 | 67772 | CDS product envelope glycoprotein H | 65918-65947 | 5:02:01 | CCCGTCTCCTACGTCCCCACGGGCCGGGCC |
| 65964 | 65994 | 65226 | 67772 | CDS product envelope glycoprotein H | 65964-65994 | 7:04:01 | GGGCCGCGGTCCGGCTGGAGGTGGGGCTCGG |
| 66106 | 66132 | 65226 | 67772 | CDS product envelope glycoprotein H | 66106-66132 | 6:03:01 | CCCGCCGCCCGGGCCCGCGCCCGGGCC |
| 66128 | 66143 | 65226 | 67772 | CDS product envelope glycoprotein H | 66128-66143 | 4:01:01 | GGGCCGCGGCGGCGGG |
| 66155 | 66176 | 65226 | 67772 | CDS product envelope glycoprotein H | 66155-66176 | 4:01:01 | GGCTACGGGAGCTCGCGGCTGG |
| 66247 | 66284 | 65226 | 67772 | CDS product envelope glycoprotein H | 66247-66284 | 7:04:01 | CCTGGCCGCCGCGCACCGGGAGGCCTTCCTGATGCTCC |

66285 66296 65226 67772 CDS product envelope glycoprotein H 66285-66296 4:01:01 GGGAGGCGGCGG
66304 66353 65226 67772 CDS product envelope glycoprotein H 66304-66353 9:06:02 CCGCGGGCCCGGGCCGGGCCCCGCGCCGGACGCCGCCTACCACGCGTACC
66354 66373 65226 67772 CDS product envelope glycoprotein H 66354-66373 4:01:01 GGGTCGCGGCGCGGCTGGGG
66655 66673 65226 67772 CDS product envelope glycoprotein H 66655-66673 4:01:01 CCCGGACGCCCCGCCCGCC
66678 66704 65226 67772 CDS product envelope glycoprotein H 66678-66704 6:03:01 GGCGGCTGGCCCACGGCGTGGTGTCGG
66728 66745 65226 67772 CDS product envelope glycoprotein H 66728-66745 4:01:01 GGCGAGCTGGCCTGGCGG
66738 66758 65226 67772 CDS product envelope glycoprotein H 66738-66758 4:01:01 CCTGGCGGCCGCCCGCGCGCC
66781 66815 65226 67772 CDS product envelope glycoprotein H 66781-66815 6:03:01 GGCGTCGGCGTTCCCGGCGGACGCGCAGGCGCTGG
66849 66890 65226 67772 CDS product envelope glycoprotein H 66849-66890 8:05:02 CCATGTGCACCGCCGGCCACGCCACGGCGGCCGCCCTGGACC
66874 66899 65226 67772 CDS product envelope glycoprotein H 66874-66899 6:03:01 GGCGGCCGCCCTGGACCTGGAGGAGG
66912 66931 65226 67772 CDS product envelope glycoprotein H 66912-66931 4:01:01 CCGGCGCGCCCGGGGCCGCC
66914 66938 65226 67772 CDS product envelope glycoprotein H 66914-66938 4:01:01 GGCGCGCCCGGGGCCGCCGGCGCGG
66975 66992 65226 67772 CDS product envelope glycoprotein H 66975-66992 4:01:01 CCTCATTCCGCCTGGACC
67041 67085 65226 67772 CDS product envelope glycoprotein H 67041-67085 8:05:02 GGGCCGCGCTGGACGCCTGGCTGGAGGTGCGGCCCGCGGCCGCGG
67073 67102 65226 67772 CDS product envelope glycoprotein H 67073-67102 5:02:01 CCCGCGGCCGCGGCGCCGAACCTCAGCGCC
67105 67130 65226 67772 CDS product envelope glycoprotein H 67105-67130 5:02:01 GGCGCTTGGCATGCTGGGCCGGGGGG
67190 67206 65226 67772 CDS product envelope glycoprotein H 67190-67206 4:01:01 GGCGGGTGGGGCGCGGG
67357 67376 65226 67772 CDS product envelope glycoprotein H 67357-67376 4:01:01 GGCCAGCGGCGCCGTGGAGG

67381 67401 65226 67772 CDS product envelope glycoprotein H 67381-67401 5:02:01 CCGCCTCCCGGTCCCCGGCCC
67543 67574 65226 67772 CDS product envelope glycoprotein H 67543-67574 7:04:01 CCCCCGCCTCCACAGCGGCCGCTCCCGCGCCC
67659 67689 65226 67772 CDS product envelope glycoprotein H 67659-67689 6:03:01 GGGCTTCGGTGGCCGGGGCGCTGGTCGCGGG
67970 68022 67966 69777 CDS product tegument protein UL21 67970-68022 13:10:03 CCCCGGCGCCGCCCCCCGCGCTCGCCTCCTCCTCCGCCTCCTCCTCTTCCTCC
68068 68086 67966 69777 CDS product tegument protein UL21 68068-68086 6:03:01 CCGGCCACCCCCAGCCGCC
68123 68146 67966 69777 CDS product tegument protein UL21 68123-68146 5:02:01 GGGCGGCGGGCGCGCGGTCGAAGG
68207 68240 67966 69777 CDS product tegument protein UL21 68207-68240 7:04:01 CCAGCGCCCGCGCCAGCTCCGGCAGCCCGCCGCC
68336 68359 67966 69777 CDS product tegument protein UL21 68336-68359 6:03:01 GGGAGGCCAGGCTCAGGACGGCGG
68416 68454 67966 69777 CDS product tegument protein UL21 68416-68454 9:06:02 CCGTCACCCCCGCTCCCGCCGCCACCGGCGCCCCGCGCC
68455 68491 67966 69777 CDS product tegument protein UL21 68455-68491 7:04:01 GGGGTCGGCGCGGGCGGCTCGCCGGGGTCGGCGCGGG
68573 68602 67966 69777 CDS product tegument protein UL21 68573-68602 6:03:01 GGGGCGCGGCGGCGCGGTGCGGGTAGTTGG
68728 68756 67966 69777 CDS product tegument protein UL21 68728-68756 4:01:01 CCAACGAAGCCGCGGCGCCCGCGCGGCCC
68900 68943 67966 69777 CDS product tegument protein UL21 68900-68943 7:04:01 GGGACGGGGGGGCCGCCACGGCCCAGGCGTTGTGGGTCCGCGGG
68973 68997 67966 69777 CDS product tegument protein UL21 68973-68997 5:02:01 GGCGGCTCCGCGGCGCCCCGGCGGG

69006 69021 67966 69777 CDS product tegument protein UL21 69006-69021 4:01:01 CCCGCCGGCGCCCGCC
69031 69060 67966 69777 CDS product tegument protein UL21 69031-69060 7:04:01 CCGGCGCCGCCGTCGTCCGCGCCGGCCCCC
69065 69091 67966 69777 CDS product tegument protein UL21 69065-69091 7:04:01 GGCGGACGCCGGCGGCGCGGGCGGCGG
69125 69162 67966 69777 CDS product tegument protein UL21 69125-69162 8:05:02 CCTCCCCCACGCCGTCGGCCCCGCTCCCGCGGCCCGCC
69197 69228 67966 69777 CDS product tegument protein UL21 69197-69228 7:04:01 GGGGGCGCGCGGGCGCCGGCGGCGCCGGCAGG
69443 69467 67966 69777 CDS product tegument protein UL21 69443-69467 5:02:01 CCGCGCCCGCGGCCGGGGCCCCGCC
69531 69565 67966 69777 CDS product tegument protein UL21 69531-69565 8:05:02 GGCCGCGCCGGCGGCGGGGGCGAGGGGGCCCGCGG
69650 69662 67966 69777 CDS product tegument protein UL21 69650-69662 4:01:01 CCTCCCCGCCCCC
69671 69687 67966 69777 CDS product tegument protein UL21 69671-69687 4:01:01 CCGAGACCACCGTGCCC
69938 70002 69889 70578 CDS product envelope protein UL20 69938-70002 17:14:04 GGGCGGCGCCGAGGCGGAGGAGGGGGCGGCCGGGGGCGGCGCCTCGGACGGCGAGGGGGAGGAGG
70031 70050 69889 70578 CDS product envelope protein UL20 70031-70050 4:01:01 GGCGTACGGGGGCGACGTGG
70109 70131 69889 70578 CDS product envelope protein UL20 70109-70131 4:01:01 GGCGTACGTGGTCTTTGGCGCGG
70249 70267 69889 70578 CDS product envelope protein UL20 70249-70267 5:02:01 GGCTGGCGGCGGCCGCGGG
70280 70298 69889 70578 CDS product envelope protein UL20 70280-70298 4:01:01 GGGCGTGCGGGCGGACCGG
70308 70401 69889 70578 CDS product envelope protein UL20 70308-70401 16:13:04 GGGCGCTGGCCGCGGGCGCTGCTGGCGGCGCTGGTCCTGGCGCGCGCGGCGGTCTTCGCGGCGGTGGCGCTGCCGGCGGCCTTCGCGGGCCCGG
70424 70441 69889 70578 CDS product envelope protein UL20 70424-70441 5:02:01 GGCGGCGGCCGACGGCGG
70451 70463 69889 70578 CDS product envelope protein UL20 70451-70463 4:01:01 GGCGGCGGGGGGG

70472 70497 69889 70578 CDS product envelope protein UL20 70472-70497 6:03:01 GGCCGGGCTGGCGGCGTACACGGCGG
70698 70720 70658 74833 CDS product major capsid protein 70698-70720 5:02:01 GGGCGGGCTGGTCTCCGGCCAGG
70808 70825 70658 74833 CDS product major capsid protein 70808-70825 4:01:01 CCGCCTTCGACGCCCTCC
70866 70885 70658 74833 CDS product major capsid protein 70866-70885 4:01:01 GGAGCTTGGGCTCTCGGTGG
70978 70998 70658 74833 CDS product major capsid protein 70978-70998 4:01:01 CCACACCCGGTGGACCAGCCC
71085 71119 70658 74833 CDS product major capsid protein 71085-71119 7:04:01 GGAGGAGGCGGCCGACGGGACGCAGGTCTCGTCGG
71283 71296 70658 74833 CDS product major capsid protein 71283-71296 4:01:01 GGCGGGACCGGCGG
71415 71437 70658 74833 CDS product major capsid protein 71415-71437 4:01:01 GGGCTGCACGGCCGCCTCGGTGG
71426 71449 70658 74833 CDS product major capsid protein 71426-71449 5:02:01 CCGCCTCGGTGGCCGTGCCGCGCC
71470 71507 70658 74833 CDS product major capsid protein 71470-71507 7:04:01 GGGCGCCCGGTGGACGGCGTGCTGGTGACGACGGCGGG
71519 71560 70658 74833 CDS product major capsid protein 71519-71560 8:05:02 GGCTGCTGGGCGGCGTGCTGGCGCTGGCCGACTCGGAGGCGG
71670 71707 70658 74833 CDS product major capsid protein 71670-71707 7:04:01 GGGGCTGGAGGGGACCGCGGGCCCGGGGCTGGCCGTGG
71745 71772 70658 74833 CDS product major capsid protein 71745-71772 6:03:01 GGCGGACCTGGTGGCCGTTGGGGACCGG
71926 71948 70658 74833 CDS product major capsid protein 71926-71948 5:02:01 CCGCCGCCCGGGCACCCCGACCC
72054 72076 70658 74833 CDS product major capsid protein 72054-72076 5:02:01 GGACGTGGACCCGGTGCTGGCGG
72101 72117 70658 74833 CDS product major capsid protein 72101-72117 4:01:01 GGCGAGAGGCGGAGTGG
72148 72177 70658 74833 CDS product major capsid protein 72148-72177 5:02:01 GGCGAGCAGGAGCTCGCGGACCAGGTGCGG
72249 72269 70658 74833 CDS product major capsid protein 72249-72269 4:01:01 GGAGCAGATGGTGGCGCCCGG

72318 72345 70658 74833 CDS product major capsid protein 72318-72345 4:01:01 GGGGCCCGCGGACGTGGAGCTGCCGGGG
72340 72359 70658 74833 CDS product major capsid protein 72340-72359 5:02:01 CCGGGGCCGCCCAACCCGCC
72489 72512 70658 74833 CDS product major capsid protein 72489-72512 6:03:01 GGCGACGGTGGCGGCGGTGCGCGG
72720 72751 70658 74833 CDS product major capsid protein 72720-72751 5:02:01 GGACTGCACGGCGGTCTACAAGGACCTCCTGG
72855 72872 70658 74833 CDS product major capsid protein 72855-72872 4:01:01 CCCGGCGCTCCCGCCGCC
73042 73056 70658 74833 CDS product major capsid protein 73042-73056 4:01:01 GGCGGCGGCCCCGGG
73188 73210 70658 74833 CDS product major capsid protein 73188-73210 4:01:01 GGTGCAGTCGGTCGTGGTCCCGG
73206 73221 70658 74833 CDS product major capsid protein 73206-73221 4:01:01 CCCGGACCTGCCCCCC
73231 73293 70658 74833 CDS product major capsid protein 73231-73293 10:07:02 CCGCCCGCGGGCCCCGAGCACCCGCGCCACCCGCTGCACCCGGCGAACCTGGTGGCCAACTCC
73395 73409 70658 74833 CDS product major capsid protein 73395-73409 5:02:01 GGTGGTGGAGGCGGG
73565 73579 70658 74833 CDS product major capsid protein 73565-73579 4:01:01 CCTGCCCGGACCACC
73594 73626 70658 74833 CDS product major capsid protein 73594-73626 6:03:01 GGGCTGGGCCAGGACGCGCTGGCGGCCGCGCGG
73809 73837 70658 74833 CDS product major capsid protein 73809-73837 5:02:01 GGGCTTCGGGCTCACGGTCGTGCGGCAGG
73923 73941 70658 74833 CDS product major capsid protein 73923-73941 4:01:01 GGCAGTCGGCGGCGTCGGG
73971 73996 70658 74833 CDS product major capsid protein 73971-73996 5:02:01 GGACCTCGGCGTGGGCTACACGGCGG
73997 74013 70658 74833 CDS product major capsid protein 73997-74013 4:01:01 CCTACGCCGCCGCGGCC
74085 74105 70658 74833 CDS product major capsid protein 74085-74105 4:01:01 GGTGAACGGGGACGCGGACGG
74136 74162 70658 74833 CDS product major capsid protein 74136-74162 5:02:01 CCGCATCCGGCCACAGGGCCCCGCGCC
74173 74192 70658 74833 CDS product major capsid protein 74173-74192 4:01:01 CCCATCATGCCGGCCGCGCC

74196 74221 70658 74833 CDS product major capsid protein 74196-74221 5:02:01 GGGCACGGCGCGCGGGCAGGCCGCGG
74290 74308 70658 74833 CDS product major capsid protein 74290-74308 4:01:01 GGGCGCTCGGCGGGCGCGG
74355 74409 70658 74833 CDS product major capsid protein 74355-74409 9:06:02
CCACGGCCAGGGCGACCCGGCACACCCGCACCGCGCCACCGTGAACCCGTGGGCC
74508 74527 70658 74833 CDS product major capsid protein 74508-74527 5:02:01 GGCGGAGGTGTCGGCCAAGG
74553 74567 70658 74833 CDS product major capsid protein 74553-74567 4:01:01 GGAGGTGGGCGCGGG
74617 74638 70658 74833 CDS product major capsid protein 74617-74638 4:01:01 GGCACCGGGGAGCTCGTGGAGG
74706 74731 70658 74833 CDS product major capsid protein 74706-74731 5:02:01 CCGCCTCGCCGCGGGCGCCGGCGCCC
74736 74768 70658 74833 CDS product major capsid protein 74736-74768 7:04:01
GGGCGCGGCCGCCGAGGGCCGGCTGGAGGAGGG
74784 74804 70658 74833 CDS product major capsid protein 74784-74804 4:01:01 CCTGATCCGCGACGCCTCGCC
74977 75014 74940 75890 CDS product capsid triplex subunit 2 74977-75014 7:04:01
CCCCGGCGACCTCTCCCACAGCGACCTGGCCGCCCTCC
75070 75086 74940 75890 CDS product capsid triplex subunit 2 75070-75086 4:01:01 GGCGGACGTGGCGCTGG
75135 75160 74940 75890 CDS product capsid triplex subunit 2 75135-75160 6:03:01
CCGCCTACCGCTGCCGGTTCCCGGCC
75325 75341 74940 75890 CDS product capsid triplex subunit 2 75325-75341 4:01:01 GGCCTCGGCGGGCGCGG
75388 75437 74940 75890 CDS product capsid triplex subunit 2 75388-75437 10:07:02
GGTGGCGCGCGTGGTGGCGCGCGCGGTGGAGGCGCTGGGGGACCGCGGGG
75430 75450 74940 75890 CDS product capsid triplex subunit 2 75430-75450 4:01:01 CCGCGGGGCCGCCGCGCGCCC
75622 75641 74940 75890 CDS product capsid triplex subunit 2 75622-75641 4:01:01 GGGCCTGCAGGACGGCGTGG
75697 75714 74940 75890 CDS product capsid triplex subunit 2 75697-75714 4:01:01 CCTGGAGCCGGCCGAGCC
75717 75733 74940 75890 CDS product capsid triplex subunit 2 75717-75733 4:01:01 GGGAGGACGGCAGACGG

75749 75766 74940 75890 CDS product capsid triplex subunit 2 75749-75766 4:01:01 GGGGCGCTGGCGGCCTGG

75775 75801 74940 75890 CDS product capsid triplex subunit 2 75775-75801 6:03:01 GGCGACCCGGCTGGGGGACGCGGTCGG

75836 75866 74940 75890 CDS product capsid triplex subunit 2 75836-75866 5:02:01 GGGCCCTCGGTGGTGCGCACGGGCGAGAAGG

76107 76154 76098 81708 CDS product DNA packaging terminase subunit 1 76107-76154 10:07:02 GGCGGACGGGCGGCCAGCGGGGCGAAGGCGCTGTCGGTGGGCGGGAGG

76171 76187 76098 81708 CDS product DNA packaging terminase subunit 1 76171-76187 4:01:01 CCATCACCAGCGCCACC

76220 76265 76098 81708 CDS product DNA packaging terminase subunit 1 76220-76265 8:05:02 GGAGAAGGCCCGGGCCTCGCCGGGGCCGGCGGTGCTCTCGGTCAGG

76228 76246 76098 81708 CDS product DNA packaging terminase subunit 1 76228-76246 4:01:01 CCCGGGCCTCGCCGGGGCC

76309 76331 76098 81708 CDS product DNA packaging terminase subunit 1 76309-76331 4:01:01 GGCGCATGGTCAGCGAGGCCAGG

76327 76354 76098 81708 CDS product DNA packaging terminase subunit 1 76327-76354 5:02:01 CCAGGTCCTGCGAGGCCACCACGCGCCC

76369 76389 76098 81708 CDS product DNA packaging terminase subunit 1 76369-76389 4:01:01 GGATGAAGTGGTCAAAGGCGG

76440 76454 76098 81708 CDS product DNA packaging terminase subunit 1 76440-76454 4:01:01 GGTGGGCGGCAGTGG

76469 76497 76098 81708 CDS product DNA packaging terminase subunit 1 76469-76497 7:04:01 GGCGGGCATGGGCACGGCCCCGGAGGCGG

76582 76608 76098 81708 CDS product DNA packaging terminase subunit 1 76582-76608 5:02:01

GGCCGTCGAGGCGGCGCCGGTGCACGG
76693 76730 76098 81708 CDS product DNA packaging terminase subunit 1 76693-76730 7:04:01 CCAGGCCCAGCACCAGCCAGCTGCCGCGGTGCCGCCCC
76736 76761 76098 81708 CDS product DNA packaging terminase subunit 1 76736-76761 5:02:01 GGCGATGCCGGAGCCGGAGGCGCGGG
76832 76850 76098 81708 CDS product DNA packaging terminase subunit 1 76832-76850 4:01:01 GGAGGGCCGGTAGAGCAGG
76870 76898 76098 81708 CDS product DNA packaging terminase subunit 1 76870-76898 5:02:01 GGGTGAACACGGCCGCGGGCTCGGCCCGG
76906 76918 76098 81708 CDS product DNA packaging terminase subunit 1 76906-76918 4:01:01 CCTCGCCGCCGCC
76943 76962 76098 81708 CDS product DNA packaging terminase subunit 1 76943-76962 5:02:01 GGGCAGGAAGGTCTCGGCGG
77034 77048 76098 81708 CDS product DNA packaging terminase subunit 1 77034-77048 4:01:01 CCGCCGTGCGCCGCC
77138 77160 76098 81708 CDS product DNA packaging terminase subunit 1 77138-77160 4:01:01 GGCCTTGCCGGTGTTGGTCGAGG
77212 77228 76098 81708 CDS product DNA packaging terminase subunit 1 77212-77228 4:01:01 GGACGGCCTCGGGGCGG
77326 77363 76098 81708 CDS product DNA packaging terminase subunit 1 77326-77363 8:05:02 GGCGGCCCCGGGCCCGGGCCGGCCCGGCGCCATGGAGG
77331 77356 76098 81708 CDS product DNA packaging terminase subunit 1 77331-77356 5:02:01 CCCCGGGCCCGGGCCGGCCCGGCGCC
77404 77432 77358 79481 CDS product DNA packaging tegument protein UL17 77404-77432 5:02:01 CCGCGGCGCCGCAAACACCGTCGTCCACC

77457 77479 77358 79481 CDS product DNA packaging tegument protein UL17 77457-77479 5:02:01
GGGCCGCGGGGGCGGACCCCGGG
77493 77506 77358 79481 CDS product DNA packaging tegument protein UL17 77493-77506 4:01:01 CCGCCGCTCCCGCC
77509 77528 77358 79481 CDS product DNA packaging tegument protein UL17 77509-77528 6:03:01
GGCGGAGGCGGCCCTGGGGG
77666 77691 77358 79481 CDS product DNA packaging tegument protein UL17 77666-77691 5:02:01
CCCACGCAGCCCGCGGACCGGCCGCC
77700 77716 77358 79481 CDS product DNA packaging tegument protein UL17 77700-77716 4:01:01
CCGCCGCCGTCGGCGCC
77711 77728 77358 79481 CDS product DNA packaging tegument protein UL17 77711-77728 4:01:01
GGCGCCGACGGCGGCGGG
77808 77826 77358 79481 CDS product DNA packaging tegument protein UL17 77808-77826 4:01:01
GGCTGGAGATGGCCGACGG
77944 77968 77358 79481 CDS product DNA packaging tegument protein UL17 77944-77968 7:04:01
CCACGCCGGCCTGCCGCCCGCCGCC
77989 78012 77358 79481 CDS product DNA packaging tegument protein UL17 77989-78012 6:03:01
GGAGGCCTGCCTGGTGGAGGGCGG
78013 78034 77358 79481 CDS product DNA packaging tegument protein UL17 78013-78034 5:02:01
CCGCGCGCCGCCGCTGCCGCCC
78035 78053 77358 79481 CDS product DNA packaging tegument protein UL17 78035-78053 4:01:01
GGCGGGCGGGTCTGCCCGG
78085 78104 77358 79481 CDS product DNA packaging tegument protein UL17 78085-78104 4:01:01
CCCGGCCGCAGTCGCCCGCC

78151 78169 77358 79481 CDS product DNA packaging tegument protein UL17 78151-78169 6:03:01 GGAGGTGGTGGCGGCGCGG
78214 78226 77358 79481 CDS product DNA packaging tegument protein UL17 78214-78226 4:01:01 GGTGGCGGCGGGG
78260 78278 77358 79481 CDS product DNA packaging tegument protein UL17 78260-78278 4:01:01 GGCGCGGCGGCCGCGCCGG
78270 78301 77358 79481 CDS product DNA packaging tegument protein UL17 78270-78301 6:03:01 CCGCGCCGGCGCTGCCCCCCGACGCCGCGGCC
78311 78327 77358 79481 CDS product DNA packaging tegument protein UL17 78311-78327 4:01:01 CCGGCGCCTGCCGGGCC
78341 78387 77358 79481 CDS product DNA packaging tegument protein UL17 78341-78387 8:05:02 GGCGCCGGCGAGCCCGGGCTCGGCGCCGTGGCGCGGGCGCTGGCGGG
78400 78422 77358 79481 CDS product DNA packaging tegument protein UL17 78400-78422 5:02:01 GGCGACGGCGGCGACCCGGCGGG
78577 78592 77358 79481 CDS product DNA packaging tegument protein UL17 78577-78592 4:01:01 GGCGGGCTGGGGCGGG
78730 78753 77358 79481 CDS product DNA packaging tegument protein UL17 78730-78753 4:01:01 GGCGGCGCCGCTGGGCGAGCCCGG
78749 78775 77358 79481 CDS product DNA packaging tegument protein UL17 78749-78775 5:02:01 CCCGGCGCACCGCCGCCCGTGGAGGCC
78816 78839 77358 79481 CDS product DNA packaging tegument protein UL17 78816-78839 4:01:01 GGGCGGCGTGCGCGGGCGCGCTGG
78858 78893 77358 79481 CDS product DNA packaging tegument protein UL17 78858-78893 6:03:01 CCTGCGAGGCCCGCCCGGACCTGCCCCAGCGCTTCC
78920 78943 77358 79481 CDS product DNA packaging tegument protein UL17 78920-78943 5:02:01

GGGTGCCTGGAGGACGTGGCGCGG
78970 79001 77358 79481 CDS product DNA packaging tegument protein UL17 78970-79001 6:03:01
GGCCGGCGCGCTGGGGGCGCTCTCGGCGCGGG
79027 79037 77358 79481 CDS product DNA packaging tegument protein UL17 79027-79037 4:01:01 GGCGGCGGTGG
79057 79093 77358 79481 CDS product DNA packaging tegument protein UL17 79057-79093 8:05:02
CCCTGACGGCCCGGGCCTCCTGCCGTACCACCGCGCC
79151 79169 77358 79481 CDS product DNA packaging tegument protein UL17 79151-79169 4:01:01
GGCGGGGAGGTGATCAAGG
79189 79224 77358 79481 CDS product DNA packaging tegument protein UL17 79189-79224 7:04:01
GGCCACGACGGCGCAGGAGCCCGGGCGGCGGCTGGG
79368 79394 77358 79481 CDS product DNA packaging tegument protein UL17 79368-79394 4:01:01
CCCTGGAGGCCGACGTCGCCGCACACC
79396 79411 77358 79481 CDS product DNA packaging tegument protein UL17 79396-79411 4:01:01 GGACTGGCCGGCGCGG
79420 79440 77358 79481 CDS product DNA packaging tegument protein UL17 79420-79440 5:02:01
GGCGGTGCTCGGGCGGCTGGG
79441 79476 77358 79481 CDS product DNA packaging tegument protein UL17 79441-79476 8:05:02
CCGCCCCCTCCCCGCGCCCCCCGGCGCGCCGGCCCC
79511 79527 79508 80539 CDS product tegument protein UL16 79511-79527 4:01:01 CCGAGGACCCCGCCGCC
79671 79696 79508 80539 CDS product tegument protein UL16 79671-79696 5:02:01 CCCTGGCGCCGCCGTCCGCGTCACCC
79859 79917 79508 80539 CDS product tegument protein UL16 79859-79917 11:08:02
CCGTCCCCGTGCCGGCCGACGTTCCCGACCCGAGCGCCGAGGCCTTGCCGCCCGCGCCC
80054 80076 79508 80539 CDS product tegument protein UL16 80054-80076 4:01:01 CCCTGCTGGCCCTGTGCCCCGCC
80098 80115 79508 80539 CDS product tegument protein UL16 80098-80115 4:01:01 GGGGGCGTGCTCGGGCGG

80211 80230 79508 80539 CDS product tegument protein UL16 80211-80230 4:01:01 CCCGTGCGCCGCGCCGTGCC
80328 80357 79508 80539 CDS product tegument protein UL16 80328-80357 5:02:01 GGGCTCGCGGCGCCGGCCGCGGATCACGGG
80376 80427 79508 80539 CDS product tegument protein UL16 80376-80427 9:06:02 GGCGGGCGTCGGGCCGCGCGGGGAGGTCGTGCCGGTGAACGGCGCCGGCTGG
80646 80663 76098 81708 CDS product DNA packaging terminase subunit 1 80646-80663 5:02:01 CCTTTCCCCCCACCCCCC
80779 80811 76098 81708 CDS product DNA packaging terminase subunit 1 80779-80811 6:03:01 CCGAACCACTGCCGGAGCCGCGCCACGATCTCC
80920 80959 76098 81708 CDS product DNA packaging terminase subunit 1 80920-80959 7:04:01 CCGTGCCGCCGCGGCACCAGGAACACCGTGGCCCGCTGCC
81167 81185 76098 81708 CDS product DNA packaging terminase subunit 1 81167-81185 4:01:01 CCGCGCCGTCGTCGCCGCC
81205 81223 76098 81708 CDS product DNA packaging terminase subunit 1 81205-81223 4:01:01 CCCACCAGCCGGCTGAACC
81314 81324 76098 81708 CDS product DNA packaging terminase subunit 1 81314-81324 4:01:01 CCGCCGCCTCC
81355 81375 76098 81708 CDS product DNA packaging terminase subunit 1 81355-81375 4:01:01 CCCAGGGCCCCCGCGAGCTCC
81446 81463 76098 81708 CDS product DNA packaging terminase subunit 1 81446-81463 5:02:01 GGCGGGAGGCCGTGGCGG
81524 81547 76098 81708 CDS product DNA packaging terminase subunit 1 81524-81547 5:02:01 GGTGGCGCTTGGGGGCCGCGACGG
81578 81629 76098 81708 CDS product DNA packaging terminase subunit 1 81578-81629 11:08:02

CCTCCCCGCGGGCCCCGCCGCTGCCGCCACCCTCGGCCCGCGCCCCCCGCCC
81772 81808 81771 82445 CDS product tegument protein UL14 81772-81808 6:03:01
GGCGACGGCGGCGCTCGCGGGAGACCCGGCGCCGGGG
81828 81845 81771 82445 CDS product tegument protein UL14 81828-81845 5:02:01 GGCGGCGGCTGCGGCTGG
81905 81926 81771 82445 CDS product tegument protein UL14 81905-81926 4:01:01 GGCGCGGACCGGGACGACCCGG
81973 81995 81771 82445 CDS product tegument protein UL14 81973-81995 5:02:01 GGGGGGGCAGATCCGGGCGGCGG
82047 82078 81771 82445 CDS product tegument protein UL14 82047-82078 6:03:01
CCGCCCAAGCGGCCGTGGCCGCCGTGCTGGCC
82144 82164 81771 82445 CDS product tegument protein UL14 82144-82164 4:01:01 GGACCAGGAGGAGCGCATGGG
82177 82223 81771 82445 CDS product tegument protein UL14 82177-82223 9:06:02
GGACTGCGGGGGCGACGTGGGCGTGGGCGGGGCCTGGCTGGACGGGG
82316 82331 81771 82445 CDS product tegument protein UL14 82316-82331 4:01:01 GGAGGGCTCGCGGTGG
82366 82396 81771 82445 CDS product tegument protein UL14 82366-82396 8:05:02
GGCGGCGGCAGCGGCGGCGGGCCAGGCTGGG
82424 82455 82354 83841 CDS product tegument serine/threonine protein kinase 82424-82455 7:04:01
CCGCCCCACAGCACCCCCTGACCCCCCGCACC
82468 82486 82354 83841 CDS product tegument serine/threonine protein kinase 82468-82486 5:02:01
CCCCCGCCTCCGGCGCGCC
82524 82553 82354 83841 CDS product tegument serine/threonine protein kinase 82524-82553 7:04:01
CCGCCCTCGCCGCCTCGGGCCGCCGGATCC
82562 82590 82354 83841 CDS product tegument serine/threonine protein kinase 82562-82590 6:03:01
CCGTGCGCCGATCCGCCCCGCGAGCCGCC
82722 82739 82354 83841 CDS product tegument serine/threonine protein kinase 82722-82739 4:01:01

GGGGCCGGCGGCTACGGG
82759 82781 82354 83841 CDS product tegument serine/threonine protein kinase 82759-82781 4:01:01 CCGCCGGCGTGGCCGTAAAGACC
82870 82888 82354 83841 CDS product tegument serine/threonine protein kinase 82870-82888 4:01:01 CCGACGCCATCATCCGGCC
82940 82970 82354 83841 CDS product tegument serine/threonine protein kinase 82940-82970 7:04:01 GGACCTGGTGGCCTACGCGGAGGCGGCGGGG
82975 82991 82354 83841 CDS product tegument serine/threonine protein kinase 82975-82991 4:01:01 CCGTCCTGTCGCCCGCC
83267 83296 82354 83841 CDS product tegument serine/threonine protein kinase 83267-83296 6:03:01 CCTGCCGCCCGACGCCCGGATGCCAGACCC
83342 83367 82354 83841 CDS product tegument serine/threonine protein kinase 83342-83367 4:01:01 CCTCGACTTCCTTAACCGGCACGGCC
83378 83400 82354 83841 CDS product tegument serine/threonine protein kinase 83378-83400 4:01:01 CCCCGAGCCGCTGCCCGCGGACC
83538 83567 82354 83841 CDS product tegument serine/threonine protein kinase 83538-83567 5:02:01 GGGCTGGTCATCGGCATTCTGGCGCACCGG
83642 83682 82354 83841 CDS product tegument serine/threonine protein kinase 83642-83682 7:04:01 GGTCGTGCGGGCGGCCATCGGCAACGTGGCGGTCCGCGCGG
83675 83699 82354 83841 CDS product tegument serine/threonine protein kinase 83675-83699 4:01:01 CCGCGCGGCCTTCGACCGCACTACC
83947 83973 83838 85301 CDS product deoxyribonuclease 83947-83973 6:03:01 GGCCGAGGCGGCGGGCGAAGGCGACGG
84043 84067 83838 85301 CDS product deoxyribonuclease 84043-84067 5:02:01 GGCCGCGGCGCGCGGCGCCCGGGGG

84087 84109 83838 85301 CDS product deoxyribonuclease 84087-84109 5:02:01 CCGCGACCCGCGGCCAGGCCGCC

84120 84184 83838 85301 CDS product deoxyribonuclease 84120-84184 10:07:02
GGTGGCTGCTCCGGCGGAGCCTGGCCACGGCCTCGTCGGTCCGCTGGGGCGCCGCCGGCCCGCGG

84130 84151 83838 85301 CDS product deoxyribonuclease 84130-84151 4:01:01 CCGGCGGAGCCTGGCCACGGCC

84171 84186 83838 85301 CDS product deoxyribonuclease 84171-84186 4:01:01 CCGCCGGCCCGCGGCC

84295 84319 83838 85301 CDS product deoxyribonuclease 84295-84319 5:02:01 CCCGCCCGGCGACCTCCCGACGCCC

84323 84343 83838 85301 CDS product deoxyribonuclease 84323-84343 6:03:01 GGGCTGGCGGACGGCGGGGGG

84385 84398 83838 85301 CDS product deoxyribonuclease 84385-84398 4:01:01 GGCCGGGGCGGCGG

84409 84428 83838 85301 CDS product deoxyribonuclease 84409-84428 5:02:01 GGCGGGGGCGGGCGCAGAGG

84435 84456 83838 85301 CDS product deoxyribonuclease 84435-84456 4:01:01 CCTGCGGCCTCCTGATCGACCC

84527 84544 83838 85301 CDS product deoxyribonuclease 84527-84544 4:01:01 CCGCATCCCACCCAGACC

84666 84691 83838 85301 CDS product deoxyribonuclease 84666-84691 5:02:01 CCTTCCTGCGGTCCGTGCGCCGCCCC

84716 84743 83838 85301 CDS product deoxyribonuclease 84716-84743 4:01:01 GGCTGCCCCGGCGCCGCGGAAGCGCTGG

84753 84792 83838 85301 CDS product deoxyribonuclease 84753-84792 9:06:02
CCGACGCCTGGGCGCCGCCCCCGCCTGCCGGCGCCGCACC

84912 84929 83838 85301 CDS product deoxyribonuclease 84912-84929 4:01:01 GGGACACCGGCGAGGCGG

85000 85035 83838 85301 CDS product deoxyribonuclease 85000-85035 5:02:01
CCTCGCGGGCCACTTCCCCGATCGCGCCGCGGCGCC

85102 85115 83838 85301 CDS product deoxyribonuclease 85102-85115 4:01:01 GGGGCCGGGGGCGG

85233 85264 83838 85301 CDS product deoxyribonuclease 85233-85264 6:03:01 GGGCGGCCGTCAAGGAGTCATGGGACAGGCGG

85275 85297 85253 85555 CDS product myristylated tegument protein 85275-85297 5:02:01
CCGATGCCGCCGCAACCGCATCC

85309 85333 85253 85555 CDS product myristylated tegument protein 85309-85333 4:01:01

GGGGACCTCGTGGCCCTGGACGCGG

85353 85407 85253 85555 CDS product myristylated tegument protein 85353-85407 9:06:02
CCTCGACGACCTGCAGGCCCTGACGACCGGGGCCGCCGCCGCGGGCTCCGGCTCC

85395 85441 85253 85555 CDS product myristylated tegument protein 85395-85441 9:06:02
GGGCTCCGGCTCCGGCGGCAGCGCGGCGGGCGCCAAGGACGGCGAGG

85459 85470 85253 85555 CDS product myristylated tegument protein 85459-85470 4:01:01 GGTGGCGGCTGG

85472 85495 85253 85555 CDS product myristylated tegument protein 85472-85495 6:03:01
CCGCCGTCGTGACCACCGCCCCCC

85509 85540 85253 85555 CDS product myristylated tegument protein 85509-85540 6:03:01
CCCTCCCACGAAACCGTACCGGCCGCAGCGCC

85769 85784 85698 86957 CDS product envelope glycoprotein M 85769-85784 4:01:01 GGCGGCGGCGCCGCGG

85796 85818 85698 86957 CDS product envelope glycoprotein M 85796-85818 5:02:01 GGCGGCGTCGTCGGTGGCGCCGG

85924 85938 85698 86957 CDS product envelope glycoprotein M 85924-85938 4:01:01 GGTGGTGCAGGCAGG

85964 85989 85698 86957 CDS product envelope glycoprotein M 85964-85989 4:01:01 GGCCACGGCCAGCAGGGCGACGCCGG

86006 86025 85698 86957 CDS product envelope glycoprotein M 86006-86025 4:01:01 GGCCAGCGGCAGCCCGGGGG

86167 86186 85698 86957 CDS product envelope glycoprotein M 86167-86186 5:02:01 GGAGGAGCACGGCGGCGAGG

86290 86306 85698 86957 CDS product envelope glycoprotein M 86290-86306 4:01:01 CCAGCAGACCGCCGGCC

86321 86357 85698 86957 CDS product envelope glycoprotein M 86321-86357 6:03:01
GGCGCGCCCGGGGCCCAGCAGGCGGTGGGCGCGCGGG

86435 86451 85698 86957 CDS product envelope glycoprotein M 86435-86451 5:02:01 GGCGGCGGCGGCGCCGG

86461 86484 85698 86957 CDS product envelope glycoprotein M 86461-86484 6:03:01 GGTAGGTGGCCGCGGCCAGGACGG

86657 86682 85698 86957 CDS product envelope glycoprotein M 86657-86682 4:01:01 GGCCGCGACGGCGAGCAGGAACAGGG

86683 86705 85698 86957 CDS product envelope glycoprotein M 86683-86705 4:01:01 CCGTGTACGCCGAGAAAGCCGCC

86745 86780 85698 86957 CDS product envelope glycoprotein M 86745-86780 7:04:01
CCGAGCTCCCGCCGCGCCCACGCGCCGCCGTCGGCC
87112 87131 87078 89549 CDS product DNA replication origin-binding helicase 87112-87131 5:02:01
CCGGCGCCACCCCGCCGCCC
87154 87174 87078 89549 CDS product DNA replication origin-binding helicase 87154-87174 4:01:01
CCCCGTCTCCCTGCCCTCCCC
87252 87281 87078 89549 CDS product DNA replication origin-binding helicase 87252-87281 6:03:01
GGCTGGGCGAGGCGCTGGGGGCCGCCGCGG
87403 87437 87078 89549 CDS product DNA replication origin-binding helicase 87403-87437 7:04:01
GGGCGCGGCCTACCGGCGGCTGCTGGTGCAGGTGG
87573 87617 87078 89549 CDS product DNA replication origin-binding helicase 87573-87617 7:04:01
GGCTGCTGCGGCGGTGCCCGCGGATTGTGGCGATGGACGCGACGG
87754 87791 87078 89549 CDS product DNA replication origin-binding helicase 87754-87791 8:05:02
GGCGGCGCGGCTCGGGGGGGACGACGGCGGCGCCGGGG
88216 88237 87078 89549 CDS product DNA replication origin-binding helicase 88216-88237 6:03:01
GGTGGCCGCGGACGGCGGCTGG
88282 88307 87078 89549 CDS product DNA replication origin-binding helicase 88282-88307 5:02:01
CCGCGCGGCCTGCGCGCCCGCCTTCC
88545 88558 87078 89549 CDS product DNA replication origin-binding helicase 88545-88558 4:01:01
CCGGCCTCCCGCCC
88569 88603 87078 89549 CDS product DNA replication origin-binding helicase 88569-88603 7:04:01
CCGAGGGCCTGGCCGAGCACCCCGCCGTGGCCGCC
88861 88884 87078 89549 CDS product DNA replication origin-binding helicase 88861-88884 5:02:01

GGCCAACTGGCGGCTGTTCGGCGG
89073 89098 87078 89549 CDS product DNA replication origin-binding helicase 89073-89098 4:01:01 CCCGCGGGCCCGTAGTCGCCGTTGCC
89103 89153 87078 89549 CDS product DNA replication origin-binding helicase 89103-89153 10:07:02 GGCTCTCCGGGCGGGGGCCGGCGCAGGGGCGGGGCGTGGGGCTGGGCGCGG
89179 89209 87078 89549 CDS product DNA replication origin-binding helicase 89179-89209 6:03:01 GGTGATATGGGAGGAGGTGTTCGGGGCCCGG
89305 89327 87078 89549 CDS product DNA replication origin-binding helicase 89305-89327 5:02:01 CCGCGCCGGCCGCGCCACGCACC
89407 89467 87078 89549 CDS product DNA replication origin-binding helicase 89407-89467 11:08:02 CCGGCTCCTCGGCCTCGCCGCGCGCGCCCCCGAGCCCGGGCCCGACGCGCCCCCCGAGGCC
89749 89764 89647 91920 CDS product helicase-primase subunit 89749-89764 4:01:01 GGCGGGGCGACGGCGG
89826 89859 89647 91920 CDS product helicase-primase subunit 89826-89859 6:03:01 GGCGCGCGGGACGTGACGGCCGCGCGGTTGGAGG
89872 89886 89647 91920 CDS product helicase-primase subunit 89872-89886 4:01:01 CCGCCGCCGCTCCCC
90037 90062 89647 91920 CDS product helicase-primase subunit 90037-90062 6:03:01 CCGCCGAGGAGGCCCCCGACGCCGCC
90095 90144 89647 91920 CDS product helicase-primase subunit 90095-90144 9:06:02 GGCGCTGCGGCTGGCCGTCGAGGAGGACGCGGTGCGCGCGGCGGCCGCGG
90139 90152 89647 91920 CDS product helicase-primase subunit 90139-90152 4:01:01 CCGCGGCCGCCCCC
90208 90233 89647 91920 CDS product helicase-primase subunit 90208-90233 4:01:01 CCAACTTCGCCGCGCCCGGGAACGCC
90283 90301 89647 91920 CDS product helicase-primase subunit 90283-90301 4:01:01 CCGACATCGCCCAGCCCCC

90439 90458 89647 91920 CDS product helicase-primase subunit 90439-90458 4:01:01 CCTTCTCCCCGGTGGCCGCC
90498 90530 89647 91920 CDS product helicase-primase subunit 90498-90530 6:03:01 GGCGGGCCGGCGCAGGTCCTCGGCTTTCTGGGG
90782 90823 89647 91920 CDS product helicase-primase subunit 90782-90823 9:06:02 GGCGGGCGGCGGCAGCGGCGACGGCGCGGACTGGCCCGCGGG
90895 90929 89647 91920 CDS product helicase-primase subunit 90895-90929 6:03:01 CCTTCTTCCCCACCCTGTACGCCCACCTCGTTCCC
91073 91095 89647 91920 CDS product helicase-primase subunit 91073-91095 4:01:01 GGCGGCCGCCGTGGAGCGCGCGG
91133 91158 89647 91920 CDS product helicase-primase subunit 91133-91158 4:01:01 GGACGGCTTCTGGGGCGCCTTCGGGG
91471 91493 89647 91920 CDS product helicase-primase subunit 91471-91493 4:01:01 CCGTTGCCCGGCCAGAGCGCCCC
91510 91523 89647 91920 CDS product helicase-primase subunit 91510-91523 4:01:01 CCGCCGCCGCGGCC
91646 91667 89647 91920 CDS product helicase-primase subunit 91646-91667 4:01:01 GGACGCGGACCACGGCCCCCGG
91655 91669 89647 91920 CDS product helicase-primase subunit 91655-91669 4:01:01 CCACGGCCCCCGGCC
91701 91747 89647 91920 CDS product helicase-primase subunit 91701-91747 9:06:02 CCCTTCCCGCTGCCCTGGACCCTCTACCGCGCCCCCGTCCTGCTCCC
91833 91861 89647 91920 CDS product helicase-primase subunit 91833-91861 6:03:01 GGCGGGGACGGCGAGGGCGAGGACGACGG
92135 92158 91977 92882 CDS product tegument protein UL7 92135-92158 6:03:01 CCCGCCGCGCCGCGCCGCGCCGCC
92336 92351 91977 92882 CDS product tegument protein UL7 92336-92351 4:01:01 GGGCGGCGGGCGCAGG
92456 92514 91977 92882 CDS product tegument protein UL7 92456-92514 12:09:03 GGTCTCGGGGCGGTACAAGGCCAGCCGGTACGGCAGCACGGCGGCGGGCACGGTGGTGG
92563 92577 91977 92882 CDS product tegument protein UL7 92563-92577 4:01:01 GGAAGGCCGGCATGG

92840 92860 91977 92882 CDS product tegument protein UL7 92840-92860 5:02:01 GGCGGCTGGGCCCGGCGTGGG
92915 92994 92764 94932 CDS product capsid portal protein 92915-92994 14:11:03
GGCTGCGGCTGCGGCTGCGGCTGCGGCTGCGGCTGCGGCTGCGGCTGCGGCTGCGGCTGCGGCTGCGGCTGCGGCTGCGG
93131 93153 92764 94932 CDS product capsid portal protein 93131-93153 6:03:01 CCTCCTCCGACCTGCACGCCTCC
93208 93229 92764 94932 CDS product capsid portal protein 93208-93229 4:01:01 GGCGCCAGGATCAGGGAGATGG
93248 93264 92764 94932 CDS product capsid portal protein 93248-93264 4:01:01 CCGACACCTCCTGGCCC
93260 93283 92764 94932 CDS product capsid portal protein 93260-93283 4:01:01 GGCCCTGGTTGTTGGCCGTCCGGG
93308 93336 92764 94932 CDS product capsid portal protein 93308-93336 5:02:01
CCTGCTCCCACAGCCGCGAGAGCCGCTCC
93425 93443 92764 94932 CDS product capsid portal protein 93425-93443 4:01:01 CCTCGTGCCCCAGCCCCCC
93450 93473 92764 94932 CDS product capsid portal protein 93450-93473 4:01:01 GGCGCCCGCGGGCAGCGGCCCTGG
93492 93510 92764 94932 CDS product capsid portal protein 93492-93510 4:01:01 CCCCGCCCCCCCGCGCGCC
93594 93610 92764 94932 CDS product capsid portal protein 93594-93610 4:01:01 GGCCAGGTCGCGGTTGG
93687 93708 92764 94932 CDS product capsid portal protein 93687-93708 4:01:01 GGACGCCCGGAAGGCGTCCTGG
93717 93732 92764 94932 CDS product capsid portal protein 93717-93732 4:01:01 CCCGCCCGCGGCCGCC
93742 93761 92764 94932 CDS product capsid portal protein 93742-93761 5:02:01 CCGCCCGCCGCGCCGAAGCC
93914 93932 92764 94932 CDS product capsid portal protein 93914-93932 4:01:01 CCGCGTCCTCGCCCTCGCC
93958 94008 92764 94932 CDS product capsid portal protein 93958-94008 9:06:02
CCCGGCGCCCCCTCACCCGCGGCGCCCTCCGCGCGCCGCCCCACGAGCACC
94070 94090 92764 94932 CDS product capsid portal protein 94070-94090 4:01:01 CCAGGACCTCGCCGTGGAGCC
94241 94258 92764 94932 CDS product capsid portal protein 94241-94258 4:01:01 GGACGGTCACGGCGCTGG
94274 94310 92764 94932 CDS product capsid portal protein 94274-94310 7:04:01
CCGCCGCGAAGGCCTCGCACTCCGCCAGCACCCGCCC

94319 94343 92764 94932 CDS product capsid portal protein 94319-94343 5:02:01 CCAGGCGCCGAGCCAGCTCCCCGCC
94344 94354 92764 94932 CDS product capsid portal protein 94344-94354 4:01:01 GGCGGCGGAGG
94355 94368 92764 94932 CDS product capsid portal protein 94355-94368 4:01:01 CCGCCCCCGGCGCC
94363 94381 92764 94932 CDS product capsid portal protein 94363-94381 4:01:01 GGCGCCTGGACGGTGCCGG
94378 94402 92764 94932 CDS product capsid portal protein 94378-94402 5:02:01 CCGGCACCGCGAGGCCGATGCCGCC
94445 94468 92764 94932 CDS product capsid portal protein 94445-94468 4:01:01 GGGCGAAGCTGGCCGGGCTGCCGG
94457 94476 92764 94932 CDS product capsid portal protein 94457-94476 4:01:01 CCGGGCTGCCGGCGCCCCCC
94559 94595 92764 94932 CDS product capsid portal protein 94559-94595 7:04:01
CCGCGAGCCGCACCACCTCTGCCTCGCCGGCGGCGCC
94650 94668 92764 94932 CDS product capsid portal protein 94650-94668 4:01:01 CCAATCCCGCGCCACGTCC
94833 94866 92764 94932 CDS product capsid portal protein 94833-94866 5:02:01
CCGCACCCACTCGCCCGGCGGCCCCGGAGCGCCC
94849 94869 92764 94932 CDS product capsid portal protein 94849-94869 4:01:01 GGCGGCCCCGGAGCGCCCGGG
95057 95100 94982 97498 CDS product helicase-primase helicase subunit 95057-95100 9:06:02
GGATCCGGCAGCTGGCGGCCGCGCGGCTGCCGGCGGCGGCCCGG
95118 95144 94982 97498 CDS product helicase-primase helicase subunit 95118-95144 4:01:01
CCGCGCGGCCGCGGCGGCCGAGTCCCC
95306 95323 94982 97498 CDS product helicase-primase helicase subunit 95306-95323 5:02:01
CCGCCGCCTACCACAGCC
95411 95428 94982 97498 CDS product helicase-primase helicase subunit 95411-95428 4:01:01
CCAGCCCGCCCAGCATCC
95486 95503 94982 97498 CDS product helicase-primase helicase subunit 95486-95503 4:01:01
GGCGGCTGCTGGGCGCGG

95538 95556 94982 97498 CDS product helicase-primase helicase subunit 95538-95556 4:01:01
GGCGGAGCTCGCGGCGGGG
95646 95664 94982 97498 CDS product helicase-primase helicase subunit 95646-95664 4:01:01
GGCCGGGCTGCTGGGCCGG
95847 95871 94982 97498 CDS product helicase-primase helicase subunit 95847-95871 5:02:01
CCTCACCTGCCTTATCACCAACCCC
96114 96130 94982 97498 CDS product helicase-primase helicase subunit 96114-96130 4:01:01
CCGCCTCCACGCGCACC
96216 96236 94982 97498 CDS product helicase-primase helicase subunit 96216-96236 5:02:01
CCGCACCGCCACGGGCCAGCC
96228 96275 94982 97498 CDS product helicase-primase helicase subunit 96228-96275 8:05:02
GGGCCAGCCGGGGCTGGGGGTGGAAAAATGGCTGCAGGCCAACTCGGG
96300 96323 94982 97498 CDS product helicase-primase helicase subunit 96300-96323 5:02:01
CCGCGACCAGGACGCCAGTCCACC
96586 96604 94982 97498 CDS product helicase-primase helicase subunit 96586-96604 4:01:01
CCCGGCCTGGACCCGGGCC
96589 96610 94982 97498 CDS product helicase-primase helicase subunit 96589-96610 4:01:01
GGCCTGGACCCGGGCCGCGTGG
96666 96700 94982 97498 CDS product helicase-primase helicase subunit 96666-96700 8:05:02
GGACGACGCGGTCGACTGGCGGGGGCTGGCGGCGG
96712 96733 94982 97498 CDS product helicase-primase helicase subunit 96712-96733 4:01:01
GGCTCTGAGGCCGGCGCCGGGG
96807 96826 94982 97498 CDS product helicase-primase helicase subunit 96807-96826 4:01:01

CCAGCGCCCTACGTCCGCCC

96988 97006 94982 97498 CDS product helicase-primase helicase subunit 96988-97006 4:01:01 GGCGGGCTGGTCTCCATGG

97068 97089 94982 97498 CDS product helicase-primase helicase subunit 97068-97089 5:02:01 GGAGGAGCCCGGCCGGCGCCGG

97075 97095 94982 97498 CDS product helicase-primase helicase subunit 97075-97095 4:01:01 CCCGGCCGGCGCCGGCTCCCC

97200 97219 94982 97498 CDS product helicase-primase helicase subunit 97200-97219 4:01:01 GGAGGCGCTGGACGACAGGG

97268 97300 94982 97498 CDS product helicase-primase helicase subunit 97268-97300 6:03:01 CCATGACCATCGCCCGCTCCCAGGGCCTGAGCC

97379 97409 94982 97498 CDS product helicase-primase helicase subunit 97379-97409 6:03:01 CCTCCCGCTTCCTCCGGATGAACCTCAACCC

97588 97608 97519 98085 CDS product nuclear protein UL4 97588-97608 4:01:01 CCCCCCTGTGGGCCCTGCCCC

97652 97674 97519 98085 CDS product nuclear protein UL4 97652-97674 5:02:01 GGTGTCGGTGTGCGGCGGGCTGG

97845 97860 97519 98085 CDS product nuclear protein UL4 97845-97860 4:01:01 GGGTGGGTGGCGACGG

97895 97920 97519 98085 CDS product nuclear protein UL4 97895-97920 4:01:01 CCTGCTGGCCGTGTGCCTGGGCGCCC

97955 97988 97519 98085 CDS product nuclear protein UL4 97955-97988 7:04:01 CCAGAGCCTGGCGCCGGCGGCCGCGCCGCCCCCC

98041 98063 97519 98085 CDS product nuclear protein UL4 98041-98063 6:03:01 CCCGCCCCCCAAACCCCCTCCCC

98157 98204 98112 98558 CDS product protein V57 98157-98204 11:08:02 GGGGGCGGCGGGGGCGCGCGGCGCTCGGCGCTGGGGAGGGAGAGGGGG

98245 98276 98112 98558 CDS product protein V57 98245-98276 5:02:01 GGCGCGCGGCCCGGGCCTCTTCGGGCGAGGGG

98320 98375 98112 98558 CDS product protein V57 98320-98375 10:07:02
GGAGGCTCGGGGCCCTGCGGACGCGCCGGGGGACGGGGTGGCCGCTGGGGCTCGGG
98379 98405 98112 98558 CDS product protein V57 98379-98405 7:04:01 CCGCCAGGGCCGCCCTCCCCGGCCGCC
98435 98462 98112 98558 CDS product protein V57 98435-98462 6:03:01 GGCCGCGGCGGCGCGGTTGGACGCGAGG
98476 98510 98112 98558 CDS product protein V57 98476-98510 8:05:02 CCGCCACCGTGGCCACCCGGGCCTCCCCGAAGACC
98521 98539 98112 98558 CDS product protein V57 98521-98539 4:01:01 CCTCGCCGGAGACCTCGCC
98815 98842 98564 99217 CDS product nuclear protein UL3 98815-98842 6:03:01 CCCGCGCGCCGCGGCCCGCCCGGCCGCC
98827 98857 98564 99217 CDS product nuclear protein UL3 98827-98857 5:02:01 GGCCCGCCCGGCCGCCGGCTTGGCGCGCGGG
99014 99083 98564 99217 CDS product nuclear protein UL3 99014-99083 13:10:03
GGGTCCGGCTCTGGCGCGGGCCCGGGCTCTGCGGGCGCTGGGGGCGCGGGCCTGGCCGGCTTGGGCCCGG
99103 99135 98564 99217 CDS product nuclear protein UL3 99103-99135 6:03:01
CCCGGGCCCGCACCCGCCGCTTTCACCGTCACC
99139 99158 98564 99217 CDS product nuclear protein UL3 99139-99158 5:02:01 GGCGGCGGGCTCGGCCGCGG
99153 99184 98564 99217 CDS product nuclear protein UL3 99153-99184 7:04:01 CCGCGGCCAACACCCGCCAGCCGCGGGCCGCC
99395 99420 99264 100160 CDS product uracil-DNA glycosylase 99395-99420 5:02:01 CCGCTTCCCGGCCGCCCCAAAGGCCC
99481 99512 99264 100160 CDS product uracil-DNA glycosylase 99481-99512 7:04:01
CCAGCACCGCGTGCACCAGCCGCCCCCAGCCC
99586 99604 99264 100160 CDS product uracil-DNA glycosylase 99586-99604 4:01:01 CCCAGGCCTCCAGGCAGCC
99661 99679 99264 100160 CDS product uracil-DNA glycosylase 99661-99679 5:02:01 GGAGGCTGGGCGGGATCGG
99703 99721 99264 100160 CDS product uracil-DNA glycosylase 99703-99721 4:01:01 CCAGGCCGTGGGCCTGCCC
99724 99749 99264 100160 CDS product uracil-DNA glycosylase 99724-99749 5:02:01 GGCTGTGGTAGGGGTCCTGGCCCAGG
99929 99962 99264 100160 CDS product uracil-DNA glycosylase 99929-99962 6:03:01
GGCGGCGAAGGCGCTCCAGGTGACGGCGCGCTGG

99998 100086 99264 100160 CDS product uracil-DNA glycosylase 99998-100086 16:13:04 GGGGCGCCGGCGCTTGGGCGGCGCGGCGCCCGCGGGGGGCGACGGGGGGCTCCGGCGGGCCGAGGGCTTGGGGGACGAAGGCGCCGGGG

100081 100103 99264 100160 CDS product uracil-DNA glycosylase 100081-100103 4:01:01 CCGGGGCCGAAGACTCCGGAGCC

100098 100137 99264 100160 CDS product uracil-DNA glycosylase 100098-100137 8:05:02 GGAGCCGAGGGCTCGGGGGCCAAGGCCAAGGGCTTGGGGG

100164 100203 100144 100632 CDS product envelope glycoprotein L 100164-100203 7:04:01 GGAGCTGCAGGGACCGGCCGTGTGGTACCGGGGCGGCAGG

100213 100239 100144 100632 CDS product envelope glycoprotein L 100213-100239 7:04:01 GGTGGCGGCGGCGGGAGCGGCGCGCGG

100274 100307 100144 100632 CDS product envelope glycoprotein L 100274-100307 5:02:01 CCTGGACGGCCAAGTCCCGATACGCCGGGGTGCC

100385 100404 100144 100632 CDS product envelope glycoprotein L 100385-100404 4:01:01 CCGCGTACCACAGCGCCGCC

100438 100461 100144 100632 CDS product envelope glycoprotein L 100438-100461 5:02:01 CCTGCCAGCTCCAGGACCGCGTCC

100490 100519 100144 100632 CDS product envelope glycoprotein L 100490-100519 6:03:01 GGTCGCCGCGGCACGGCTCGGCCAGGATGG

100523 100564 100144 100632 CDS product envelope glycoprotein L 100523-100564 6:03:01 CCGCGCTGTCCACGCGCCGCGCCGCGCAAGCCCGCGGGAGCC

100574 100600 100144 100632 CDS product envelope glycoprotein L 100574-100600 5:02:01 CCGCCAGCAGCGCCGCCAGCAGCGCCC

101708 101752 101677 103839 CDS product ubiquitin E3 ligase ICP0 101708-101752 9:06:02 GGTGGTGTGGCGCGGTGGGCTGCGGGTCGCGGGGTAGGGGCGGGG

101776 101796 101677 103839 CDS product ubiquitin E3 ligase ICP0 101776-101796 6:03:01 CCGCCGCCAGGAGCCGCCGCC

101805  101836  101677  103839  CDS product ubiquitin E3 ligase ICP0  101805-101836  6:03:01 GGCCGAGCGGCCTGGCCGGGCTCGGGCCTGGG

101874  101892  101677  103839  CDS product ubiquitin E3 ligase ICP0  101874-101892  5:02:01 GGCGGGGGTCTTTAGGAGG

101903  101919  101677  103839  CDS product ubiquitin E3 ligase ICP0  101903-101919  4:01:01 CCCAGGCGGCCACCTCC

101959  102001  101677  103839  CDS product ubiquitin E3 ligase ICP0  101959-102001  8:05:02 CCGGGGTCCAGGCCGTCCCGCGCCATGGCGCCCGCGGGCCTCC

103264  103300  101677  103839  CDS product ubiquitin E3 ligase ICP0  103264-103300  7:04:01 CCCACCAGAGCGCCCTCGACCCACTCCAGCACCCGCC

103313  103329  101677  103839  CDS product ubiquitin E3 ligase ICP0  103313-103329  4:01:01 CCCCCGGCCCGTGCCCC

103330  103359  101677  103839  CDS product ubiquitin E3 ligase ICP0  103330-103359  5:02:01 GGCGCGTTCGGCAGCAGGGGCAGGCGCGGG

103415  103459  101677  103839  CDS product ubiquitin E3 ligase ICP0  103415-103459  6:03:01 GGCGCCCGGCCCCGTCGGCCGCCCCGGCCCCTTGGGGCCCCTCGG

103419  103466  101677  103839  CDS product ubiquitin E3 ligase ICP0  103419-103466  8:05:02 CCCGGCCCCGTCGGCCGCCCCGGCCCCTTGGGGCCCCTCGGCCTCCCC

103479  103500  101677  103839  CDS product ubiquitin E3 ligase ICP0  103479-103500  5:02:01 CCCGGCGTCCCCGCCGGCCGCC

103559  103594  101677  103839  CDS product ubiquitin E3 ligase ICP0  103559-103594  7:04:01 CCGCGTCCGACGCGCCGTCTGCCTCCGGCCCTCCCC

103697  103733  101677  103839  CDS product ubiquitin E3 ligase ICP0  103697-103733  7:04:01 GGCGGATGCAGGCCAGGCAGAAGGCGTGCAGGCAGGG

103792  103817  101677  103839  CDS product ubiquitin E3 ligase ICP0  103792-103817  7:04:01 GGGGCGGCGGCGGCGGCGGCAGAAGG

105167 105207 105114 109340 CDS product transcriptional regulator ICP4 105167-105207 8:05:02
GGACGCGGACGACGAGGAGGACGCGGAGGACGCGGACGAGG
105217 105255 105114 109340 CDS product transcriptional regulator ICP4 105217-105255 7:04:01
CCACGCGCGCCGGCCCGCGCCGCGGCCGGGGCCGGGGCC
105240 105268 105114 109340 CDS product transcriptional regulator ICP4 105240-105268 6:03:01
GGCCGGGGCCGGGGCCGGGCGCGGCGCGG
105270 105320 105114 109340 CDS product transcriptional regulator ICP4 105270-105320 10:07:02
CCCCCCGCCGATGCCGCCGTCGCCGGCCCCGGGAAGCCCGAGTCCGTGCCC
105336 105354 105114 109340 CDS product transcriptional regulator ICP4 105336-105354 4:01:01
GGCGCCCAGGCCGGCGGGG
105382 105464 105114 109340 CDS product transcriptional regulator ICP4 105382-105464 14:11:03
CCGGGACGCCGCCGCGCCGCGCCTGCACCAGCGTCTCCCGCGGGCCCTGGTCCGCGTCCCAGGCCACGCCGGGCGCCGCGGCC
105443 105483 105114 109340 CDS product transcriptional regulator ICP4 105443-105483 9:06:02
GGCCACGCCGGGCGCCGCGGCCGCGGCGGCGGCGGGGGCGG
105486 105500 105114 109340 CDS product transcriptional regulator ICP4 105486-105500 4:01:01 CCCGCCTCCAGCACC
105504 105523 105114 109340 CDS product transcriptional regulator ICP4 105504-105523 4:01:01
GGCGGCGCCTCGGCGTGCGG
105550 105563 105114 109340 CDS product transcriptional regulator ICP4 105550-105563 4:01:01 CCACCGCCGCGGCC
105596 105621 105114 109340 CDS product transcriptional regulator ICP4 105596-105621 5:02:01
CCGCCCGCAGGCCAGGTACACCGGCC
105605 105628 105114 109340 CDS product transcriptional regulator ICP4 105605-105628 4:01:01
GGCCAGGTACACCGGCCGCAGCGG
105646 105666 105114 109340 CDS product transcriptional regulator ICP4 105646-105666 6:03:01

GGTTGGCGGCGCGGTGGCTGG
105667 105710 105114 109340 CDS product transcriptional regulator ICP4 105667-105710 8:05:02
CCGCCTCGCCCTCCGCGAAGTCCGGCTCCCCGAGCCCCAGCGCC
105724 105747 105114 109340 CDS product transcriptional regulator ICP4 105724-105747 4:01:01
CCATGTCCTTGCGCCCGTCGAGCC
105777 105816 105114 109340 CDS product transcriptional regulator ICP4 105777-105816 7:04:01
GGCGGCACGGGCACCGCGGTGCGCGGGCCCAGGCGCGTGG
106027 106045 105114 109340 CDS product transcriptional regulator ICP4 106027-106045 4:01:01
CCTGCCCGACGGCCGGGCC
106091 106113 105114 109340 CDS product transcriptional regulator ICP4 106091-106113 6:03:01
CCGCCGCCGCGCCGCGGCCAGCC
106201 106227 105114 109340 CDS product transcriptional regulator ICP4 106201-106227 5:02:01
CCGGCGCGCCCGTCCAGGACCCGGCCC
106289 106320 105114 109340 CDS product transcriptional regulator ICP4 106289-106320 6:03:01
CCCGCCGCGCACCGGCGGCCACTCAGGCCGCC
106302 106322 105114 109340 CDS product transcriptional regulator ICP4 106302-106322 4:01:01
GGCGGCCACTCAGGCCGCCGG
106345 106360 105114 109340 CDS product transcriptional regulator ICP4 106345-106360 4:01:01 CCGCCAGCGCCTCCCC
106421 106437 105114 109340 CDS product transcriptional regulator ICP4 106421-106437 4:01:01
CCAGGCCACGCGCCGCC
106530 106559 105114 109340 CDS product transcriptional regulator ICP4 106530-106559 5:02:01
GGGCGCCAGGGCTCGGGGAAGAGCGGGTGG
106561 106580 105114 109340 CDS product transcriptional regulator ICP4 106561-106580 4:01:01

CCGCGAGCCGCGCCGCGACC

106610 106631 105114 109340 CDS product transcriptional regulator ICP4 106610-106631 4:01:01 GGCGCTGGGCGCGGGCGTGTGG

106640 106655 105114 109340 CDS product transcriptional regulator ICP4 106640-106655 4:01:01 GGGCGGCACGCGGCGG

106667 106694 105114 109340 CDS product transcriptional regulator ICP4 106667-106694 6:03:01 GGCGGCGGGGCCGCCGGGCGGCATGGGG

106705 106720 105114 109340 CDS product transcriptional regulator ICP4 106705-106720 4:01:01 GGGCGGGCAGCGGCGG

106734 106785 105114 109340 CDS product transcriptional regulator ICP4 106734-106785 9:06:02 GGGGACCCGGCGGCGCGCCGGCTTTTGGCCGGCGCCGGGCCCCTGGCCCCGG

106762 106787 105114 109340 CDS product transcriptional regulator ICP4 106762-106787 5:02:01 CCGGCGCCGGGCCCCTGGCCCCGGCC

106806 106832 105114 109340 CDS product transcriptional regulator ICP4 106806-106832 5:02:01 GGCGGGCCGTCGCGGCGGGCCTCGAGG

106825 106841 105114 109340 CDS product transcriptional regulator ICP4 106825-106841 4:01:01 CCTCGAGGCCCGCCCCC

106856 106878 105114 109340 CDS product transcriptional regulator ICP4 106856-106878 6:03:01 GGCGGCCGGGAGGGGCCGGGCGG

106894 106911 105114 109340 CDS product transcriptional regulator ICP4 106894-106911 5:02:01 GGGCGGCAGGGCCGGCGG

106905 107018 105114 109340 CDS product transcriptional regulator ICP4 106905-107018 24:21:06 CCGGCGGCCCCCTCGTCCCTTTCTTCCTCCTCCTCCTCCTCCTCGGCCCCGTCCCCGGGCCCCCCCAGCTCCTCCGGCCCCAGCACCTCCACCGCCGCGGCCGGGCTCCGGCCC

107003 107082 105114 109340 CDS product transcriptional regulator ICP4 107003-107082 14:11:03 GGCCGGGCTCCGGCCCGGACTAGGGCCCGGGCTGAGGCTGGGGCTCGGGCCTGGCGGCCCGGCGCCGGGGCTCCCGCGGG

107052  107221  105114  109340  CDS product transcriptional regulator ICP4  107052-107221  28:25:07
CCTGGCGGCCCGGCGCCGGGGCTCCCGCGGGCCTCCCCGTCCCCGTCCCCGTCCCCGTCCCCGTCCCCGTCCCCGTCCCCGTCCCCGTCCCCGTCCCCGTCCCCGTCCCCGTCCCCGTCCCCGTCCCC
GTCCCCGTCCCCGTCCCCGTCCCCGTCCCCGTCCGAGGCCCC

107216  107232  105114  109340  CDS product transcriptional regulator ICP4  107216-107232  5:02:01
GGCCCCGGCGGCGGCGG

107233  107279  105114  109340  CDS product transcriptional regulator ICP4  107233-107279  13:10:03
CCTCTTCCTCGTCCTCCTCCTCCTCCCTCTCCTCCTCCTCCTCCCCC

107290  107432  105114  109340  CDS product transcriptional regulator ICP4  107290-107432  28:25:07
CCGAGGCCCCGGCCTCCTCGTCCCCCCCGTCCCCCCCGTCCTCGTCCTCCCCGTCCTCCTCCAGGTCCGCCCGCGGTGCCCCGGCCCGCATGTCCTCCACCTCCACCCGCGCCGCGGCGCCCGGCGCG
CCGGCCTTCAGGGCC

107405  107430  105114  109340  CDS product transcriptional regulator ICP4  107405-107430  4:01:01
GGCGCCCGGCGCGCCGGCCTTCAGGG

107443  107468  105114  109340  CDS product transcriptional regulator ICP4  107443-107468  6:03:01
CCGCCAGCCGCGCCGGCACTTCCGCC

107563  107582  105114  109340  CDS product transcriptional regulator ICP4  107563-107582  5:02:01
CCGCCAGCGCGGCCGCCTCC

107718  107747  105114  109340  CDS product transcriptional regulator ICP4  107718-107747  6:03:01
GGAGCGCAGGCCGCGGCCGGCAGGCCGCGG

107728  107768  105114  109340  CDS product transcriptional regulator ICP4  107728-107768  6:03:01
CCGCGGCCGGCAGGCCGCGGCCCGCTGCAGCCGAGAGCACC

107810  107828  105114  109340  CDS product transcriptional regulator ICP4  107810-107828  4:01:01
CCGCGCCGCCTCTAGCGCC

107888  107917  105114  109340  CDS product transcriptional regulator ICP4  107888-107917  6:03:01

GGGCCCGGGCGCTGGCGGCAGCGGCGCCGG
107970 108002 105114 109340 CDS product transcriptional regulator ICP4 107970-108002 7:04:01
CCCGCGCCGCCGGCGCCGGCCTCCGGGTAGGCC
107981 108007 105114 109340 CDS product transcriptional regulator ICP4 107981-108007 5:02:01
GGCGCCGGCCTCCGGGTAGGCCATGGG
108064 108084 105114 109340 CDS product transcriptional regulator ICP4 108064-108084 4:01:01
GGCGGCTCATGGCCACGCAGG
108292 108308 105114 109340 CDS product transcriptional regulator ICP4 108292-108308 4:01:01
CCAGGTCCACCAGCGCC
108320 108338 105114 109340 CDS product transcriptional regulator ICP4 108320-108338 4:01:01
CCCCGCGTCCCCCATCTCC
108352 108371 105114 109340 CDS product transcriptional regulator ICP4 108352-108371 4:01:01
CCGCGGGCCCCGCGGCCGCC
108454 108469 105114 109340 CDS product transcriptional regulator ICP4 108454-108469 5:02:01 GGCCGGGCGGCGGCGG
108477 108493 105114 109340 CDS product transcriptional regulator ICP4 108477-108493 4:01:01
CCGGGCCAGGCCTCGCC
108479 108499 105114 109340 CDS product transcriptional regulator ICP4 108479-108499 4:01:01
GGGCCAGGCCTCGCCGGAGGG
108585 108647 105114 109340 CDS product transcriptional regulator ICP4 108585-108647 11:08:02
GGGCGCCGCGGCGAGGGCGCCGGGCCCTGGCCTTGGCTGGATCCGCGGGCGGGTCCGGCGGGG
108650 108671 105114 109340 CDS product transcriptional regulator ICP4 108650-108671 4:01:01
CCCCCCGGCGCCAGGGCTCCCC
108683 108715 105114 109340 CDS product transcriptional regulator ICP4 108683-108715 6:03:01

CCCCGAGTCCCCGGCCCCCGAGTCCCTAAATCC
108722 108738 105114 109340 CDS product transcriptional regulator ICP4 108722-108738 5:02:01
GGCCGCGGCGGCGGCGG
108739 108759 105114 109340 CDS product transcriptional regulator ICP4 108739-108759 4:01:01
CCCCCGTGTCATCCCCAACCC
108770 108792 105114 109340 CDS product transcriptional regulator ICP4 108770-108792 5:02:01
GGCATCGGCCCCGGAGTCGGCGG
108806 108837 105114 109340 CDS product transcriptional regulator ICP4 108806-108837 6:03:01
GGTCCCCGGCTCGGGCTTGGGCCTGGCTTCGG
108887 108901 105114 109340 CDS product transcriptional regulator ICP4 108887-108901 4:01:01 GGGCGCAGGCGGCGG
108922 108939 105114 109340 CDS product transcriptional regulator ICP4 108922-108939 4:01:01
GGGCGGCGGCCACGACGG
108931 108959 105114 109340 CDS product transcriptional regulator ICP4 108931-108959 6:03:01
CCACGACGGCCGCCTCAGCCGCGGCCGCC
108953 108975 105114 109340 CDS product transcriptional regulator ICP4 108953-108975 5:02:01
GGCCGCCTCGGCTTCGGCGGCGG
108979 109046 105114 109340 CDS product transcriptional regulator ICP4 108979-109046 12:09:03
CCTCCGCCGCGGCCGCGAGCGCCCGCGCCGCGGCCCAGGCGCCCCCGGCCGCGGCGTCCTCTGGGGCC
109010 109032 105114 109340 CDS product transcriptional regulator ICP4 109010-109032 4:01:01
GGCCCAGGCGCCCCCGGCCGCGG
109041 109088 105114 109340 CDS product transcriptional regulator ICP4 109041-109088 7:04:01
GGGGCCTCGGAGGGCGCCGAGGCCCCCGGGCCCCCCGGGTTCTCCGGG
109057 109109 105114 109340 CDS product transcriptional regulator ICP4 109057-109109 12:09:03

CCGAGGCCCCCGGGCCCCCCGGGTTCTCCGGGTCCTCCTCTTCCTCCTCCCCC

109144 109175 105114 109340 CDS product transcriptional regulator ICP4 109144-109175 7:04:01 CCCCCGCGGCCACCATGCGCCACAGCTCCGCC

109181 109199 105114 109340 CDS product transcriptional regulator ICP4 109181-109199 4:01:01 GGCGCGGGCGGCCCGCCGG

109240 109277 105114 109340 CDS product transcriptional regulator ICP4 109240-109277 9:06:02 CCGGGTCCTCCTCCGGGCCCCCCTCCGGGCCGAGGTCC

114243 114306 114207 115151 CDS product regulatory protein ICP22 114243-114306 11:08:02 CCTGCCGCCTCTGCCGCTCGCCCGACCGCGTGCTCCCAGGGCCCGCGCCTGCCCAGGCGGGCCC

114316 114342 114207 115151 CDS product regulatory protein ICP22 114316-114342 5:02:01 CCGCCGCGGGCCTGGCGCCTTCTGCCC

114536 114560 114207 115151 CDS product regulatory protein ICP22 114536-114560 4:01:01 CCCGAGCAGGCCGGCCCGCTGCACC

114611 114633 114207 115151 CDS product regulatory protein ICP22 114611-114633 5:02:01 CCCCCGGCGCGCCTGCCGGGCCC

114744 114758 114207 115151 CDS product regulatory protein ICP22 114744-114758 4:01:01 CCGCCGTCCCCTCCC

114777 114844 114207 115151 CDS product regulatory protein ICP22 114777-114844 15:12:03 CCGCCTCCGGGCCCCCCTCTTCCTCCGCCGCCTCCTCCCGGTCCTCGGCCTCGTCGTCCTCGTCGTCC

114956 114975 114207 115151 CDS product regulatory protein ICP22 114956-114975 5:02:01 GGCGCCAACGGCGGCGGCGG

114981 115004 114207 115151 CDS product regulatory protein ICP22 114981-115004 6:03:01 CCGCCGCCGGCCGCGACAACCCCC

115036 115054 114207 115151 CDS product regulatory protein ICP22 115036-115054 5:02:01 CCGCCGCCGCCCCGCGCCC

115068 115108 114207 115151 CDS product regulatory protein ICP22 115068-115108 8:05:02

CCTCCGGCTCCTCGACGGCCTCGTCGCCGGGCCCGTCCCCC
115124 115139 114207 115151 CDS product regulatory protein ICP22 115124-115139 4:01:01 CCGCCCAGGCGCCGCC
115968 115990 115937 116680 CDS product virion protein V67 115968-115990 5:02:01 CCCGCCGCGGGCCCGGCCCGGCC
115976 116004 115937 116680 CDS product virion protein V67 115976-116004 7:04:01
GGGCCCGGCCCGGCCGGCGGCTGCGGCGG
116130 116145 115937 116680 CDS product virion protein V67 116130-116145 4:01:01 GGCGGTGCGGCAGCGG
116372 116388 115937 116680 CDS product virion protein V67 116372-116388 4:01:01 GGCCGGCGCGGCGCGGG
116495 116515 115937 116680 CDS product virion protein V67 116495-116515 4:01:01 GGGGGGAGCATGGCGTACAGG
116541 116599 115937 116680 CDS product virion protein V67 116541-116599 10:07:02
CCCTGAAAAGCCGCCAGCCGACGGGCCCGCCGTCGCGGCCGGGGCCAGCGTCCGCGGCC
116814 116846 116738 117418 CDS product virion protein US2 116814-116846 6:03:01
CCGCGCCGCGGTCCACGACCTCGTAAACCAGCC
116911 116929 116738 117418 CDS product virion protein US2 116911-116929 4:01:01 GGCGATGGCCGGCCCGCGG
116938 116953 116738 117418 CDS product virion protein US2 116938-116953 4:01:01 CCGCCACGCTGCCCCC
117115 117136 116738 117418 CDS product virion protein US2 117115-117136 5:02:01 CCACTCTGCCGCTGCCGCCACC
117137 117186 116738 117418 CDS product virion protein US2 117137-117186 9:06:02
GGCGGTGCGGGCGGCGAACCGGGCCGGCAGTCCCGGATGCGGGCCAGGGG
117204 117223 116738 117418 CDS product virion protein US2 117204-117223 5:02:01 CCACCCGCGCCGCCGGCGCC
117233 117247 116738 117418 CDS product virion protein US2 117233-117247 4:01:01 CCCGCCGCCACGTCC
117273 117292 116738 117418 CDS product virion protein US2 117273-117292 4:01:01 CCGGCGTCCCTAGCCGGCCC
117672 117698 117541 118875 CDS product serine/threonine protein kinase US3 117672-117698 5:02:01
CCCGCGCGCCTTGCCGCGGAAGCCCCC
117773 117816 117541 118875 CDS product serine/threonine protein kinase US3 117773-117816 8:05:02

GGACGGGTGCGGGTCAGGGGCCGCGGCCGGGGACGATAGGGCGG

117823 117852 117541 118875 CDS product serine/threonine protein kinase US3 117823-117852 8:05:02

CCGCAGCCGAGGCCGCCCCCACCCCCGCCC

117894 117927 117541 118875 CDS product serine/threonine protein kinase US3 117894-117927 5:02:01

GGCGACTGGGGCGACGACGGGCCCGCCGGCGGGG

118156 118179 117541 118875 CDS product serine/threonine protein kinase US3 118156-118179 4:01:01

GGGAGCTGGTGTGCGTGGTGCTGG

118244 118263 117541 118875 CDS product serine/threonine protein kinase US3 118244-118263 4:01:01

GGCGCTGGCGGTGACGCGGG

118277 118308 117541 118875 CDS product serine/threonine protein kinase US3 118277-118308 5:02:01

CCTCGCGTACCTGCACTCCCGCCGGATCGCCC

118364 118388 117541 118875 CDS product serine/threonine protein kinase US3 118364-118388 4:01:01

GGGCGACTTCGGCGCGGCACACGGG

118413 118431 117541 118875 CDS product serine/threonine protein kinase US3 118413-118431 4:01:01

GGCCTGGCTGGCACCCTGG

118580 118600 117541 118875 CDS product serine/threonine protein kinase US3 118580-118600 4:01:01

GGCAGCGGGCCTGGGCGAAGG

118631 118652 117541 118875 CDS product serine/threonine protein kinase US3 118631-118652 4:01:01

CCGCCGGCTGGCCGTGCGCGCC

118661 118704 117541 118875 CDS product serine/threonine protein kinase US3 118661-118704 8:05:02

CCCGCCTAGCCCCACCGACCGGCTGACCCGCAACTTCCAACGCC

118718 118746 117541 118875 CDS product serine/threonine protein kinase US3 118718-118746 5:02:01

CCGAGAGCCGCGCAGCCCGTACCCCTGCC

118778 118791 117541 118875 CDS product serine/threonine protein kinase US3 118778-118791 4:01:01 CCACCTCCTGCACC
118985 119014 118983 120305 CDS product envelope glycoprotein G 118985-119014 6:03:01 CCCGCCGCCGCTCAAGCCGGCACCCTCGCC
119098 119164 118983 120305 CDS product envelope glycoprotein G 119098-119164 10:07:02 CCGCAACAGCCTGGGGCCCCTTCGCCCGCTGGGGCCCGCCCGAGACCTGGGAGCCTTGGACCTGGCC
119146 119177 118983 120305 CDS product envelope glycoprotein G 119146-119177 5:02:01 GGGAGCCTTGGACCTGGCCTCGCGGATTTCGG
119570 119602 118983 120305 CDS product envelope glycoprotein G 119570-119602 7:04:01 GGGCTGGACCGGGGGATCGGCGCGGCCCTGGGG
119623 119639 118983 120305 CDS product envelope glycoprotein G 119623-119639 4:01:01 GGTCCTGTCGGAGGAGG
119910 119936 118983 120305 CDS product envelope glycoprotein G 119910-119936 6:03:01 CCATGCCCCTTGGCCTGCCGGCCGGCC
120007 120023 118983 120305 CDS product envelope glycoprotein G 120007-120023 5:02:01 GGCGGCGGTGGTAGTGG
120029 120085 118983 120305 CDS product envelope glycoprotein G 120029-120085 11:08:02 CCACCACCGCCCCAGAAGTCCGCCCGCTCCGAGCGCCGCTCCGATTCCCGGGCAACC
120211 120235 118983 120305 CDS product envelope glycoprotein G 120211-120235 5:02:01 CCAGCTCGCCCGCAAGGCCACCGCC
120246 120265 118983 120305 CDS product envelope glycoprotein G 120246-120265 4:01:01 CCCGCGCCGCCCAGTTTGCC
120654 120674 120560 121813 CDS product envelope glycoprotein D 120654-120674 5:02:01 CCCGCCGGCGTACCCGCCGCC
120704 120739 120560 121813 CDS product envelope glycoprotein D 120704-120739 7:04:01 CCACCGGGCCCATCCCGTCGCCCTTCCAGGACGGCC
120966 120983 120560 121813 CDS product envelope glycoprotein D 120966-120983 4:01:01 CCGCTACCGCACGCCCCC

120989 121007 120560 121813 CDS product envelope glycoprotein D 120989-121007 4:01:01 GGGACGGCTTCCTGGCGGG
121035 121051 120560 121813 CDS product envelope glycoprotein D 121035-121051 4:01:01 GGGGCTGGTCATGGCGG
121187 121210 120560 121813 CDS product envelope glycoprotein D 121187-121210 4:01:01 CCTTCGGCGCCTGCTTCCCGATCC
121357 121368 120560 121813 CDS product envelope glycoprotein D 121357-121368 4:01:01 CCGCCGCCCGCC
121379 121395 120560 121813 CDS product envelope glycoprotein D 121379-121395 4:01:01 CCTCTCCCGCACCGCCC
121408 121486 120560 121813 CDS product envelope glycoprotein D 121408-121486 17:14:04
GGCGAGGCCCACGGAGAAGGCGGCGGGGAAGAGGACGGAGCCGGCGGGCAGGAGACCGGCGGCGAGGGGGAGGGCCCGG
121482 121545 120560 121813 CDS product envelope glycoprotein D 121482-121545 10:07:02
CCCGGCCGCCGCTGGGCCCGACGGCGCCCCCCCGGGCGAGCCGAAGCCCGGCCCCGGCGGCCCC
121531 121552 120560 121813 CDS product envelope glycoprotein D 121531-121552 5:02:01 GGCCCCGGCGGCCCCGGCGCGG
121600 121643 120560 121813 CDS product envelope glycoprotein D 121600-121643 11:08:02
CCCCCGCCCACCCCCGCCACGCCCGCGCCCCCTACTGCCCTGCC
121644 121675 120560 121813 CDS product envelope glycoprotein D 121644-121675 6:03:01
GGTCGGCATCGGGGTCGGCATCGCGGCCGCGG
121691 121707 120560 121813 CDS product envelope glycoprotein D 121691-121707 5:02:01 CCGCCGCCGGTGCCGCC
121984 122010 121963 123126 CDS product envelope glycoprotein I 121984-122010 6:03:01
GGGTGGCGGCGCTGGCCGCGCGGGCGG
122037 122050 121963 123126 CDS product envelope glycoprotein I 122037-122050 4:01:01 GGCGAGGCGGTCGG
122080 122093 121963 123126 CDS product envelope glycoprotein I 122080-122093 4:01:01 CCGTCCACCCAGCC
122129 122150 121963 123126 CDS product envelope glycoprotein I 122129-122150 4:01:01 CCTGGAGCACCAGCTCCCGGCC
122227 122245 121963 123126 CDS product envelope glycoprotein I 122227-122245 4:01:01 CCGCGTTCGCCTCCTGCCC
122263 122285 121963 123126 CDS product envelope glycoprotein I 122263-122285 5:02:01 CCTTCCGCTCCTGCCTGCACGCC
122350 122372 121963 123126 CDS product envelope glycoprotein I 122350-122372 5:02:01 CCATCGCCGGCCCGCGCCCGGCC

122400  122422  121963  123126  CDS product envelope glycoprotein I  122400-122422  4:01:01 GGCATCGACGGCGGCGCCGAGGG
122416  122436  121963  123126  CDS product envelope glycoprotein I  122416-122436  4:01:01 CCGAGGGCGCCGAGCGCCGCC
122481  122531  121963  123126  CDS product envelope glycoprotein I  122481-122531  8:05:02
CCCGGCTCCGACGACCCGGAGGCCGCGACCGCGCGCCCCGGCGGCGCCGCC
122520  122544  121963  123126  CDS product envelope glycoprotein I  122520-122544  4:01:01
GGCGGCGCCGCCGGGGCGATCGCGG
122610  122645  121963  123126  CDS product envelope glycoprotein I  122610-122645  8:05:02
CCCGCGGGCGCCGCCGCCGCTACGCCCACCCCCACC
122648  122665  121963  123126  CDS product envelope glycoprotein I  122648-122665  5:02:01 GGACGGCGGGGGCGAAGG
122678  122715  121963  123126  CDS product envelope glycoprotein I  122678-122715  6:03:01
GGATCAGGCCGGGGAGACGTCGGAGACCGGGACGAGGG
122720  122800  121963  123126  CDS product envelope glycoprotein I  122720-122800  17:14:04
CCTGACCCCCGACCCCGCCGCGACCCCGACCCCCCCCGCTACGCCCCCGGCACCCGCCGACAACTCCTCCTCCCGCGCTCC
122801  122817  121963  123126  CDS product envelope glycoprotein I  122801-122817  4:01:01 GGGCGCGGCCGGCGCGG
122945  122967  121963  123126  CDS product envelope glycoprotein I  122945-122967  5:02:01 CCGCCGCCTGCTGCCGTGCTCCC
123048  123065  121963  123126  CDS product envelope glycoprotein I  123048-123065  4:01:01 CCGATCGCCCCTGCCACC
123087  123110  121963  123126  CDS product envelope glycoprotein I  123087-123110  5:02:01 CCGAAGGCGCCCCCCCTCGCCACC
123421  123439  123415  125214  CDS product envelope glycoprotein E  123421-123439  5:02:01 CCACCGCGCCGCCCCGGCC
123489  123514  123415  125214  CDS product envelope glycoprotein E  123489-123514  5:02:01
GGGCTACCGGTCGGGGCCGGCCTTGG
123505  123542  123415  125214  CDS product envelope glycoprotein E  123505-123542  7:04:01
CCGGCCTTGGCCCCAGCCCCAGCCCCGAAGCCGACACC
123543  123567  123415  125214  CDS product envelope glycoprotein E  123543-123567  5:02:01

GGGGCAAAGGCCCCGGCCGGCGCGG
123591 123623 123415 125214 CDS product envelope glycoprotein E 123591-123623 6:03:01
CCCGTCTTCCTCCCTGGGCCTGACCCGCGCCCC
123668 123705 123415 125214 CDS product envelope glycoprotein E 123668-123705 7:04:01
CCCACCGCCCGAGCCGACGCCCGTCTGCCTCGACGACC
123896 123907 123415 125214 CDS product envelope glycoprotein E 123896-123907 4:01:01 GGAGGACGGCGG
123917 123944 123415 125214 CDS product envelope glycoprotein E 123917-123944 5:02:01
CCTGTACGACCGGGCCGCCGGCGACGCC
123928 123954 123415 125214 CDS product envelope glycoprotein E 123928-123954 4:01:01
GGGCCGCCGGCGACGCCGGCGACGAGG
124011 124062 123415 125214 CDS product envelope glycoprotein E 124011-124062 11:08:02
GGGCAGGGCGAGGGCGGGGAAGGGGAAGGGGGGAAGGGGGGGCGAGGGGCGG
124063 124135 123415 125214 CDS product envelope glycoprotein E 124063-124135 18:15:04
CCAAGCCCACCCCCACCCCCACCCCCGCCCCCAGCCCGCCCCGCCCCACGCCCGCGCGCCCCGCGCCCCCCCC
124138 124150 123415 125214 CDS product envelope glycoprotein E 124138-124150 4:01:01 GGCGGCGGCACGG
124334 124363 123415 125214 CDS product envelope glycoprotein E 124334-124363 6:03:01
CCACCCCGAGGCGCCGGCCTGCCTGCACCC
124392 124409 123415 125214 CDS product envelope glycoprotein E 124392-124409 4:01:01 CCCTACCGCTCCGAGACC
124436 124462 123415 125214 CDS product envelope glycoprotein E 124436-124462 7:04:01
CCGCCCAGCCTCCGCCGACCGCTGGCC
124545 124568 123415 125214 CDS product envelope glycoprotein E 124545-124568 4:01:01 GGCGCGCCGGCCTCGGCCTCGGGG
124596 124614 123415 125214 CDS product envelope glycoprotein E 124596-124614 4:01:01 GGCCACGTGGAGGCCTGGG
124654 124723 123415 125214 CDS product envelope glycoprotein E 124654-124723 14:11:03

CCGTCACCGACCACACGCGCCCCGCGGCCGCCGACGCCCCCGAGCCGAGCCCGCCGCCCGCCGACGGGCC

124765 124818 123415 125214 CDS product envelope glycoprotein E 124765-124818 8:05:02

GGCTCGTGGTGCTGGGGGGCGCGCTCGGGCTCGCGGGCCTAATCGGCGTCGCGG

124900 124921 123415 125214 CDS product envelope glycoprotein E 124900-124921 5:02:01 CCAGCCTGCCGACCAACGAGCC

125014 125091 123415 125214 CDS product envelope glycoprotein E 125014-125091 15:12:03

CCGACGAGCCCCCCGCCACCGCCGCCTACGACCTCGCCGGGCCCGCCCAGGGGGCCGGCGGGCCCGCGCGCCCGAGCC

125063 125075 123415 125214 CDS product envelope glycoprotein E 125063-125075 4:01:01 GGGGGCCGGCGGG

125096 125118 123415 125214 CDS product envelope glycoprotein E 125096-125118 4:01:01 GGGCTTCAAGGTCTGGTTTAGGG

125145 125167 123415 125214 CDS product envelope glycoprotein E 125145-125167 4:01:01 CCGGCGCGGCCCCAGACCGCGCC

125180 125192 123415 125214 CDS product envelope glycoprotein E 125180-125192 4:01:01 GGTGGCGGCGCGG

125340 125383 125306 125710 CDS product membrane protein US9 125340-125383 8:05:02

CCGGGGCGCCGACGCGGCCGACGCCGCAAGCCCTCCGCCGCCCC

125451 125467 125306 125710 CDS product membrane protein US9 125451-125467 4:01:01 GGCGGCGGCCGCGCCGG

125571 125604 125306 125710 CDS product membrane protein US9 125571-125604 8:05:02

GGGGCGGCAGCAGCGGCGGCGGCACCGGCGGCGG

125613 125672 125306 125710 CDS product membrane protein US9 125613-125672 11:08:02

GGTGGCGGCGGCGCTGGCCTGTGCGGGGCTTGGGGCCTGCGCGGCCGCCGCGGCGGCGGG

126734 126749 126724 127668 CDS product regulatory protein ICP22 126734-126749 4:01:01 GGCGGCGCCTGGGCGG

126765 126805 126724 127668 CDS product regulatory protein ICP22 126765-126805 8:05:02

GGGGGACGGGCCCGGCGACGAGGCCGTCGAGGAGCCGGAGG

126819 126837 126724 127668 CDS product regulatory protein ICP22 126819-126837 5:02:01 GGGCGCGGGGCGGCGGCGG

126869 126892 126724 127668 CDS product regulatory protein ICP22 126869-126892 6:03:01

GGGGGTTGTCGCGGCCGGCGGCGG

126898  126917  126724  127668  CDS product regulatory protein ICP22    126898-126917   5:02:01 CCGCCGCCGCCGTTGGCGCC

127029  127096  126724  127668  CDS product regulatory protein ICP22    127029-127096   15:12:03
GGACGACGAGGACGACGAGGCCGAGGACCGGGAGGAGGCGGCGGAGGAAGAGGGGGGCCCGGAGGCGG

127115  127129  126724  127668  CDS product regulatory protein ICP22    127115-127129   4:01:01 GGGAGGGGACGGCGG

127240  127262  126724  127668  CDS product regulatory protein ICP22    127240-127262   5:02:01
GGGCCCGGCAGGCGCGCCGGGGG

127313  127337  126724  127668  CDS product regulatory protein ICP22    127313-127337   4:01:01
GGTGCAGCGGGCCGGCCTGCTCGGG

127531  127557  126724  127668  CDS product regulatory protein ICP22    127531-127557   5:02:01
GGGCAGAAGGCGCCAGGCCCGCGGCGG

127567  127630  126724  127668  CDS product regulatory protein ICP22    127567-127630   11:08:02
GGGCCCGCCTGGGCAGGCGCGGGCCCTGGGAGCACGCGGTCGGGCGAGCGGCAGAGGCGGCAGG

132596  132633  132535  136761  CDS product transcriptional regulator ICP4    132596-132633   9:06:02
GGACCTCGGCCCGGAGGGGGGCCCGGAGGAGGACCCGG

132674  132692  132535  136761  CDS product transcriptional regulator ICP4    132674-132692   4:01:01
CCGGCGGGCCGCCCGCGCC

132698  132729  132535  136761  CDS product transcriptional regulator ICP4    132698-132729   7:04:01
GGCGGAGCTGTGGCGCATGGTGGCCGCGGGGG

132764  132816  132535  136761  CDS product transcriptional regulator ICP4    132764-132816   12:09:03
GGGGGAGGAGGAAGAGGAGGACCCGGAGAACCCGGGGGGCCCGGGGGCCTCGG

132785  132832  132535  136761  CDS product transcriptional regulator ICP4    132785-132832   7:04:01
CCCGGAGAACCCGGGGGGCCCGGGGGCCTCGGCGCCCTCCGAGGCCCC

132827  132894  132535  136761  CDS product transcriptional regulator ICP4    132827-132894   12:09:03

GGCCCCAGAGGACGCCGCGGCCGGGGGCGCCTGGGCCGCGGCGCGGGCGCTCGCGGCCGCGGCGGAGG
132841 132863 132535 136761 CDS product transcriptional regulator ICP4 132841-132863 4:01:01
CCGCGGCCGGGGGCGCCTGGGCC
132898 132920 132535 136761 CDS product transcriptional regulator ICP4 132898-132920 5:02:01
CCGCCGCCGAAGCCGAGGCGGCC
132914 132942 132535 136761 CDS product transcriptional regulator ICP4 132914-132942 6:03:01
GGCGGCCGCGGCTGAGGCGGCCGTCGTGG
132934 132951 132535 136761 CDS product transcriptional regulator ICP4 132934-132951 4:01:01
CCGTCGTGGCCGCCGCCC
132972 132986 132535 136761 CDS product transcriptional regulator ICP4 132972-132986 4:01:01 CCGCCGCCTGCGCCC
133036 133067 132535 136761 CDS product transcriptional regulator ICP4 133036-133067 6:03:01
CCGAAGCCAGGCCCAAGCCCGAGCCGGGGACC
133081 133103 132535 136761 CDS product transcriptional regulator ICP4 133081-133103 5:02:01
CCGCCGACTCCGGGGCCGATGCC
133114 133134 132535 136761 CDS product transcriptional regulator ICP4 133114-133134 4:01:01
GGGTTGGGGATGACACGGGGG
133135 133151 132535 136761 CDS product transcriptional regulator ICP4 133135-133151 5:02:01
CCGCCGCCGCCGCGGCC
133158 133190 132535 136761 CDS product transcriptional regulator ICP4 133158-133190 6:03:01
GGATTTAGGGACTCGGGGGCCGGGGACTCGGGG
133202 133223 132535 136761 CDS product transcriptional regulator ICP4 133202-133223 4:01:01
GGGGAGCCCTGGCGCCGGGGGG
133226 133288 132535 136761 CDS product transcriptional regulator ICP4 133226-133288 11:08:02

CCCCGCCGGACCCGCCCGCGGATCCAGCCAAGGCCAGGGCCCGGCGCCCTCGCCGCGGCGCCC
133374 133394 132535 136761 CDS product transcriptional regulator ICP4 133374-133394 4:01:01
CCCTCCGGCGAGGCCTGGCCC
133380 133396 132535 136761 CDS product transcriptional regulator ICP4 133380-133396 4:01:01
GGCGAGGCCTGGCCCGG
133404 133419 132535 136761 CDS product transcriptional regulator ICP4 133404-133419 5:02:01 CCGCCGCCGCCCGGCC
133502 133521 132535 136761 CDS product transcriptional regulator ICP4 133502-133521 4:01:01
GGCGGCCGCGGGGCCCGCGG
133535 133553 132535 136761 CDS product transcriptional regulator ICP4 133535-133553 4:01:01
GGAGATGGGGGACGCGGGG
133565 133581 132535 136761 CDS product transcriptional regulator ICP4 133565-133581 4:01:01
GGCGCTGGTGGACCTGG
133789 133809 132535 136761 CDS product transcriptional regulator ICP4 133789-133809 4:01:01
CCTGCGTGGCCATGAGCCGCC
133866 133892 132535 136761 CDS product transcriptional regulator ICP4 133866-133892 5:02:01
CCCATGGCCTACCCGGAGGCCGGCGCC
133871 133903 132535 136761 CDS product transcriptional regulator ICP4 133871-133903 7:04:01
GGCCTACCCGGAGGCCGGCGCCGGCGGCGCGGG
133956 133985 132535 136761 CDS product transcriptional regulator ICP4 133956-133985 6:03:01
CCGGCGCCGCTGCCGCCAGCGCCCGGGCCC
134045 134063 132535 136761 CDS product transcriptional regulator ICP4 134045-134063 4:01:01
GGCGCTAGAGGCGGCGCGG
134105 134145 132535 136761 CDS product transcriptional regulator ICP4 134105-134145 6:03:01

GGTGCTCTCGGCTGCAGCGGGCCGCGGCCTGCCGGCCGCGG

134126  134155  132535  136761  CDS product transcriptional regulator ICP4  134126-134155  6:03:01

CCGCGGCCTGCCGGCCGCGGCCTGCGCTCC

134291  134310  132535  136761  CDS product transcriptional regulator ICP4  134291-134310  5:02:01

GGAGGCGGCCGCGCTGGCGG

134405  134430  132535  136761  CDS product transcriptional regulator ICP4  134405-134430  6:03:01

GGCGGAAGTGCCGGCGCGGCTGGCGG

134441  134583  132535  136761  CDS product transcriptional regulator ICP4  134441-134583  28:25:07

GGCCCTGAAGGCCGGCGCGCCGGGCGCCGCGGCGCGGGTGGAGGTGGAGGACATGCGGGCCGGGGCACCGCGGGCGGACCTGGAGGAGGACGGGGAGGACGAGGACGGGGGGGACGGGGGGGACGAGGAGGCCGGGGCCTCGG

134443  134468  132535  136761  CDS product transcriptional regulator ICP4  134443-134468  4:01:01

CCCTGAAGGCCGGCGCGCCGGGCGCC

134594  134640  132535  136761  CDS product transcriptional regulator ICP4  134594-134640  13:10:03

GGGGGAGGAGGAGGAGGAGAGGGAGGAGGAGGAGGACGAGGAAGAGG

134641  134657  132535  136761  CDS product transcriptional regulator ICP4  134641-134657  5:02:01

CCGCCGCCGCCGGGGCC

134652  134821  132535  136761  CDS product transcriptional regulator ICP4  134652-134821  28:25:07

GGGGCCTCGGACGGGGACGGGGACGGGGACGGGGACGGGGACGGGGACGGGGACGGGGACGGGGACGGGGACGGGGACGGGGACGGGGACGGGGACGGGGACGGGGACGGGGACGGGGACGGGGACGGGGACGGGGAGGCCCGCGGGAGCCCCGGCGCCGGGCCGCCAGG

134791  134870  132535  136761  CDS product transcriptional regulator ICP4  134791-134870  14:11:03

CCCGCGGGAGCCCCGGCGCCGGGCCGCCAGGCCCGAGCCCCAGCCTCAGCCCGGGCCCTAGTCCGGGCCGGAGCCCGGCC

134855  134968  132535  136761  CDS product transcriptional regulator ICP4  134855-134968  24:21:06

GGGCCGGAGCCCGGCCGCGGCGGTGGAGGTGCTGGGGCCGGAGGAGCTGGGGGGGCCCGGGGACGGGGCCGAGGAGGAGGAGGAGGAAGAAAGGGACGAGGGGGCCGCCGG

134962 134979 132535 136761 CDS product transcriptional regulator ICP4 134962-134979 5:02:01
CCGCCGGCCCTGCCGCCC
134995 135017 132535 136761 CDS product transcriptional regulator ICP4 134995-135017 6:03:01
CCGCCCGGCCCCTCCCGGCCGCC
135032 135048 132535 136761 CDS product transcriptional regulator ICP4 135032-135048 4:01:01
GGGGGCGGGCCTCGAGG
135041 135067 132535 136761 CDS product transcriptional regulator ICP4 135041-135067 5:02:01
CCTCGAGGCCCGCCGCGACGGCCCGCC
135086 135111 132535 136761 CDS product transcriptional regulator ICP4 135086-135111 5:02:01
GGCCGGGGCCAGGGGCCCGGCGCCGG
135088 135139 132535 136761 CDS product transcriptional regulator ICP4 135088-135139 9:06:02
CCGGGGCCAGGGGCCCGGCGCCGGCCAAAAGCCGGCGCGCCGCCGGGTCCCC
135153 135168 132535 136761 CDS product transcriptional regulator ICP4 135153-135168 4:01:01 CCGCCGCTGCCCGCCC
135179 135206 132535 136761 CDS product transcriptional regulator ICP4 135179-135206 6:03:01
CCCCATGCCGCCCGGCGGCCCCGCCGCC
135218 135233 132535 136761 CDS product transcriptional regulator ICP4 135218-135233 4:01:01 CCGCCGCGTGCCGCCC
135242 135263 132535 136761 CDS product transcriptional regulator ICP4 135242-135263 4:01:01
CCACACGCCCGCGCCCAGCGCC
135293 135312 132535 136761 CDS product transcriptional regulator ICP4 135293-135312 4:01:01
GGTCGCGGCGCGGCTCGCGG
135314 135343 132535 136761 CDS product transcriptional regulator ICP4 135314-135343 5:02:01
CCACCCGCTCTTCCCCGAGCCCTGGCGCCC
135436 135452 132535 136761 CDS product transcriptional regulator ICP4 135436-135452 4:01:01

GGCGGCGCGTGGCCTGG
135513 135528 132535 136761 CDS product transcriptional regulator ICP4 135513-135528 4:01:01 GGGGAGGCGCTGGCGG
135551 135571 132535 136761 CDS product transcriptional regulator ICP4 135551-135571 4:01:01
CCGGCGGCCTGAGTGGCCGCC
135553 135584 132535 136761 CDS product transcriptional regulator ICP4 135553-135584 6:03:01
GGCGGCCTGAGTGGCCGCCGGTGCGCGGCGGG
135646 135672 132535 136761 CDS product transcriptional regulator ICP4 135646-135672 5:02:01
GGGCCGGGTCCTGGACGGGCGCGCCGG
135760 135782 132535 136761 CDS product transcriptional regulator ICP4 135760-135782 6:03:01
GGCTGGCCGCGGCGCGGCGGCGG
135828 135846 132535 136761 CDS product transcriptional regulator ICP4 135828-135846 4:01:01
GGCCCGGCCGTCGGGCAGG
136057 136096 132535 136761 CDS product transcriptional regulator ICP4 136057-136096 7:04:01
CCACGCGCCTGGGCCCGCGCACCGCGGTGCCCGTGCCGCC
136126 136149 132535 136761 CDS product transcriptional regulator ICP4 136126-136149 4:01:01
GGCTCGACGGGCGCAAGGACATGG
136163 136206 132535 136761 CDS product transcriptional regulator ICP4 136163-136206 8:05:02
GGCGCTGGGGCTCGGGGAGCCGGACTTCGCGGAGGGCGAGGCGG
136207 136227 132535 136761 CDS product transcriptional regulator ICP4 136207-136227 6:03:01
CCAGCCACCGCGCCGCCAACC
136245 136268 132535 136761 CDS product transcriptional regulator ICP4 136245-136268 4:01:01
CCGCTGCGGCCGGTGTACCTGGCC
136252 136277 132535 136761 CDS product transcriptional regulator ICP4 136252-136277 5:02:01

GGCCGGTGTACCTGGCCTGCGGGCGG
136310 136323 132535 136761 CDS product transcriptional regulator ICP4 136310-136323 4:01:01 GGCCGCGGCGGTGG
136350 136369 132535 136761 CDS product transcriptional regulator ICP4 136350-136369 4:01:01
CCGCACGCCGAGGCGCCGCC
136373 136387 132535 136761 CDS product transcriptional regulator ICP4 136373-136387 4:01:01 GGTGCTGGAGGCGGG
136390 136430 132535 136761 CDS product transcriptional regulator ICP4 136390-136430 9:06:02
CCGCCCCCGCCGCCGCCGCGGCCGCGGCGCCCGGCGTGGCC
136409 136491 132535 136761 CDS product transcriptional regulator ICP4 136409-136491 14:11:03
GGCCGCGGCGCCCGGCGTGGCCTGGGACGCGGACCAGGGCCCGCGGGAGACGCTGGTGCAGGCGCGGCGCGGCGGCGTCCCGG
136519 136537 132535 136761 CDS product transcriptional regulator ICP4 136519-136537 4:01:01
CCCCGCCGGCCTGGGCGCC
136553 136603 132535 136761 CDS product transcriptional regulator ICP4 136553-136603 10:07:02
GGGCACGGACTCGGGCTTCCCGGGGCCGGCGACGGCGGCATCGGCGGGGGG
136605 136633 132535 136761 CDS product transcriptional regulator ICP4 136605-136633 6:03:01
CCGCGCCGCGCCCGGCCCCGGCCCCGGCC
136618 136656 132535 136761 CDS product transcriptional regulator ICP4 136618-136656 7:04:01
GGCCCCGGCCCCGGCCGCGGCGCGGGCCGGCGCGCGTGG
136666 136706 132535 136761 CDS product transcriptional regulator ICP4 136666-136706 8:05:02
CCTCGTCCGCGTCCTCCGCGTCCTCCTCGTCGTCCGCGTCC

gi|51557483|ref|NC_006151.1| Suid herpesvirus 1, complete genome
1651 1674 1636 2259 CDS product membrane protein UL56 1651-1674 5:02:01 CCCGCGGGGCCCCGCCGCGCCGCC
1685 1709 1636 2259 CDS product membrane protein UL56 1685-1709 5:02:01 CCCGCCCGATCCCGGCAGCCTCGCC

1725 1744 1636 2259 CDS product membrane protein UL56 1725-1744 6:03:01 GGGGGGAGGAGCGGCGGAGG
1783 1799 1636 2259 CDS product membrane protein UL56 1783-1799 4:01:01 CCGTCGCCTCGGCCCCC
1820 1846 1636 2259 CDS product membrane protein UL56 1820-1846 5:02:01 GGAGGCTCTCGGACTCGGCGCCCCCGG
1840 1862 1636 2259 CDS product membrane protein UL56 1840-1862 6:03:01 CCCCCGGCTCCAGACCGCCCTCC
1882 1915 1636 2259 CDS product membrane protein UL56 1882-1915 6:03:01
CCGGGCCGCGCCCGCACCGATCGCCGGACACGCC
1924 1943 1636 2259 CDS product membrane protein UL56 1924-1943 5:02:01 CCCGGGGCCCGCCCCCGTCC
1987 2003 1636 2259 CDS product membrane protein UL56 1987-2003 4:01:01 CCATCCCGGACCCGCCC
2028 2046 1636 2259 CDS product membrane protein UL56 2028-2046 4:01:01 CCGCAGCCGCCGAGGCCCC
2093 2113 1636 2259 CDS product membrane protein UL56 2093-2113 4:01:01 CCCCTTCAGCCAGTGCCTCCC
2122 2155 1636 2259 CDS product membrane protein UL56 2122-2155 6:03:01
CCTGCCCCGACTGCCGCTACCCCGAAGACCGCCC
2800 2812 2730 3815 CDS product multifunctional expression regulator 2800-2812 4:01:01
GGCGGCAGGTGGG
2839 2860 2730 3815 CDS product multifunctional expression regulator 2839-2860 4:01:01
CCAGCGCGTCCATGATCCCGCC
3100 3116 2730 3815 CDS product multifunctional expression regulator 3100-3116 4:01:01
CCGCCCCCGCGGTGGCC
3283 3305 2730 3815 CDS product multifunctional expression regulator 3283-3305 5:02:01
GGCGGTGCAGGTCGGCCCCGTGG
3370 3385 2730 3815 CDS product multifunctional expression regulator 3370-3385 4:01:01
GGACGGCGGCCCACGG
3548 3582 2730 3815 CDS product multifunctional expression regulator 3548-3582 8:05:02

CCCGCCGTCCGGACGATCCGCCTCCTGTCGCCGCC
3620 3641 2730 3815 CDS product multifunctional expression regulator 3620-3641 5:02:01
CCGCCGCCGCTGCCGCAGGGCC
3656 3714 2730 3815 CDS product multifunctional expression regulator 3656-3714 12:09:03
CCCGCCGCGACCCCCGCGCCGGCGCCGGATGGCCCCCAGCCGCGCCCGGACGGGCCGCC
3748 3790 2730 3815 CDS product multifunctional expression regulator 3748-3790 7:04:01
CCGGGCGCCGCTCCTCGCTCCCCGACCTGGACGCCTCGCTGCC
4100 4128 3895 4833 CDS product envelope glycoprotein K 4100-4128 6:03:01
CCGCCAGGAGCTCCAGCGCCCCGACCGCC
4296 4317 3895 4833 CDS product envelope glycoprotein K 4296-4317 4:01:01 GGGGTCCTCGCGGAGGTCGCGG
4435 4467 3895 4833 CDS product envelope glycoprotein K 4435-4467 6:03:01
GGGGTCAGGAACTCGGCGGGGTCGCGGAAGGGG
4556 4575 3895 4833 CDS product envelope glycoprotein K 4556-4575 4:01:01 GGTAGGCGCGGCAGTCGCGG
4667 4688 3895 4833 CDS product envelope glycoprotein K 4667-4688 5:02:01 CCTCCCAGTCCGGCGCGCCCCC
4728 4750 3895 4833 CDS product envelope glycoprotein K 4728-4750 5:02:01 GGGGTGGGGCAGGCGGGCGACGG
4796 4815 3895 4833 CDS product envelope glycoprotein K 4796-4815 4:01:01 GGACGAGGAGGTGCAGCGGG
4945 4970 4788 7676 CDS product helicase-primase primase subunit 4945-4970 5:02:01
GGGCCACGAAGGTGCGGGCCGGCGGG
5128 5153 4788 7676 CDS product helicase-primase primase subunit 5128-5153 5:02:01
CCATGGCCACGTCCGCCACGAAGGCC
5165 5191 4788 7676 CDS product helicase-primase primase subunit 5165-5191 6:03:01
CCGGTCCTCCGCGTCCACGGCCAGGCC
5390 5414 4788 7676 CDS product helicase-primase primase subunit 5390-5414 5:02:01

CCCGTGGCAGCCGCCGCCGTCGACC

| | | | | | | |
|---|---|---|---|---|---|---|
| 5485 | 5503 | 4788 | 7676 | CDS product helicase-primase primase subunit | 5485-5503 | 4:01:01 |

GGAGGTACGGGCCCATGGG

| | | | | | | |
|---|---|---|---|---|---|---|
| 5661 | 5696 | 4788 | 7676 | CDS product helicase-primase primase subunit | 5661-5696 | 8:05:02 |

GGGACGGGGGCGCGGACGAGGGCGGAGGCGCCGGGG

| | | | | | | |
|---|---|---|---|---|---|---|
| 5691 | 5729 | 4788 | 7676 | CDS product helicase-primase primase subunit | 5691-5729 | 8:05:02 |

CCGGGGTCCCCGCCCGCGCCCCCGATCCCGACCCCGACC

| | | | | | | |
|---|---|---|---|---|---|---|
| 5766 | 5798 | 4788 | 7676 | CDS product helicase-primase primase subunit | 5766-5798 | 6:03:01 |

CCCGCGTCCCAGTCCGCCCAGTCCTCGTCGCCC

| | | | | | | |
|---|---|---|---|---|---|---|
| 5836 | 5857 | 4788 | 7676 | CDS product helicase-primase primase subunit | 5836-5857 | 4:01:01 |

CCCAGGGCGCCTCCTCGAGGCC

| | | | | | | |
|---|---|---|---|---|---|---|
| 5864 | 5904 | 4788 | 7676 | CDS product helicase-primase primase subunit | 5864-5904 | 10:07:02 |

GGAGGAGGCCGCGGAGGCGGACCCCGCGGCGGCGGCGAGGG

| | | | | | | |
|---|---|---|---|---|---|---|
| 6197 | 6251 | 4788 | 7676 | CDS product helicase-primase primase subunit | 6197-6251 | 9:06:02 |

CCCCCAGGCGTCCCCGGCCAGCACCGCGAAGGCCTGGCGCCGGTGCGCCAGCTCC

| | | | | | | |
|---|---|---|---|---|---|---|
| 6256 | 6275 | 4788 | 7676 | CDS product helicase-primase primase subunit | 6256-6275 | 4:01:01 |

GGTACACCGGCGCCGGGGGG

| | | | | | | |
|---|---|---|---|---|---|---|
| 6355 | 6385 | 4788 | 7676 | CDS product helicase-primase primase subunit | 6355-6385 | 6:03:01 |

GGCGGCGCAGGACGGCGCCGTCGGCGCCCGG

| | | | | | | |
|---|---|---|---|---|---|---|
| 6446 | 6474 | 4788 | 7676 | CDS product helicase-primase primase subunit | 6446-6474 | 6:03:01 |

GGCGGTCACGCGGCGCGCGAGGCCGGCGG

| | | | | | | |
|---|---|---|---|---|---|---|
| 6529 | 6554 | 4788 | 7676 | CDS product helicase-primase primase subunit | 6529-6554 | 5:02:01 |

CCTCCACGTAGCCGGCGCCCATGGCC

6542 6563 4788 7676 CDS product helicase-primase primase subunit 6542-6563 4:01:01
GGCGCCCATGGCCGGGGCGAGG
6628 6645 4788 7676 CDS product helicase-primase primase subunit 6628-6645 5:02:01
GGCGGAAGAAGGCGGCGG
6781 6813 4788 7676 CDS product helicase-primase primase subunit 6781-6813 5:02:01
GGTCGGGGAGCTTGAGGCTGCCCCGGTCGTGGG
6962 6986 4788 7676 CDS product helicase-primase primase subunit 6962-6986 4:01:01
GGGGTCCAGGCCGCTGGGGTTGGGG
7015 7074 4788 7676 CDS product helicase-primase primase subunit 7015-7074 11:08:02
GGTCCTTGAGGGCCCGCAGGTCGTAGGCCGGCCGCGGGTGGAAGAGGTGGAGGCGCGTGG
7147 7161 4788 7676 CDS product helicase-primase primase subunit 7147-7161 4:01:01 GGCGCCGGAAGGCGG
7309 7332 4788 7676 CDS product helicase-primase primase subunit 7309-7332 5:02:01
GGCCGTCGTGGAGGGCGAAGGTGG
7333 7349 4788 7676 CDS product helicase-primase primase subunit 7333-7349 4:01:01
CCGCCTCGTCCATGGCC
7683 7713 7663 8373 CDS product tegument protein UL51 7683-7713 6:03:01
GGGTGGTGCGGGCGCCGCGGCCGTGGGAGGG
7772 7793 7663 8373 CDS product tegument protein UL51 7772-7793 5:02:01 CCTCCCCGCCCCGCTCGCCGCC
7796 7818 7663 8373 CDS product tegument protein UL51 7796-7818 4:01:01 GGACGTGATGGCCTCGGCCGAGG
7807 7836 7663 8373 CDS product tegument protein UL51 7807-7836 6:03:01
CCTCGGCCGAGGCCGCGCGCCGCCTGGCCC
7858 7910 7663 8373 CDS product tegument protein UL51 7858-7910 9:06:02
CCTACCAGGCCTGCCAGCGCAACCTGGAGTGCCTGGCGCGCCACGAGGCCTCC

7904 7950 7663 8373 CDS product tegument protein UL51 7904-7950 7:04:01
GGCCTCCGGCGACGACGGCCTGGACGCCGTGGTCGCGGCGCACGCGG

8096 8142 7663 8373 CDS product tegument protein UL51 8096-8142 9:06:02
GGCCGTCCTGGAGCGGACGCTGGGACTGGCCCGGCGGCGGCAGCCGG

8139 8165 7663 8373 CDS product tegument protein UL51 8139-8165 6:03:01 CCGGCGCCCGTCCGGGCCGCGCCCGCC

8178 8200 7663 8373 CDS product tegument protein UL51 8178-8200 5:02:01 CCCGTCCCCGCCCAGCCCGCCCC

8209 8240 7663 8373 CDS product tegument protein UL51 8209-8240 9:06:02
CCGCCGCCCGCCCGGCCCCGCCGCCCCCGCCC

8243 8268 7663 8373 CDS product tegument protein UL51 8243-8268 7:04:01 GGAGGCGGGGGAAGACGAGGAGGGGG

8290 8318 7663 8373 CDS product tegument protein UL51 8290-8318 6:03:01
CCCCGCTGCTGCCCCCCCGCGCCCCCGCC

8339 8358 7663 8373 CDS product tegument protein UL51 8339-8358 5:02:01 GGCCCGGAGGCTGGCCGAGG

8620 8647 8527 9333 CDS product deoxyuridine triphosphatase 8620-8647 5:02:01
GGGGCGCGCGGGGTGGCGGGGAACGGGG

8649 8667 8527 9333 CDS product deoxyuridine triphosphatase 8649-8667 4:01:01 CCGTCCCGTGATCCAGCCC

8738 8763 8527 9333 CDS product deoxyuridine triphosphatase 8738-8763 5:02:01
GGTTCTGGATCCGGAAGCGGCACGGG

8874 8894 8527 9333 CDS product deoxyuridine triphosphatase 8874-8894 4:01:01 GGTCTCGGCGCCCCCGGGGGG

9053 9089 8527 9333 CDS product deoxyuridine triphosphatase 9053-9089 6:03:01
CCTGCACCTCGCCGCGAAAGCCCGCGTCCACGATCCC

9149 9171 8527 9333 CDS product deoxyuridine triphosphatase 9149-9171 4:01:01 CCACCTTGAGGCCCAGGGGAACC

9181 9225 8527 9333 CDS product deoxyuridine triphosphatase 9181-9225 9:06:02
CCCTCCGGCCCCGGACTCCGGCACACCAGCAGCCGCCCTCCGTCC

9329 9356 9257 9553 CDS product envelope glycoprotein N 9329-9356 5:02:01 CCATCGTCTCCACCGAGGGGCCGCTGCC

9395 9408 9257 9553 CDS product envelope glycoprotein N 9395-9408 4:01:01 CCGCCTGCGCCGCC

9430 9444 9257 9553 CDS product envelope glycoprotein N 9430-9444 4:01:01 CCCACGGCCGCCGCC

9609 9634 9591 10340 CDS product tegument protein VP22 9609-9634 5:02:01 CCCGGGTCGCCGCCGACGAGACCGCC

9646 9718 9591 10340 CDS product tegument protein VP22 9646-9718 14:11:03 CCGCCGCGCCGGCAGCGCCTCTCGCACCCGGACCACCGCCCCGGCCGCCGCGACCCCCAGACGCCCCTCGGCC

9776 9801 9591 10340 CDS product tegument protein VP22 9776-9801 4:01:01 GGCTACTACGGCTACGATGGCTACGG

9804 9826 9591 10340 CDS product tegument protein VP22 9804-9826 6:03:01 CCTCCCGCGCCCCCCGCGCCGCC

9836 9997 9591 10340 CDS product tegument protein VP22 9836-9997 34:31:08 CCCGCGGCCGCCTCGCGGGCCTCGACCGGGGCCAAGAGCGCCTCGGCCGCCAAGACCCCCGCGTCCGCGGCCAAGACCGCCCGCTCGGCCCCGGCCGCCGCCCCGGCCGCCACCACCACCACCACCAC CACCGCCGCCGCGGAACCGGCCGCCCGGCGCGCC

10036 10051 9591 10340 CDS product tegument protein VP22 10036-10051 4:01:01 CCGCCGCCGGCTCGCC

10129 10151 9591 10340 CDS product tegument protein VP22 10129-10151 4:01:01 GGCCGTGGGACGCGTGGCCGAGG

10146 10178 9591 10340 CDS product tegument protein VP22 10146-10178 7:04:01 CCGAGGCCCACGCCCGCGCCGCCGCCGAGTCCC

10438 10468 10404 11645 CDS product transactivating tegument protein VP16 10438-10468 6:03:01 GGCCCTGCTGGGGGGCGCGGCCCCGGGCCGG

10458 10488 10404 11645 CDS product transactivating tegument protein VP16 10458-10488 6:03:01 CCCCGGGCCGGCCGCCCGTGGTGGCCGTCCC

10549 10589 10404 11645 CDS product transactivating tegument protein VP16 10549-10589 6:03:01 GGGCCCGGCGCTGCTGGGCGCCATGGAGCGCTGGAACGAGG

10648 10694 10404 11645 CDS product transactivating tegument protein VP16 10648-10694 9:06:02 GGCCTCCGCGGACGAGGTGGCGGCCCAGGTCCGGGCGCCGACGGCGG

10711 10727 10404 11645 CDS product transactivating tegument protein VP16 10711-10727 4:01:01 GGGGGTGCCCGGGGCGG

10730 10750 10404 11645 CDS product transactivating tegument protein VP16 10730-10750 4:01:01 CCCATGCCGCGAGTCCCGGCC

10747 10760 10404 11645 CDS product transactivating tegument protein VP16 10747-10760 4:01:01 GGCCGTGGAGGAGG

11020 11039 10404 11645 CDS product transactivating tegument protein VP16 11020-11039 5:02:01 GGACCAGGTGGTGGCCCAGG

11169 11183 10404 11645 CDS product transactivating tegument protein VP16 11169-11183 4:01:01 CCGTCAACCACCGCC

11255 11303 10404 11645 CDS product transactivating tegument protein VP16 11255-11303 9:06:02 CCGCCGTTCTCCGCCGCGCTGCCGCGCGCCGCCGGCTACCTCTCGCACC

11359 11380 10404 11645 CDS product transactivating tegument protein VP16 11359-11380 5:02:01 CCGCCCGCCCTCGCCCGTGGCC

11394 11457 10404 11645 CDS product transactivating tegument protein VP16 11394-11457 11:08:02 CCGCGGAGGCCCTGCTGCCGCCCCCGTCGCCCTCGGCCGTGCTGCCCTGCGACCCGACGCCGCC

11506 11520 10404 11645 CDS product transactivating tegument protein VP16 11506-11520 4:01:01 GGCCGAGGAGGATGG

11533 11552 10404 11645 CDS product transactivating tegument protein VP16 11533-11552 4:01:01 GGCGGCGCCCGCGGCCGCGG

11571 11612 10404 11645 CDS product transactivating tegument protein VP16 11571-11612 11:08:02 CCTCGCCCGGGCCCGCCACCCCCGCCTACCACCTCATCCCCC

11798 11811 11746 13998 CDS product tegument protein VP13/14 11798-11811 4:01:01 CCAGCCGTTCCCCC

11849 11868 11746 13998 CDS product tegument protein VP13/14 11849-11868 4:01:01 GGGCGAGGTGCGGCGCCCGG
11864 11885 11746 13998 CDS product tegument protein VP13/14 11864-11885 5:02:01 CCCGGACTTCCGCCCGCCGCCC
11933 12000 11746 13998 CDS product tegument protein VP13/14 11933-12000 18:15:04
GGAGGAGGAAGAGGGGGGGGCGGAGGAGGAGAGGACGAGCGGGGGCCGGCGCGGGGGTGAGGAGGGGG
12009 12069 11746 13998 CDS product tegument protein VP13/14 12009-12069 13:10:03
GGGGACGAGGAGGAGGAGGAGGAGGAGGAGAGCGAGGGGGGCGCGTGGTCCGACGGGG
12100 12144 11746 13998 CDS product tegument protein VP13/14 12100-12144 9:06:02
GGGACGCGGAAGAGGAGGAGGACGAGGATGGGGACGAGGATGGGG
12171 12195 11746 13998 CDS product tegument protein VP13/14 12171-12195 4:01:01
GGCTATGACGGCCGCGGCGCCGCGG
12182 12224 11746 13998 CDS product tegument protein VP13/14 12182-12224 9:06:02
CCGCGGCGCCGCGGCCTCCCGCGCCGCGGCCGGGCCCCCCGCC
12255 12274 11746 13998 CDS product tegument protein VP13/14 12255-12274 4:01:01 GGCATGGAGACGGCCTCCGG
12336 12409 11746 13998 CDS product tegument protein VP13/14 12336-12409 16:13:04
CCCGGGCGGCCGGGGTACCGGCCCCCGCGCCCGCGCGACCCCCCGCCGCCCCCGCCCCCGCGGGCCCGGCCACC
12339 12356 11746 13998 CDS product tegument protein VP13/14 12339-12356 4:01:01 GGGCGGCCGGGGTACCGG
12424 12447 11746 13998 CDS product tegument protein VP13/14 12424-12447 8:05:02 CCGCCGCCTCCACCGCCGCCGCCC
12456 12470 11746 13998 CDS product tegument protein VP13/14 12456-12470 4:01:01 CCCGCCCGCGCCGCC
12543 12568 11746 13998 CDS product tegument protein VP13/14 12543-12568 5:02:01
CCCGACGGGACCTTCCTGGCCATGCC
12587 12649 11746 13998 CDS product tegument protein VP13/14 12587-12649 11:08:02
CCGCGAGCCGCGCGGGCCCCCGGGCCGCCCCGTCGGGCCCCCGGACATCCTGCGCCGCGCCCC
12674 12687 11746 13998 CDS product tegument protein VP13/14 12674-12687 4:01:01 GGCGGCCTCGGCGG

| | | | | | |
|---|---|---|---|---|---|
| 12707 | 12726 | 11746 | 13998 | CDS product tegument protein VP13/14 | 12707-12726 4:01:01 GGACGCCTGGGAGGCGCTGG |
| 12729 | 12744 | 11746 | 13998 | CDS product tegument protein VP13/14 | 12729-12744 4:01:01 CCGCTGCACCACCTCC |
| 12847 | 12866 | 11746 | 13998 | CDS product tegument protein VP13/14 | 12847-12866 4:01:01 CCATCGCCCTGCACCACGCC |
| 13038 | 13076 | 11746 | 13998 | CDS product tegument protein VP13/14 | 13038-13076 7:04:01 CCCGCGGCCTCGGGGCCGCGCGCCGGCCTCTTCCGCGCC |
| 13083 | 13104 | 11746 | 13998 | CDS product tegument protein VP13/14 | 13083-13104 4:01:01 GGCTCCCTGGCCTACTGGCCGG |
| 13150 | 13163 | 11746 | 13998 | CDS product tegument protein VP13/14 | 13150-13163 4:01:01 CCTTCCACCTGGCC |
| 13436 | 13446 | 11746 | 13998 | CDS product tegument protein VP13/14 | 13436-13446 4:01:01 GGTGGCGGCGG |
| 13456 | 13475 | 11746 | 13998 | CDS product tegument protein VP13/14 | 13456-13475 4:01:01 CCACGGGCCTGGGCCAGGCC |
| 13620 | 13647 | 11746 | 13998 | CDS product tegument protein VP13/14 | 13620-13647 6:03:01 GGCGCCGCGGTCCACGGCGGCCGGACGG |
| 13771 | 13789 | 11746 | 13998 | CDS product tegument protein VP13/14 | 13771-13789 4:01:01 GGTTGACGGCGGCCCCGGG |
| 13824 | 13845 | 11746 | 13998 | CDS product tegument protein VP13/14 | 13824-13845 4:01:01 CCCGCGCTGCCCTCCGAGGACC |
| 13859 | 13884 | 11746 | 13998 | CDS product tegument protein VP13/14 | 13859-13884 5:02:01 CCCGCCCACGGCCGTGCGCCCCGCCC |
| 13904 | 13930 | 11746 | 13998 | CDS product tegument protein VP13/14 | 13904-13930 5:02:01 CCTCGCCGAATACGCCGCGCGCCACCC |
| 14108 | 14145 | 14017 | 16098 | CDS product tegument protein VP11/12 | 14108-14145 8:05:02 GGCGGCCCCCGGGGAGGTGCTGACGGCGGCCGTGGCGG |
| 14315 | 14352 | 14017 | 16098 | CDS product tegument protein VP11/12 | 14315-14352 7:04:01 GGGCATCACGGCGGACGTGGCCTGGCGCGCGGTGCTGG |
| 14375 | 14403 | 14017 | 16098 | CDS product tegument protein VP11/12 | 14375-14403 5:02:01 GGCCAGCTCGGGCGTGGAGGTGCGCTCGG |

14509 14543 14017 16098 CDS product tegument protein VP11/12 14509-14543 5:02:01
CCACGCGCGCCGCGCTCGCCGAGGCGACCGAGGCC
14531 14552 14017 16098 CDS product tegument protein VP11/12 14531-14552 4:01:01 GGCGACCGAGGCCGTGGAGCGG
14570 14598 14017 16098 CDS product tegument protein VP11/12 14570-14598 5:02:01
CCCGCTCGACCCCATGGTCTCCAGCCCCC
14653 14676 14017 16098 CDS product tegument protein VP11/12 14653-14676 4:01:01 GGCTGCTGTGGATGATGGACACGG
14771 14795 14017 16098 CDS product tegument protein VP11/12 14771-14795 5:02:01
CCGCGCCGGCGGGCCCGCCGTGGCC
14792 14811 14017 16098 CDS product tegument protein VP11/12 14792-14811 4:01:01 GGCCTCGGGCTCGGGCGAGG
14851 14888 14017 16098 CDS product tegument protein VP11/12 14851-14888 5:02:01
CCCTGGAGATCCTCGGCGCCACGTGGGCCGAGACGCCC
14890 14916 14017 16098 CDS product tegument protein VP11/12 14890-14916 4:01:01
GGAAGAGCGGCGTGTGCGGCCTCACGG
14909 14948 14017 16098 CDS product tegument protein VP11/12 14909-14948 7:04:01
CCTCACGGCCGCCGTCGTGGCCGCCGTGGACCTCGTGACC
15062 15075 14017 16098 CDS product tegument protein VP11/12 15062-15075 4:01:01 CCGCTTCCACCACC
15256 15286 14017 16098 CDS product tegument protein VP11/12 15256-15286 7:04:01
CCCCCGCGCAGCCGCCGCTGCCGCCGCTGCC
15303 15324 14017 16098 CDS product tegument protein VP11/12 15303-15324 5:02:01 CCGTCGCCGCCGCCCCGCGACC
15381 15421 14017 16098 CDS product tegument protein VP11/12 15381-15421 8:05:02
CCCGTGCCCGTCTCGCCGCCCCCGCTCCCCGAGGGGCCCCC
15440 15461 14017 16098 CDS product tegument protein VP11/12 15440-15461 7:04:01 GGAGGTGGACGGCGGCGGGCGG
15464 15491 14017 16098 CDS product tegument protein VP11/12 15464-15491 6:03:01

CCCCCTCCGGCGCAGCCGCGACGCCGCC
15503 15523 14017 16098 CDS product tegument protein VP11/12 15503-15523 4:01:01 CCGCAAGGACCTCGCGCCCCC
15530 15550 14017 16098 CDS product tegument protein VP11/12 15530-15550 7:04:01 GGCGGCGGGGGAGGGGGAGGG
15560 15581 14017 16098 CDS product tegument protein VP11/12 15560-15581 5:02:01 GGAAGAGGAGGAGGATGACCGG
15701 15714 14017 16098 CDS product tegument protein VP11/12 15701-15714 5:02:01 GGGGGTGGAGGAGG
15738 15766 14017 16098 CDS product tegument protein VP11/12 15738-15766 6:03:01
GGGGACGGCGAACGAGGCGCCGGCGGCGG
15931 15963 14017 16098 CDS product tegument protein VP11/12 15931-15963 8:05:02
CCGCGGCCCCCGCCACCCCCGCGGTCCCCGCCC
15973 16008 14017 16098 CDS product tegument protein VP11/12 15973-16008 7:04:01
CCGACCACGTGTACCAGCACCCCCGCCCGCGGACCC
16022 16043 14017 16098 CDS product tegument protein VP11/12 16022-16043 5:02:01 CCTGTACCAGCACCCCCGACCC
16052 16065 14017 16098 CDS product tegument protein VP11/12 16052-16065 4:01:01 CCTCACCGGCCACC
16882 16908 16854 19595 CDS product envelope glycoprotein B 16882-16908 5:02:01 GGCGCTGGTAGTGCCGGCGGCGCGTGG
17017 17053 16854 19595 CDS product envelope glycoprotein B 17017-17053 6:03:01
CCCGGGCCTGGTCCAGCTTGGCCTCGTCCACGTCGCC
17093 17126 16854 19595 CDS product envelope glycoprotein B 17093-17126 5:02:01
GGGGTACAGGGCCTTCATGGGGTTGCGGCGCAGG
17138 17185 16854 19595 CDS product envelope glycoprotein B 17138-17185 8:05:02
CCGGTAGGCCAGGAAGGCCGCGACCAGGCCGGCCAGCACCAGCAGCCC
17140 17154 16854 19595 CDS product envelope glycoprotein B 17140-17154 4:01:01 GGTAGGCCAGGAAGG
17204 17220 16854 19595 CDS product envelope glycoprotein B 17204-17220 4:01:01 GGGGTTGGACAGGAAGG
17224 17240 16854 19595 CDS product envelope glycoprotein B 17224-17240 4:01:01 CCATGCCGCCGACGGCC

| | | | | | | |
|---|---|---|---|---|---|---|
| 17251 | 17278 | 16854 | 19595 | CDS product envelope glycoprotein B | 17251-17278 6:03:01 | CCCCCGTGGCACCCAGGACCACCTTGCC |
| 17608 | 17626 | 16854 | 19595 | CDS product envelope glycoprotein B | 17608-17626 4:01:01 | GGTGGTTGCCGGTGCAGGG |
| 17778 | 17799 | 16854 | 19595 | CDS product envelope glycoprotein B | 17778-17799 5:02:01 | CCGCCGCGCACCTCCACGCACC |
| 17855 | 17882 | 16854 | 19595 | CDS product envelope glycoprotein B | 17855-17882 5:02:01 | GGCCGTGGCCACGGCGCTGGGGTTCAGG |
| 18053 | 18067 | 16854 | 19595 | CDS product envelope glycoprotein B | 18053-18067 4:01:01 | GGCCGGCGGCTCGGG |
| 18078 | 18125 | 16854 | 19595 | CDS product envelope glycoprotein B | 18078-18125 9:06:02 | GGGCCGGGGGAGCGCCGGGCCCGACGGGCGGCCGCGGGGGCCGCGGGG |
| 18109 | 18145 | 16854 | 19595 | CDS product envelope glycoprotein B | 18109-18145 5:02:01 | CCGCGGGGGCCGCGGGGCCCACGACGCCGGCGAGGCC |
| 18208 | 18223 | 16854 | 19595 | CDS product envelope glycoprotein B | 18208-18223 4:01:01 | CCACCACGAAGCCCCC |
| 18238 | 18257 | 16854 | 19595 | CDS product envelope glycoprotein B | 18238-18257 4:01:01 | CCTCGGGCCTGTCGCCGGCC |
| 18286 | 18323 | 16854 | 19595 | CDS product envelope glycoprotein B | 18286-18323 6:03:01 | GGTAGATGGCGTCGATGGCCTCCGAGGCCTCGCGGAGG |
| 18445 | 18467 | 16854 | 19595 | CDS product envelope glycoprotein B | 18445-18467 4:01:01 | CCTCGGCCTCGCGCCACTTGGCC |
| 18496 | 18512 | 16854 | 19595 | CDS product envelope glycoprotein B | 18496-18512 4:01:01 | CCCAGTCCCAGGCCACC |
| 18554 | 18566 | 16854 | 19595 | CDS product envelope glycoprotein B | 18554-18566 4:01:01 | GGAGGCGCGGAGG |
| 18695 | 18726 | 16854 | 19595 | CDS product envelope glycoprotein B | 18695-18726 5:02:01 | GGACAGGGCGAAGGAGTCGTAGGGGTACACGG |
| 18733 | 18743 | 16854 | 19595 | CDS product envelope glycoprotein B | 18733-18743 4:01:01 | CCTCCACCTCC |
| 18800 | 18819 | 16854 | 19595 | CDS product envelope glycoprotein B | 18800-18819 4:01:01 | GGTGTAGGTGTCGTTGGTGG |
| 19183 | 19204 | 16854 | 19595 | CDS product envelope glycoprotein B | 19183-19204 5:02:01 | CCAGCCGCACCACCGTGGAGCC |
| 19199 | 19217 | 16854 | 19595 | CDS product envelope glycoprotein B | 19199-19217 5:02:01 | GGAGCCGGACGGCGGCGGG |
| 19261 | 19291 | 16854 | 19595 | CDS product envelope glycoprotein B | 19261-19291 5:02:01 | |

CCGTCCGCGCGTCCAGGTCGCCGTACTCGCC

19295 19317 16854 19595 CDS product envelope glycoprotein B 19295-19317 5:02:01 GGGGGCCTCCGAGGGGCCGGGGG

19382 19404 16854 19595 CDS product envelope glycoprotein B 19382-19404 5:02:01 GGGCGTCGGCGAGGCCGAGGCGG

19521 19585 19466 21640 CDS product DNA packaging terminase subunit 2 19521-19585 13:10:03 CCGAGGCCAGCACCGCCGTGGTGCCCGGGCCGATGCCCCCGGGGCCCGCGCCAAAGACCGCCACC

19737 19768 19466 21640 CDS product DNA packaging terminase subunit 2 19737-19768 6:03:01 GGGTGGCGGTTACGCCGGCCGTCTGGAAGCGG

19836 19849 19466 21640 CDS product DNA packaging terminase subunit 2 19836-19849 4:01:01 GGTGCGGCAGGAGG

19885 19901 19466 21640 CDS product DNA packaging terminase subunit 2 19885-19901 4:01:01 GGGGGGCTGGGCGAAGG

19960 19990 19466 21640 CDS product DNA packaging terminase subunit 2 19960-19990 6:03:01 GGCCGGAAGGAGCGCGGCGTCCACGGGGTGG

20104 20119 19466 21640 CDS product DNA packaging terminase subunit 2 20104-20119 4:01:01 CCCGGCGGCCACCGCC

20205 20225 19466 21640 CDS product DNA packaging terminase subunit 2 20205-20225 4:01:01 CCAGGCTCGCCGCCGAGCGCC

20277 20314 19466 21640 CDS product DNA packaging terminase subunit 2 20277-20314 7:04:01 CCGCGGCCGGCAGGTCCGCCCACGCCACGGCGTCCGCC

20315 20331 19466 21640 CDS product DNA packaging terminase subunit 2 20315-20331 4:01:01 GGCGGGGGCGCGCCGGG

20345 20388 19466 21640 CDS product DNA packaging terminase subunit 2 20345-20388 9:06:02 CCGACGCCCCCGTCGCCGGCGTCCGCGCCCCCCGCGGCGCCGCC

20418 20455 19466 21640 CDS product DNA packaging terminase subunit 2 20418-20455 7:04:01 GGAGGCGCTCGAGGGCGTCGCGGTTGGCCCGGTCCGGG

20473 20491 19466 21640 CDS product DNA packaging terminase subunit 2 20473-20491 4:01:01
GGGCGTGGACATGTGGTGG
20637 20656 19466 21640 CDS product DNA packaging terminase subunit 2 20637-20656 4:01:01
CCACGGCCGCCGCGCGCACC
20820 20902 19466 21640 CDS product DNA packaging terminase subunit 2 20820-20902 16:13:04
CCGCGCGCCGGCGCGCCGTCTCGCCCGCGTCCCCGGCCTCGGCGCCCGCCGCCGCCGTGGACGCCGCCTCCGCCTCCCGGGCC
20898 20921 19466 21640 CDS product DNA packaging terminase subunit 2 20898-20921 5:02:01
GGGCCTCGGCGGCGTCCACGGCGG
20988 21023 19466 21640 CDS product DNA packaging terminase subunit 2 20988-21023 6:03:01
CCACGTCCTCGGGGTCCACGCGCACCGCCAGCTGCC
21141 21165 19466 21640 CDS product DNA packaging terminase subunit 2 21141-21165 5:02:01
CCGGCGGCACCACGAGCCCGCCGCC
21189 21208 19466 21640 CDS product DNA packaging terminase subunit 2 21189-21208 4:01:01
CCACGGCCGACAGGTCCACC
21252 21271 19466 21640 CDS product DNA packaging terminase subunit 2 21252-21271 4:01:01
GGTTCTCGGCGTCGTGGAGG
21282 21300 19466 21640 CDS product DNA packaging terminase subunit 2 21282-21300 4:01:01
CCGTCTCCACGCGCCCGCC
21866 21895 21788 25315 CDS product single-stranded DNA-binding protein 21866-21895 5:02:01
CCCCCCAGCTCCGCGGGCACCAGCGAGGCC
21904 21936 21788 25315 CDS product single-stranded DNA-binding protein 21904-21936 5:02:01
CCCCGCGACGTCCCCGCCCGCGCCGCAGGCCCC
21954 21982 21788 25315 CDS product single-stranded DNA-binding protein 21954-21982 6:03:01

CCTCGCCGCCGTCCGCGCCCGTCTGGCCC
22008 22029 21788 25315 CDS product single-stranded DNA-binding protein 22008-22029 5:02:01
CCGCCTCCACGCTCCAGCGCCC
22059 22075 21788 25315 CDS product single-stranded DNA-binding protein 22059-22075 4:01:01
CCGCCAGCGCGGCCGCC
22167 22195 21788 25315 CDS product single-stranded DNA-binding protein 22167-22195 5:02:01
CCGTCTGCACCAGCGGCCCGCCGTCGGCC
22237 22252 21788 25315 CDS product single-stranded DNA-binding protein 22237-22252 4:01:01 CCCGGCGCCGCCGCCC
22340 22369 21788 25315 CDS product single-stranded DNA-binding protein 22340-22369 6:03:01
CCGCCGTTGAGCCCGCTCCAGTTCCCCGCC
22415 22432 21788 25315 CDS product single-stranded DNA-binding protein 22415-22432 4:01:01
CCCAGGCCCAGCACCACC
22436 22472 21788 25315 CDS product single-stranded DNA-binding protein 22436-22472 7:04:01
GGGCGCGAGGCCATGACGGAGCGGAGCAGGTGGCTGG
22536 22555 21788 25315 CDS product single-stranded DNA-binding protein 22536-22555 4:01:01
CCGCGTCGGCCGCGTCCGCC
22573 22600 21788 25315 CDS product single-stranded DNA-binding protein 22573-22600 5:02:01
GGCCATGACGGCGGCCGGGTCCCGCGGG
22668 22694 21788 25315 CDS product single-stranded DNA-binding protein 22668-22694 5:02:01
GGATCAGGTTGGCCAGGTAGAACTGGG
23237 23254 21788 25315 CDS product single-stranded DNA-binding protein 23237-23254 4:01:01
GGCTGGAACTGGTTGCGG
23295 23314 21788 25315 CDS product single-stranded DNA-binding protein 23295-23314 4:01:01

GGCACTGGCTCAGGGCCAGG

23310 23329 21788 25315 CDS product single-stranded DNA-binding protein 23310-23329 4:01:01

CCAGGTCCTGCAGCACCGCC

23397 23414 21788 25315 CDS product single-stranded DNA-binding protein 23397-23414 4:01:01

GGGACATGGTGTGGTTGG

23478 23494 21788 25315 CDS product single-stranded DNA-binding protein 23478-23494 4:01:01

CCGCCGCCTGCTCGACC

23564 23590 21788 25315 CDS product single-stranded DNA-binding protein 23564-23590 6:03:01

CCGGCCACGTCCGCCAGCAGCCGGTCC

23586 23602 21788 25315 CDS product single-stranded DNA-binding protein 23586-23602 4:01:01

GGTCCACGGCGGCGCGG

23757 23783 21788 25315 CDS product single-stranded DNA-binding protein 23757-23783 6:03:01

GGGAGCGGTGGCGCAGGCGGTGCAGGG

23797 23815 21788 25315 CDS product single-stranded DNA-binding protein 23797-23815 4:01:01

GGCGGGGCGCGAGGCGCGG

23830 23855 21788 25315 CDS product single-stranded DNA-binding protein 23830-23855 4:01:01

GGGGACCTCGGCGTCGAGGCTGCCGG

23957 23980 21788 25315 CDS product single-stranded DNA-binding protein 23957-23980 4:01:01

CCGCACGCCATCACCAGGTGGTCC

23994 24023 21788 25315 CDS product single-stranded DNA-binding protein 23994-24023 4:01:01

GGTTGCCCGGGGCCGTCGCGGGCTGCGAGG

24062 24081 21788 25315 CDS product single-stranded DNA-binding protein 24062-24081 4:01:01

GGGTTCCCGGCCAGGTAGGG

24146 24163 21788 25315 CDS product single-stranded DNA-binding protein 24146-24163 4:01:01
CCCGCGTCGTCCACCTCC

24195 24217 21788 25315 CDS product single-stranded DNA-binding protein 24195-24217 5:02:01
CCAGCGCCCCCACGAGGCCGGCC

24271 24284 21788 25315 CDS product single-stranded DNA-binding protein 24271-24284 4:01:01 GGAGGCGGCCGCGG

24279 24307 21788 25315 CDS product single-stranded DNA-binding protein 24279-24307 6:03:01
CCGCGGCGCCGCCGTCCTTGGCGCCCGCC

24420 24440 21788 25315 CDS product single-stranded DNA-binding protein 24420-24440 4:01:01
GGCGCTCGAGGCCGCCGGCGG

24431 24461 21788 25315 CDS product single-stranded DNA-binding protein 24431-24461 7:04:01
CCGCCGGCGGCCGCGTCGCCGCCGCGCCGCC

24481 24503 21788 25315 CDS product single-stranded DNA-binding protein 24481-24503 4:01:01
GGTCACGTCGGCGGGCAGCACGG

24528 24566 21788 25315 CDS product single-stranded DNA-binding protein 24528-24566 7:04:01
CCAGGTGCGCCGCGCCGCGCGCCACCGCGTCCAGGTTCC

24579 24595 21788 25315 CDS product single-stranded DNA-binding protein 24579-24595 4:01:01
CCACGGCCGCCGGGCCC

24670 24698 21788 25315 CDS product single-stranded DNA-binding protein 24670-24698 5:02:01
GGAGCGGTTCTTGGCGTTGAAGGGGTCGG

24780 24795 21788 25315 CDS product single-stranded DNA-binding protein 24780-24795 4:01:01 CCGTGCCCGCGCCCCC

24825 24850 21788 25315 CDS product single-stranded DNA-binding protein 24825-24850 6:03:01
CCGCCTCCTTGAAGCCGTCGGCCACC

24873 24890 21788 25315 CDS product single-stranded DNA-binding protein 24873-24890 4:01:01

CCGCGGCCAGGCCGAGCC

24900 24919 21788 25315 CDS product single-stranded DNA-binding protein 24900-24919 4:01:01 CCTCCGCGCCCGTCGTCTCC

24930 24957 21788 25315 CDS product single-stranded DNA-binding protein 24930-24957 5:02:01 CCACCGGCGTCCGGTAGGCCGAGAACCC

25051 25068 21788 25315 CDS product single-stranded DNA-binding protein 25051-25068 4:01:01 GGGGTGGAAGTGGTTGGG

25087 25150 21788 25315 CDS product single-stranded DNA-binding protein 25087-25150 12:09:03 CCCGCCGCCGAGCCCGGTGGTCCGGGCGCCCGCCACCACGGCCACGTGCCCGGCGAAGCCGGCC

25138 25162 21788 25315 CDS product single-stranded DNA-binding protein 25138-25162 4:01:01 GGCGAAGCCGGCCTCGACGGTCAGG

25163 25186 21788 25315 CDS product single-stranded DNA-binding protein 25163-25186 5:02:01 CCGCGCACCAGCGGCGCCACCGCC

25274 25307 21788 25315 CDS product single-stranded DNA-binding protein 25274-25307 6:03:01 GGCGCGGCGCGCACGGTCACGGTCTTGGCCGCGG

25607 25621 25606 28752 CDS product DNA polymerase catalytic subunit 25607-25621 4:01:01 GGCGGCGCGGCAGGG

25744 25761 25606 28752 CDS product DNA polymerase catalytic subunit 25744-25761 4:01:01 CCACGCGGCCCATCCTCC

25807 25828 25606 28752 CDS product DNA polymerase catalytic subunit 25807-25828 4:01:01 CCCCCGAGCGGCCCGTGAGTCC

25999 26016 25606 28752 CDS product DNA polymerase catalytic subunit 25999-26016 4:01:01 CCGAGGCCGACGCCGCCC

26075 26093 25606 28752 CDS product DNA polymerase catalytic subunit 26075-26093 4:01:01

CCCGCGCCTCGGGCCCGCC

26143 26163 25606 28752 CDS product DNA polymerase catalytic subunit 26143-26163 4:01:01

CCCCCCGGCGGCCCTTCTACC

26300 26318 25606 28752 CDS product DNA polymerase catalytic subunit 26300-26318 4:01:01

CCGCCTGCGGCCCGGGCCC

26308 26342 25606 28752 CDS product DNA polymerase catalytic subunit 26308-26342 6:03:01

GGCCCGGGCCCGCGGGGGAGCGCGTGGTGCTGCGG

26476 26514 25606 28752 CDS product DNA polymerase catalytic subunit 26476-26514 7:04:01

CCTTCCCCGCCGCCGAGAACCCCGAGGACCTGGTGATCC

26933 26950 25606 28752 CDS product DNA polymerase catalytic subunit 26933-26950 4:01:01

GGCCGAGGAGGCGCTCGG

27242 27273 25606 28752 CDS product DNA polymerase catalytic subunit 27242-27273 6:03:01

GGACGACGGCGGCGGCTACCAGGGCGCCAAGG

27584 27600 25606 28752 CDS product DNA polymerase catalytic subunit 27584-27600 4:01:01

GGCGGCCATCAAGGTGG

27611 27641 25606 28752 CDS product DNA polymerase catalytic subunit 27611-27641 5:02:01

GGTGTACGGGTTCACGGGCGTGGCCAACGGG

27647 27687 25606 28752 CDS product DNA polymerase catalytic subunit 27647-27687 7:04:01

CCCCTGCCTCCCCGTGGCGGCCACCGTGACGACCATCGGCC

27758 27776 25606 28752 CDS product DNA polymerase catalytic subunit 27758-27776 4:01:01

CCTGCCCGCGCGCCCGCCC

27800 27822 25606 28752 CDS product DNA polymerase catalytic subunit 27800-27822 4:01:01

GGTCTACGGGGACACGGACTCGG

27902 27917 25606 28752 CDS product DNA polymerase catalytic subunit 27902-27917 4:01:01 CCTCTTCCGCCCCCCC
28089 28116 25606 28752 CDS product DNA polymerase catalytic subunit 28089-28116 5:02:01 GGCGACGAGGCCGTCTCGGCGGCAGCGG
28221 28242 25606 28752 CDS product DNA polymerase catalytic subunit 28221-28242 5:02:01 GGCGGCGCCGGCCTGGACGTGG
28301 28323 25606 28752 CDS product DNA polymerase catalytic subunit 28301-28323 5:02:01 CCTGCCGCACCTCACCGTCTACC
28417 28445 25606 28752 CDS product DNA polymerase catalytic subunit 28417-28445 5:02:01 CCGGGGCCGTGCGCCCGGGCCTCGCGCCC
28466 28484 25606 28752 CDS product DNA polymerase catalytic subunit 28466-28484 4:01:01 CCTGGCCGAGGACCCCGCC
28875 28889 28673 29488 CDS product nuclear egress lamina protein 28875-28889 4:01:01 GGTGGCCGGCGCAGG
29085 29113 28673 29488 CDS product nuclear egress lamina protein 29085-29113 5:02:01 CCGAGGGGTCCCGCGCGGCCACCGAGGCC
29101 29125 28673 29488 CDS product nuclear egress lamina protein 29101-29125 4:01:01 GGCCACCGAGGCCAGGAACACGCGG
29198 29221 28673 29488 CDS product nuclear egress lamina protein 29198-29221 5:02:01 GGCTCGGCGGCGGCGCACGTGGGG
29280 29292 28673 29488 CDS product nuclear egress lamina protein 29280-29292 4:01:01 CCACCGCCTGGCC
29371 29400 28673 29488 CDS product nuclear egress lamina protein 29371-29400 4:01:01 GGGCTGCGCGGCCGCGTAGGCAAAGTAGGG
29718 29753 29481 30893 CDS product DNA packaging protein UL32 29718-29753 6:03:01 GGGTCCGCCTGGCGCTCGGCCACGGCGCACAGGGGG

30059 30077 29481 30893 CDS product DNA packaging protein UL32 30059-30077 4:01:01 GGCGGTGTTGCGGGCCAGG
30073 30096 29481 30893 CDS product DNA packaging protein UL32 30073-30096 4:01:01 CCAGGAAGCCCTCGTGCTCCGCCC
30157 30176 29481 30893 CDS product DNA packaging protein UL32 30157-30176 4:01:01 CCCCCGTGGCCACGTTGCCC
30188 30216 29481 30893 CDS product DNA packaging protein UL32 30188-30216 6:03:01
GGCGAGGTCGGCGTAGCCGGGCCCGGCGG
30220 30241 29481 30893 CDS product DNA packaging protein UL32 30220-30241 5:02:01 CCGCGTCCGCGGCCCCGCCCCC
30242 30258 29481 30893 CDS product DNA packaging protein UL32 30242-30258 5:02:01 GGTGGCGGCGGCGACGG
30280 30296 29481 30893 CDS product DNA packaging protein UL32 30280-30296 4:01:01 CCTCGCGCTCCGCCGCC
30324 30347 29481 30893 CDS product DNA packaging protein UL32 30324-30347 5:02:01 CCCGTGTGGTCCTTCCAGCCCCCC
30593 30627 29481 30893 CDS product DNA packaging protein UL32 30593-30627 7:04:01
GGCCGTGGCGCACGGGGGGCAGGCCAGGGCCTTGG
30637 30654 29481 30893 CDS product DNA packaging protein UL32 30637-30654 4:01:01 CCGCGTAGTCCGCCAGCC
30721 30758 29481 30893 CDS product DNA packaging protein UL32 30721-30758 6:03:01
CCAGCGCCAGCGGCGCCGCGGCGGCCTCCACGAAGCCC
30893 30918 30892 31239 CDS product DNA packaging protein UL33 30893-30918 7:04:01
GGCGCGCGGCGGCGGCGGCGCGGCGG
30982 30997 30892 31239 CDS product DNA packaging protein UL33 30982-30997 4:01:01 CCAACTACCTGCCCCC
31134 31154 30892 31239 CDS product DNA packaging protein UL33 31134-31154 4:01:01 GGGGAGGCGCTGCGGGCGCGG
31473 31494 31398 32186 CDS product nuclear egress membrane protein 31473-31494 4:01:01
CCTGCGACCCCGAGCGCCCCCC
31513 31530 31398 32186 CDS product nuclear egress membrane protein 31513-31530 4:01:01 GGTCCACGGGCAGGACGG
31657 31681 31398 32186 CDS product nuclear egress membrane protein 31657-31681 6:03:01
CCGGCCCGCCGACGCCCCCCTGGCC

31772 31789 31398 32186 CDS product nuclear egress membrane protein 31772-31789 4:01:01 GGGGGCGTGGACACGCGG
31891 31906 31398 32186 CDS product nuclear egress membrane protein 31891-31906 4:01:01 CCTCCTGGACCGCGCC
31937 31972 31398 32186 CDS product nuclear egress membrane protein 31937-31972 8:05:02 GGCGGGGGTGCCGGCGGCGGCGACGACGGCGAGGGG
31973 32002 31398 32186 CDS product nuclear egress membrane protein 31973-32002 6:03:01 CCCTCGCCCCGCGCGCCCATCCGCCCGACC
32047 32068 31398 32186 CDS product nuclear egress membrane protein 32047-32068 5:02:01 CCAGGCCGCGTACCCCCCGCCC
32406 32426 32241 32552 CDS product small capsid protein 32406-32426 4:01:01 CCTCCCTGGCCCTCGTGAACC
32461 32479 32241 32552 CDS product small capsid protein 32461-32479 4:01:01 CCAGCCCATGTTCGCCACC
32496 32514 32241 32552 CDS product small capsid protein 32496-32514 4:01:01 GGGCCCGGCCCACGGTCGG
33280 33329 33060 42314 CDS product large tegument protein 33280-33329 10:07:02 CCTCGTCCCCGTCCTCGTCCCCCTCCTCGTCCTCCAGGCCCCAGGGGTCC
33388 33424 33060 42314 CDS product large tegument protein 33388-33424 8:05:02 GGCCGAGGCCGCCCAGGGGAGAGGCCGGCGGCGGAGG
33444 33464 33060 42314 CDS product large tegument protein 33444-33464 6:03:01 GGCGGAGGATGGGCGGGAGGG
33495 33509 33060 42314 CDS product large tegument protein 33495-33509 5:02:01 GGGGGCTGGTGGGGG
33519 33538 33060 42314 CDS product large tegument protein 33519-33538 4:01:01 GGGGAGCGCGGGGCCGGCGG
33549 33574 33060 42314 CDS product large tegument protein 33549-33574 6:03:01 GGCGGGAGCTCGGGGGTGGTGCCGGG
33585 33598 33060 42314 CDS product large tegument protein 33585-33598 4:01:01 GGCGGCGGCCACGG
33666 33697 33060 42314 CDS product large tegument protein 33666-33697 9:06:02 GGCTGAGGGGGCGGCGGTGGCGGCAGCGGCGG
33710 33744 33060 42314 CDS product large tegument protein 33710-33744 7:04:01

GGGGAACTGGACCGGCGGGTCCGACGGCGGCGGGG
33785 33803 33060 42314 CDS product large tegument protein 33785-33803 4:01:01 CCACGTCCACGGCGCCTCC
33822 33844 33060 42314 CDS product large tegument protein 33822-33844 6:03:01 GGCTGCGGCGGCAGCGGCGGAGG
33882 34008 33060 42314 CDS product large tegument protein 33882-34008 30:27:07
GGCGTCGGGGCCGGCCGCGAAGGTGGTGGGGCCGGCGGTGGTGCGGCCGGCCGCGAAGGTGGTGGGGCCGGCGGTGGTGCGGCGGGGGCGGAAGAGGTGGTGGCGGAAGACGAGGAGGTGGTGGTGG
34015 34034 33060 42314 CDS product large tegument protein 34015-34034 5:02:01 CCGCCGCCGCTGCTGCCGCC
34058 34068 33060 42314 CDS product large tegument protein 34058-34068 4:01:01 GGAGGGGGCGG
34092 34110 33060 42314 CDS product large tegument protein 34092-34110 4:01:01 GGAGACCGCGGCGGCTGGG
34119 34150 33060 42314 CDS product large tegument protein 34119-34150 6:03:01
GGGGCGGGAGTCTCTGGCGAGGCCGGCAGAGG
34176 34233 33060 42314 CDS product large tegument protein 34176-34233 13:10:03
GGCTGCGGCGGCGGGATGTGGAGACGGAGGTGCCCGGCCGGCGGGCGCGGGCCGGCGG
34248 34264 33060 42314 CDS product large tegument protein 34248-34264 4:01:01 CCTCCGGGGCCGACTCC
34265 34278 33060 42314 CDS product large tegument protein 34265-34278 4:01:01 GGGGGCGGGGACGG
34332 34363 33060 42314 CDS product large tegument protein 34332-34363 6:03:01
GGCGCTTGGGCTTGGGCTTGAGGCGGGCGCGG
34376 34393 33060 42314 CDS product large tegument protein 34376-34393 4:01:01 GGGAGGACGGCCCTCGGG
34418 34450 33060 42314 CDS product large tegument protein 34418-34450 5:02:01
GGTCTGGCGCGGGGCCGATACGGGCCTTCGCGG
34594 34615 33060 42314 CDS product large tegument protein 34594-34615 5:02:01 CCGCAGCCGGGCCACCGTCTCC
34626 34645 33060 42314 CDS product large tegument protein 34626-34645 4:01:01 CCACCACTCCCGGGGGCTCC
34637 34659 33060 42314 CDS product large tegument protein 34637-34659 5:02:01 GGGGGCTCCAGGAGGTGCTCGGG
34707 34791 33060 42314 CDS product large tegument protein 34707-34791 16:13:04

GGTGTTCCGGCCGGGGAAGAAGAGGATGAGGCGGCTTCTCGGGACGGGGTCGAGGCGGCGGAGGAGGACGCGGACGCGGGCTCGG

34891 34911 33060 42314 CDS product large tegument protein 34891-34911 6:03:01 GGGGGCGGCCCGGGGAGGCGG

34914 34930 33060 42314 CDS product large tegument protein 34914-34930 5:02:01 CCCGCCACCTTCCCACC

34937 34972 33060 42314 CDS product large tegument protein 34937-34972 6:03:01
GGCGTCGCGGACGTCCGGCGGTGCACCGGCCTTCGG

34994 35040 33060 42314 CDS product large tegument protein 34994-35040 9:06:02
GGATTTCTTGGCCGATGGGGCGGCCCGGGCGCGGGGGGCGTGGGGG

35049 35081 33060 42314 CDS product large tegument protein 35049-35081 7:04:01
GGGGCAGACGGTGGTGGAGGGGCGGGCCTGGGG

35100 35135 33060 42314 CDS product large tegument protein 35100-35135 9:06:02
GGTGGTGGAGGGGCGGGCGGTGCGGCGGGCCTGGGG

35154 35182 33060 42314 CDS product large tegument protein 35154-35182 8:05:02
GGTGGTGGGGGGGCGGGCGGGGCGGGGGG

35193 35231 33060 42314 CDS product large tegument protein 35193-35231 9:06:02
GGCTTGGCGGCCGGTTGGGACGACGGGGGCTGAGGGGGG

35240 35274 33060 42314 CDS product large tegument protein 35240-35274 9:06:02
GGCCGCGGTCGCGGCGGATGGTGGAGGGGGGGGCGG

35284 35299 33060 42314 CDS product large tegument protein 35284-35299 4:01:01 GGGCCCGGGTCGGCGG

35308 35496 33060 42314 CDS product large tegument protein 35308-35496 40:37:10
GGGGGGTGGCCTTGGGCGCGGTGGTGGCGGCGGCGGTGGCGGGCTGCGCCGGGGGCTTCTGGGCAGGGGGGGCCTGGGCCGACGGGGGCTGCGGCGGCGGTGGGGGACCCTGGGGTTGGGGGGTCGGC
TTGGCCGCGGCGGGCGGCTGGGCCTGGGGGGCCTCCGGGGGGGCCGTCTCGGGCGCCGGGG

35458 35480 33060 42314 CDS product large tegument protein 35458-35480 4:01:01 CCTGGGGGGCCTCCGGGGGGGCC

35526 35536 33060 42314 CDS product large tegument protein 35526-35536 4:01:01 GGCGGCGGGGG

35586 35608 33060 42314 CDS product large tegument protein 35586-35608 5:02:01 GGCTTGGGGGCGGGCTCCCGGGG
35618 35650 33060 42314 CDS product large tegument protein 35618-35650 8:05:02 GGGGGCCGCGGCGGCCTTGGTGCCGGCGGCGGG
35659 35671 33060 42314 CDS product large tegument protein 35659-35671 4:01:01 GGGCGGCGGGGGG
35682 35709 33060 42314 CDS product large tegument protein 35682-35709 5:02:01 GGCTGCGGCTGCGGCTTGGTGAGCGGGG
35720 35764 33060 42314 CDS product large tegument protein 35720-35764 10:07:02 GGGCTTGGCGGGCGGCGGCGCCGGCGGGGCCAGCTTGGCGGCGGG
35777 35863 33060 42314 CDS product large tegument protein 35777-35863 17:14:04 GGGCTTGGCGGGCGGCGGCGCCGGCGGGGCCGCCGGGGCCAACGGCGGGAGCGTGGGCGGGTCGACCGGCGGCGACGGGGGCAGCGG
35797 35816 33060 42314 CDS product large tegument protein 35797-35816 4:01:01 CCGGCGGGGCCGCCGGGGCC
35939 35957 33060 42314 CDS product large tegument protein 35939-35957 5:02:01 CCCGTCGTCCTCCCCCGCC
35963 36001 33060 42314 CDS product large tegument protein 35963-36001 7:04:01 GGGGGGCAGCCCGGGGATCGGGGCCGCGGGCCGGGTGGG
36014 36056 33060 42314 CDS product large tegument protein 36014-36056 7:04:01 GGCGTATGGGAACGGGTCGCCCAGGTTGCGGAAGCTCGGCCGG
36067 36086 33060 42314 CDS product large tegument protein 36067-36086 4:01:01 CCTCCGAGGTGGCCGGGTCC
36074 36093 33060 42314 CDS product large tegument protein 36074-36093 4:01:01 GGTGGCCGGGTCCACGCGGG
36216 36238 33060 42314 CDS product large tegument protein 36216-36238 5:02:01 GGGCGTCGCGGCGGCTCGGGCGG
36286 36310 33060 42314 CDS product large tegument protein 36286-36310 5:02:01 GGCAGGCCGGCTCGCCGGCCAGGGG
36292 36317 33060 42314 CDS product large tegument protein 36292-36317 5:02:01 CCGGCTCGCCGGCCAGGGGCCGCGCC
36547 36566 33060 42314 CDS product large tegument protein 36547-36566 5:02:01 CCGCCGCCGCGGCCAGGTCC
36651 36680 33060 42314 CDS product large tegument protein 36651-36680 5:02:01 CCGGCGCCCGGCGCGCCGAGCGCCGCGGCC

36698 36723 33060 42314 CDS product large tegument protein 36698-36723 4:01:01 GGCGTCCAGGCGCTCGGTGACCGCGG

36844 36896 33060 42314 CDS product large tegument protein 36844-36896 8:05:02 CCAGCCGCTGGCCCGTCTCCGTGTGCGCCCAGGGCGCCCACAGCTCCGTCTCC

36981 37007 33060 42314 CDS product large tegument protein 36981-37007 5:02:01 CCCGCGTCCAGCGTGTCCACCGCGTCC

37090 37116 33060 42314 CDS product large tegument protein 37090-37116 4:01:01 GGTGCGTCGGCTCGCCGGCCACCGCGG

37104 37121 33060 42314 CDS product large tegument protein 37104-37121 5:02:01 CCGGCCACCGCGGCCACC

37142 37157 33060 42314 CDS product large tegument protein 37142-37157 4:01:01 CCGCACCGCCGGCTCC

37234 37260 33060 42314 CDS product large tegument protein 37234-37260 5:02:01 CCGCGGCGGCCTTGGCCGCCGAGTCCC

37265 37277 33060 42314 CDS product large tegument protein 37265-37277 4:01:01 GGCGCGGCGGTGG

37368 37397 33060 42314 CDS product large tegument protein 37368-37397 5:02:01 CCCGCCACGGTCGCCGTCTCCATGAGCCCC

37634 37657 33060 42314 CDS product large tegument protein 37634-37657 4:01:01 GGCGCGCGGCCGCGCCGGCGTCGG

37658 37676 33060 42314 CDS product large tegument protein 37658-37676 4:01:01 CCCCGCGCCGCCGTAGGCC

37711 37733 33060 42314 CDS product large tegument protein 37711-37733 5:02:01 GGTAGGCGGCGTACTTGGGCGGG

37736 37754 33060 42314 CDS product large tegument protein 37736-37754 4:01:01 CCACCGCGGCAGCCGCTCC

37937 37959 33060 42314 CDS product large tegument protein 37937-37959 5:02:01 GGCGGGCAGGCGCGGGTAGACGG

37975 37994 33060 42314 CDS product large tegument protein 37975-37994 4:01:01 CCAGCACCTCCATGGTCGCC

38014 38031 33060 42314 CDS product large tegument protein 38014-38031 4:01:01 GGGCCTCGGCCAGGGGGG

38017 38039 33060 42314 CDS product large tegument protein 38017-38039 4:01:01 CCTCGGCCAGGGGGGCCTCGTCC

38042 38057 33060 42314 CDS product large tegument protein 38042-38057 4:01:01 GGAGGCGCCGGCGCGG

38069 38124 33060 42314 CDS product large tegument protein 38069-38124 9:06:02 CCCAAAGTCCTCCCAGGCCTCGTCCCAGGCGACCTCGGCGGCCGTCAGCCGCTGCC

38314 38336 33060 42314 CDS product large tegument protein 38314-38336 5:02:01 CCGCCTGCGTCACCAGCTCCTCC

38339 38351 33060 42314 CDS product large tegument protein 38339-38351 4:01:01 GGAGGCGGCCCGG
38366 38377 33060 42314 CDS product large tegument protein 38366-38377 4:01:01 GGCCGGCGGCGG
38437 38456 33060 42314 CDS product large tegument protein 38437-38456 6:03:01 CCGCCGCCGCGTCCACCACC
38482 38510 33060 42314 CDS product large tegument protein 38482-38510 7:04:01 CCGTCGCCGCGGCCTGGTCCGCCTCCGCC
38745 38762 33060 42314 CDS product large tegument protein 38745-38762 4:01:01 CCGCGGCTGCCGCCGGCC
38773 38807 33060 42314 CDS product large tegument protein 38773-38807 5:02:01 CCGCGCGCCGCGCGCCCAGGGCGGCCAGCTCGGCC
38869 38891 33060 42314 CDS product large tegument protein 38869-38891 4:01:01 CCATGTCCGCCCCGACGAAGCCC
38924 38937 33060 42314 CDS product large tegument protein 38924-38937 4:01:01 GGCGGCGGCGCGGG
39072 39085 33060 42314 CDS product large tegument protein 39072-39085 4:01:01 CCCGCCGCCGCGCC
39086 39123 33060 42314 CDS product large tegument protein 39086-39123 6:03:01 GGCGTTCTCGGGCAGGTGCGGGTTGAAGGCGAACACGG
39137 39156 33060 42314 CDS product large tegument protein 39137-39156 4:01:01 GGTCACGGTCTCGGCGTTGG
39322 39332 33060 42314 CDS product large tegument protein 39322-39332 4:01:01 CCACCTCCGCC
39343 39376 33060 42314 CDS product large tegument protein 39343-39376 8:05:02 CCTCCACGGCCGCCACGGCCGCCTCCAGCTCGCC
39421 39455 33060 42314 CDS product large tegument protein 39421-39455 7:04:01 CCTCCACGGCGGCCGCGCGCGCCACCTCCAGCGCC
39517 39539 33060 42314 CDS product large tegument protein 39517-39539 6:03:01 CCACGGCCGCCGCCACGGCCGCC
39550 39572 33060 42314 CDS product large tegument protein 39550-39572 4:01:01 CCTGCAGCGCCGCCACGCGCTCC
39608 39647 33060 42314 CDS product large tegument protein 39608-39647 6:03:01 CCGCGCCAGCGGCTCCAGGAAGCCCAGCTCCGGGTGCTCC

39664 39722 33060 42314 CDS product large tegument protein 39664-39722 14:11:03
CCCCCGCCGCGCGCGCCGCCTTCTCCGCCGTGTCCGCGTCCGCCAGCGCCGCCGCCACC
39800 39820 33060 42314 CDS product large tegument protein 39800-39820 4:01:01 CCCGGCGCCCACCAGCGCGCC
39853 39887 33060 42314 CDS product large tegument protein 39853-39887 6:03:01
CCACCAGGCGCCACAGCGCCGCGAGCACCGTGCCC
39922 39935 33060 42314 CDS product large tegument protein 39922-39935 4:01:01 CCACCGCCGGGCCC
40138 40157 33060 42314 CDS product large tegument protein 40138-40157 4:01:01 CCTCGTCCGGGGCGGCCGCC
40146 40173 33060 42314 CDS product large tegument protein 40146-40173 5:02:01 GGGGCGGCCGCCAGGGCGGCGTCCATGG
40189 40215 33060 42314 CDS product large tegument protein 40189-40215 5:02:01 GGTCGGCGTCGGCCGAGGCGCGCTTGG
40268 40301 33060 42314 CDS product large tegument protein 40268-40301 6:03:01
CCGCTCCAGGTGCCCGTGCGCCTGCGCCTCGGCC
40345 40358 33060 42314 CDS product large tegument protein 40345-40358 4:01:01 CCTCGCCCGCCTCC
40418 40436 33060 42314 CDS product large tegument protein 40418-40436 4:01:01 CCGCTCCTCGGCGGCCGCC
40642 40673 33060 42314 CDS product large tegument protein 40642-40673 7:04:01
CCGCGCCGCCCGCGCCGGGCGCCCCCGCGTCC
40830 40856 33060 42314 CDS product large tegument protein 40830-40856 5:02:01 CCGCCGGGGTCCCGCGCCGCGGCGTCC
40951 40973 33060 42314 CDS product large tegument protein 40951-40973 4:01:01 CCGCGACCAGCGCCAGCTTGGCC
41047 41075 33060 42314 CDS product large tegument protein 41047-41075 5:02:01
CCACAAAGTCCCCGGCCGGGCCGCGCTCC
41145 41165 33060 42314 CDS product large tegument protein 41145-41165 4:01:01 CCGTTCTCCACCAGAAAGTCC
41210 41234 33060 42314 CDS product large tegument protein 41210-41234 4:01:01 GGCGCGCCCGGGGAACAGGCTCCGG
41336 41357 33060 42314 CDS product large tegument protein 41336-41357 4:01:01 GGGCTCGCGGGTGCGCGGGCGG
41372 41384 33060 42314 CDS product large tegument protein 41372-41384 4:01:01 CCGCCCGCCGACC

41401 41432 33060 42314 CDS product large tegument protein 41401-41432 6:03:01
GGTCCTCCTGGCTCGCCGGCCACGGGGGCCGG

41404 41436 33060 42314 CDS product large tegument protein 41404-41436 6:03:01
CCTCCTGGCTCGCCGGCCACGGGGGCCGGCGCC

41461 41485 33060 42314 CDS product large tegument protein 41461-41485 6:03:01 GGACCCGCGGCGGCGGCGGGAGCGG

41495 41517 33060 42314 CDS product large tegument protein 41495-41517 6:03:01 GGGTGCCGGGGCGGGGGCGGCGG

41528 41576 33060 42314 CDS product large tegument protein 41528-41576 10:07:02
GGGGACGGCGGGGACGGGGGCGACGGCGAGGACGGGGATGGCCCCGGGG

41586 41638 33060 42314 CDS product large tegument protein 41586-41638 11:08:02
GGCGGCGCGGCGGCCGCCGGGTCCGGCGGCAGGGCCATCGGGTGCACGGAGGG

41699 41729 33060 42314 CDS product large tegument protein 41699-41729 6:03:01
GGGCACGGCGGCGAACTGGTCGTGGGGCAGG

41878 41901 33060 42314 CDS product large tegument protein 41878-41901 5:02:01 GGCGGTAGAGGCCCAGGCCCACGG

41889 41915 33060 42314 CDS product large tegument protein 41889-41915 5:02:01 CCCAGGCCCACGGCGCCGACCACGACC

42114 42143 33060 42314 CDS product large tegument protein 42114-42143 7:04:01
CCCCCGCCGGGCCCCGTCCACGCCTGGCCC

42164 42183 33060 42314 CDS product large tegument protein 42164-42183 4:01:01 GGCGTCGGCCGTCAGGGCGG

42208 42236 33060 42314 CDS product large tegument protein 42208-42236 6:03:01
GGCGCAGGAAGGAGAGGGAGGAGCGCAGG

42288 42308 33060 42314 CDS product large tegument protein 42288-42308 4:01:01 CCGACGACCACCGCGTCGGCC

42371 42400 42352 45111 CDS product tegument protein UL37 42371-42400 5:02:01
CCTCGGCCAGGAGCCGATCCACGGGCACCC

42532 42555 42352 45111 CDS product tegument protein UL37 42532-42555 6:03:01 CCCGCCTCGCCGTCGCCCGCCACC

42603 42626 42352 45111 CDS product tegument protein UL37 42603-42626 5:02:01 GGGCGTGAAGGCGGGCGCGGGCGG
42629 42652 42352 45111 CDS product tegument protein UL37 42629-42652 4:01:01 CCTGCTCCTCGCGCGCCTCGGTCC
42756 42802 42352 45111 CDS product tegument protein UL37 42756-42802 10:07:02
GGGCCGCAGGACGGTGGCGGCCTGCAGGCGGCCGGCCGTGAGGGCGG
42972 42991 42352 45111 CDS product tegument protein UL37 42972-42991 4:01:01 GGCGCGGAAGGCGCCCACGG
42985 43023 42352 45111 CDS product tegument protein UL37 42985-43023 6:03:01
CCCACGGCGTCCACGACGTCCGCCAGACGCCACGTGCCC
43122 43144 42352 45111 CDS product tegument protein UL37 43122-43144 4:01:01 GGCCACGGCGCCCGCGGCCGTGG
43124 43155 42352 45111 CDS product tegument protein UL37 43124-43155 5:02:01
CCACGGCGCCCGCGGCCGTGGCCATCGAGTCC
43190 43204 42352 45111 CDS product tegument protein UL37 43190-43204 4:01:01 GGCGGCGCCGGAGGG
43217 43237 42352 45111 CDS product tegument protein UL37 43217-43237 4:01:01 GGTCCCCGAGGAAGGCGCTGG
43248 43265 42352 45111 CDS product tegument protein UL37 43248-43265 4:01:01 GGGCGGCGCCGGCGCCGG
43256 43287 42352 45111 CDS product tegument protein UL37 43256-43287 5:02:01
CCGGCGCCGGCGCCTCGAGCACCTCGGCGGCC
43343 43374 42352 45111 CDS product tegument protein UL37 43343-43374 6:03:01
CCACCGTCAGGTCCTCGCGCGCCAGCGCCGCC
43463 43497 42352 45111 CDS product tegument protein UL37 43463-43497 7:04:01
GGTCCGCGCGGCGGTCGTAGGAGGCCGTCAGGAGG
43536 43569 42352 45111 CDS product tegument protein UL37 43536-43569 6:03:01
GGGCGCGAAGGACGGGTCGGCCATGGCCTCGAGG
43595 43614 42352 45111 CDS product tegument protein UL37 43595-43614 5:02:01 GGTCCGCGGCGGCGGCGAGG
43659 43686 42352 45111 CDS product tegument protein UL37 43659-43686 4:01:01 GGCGCCGTCGGCGCGCAGGCCCCAGCGG

43763 43773 42352 45111 CDS product tegument protein UL37 43763-43773 4:01:01 CCGCCGCCACC
43787 43812 42352 45111 CDS product tegument protein UL37 43787-43812 4:01:01 CCGCGCGCGCCGCGCCCACGTGCTCC
43872 43895 42352 45111 CDS product tegument protein UL37 43872-43895 4:01:01 CCCCAGCGCCGCCGTCACGTCCCC
43987 44009 42352 45111 CDS product tegument protein UL37 43987-44009 5:02:01 CCGCCGACGACCGCCGTGCAGCC
44066 44082 42352 45111 CDS product tegument protein UL37 44066-44082 4:01:01 GGAGGTCGCAGTGGCGG
44138 44172 42352 45111 CDS product tegument protein UL37 44138-44172 7:04:01
CCTGCACCGCCTCCCACACGCCCGTGGCGTCCACC
44180 44200 42352 45111 CDS product tegument protein UL37 44180-44200 5:02:01 GGTCGAGGTCCGCGGCGGCGG
44213 44253 42352 45111 CDS product tegument protein UL37 44213-44253 6:03:01
CCTGCGCCCCGGGGTCCAGCAGCGCCAGCACGCCCTGGTCC
44366 44412 42352 45111 CDS product tegument protein UL37 44366-44412 9:06:02
CCTCGCCCACGGGCACCAGCGCCGCCTCGTCCACGGCCCGCGCCGCC
44437 44454 42352 45111 CDS product tegument protein UL37 44437-44454 4:01:01 GGGTTCTGGCGGTCGTGG
44747 44766 42352 45111 CDS product tegument protein UL37 44747-44766 4:01:01 GGCGGAACATGGCGTCGGGG
44793 44815 42352 45111 CDS product tegument protein UL37 44793-44815 5:02:01 GGCCTCGGTGAGGGCGGCCGTGG
44889 44907 42352 45111 CDS product tegument protein UL37 44889-44907 4:01:01 CCACAGCGCCAGGCCGGCC
44900 44926 42352 45111 CDS product tegument protein UL37 44900-44926 5:02:01 GGCCGGCCAGGTTCTCGGCGAGCACGG
44955 44970 42352 45111 CDS product tegument protein UL37 44955-44970 4:01:01 GGCGCGGCAGGCCAGG
45063 45079 42352 45111 CDS product tegument protein UL37 45063-45079 4:01:01 GGCGACGGCGTGGTCGG
45322 45367 45168 46274 CDS product capsid triplex subunit 1 45322-45367 9:06:02
CCACCCCGCGGACCTGGCCGGCGCCGCCTACGCGGCCACGCCGCCC
45388 45400 45168 46274 CDS product capsid triplex subunit 1 45388-45400 4:01:01 GGAGGAGGCGTGG
45415 45441 45168 46274 CDS product capsid triplex subunit 1 45415-45441 6:03:01

GGGCGGGCGCGGCCTCGGCGGCGACGG

45585 45614 45168 46274 CDS product capsid triplex subunit 1 45585-45614 5:02:01 CCATGATCCGGAGCCACGTGTTCCCGCACC

45685 45707 45168 46274 CDS product capsid triplex subunit 1 45685-45707 5:02:01 GGCCCGCGGCGACCAGGAGGCGG

45909 45928 45168 46274 CDS product capsid triplex subunit 1 45909-45928 4:01:01 CCCTCAGCTTCCTCCTGGCC

45969 46016 45168 46274 CDS product capsid triplex subunit 1 45969-46016 11:08:02 CCGACGCCCCCGCCGCGCCCGCCGCCGCCGCGGCCTTCCCCGCGTACC

46239 46259 45168 46274 CDS product capsid triplex subunit 1 46239-46259 5:02:01 GGGACGTGGGGGCGTGGGAGG

46622 46651 46605 48977 CDS product ribonucleotide reductase subunit 1 46622-46651 9:06:02 CCCGCCGCCTCCTCGTCCGCCGCCGCCCCC

46666 46684 46605 48977 CDS product ribonucleotide reductase subunit 1 46666-46684 4:01:01 CCTCGACGCCGCCTGCCCC

46695 46711 46605 48977 CDS product ribonucleotide reductase subunit 1 46695-46711 4:01:01 CCCGGGCCCTCGCCGCC

46754 46775 46605 48977 CDS product ribonucleotide reductase subunit 1 46754-46775 4:01:01 CCCGCGCCCGGCGCCTCGCGCC

46894 46912 46605 48977 CDS product ribonucleotide reductase subunit 1 46894-46912 4:01:01 GGCGGACCTGGACGCCTGG

46900 46923 46605 48977 CDS product ribonucleotide reductase subunit 1 46900-46923 4:01:01 CCTGGACGCCTGGCTCGCCTCGCC

46953 46970 46605 48977 CDS product ribonucleotide reductase subunit 1 46953-46970 4:01:01 CCGCCGTGCGCCGGCACC

46998 47021 46605 48977 CDS product ribonucleotide reductase subunit 1 46998-47021 5:02:01

GGTTCTGGCGGGAGGCCTACCCGG

47125 47143 46605 48977 CDS product ribonucleotide reductase subunit 1 47125-47143 5:02:01

CCTCGCCGCGGCCGCCGCC

47180 47201 46605 48977 CDS product ribonucleotide reductase subunit 1 47180-47201 4:01:01

GGCTCGGACGCGTGGCCCGAGG

47305 47332 46605 48977 CDS product ribonucleotide reductase subunit 1 47305-47332 6:03:01

CCTGCTGAACCCCCTGCCGCGCACCACC

47367 47384 46605 48977 CDS product ribonucleotide reductase subunit 1 47367-47384 4:01:01

CCCCCATCCTGCTGCGCC

47639 47672 46605 48977 CDS product ribonucleotide reductase subunit 1 47639-47672 5:02:01

CCGGACCTGTTCTTCCAGCGCTACCAGCGGCACC

47713 47731 46605 48977 CDS product ribonucleotide reductase subunit 1 47713-47731 4:01:01

CCGCGCCTCGCACCTGGCC

47820 47842 46605 48977 CDS product ribonucleotide reductase subunit 1 47820-47842 4:01:01

CCTTCCTCATCGTCCGCAGCGCC

47916 47936 46605 48977 CDS product ribonucleotide reductase subunit 1 47916-47936 5:02:01

CCGCCCTCGCCACGTCCAACC

48115 48139 46605 48977 CDS product ribonucleotide reductase subunit 1 48115-48139 4:01:01

GGGCGCCTCGGTCGAGGGCGTGCGG

48190 48226 46605 48977 CDS product ribonucleotide reductase subunit 1 48190-48226 7:04:01

GGCGCTGGACATGGACATGGCCGACCCGGCGGCGCGG

48229 48247 46605 48977 CDS product ribonucleotide reductase subunit 1 48229-48247 4:01:01

CCTCAACGCCGCCATCGCC

48559 48576 46605 48977 CDS product ribonucleotide reductase subunit 1 48559-48576 4:01:01
CCTGCGCCCCAACCTGCC
48624 48644 46605 48977 CDS product ribonucleotide reductase subunit 1 48624-48644 4:01:01
GGGACGCCGTGGCGCGGCTGG
48657 48680 46605 48977 CDS product ribonucleotide reductase subunit 1 48657-48680 4:01:01
GGTCCGTGGCCGCGGCCTTCGGGG
48686 48706 46605 48977 CDS product ribonucleotide reductase subunit 1 48686-48706 4:01:01
CCCGCCGGGCACCCGCTGGCC
48781 48820 46605 48977 CDS product ribonucleotide reductase subunit 1 48781-48820 8:05:02
CCACAGCCAGTCCATGTCCCTCTTCCTGACCGAGCCCGCC
49132 49159 48987 49898 CDS product ribonucleotide reductase subunit 2 49132-49159 6:03:01
CCGCTTCCTCTTCGCCTTCCTCTCCGCC
49458 49474 48987 49898 CDS product ribonucleotide reductase subunit 2 49458-49474 4:01:01
CCTCCTCGTTCGCCTCC
49548 49564 48987 49898 CDS product ribonucleotide reductase subunit 2 49548-49564 4:01:01
CCATCCACACCTCGGCC
49599 49628 48987 49898 CDS product ribonucleotide reductase subunit 2 49599-49628 5:02:01
CCCCGCGCCCCGACGAGGCCCGCATCCACC
49693 49714 48987 49898 CDS product ribonucleotide reductase subunit 2 49693-49714 5:02:01
CCTCCTGCTGGACCTGCCGGCC
50444 50471 50401 51498 CDS product tegument host shutoff protein 50444-50471 7:04:01
GGACGGCCTGGTAGTTCGGGGGCGGGGG
50491 50509 50401 51498 CDS product tegument host shutoff protein 50491-50509 4:01:01 CCCGCCAGCTCCTTCGCCC

50765 50790 50401 51498 CDS product tegument host shutoff protein 50765-50790 5:02:01
CCTCGGGGGCCACCTCGCGGCCCTCC
50801 50833 50401 51498 CDS product tegument host shutoff protein 50801-50833 6:03:01
CCACCTGCTGCACCGAGTCCACGTCCGGCGCCC
50827 50850 50401 51498 CDS product tegument host shutoff protein 50827-50850 4:01:01
GGCGCCCGGTGCAGGTCCGTGTGG
50867 50892 50401 51498 CDS product tegument host shutoff protein 50867-50892 5:02:01
CCAGGAACTCCGAGTAGTCCACCCCC
51015 51031 50401 51498 CDS product tegument host shutoff protein 51015-51031 4:01:01 GGTGTGGTAGAGGTTGG
51044 51066 50401 51498 CDS product tegument host shutoff protein 51044-51066 5:02:01
CCGCCTCCATGTCCGACACGTCC
51151 51172 50401 51498 CDS product tegument host shutoff protein 51151-51172 5:02:01
GGCCGCGGCGGCGCGTCGGCGG
51186 51217 50401 51498 CDS product tegument host shutoff protein 51186-51217 5:02:01
GGCGCGCACGGCCGCGGCCATGATGGCCTTGG
51197 51221 50401 51498 CDS product tegument host shutoff protein 51197-51221 4:01:01
CCGCGGCCATGATGGCCTTGGCGCC
51235 51258 50401 51498 CDS product tegument host shutoff protein 51235-51258 4:01:01
CCGTTCCCGAAGATGCCGCGGTCC
51305 51331 50401 51498 CDS product tegument host shutoff protein 51305-51331 5:02:01
GGCGCAGCAGGCAGCGGGCGGTCGTGG
51395 51439 50401 51498 CDS product tegument host shutoff protein 51395-51439 8:05:02
GGTCGATGGCGATTGGCGTGAGGACCCCCGGCGGCGTGGAGATGG

51647 51688 51628 52782 CDS product DNA polymerase processivity subunit 51647-51688 8:05:02 CCTCGAGGACCTGGACCGCCACCCCACCCACGCGCACCACCC
51793 51819 51628 52782 CDS product DNA polymerase processivity subunit 51793-51819 5:02:01 CCGCCATCGCCCCGCTGGCCGCGCACC
51998 52013 51628 52782 CDS product DNA polymerase processivity subunit 51998-52013 4:01:01 GGTCGACCGGCGGCGG
52214 52238 51628 52782 CDS product DNA polymerase processivity subunit 52214-52238 5:02:01 CCCCCAGGTGGCCAAGCTCGCCGCC
52241 52262 51628 52782 CDS product DNA polymerase processivity subunit 52241-52262 4:01:01 GGCGAAGGGCGCCGCGGCCGGG
52297 52313 51628 52782 CDS product DNA polymerase processivity subunit 52297-52313 4:01:01 CCGTCTCCTCCAGCGCC
52584 52646 51628 52782 CDS product DNA polymerase processivity subunit 52584-52646 16:13:04 CCCGCCGACCCCGCCACCGTGCCCGCCGCCCCCGAGGGCGCCGCCGCCACCGTCGCCGCCGCC
52663 52676 51628 52782 CDS product DNA polymerase processivity subunit 52663-52676 5:02:01 CCGCCGCCCCCGCC
52716 52742 51628 52782 CDS product DNA polymerase processivity subunit 52716-52742 5:02:01 CCCATCGCCAAGCGCCCGCGGGCCGCC
52735 52756 51628 52782 CDS product DNA polymerase processivity subunit 52735-52756 5:02:01 GGGCCGCCTCGGGGGAAGGGGG
52852 52866 52842 53963 CDS product envelope protein UL43 52852-52866 4:01:01 GGGGAACGGAGGGGG
53029 53062 52842 53963 CDS product envelope protein UL43 53029-53062 5:02:01 CCTGCCCATGCCCCTCGTGGACCGGTTCATCCCC
53227 53240 52842 53963 CDS product envelope protein UL43 53227-53240 4:01:01 CCGCCCCGCCTGCC
53260 53279 52842 53963 CDS product envelope protein UL43 53260-53279 5:02:01 GGCCTGCGGCGCGGCGGGGG

53382 53426 52842 53963 CDS product envelope protein UL43 53382-53426 8:05:02 CCTACGTCGCCAGCCGCGCCCGGTTCCTGGCCGCGCCCGCGGACC
53460 53479 52842 53963 CDS product envelope protein UL43 53460-53479 5:02:01 CCGACCCCACCGCCGAGCCC
53502 53527 52842 53963 CDS product envelope protein UL43 53502-53527 5:02:01 CCGTCCCGCACACCCACGCCTACGCC
53562 53576 52842 53963 CDS product envelope protein UL43 53562-53576 5:02:01 CCGCCTCCTCCCTCC
53624 53660 52842 53963 CDS product envelope protein UL43 53624-53660 8:05:02 CCCACCACCGCGGCCGCGGCCGGCGCCGCCGGGTTCC
53635 53672 52842 53963 CDS product envelope protein UL43 53635-53672 7:04:01 GGCCGCGGCCGGCGCCGCCGGGTTCCTGGACGCGGCGG
53740 53782 52842 53963 CDS product envelope protein UL43 53740-53782 7:04:01 CCTCATCCTCGCGGCCATCGTCCTCACGGCCGCCGGCGAGCCC
53792 53819 52842 53963 CDS product envelope protein UL43 53792-53819 6:03:01 CCCATCAGCCTCGCGGCCTCCACCGGCC
53840 53881 52842 53963 CDS product envelope protein UL43 53840-53881 7:04:01 CCACGTCCGCTGCGCCACACCCGCGCGTACCGGCTCGCCGCC
54080 54102 54029 55468 CDS product envelope glycoprotein C 54080-54102 4:01:01 CCATCGCCGCGGCGCCGTCGACC
54130 54143 54029 55468 CDS product envelope glycoprotein C 54130-54143 5:02:01 GGGGGCGGCGGCGG
54169 54204 54029 55468 CDS product envelope glycoprotein C 54169-54204 9:06:02 CCCTCTCCGCCCCCGACCCCCGCGCCCGCCTCGCCC
54226 54243 54029 55468 CDS product envelope glycoprotein C 54226-54243 5:02:01 CCCCCGGTCCCGCCGCCC
54354 54373 54029 55468 CDS product envelope glycoprotein C 54354-54373 5:02:01 GGCGCGGGTGGGGGACGCGG
54389 54414 54029 55468 CDS product envelope glycoprotein C 54389-54414 4:01:01 CCGTCTTCCCGCGCGCCGGGGAGACC
54447 54468 54029 55468 CDS product envelope glycoprotein C 54447-54468 4:01:01 CCGCTCGCCCGACGCCGACCCC
54484 54503 54029 55468 CDS product envelope glycoprotein C 54484-54503 4:01:01 CCCCCGCGCCCGGAGCTCCC

54527 54547 54029 55468 CDS product envelope glycoprotein C 54527-54547 5:02:01 CCGCCAACGCCTCCCTCGCCC

54680 54708 54029 55468 CDS product envelope glycoprotein C 54680-54708 5:02:01
CCGCCAACGGCACCGAGGTCCGGAGCGCC

54720 54738 54029 55468 CDS product envelope glycoprotein C 54720-54738 4:01:01 CCTCCTGTACAGCCAGCCC

54866 54894 54029 55468 CDS product envelope glycoprotein C 54866-54894 5:02:01
CCGCCTTCGTGACCAACAGCACCGTGGCC

54945 54979 54029 55468 CDS product envelope glycoprotein C 54945-54979 5:02:01
GGACGTCCCGGGCCTCGCGGCCGCGGACGCCGCGG

54951 54975 54029 55468 CDS product envelope glycoprotein C 54951-54975 4:01:01 CCCGGGCCTCGCGGCCGCGGACGCC

55049 55079 54029 55468 CDS product envelope glycoprotein C 55049-55079 6:03:01
CCGAGGCCCTGCGCCCCCACGTCTACCACCC

55080 55093 54029 55468 CDS product envelope glycoprotein C 55080-55093 4:01:01 GGCGGCGGTCTCGG

55152 55200 54029 55468 CDS product envelope glycoprotein C 55152-55200 10:07:02
CCTCGCCTGGTCCGACCACGCCGCCGACACCGTCTACCACCTCGGCGCC

55277 55299 54029 55468 CDS product envelope glycoprotein C 55277-55299 5:02:01 CCTGCCGCCTCGAGGGCCTGCCC

55340 55353 54029 55468 CDS product envelope glycoprotein C 55340-55353 4:01:01 CCTCCCCCGCGTCC

55745 55786 55699 56535 CDS product capsid scaffold protein 55745-55786 8:05:02
GGCAGGCCACGGCGGCCGAGCTGGCGTCGATGGTGCCGGCGG

55793 55815 55699 56535 CDS product capsid scaffold protein 55793-55815 5:02:01 CCGCCGGGGCCGAGACCGAGGCC

55812 55885 55699 56535 CDS product capsid scaffold protein 55812-55885 17:14:04
GGCCTGGACGGGCGGCTGGGGGGCCGAGGCGGGCGCGGCCGGGGCGGCGGCCTGAACGGGGGCGGCCGCGGCGG

55897 55948 55699 56535 CDS product capsid scaffold protein 55897-55948 12:09:03
GGAGCCGGAGCGGGGGCGGCCGCGGCGGCGGCGGGCACGGCCACCGGCTGGG

55937 55962 55699 56535 CDS product capsid scaffold protein 55937-55962 6:03:01 CCACCGGCTGGGCCTGCACCACCACC
55963 55989 55699 56535 CDS product capsid scaffold protein 55963-55989 4:01:01 GGCTGCTGCGGGACCACCTGGGCGGGG
56001 56050 55699 56535 CDS product capsid scaffold protein 56001-56050 10:07:02
GGGGGCCGCCAGGCCCTGGAGGCCCGGCAGGCCCGGGAGCGCCGGCACGG
56006 56043 55699 56535 CDS product capsid scaffold protein 56006-56043 6:03:01
CCGCCAGGCCCTGGAGGCCCGGCAGGCCCGGGAGCGCC
56061 56075 55699 56535 CDS product capsid scaffold protein 56061-56075 4:01:01 GGGCGAGGGGGGCGG
56124 56145 55699 56535 CDS product capsid scaffold protein 56124-56145 4:01:01 GGCTGCGTGGTGCGGGTGCGGG
56203 56238 55699 56535 CDS product capsid scaffold protein 56203-56238 6:03:01
GGCGTCGGGTAGCGGAGCTGCTGGGCGCGCAGGTGG
56352 56407 55699 56535 CDS product capsid scaffold protein 56352-56407 13:10:03
GGCGGGGGGCCGGGGCTCGGGCGAGGGCGAGCGGCGGTGGCGGCGGTCCATGGCGG
56429 56457 55699 56535 CDS product capsid scaffold protein 56429-56457 5:02:01
GGGACTCCAGGCTGGGCGCCGGGCGCTGG
56747 56764 55699 57273 CDS product capsid maturation protease 56747-56764 4:01:01 GGTCCGGCGACTCGGCGG
56807 56836 55699 57273 CDS product capsid maturation protease 56807-56836 6:03:01
GGAACGGCGCCACGGCGGCCTCGGGCGAGG
56910 56931 55699 57273 CDS product capsid maturation protease 56910-56931 4:01:01 GGGGGCCTCGCCGGGCGCCAGG
56915 56935 55699 57273 CDS product capsid maturation protease 56915-56935 4:01:01 CCTCGCCGGGCGCCAGGCGCC
56940 56961 55699 57273 CDS product capsid maturation protease 56940-56961 4:01:01 GGACAGGGACGCCGAGGGCAGG
56985 57014 55699 57273 CDS product capsid maturation protease 56985-57014 5:02:01
CCGCTCCTCGTCCGAGAGCCGCATGTCCCC
57025 57049 55699 57273 CDS product capsid maturation protease 57025-57049 4:01:01

GGGCCCACGGCGCGGGCGAGCACGG

57159 57190 55699 57273 CDS product capsid maturation protease 57159-57190 6:03:01

GGGCAGCGGGCCCGCCGGGGGCAGCGCGGCGG

57527 57546 57307 58911 CDS product DNA packaging tegument protein UL25 57527-57546 4:01:01

CCAGCGCCACCAGGCGCACC

57539 57561 57307 58911 CDS product DNA packaging tegument protein UL25 57539-57561 4:01:01

GGCGCACCAGGTTGAGCTGGTGG

57593 57614 57307 58911 CDS product DNA packaging tegument protein UL25 57593-57614 4:01:01

CCAGCGCCAGCGCGGCCAGCCC

57742 57758 57307 58911 CDS product DNA packaging tegument protein UL25 57742-57758 4:01:01

GGCGGCGGCGCGTTGGG

57893 57936 57307 58911 CDS product DNA packaging tegument protein UL25 57893-57936 9:06:02

GGAAGAGGTTCTGGCCGCGGCCCAGGAAGGCGGCCGCGGCGCGG

57941 57969 57307 58911 CDS product DNA packaging tegument protein UL25 57941-57969 5:02:01

CCTCGTCCACGAAGCCCGCGCCGTCGACC

58107 58125 57307 58911 CDS product DNA packaging tegument protein UL25 58107-58125 4:01:01

CCCGCCCTCGGCCAGCGCC

58154 58179 57307 58911 CDS product DNA packaging tegument protein UL25 58154-58179 4:01:01

CCCGCTCCAGCAGGTCCGCGATGCCC

58183 58207 57307 58911 CDS product DNA packaging tegument protein UL25 58183-58207 5:02:01

GGGGCGGCGGCGCCCGCGGCGCCGG

58195 58216 57307 58911 CDS product DNA packaging tegument protein UL25 58195-58216 5:02:01

CCCGCGGCGCCGGCCCCCTGCC

58535 58578 57307 58911 CDS product DNA packaging tegument protein UL25 58535-58578 7:04:01 CCTCGCGGCCCTCGTCCGCGGCCGCGGTCGCCTCGGGCGCCTCC
58592 58605 57307 58911 CDS product DNA packaging tegument protein UL25 58592-58605 4:01:01 CCGCGTCCGCCTCC
58619 58678 57307 58911 CDS product DNA packaging tegument protein UL25 58619-58678 10:07:02 CCGTCTCCTCGAGCACCACGAGCGCCTCGGCCACGCGCTCCACCTGCCGCTCCAGCGGCC
58743 58758 57307 58911 CDS product DNA packaging tegument protein UL25 58743-58758 4:01:01 GGCGGCCGCGCGGCGG
59172 59195 59004 59519 CDS product nuclear protein UL24 59172-59195 7:04:01 GGGGGCACGGCGGCGGCGAGGAGG
59260 59276 59004 59519 CDS product nuclear protein UL24 59260-59276 4:01:01 GGTTCCGGGGGAAGCGG
59312 59330 59004 59519 CDS product nuclear protein UL24 59312-59330 5:02:01 CCCCACCCCCAGCCGCACC
59441 59459 59004 59519 CDS product nuclear protein UL24 59441-59459 4:01:01 GGACGGGTCGCGGCGCAGG
59572 59592 59512 60474 CDS product thymidine kinase 59572-59592 4:01:01 GGGTGATGGCGCTCGGCGGGG
59642 59656 59512 60474 CDS product thymidine kinase 59642-59656 4:01:01 GGACACGGTGGCCGG
60656 60678 60610 62670 CDS product envelope glycoprotein H 60656-60678 4:01:01 GGGCCTCCTGGCCCTCGCGGGGG
60679 60693 60610 62670 CDS product envelope glycoprotein H 60679-60693 4:01:01 CCGCCGCCCTCGCCC
60735 60755 60610 62670 CDS product envelope glycoprotein H 60735-60755 4:01:01 CCCGCGCCCACCGCGGCGCCC
60819 60831 60610 62670 CDS product envelope glycoprotein H 60819-60831 4:01:01 GGCGGGCTGGGGG
60851 60876 60610 62670 CDS product envelope glycoprotein H 60851-60876 4:01:01 CCGCGGGCACCTGCTCGGCCGGTACC
60897 60911 60610 62670 CDS product envelope glycoprotein H 60897-60911 4:01:01 CCCCCGCCCGTCTCC
61022 61047 60610 62670 CDS product envelope glycoprotein H 61022-61047 7:04:01 GGCGGCGGTGTGGCCCGCGGAGGTGG
61199 61228 60610 62670 CDS product envelope glycoprotein H 61199-61228 5:02:01 GGCGCTCGGCTCGGACCTGGAGCCGCCGGG
61214 61248 60610 62670 CDS product envelope glycoprotein H 61214-61248 7:04:01 CCTGGAGCCGCCGGGCCCGCCCGCGGGCCGCTTCC

61268    61290    60610    62670    CDS product envelope glycoprotein H    61268-61290 4:01:01 CCTCTCCGACGGGGCCATGCACC
61352    61383    60610    62670    CDS product envelope glycoprotein H    61352-61383 5:02:01
GGCGGCGCACGCGGCCGCCCTGGCCGCCGTGG
61366    61379    60610    62670    CDS product envelope glycoprotein H    61366-61379 4:01:01 CCGCCCTGGCCGCC
61463    61494    60610    62670    CDS product envelope glycoprotein H    61463-61494 5:02:01
CCGCGACCGGCGCGCCTCGGCGCCGGCGCTCC
61598    61637    60610    62670    CDS product envelope glycoprotein H    61598-61637 7:04:01
CCCGGTGGCGCCCGCGGACCAGCTCCGCGCCGCCTGGACC
61633    61665    60610    62670    CDS product envelope glycoprotein H    61633-61665 6:03:01
GGACCTTCGGCGAGGACCCGGCGCCCCGGCTGG
61795    61823    60610    62670    CDS product envelope glycoprotein H    61795-61823 5:02:01
CCCTGCTCCGCACCACGGCCATGTGCACC
61834    61847    60610    62670    CDS product envelope glycoprotein H    61834-61847 4:01:01 CCGCCGCGGCCGCC
61960    61982    60610    62670    CDS product envelope glycoprotein H    61960-61982 5:02:01 CCACCCTGGCCAACCTGGGCGCC
61967    61991    60610    62670    CDS product envelope glycoprotein H    61967-61991 4:01:01 GGCCAACCTGGGCGCCGCGGCGCGG
62010    62064    60610    62670    CDS product envelope glycoprotein H    62010-62064 14:11:03
GGGGCCCCGGGCGCGGCGGCGGCGACGGACGAGGGGGCGGAGGAGGAGGAGGAGG
62080    62099    60610    62670    CDS product envelope glycoprotein H    62080-62099 4:01:01 CCGCGCCCGAGATCCCCGCC
62155    62175    60610    62670    CDS product envelope glycoprotein H    62155-62175 4:01:01 GGCGCCCGGACTGCGGCCCGG
62268    62287    60610    62670    CDS product envelope glycoprotein H    62268-62287 4:01:01 CCCGAGTCCCAGCACCTGCC
62410    62432    60610    62670    CDS product envelope glycoprotein H    62410-62432 5:02:01 CCGGCGCCAACTCCACCATCCCC
64504    64532    64488    66065    CDS product tegument protein UL21    64504-64532 5:02:01
GGACGATGGAGATGTTGGCGCGGGTCAGG

64555 64582 64488 66065 CDS product tegument protein UL21 64555-64582 5:02:01 GGTACTGGGCCCCGCGGAGGGCCGCGGG
64616 64636 64488 66065 CDS product tegument protein UL21 64616-64636 4:01:01 GGCGCAGACGGCGGACGTGGG
64667 64686 64488 66065 CDS product tegument protein UL21 64667-64686 4:01:01 GGCGACGAGGCCCCGGCCGG
64694 64727 64488 66065 CDS product tegument protein UL21 64694-64727 8:05:02
CCCGTCCAGCACCCGCGCCCCGGCCGCCTCCACC
64780 64812 64488 66065 CDS product tegument protein UL21 64780-64812 7:04:01
CCGCCAGCTCCAGGCCCGTCGCCGCGTCCTCCC
64840 64853 64488 66065 CDS product tegument protein UL21 64840-64853 4:01:01 CCCCCGCCGGGTCC
64936 64965 64488 66065 CDS product tegument protein UL21 64936-64965 5:02:01
GGTTGACGGCGTGGGCCACGGCGCTCGCGG
64966 65008 64488 66065 CDS product tegument protein UL21 64966-65008 11:08:02
CCCGAGAGCCGCCGTGGCCGCCCCCTCCTCCCCCTTCTCCTCC
65094 65123 64488 66065 CDS product tegument protein UL21 65094-65123 5:02:01
GGCAGGTGCCAGCGGGCCAGGATGAACTGG
65230 65250 64488 66065 CDS product tegument protein UL21 65230-65250 4:01:01 CCGAGGCGCCCGCGGCCCGCC
65431 65466 64488 66065 CDS product tegument protein UL21 65431-65466 7:04:01
CCGCGCGCCCGCCCTTCACCACCACGGCCGTGGGCC
65627 65669 64488 66065 CDS product tegument protein UL21 65627-65669 8:05:02
GGCGGGCTCGGCCAGGAGCTCCAGGACGCGGTCCTGCTGGCGG
65924 65952 64488 66065 CDS product tegument protein UL21 65924-65952 4:01:01
CCCGAACTTGCCGATCTCCCGCGAGCGCC
66437 66461 66172 66657 CDS product envelope protein UL20 66437-66461 5:02:01 GGCGCGGAACGTGGCCCGGGACCGG
66451 66475 66172 66657 CDS product envelope protein UL20 66451-66475 4:01:01 CCCGGGACCGGATGCCGCTCTCGCC

66476 66519 66172 66657 CDS product envelope protein UL20 66476-66519 9:06:02
GGCGCAGCAGGCGGCGCTGGGGCTCCTGGCGGCGGCGCGGCTGG
66751 66765 66744 70736 CDS product major capsid protein 66751-66765 4:01:01 CCCGGCCATCCTGCC
66922 66941 66744 70736 CDS product major capsid protein 66922-66941 4:01:01 GGAGCTCGGGCTCTCGGTGG
67034 67054 66744 70736 CDS product major capsid protein 67034-67054 5:02:01 CCGCACCCGGCCGACCAGCCC
67078 67105 66744 70736 CDS product major capsid protein 67078-67105 5:02:01 CCTCGATCGCCGCTCCCTCAACGCCGCC
67324 67346 66744 70736 CDS product major capsid protein 67324-67346 4:01:01 GGGCCGGCTCGCGGACCGCGTGG
67426 67457 66744 70736 CDS product major capsid protein 67426-67457 6:03:01
GGACGCGGTCCTGGACCGGCTCTCGGACCTGG
67529 67551 66744 70736 CDS product major capsid protein 67529-67551 4:01:01 GGGGTGCTGGTGACCACGGCCGG
67657 67676 66744 70736 CDS product major capsid protein 67657-67676 4:01:01 GGCGCTGGTGATGGGCAAGG
67792 67814 66744 70736 CDS product major capsid protein 67792-67814 4:01:01 CCGCCTGGTGTTCCTCGAGGCCC
67907 67919 66744 70736 CDS product major capsid protein 67907-67919 4:01:01 CCGGCCGCCGACC
67949 67971 66744 70736 CDS product major capsid protein 67949-67971 5:02:01 CCCACGCCGGGCCTCCCGGACCC
68046 68075 66744 70736 CDS product major capsid protein 68046-68075 6:03:01
CCATGGGCACCCTCTGCCACCCCTCCTTCC
68238 68258 66744 70736 CDS product major capsid protein 68238-68258 6:03:01 CCGCGCCGCCGGCCGCCGACC
68270 68291 66744 70736 CDS product major capsid protein 68270-68291 4:01:01 CCCGGCAACGCCGACCTGCGCC
68341 68363 66744 70736 CDS product major capsid protein 68341-68363 4:01:01 CCCCGGGCCCTTCGCCGTGCCCC
68470 68514 66744 70736 CDS product major capsid protein 68470-68514 9:06:02
GGACCGGCATCGGCTGGCCCCGGCCACGGTGGCCGCGGTGCGCGG
68517 68539 66744 70736 CDS product major capsid protein 68517-68539 4:01:01 CCTTCCGCGACGCCAACTACCCC
69005 69017 66744 70736 CDS product major capsid protein 69005-69017 4:01:01 GGCGGCGGCCTGG

69046 69073 66744 70736 CDS product major capsid protein 69046-69073 5:02:01 CCAGCCCCTGCACCCGCACCACGACGCC

69205 69224 66744 70736 CDS product major capsid protein 69205-69224 5:02:01 GGACGAGGAGGTGGGCACGG

69229 69265 66744 70736 CDS product major capsid protein 69229-69265 6:03:01
CCCCCGGCACCCGCTGCACCCGCGCAACCTCGTGCCC

69376 69422 66744 70736 CDS product major capsid protein 69376-69422 9:06:02
GGCCTCGGTGGCGCCGGACGCGGGCATGGCCACGGTGGCCACGCGGG

69471 69490 66744 70736 CDS product major capsid protein 69471-69490 4:01:01 CCTACCAGCCCAACGACGCC

69543 69565 66744 70736 CDS product major capsid protein 69543-69565 5:02:01 CCTGCGCCGACCACCTCGGCGCC

69745 69764 66744 70736 CDS product major capsid protein 69745-69764 4:01:01 CCCCGTGGCCTTCACCCACC

69949 69971 66744 70736 CDS product major capsid protein 69949-69971 4:01:01 GGACCTGGGCGTGGGCTTCACGG

70021 70042 66744 70736 CDS product major capsid protein 70021-70042 4:01:01 CCTGCCGCAGAACCTCTTCGCC

70107 70132 66744 70736 CDS product major capsid protein 70107-70132 5:02:01 CCGGCAACCGCCTGGCGCCCGTGCCC

70151 70175 66744 70736 CDS product major capsid protein 70151-70175 4:01:01 CCCCAGGTGCCCGCGGGCCTGGCCC

70156 70180 66744 70736 CDS product major capsid protein 70156-70180 4:01:01 GGTGCCCGCGGGCCTGGCCCGCGGG

70271 70294 66744 70736 CDS product major capsid protein 70271-70294 5:02:01 GGCGAGGTGCACGGCGAGGAGGGG

70306 70360 66744 70736 CDS product major capsid protein 70306-70360 11:08:02
CCACAGCCACGCGGACCCCGCGCACCCCCACCGCGCCACCGCCAATCCCTGGGCC

70476 70489 66744 70736 CDS product major capsid protein 70476-70489 4:01:01 GGGGGCTGGCGCGG

70509 70521 66744 70736 CDS product major capsid protein 70509-70521 4:01:01 CCGCCGCCTCGCC

70953 70979 70896 71783 CDS product capsid triplex subunit 2 70953-70979 4:01:01
GGTGCGAGGGCCGCGTGGTGTTTCTGG

71044 71062 70896 71783 CDS product capsid triplex subunit 2 71044-71062 4:01:01 GGCCATGGCTGACCGGCGG

71120 71137 70896 71783 CDS product capsid triplex subunit 2 71120-71137 4:01:01 GGCGTCGGCGGCCGCGGG

71188 71208 70896 71783 CDS product capsid triplex subunit 2 71188-71208 4:01:01 CCACGTGTGCCTCGTCCCGCC

71302 71315 70896 71783 CDS product capsid triplex subunit 2 71302-71315 4:01:01 GGCGCGGGTGGTGG

71383 71411 70896 71783 CDS product capsid triplex subunit 2 71383-71411 6:03:01 CCGGCGCTACCAGCTGCCCCCGCCGCACC

71518 71532 70896 71783 CDS product capsid triplex subunit 2 71518-71532 4:01:01 GGGGGCGCAGGACGG

71597 71621 70896 71783 CDS product capsid triplex subunit 2 71597-71621 6:03:01 CCCCCGCCGCCGCTGCCGCAGGACC

71647 71662 70896 71783 CDS product capsid triplex subunit 2 71647-71662 4:01:01 GGCGCTGGAGGCCTGG

71734 71752 70896 71783 CDS product capsid triplex subunit 2 71734-71752 5:02:01 CCCCCCGACCGTCCCCCCC

71753 71777 70896 71783 CDS product capsid triplex subunit 2 71753-71777 6:03:01 GGCGAGAAGGCCGCGGTGGTGGAGG

72028 72048 71979 77065 CDS product DNA packaging terminase subunit 1 72028-72048 4:01:01 GGGCCACGGCGAGGTCGTCGG

72087 72107 71979 77065 CDS product DNA packaging terminase subunit 1 72087-72107 5:02:01 CCGGCCCCCGAGGCCGTCTCC

72375 72398 71979 77065 CDS product DNA packaging terminase subunit 1 72375-72398 4:01:01 CCCACGATGGCCACGGCCGAGTCC

72411 72434 71979 77065 CDS product DNA packaging terminase subunit 1 72411-72434 4:01:01 CCCTCGACGGCCACGCGCACCGCC

72576 72598 71979 77065 CDS product DNA packaging terminase subunit 1 72576-72598 4:01:01 CCCACGACGGCCACGCCGGTGCC

72584 72603 71979 77065 CDS product DNA packaging terminase subunit 1 72584-72603 4:01:01 GGCCACGCCGGTGCCGGAGG

72653 72698 71979 77065 CDS product DNA packaging terminase subunit 1 72653-72698 9:06:02
GGCCAGCAGGCGGCGGTTGGCCACCGAGGAGGGGCGGTAGAGCAGG
72797 72825 71979 77065 CDS product DNA packaging terminase subunit 1 72797-72825 5:02:01
GGCCGTGGTGCGCGTGGCGGCGTCCATGG
72863 72876 71979 77065 CDS product DNA packaging terminase subunit 1 72863-72876 4:01:01 GGCGGTGGCGTCGG
72938 72993 71979 77065 CDS product DNA packaging terminase subunit 1 72938-72993 11:08:02
GGTGCCGCGGAGGTTGGTGAGGAAGCTGGTGCTGGCCTTGCCGGTGTTGGTGGAGG
73036 73058 71979 77065 CDS product DNA packaging terminase subunit 1 73036-73058 4:01:01
GGATGGTCTGCACGGCATCGGGG
73239 73252 73166 74959 CDS product DNA packaging tegument protein UL17 73239-73252 4:01:01 CCCGGACCCCCTCC
73272 73294 73166 74959 CDS product DNA packaging tegument protein UL17 73272-73294 4:01:01
CCCGCTCGCGCCCTTCGCCGCCC
73572 73599 73166 74959 CDS product DNA packaging tegument protein UL17 73572-73599 5:02:01
CCTGGAGCGCCTGTGCCGCCAGTTCGCC
73616 73649 73166 74959 CDS product DNA packaging tegument protein UL17 73616-73649 7:04:01
GGCGCGTGGCCCCGGCGGTCGCGGCCGCGGGGGG
73862 73936 73166 74959 CDS product DNA packaging tegument protein UL17 73862-73936 16:13:04
GGCGCGGCGGCGGAGGAGGCGGCGCGGGGGCGCTCGAGGCGGCGGCGACGGTGCCGGCGCCCGCGGCGCGCGAGG
73984 74000 73166 74959 CDS product DNA packaging tegument protein UL17 73984-74000 4:01:01
CCCCAGGACCCCCGGCC
74103 74118 73166 74959 CDS product DNA packaging tegument protein UL17 74103-74118 4:01:01 CCGCCACTACCAGACC
74162 74184 73166 74959 CDS product DNA packaging tegument protein UL17 74162-74184 5:02:01
GGGGCGGCCGGCGCGGGCCCCGG

74259 74286 73166 74959 CDS product DNA packaging tegument protein UL17 74259-74286 7:04:01 GGAGGCGCGGCGGGAGCTGGTGCGGCGG

74405 74435 73166 74959 CDS product DNA packaging tegument protein UL17 74405-74435 5:02:01 CCGGCGTCCGCGAGTCCGTGCGCCGCGAGCC

74466 74491 73166 74959 CDS product DNA packaging tegument protein UL17 74466-74491 5:02:01 CCGCCCGCGCGACCGCGAGGCCGTCC

74556 74579 73166 74959 CDS product DNA packaging tegument protein UL17 74556-74579 4:01:01 GGCGCGGGAGATGGTCGACTCGGG

74604 74625 73166 74959 CDS product DNA packaging tegument protein UL17 74604-74625 5:02:01 CCTCGCCGCGCGCGCCACCGCC

74692 74746 73166 74959 CDS product DNA packaging tegument protein UL17 74692-74746 9:06:02 GGCGGGGAGGTTATAAGGGTGACGCGGGCCCCGCCGGCCGTGGCAGTGGAGCCGG

74987 75012 74986 75972 CDS product tegument protein UL16 74987-75012 6:03:01 GGAGGTGGCCGCGGCCCTGACGGAGG

75089 75125 74986 75972 CDS product tegument protein UL16 75089-75125 7:04:01 CCTGGCCGCCGTGGCGCCCGGCGCCGCGACCGTCGCC

75226 75246 74986 75972 CDS product tegument protein UL16 75226-75246 4:01:01 CCGTGACCACGACCACGCTCC

75306 75405 74986 75972 CDS product tegument protein UL16 75306-75405 19:16:04 CCCGCCGACCTGCCCGTCGCCGAGCCCGTGCCCGCCGCGCCGGCGGGCCGCCTGGACCTGGACGCCGCCGAGCCCGTGGCCGCGGCGCCGGCGGGCCGCC

75347 75366 74986 75972 CDS product tegument protein UL16 75347-75366 4:01:01 GGCGGGCCGCCTGGACCTGG

75383 75400 74986 75972 CDS product tegument protein UL16 75383-75400 4:01:01 GGCCGCGGCGCCGGCGGG

75416 75448 74986 75972 CDS product tegument protein UL16 75416-75448 6:03:01 GGTCCTGGCCCCCGGCGCGTGGTGGGCGCGCGG

75515 75536 74986 75972 CDS product tegument protein UL16 75515-75536 4:01:01 CCACCTCGGCGCCGTGCTCGCC

75621 75678 74986 75972 CDS product tegument protein UL16 75621-75678 10:07:02
CCGCACCCCGACGGGGCCGCCGCGCCGTGCCTGTGCGCCGCGCCGTGCCTGTGGCGCC

75673 75693 74986 75972 CDS product tegument protein UL16 75673-75693 4:01:01 GGCGCCAGGCGGACAAGCGGG

75849 75876 74986 75972 CDS product tegument protein UL16 75849-75876 6:03:01 GGCTGGCGCCTGGTGGCCCTGGACCCGG

75929 75950 74986 75972 CDS product tegument protein UL16 75929-75950 5:02:01 CCCCCCCGCCCGCGCCATCCCC

76095 76120 71979 77065 CDS product DNA packaging terminase subunit 1 76095-76120 4:01:01
GGCACCAGCGGCGCAGGCGCGCGTGG

76135 76160 71979 77065 CDS product DNA packaging terminase subunit 1 76135-76160 4:01:01
GGGCTCCGTGGCCTTGCGGATGTGGG

76176 76193 71979 77065 CDS product DNA packaging terminase subunit 1 76176-76193 4:01:01
GGATGCCGCGGAAGGTGG

76329 76354 71979 77065 CDS product DNA packaging terminase subunit 1 76329-76354 6:03:01
GGTAGGCGGTGGCGCGCTCGGCGTGG

76419 76477 71979 77065 CDS product DNA packaging terminase subunit 1 76419-76477 13:10:03
CCAGCGTGCCGCGCCCGCCGCCGCCTTTGCCCTCGCCCCCGCCTCCATCGTGGCCCTCC

76502 76572 71979 77065 CDS product DNA packaging terminase subunit 1 76502-76572 12:09:03
CCGGGCGCGCCCTTCGCGCCGCCCCGGGCCCTCTCGTCCTTCTCCTCTTCCCCCCCATCCTCCTCGTCGCC

76581 76611 71979 77065 CDS product DNA packaging terminase subunit 1 76581-76611 8:05:02
CCCCGGGCCCCGCCGCCGCCGCCGCCGTCCC

76614 76640 71979 77065 CDS product DNA packaging terminase subunit 1 76614-76640 5:02:01
GGGCGGCCGTCGCGGCGTCGCGGAAGG

76680 76700 71979 77065 CDS product DNA packaging terminase subunit 1 76680-76700 5:02:01
GGAGCTGGCGGAAGGCCCCGG

76716 76726 71979 77065 CDS product DNA packaging terminase subunit 1 76716-76726 4:01:01 CCACCGCCGCC
76749 76762 71979 77065 CDS product DNA packaging terminase subunit 1 76749-76762 4:01:01 GGTGGAAGGCCAGG
76817 76834 71979 77065 CDS product DNA packaging terminase subunit 1 76817-76834 4:01:01
GGGTCCACGGCGGCGCGG
76913 76930 71979 77065 CDS product DNA packaging terminase subunit 1 76913-76930 5:02:01
CCGACCGCCGGGACCACC
76935 76958 71979 77065 CDS product DNA packaging terminase subunit 1 76935-76958 5:02:01
GGTGGCACTGTGGGGCGGCGATGG
77070 77099 77064 77543 CDS product tegument protein UL14 77070-77099 5:02:01
CCTCGGACCGCCGGGAGCGCCGCGTGCGCC
77193 77225 77064 77543 CDS product tegument protein UL14 77193-77225 6:03:01
GGGCCTTTATGGCGGCCAAACAGGCCCGGCGGG
77244 77270 77064 77543 CDS product tegument protein UL14 77244-77270 5:02:01 GGCTGGCCGCCCGGGTGGAGTCTGTGG
77293 77315 77064 77543 CDS product tegument protein UL14 77293-77315 4:01:01 GGCCCGCGTGGAAGCCCAGGCGG
77307 77330 77064 77543 CDS product tegument protein UL14 77307-77330 4:01:01 CCCAGGCGGCCGTCCGCGGGGTCC
77324 77372 77064 77543 CDS product tegument protein UL14 77324-77372 9:06:02
GGGGTCCTGGACAGGCACCGGCGGTTCACGCGGGCGGACTTTGCGGAGG
77401 77416 77064 77543 CDS product tegument protein UL14 77401-77416 4:01:01 GGCCGGCGAGGACCGG
77443 77462 77064 77543 CDS product tegument protein UL14 77443-77462 5:02:01 GGACTGGGCCGGCGGCGGGG
77473 77498 77064 77543 CDS product tegument protein UL14 77473-77498 7:04:01 GGACGAGGGGGAGGAGGCGGACGAGG
77514 77537 77064 77543 CDS product tegument protein UL14 77514-77537 7:04:01 GGCTGCTGGAGGAGGCGGAGGAGG
77563 77575 77513 78709 CDS product tegument serine/threonine protein kinase 77563-77575 4:01:01
CCGCCCATCCACC

77600 77613 77513 78709 CDS product tegument serine/threonine protein kinase 77600-77613 4:01:01
CCATCGCCGCCGCC
77614 77654 77513 78709 CDS product tegument serine/threonine protein kinase 77614-77654 7:04:01
GGCGGCGACGGGGACGGGGACGAGGCCTCCCGGCTCCTGGG
77639 77656 77513 78709 CDS product tegument serine/threonine protein kinase 77639-77656 4:01:01
CCTCCCGGCTCCTGGGCC
77675 77696 77513 78709 CDS product tegument serine/threonine protein kinase 77675-77696 5:02:01
CCCCCTACCTGATCCCGCGCCC
77706 77725 77513 78709 CDS product tegument serine/threonine protein kinase 77706-77725 4:01:01
CCTCGCCGTGCCGGACGACC
77763 77786 77513 78709 CDS product tegument serine/threonine protein kinase 77763-77786 4:01:01
GGCCGTCGGGGCCGGATCGTACGG
77865 77878 77513 78709 CDS product tegument serine/threonine protein kinase 77865-77878 4:01:01
GGCCGCGGAGGAGG
78201 78223 77513 78709 CDS product tegument serine/threonine protein kinase 78201-78223 4:01:01
CCCGCGCTTCCGCCTCGCGCGCC
78333 78356 77513 78709 CDS product tegument serine/threonine protein kinase 78333-78356 7:04:01
CCCGCCGCCGCCGGGCCCCCTGCC
78525 78546 77513 78709 CDS product tegument serine/threonine protein kinase 78525-78546 5:02:01
CCCCTCCGCCGAGGCCCTCGCC
78561 78578 77513 78709 CDS product tegument serine/threonine protein kinase 78561-78578 4:01:01
GGAGGAGCTGGCCGCCGG
78606 78623 77513 78709 CDS product tegument serine/threonine protein kinase 78606-78623 4:01:01

CCCCGGCAACCAGCCCCC

78654 78680 77513 78709 CDS product tegument serine/threonine protein kinase 78654-78680 6:03:01 GGCCCGGTTCTGCGGCGAGGATGGCGG

78717 78740 78675 80126 CDS product deoxyribonuclease 78717-78740 4:01:01 CCGCGCGCACCTTCCTGCGGTTCC

78763 78785 78675 80126 CDS product deoxyribonuclease 78763-78785 5:02:01 GGCCGGCGGCGCGGCGCCGCTGG

78832 78868 78675 80126 CDS product deoxyribonuclease 78832-78868 7:04:01 CCACGGCCTCGCCGCCCCCGACGCGGCCGCGGCGGCC

78856 78876 78675 80126 CDS product deoxyribonuclease 78856-78876 5:02:01 GGCCGCGGCGGCCTTTGGCGG

78883 78898 78675 80126 CDS product deoxyribonuclease 78883-78898 4:01:01 CCCGCCGCCCGCGCCC

78940 78950 78675 80126 CDS product deoxyribonuclease 78940-78950 4:01:01 GGTGGAGGCGG

79032 79053 78675 80126 CDS product deoxyribonuclease 79032-79053 4:01:01 CCGACGGCCCGCGCTTCCCGCC

79137 79189 78675 80126 CDS product deoxyribonuclease 79137-79189 9:06:02 CCCGCGCCGCCGTCGCGGCCCTGTACGCCGAGGCGCCCACGCCGGACCTGCCC

79190 79235 78675 80126 CDS product deoxyribonuclease 79190-79235 9:06:02 GGGGCGATCGCGGGCGGCGACGGGGGCGGGGACGGGGCGAAGGAGG

79265 79283 78675 80126 CDS product deoxyribonuclease 79265-79283 5:02:01 CCACCGCCGGGCCACGACC

79319 79349 78675 80126 CDS product deoxyribonuclease 79319-79349 5:02:01 GGCATGGTCGGGGCCTCGCTGGACCTGCTGG

79525 79545 78675 80126 CDS product deoxyribonuclease 79525-79545 4:01:01 CCTGTACTCCATCGCCCGGCC

79564 79598 78675 80126 CDS product deoxyribonuclease 79564-79598 6:03:01 GGGCGCCCCGGGGCCCGGCGAGGCGCTGGCGACGG

79611 79631 78675 80126 CDS product deoxyribonuclease 79611-79631 4:01:01 GGCGACGCGGGGGCGCCGAGG

79626 79649 78675 80126 CDS product deoxyribonuclease 79626-79649 4:01:01 CCGAGGACGCCCCGCCGACGCGCC

79729 79745 78675 80126 CDS product deoxyribonuclease 79729-79745 4:01:01 GGTGGACGGCCGTCAGG

79861 79894 78675 80126 CDS product deoxyribonuclease 79861-79894 8:05:02 CCCCGACCGCCCCGCGCGCCCCCACCTGGCCACC
79906 79918 78675 80126 CDS product deoxyribonuclease 79906-79918 4:01:01 CCGCCGCCGCCCC
79964 79984 78675 80126 CDS product deoxyribonuclease 79964-79984 5:02:01 CCGCCCGCCTGCGCCGTGCCC
80020 80042 78675 80126 CDS product deoxyribonuclease 80020-80042 4:01:01 GGAGGCGTTCGAGGACCTGCGGG
80200 80230 80084 80275 CDS product myristylated tegument protein 80200-80230 5:02:01 CCCGGCCCCGTGCGCCCCAAGGCCCCGTACC
80873 80900 80754 81935 CDS product envelope glycoprotein M 80873-80900 6:03:01 CCGCCGGGCCGTCGTCCGCGCCTTGTCC
80917 80957 80754 81935 CDS product envelope glycoprotein M 80917-80957 7:04:01 GGCTGCCCCGGGCGCGGTGGGCGATGCAGGCGCGGACCAGG
81202 81225 80754 81935 CDS product envelope glycoprotein M 81202-81225 5:02:01 CCAGGGCGGCCTCCGCCGTCTTCC
81272 81304 80754 81935 CDS product envelope glycoprotein M 81272-81304 7:04:01 CCCGACCACGGCCGCGCCCAGGCCGACCGCGCC
81349 81378 80754 81935 CDS product envelope glycoprotein M 81349-81378 6:03:01 GGCGCAGCGGGGGGCTGGCCGCCAGGGCGG
81442 81462 80754 81935 CDS product envelope glycoprotein M 81442-81462 5:02:01 GGGTGATGTAGGCGGCGGCGG
81500 81516 80754 81935 CDS product envelope glycoprotein M 81500-81516 4:01:01 GGCCTGGAGGGTCCAGG
81542 81566 80754 81935 CDS product envelope glycoprotein M 81542-81566 4:01:01 CCCCGGGGCGGCCACCGTCGACGCC
81595 81638 80754 81935 CDS product envelope glycoprotein M 81595-81638 9:06:02 CCAGCCGGCCGGCCTCGCGCCGCAGCACCACGGCCCCCGCGACC
82337 82350 81934 84465 CDS product DNA replication origin-binding helicase 82337-82350 4:01:01 CCGGCCCTACCGCC
82643 82676 81934 84465 CDS product DNA replication origin-binding helicase 82643-82676 6:03:01

CCGCCGCTGCCTCGTGCTCCGGCACCTCGGGGCC

82663 82751 81934 84465 CDS product DNA replication origin-binding helicase 82663-82751 17:14:04 GGCACCTCGGGGCCGAGGTCGCGGCGGGCGCGGCGGGCGCGCGGGAGGACGGCGGCGGCGACGGGAGCGAGGACGCGGCGCGGGCCGGG

82769 82812 81934 84465 CDS product DNA replication origin-binding helicase 82769-82812 13:10:03 GGCGGCGGCGACGACGGCGGTGGAGGCGGCGGGGGCGGCGGGGG

84466 84479 84462 86513 CDS product helicase-primase subunit 84466-84479 4:01:01 GGCCGCGGCGGGGG

84508 84534 84462 86513 CDS product helicase-primase subunit 84508-84534 5:02:01 GGCCTGGACGGAGCCCGGCGCGCCGGG

84610 84650 84462 86513 CDS product helicase-primase subunit 84610-84650 9:06:02 GGCGGCGCCGTGGACGGGCTCGGCGGCCGCGGTGGCCGCGG

84668 84728 84462 86513 CDS product helicase-primase subunit 84668-84728 12:09:03 GGCACGGGGGCGCTGGCGGACCCGGCGCTGTGGCGCGGCGCGCACGGCGCGGTCATGGCGG

84729 84747 84462 86513 CDS product helicase-primase subunit 84729-84747 4:01:01 CCCTGCGCCGCGCCCTCCC

84820 84842 84462 86513 CDS product helicase-primase subunit 84820-84842 6:03:01 GGCGGGCGGCGGCGGCGAAGAGG

84851 84885 84462 86513 CDS product helicase-primase subunit 84851-84885 8:05:02 GGGGAGGGCGGCGAGGAGGACGAGGACGAGGCCGG

84990 85015 84462 86513 CDS product helicase-primase subunit 84990-85015 4:01:01 CCATGGCCGGCTGCGCCGCGCTCTCC

85126 85157 84462 86513 CDS product helicase-primase subunit 85126-85157 6:03:01 GGTGCTGCGGCCCGCGGGCCGGCGCGTGGCGG

85275 85313 84462 86513 CDS product helicase-primase subunit 85275-85313 6:03:01 CCCCGCCCGTCTTCGCCTTCCTGGGGCCCGAGCTCGCCC

85375 85407 84462 86513 CDS product helicase-primase subunit 85375-85407 7:04:01

GGTGGCCGCGGCCGCGCCGGAGGCGGTCCGCGG
85425 85444 84462 86513 CDS product helicase-primase subunit 85425-85444 4:01:01 GGCTCACGGACGGGCTGTGG
85459 85483 84462 86513 CDS product helicase-primase subunit 85459-85483 5:02:01
CCGCGCCTTCCACGCCCTCGGGCCC
85552 85582 84462 86513 CDS product helicase-primase subunit 85552-85582 5:02:01
CCCGTGCGCCCTCCGCGGGGCCTGGCTGGCC
85662 85691 84462 86513 CDS product helicase-primase subunit 85662-85691 4:01:01
GGGCGCGCGCGGTCAAGCCGGCGCTCGTGG
85777 85802 84462 86513 CDS product helicase-primase subunit 85777-85802 6:03:01
GGCGGCCGCGGCCGAGGCCGGCCTGG
85782 85799 84462 86513 CDS product helicase-primase subunit 85782-85799 4:01:01 CCGCGGCCGAGGCCGGCC
85822 85845 84462 86513 CDS product helicase-primase subunit 85822-85845 4:01:01 GGACGGCTTCTGGGGCGTGCTCGG
85858 85874 84462 86513 CDS product helicase-primase subunit 85858-85874 4:01:01 GGCCGCGCCGGAGGCGG
85875 85891 84462 86513 CDS product helicase-primase subunit 85875-85891 5:02:01 CCGCCGCCGCCGCGGCC
85915 85949 84462 86513 CDS product helicase-primase subunit 85915-85949 6:03:01
GGCCGCCGCGGCGCGGCACCTGGCCGACGCGGGGG
85998 86020 84462 86513 CDS product helicase-primase subunit 85998-86020 5:02:01 CCCTGGCCTGGTCCTGCCACGCC
86025 86051 84462 86513 CDS product helicase-primase subunit 86025-86051 5:02:01
GGCTGCGGCGGCGCGAGGGCCCCGCGG
86086 86101 84462 86513 CDS product helicase-primase subunit 86086-86101 4:01:01 CCGCGCCGCCAAGGCC
86125 86153 84462 86513 CDS product helicase-primase subunit 86125-86153 4:01:01
GGCCGTCGAGGGCCGCGCGGACGCCGAGG
86148 86167 84462 86513 CDS product helicase-primase subunit 86148-86167 4:01:01 CCGAGGCCGCGCGCGCCGCC

86190 86212 84462 86513 CDS product helicase-primase subunit 86190-86212 4:01:01 CCTTCGCCTCGCGCCACGACCCC
86238 86251 84462 86513 CDS product helicase-primase subunit 86238-86251 4:01:01 CCCTCGCCCCCGCC
86350 86368 84462 86513 CDS product helicase-primase subunit 86350-86368 4:01:01 CCCCCCGCTGATCCTGCCC
86451 86469 84462 86513 CDS product helicase-primase subunit 86451-86469 4:01:01 CCGGCGACGCCCCGCCGCC
86740 86764 86679 87479 CDS product tegument protein UL7 86740-86764 4:01:01 GGTACACGGCCTCGGCCATCTCGGG
86888 86904 86679 87479 CDS product tegument protein UL7 86888-86904 4:01:01 GGGCGGGGCCCGGGGGG
86914 86938 86679 87479 CDS product tegument protein UL7 86914-86938 4:01:01 GGCGCTGGGCGATGGGGTAGTGGGG
87061 87074 86679 87479 CDS product tegument protein UL7 87061-87074 4:01:01 CCAGCCACCGGCCC
87097 87107 86679 87479 CDS product tegument protein UL7 87097-87107 4:01:01 CCGCCTCCGCC
87159 87193 86679 87479 CDS product tegument protein UL7 87159-87193 6:03:01 GGGTCCTCGGGGCGCACCAGGGCCAGGCGGTGCGG
87232 87251 86679 87479 CDS product tegument protein UL7 87232-87251 4:01:01 CCTCGGCGGCCGTGACCACC
87268 87299 86679 87479 CDS product tegument protein UL7 87268-87299 7:04:01 GGAAGGCCTCCCCGGCGAGGCAGCGGCGGCGG
87404 87435 86679 87479 CDS product tegument protein UL7 87404-87435 5:02:01 CCCGGGCACCTCGCGCACCTCGCACACCAGCC
87608 87633 87370 89301 CDS product capsid portal protein 87608-87633 5:02:01 GGCTGGTGATGAGGAAGCCGAGGTGG
87884 87906 87370 89301 CDS product capsid portal protein 87884-87906 5:02:01 GGCCGAGGTCCCGCGGCGGCTGG
87930 87972 87370 89301 CDS product capsid portal protein 87930-87972 8:05:02 CCCCCCGGCGGGGCCGGCGGCGCCGGTCCCGTCCGCGCCCGCC
87936 87955 87370 89301 CDS product capsid portal protein 87936-87955 5:02:01 GGCGGGGCCGGCGGCGCCGG
88045 88059 87370 89301 CDS product capsid portal protein 88045-88059 4:01:01 CCCCCGTTGTCCGCC
88131 88144 87370 89301 CDS product capsid portal protein 88131-88144 4:01:01 GGAGGTGCGGAAGG

88165 88188 87370 89301 CDS product capsid portal protein 88165-88188 4:01:01 CCGGCGCCGTGGTGCCCGAAGCCC
88316 88343 87370 89301 CDS product capsid portal protein 88316-88343 6:03:01 CCGCGGCCGAGCCCGCCGGGTCCTCGCC
88355 88377 87370 89301 CDS product capsid portal protein 88355-88377 4:01:01 CCACGGCGCCGGCCGGGTGCCCC
88536 88566 87370 89301 CDS product capsid portal protein 88536-88566 4:01:01 CCAGAGCACCAGGCACTCCCCGCGCTCGCCC
88673 88695 87370 89301 CDS product capsid portal protein 88673-88695 5:02:01 CCACCTCGTCCAGGCCCTGGCCC
88758 88772 87370 89301 CDS product capsid portal protein 88758-88772 4:01:01 CCGCCGCTTCCCCCC
88781 88802 87370 89301 CDS product capsid portal protein 88781-88802 4:01:01 CCCGGCGCACCGGGACCACGCC
88937 88955 87370 89301 CDS product capsid portal protein 88937-88955 4:01:01 GGCGCTCGGCCACGGCGGG
88958 89000 87370 89301 CDS product capsid portal protein 88958-89000 7:04:01 CCTCGTCGCCGAAGCGCCGCGCCAGCGCCTCGGCGCCGAGGCC
89018 89040 87370 89301 CDS product capsid portal protein 89018-89040 5:02:01 CCATCCAGTCCCGGGCCACGTCC
89097 89110 87370 89301 CDS product capsid portal protein 89097-89110 4:01:01 GGCGGCCTCGGTGG
89184 89208 87370 89301 CDS product capsid portal protein 89184-89208 5:02:01 GGTGCGCGGCGTGGGGTGGATGAGG
89240 89258 87370 89301 CDS product capsid portal protein 89240-89258 5:02:01 CCTCCTCCGCGGCGCCGCC
89326 89346 89300 91804 CDS product helicase-primase helicase subunit 89326-89346 5:02:01 CCGCGCGCCACCAACGCCACC
89463 89494 89300 91804 CDS product helicase-primase helicase subunit 89463-89494 6:03:01 GGCGGACAACCCGGTGGATATAGAGGCGCTGG
89865 89899 89300 91804 CDS product helicase-primase helicase subunit 89865-89899 6:03:01 GGAGCGGCTCACGGGGCGCGCGGCGGAGCACCTGG
89961 89979 89300 91804 CDS product helicase-primase helicase subunit 89961-89979 4:01:01 GGCCGGGCTGCTCGGGCGG

90515 90537 89300 91804 CDS product helicase-primase helicase subunit 90515-90537 4:01:01
CCTTCGACCGGTACCGCGAGGCC
90578 90597 89300 91804 CDS product helicase-primase helicase subunit 90578-90597 4:01:01
CCAACGCCGGCCGCATCACC
90705 90727 89300 91804 CDS product helicase-primase helicase subunit 90705-90727 4:01:01
CCAGGTGGCCGTCACCACGCGCC
90758 90781 89300 91804 CDS product helicase-primase helicase subunit 90758-90781 4:01:01
CCTTCGAGGCCTTCGCGGCCGTCC
90898 90940 89300 91804 CDS product helicase-primase helicase subunit 90898-90940 7:04:01
GGGCTGGCGCCCGAGGCCGTCACGGCGGCCTACCGGCGCCTGG
90926 90945 89300 91804 CDS product helicase-primase helicase subunit 90926-90945 5:02:01
CCTACCGGCGCCTGGCCGCC
91006 91026 89300 91804 CDS product helicase-primase helicase subunit 91006-91026 5:02:01
CCCGCCGCCCCCGCGGGCCCC
91020 91042 89300 91804 CDS product helicase-primase helicase subunit 91020-91042 5:02:01
GGGCCCCGGGGGCGCGCCGGCGG
91257 91295 89300 91804 CDS product helicase-primase helicase subunit 91257-91295 6:03:01
GGTGCGCGGGTGCGAGGTGTTTATGGGGGGGCTGCGCGG
91515 91537 89300 91804 CDS product helicase-primase helicase subunit 91515-91537 4:01:01
GGACGACCTGGAGCTGGACATGG
91907 91933 91863 92300 CDS product nuclear protein UL4 91907-91933 5:02:01 GGCGGCTGGGCGCAGTCGGGCTGTGGG
92033 92077 91863 92300 CDS product nuclear protein UL4 92033-92077 8:05:02
GGGCCGGTGCTCACGGTCCTGGCCGTGGACGGGGAGCCGGAGCGG

92097 92121 91863 92300 CDS product nuclear protein UL4 92097-92121 5:02:01 GGACCTGGCCGGCGGCCCCCGAGGG
92100 92116 91863 92300 CDS product nuclear protein UL4 92100-92116 4:01:01 CCTGGCCGGCGGCCCCC
92180 92201 91863 92300 CDS product nuclear protein UL4 92180-92201 4:01:01 GGGCCCGCGGGCGGCCTGCTGG
92235 92254 91863 92300 CDS product nuclear protein UL4 92235-92254 4:01:01 CCATCACCGCGTACCGGCCC
92496 92507 92476 93150 CDS product protein V57 92496-92507 4:01:01 GGAGGCGGGGGG
92516 92586 92476 93150 CDS product protein V57 92516-92586 14:11:03
GGCCCCGGGACGGGGTCCGGGGGCGCGGGAGGGCGGCCGCCGAGGGGACGGCGGCGCGGGCCGAGGCGTGG
92597 92629 92476 93150 CDS product protein V57 92597-92629 6:03:01 GGCGGGGTCTCGGCAGCTGGGGCGTCGGGGCGG
92660 92689 92476 93150 CDS product protein V57 92660-92689 5:02:01 GGGGCCGGGGCAGCTGGACCGCCGGGGCGG
92678 92697 92476 93150 CDS product protein V57 92678-92697 4:01:01 CCGCCGGGGCGGCCGTCGCC
92717 92734 92476 93150 CDS product protein V57 92717-92734 5:02:01 GGCGGCGCGGGGGTGCGG
92752 92769 92476 93150 CDS product protein V57 92752-92769 4:01:01 CCGCCCGCGGCCGTCGCC
92867 92920 92476 93150 CDS product protein V57 92867-92920 12:09:03
GGTGGTGGTGGTGGGTAGGCTGGACGACGGTGGCGTAGACGGGGTCCGGCTCGG
92948 92978 92476 93150 CDS product protein V57 92948-92978 5:02:01 GGGCGGTGCCCAGGCCGCCGGCGCGCAGGGG
93036 93076 92476 93150 CDS product protein V57 93036-93076 9:06:02
GGCGGAGACGCGGCGGGAGGCCGCGGCCAGGCGCCGGGCGG
93056 93078 92476 93150 CDS product protein V57 93056-93078 4:01:01 CCGCGGCCAGGCGCCGGGCGGCC
93087 93105 92476 93150 CDS product protein V57 93087-93105 4:01:01 GGCCTGCAGGAACCGGTGG
93249 93279 93147 93860 CDS product nuclear protein UL3 93249-93279 4:01:01 GGGCGCGCGCGGGGCTCGCGTGGGCGTCGGG
93363 93388 93147 93860 CDS product nuclear protein UL3 93363-93388 4:01:01 CCGCGCCCGGGGGCGCCGCGCGGGCC
93410 93467 93147 93860 CDS product nuclear protein UL3 93410-93467 11:08:02
GGACGTCTTGGTGCAGGCGACGGGGAGGCCGGCCAGGGCCGCGGCCAGGTCCCGGCGG

93438 93462 93147 93860 CDS product nuclear protein UL3 93438-93462 5:02:01 CCGGCCAGGGCCGCGGCCAGGTCCC
93475 93493 93147 93860 CDS product nuclear protein UL3 93475-93493 4:01:01 CCGTCAGCCGCCGGCGCCC
93558 93569 93147 93860 CDS product nuclear protein UL3 93558-93569 4:01:01 CCCGCCGCCGCC
93578 93664 93147 93860 CDS product nuclear protein UL3 93578-93664 16:13:04
CCCTTCGTCCCCCCCGTCTTCCTCGTCTTCCGTCTCCTCCTTCTCCGTCGCCTTCCCCTCCTCGCCGTCTTCCACGTCCCCCTCTCC
93674 93690 93147 93860 CDS product nuclear protein UL3 93674-93690 4:01:01 CCCGAGCACCCCCTCCC
93732 93748 93147 93860 CDS product nuclear protein UL3 93732-93748 4:01:01 GGGCGCGGCCCGGAGGG
93754 93774 93147 93860 CDS product nuclear protein UL3 93754-93774 6:03:01 CCTCCTCCACGGCCCACCCCC
93809 93832 93147 93860 CDS product nuclear protein UL3 93809-93832 4:01:01 GGCCGGGGCGCCGGCGAGCGCGGG
93936 93960 93916 94866 CDS product uracil-DNA glycosylase 93936-93960 5:02:01 GGGGGCCCGGCCCGTCTGGACGAGG
93985 94033 93916 94866 CDS product uracil-DNA glycosylase 93985-94033 8:05:02
GGGCAGGTCCTGAAGGGCGTGCGGGCCAGCGGCGACGGATGGCTGAAGG
94046 94069 93916 94866 CDS product uracil-DNA glycosylase 94046-94069 5:02:01 GGCGGCGCGGGTCCGGCGCGCAGG
94238 94256 93916 94866 CDS product uracil-DNA glycosylase 94238-94256 4:01:01 CCCAGGCCTCCAGGCAGCC
94242 94281 93916 94866 CDS product uracil-DNA glycosylase 94242-94281 6:03:01
GGCCTCCAGGCAGCCGTGGGCGGGCGCGGGCAGCGTCGGG
94311 94331 93916 94866 CDS product uracil-DNA glycosylase 94311-94331 5:02:01 GGCCAGGCTCGGGGGGATCGG
94355 94379 93916 94866 CDS product uracil-DNA glycosylase 94355-94379 5:02:01 CCAGCCCGTGGGCCTGGCCCGGCCC
94364 94395 93916 94866 CDS product uracil-DNA glycosylase 94364-94395 6:03:01
GGGCCTGGCCCGGCCCGTGGTACGGGTCCTGG
94406 94428 93916 94866 CDS product uracil-DNA glycosylase 94406-94428 4:01:01 CCACCTTGACGTCCTCGGGCGCC
94479 94504 93916 94866 CDS product uracil-DNA glycosylase 94479-94504 5:02:01 GGTCAGGCAGCGGCCGCGGTACTCGG
94602 94648 93916 94866 CDS product uracil-DNA glycosylase 94602-94648 9:06:02

GGGCGCCCCGGGGTCGGGGGGCGGGGCGGAGGCGTCGGCCCCCGTGG
94686 94729 93916 94866 CDS product uracil-DNA glycosylase 94686-94729 10:07:02
GGGCCGGGCGGCGGCGGAGGGTCGGGCCGGCTTCGAGGCCCCGG
94761 94802 93916 94866 CDS product uracil-DNA glycosylase 94761-94802 10:07:02
GGCGGTGGCGGCGTTAGAGGCAGAAGCGGCGGCGGCGGCAGG
94821 94838 93916 94866 CDS product uracil-DNA glycosylase 94821-94838 4:01:01 GGGCGGCAGGCCGCAGGG
94902 94930 94844 95314 CDS product envelope glycoprotein L 94902-94930 6:03:01
CCACCTCTCCGAGCTCCTTCAGGGCCGCC
94951 94994 94844 95314 CDS product envelope glycoprotein L 94951-94994 8:05:02
GGCCAGGGCCTGGATGGCGACAAAGGGGTTGACCAGGTAGGCGG
95154 95174 94844 95314 CDS product envelope glycoprotein L 95154-95174 4:01:01 CCCATTCCAGCTCCTCGCGCC
95175 95211 94844 95314 CDS product envelope glycoprotein L 95175-95211 7:04:01
GGGGAGCGCGGGTGGGGGGCGCGGGCGTGACCGGCGG
95243 95278 94844 95314 CDS product envelope glycoprotein L 95243-95278 7:04:01
CCCGCGACGCCGGTGCCAGGAACCCCCAGCGCCGCC
96146 96219 96112 109118 misc_RNA product large latency transcript 96146-96219 14:11:03
CCATCTCGCCAGCCAGCCAACCAGCCGAGCCGCCCAGCCGACCCGAGAGCCCCGAGAGCCAGACTCCCTCAGCC
97208 97231 96481 97584 CDS product ubiquitin E3 ligase ICP0 97208-97231 4:01:01 CCGTCGAGACCTGCCCATAAAGCC
97266 97296 96481 97584 CDS product ubiquitin E3 ligase ICP0 97266-97296 6:03:01
CCCAGACCCGGAGCCCTGCCCTTCGGCCTCC
97327 97373 96481 97584 CDS product ubiquitin E3 ligase ICP0 97327-97373 8:05:02
CCCCAGATGACCGCGAAGCCCCCCCCTACCGGCTCATCCTCTTCCCC
97382 97395 96481 97584 CDS product ubiquitin E3 ligase ICP0 97382-97395 4:01:01 CCGTCGCCCCCTCC

97433 97456 96481 97584 CDS product ubiquitin E3 ligase ICP0 97433-97456 6:03:01 GGTGGAGGATGGAGGTGACGCGGG

103175 103207 103171 107511 CDS product transcriptional regulator ICP4 103175-103207 5:02:01 GGAGCAGCAGGTAGGGGTTGCCGGCGTCCTCGG

103195 103236 103171 107511 CDS product transcriptional regulator ICP4 103195-103236 8:05:02 CCGGCGTCCTCGGCCTCCTCGTCGTCCGAGATGGCCTCCACC

103242 103266 103171 107511 CDS product transcriptional regulator ICP4 103242-103266 4:01:01 GGGCCCGAGCGGGCCGCGGGGCCGG

103245 103286 103171 107511 CDS product transcriptional regulator ICP4 103245-103286 7:04:01 CCCGAGCGGGCCGCGGGGCCGGCCGTCGCCGCCGCGGACGCC

103295 103351 103171 107511 CDS product transcriptional regulator ICP4 103295-103351 10:07:02 CCACAGAGTCCCCGTCCTCGCCGGGGCCGGCCCCGGCGCCCGAGGCCCCCGCGGGCC

103317 103405 103171 107511 CDS product transcriptional regulator ICP4 103317-103405 15:12:03 GGGGCCGGCCCCGGCGCCCGAGGCCCCCGCGGGCCGGTGGGTCTCCACGGCGCCCCCGGCGGCGGCGCGGACGCTGGTCTCGAAGGGGG

103422 103459 103171 107511 CDS product transcriptional regulator ICP4 103422-103459 7:04:01 GGCCGGCGGGGCGCCCGCGGCGGCGACGGCGCCCGGGG

103471 103490 103171 107511 CDS product transcriptional regulator ICP4 103471-103490 4:01:01 GGGGCGGCCTCGGCGTCGGG

103527 103579 103171 107511 CDS product transcriptional regulator ICP4 103527-103579 10:07:02 GGCCGGCAGGCCCTCGGGGCCGCGGAGCTCGGCGAGGCCCCGGCGGCCGCAGG

103697 103716 103171 107511 CDS product transcriptional regulator ICP4 103697-103716 4:01:01 CCATGTCCTTGCAGCCGTCC

103736 103786 103171 107511 CDS product transcriptional regulator ICP4 103736-103786 10:07:02 GGCGGTAGGCGCGCGGCGGCAGCGGGACCGGGGTCCGGGGCCCGGCGCGGG

103814 103864 103171 107511 CDS product transcriptional regulator ICP4 103814-103864 10:07:02
GGCGGCAGAGGCGCAGCGGCTCGGCCCCGGGGTGCAGGCGGGCGAAGGAGG
103917 103943 103171 107511 CDS product transcriptional regulator ICP4 103917-103943 4:01:01
GGCCGCGCGGAGCTCGCGGCACCCGGG
103953 103986 103171 107511 CDS product transcriptional regulator ICP4 103953-103986 7:04:01
GGCGCACTGGGCGGCCGGGTCCAGGCGGACGCGG
104003 104034 103171 107511 CDS product transcriptional regulator ICP4 104003-104034 7:04:01
CCCCCACGGCCGGGCCGTCCGCGGGCCAGTCC
104058 104080 103171 107511 CDS product transcriptional regulator ICP4 104058-104080 5:02:01
CCGGCGCCGCGCCGCGCCGAGCC
104096 104127 103171 107511 CDS product transcriptional regulator ICP4 104096-104127 5:02:01
CCACGGCGCCGGCGAAGCCGAGGTCCCGCGCC
104175 104194 103171 107511 CDS product transcriptional regulator ICP4 104175-104194 4:01:01
CCCGGTCCAGTTCCCGGCCC
104219 104236 103171 107511 CDS product transcriptional regulator ICP4 104219-104236 5:02:01
GGCGGTGGGCGAAGGCGG
104252 104305 103171 107511 CDS product transcriptional regulator ICP4 104252-104305 9:06:02
GGCCGCCGCGGCGCGGGTCCCAGGCCGGGCGCGGGGCGCCCTCGGCGGGCTCGG
104385 104422 103171 107511 CDS product transcriptional regulator ICP4 104385-104422 8:05:02
GGCGGCGCGGCGGCGGAGCGGGGCGCCGCGGCGCGCGG
104435 104452 103171 107511 CDS product transcriptional regulator ICP4 104435-104452 4:01:01
GGGCGGCCGGGTCGAAGG
104489 104586 103171 107511 CDS product transcriptional regulator ICP4 104489-104586 16:13:04

GGTCCACGAGGGCGCGGGCCACCTCGGGCGGGCAGTAGGCGGCGAGGGCCGCCTCGGAGGGCCGCGGCGTGTGGGTCTCGCCGGCCGGGACGCGGCGG

104598 104643 103171 107511 CDS product transcriptional regulator ICP4 104598-104643 7:04:01 GGGCGCGGGGTGCTCGGGCATGGGCCCGAGCGGGCGCCGGAGCCGG

104652 104671 103171 107511 CDS product transcriptional regulator ICP4 104652-104671 7:04:01 GGAGGAGGAGGAGGAGGAGG

104682 104702 103171 107511 CDS product transcriptional regulator ICP4 104682-104702 4:01:01 GGGAGCGGGGTCCGGAGCGGG

104730 104745 103171 107511 CDS product transcriptional regulator ICP4 104730-104745 4:01:01 CCGGGGCCCCCGGTCC

104786 104822 103171 107511 CDS product transcriptional regulator ICP4 104786-104822 7:04:01 CCGCGCCGGAGAGCCCCTCGTCCTCCTCGCCGTCCCC

104823 104847 103171 107511 CDS product transcriptional regulator ICP4 104823-104847 5:02:01 GGGGCGGCGGGCCCCGGGCGCGCGG

104868 105016 103171 107511 CDS product transcriptional regulator ICP4 104868-105016 30:27:07 GGGCGCTGGGTCCGGGCCGGCGGCGGGGGAGCTGGCGTAGCCGGAGGAGCCGGAGAGGCCGGACTTGGTGCTGGAGCTGGACTTGGTGCTGGAGCTGGACTTGGTGCTGGCGGGGCTGGAGGGCCCGGAGCCGGGGAGGCCGGAGGGGG

105019 105036 103171 107511 CDS product transcriptional regulator ICP4 105019-105036 5:02:01 CCCGCCGCCGCCGGCGCC

105031 105202 103171 107511 CDS product transcriptional regulator ICP4 105031-105202 27:24:06 GGCGCCGGCGCTGGGACGACGAGGCCGGGCTGCTCGGGCCAGAGCGGGGGCAGGCCGGGCGCGGGCTCCGCGGGCCCGGGCCGCGCGGCGGCCTCGGCGAGCCGGGCCGCGGCCACGTTGGCCGGGGCGAAGAGGGCCGCGGCGTAGGTCCAGGCGGCCTCGCGGGCGCGGG

105132 105153 103171 107511 CDS product transcriptional regulator ICP4 105132-105153 4:01:01 CCGGGCCGCGGCCACGTTGGCC

105229 105253 103171 107511 CDS product transcriptional regulator ICP4 105229-105253 4:01:01

GGCGCCACGGTGCGGGCGACGAGGG

105263 105296 103171 107511 CDS product transcriptional regulator ICP4 105263-105296 7:04:01

CCGCGGCCTGCCGCCGCTCGGCCGGGCCGGCCCC

105282 105300 103171 107511 CDS product transcriptional regulator ICP4 105282-105300 4:01:01

GGCCGGGCCGGCCCCGGGG

105344 105445 103171 107511 CDS product transcriptional regulator ICP4 105344-105445 18:15:04

GGCAGGCGGGCCCGAGGGCGGCCGGGGCGCGGGCGGGCGCGGGCAGCCGGAGCGGGCAGGGCAGCAGGCGCTCGAGGACGCCGCGGCAGGCCAGGACGCAGG

105461 105526 103171 107511 CDS product transcriptional regulator ICP4 105461-105526 11:08:02

GGGGCACGCGGCCGGGCTGCGCGGCGGCGAAGGCGGCGCGGACGCGGGCGCAGAGGGCCTCGACGG

105537 105619 103171 107511 CDS product transcriptional regulator ICP4 105537-105619 15:12:03

GGCGCGGGGGTCCGCGGCGCGGCCCGGGTAGGCCATGTCGGCGTAGGCCCGGCGGAGGCTCTGCAGGATGAAGGTCTTCTGGG

105632 105649 103171 107511 CDS product transcriptional regulator ICP4 105632-105649 4:01:01

GGCGGCTCATGGCCACGG

105679 105697 103171 107511 CDS product transcriptional regulator ICP4 105679-105697 4:01:01

GGGTCCTGGGCGGCCATGG

105735 105789 103171 107511 CDS product transcriptional regulator ICP4 105735-105789 8:05:02

GGTGATGAAGGAGCCGTGGCCGTGGGGCGCGTGGACCCGGCGCTGGCAGAACTGG

105834 105853 103171 107511 CDS product transcriptional regulator ICP4 105834-105853 4:01:01

GGACATGGCCTCGCCGGCGG

105894 105912 103171 107511 CDS product transcriptional regulator ICP4 105894-105912 4:01:01

CCTCGAGTCCCCCATCTCC

105924 105969 103171 107511 CDS product transcriptional regulator ICP4 105924-105969 9:06:02

GGGCACGGGCCCGGCCGCGGCGCGGTAGCGGGCCGCGGCCTGGCGG

106022 106061 103171 107511 CDS product transcriptional regulator ICP4 106022-106061 8:05:02
GGACGCGGCCGTCGGCCGGAGGGTCGGAGCCGGGCCAGGG
106084 106111 103171 107511 CDS product transcriptional regulator ICP4 106084-106111 6:03:01
GGCCCGTCGGTCGGCGGGGGCCCGTCGG
106124 106143 103171 107511 CDS product transcriptional regulator ICP4 106124-106143 5:02:01
GGTGGTTGTTGGTGGAGCGG
106156 106296 103171 107511 CDS product transcriptional regulator ICP4 106156-106296 30:27:07
GGGGGCCGGGCGGGCTCCGGGGCCGGGGCCGGGGAGGCCGCGGCGGAGGAGGAGGCGGAGGAGGCCGAGGGCCGCGGGGCCGCGGCGGGCGCCGGCGGAGACGGTGGCGGCCCGGCGCGGGCGAGTGG
GGCGCCGGGCCGG
106172 106194 103171 107511 CDS product transcriptional regulator ICP4 106172-106194 4:01:01
CCGGGGCCGGGGCCGGGGAGGCC
106320 106378 103171 107511 CDS product transcriptional regulator ICP4 106320-106378 14:11:03
GGAGGAGGACGAGGACGAGGAGGACGAGGAGGACGAGGAGGACGAGGACGAGGAGGAGG
106392 106440 103171 107511 CDS product transcriptional regulator ICP4 106392-106440 12:09:03
GGCGGCGGCGGGGGCCCGGGGGGCGGAGGGCGAGCGGGCCGGGGAGAGG
106481 106495 103171 107511 CDS product transcriptional regulator ICP4 106481-106495 4:01:01 GGCCGCGGCGGAGGG
106506 106523 103171 107511 CDS product transcriptional regulator ICP4 106506-106523 4:01:01
GGCGCGGCGCCGGCGGGG
106541 106578 103171 107511 CDS product transcriptional regulator ICP4 106541-106578 7:04:01
CCCCGCCGTCCCCGGCGAGGCCGAGTCCGTCCTCGTCC
106584 106666 103171 107511 CDS product transcriptional regulator ICP4 106584-106666 15:12:03
GGGGCCGCGGGCGACGGTCTCGACGGCGACGGTGGTGGTGGAGTTGGAGTTGGAGTTGGGGTTGGAGGAGACGGGGCTCCGGG
106718 106740 103171 107511 CDS product transcriptional regulator ICP4 106718-106740 4:01:01

GGGGCTCGCGGTGCTGGTGATGG

106761 106771 103171 107511 CDS product transcriptional regulator ICP4 106761-106771 4:01:01 GGCGGAGGGGG

106774 106802 103171 107511 CDS product transcriptional regulator ICP4 106774-106802 7:04:01 CCGCCGCCGCCGGGCGCCGAGACCGGCCC

106798 106829 103171 107511 CDS product transcriptional regulator ICP4 106798-106829 8:05:02 GGCCCGGCGGCGGGGGAGGCTGGGGAAGCGGG

106830 106842 103171 107511 CDS product transcriptional regulator ICP4 106830-106842 4:01:01 CCCCCGCCGTGCC

106864 106892 103171 107511 CDS product transcriptional regulator ICP4 106864-106892 5:02:01 GGCTGTGCTGGTGGCGCCGGGGTCCGAGG

106880 106930 103171 107511 CDS product transcriptional regulator ICP4 106880-106930 10:07:02 CCGGGGTCCGAGGCCGCGCCGCCGGCCCGGGCTCACCGACCGGGTCCCCCC

106951 107008 103171 107511 CDS product transcriptional regulator ICP4 106951-107008 12:09:03 GGGGCTGCCGAGGGGCCGGGAGAGCCGGAGGAGGAGGCCGGGGAGGCTGCGGAGGGGG

107016 107046 103171 107511 CDS product transcriptional regulator ICP4 107016-107046 6:03:01 CCCGGGGCCGCCGGGGGCCCCGGCCTCTGCC

107019 107038 103171 107511 CDS product transcriptional regulator ICP4 107019-107038 4:01:01 GGGGCCGCCGGGGGCCCCGG

107062 107115 103171 107511 CDS product transcriptional regulator ICP4 107062-107115 10:07:02 GGGGTCGGCGGCCGGGGCCCGGAGCCGGCCCGGGACCGGGGCCCGAGGACGAGG

107073 107105 103171 107511 CDS product transcriptional regulator ICP4 107073-107105 6:03:01 CCGGGGCCCGGAGCCGGCCCGGGACCGGGGCCC

107144 107174 103171 107511 CDS product transcriptional regulator ICP4 107144-107174 5:02:01 CCCGACCGGGGGACCCGGCGGCCGGGGACCC

107151 107177 103171 107511 CDS product transcriptional regulator ICP4 107151-107177 6:03:01
GGGGGACCCGGCGGCCGGGGACCCGGG
107252 107358 103171 107511 CDS product transcriptional regulator ICP4 107252-107358 18:15:04
GGGCCGAAGGAGGACGGGGCGGCCTCGTGGCTCCGGCCGCGGCCGCGAGGACGGCGGCCTCGGCCTCGGCGGCGTCGTCGGAGAAGAGGCCGCCCGGGCCGAAGAGG
107363 107414 103171 107511 CDS product transcriptional regulator ICP4 107363-107414 8:05:02
CCTCGCCGGAGGAGCCGCGGCGCCGGGAGCCCTGGCTGCCGCCGTCGGGGCC
107370 107397 103171 107511 CDS product transcriptional regulator ICP4 107370-107397 5:02:01
GGAGGAGCCGCGGCGCCGGGAGCCCTGG
107436 107463 103171 107511 CDS product transcriptional regulator ICP4 107436-107463 6:03:01
GGCCGCGGCGGCGGCCGCCAGGAGCTGG
107938 107957 96112 109118 misc_RNA product large latency transcript 107938-107957 4:01:01
GGCGGCCGCCGGCCAATGGG
107996 108024 96112 109118 misc_RNA product large latency transcript 107996-108024 5:02:01
CCCGGCCCCCGCGGCGGCCATCTTGCCCC
108081 108117 96112 109118 misc_RNA product large latency transcript 108081-108117 5:02:01
CCGGCCCCGAGGGCGCCATCTTGGCCCCTCGACGGCC
108163 108180 96112 109118 misc_RNA product large latency transcript 108163-108180 4:01:01
CCGGCCCCCGCGGCGGCC
108165 108215 96112 109118 misc_RNA product large latency transcript 108165-108215 8:05:02
GGCCCCCGCGGCGGCCATCTCGGCTCGCCCGGGCCAATGGGCGCGCGGAGG
108414 108444 96112 109118 misc_RNA product large latency transcript 108414-108444 5:02:01
CCCCACGTGGCCGCCCTCGGCCAATGGGGCC
108692 108706 96112 109118 misc_RNA product large latency transcript 108692-108706 5:02:01

CCGCCTCCCCCTGCC
108717 108801 96112 109118 misc_RNA product large latency transcript 108717-108801 16:13:04
CCCCGAGACCCCCGTTTCCCCCTCCTCGAGACCCCTGAGACCCCCGAGACCCTCCCGCGACCCCCGCGGTCGCCCCACCCGCGCC
108857 108894 96112 109118 misc_RNA product large latency transcript 108857-108894 7:04:01
CCGCCGTCGGACCGGGGACCGGCGACCGGACCCGAACC
108865 108885 96112 109118 misc_RNA product large latency transcript 108865-108885 4:01:01
GGACCGGGGACCGGCGACCGG
108920 108956 96112 109118 misc_RNA product large latency transcript 108920-108956 7:04:01
CCGGACCCGAACCTCGAGCCCGGACCCGCCCGGACCC
108951 108972 96112 109118 misc_RNA product large latency transcript 108951-108972 5:02:01
GGACCCGGAAGGAAGGAGCCGG
109000 109028 96112 109118 misc_RNA product large latency transcript 109000-109028 8:05:02
CCCACCCACCCCCTCCTCTCCCCCACCCC
109042 109062 96112 109118 misc_RNA product large latency transcript 109042-109062 5:02:01
CCCGGTCCCCCCTCCCACCCC
115996 116017 115995 117089 CDS product regulatory protein ICP22 115996-116017 4:01:01
GGACCGGGTCTGGGCCGACTGG
116024 116088 115995 117089 CDS product regulatory protein ICP22 116024-116088 11:08:02
CCCGTGCCCTCCCCGCCGTTCTCGCCCGTCGACCCGCCCGGGCCCCGGCCCACGACCCCGGTCCC
116098 116131 115995 117089 CDS product regulatory protein ICP22 116098-116131 9:06:02
CCCCCCGTCCCCCGCCTCGACCCCCACGCCCCCC
116171 116185 115995 117089 CDS product regulatory protein ICP22 116171-116185 4:01:01 CCCCCGCCCGACCCC
116380 116398 115995 117089 CDS product regulatory protein ICP22 116380-116398 4:01:01 CCAGCGCCCCTCGGCCCCC

116488 116508 115995 117089 CDS product regulatory protein ICP22 116488-116508 4:01:01

CCCGCGCGTCCTCTCCCCGCC

116603 116687 115995 117089 CDS product regulatory protein ICP22 116603-116687 15:12:03

GGCTCGGTGTGCGAGGACGACGGGGAGGACGAGGACGAAGAGGAAGACGGGGAGGAGGAAGACGAGGACGAGGAGGGGGAAGAGG

116698 116732 115995 117089 CDS product regulatory protein ICP22 116698-116732 7:04:01

GGAGGAAGAGGAAGGGGACGAGGACGGGGAGACGG

116743 116786 115995 117089 CDS product regulatory protein ICP22 116743-116786 9:06:02

GGAGGACGACGAGGCCGAGGACGAGGAGGACGAGGAGGACGGGG

116839 116888 115995 117089 CDS product regulatory protein ICP22 116839-116888 10:07:02

GGACGGCTCGGACGGAGAGGGCTCGGGCTCGGACGACGGCGGGGACGGGG

116920 116988 115995 117089 CDS product regulatory protein ICP22 116920-116988 13:10:03

GGACGAGGACGATGGAGAGGACGAGGAGGACGAGGAAGGGGAGGACGGGGGGGAAGACGGCGAAGACGG

117011 117035 115995 117089 CDS product regulatory protein ICP22 117011-117035 6:03:01

GGAGAGGGCGAGGAGGGCGGGAAGG

117060 117084 115995 117089 CDS product regulatory protein ICP22 117060-117084 5:02:01

CCCCGACGCGGCCCGCCGCCGCCCC

118439 118463 118332 119336 CDS product serine/threonine protein kinase US3 118439-118463 5:02:01

GGGGGGCTGACGCGGCAGGCCGCGG

118537 118581 118332 119336 CDS product serine/threonine protein kinase US3 118537-118581 9:06:02

GGCCCGCCGGCCGGGCGAGGCGGACACGGTGGTGCTGAAGGTGGG

118935 118960 118332 119336 CDS product serine/threonine protein kinase US3 118935-118960 5:02:01

CCGGGACCATCGAGACCAACGCCCCC

119004 119018 118332 119336 CDS product serine/threonine protein kinase US3 119004-119018 4:01:01

GGGGCGCGGGGGTGG
119092 119114 118332 119336 CDS product serine/threonine protein kinase US3 119092-119114 4:01:01 CCTGATCGACCTCATCCGCGCCC
119170 119196 118332 119336 CDS product serine/threonine protein kinase US3 119170-119196 6:03:01 CCGGTACGCCGGGACCCACCGCCAGCC
119279 119296 118332 119336 CDS product serine/threonine protein kinase US3 119279-119296 5:02:01 CCCGTCCGCCGCCCTTCC
119415 119465 119396 120892 CDS product envelope glycoprotein G 119415-119465 7:04:01 CCTCGCCCTCGGGCTCCTCGTGGTCCGCACCGTCGTGGCCAGAGAGGCCCC
119484 119568 119396 120892 CDS product envelope glycoprotein G 119484-119568 15:12:03 CCACCCCGTCCACGACGACCGGCGGCCCGTCGGGCCCGCGACCGACGCCCAGCCCGTGAACCCGCTCGCCCCCGCCAACGCCACC
119601 119615 119396 120892 CDS product envelope glycoprotein G 119601-119615 4:01:01 CCTCCTGGATCCGCC
119724 119745 119396 120892 CDS product envelope glycoprotein G 119724-119745 4:01:01 CCCCGGGGACGCCATGCCCTCC
119894 119914 119396 120892 CDS product envelope glycoprotein G 119894-119914 4:01:01 CCTACCTCGGCACCGTCTCCC
119919 119935 119396 120892 CDS product envelope glycoprotein G 119919-119935 4:01:01 GGTGGAGGCCAACCTGG
119972 120022 119396 120892 CDS product envelope glycoprotein G 119972-120022 11:08:02 CCCGCCCCGGGGCCACCCTCCCACCCATCGCCCCCACGGCCGGCGACCACC
120173 120186 119396 120892 CDS product envelope glycoprotein G 120173-120186 4:01:01 CCCCCGAGGCCCCC
120180 120214 119396 120892 CDS product envelope glycoprotein G 120180-120214 7:04:01 GGCCCCCGAGGGCGAGGAGGTGACCGAGGAGGAGG
120215 120235 119396 120892 CDS product envelope glycoprotein G 120215-120235 4:01:01 CCGAGCTGACCTCCAGCGACC
120400 120436 119396 120892 CDS product envelope glycoprotein G 120400-120436 10:07:02 CCGCCTCCACCACCCCCGCGCCCCCACCCGCGCGGCC

120447 120466 119396 120892 CDS product envelope glycoprotein G 120447-120466 4:01:01 CCATGACCACGGTCACCACC
120498 120519 119396 120892 CDS product envelope glycoprotein G 120498-120519 5:02:01 CCGACTGCCGCCGGAGCCGACC
120548 120583 119396 120892 CDS product envelope glycoprotein G 120548-120583 7:04:01
CCCCCACCGGCAGCCCCGCCCTGCTCCTGGGCTTCC
120599 120624 119396 120892 CDS product envelope glycoprotein G 120599-120624 5:02:01
CCTCGCGCCCCCTGCACCTGACGGCC
120666 120694 119396 120892 CDS product envelope glycoprotein G 120666-120694 6:03:01
CCGCCACATCCGCTCCCTCGGCGGCCTCC
120710 120745 119396 120892 CDS product envelope glycoprotein G 120710-120745 8:05:02
CCGAGACCACCAACACCACCACCACCCAGACGGGCC
120845 120866 119396 120892 CDS product envelope glycoprotein G 120845-120866 4:01:01 GGGCCCGGGCCCGGAACGACGG
121094 121112 121075 122277 CDS product envelope glycoprotein D 121094-121112 4:01:01 GGCGGCGCTGGTCGCCCGG
121138 121181 121075 122277 CDS product envelope glycoprotein D 121138-121181 9:06:02
CCGTGCCCGCGCCGACCTTCCCCCCGCCCGCGTACCCGTACACC
121322 121340 121075 122277 CDS product envelope glycoprotein D 121322-121340 5:02:01 GGCGGTGGCCCACCGGCGG
121330 121371 121075 122277 CDS product envelope glycoprotein D 121330-121371 7:04:01
CCCACCGGCGGCCCACGTACCGCGCCCACGTGGCCTGGTACC
121451 121468 121075 122277 CDS product envelope glycoprotein D 121451-121468 4:01:01 CCGGCGCCGCACCACGCC
121532 121547 121075 122277 CDS product envelope glycoprotein D 121532-121547 4:01:01 GGTGGCTCCGGGGCGG
121559 121580 121075 122277 CDS product envelope glycoprotein D 121559-121580 4:01:01 CCAGTACCGGCGCCTGGTGTCC
121640 121665 121075 122277 CDS product envelope glycoprotein D 121640-121665 4:01:01
CCCGTTCGCCCGCGTGGACCAGCACC
121733 121764 121075 122277 CDS product envelope glycoprotein D 121733-121764 6:03:01

CCTGACGCCGTTCTACCAGCAGCCCCCGCACC

121799 121859 121075 122277 CDS product envelope glycoprotein D 121799-121859 10:07:02
CCGGACGCTCCCGCGGGCCTACGCCGCCGCCACGCCGTACGCCATCGACCCCGCGCGGCCC

121872 121957 121075 122277 CDS product envelope glycoprotein D 121872-121957 16:13:04
CCGAGGCCCAGGCCCCGGCCCCGGCCCCGGCCGAAGCCCGAGCCCGCCCCGGTGACGCCCGCGCCCCCCGGCCGCCTGCCCGAGCC

121876 121901 121075 122277 CDS product envelope glycoprotein D 121876-121901 5:02:01
GGCCCAGGCCCCGGCCCCGGCCCCGG

121970 122054 121075 122277 CDS product envelope glycoprotein D 121970-122054 16:13:04
CCACGCCGCCGGGGGCCACCCCACGCCGCGACCCCCGAGGCCCGAGACGCCGCACCGCCCCTTCGCCCCGCCGGCCGTCGTGCCC

122064 122107 121075 122277 CDS product envelope glycoprotein D 122064-122107 9:06:02
CCGCAGCCCGCGGAGCCGTTCCAGCCGCGGACCCCCGCCGCGCC

122341 122376 122298 123398 CDS product envelope glycoprotein I 122341-122376 8:05:02
CCTCCTCGCCGCCCTGACCCTGGCCGCCCTGACCCC

122427 122449 122298 123398 CDS product envelope glycoprotein I 122427-122449 4:01:01 CCGGCAGCGCCGTCCTCGTGCCC

122593 122624 122298 123398 CDS product envelope glycoprotein I 122593-122624 6:03:01
CCTCTGCCCCCGCGTGCACCACGAGGCCTTCC

122674 122689 122298 123398 CDS product envelope glycoprotein I 122674-122689 4:01:01 GGTGGAGGCGCGCCGG

122806 122830 122298 123398 CDS product envelope glycoprotein I 122806-122830 6:03:01
CCCCCACCACCCCACGCCGTCGTCC

122887 122992 122298 123398 CDS product envelope glycoprotein I 122887-122992 20:17:05
CCCCGCCGAGGACGCCGTGTTCACCACGCCGCCCCCGATCGAGCCAGAGCCGCCGACGACCCCCGCGCCCCCCCGGGGGACCGGCGCCACCCCCGAGCCCCGCTCC

123001 123029 122298 123398 CDS product envelope glycoprotein I 123001-123029 7:04:01
GGAGGAGGACGAGGAGGGGGCGACGACGG

123103 123124 122298 123398 CDS product envelope glycoprotein I 123103-123124 5:02:01 CCTGCTCGCCGCCGCCAACGCC

123127 123148 122298 123398 CDS product envelope glycoprotein I 123127-123148 4:01:01 GGCGGGCGCCCGGGGCCCCGGG

123559 123629 123502 125235 CDS product envelope glycoprotein E 123559-123629 12:09:03
CCACCGAGGCCCCGAGCCTCTCCGCCGAGACGACCCCGGGCCCCGTCACCGAGGTCCCGAGTCCCTCGGCC

123641 123656 123502 125235 CDS product envelope glycoprotein E 123641-123656 4:01:01 CCTCTCCACCGAGGCC

123733 123756 123502 125235 CDS product envelope glycoprotein E 123733-123756 4:01:01 GGGAGGCACCCCCGGCCCATCTGG

123798 123827 123502 125235 CDS product envelope glycoprotein E 123798-123827 6:03:01
GGCGACGGCGCCGTGGTGGCCGGGATCTGG

123886 123903 123502 125235 CDS product envelope glycoprotein E 123886-123903 5:02:01 CCGCCTGCCACCCGGACC

123914 123937 123502 125235 CDS product envelope glycoprotein E 123914-123937 4:01:01 CCGCGCCTGCGTCCCCGAGGCCCC

123932 123952 123502 125235 CDS product envelope glycoprotein E 123932-123952 4:01:01 GGCCCCGGAGCGGGGCATCGG

123959 123984 123502 125235 CDS product envelope glycoprotein E 123959-123984 5:02:01
CCTGCCGCCCGAGGTGCCGCGGCTCC

124023 124052 123502 125235 CDS product envelope glycoprotein E 124023-124052 6:03:01
CCGCACCTGACCGTCCGGCGGGCCACGCCC

124112 124127 123502 125235 CDS product envelope glycoprotein E 124112-124127 4:01:01 GGCGGTGGGCGACCGG

124124 124179 123502 125235 CDS product envelope glycoprotein E 124124-124179 9:06:02
CCGGCCGCCCGCGCCGCTGGCCCCGGTGGGCCCCGCGCGCCACGAGCCCCGCTTCC

124355 124393 123502 125235 CDS product envelope glycoprotein E 124355-124393 6:03:01
CCACCCGCGCGCGCCCGAGTGCCTGCGCCCGGTGGACCC

124486 124505 123502 125235 CDS product envelope glycoprotein E 124486-124505 4:01:01 CCGCCTGCCCCTTCGACGCC

124570 124592 123502 125235 CDS product envelope glycoprotein E 124570-124592 4:01:01 CCCACAACGGCCACGTCGCCACC

124634 124672 123502 125235 CDS product envelope glycoprotein E 124634-124672 6:03:01

GGTCATCAAGGAGCTGACGGCCCCGGCCCGGGCCCCGGG

124654 124688 123502 125235 CDS product envelope glycoprotein E 124654-124688 5:02:01
CCCCGGCCCGGGCCCCGGGCACCCCGTGGGGCCCC

124681 124696 123502 125235 CDS product envelope glycoprotein E 124681-124696 4:01:01 GGGGCCCCGGCGGCGG

124731 124767 123502 125235 CDS product envelope glycoprotein E 124731-124767 6:03:01
CCGGCGCCGCCCGCGCGCCCGTGGAACCCGTACGGCC

124861 124897 123502 125235 CDS product envelope glycoprotein E 124861-124897 7:04:01
CCCGCCGCCGGGCGGCCTCGCGGCCGTTCCGGGTGCC

124870 124893 123502 125235 CDS product envelope glycoprotein E 124870-124893 4:01:01 GGGCGGCCTCGCGGCCGTTCCGGG

124999 125021 123502 125235 CDS product envelope glycoprotein E 124999-125021 6:03:01 CCCGCCGGCGGCCCTCCTCCCCC

125022 125045 123502 125235 CDS product envelope glycoprotein E 125022-125045 4:01:01 GGCGGGGACAGCGGCTACGAGGGG

125200 125211 123502 125235 CDS product envelope glycoprotein E 125200-125211 4:01:01 CCGCCAGCCGCC

125306 125323 125293 125589 CDS product membrane protein US9 125306-125323 4:01:01 CCCCAGCGCCCCCGTCCC

125339 125394 125293 125589 CDS product membrane protein US9 125339-125394 10:07:02
CCCGGCCGCCGACGTCCTGCTGGCCCCCAAGGGACCCCGCTCCCCGCTGCGCCCCC

125462 125486 125293 125589 CDS product membrane protein US9 125462-125486 5:02:01
GGGACGCCGGCAGGCGGCGCGCCGG

125483 125502 125293 125589 CDS product membrane protein US9 125483-125502 4:01:01 CCGGAGACGCCGCCGCTGCC

125875 125903 125811 126581 CDS product virion protein US2 125875-125903 5:02:01
GGGCGAGGCGCACCCGGACCTGTGGAAGG

126018 126043 125811 126581 CDS product virion protein US2 126018-126043 4:01:01 CCCTGCTGCCGCGCGCCACGCGCCCC

126137 126168 125811 126581 CDS product virion protein US2 126137-126168 6:03:01
CCCGGGGAGCCCCTCCGTATCAGCCCGCCCCC

126171 126187 125811 126581 CDS product virion protein US2 126171-126187 4:01:01 GGCTGGACACGGAGTGG
126223 126239 125811 126581 CDS product virion protein US2 126223-126239 4:01:01 GGGGGCCCGGGGCACGG
126240 126336 125811 126581 CDS product virion protein US2 126240-126336 18:15:04
CCCCGATCCACCTGTGGATCCTGGGCGCCGCCGACCTCTGCGACCAGGTGCTCCTGGCCGCCTCCCGCAGCACCGCCGCCGGAGCCCCCGGCGCCCC
126357 126377 125811 126581 CDS product virion protein US2 126357-126377 5:02:01 GGCGGCGGCCCGGGCTGACGG
126405 126427 125811 126581 CDS product virion protein US2 126405-126427 5:02:01 CCGGGATCCCGGCCACCCGCGCC
126442 126466 125811 126581 CDS product virion protein US2 126442-126466 5:02:01 CCACAACCGCTCCTGGCGCCACGCC
126467 126482 125811 126581 CDS product virion protein US2 126467-126482 4:01:01 GGCGAGTGGACGGAGG
126522 126546 125811 126581 CDS product virion protein US2 126522-126546 5:02:01 GGCGCCGGGGCGGGCGCGGCAACGG
127516 127540 127513 128607 CDS product regulatory protein ICP22 127516-127540 5:02:01
GGGGCGGCGGCGGGCCGCGTCGGGG
127565 127589 127513 128607 CDS product regulatory protein ICP22 127565-127589 6:03:01
CCTTCCCGCCCTCCTCGCCCTCTCC
127612 127680 127513 128607 CDS product regulatory protein ICP22 127612-127680 13:10:03
CCGTCTTCGCCGTCTTCCCCCCCGTCCTCCCCTTCCTCGTCCTCCTCGTCCTCTCCATCGTCCTCGTCC
127712 127761 127513 128607 CDS product regulatory protein ICP22 127712-127761 10:07:02
CCCCGTCCCCGCCGTCGTCCGAGCCCGAGCCCTCTCCGTCCGAGCCGTCC
127814 127857 127513 128607 CDS product regulatory protein ICP22 127814-127857 9:06:02
CCCCGTCCTCCTCGTCCTCCTCGTCCTCGGCCTCGTCGTCCTCC
127868 127902 127513 128607 CDS product regulatory protein ICP22 127868-127902 7:04:01
CCGTCTCCCCGTCCTCGTCCCCTTCCTCTTCCTCC
127913 127997 127513 128607 CDS product regulatory protein ICP22 127913-127997 15:12:03
CCTCTTCCCCCTCCTCGTCCTCGTCTTCCTCCTCCCCGTCTTCCTCTTCGTCCTCGTCCTCCCCGTCGTCCTCGCACACCGAGCC

128092 128112 127513 128607 CDS product regulatory protein ICP22 128092-128112 4:01:01
GGCGGGGAGAGGACGCGCGGG
128202 128220 127513 128607 CDS product regulatory protein ICP22 128202-128220 4:01:01 GGGGGCCGAGGGGCGCTGG
128415 128429 127513 128607 CDS product regulatory protein ICP22 128415-128429 4:01:01 GGGGTCGGGCGGGGG
128469 128502 127513 128607 CDS product regulatory protein ICP22 128469-128502 9:06:02
GGGGGGCGTGGGGGTCGAGGCGGGGGACGGGGGG
128512 128576 127513 128607 CDS product regulatory protein ICP22 128512-128576 11:08:02
GGGACCGGGGTCGTGGGCCGGGGCCCGGGCGGGTCGACGGGCGAGAACGGCGGGGAGGGCACGGG
128583 128604 127513 128607 CDS product regulatory protein ICP22 128583-128604 4:01:01
CCAGTCGGCCCAGACCCGGTCC
137137 137164 137091 141431 CDS product transcriptional regulator ICP4 137137-137164 6:03:01
CCAGCTCCTGGCGGCCGCCGCCGCGGCC
137186 137237 137091 141431 CDS product transcriptional regulator ICP4 137186-137237 8:05:02
GGCCCCGACGGCGGCAGCCAGGGCTCCCGGCGCCGCGGCTCCTCCGGCGAGG
137203 137230 137091 141431 CDS product transcriptional regulator ICP4 137203-137230 5:02:01
CCAGGGCTCCCGGCGCCGCGGCTCCTCC
137242 137348 137091 141431 CDS product transcriptional regulator ICP4 137242-137348 18:15:04
CCTCTTCGGCCCGGGCGGCCTCTTCTCCGACGACGCCGCCGAGGCCGAGGCCGCCGTCCTCGCGGCCGCGGCCGGAGCCACGAGGCCGCCCCGTCCTCCTTCGGCCC
137423 137449 137091 141431 CDS product transcriptional regulator ICP4 137423-137449 6:03:01
CCCGGGTCCCCGGCCGCCGGGTCCCCC
137426 137456 137091 141431 CDS product transcriptional regulator ICP4 137426-137456 5:02:01
GGGTCCCCGGCCGCCGGGTCCCCCGGTCGGG
137485 137538 137091 141431 CDS product transcriptional regulator ICP4 137485-137538 10:07:02

CCTCGTCCTCGGGCCCCGGTCCCGGGCCGGCTCCGGGCCCCGGCCGCCGACCCC
137495 137527 137091 141431 CDS product transcriptional regulator ICP4 137495-137527 6:03:01
GGGCCCCGGTCCCGGGCCGGCTCCGGGCCCCGG
137554 137584 137091 141431 CDS product transcriptional regulator ICP4 137554-137584 6:03:01
GGCAGAGGCCGGGGCCCCCGGCGGCCCCGGG
137562 137581 137091 141431 CDS product transcriptional regulator ICP4 137562-137581 4:01:01
CCGGGGCCCCCGGCGGCCCC
137592 137649 137091 141431 CDS product transcriptional regulator ICP4 137592-137649 12:09:03
CCCCCTCCGCAGCCTCCCCGGCCTCCTCCTCCGGCTCTCCCGGCCCCTCGGCAGCCCC
137670 137720 137091 141431 CDS product transcriptional regulator ICP4 137670-137720 10:07:02
GGGGGGACCCGGTCGGTGAGCCCGGGCCGGCGGCGCGGCCTCGGACCCCGG
137708 137736 137091 141431 CDS product transcriptional regulator ICP4 137708-137736 5:02:01
CCTCGGACCCCGGCGCCACCAGCACAGCC
137758 137770 137091 141431 CDS product transcriptional regulator ICP4 137758-137770 4:01:01 GGCACGGCGGGGG
137771 137802 137091 141431 CDS product transcriptional regulator ICP4 137771-137802 8:05:02
CCCGCTTCCCCAGCCTCCCCCGCCGCCGGGCC
137798 137826 137091 141431 CDS product transcriptional regulator ICP4 137798-137826 7:04:01
GGGCCGGTCTCGGCGCCCGGCGGCGGCGG
137829 137839 137091 141431 CDS product transcriptional regulator ICP4 137829-137839 4:01:01 CCCCCTCCGCC
137860 137882 137091 141431 CDS product transcriptional regulator ICP4 137860-137882 4:01:01
CCATCACCAGCACCGCGAGCCCC
137934 138016 137091 141431 CDS product transcriptional regulator ICP4 137934-138016 15:12:03
CCCGGAGCCCCGTCTCCTCCAACCCCAACTCCAACTCCAACTCCACCACCACCGTCGCCGTCGAGACCGTCGCCCGCGGCCCC

138022 138059 137091 141431 CDS product transcriptional regulator ICP4 138022-138059 7:04:01
GGACGAGGACGGACTCGGCCTCGCCGGGGACGGCGGGG
138077 138094 137091 141431 CDS product transcriptional regulator ICP4 138077-138094 4:01:01
CCCCGCCGGCGCCGCGCC
138105 138119 137091 141431 CDS product transcriptional regulator ICP4 138105-138119 4:01:01 CCCTCCGCCGCGGCC
138160 138208 137091 141431 CDS product transcriptional regulator ICP4 138160-138208 12:09:03
CCTCTCCCCGGCCCGCTCGCCCTCCGCCCCCCGGGCCCCCGCCGCCGCC
138222 138280 137091 141431 CDS product transcriptional regulator ICP4 138222-138280 14:11:03
CCTCCTCCTCGTCCTCGTCCTCCTCGTCCTCCTCGTCCTCCTCGTCCTCGTCCTCCTCC
138304 138444 137091 141431 CDS product transcriptional regulator ICP4 138304-138444 30:27:07
CCGGCCCGGCGCCCCACTCGCCCGCGCCGGGCCGCCACCGTCTCCGCCGGCGCCCGCCGCGGCCCCGCGGCCCTCGGCCTCCTCCGCCTCCTCCTCCGCCGCGGCCTCCCCGGCCCCGGCCCCGGAGC
CCGCCCGGCCCCC
138406 138428 137091 141431 CDS product transcriptional regulator ICP4 138406-138428 4:01:01
GGCCTCCCCGGCCCCGGCCCCGG
138457 138476 137091 141431 CDS product transcriptional regulator ICP4 138457-138476 5:02:01
CCGCTCCACCAACAACCACC
138489 138516 137091 141431 CDS product transcriptional regulator ICP4 138489-138516 6:03:01
CCGACGGGCCCCCGCCGACCGACGGGCC
138539 138578 137091 141431 CDS product transcriptional regulator ICP4 138539-138578 8:05:02
CCCTGGCCCGGCTCCGACCCTCCGGCCGACGGCCGCGTCC
138631 138676 137091 141431 CDS product transcriptional regulator ICP4 138631-138676 9:06:02
CCGCCAGGCCGCGGCCCGCTACCGCGCCGCGGCCGGGCCCGTGCCC
138688 138706 137091 141431 CDS product transcriptional regulator ICP4 138688-138706 4:01:01

GGAGATGGGGGACTCGAGG

138747 138766 137091 141431 CDS product transcriptional regulator ICP4 138747-138766 4:01:01

CCGCCGGCGAGGCCATGTCC

138811 138865 137091 141431 CDS product transcriptional regulator ICP4 138811-138865 8:05:02

CCAGTTCTGCCAGCGCCGGGTCCACGCGCCCCACGGCCACGGCTCCTTCATCACC

138903 138921 137091 141431 CDS product transcriptional regulator ICP4 138903-138921 4:01:01

CCATGGCCGCCCAGGACCC

138951 138968 137091 141431 CDS product transcriptional regulator ICP4 138951-138968 4:01:01

CCGTGGCCATGAGCCGCC

138981 139063 137091 141431 CDS product transcriptional regulator ICP4 138981-139063 15:12:03

CCCAGAAGACCTTCATCCTGCAGAGCCTCCGCCGGGCCTACGCCGACATGGCCTACCCGGGCCGCGCCGCGGACCCCCGCGCC

139074 139139 137091 141431 CDS product transcriptional regulator ICP4 139074-139139 11:08:02

CCGTCGAGGCCCTCTGCGCCCGCGTCCGCGCCGCCTTCGCCGCCGCGCAGCCCGGCCGCGTGCCCC

139155 139256 137091 141431 CDS product transcriptional regulator ICP4 139155-139256 18:15:04

CCTGCGTCCTGGCCTGCCGCGGCGTCCTCGAGCGCCTGCTGCCCTGCCCGCTCCGGCTGCCCGCGCCCGCCCGCGCCCCGGCCGCCCTCGGGCCCGCCTGCC

139300 139318 137091 141431 CDS product transcriptional regulator ICP4 139300-139318 4:01:01

CCCCGGGGCCGGCCCGGCC

139304 139337 137091 141431 CDS product transcriptional regulator ICP4 139304-139337 7:04:01

GGGGCCGGCCCGGCCGAGCGGCGGCAGGCCGCGG

139347 139371 137091 141431 CDS product transcriptional regulator ICP4 139347-139371 4:01:01

CCCTCGTCGCCCGCACCGTGGCGCC

139398 139569 137091 141431 CDS product transcriptional regulator ICP4 139398-139569 27:24:06

CCCGCGCCCGCGAGGCCGCCTGGACCTACGCCGCGGCCCTCTTCGCCCCGGCCAACGTGGCCGCGGCCCGGCTCGCCGAGGCCGCCGCGCGGCCCGGGCCCGCGGAGCCCGCGCCCGGCCTGCCCCCG

CTCTGGCCCGAGCAGCCCGGCCTCGTCGTCCCAGCGCCGGCGCC
139447 139468 137091 141431 CDS product transcriptional regulator ICP4 139447-139468 4:01:01
GGCCAACGTGGCCGCGGCCCGG
139564 139581 137091 141431 CDS product transcriptional regulator ICP4 139564-139581 5:02:01
GGCGCCGGCGGCGGCGGG
139584 139732 137091 141431 CDS product transcriptional regulator ICP4 139584-139732 30:27:07
CCCCCTCCGGCCTCCCCGGCTCCGGGCCCTCCAGCCCCGCCAGCACCAAGTCCAGCTCCAGCACCAAGTCCAGCTCCAGCACCAAGTCCGGCCTCTCCGGCTCCTCCGGCTACGCCAGCTCCCCCGCC
GCCGGCCCGGACCCAGCGCCC
139753 139777 137091 141431 CDS product transcriptional regulator ICP4 139753-139777 5:02:01
CCGCGCGCCCGGGGCCCGCCGCCCC
139778 139814 137091 141431 CDS product transcriptional regulator ICP4 139778-139814 7:04:01
GGGGACGGCGAGGAGGACGAGGGGCTCTCCGGCGCGG
139855 139870 137091 141431 CDS product transcriptional regulator ICP4 139855-139870 4:01:01 GGACCGGGGGCCCCGG
139898 139918 137091 141431 CDS product transcriptional regulator ICP4 139898-139918 4:01:01
CCCGCTCCGGACCCCGCTCCC
139929 139948 137091 141431 CDS product transcriptional regulator ICP4 139929-139948 7:04:01
CCTCCTCCTCCTCCTCCTCC
139957 140002 137091 141431 CDS product transcriptional regulator ICP4 139957-140002 7:04:01
CCGGCTCCGGCGCCCGCTCGGGCCCATGCCCGAGCACCCCGCGCCC
140014 140111 137091 141431 CDS product transcriptional regulator ICP4 140014-140111 16:13:04
CCGCCGCGTCCCGGCCGGCGAGACCCACACGCCGCGGCCCTCCGAGGCGGCCCTCGCCGCCTACTGCCCGCCCGAGGTGGCCCGCGCCCTCGTGGACC
140148 140165 137091 141431 CDS product transcriptional regulator ICP4 140148-140165 4:01:01
CCTTCGACCCGGCCGCCC

140178 140215 137091 141431 CDS product transcriptional regulator ICP4 140178-140215 8:05:02
CCGCGCGCCGCGGCGCCCCGCTCCGCCGCCGCGCCGCC
140295 140348 137091 141431 CDS product transcriptional regulator ICP4 140295-140348 9:06:02
CCGAGCCCGCCGAGGGCGCCCCGCGCCCGGCCTGGGACCCGCGCCGCGGCGGCC
140364 140381 137091 141431 CDS product transcriptional regulator ICP4 140364-140381 5:02:01
CCGCCTTCGCCCACCGCC
140406 140425 137091 141431 CDS product transcriptional regulator ICP4 140406-140425 4:01:01
GGGCCGGGAACTGGACCGGG
140473 140504 137091 141431 CDS product transcriptional regulator ICP4 140473-140504 5:02:01
GGCGCGGGACCTCGGCTTCGCCGGCGCCGTGG
140520 140542 137091 141431 CDS product transcriptional regulator ICP4 140520-140542 5:02:01
GGCTCGGCGCGGCGCGGCGCCGG
140566 140597 137091 141431 CDS product transcriptional regulator ICP4 140566-140597 7:04:01
GGACTGGCCCGCGGACGGCCCGGCCGTGGGGG
140614 140647 137091 141431 CDS product transcriptional regulator ICP4 140614-140647 7:04:01
CCGCGTCCGCCTGGACCCGGCCGCCCAGTGCGCC
140657 140683 137091 141431 CDS product transcriptional regulator ICP4 140657-140683 4:01:01
CCCGGGTGCCGCGAGCTCCGCGCGGCC
140736 140786 137091 141431 CDS product transcriptional regulator ICP4 140736-140786 10:07:02
CCTCCTTCGCCCGCCTGCACCCCGGGGCCGAGCCGCTGCGCCTCTGCCGCC
140814 140864 137091 141431 CDS product transcriptional regulator ICP4 140814-140864 10:07:02
CCCGCGCCGGGCCCCGGACCCCGGTCCCGCTGCCGCCGCGCGCCTACCGCC
140884 140903 137091 141431 CDS product transcriptional regulator ICP4 140884-140903 4:01:01

GGACGGCTGCAAGGACATGG
141021 141073 137091 141431 CDS product transcriptional regulator ICP4 141021-141073 10:07:02
CCTGCGGCCGCCGGGGCCTCGCCGAGCTCCGCGGCCCCGAGGGCCTGCCGGCC
141110 141129 137091 141431 CDS product transcriptional regulator ICP4 141110-141129 4:01:01
CCCGACGCCGAGGCCGCCCC
141141 141178 137091 141431 CDS product transcriptional regulator ICP4 141141-141178 7:04:01
CCCCGGGCGCCGTCGCCGCCGCGGGCGCCCCGCCGGCC
141195 141283 137091 141431 CDS product transcriptional regulator ICP4 141195-141283 15:12:03
CCCCCTTCGAGACCAGCGTCCGCGCCGCCGCCGGGGGCGCCGTGGAGACCCACCGGCCCGCGGGGGCCTCGGGCGCCGGGGCCGGCCCC
141249 141305 137091 141431 CDS product transcriptional regulator ICP4 141249-141305 10:07:02
GGCCCGCGGGGGCCTCGGGCGCCGGGGCCGGCCCCGGCGAGGACGGGGACTCTGTGG
141314 141355 137091 141431 CDS product transcriptional regulator ICP4 141314-141355 7:04:01
GGCGTCCGCGGCGGCGACGGCCGGCCCCGCGGCCCGCTCGGG
141334 141358 137091 141431 CDS product transcriptional regulator ICP4 141334-141358 4:01:01
CCGGCCCCGCGGCCCGCTCGGGCCC
141364 141405 137091 141431 CDS product transcriptional regulator ICP4 141364-141405 8:05:02
GGTGGAGGCCATCTCGGACGACGAGGAGGCCGAGGACGCCGG
141393 141425 137091 141431 CDS product transcriptional regulator ICP4 141393-141425 5:02:01
CCGAGGACGCCGGCAACCCCTACCTGCTGCTCC

gi|216905852|ref|NC_011644.1| Equid herpesvirus 9, complete genome
873 915 848 1456 CDS product membrane protein UL56 873-915 9:06:02 CCGCGCCTCCTCTGTCTCCATCTCCATGTGCCCACCGCCACCC
1043 1083 848 1456 CDS product membrane protein UL56 1043-1083 7:04:01

CCAGAAGCTCCAGCCTGCCTTCTACCGGCTCCCTGAGATCC
1108 1131 848 1456 CDS product membrane protein UL56 1108-1131 5:02:01 CCGTCGAGGCCGCCGTCCTACACC
1172 1189 848 1456 CDS product membrane protein UL56 1172-1189 4:01:01 GGCCGCGGGCGTGGACGG
1226 1245 848 1456 CDS product membrane protein UL56 1226-1245 4:01:01 CCCTGTGCCAGGCTCCGCCC
1320 1332 848 1456 CDS product membrane protein UL56 1320-1332 4:01:01 CCCCCACCAGCCC
1503 1519 1495 2109 CDS product membrane protein V1 1503-1519 4:01:01 CCCCTTTCCAAACCCCC
1758 1782 1495 2109 CDS product membrane protein V1 1758-1782 4:01:01 GGCATCGGTACAGCGGCCCGTGGGG
1895 1911 1495 2109 CDS product membrane protein V1 1895-1911 4:01:01 CCGGACCCTGCCAGGCC
1908 1924 1495 2109 CDS product membrane protein V1 1908-1924 4:01:01 GGCCGACGGGGGGAAGG
2663 2689 2386 3159 CDS product myristylated tegument protein CIRC 2663-2689 5:02:01
GGTCTTTGGCTACGAGGACGGTACCGG
2882 2917 2386 3159 CDS product myristylated tegument protein CIRC 2882-2917 6:03:01
CCTACTGGGCCACACCAAGAGCTCCACGCACCGTCC
2958 2987 2386 3159 CDS product myristylated tegument protein CIRC 2958-2987 5:02:01
CCCCCCACGCAACCGAGTAGCCAAAGGCCC
2983 3001 2386 3159 CDS product myristylated tegument protein CIRC 2983-3001 4:01:01
GGCCCAAAGGTAAGGGAGG
4051 4074 4014 5426 CDS product multifunctional expression regulator 4051-4074 4:01:01
GGTAAACCGGAACTAGGGTGTAGG
4941 4957 4014 5426 CDS product multifunctional expression regulator 4941-4957 4:01:01
CCCGCGCCGCCATGTCC
5021 5054 4014 5426 CDS product multifunctional expression regulator 5021-5054 5:02:01
CCGCTTTGGCCAGCTCCGCGCTGCCTGGTGCGCC

5141 5160 4014 5426 CDS product multifunctional expression regulator 5141-5160 4:01:01
CCCCTGGCCAAACGCCGGCC
5720 5739 5560 6591 CDS product envelope glycoprotein K 5720-5739 6:03:01 GGGTGGCGGGGGTTGGAGGG
6569 6581 5560 6591 CDS product envelope glycoprotein K 6569-6581 4:01:01 CCGTCCTACCCCC
6921 6937 6605 9844 CDS product helicase-primase primase subunit 6921-6937 4:01:01
CCACCTCACCGAGCACC
7223 7249 6605 9844 CDS product helicase-primase primase subunit 7223-7249 5:02:01
CCTCCATAAACCTCACCAGAGTCGCCC
7265 7282 6605 9844 CDS product helicase-primase primase subunit 7265-7282 4:01:01
GGAAGGCGAAGGCTGCGG
7401 7440 6605 9844 CDS product helicase-primase primase subunit 7401-7440 9:06:02
GGGCTTCCGGTCCGGCCAGGAGGTAAGGGGGCGGCACTGG
8166 8183 6605 9844 CDS product helicase-primase primase subunit 8166-8183 4:01:01
GGCGCTTGGAAGGCAGGG
8207 8226 6605 9844 CDS product helicase-primase primase subunit 8207-8226 4:01:01
CCGGGAACCCCCTTTTGGCC
8410 8425 6605 9844 CDS product helicase-primase primase subunit 8410-8425 4:01:01 CCAGCCGCGGCCCGCC
8995 9012 6605 9844 CDS product helicase-primase primase subunit 8995-9012 4:01:01
GGGGTTTGGTGCGGCCGG
9043 9074 6605 9844 CDS product helicase-primase primase subunit 9043-9074 5:02:01
GGCCTGCAGGTCGTAGGTGCCCCCGGATCTGG
9465 9485 6605 9844 CDS product helicase-primase primase subunit 9465-9485 4:01:01
CCAACGCCCGGGCTCCCGTCC

9545 9558 6605 9844 CDS product helicase-primase primase subunit 9545-9558 4:01:01 CCTCCCACCATACC
9643 9661 6605 9844 CDS product helicase-primase primase subunit 9643-9661 4:01:01 CCAACCGTCACCAGTGTCC
9928 9955 9843 10580 CDS product tegument protein UL51 9928-9955 5:02:01 CCCGGCTACCATGCTCCGCCTACAGTCC
10119 10141 9843 10580 CDS product tegument protein UL51 10119-10141 4:01:01 CCGACAACCCGAACCTGAACGCC
10185 10208 9843 10580 CDS product tegument protein UL51 10185-10208 4:01:01 CCTGCCTCGCGGCCCTAATGCACC
10690 10710 10685 11665 CDS product deoxyuridine triphosphatase 10690-10710 4:01:01 CCCGGTTGAGCCAAACCCTCC
11680 11721 11634 11936 CDS product envelope glycoprotein N 11680-11721 8:05:02 GGTGGTGCTTGGTCTGGCTAGAGGGGCTGGTGGCGACCTAGG
11871 11890 11634 11936 CDS product envelope glycoprotein N 11871-11890 4:01:01 CCTGCCAGGCATACCGCGCC
12414 12425 12098 13015 CDS product tegument protein VP22 12414-12425 4:01:01 GGTTGGCGGTGG
12437 12459 12098 13015 CDS product tegument protein VP22 12437-12459 5:02:01 CCTCCCATGGCCGCCAAAGCCCC
12494 12524 12098 13015 CDS product tegument protein VP22 12494-12524 6:03:01 CCGCACCACCCCCACGTGTTCCAACGCGACC
12895 12921 12098 13015 CDS product tegument protein VP22 12895-12921 4:01:01 GGATACGCGGCAGCTGGGCCCAACGGG
13269 13293 13149 14498 CDS product transactivating tegument protein VP16 13269-13293 4:01:01 CCATCGAGTCCGTTTATTCCAGTCC
13316 13339 13149 14498 CDS product transactivating tegument protein VP16 13316-13339 6:03:01 CCACCGCCCAAGGCCGCCAGCCCC
13356 13386 13149 14498 CDS product transactivating tegument protein VP16 13356-13386 5:02:01 GGCTACAAGGCGAGCTGGGTTTCCCGGAGGG
13526 13549 13149 14498 CDS product transactivating tegument protein VP16 13526-13549 4:01:01 CCCACTCCCAGCCACTATAGCCCC

13580 13601 13149 14498 CDS product transactivating tegument protein VP16 13580-13601 4:01:01
CCCTTCCCAGAGGTTCCAGCCC
13816 13837 13149 14498 CDS product transactivating tegument protein VP16 13816-13837 4:01:01
CCGCGAGGCCGCTAACCTGGCC
13884 13904 13149 14498 CDS product transactivating tegument protein VP16 13884-13904 4:01:01
CCTGGCGCCTGCATGCCAGCC
14317 14368 13149 14498 CDS product transactivating tegument protein VP16 14317-14368 10:07:02
CCCGCCCTCCCCCAGCGCCGTGCTACCCGGTGACCCGGTCCCACCTCTTACC
15148 15176 14871 17477 CDS product tegument protein VP13/14 15148-15176 4:01:01
CCCCGCACAGCCACAAGCACCCAGACCCC
15220 15237 14871 17477 CDS product tegument protein VP13/14 15220-15237 4:01:01 GGAAGAGGAGGAAGATGG
15392 15413 14871 17477 CDS product tegument protein VP13/14 15392-15413 4:01:01 GGTGAAATGGGAGCCGGGGCGG
15727 15743 14871 17477 CDS product tegument protein VP13/14 15727-15743 4:01:01 GGGGGACTACATGGAGG
15834 15849 14871 17477 CDS product tegument protein VP13/14 15834-15849 4:01:01 CCAACCACACCAACCC
16320 16343 14871 17477 CDS product tegument protein VP13/14 16320-16343 5:02:01 GGGAGAGGCGAAAGGAAACGGGGG
16605 16630 14871 17477 CDS product tegument protein VP13/14 16605-16630 4:01:01
CCCACTCCGCAAGTCCCAGCTACACC
16642 16655 14871 17477 CDS product tegument protein VP13/14 16642-16655 4:01:01 GGTGGCAATGGAGG
16984 17004 14871 17477 CDS product tegument protein VP13/14 16984-17004 4:01:01 GGGGGTGTTTTTGGGCGTGGG
17005 17033 14871 17477 CDS product tegument protein VP13/14 17005-17033 6:03:01
CCTAATCCTTCAGCGCCTGGCCGGCCACC
17219 17240 14871 17477 CDS product tegument protein VP13/14 17219-17240 5:02:01 CCGCGACCGGGTCCTCCTACCC
17321 17351 14871 17477 CDS product tegument protein VP13/14 17321-17351 6:03:01

GGCGCAGGCCTGGGCCGCATGGTGGACCTGG

17397 17407 14871 17477 CDS product tegument protein VP13/14 17397-17407 4:01:01 CCACCTCCTCC

17629 17640 17628 19862 CDS product tegument protein VP11/12 17629-17640 4:01:01 GGACGGAGGGGG

17986 18007 17628 19862 CDS product tegument protein VP11/12 17986-18007 4:01:01 CCTCCGAGAGTCCAGCGGCGCC

18023 18038 17628 19862 CDS product tegument protein VP11/12 18023-18038 4:01:01 GGCGCGGTGGGCCAGG

18465 18478 17628 19862 CDS product tegument protein VP11/12 18465-18478 4:01:01 CCACCGCCGACGCC

18642 18659 17628 19862 CDS product tegument protein VP11/12 18642-18659 4:01:01 GGCTCGATGGCGGCGTGG

18944 18973 17628 19862 CDS product tegument protein VP11/12 18944-18973 6:03:01

CCTCCCCAAGGGCCGCTACCGGCACCCCCC

19268 19288 17628 19862 CDS product tegument protein VP11/12 19268-19288 4:01:01 CCCTCAACTCCATCGCCAACC

19305 19324 17628 19862 CDS product tegument protein VP11/12 19305-19324 4:01:01 CCGATCCGCTCCTGGGGCCC

19544 19561 17628 19862 CDS product tegument protein VP11/12 19544-19561 4:01:01 CCACCAGCCAATTCTCCC

19572 19582 17628 19862 CDS product tegument protein VP11/12 19572-19582 4:01:01 CCGCCGCCTCC

20555 20584 20016 20699 CDS product membrane protein UL45 20555-20584 7:04:01

GGTGGGTGTAGGGGGAGTTGGCAGTGGGGG

21799 21811 20971 22377 CDS product envelope glycoprotein C 21799-21811 4:01:01 CCGGCCGTCCGCC

22679 22694 22555 23760 CDS product envelope protein UL43 22679-22694 4:01:01 CCCCCAATATGCCCCC

22769 22789 22555 23760 CDS product envelope protein UL43 22769-22789 4:01:01 GGGCCGTGGAGTGGATGCCGG

23411 23439 22555 23760 CDS product envelope protein UL43 23411-23439 5:02:01

CCGATCCCGCGACCCAGACACCGGCGGCC

24207 24227 24005 25225 CDS product DNA polymerase processivity subunit 24207-24227 5:02:01

GGCACGGCTGGGTCGGGTAGG

26493 26514 25789 27282 CDS product tegument host shutoff protein 26493-26514 5:02:01

CCGCTGTTTCCCCCAACCCTCC

26582 26610 25789 27282 CDS product tegument host shutoff protein 26582-26610 5:02:01 CCACACGGACCTCCACGAGCCCCAAACCC

26884 26911 25789 27282 CDS product tegument host shutoff protein 26884-26911 6:03:01 GGCCTCGGCGGTCCGTGGAGGCCTCCGG

26991 27006 25789 27282 CDS product tegument host shutoff protein 26991-27006 4:01:01 CCCCCCAAAACCCGCC

27092 27112 25789 27282 CDS product tegument host shutoff protein 27092-27112 4:01:01 CCTACCCATCACCCAGGAGCC

27131 27174 25789 27282 CDS product tegument host shutoff protein 27131-27174 6:03:01 CCACGATACCCTTAGAAACCTGGTCTCCGAACCCGAGATTGCCC

27905 27955 27424 28389 CDS product ribonucleotide reductase subunit 2 27905-27955 10:07:02 CCGCCACCGATCCACATTCCCGCACCTTTGCCTGTAGCCAGGCAACCTTCC

30065 30086 28431 30803 CDS product ribonucleotide reductase subunit 1 30065-30086 4:01:01 CCCGGCCTCTTCCATCAGAGCC

30196 30212 28431 30803 CDS product ribonucleotide reductase subunit 1 30196-30212 4:01:01 CCTGTCCAGCCAGCGCC

30239 30272 28431 30803 CDS product ribonucleotide reductase subunit 1 30239-30272 6:03:01 CCAGCCTACTTCCCCGCTGACCACCACGCGTGCC

30678 30696 28431 30803 CDS product ribonucleotide reductase subunit 1 30678-30696 4:01:01 GGGCGGGGAGGCAGCGTGG

31061 31081 31045 32442 CDS product capsid triplex subunit 1 31061-31081 4:01:01 CCCAATCCTGTGGGTCCCACC

31149 31176 31045 32442 CDS product capsid triplex subunit 1 31149-31176 5:02:01 GGGGGAGTCTGAGGCTATGGCCCCCAGG

31316 31355 31045 32442 CDS product capsid triplex subunit 1 31316-31355 8:05:02
GGTCCTGGAGCGGGAGGGCGGCGTTTGGGTGCGGCTCTGG
31457 31479 31045 32442 CDS product capsid triplex subunit 1 31457-31479 5:02:01 CCGCCACGTACACCCTCACCCCC
31630 31646 31045 32442 CDS product capsid triplex subunit 1 31630-31646 4:01:01 CCCTCCTGAACACCCCC
31653 31666 31045 32442 CDS product capsid triplex subunit 1 31653-31666 4:01:01 GGCGGTGAGGCTGG
31859 31878 31045 32442 CDS product capsid triplex subunit 1 31859-31878 4:01:01 CCCCGGACCTGGATGCCACC
31905 31971 31045 32442 CDS product capsid triplex subunit 1 31905-31971 10:07:02
CCGCAGCCCCGTCGTATCCGAGCGACCGGACACCGAGAGTTCCGCCAGCGCGGCCCAGGCCTCGTCC
32022 32063 31045 32442 CDS product capsid triplex subunit 1 32022-32063 8:05:02
GGCATCGAGGTGGTTACGGAGGCGGATTATCGGAAGTCCTGG
32895 32913 32819 35884 CDS product tegument protein UL37 32895-32913 4:01:01 CCCCAGCCTCGAGCCTGCC
32967 32989 32819 35884 CDS product tegument protein UL37 32967-32989 5:02:01 GGTGTCGGTGGACGAGGCCGCGG
33110 33136 32819 35884 CDS product tegument protein UL37 33110-33136 7:04:01 GGACGGACAGGGAAATGGAGGCGGTGG
33460 33484 32819 35884 CDS product tegument protein UL37 33460-33484 4:01:01 GGCCTCATGGTCAAGGGATACAAGG
33902 33942 32819 35884 CDS product tegument protein UL37 33902-33942 7:04:01
CCAGTAACACCTGGGAGACCATCCAGGCCAGCACGACCCCC
33994 34018 32819 35884 CDS product tegument protein UL37 33994-34018 5:02:01 GGAATTTTGGAGCGCGTGGTGGTGG
34099 34121 32819 35884 CDS product tegument protein UL37 34099-34121 4:01:01 GGTTGCGTGGCTATAGTGGGTGG
34227 34255 32819 35884 CDS product tegument protein UL37 34227-34255 6:03:01
GGCCCTGGGGCTGGCGGACAAGGGAGTGG
34697 34719 32819 35884 CDS product tegument protein UL37 34697-34719 4:01:01 GGGCCAGGGACTACGGCGCTGGG
34799 34824 32819 35884 CDS product tegument protein UL37 34799-34824 5:02:01 CCCCTACCCCAACCATGGCCCATGCC
34833 34849 32819 35884 CDS product tegument protein UL37 34833-34849 4:01:01 GGAGGCGCTTTTGGGGG

35155 35192 32819 35884 CDS product tegument protein UL37 35155-35192 7:04:01
GGTAGGTGGCGTCTGGTAGACGTGGTCGACGCGGTTGG
35271 35305 32819 35884 CDS product tegument protein UL37 35271-35305 6:03:01
GGCCGCGGAGGCACTGCAGGAGTGCGAGGCCCTGG
35373 35391 32819 35884 CDS product tegument protein UL37 35373-35391 4:01:01 GGCCAGGGCCCAGAGGGGG
35419 35438 32819 35884 CDS product tegument protein UL37 35419-35438 4:01:01 GGGGGTTCCGAGGCTCCAGG
36132 36166 36113 46432 CDS product large tegument protein 36132-36166 6:03:01
GGCGAATAAGGCGGGTGGCGCTCAGGCCGATGTGG
36574 36592 36113 46432 CDS product large tegument protein 36574-36592 4:01:01 GGGGCTACTGGTCTGGCGG
36898 36912 36113 46432 CDS product large tegument protein 36898-36912 4:01:01 CCCCCACCGCTAACC
36918 36945 36113 46432 CDS product large tegument protein 36918-36945 7:04:01 GGCTGTGGGGGCGGTTTCGGAGGCGTGG
37084 37104 36113 46432 CDS product large tegument protein 37084-37104 5:02:01 CCCCCGTGGACGCCTCCCACC
37267 37287 36113 46432 CDS product large tegument protein 37267-37287 4:01:01 CCCGTCCAGGCCGTAGGCGCC
37382 37396 36113 46432 CDS product large tegument protein 37382-37396 4:01:01 GGCTCTCGGGGGAGG
37602 37620 36113 46432 CDS product large tegument protein 37602-37620 4:01:01 GGTGGAAAACGGCACGCGG
37676 37692 36113 46432 CDS product large tegument protein 37676-37692 4:01:01 CCATTCCTACCCAATCC
37851 37879 36113 46432 CDS product large tegument protein 37851-37879 5:02:01
GGAGCTCGTGGTTAAGGTTGAGGAATTGG
37974 38004 36113 46432 CDS product large tegument protein 37974-38004 5:02:01
CCTATTTGCCCGAGAACCGAAACCGGGGGCC
38214 38241 36113 46432 CDS product large tegument protein 38214-38241 6:03:01 CCTCTCCGCCGTTCCTACCCGCGTAGCC
38457 38472 36113 46432 CDS product large tegument protein 38457-38472 4:01:01 CCAGGGACCCCCACCC
38805 38821 36113 46432 CDS product large tegument protein 38805-38821 4:01:01 GGCAAAGGTGGTCGAGG

| | | | | | | | |
|---|---|---|---|---|---|---|---|
| 39165 | 39190 | 36113 | 46432 | CDS product large tegument protein | 39165-39190 | 5:02:01 | CCCAAACTCCACTACCTCATCCGCCC |
| 39192 | 39208 | 36113 | 46432 | CDS product large tegument protein | 39192-39208 | 4:01:01 | GGCGGGAGTGGGCCCGG |
| 39635 | 39648 | 36113 | 46432 | CDS product large tegument protein | 39635-39648 | 4:01:01 | CCGCCGCCACTGCC |
| 39656 | 39673 | 36113 | 46432 | CDS product large tegument protein | 39656-39673 | 4:01:01 | GGGAGCTGGCGAGGCTGG |
| 39911 | 39932 | 36113 | 46432 | CDS product large tegument protein | 39911-39932 | 4:01:01 | GGGACCTCGGTGCGGCTCGCGG |
| 40446 | 40474 | 36113 | 46432 | CDS product large tegument protein | 40446-40474 | 5:02:01 | GGTCGCGGAAAAGGTTTTGGCCGAAGGGG |
| 40499 | 40525 | 36113 | 46432 | CDS product large tegument protein | 40499-40525 | 4:01:01 | CCCTGATAGCCAAAGCCCTTAAGTACC |
| 40791 | 40810 | 36113 | 46432 | CDS product large tegument protein | 40791-40810 | 4:01:01 | GGACAAGGCTACCTCGGCGG |
| 40869 | 40890 | 36113 | 46432 | CDS product large tegument protein | 40869-40890 | 4:01:01 | GGACATTAAGGCCGTGGAGTGG |
| 41051 | 41067 | 36113 | 46432 | CDS product large tegument protein | 41051-41067 | 4:01:01 | GGGATGAGGCGTGGGGG |
| 41196 | 41207 | 36113 | 46432 | CDS product large tegument protein | 41196-41207 | 4:01:01 | CCGCCTACCCCC |
| 41508 | 41526 | 36113 | 46432 | CDS product large tegument protein | 41508-41526 | 5:02:01 | CCGCCCCCACCTGGAAGCC |
| 41846 | 41863 | 36113 | 46432 | CDS product large tegument protein | 41846-41863 | 4:01:01 | GGTTTGGACTGGAAAAGG |
| 42020 | 42038 | 36113 | 46432 | CDS product large tegument protein | 42020-42038 | 4:01:01 | GGTTCATGGCGGGTTCGGG |
| 42433 | 42451 | 36113 | 46432 | CDS product large tegument protein | 42433-42451 | 4:01:01 | GGCGAAAGCGGACGGGCGG |
| 42452 | 42504 | 36113 | 46432 | CDS product large tegument protein | 42452-42504 | 11:08:02 | CCGCAGCCAAGATAGCCTCCGCCATTCCCGGAAACCCCCTAGCCACCTTTACC |
| 42629 | 42648 | 36113 | 46432 | CDS product large tegument protein | 42629-42648 | 4:01:01 | CCTCTACCGTACAGACCTCC |
| 42651 | 42664 | 36113 | 46432 | CDS product large tegument protein | 42651-42664 | 4:01:01 | GGCGGTGGACCCGG |
| 43213 | 43235 | 36113 | 46432 | CDS product large tegument protein | 43213-43235 | 4:01:01 | CCTCCGATGAACCCGGATGACCC |
| 43419 | 43438 | 36113 | 46432 | CDS product large tegument protein | 43419-43438 | 4:01:01 | GGAGGACCCAACGGGTATGG |
| 43491 | 43518 | 36113 | 46432 | CDS product large tegument protein | 43491-43518 | 6:03:01 | GGCGTGGCAAGAGTGGTTGGAGGATGGG |

43547 43580 36113 46432 CDS product large tegument protein 43547-43580 7:04:01 CCAACGCCATACCAGCGCCCCCCAAACCTACTCC
43724 43787 36113 46432 CDS product large tegument protein 43724-43787 15:12:03 CCGTTCCCCCTTCCGTACCTGCTCCTCCCACCCTTCCACCCGCTCCCCCTCTGCCCCAACCCCCC
44068 44106 36113 46432 CDS product large tegument protein 44068-44106 8:05:02 CCAGTTCCACCCACCGATAAGCCGTCAACCACCACTCCC
44266 44285 36113 46432 CDS product large tegument protein 44266-44285 5:02:01 CCCGGAGCCAAACCACCCCC
44459 44517 36113 46432 CDS product large tegument protein 44459-44517 11:08:02 CCCAATCAACCAAGGAGCCCCCAAAGCCTGCCGTAGAGGCCCCCGCCGCGGCCCCGGCC
44630 44656 36113 46432 CDS product large tegument protein 44630-44656 6:03:01 CCCCCGCCGCGGCCCCGGCCAAAGACC
44688 44797 36113 46432 CDS product large tegument protein 44688-44797 19:16:04 GGCCAAGGACCAGGCCAAGGACCAGGCCAAGGACCAGGCCAAGGACCAGGCCAAGGACCAGGCCAAGGACCAGGCCAAGGACCAGGCCAAGGACCAGGCCAAGGACCAGG
45138 45178 36113 46432 CDS product large tegument protein 45138-45178 7:04:01 CCCATTGCCCGATTCTCCCACCGACGAACCCTCCAGCGGCC
45514 45527 36113 46432 CDS product large tegument protein 45514-45527 4:01:01 CCGGCCACCATTCC
45676 45728 36113 46432 CDS product large tegument protein 45676-45728 11:08:02 CCTCAGCCGCCACCAGCAAACCAGACACCACCGCCTCACGAACCCCCAGCACC
47098 47117 46982 47809 CDS product nuclear egress membrane protein 47098-47117 4:01:01 GGAGATGCTGGTAAGGCTGG
47124 47138 46982 47809 CDS product nuclear egress membrane protein 47124-47138 4:01:01 CCTCCGCCGCGCACC
47386 47406 46982 47809 CDS product nuclear egress membrane protein 47386-47406 5:02:01 CCTCAGGTCCAGGCCTCCCCC
47677 47694 46982 47809 CDS product nuclear egress membrane protein 47677-47694 4:01:01 GGCTGGTAGGTGTTTGGG
47768 47779 46982 47809 CDS product nuclear egress membrane protein 47768-47779 4:01:01 CCACCACCGACC

48514 48529 48338 50200 CDS product DNA packaging protein UL32 48514-48529 4:01:01 CCCACGCCACCGCCCC
48736 48753 48338 50200 CDS product DNA packaging protein UL32 48736-48753 4:01:01 CCGCCGTGTGCCGTGGCC
48964 48979 48338 50200 CDS product DNA packaging protein UL32 48964-48979 4:01:01 GGACCCGGTATGGCGG
49119 49145 48338 50200 CDS product DNA packaging protein UL32 49119-49145 7:04:01
GGCGGATGGGGGCGGTGGGGTGTTTGG
49407 49428 48338 50200 CDS product DNA packaging protein UL32 49407-49428 4:01:01 CCAGTCCAGTACCTGGGAGGCC
49560 49579 48338 50200 CDS product DNA packaging protein UL32 49560-49579 4:01:01 GGACACGGCCACTGGGCCGG
49806 49818 48338 50200 CDS product DNA packaging protein UL32 49806-49818 4:01:01 GGGTGGCGGTGGG
49954 49976 48338 50200 CDS product DNA packaging protein UL32 49954-49976 5:02:01 CCCATAGGCCCACCTCCAGAACC
50609 50635 50193 51173 CDS product nuclear egress lamina protein 50609-50635 5:02:01
CCTCGCCTTCACCGCGCAGACCGCGCC
50712 50746 50193 51173 CDS product nuclear egress lamina protein 50712-50746 7:04:01
CCGCCGCCCAGGGGGACCCTGCCGGCGTTGCCGCC
51375 51406 51097 54759 CDS product DNA polymerase catalytic subunit 51375-51406 6:03:01
GGAAACATAGGTCGGGTCTTCGGCGAGGTCGG
51427 51469 51097 54759 CDS product DNA polymerase catalytic subunit 51427-51469 8:05:02
GGTTGGGGGCGTGCGGTCTTGGTTGCCACGGGGTTTGGGGCGG
51597 51612 51097 54759 CDS product DNA polymerase catalytic subunit 51597-51612 4:01:01 GGTGAGGTGCGGCAGG
51788 51807 51097 54759 CDS product DNA polymerase catalytic subunit 51788-51807 4:01:01
CCTCTGCGGCCGCCGTGGCC
52039 52056 51097 54759 CDS product DNA polymerase catalytic subunit 52039-52056 4:01:01
CCCAGCTCCGATACCCCC
52119 52140 51097 54759 CDS product DNA polymerase catalytic subunit 52119-52140 4:01:01

GGAGTAAGGCTTGTGGTTTCGG

52279 52301 51097 54759 CDS product DNA polymerase catalytic subunit 52279-52301 5:02:01 CCGTTTGCCACCCCCGTGAATCC

52655 52681 51097 54759 CDS product DNA polymerase catalytic subunit 52655-52681 4:01:01 CCTTTGCTCCCTGGTAGCCCACGGCCC

52690 52730 51097 54759 CDS product DNA polymerase catalytic subunit 52690-52730 8:05:02 CCGGGCTTTCCGCCTCCATTTTCCGAAGCCGCGACCGATCC

53050 53078 51097 54759 CDS product DNA polymerase catalytic subunit 53050-53078 5:02:01 CCTATCACGCCCCGCCGGTCAGGCCCACC

53589 53616 51097 54759 CDS product DNA polymerase catalytic subunit 53589-53616 4:01:01 GGAGATCTGGATAACCAGGTCCTCTTGG

53712 53735 51097 54759 CDS product DNA polymerase catalytic subunit 53712-53735 4:01:01 GGGGATCGGCTCCAGGTTATCTGG

53807 53826 51097 54759 CDS product DNA polymerase catalytic subunit 53807-53826 4:01:01 GGGTGCCGGGCCGCAGGCGG

53942 53956 51097 54759 CDS product DNA polymerase catalytic subunit 53942-53956 4:01:01 GGCGGCTGCTGGAGG

54055 54083 51097 54759 CDS product DNA polymerase catalytic subunit 54055-54083 8:05:02 CCGCCCCACCATCCCCCGCCGCTACCGCC

54094 54117 51097 54759 CDS product DNA polymerase catalytic subunit 54094-54117 5:02:01 CCTCCGTTGCCATTTCCCAAGGCC

54197 54213 51097 54759 CDS product DNA polymerase catalytic subunit 54197-54213 5:02:01 CCACCTCCGCCTTTGCC

54406 54454 51097 54759 CDS product DNA polymerase catalytic subunit 54406-54454 8:05:02

CCATCGCCCCGAAAATCCTGGCCGTTCCAAACCCGAATCCTGCGGGGCC

54554 54565 51097 54759 CDS product DNA polymerase catalytic subunit 54554-54565 4:01:01 GGTCGGCGGGGG

54632 54661 51097 54759 CDS product DNA polymerase catalytic subunit 54632-54661 5:02:01 GGGGGGGCTTGGGGCGCTCGGTCTCCGAGG

55228 55244 55016 58645 CDS product single-stranded DNA-binding protein 55228-55244 4:01:01 GGACTGGGTGGTGCTGG

55331 55345 55016 58645 CDS product single-stranded DNA-binding protein 55331-55345 4:01:01 CCCCCAACCTCACCC

55356 55376 55016 58645 CDS product single-stranded DNA-binding protein 55356-55376 5:02:01 GGCGGCTAGACGGAGGTTTGG

55452 55470 55016 58645 CDS product single-stranded DNA-binding protein 55452-55470 4:01:01 CCTGTCTCCGGAGACCACC

55792 55813 55016 58645 CDS product single-stranded DNA-binding protein 55792-55813 4:01:01 GGAGCGGCCCACCTGGCCTTGG

55879 55901 55016 58645 CDS product single-stranded DNA-binding protein 55879-55901 4:01:01 GGAAACTCCGGCAAGGGTTCTGG

55917 55932 55016 58645 CDS product single-stranded DNA-binding protein 55917-55932 4:01:01 GGGGGACGGTAGCGGG

56134 56152 55016 58645 CDS product single-stranded DNA-binding protein 56134-56152 4:01:01 GGACTGGTTGGAGCCATGG

56230 56265 55016 58645 CDS product single-stranded DNA-binding protein 56230-56265 7:04:01 CCTTCCTACCACCGCTTCTACCTAATAGCCGCTCCC

56753 56773 55016 58645 CDS product single-stranded DNA-binding protein 56753-56773 4:01:01 GGGCCACGATGGAGCGGCTGG

56825 56847 55016 58645 CDS product single-stranded DNA-binding protein 56825-56847 5:02:01

CCAGCCCCTGCTCAGCCCCCACC

57271 57291 55016 58645 CDS product single-stranded DNA-binding protein 57271-57291 5:02:01 CCCGCCTCAGAGCCACCCACC

57393 57424 55016 58645 CDS product single-stranded DNA-binding protein 57393-57424 7:04:01 GGAAGCGGCCAGGGCCAGGGTGGCCGGCATGG

57401 57426 55016 58645 CDS product single-stranded DNA-binding protein 57401-57426 4:01:01 CCAGGGCCAGGGTGGCCGGCATGGCC

57566 57590 55016 58645 CDS product single-stranded DNA-binding protein 57566-57590 4:01:01 CCCTGCTCCAGCGCAACCAGATGCC

58038 58067 55016 58645 CDS product single-stranded DNA-binding protein 58038-58067 6:03:01 GGCAGGCAACTGGAACGGCCTCAACGGCGG

58149 58171 55016 58645 CDS product single-stranded DNA-binding protein 58149-58171 6:03:01 GGCCGGAGGATTTGGCACGGGGG

58304 58318 55016 58645 CDS product single-stranded DNA-binding protein 58304-58318 4:01:01 GGATCGGTCTGGTGG

58378 58411 55016 58645 CDS product single-stranded DNA-binding protein 58378-58411 6:03:01 GGAGAGTGGAGCGTGGAGGCTGCCCAGGAGCTGG

58525 58540 55016 58645 CDS product single-stranded DNA-binding protein 58525-58540 4:01:01 GGTGGCCTGGCCATGG

59348 59376 58804 61131 CDS product DNA packaging terminase subunit 2 59348-59376 5:02:01 GGTGGGCAGCGGTATCGTGGTACCCCCGG

59387 59405 58804 61131 CDS product DNA packaging terminase subunit 2 59387-59405 4:01:01 CCCCACCCATCCCTGCTCC

59569 59612 58804 61131 CDS product DNA packaging terminase subunit 2 59569-59612 7:04:01 GGCGCGCGGCCGCGGAAGCGGCTCTCCGGGCCCTCGAGGCCAGG

60126 60144 58804 61131 CDS product DNA packaging terminase subunit 2 60126-60144 4:01:01 GGCGCCGGCGAGTTGGGGG
60560 60590 58804 61131 CDS product DNA packaging terminase subunit 2 60560-60590 6:03:01 CCACCCCATCGACCAGTCCCTCATGCCGTCC
61029 61053 58804 61131 CDS product DNA packaging terminase subunit 2 61029-61053 5:02:01 GGGGCCATTGGCGCGGAGACGGTGG
61407 61432 61290 63935 CDS product envelope glycoprotein B 61407-61432 7:04:01 CCTGCCCACCGCCTACCGGATCCACC
61629 61649 61290 63935 CDS product envelope glycoprotein B 61629-61649 4:01:01 GGGTTCCGGTTTCAGTGGAGG
61787 61813 61290 63935 CDS product envelope glycoprotein B 61787-61813 5:02:01 CCGGGGGCCAGAGCCTGGCAGACCACC
61834 61852 61290 63935 CDS product envelope glycoprotein B 61834-61852 4:01:01 GGGGTGGATGCCATGGAGG
61888 61903 61290 63935 CDS product envelope glycoprotein B 61888-61903 5:02:01 GGAGGTGGAGGCGCGG
61905 61939 61290 63935 CDS product envelope glycoprotein B 61905-61939 6:03:01 CCGTCTACCCCTACGACTCCTTCGCCCTGTCCACC
62478 62495 61290 63935 CDS product envelope glycoprotein B 62478-62495 4:01:01 CCAACCGCACCTACGACC
62560 62588 61290 63935 CDS product envelope glycoprotein B 62560-62588 6:03:01 CCTGTCCGTACCAGAACCTCAGCCAACCC
62641 62655 61290 63935 CDS product envelope glycoprotein B 62641-62655 4:01:01 GGAGGCAACGGCAGG
62658 62680 61290 63935 CDS product envelope glycoprotein B 62658-62680 4:01:01 CCGATTCTTCCAACGTCACCGCC
63436 63465 61290 63935 CDS product envelope glycoprotein B 63436-63465 6:03:01 GGGCCTGGGTAAAGTGGGGGAGGCCGTGGG
63678 63700 61290 63935 CDS product envelope glycoprotein B 63678-63700 5:02:01 CCAAGACTTCCTCCGGCCAGACC
63775 63797 61290 63935 CDS product envelope glycoprotein B 63775-63797 4:01:01 GGTTTCGGCCCTGGAAAAGCAGG
64177 64200 64137 64616 CDS product protein V32 64177-64200 4:01:01 CCGCACCGAGGCCGCAGTTTCCCC

64547 64565 64137 64616 CDS product protein V32 64547-64565 4:01:01 CCCGACACTCCCACCGCCC
64759 64776 64701 65690 CDS product capsid scaffold protein 64759-64776 4:01:01 GGCCGGTGGACGCACTGG
64960 65007 64701 65690 CDS product capsid scaffold protein 64960-65007 8:05:02 GGGTTGGCTGAGAGGGGATTCCTGGTATCTGCGGAGCAAGGATGGCGG
65052 65071 64701 65690 CDS product capsid scaffold protein 65052-65071 4:01:01 GGAGAAACGGCGGGTATGGG
65088 65115 64701 65690 CDS product capsid scaffold protein 65088-65115 5:02:01 GGAGCGGCTTGGGGCTGCGCGGTCACGG
65183 65202 64701 65690 CDS product capsid scaffold protein 65183-65202 5:02:01 GGTGGTGGACGGGCGCCCGG
65484 65512 64701 65690 CDS product capsid scaffold protein 65484-65512 6:03:01 GGCGGCATCAGGGAATTGTAGGCCGGCGG
65841 65866 64701 66641 CDS product capsid maturation protease 65841-65866 5:02:01 CCGCATGTGCCTACCAGCTCCGCCCC
65933 65955 64701 66641 CDS product capsid maturation protease 65933-65955 4:01:01 CCCTTCTATGCCAGCCTCGCGCC
66050 66068 64701 66641 CDS product capsid maturation protease 66050-66068 4:01:01 CCACGTGGCCGCATCCCCC
66311 66331 64701 66641 CDS product capsid maturation protease 66311-66331 4:01:01 CCGCTCCTGCTCCGAAAGCCC
66451 66469 64701 66641 CDS product capsid maturation protease 66451-66469 4:01:01 CCTCCCCGACCACACACCC
66507 66537 64701 66641 CDS product capsid maturation protease 66507-66537 5:02:01 GGGGGAAGCGCGGCTGCGACGGTTTCCCTGG
66961 66978 66763 68526 CDS product DNA packaging tegument protein UL25 66961-66978 4:01:01 GGTACCGGGGCGGTCCGG
67146 67162 66763 68526 CDS product DNA packaging tegument protein UL25 67146-67162 4:01:01 GGCGAGGTATATGGGGG
67214 67246 66763 68526 CDS product DNA packaging tegument protein UL25 67214-67246 6:03:01 CCATAGCTGCCGCATCTCCCCCGCTCGCCCCCC

| | | | | | |
|---|---|---|---|---|---|
| 67611 | 67622 | 66763 | 68526 | CDS product DNA packaging tegument protein UL25 | 67611-67622 4:01:01 CCCCCCTCCTCC |
| 67671 | 67704 | 66763 | 68526 | CDS product DNA packaging tegument protein UL25 | 67671-67704 6:03:01 CCCAACCTTTCCGAGAGTTCCGTCTCCGAGTGCC |
| 67785 | 67806 | 66763 | 68526 | CDS product DNA packaging tegument protein UL25 | 67785-67806 4:01:01 GGCGCGATGGGCTAAATGGAGG |
| 67986 | 68018 | 66763 | 68526 | CDS product DNA packaging tegument protein UL25 | 67986-68018 6:03:01 CCAGGTCCCGAATACCACCCCGTTTGACCCGCC |
| 68168 | 68185 | 66763 | 68526 | CDS product DNA packaging tegument protein UL25 | 68168-68185 4:01:01 GGGGTGTGGCGGCGTCGG |
| 68216 | 68242 | 66763 | 68526 | CDS product DNA packaging tegument protein UL25 | 68216-68242 4:01:01 GGGCCGCCAGGGCGTCGGCTACCTTGG |
| 68767 | 68798 | 68629 | 69369 | CDS product nuclear protein UL24 | 68767-68798 5:02:01 GGCTGCTGGGCTGGCGTTATAGGTTGCACGGG |
| 69775 | 69795 | 69460 | 70518 | CDS product thymidine kinase | 69775-69795 5:02:01 CCACGCCCTACCTTATCCTCC |
| 69844 | 69860 | 69460 | 70518 | CDS product thymidine kinase | 69844-69860 4:01:01 CCAGGCCAGACCTAACC |
| 69872 | 69893 | 69460 | 70518 | CDS product thymidine kinase | 69872-69893 5:02:01 CCGCCACCCTGTCGCCTCTGCC |
| 69952 | 69979 | 69460 | 70518 | CDS product thymidine kinase | 69952-69979 5:02:01 CCATGGTCGCCACCCTACCCAGGGAACC |
| 70140 | 70167 | 69460 | 70518 | CDS product thymidine kinase | 70140-70167 5:02:01 GGGAGAGTCTGGCGTGACGGCTGGGGGG |
| 71030 | 71047 | 70743 | 73301 | CDS product envelope glycoprotein H | 71030-71047 4:01:01 CCTCCGCAACACCACCCC |
| 71063 | 71088 | 70743 | 73301 | CDS product envelope glycoprotein H | 71063-71088 4:01:01 CCGGAGCCTGGTGACCCACGCGGACC |
| 71700 | 71717 | 70743 | 73301 | CDS product envelope glycoprotein H | 71700-71717 4:01:01 GGATTTCCCGGGAGGTGG |
| 71941 | 71960 | 70743 | 73301 | CDS product envelope glycoprotein H | 71941-71960 5:02:01 CCTCCGCCTCATTGTCCCCC |
| 72169 | 72187 | 70743 | 73301 | CDS product envelope glycoprotein H | 72169-72187 4:01:01 GGTGACCTTGGCGGACCGG |

| | | | | | | |
|---|---|---|---|---|---|---|
| 73056 | 73075 | 70743 | 73301 | CDS product envelope glycoprotein H | 73056-73075 4:01:01 | CCATCCCGGCCTTTAATCCC |
| 73140 | 73162 | 70743 | 73301 | CDS product envelope glycoprotein H | 73140-73162 4:01:01 | CCGCCTTCGCGTCCTATTCATCC |
| 74304 | 74323 | 74168 | 75760 | CDS product tegument protein UL21 | 74304-74323 4:01:01 | CCACCGGTAGGCCGCTTCCC |
| 74340 | 74361 | 74168 | 75760 | CDS product tegument protein UL21 | 74340-74361 5:02:01 | CCTGCGCCGCTCCCCCGATTCC |
| 74525 | 74540 | 74168 | 75760 | CDS product tegument protein UL21 | 74525-74540 4:01:01 | GGATCGGTGGGTTGGG |
| 74611 | 74637 | 74168 | 75760 | CDS product tegument protein UL21 | 74611-74637 5:02:01 | GGCCACCAGGTGTGGGTCTGGGAGTGG |
| 74803 | 74824 | 74168 | 75760 | CDS product tegument protein UL21 | 74803-74824 4:01:01 | CCCCTCGTTGCCGTATACCGCC |
| 74871 | 74890 | 74168 | 75760 | CDS product tegument protein UL21 | 74871-74890 4:01:01 | CCAACCCGGGCCACGGTTCC |
| 74951 | 74978 | 74168 | 75760 | CDS product tegument protein UL21 | 74951-74978 4:01:01 | CCGCTGGCCACGCGACCCGAAGAATCCC |
| 74989 | 75013 | 74168 | 75760 | CDS product tegument protein UL21 | 74989-75013 4:01:01 | GGGCTTTGGAATCAGGCGCACTTGG |
| 75042 | 75062 | 74168 | 75760 | CDS product tegument protein UL21 | 75042-75062 4:01:01 | GGGGGCGTTTGGAGCACCGGG |
| 75209 | 75226 | 74168 | 75760 | CDS product tegument protein UL21 | 75209-75226 4:01:01 | GGGGGTAACTCGGTTAGG |
| 75631 | 75651 | 74168 | 75760 | CDS product tegument protein UL21 | 75631-75651 4:01:01 | GGACGTGGCATGTGGTCGAGG |
| 77560 | 77590 | 77244 | 81374 | CDS product major capsid protein | 77560-77590 6:03:01 | CCCACATCCTGTGGACCAGCCTACCCACACC |
| 77644 | 77670 | 77244 | 81374 | CDS product major capsid protein | 77644-77670 5:02:01 | GGCCGAGGCCCTGGGCCTGATTGGGGG |
| 78006 | 78019 | 77244 | 81374 | CDS product major capsid protein | 78006-78019 4:01:01 | CCCCCTCCGTCGCC |
| 78394 | 78417 | 77244 | 81374 | CDS product major capsid protein | 78394-78417 4:01:01 | CCAGGCGACCAACGTTCCGTACCC |
| 78470 | 78482 | 77244 | 81374 | CDS product major capsid protein | 78470-78482 4:01:01 | CCGGCCACCGACC |
| 78512 | 78556 | 77244 | 81374 | CDS product major capsid protein | 78512-78556 9:06:02 | CCAACCCCCGGGCAGCCAGACCCTCGCACCTACCCACCCCAAACC |
| 78705 | 78733 | 77244 | 81374 | CDS product major capsid protein | 78705-78733 6:03:01 | CCTACGTGACCCTACCCCCAGCCGGCACC |

78985 79002 77244 81374 CDS product major capsid protein 78985-79002 5:02:01 CCCCCTCCCCCTATGTCC
79373 79395 77244 81374 CDS product major capsid protein 79373-79395 4:01:01 GGGGAAGCCGTGGGAGGCCAGGG
79427 79443 77244 81374 CDS product major capsid protein 79427-79443 4:01:01 CCGGCCCTGCTACCGCC
79476 79526 77244 81374 CDS product major capsid protein 79476-79526 9:06:02
GGGCCGACATGGGTAGGGCCAGGGCACAGGATCTATGGGTGGACGGGGTGG
79630 79653 77244 81374 CDS product major capsid protein 79630-79653 4:01:01 CCCGATTGTGCCTCACCACGACCC
79803 79857 77244 81374 CDS product major capsid protein 79803-79857 10:07:02
CCCCAACCAGCCCAAGCGACCCTCGCCATCCGCTGAACCCGCGCCACCTAGTGCC
79887 79925 77244 81374 CDS product major capsid protein 79887-79925 6:03:01
CCAGAGTTGCCGTCGACACCGACGCCCTGCTACTCCTCC
80494 80507 77244 81374 CDS product major capsid protein 80494-80507 4:01:01 GGCGGTTGGGGGGG
80532 80556 77244 81374 CDS product major capsid protein 80532-80556 5:02:01 GGGCTAACGTGGACTTGGGGGTTGG
80710 80733 77244 81374 CDS product major capsid protein 80710-80733 4:01:01 CCTGGGGCCCCAGGGACCCAGGCC
80775 80791 77244 81374 CDS product major capsid protein 80775-80791 4:01:01 CCCACGGCCAGGCCGCC
80942 80973 77244 81374 CDS product major capsid protein 80942-80973 7:04:01
CCGGCCTACCCAAGCCGCGCCACCGTTAACCC
81188 81209 77244 81374 CDS product major capsid protein 81188-81209 4:01:01 GGCTCGACGGACCTGGTTGAGG
81656 81680 81621 82565 CDS product capsid triplex subunit 2 81656-81680 4:01:01
CCCAGTGACCTATCTCCCGCTGACC
82074 82097 81621 82565 CDS product capsid triplex subunit 2 82074-82097 5:02:01 GGCTGGTGGCGCGCGCGGTTCAGG
82202 82220 81621 82565 CDS product capsid triplex subunit 2 82202-82220 4:01:01 GGCGTGGCGCGGTCTCTGG
82277 82301 81621 82565 CDS product capsid triplex subunit 2 82277-82301 4:01:01
CCCAACCTGCTTACCCTGGGAACCC

82357 82389 81621 82565 CDS product capsid triplex subunit 2 82357-82389 6:03:01 CCAGCTCGTCCACCAGCAGCCCGTACCCCAACC

82719 82740 82687 88447 CDS product DNA packaging terminase subunit 1 82719-82740 6:03:01 GGTTGGTGGGAGGCTGGACAGG

83315 83334 82687 88447 CDS product DNA packaging terminase subunit 1 83315-83334 4:01:01 CCACACCGGTTCCTGAAGCC

83953 83984 82687 88447 CDS product DNA packaging terminase subunit 1 83953-83984 6:03:01 CCTGCTACCCGGTTCCATACCCCCGGCGTACC

84149 84178 84016 86136 CDS product DNA packaging tegument protein UL17 84149-84178 6:03:01 CCGCCATAGAGCCAGCCCATCCGCGGCTCC

84291 84313 84016 86136 CDS product DNA packaging tegument protein UL17 84291-84313 4:01:01 CCGACAGTTCCCGCTGACCACCC

84430 84449 84016 86136 CDS product DNA packaging tegument protein UL17 84430-84449 4:01:01 CCGACGACCCACGGACCACC

85017 85040 84016 86136 CDS product DNA packaging tegument protein UL17 85017-85040 4:01:01 CCCCCTCAGGGACCCAGGAGCCCC

85102 85119 84016 86136 CDS product DNA packaging tegument protein UL17 85102-85119 4:01:01 CCAACCCACTCACCCCCC

85270 85284 84016 86136 CDS product DNA packaging tegument protein UL17 85270-85284 4:01:01 GGGGGGGTCGGAGGG

85437 85451 84016 86136 CDS product DNA packaging tegument protein UL17 85437-85451 4:01:01 CCACCACCCGTGGCC

85501 85518 84016 86136 CDS product DNA packaging tegument protein UL17 85501-85518 4:01:01 CCTGTGTTTCCGCCCTCC

86198 86228 86156 87265 CDS product tegument protein UL16 86198-86228 6:03:01

CCGCGTCCTCTCCCAACCGCCAACTAACCCC

86359 86397 86156 87265 CDS product tegument protein UL16 86359-86397 7:04:01 CCGCCCCCGGCCAAGCTCACCCACACAGCCAAGTGCGCC

86518 86550 86156 87265 CDS product tegument protein UL16 86518-86550 6:03:01 CCCTTTCCCGTGGGCCCCCAGAGATTCCGCGCC

86581 86600 86156 87265 CDS product tegument protein UL16 86581-86600 4:01:01 CCGTCCGACGTACCGAACCC

86703 86721 86156 87265 CDS product tegument protein UL16 86703-86721 4:01:01 GGCTCCTGGCGTCTGGTGG

87087 87110 86156 87265 CDS product tegument protein UL16 87087-87110 4:01:01 GGTCGTGGGAATAGGGCCGCACGG

87136 87151 86156 87265 CDS product tegument protein UL16 87136-87151 4:01:01 GGATGGAAACTGGTGG

87973 88003 82687 88447 CDS product DNA packaging terminase subunit 1 87973-88003 5:02:01 GGCTACTTGGTGAAAGGCGTCTGAGGAGCGG

88098 88129 82687 88447 CDS product DNA packaging terminase subunit 1 88098-88129 5:02:01 CCTGTCGAACCTTGGGGTCCACGTAGGCCTCC

88123 88141 82687 88447 CDS product DNA packaging terminase subunit 1 88123-88141 4:01:01 GGCCTCCACGGAGGACGGG

88262 88293 82687 88447 CDS product DNA packaging terminase subunit 1 88262-88293 6:03:01 GGAACGACGGTCTGGTGGCGCTGGGGAGTAGG

88386 88402 82687 88447 CDS product DNA packaging terminase subunit 1 88386-88402 4:01:01 CCTCCCTCCGCAGCGCC

88422 88440 82687 88447 CDS product DNA packaging terminase subunit 1 88422-88440 4:01:01 CCCTGCCCAGCACCCTACC

88487 88508 88477 89433 CDS product tegument protein UL14 88487-88508 4:01:01 GGCACGGTCTAGGCGCCAAAGG

88866 88885 88477 89433 CDS product tegument protein UL14 88866-88885 6:03:01 GGAGGCGGCGGTGAGGTCGG

88982 89014 88477 89433 CDS product tegument protein UL14 88982-89014 5:02:01 CCCTCATTCTCCCCGCTCGACCTGTCCCGCCCC
89303 89335 88477 89433 CDS product tegument protein UL14 89303-89335 7:04:01 GGTCGGCCCGGCCGATCAGGAATGGCTGGGTGG
89585 89609 88899 90686 CDS product tegument serine/threonine protein kinase 89585-89609 5:02:01 GGAGGCGCCGGTAGCTACGGAGAGG
89845 89858 88899 90686 CDS product tegument serine/threonine protein kinase 89845-89858 4:01:01 CCCCCCCTCCGTCC
90416 90438 88899 90686 CDS product tegument serine/threonine protein kinase 90416-90438 5:02:01 CCCGCCCTCTTTCCCACCACCCC
90584 90598 88899 90686 CDS product tegument serine/threonine protein kinase 90584-90598 4:01:01 CCGTCCGCCTTTACC
90936 90954 90668 92365 CDS product deoxyribonuclease 90936-90954 5:02:01 CCGTCCGCTCCCCCATACC
91005 91043 90668 92365 CDS product deoxyribonuclease 91005-91043 6:03:01 GGTAGGAGAAATGGAGCTAGAGGGGATCGTGGAACGCGG
91077 91094 90668 92365 CDS product deoxyribonuclease 91077-91094 4:01:01 GGCTGGCTTTGCGGCAGG
91121 91140 90668 92365 CDS product deoxyribonuclease 91121-91140 4:01:01 CCAAAACCATGTCCCTGGCC
91401 91439 90668 92365 CDS product deoxyribonuclease 91401-91439 7:04:01 GGCGGCGACCCCGGACCTACTGGTGGATCCGGGATACGG
91519 91549 90668 92365 CDS product deoxyribonuclease 91519-91549 4:01:01 GGCGTCGTTGGGGCGTCCTTGGATATGCTGG
91953 91993 90668 92365 CDS product deoxyribonuclease 91953-91993 6:03:01 GGCCCGCTGGGACACAGGAGAGTTGGCTCTGTCGGTTCCGG
92054 92084 90668 92365 CDS product deoxyribonuclease 92054-92084 7:04:01 CCGGCCATTTTCCCGACCACCAACTCCGTCC

92235 92263 90668 92365 CDS product deoxyribonuclease 92235-92263 4:01:01 GGTGATTGTGGACCGCGAGGGTTGCTGGG
92436 92459 92317 92538 CDS product myristylated tegument protein 92436-92459 6:03:01 CCCCCACCCCCACCACTTAAACCC
93181 93205 92650 94002 CDS product envelope glycoprotein M 93181-93205 4:01:01 CCCACGGCAACCGTTACCGTTTGCC
93305 93324 92650 94002 CDS product envelope glycoprotein M 93305-93324 4:01:01 CCGCGCGTCCGGGTCCGGCC
93340 93357 92650 94002 CDS product envelope glycoprotein M 93340-93357 4:01:01 GGTAGGTTGGTCTTTAGG
93455 93471 92650 94002 CDS product envelope glycoprotein M 93455-93471 4:01:01 CCGCCCCCAGGACTGCC
94026 94043 93920 96583 CDS product DNA replication origin-binding helicase 94026-94043 4:01:01 GGGCGGCAAAGCGGAAGG
94108 94129 93920 96583 CDS product DNA replication origin-binding helicase 94108-94129 5:02:01 GGGGGCGACCTGGAGGAGTGGG
94189 94209 93920 96583 CDS product DNA replication origin-binding helicase 94189-94209 5:02:01 CCCCCGCCCAACAACCCGTCC
94650 94676 93920 96583 CDS product DNA replication origin-binding helicase 94650-94676 5:02:01 GGTGGATTTTTTGGCTTCGGCGCGTGG
95073 95087 93920 96583 CDS product DNA replication origin-binding helicase 95073-95087 4:01:01 GGTGGTAACGGTGGG
95787 95818 93920 96583 CDS product DNA replication origin-binding helicase 95787-95818 6:03:01 CCTCGGCGCCTGCCTGCGCCTCCCCGCGGCCC
96542 96562 93920 96583 CDS product DNA replication origin-binding helicase 96542-96562 4:01:01 GGCCACAGGTTTACGGGGCGG
96833 96868 96595 98850 CDS product helicase-primase subunit 96833-96868 5:02:01 GGCTGTGCCGGGGTGCTGGCCCTTGGCTCCCCTGGG

96994 97007 96595 98850 CDS product helicase-primase subunit 96994-97007 4:01:01 CCCTCCGACCGGCC
97017 97033 96595 98850 CDS product helicase-primase subunit 97017-97033 5:02:01 GGTGAAGGCGGTGGCGG
97638 97657 96595 98850 CDS product helicase-primase subunit 97638-97657 4:01:01 GGGCTGTGGCCGGCTCTAGG
97679 97719 96595 98850 CDS product helicase-primase subunit 97679-97719 7:04:01
GGCTCCATGGGAACCGGAAGATAGGTGGCCCGGTGAATCGG
97729 97751 96595 98850 CDS product helicase-primase subunit 97729-97751 4:01:01 GGGTAGATGGGGTGGCACACAGG
97769 97791 96595 98850 CDS product helicase-primase subunit 97769-97791 6:03:01 GGATGGTTGGGGGGCAGGGAGGG
97937 97950 96595 98850 CDS product helicase-primase subunit 97937-97950 4:01:01 CCACCACCCCACCC
97997 98030 96595 98850 CDS product helicase-primase subunit 97997-98030 5:02:01
CCCCGTGGCCTATAGGTCCATCATAGCCCTCGCC
98103 98122 96595 98850 CDS product helicase-primase subunit 98103-98122 4:01:01 GGCTTTTGGGGGGCAGCTGG
98370 98391 96595 98850 CDS product helicase-primase subunit 98370-98391 4:01:01 GGGGCCGGCAGGGCCGCAAAGG
99083 99108 98946 99857 CDS product tegument protein UL7 99083-99108 4:01:01 GGGACATGGGGTAGTGGGCTTTTTGG
99589 99607 98946 99857 CDS product tegument protein UL7 99589-99607 5:02:01 CCTCCGAGCCCTCCTGGCC
99668 99694 98946 99857 CDS product tegument protein UL7 99668-99694 5:02:01 GGTCGTAAAGGAGGGGGGCAAGCCTGG
100286 100305 99655 101916 CDS product capsid portal protein 100286-100305 4:01:01 CCACGTAGCCATCCTCTCCC
100328 100370 99655 101916 CDS product capsid portal protein 100328-100370 8:05:02
CCTCGTGCCCTAGTCCTCCTGCGCCAGCCTTGGCGCCACTCCC
100408 100422 99655 101916 CDS product capsid portal protein 100408-100422 4:01:01 CCGTCGGCCGCCGCC
100487 100518 99655 101916 CDS product capsid portal protein 100487-100518 5:02:01
CCAAAAGCCCGCTATTCCCGGCCCTCAGGTCC
100549 100571 99655 101916 CDS product capsid portal protein 100549-100571 4:01:01 CCCTCCAGCATTCCGTTAATGCC
100832 100872 99655 101916 CDS product capsid portal protein 100832-100872 7:04:01

CCACGGCCTTGGAGGCCCACCCCGGGCCCTGGGCCTCGTCC

101123 101182 99655 101916 CDS product capsid portal protein 101123-101182 11:08:02 CCATGATTGCCACGCTGGCCGTGCACCTGGCCAGCTCCACCACGGCCTCCAGCCCCTCCC

101284 101305 99655 101916 CDS product capsid portal protein 101284-101305 5:02:01 GGGTGGACGGTTGCGCCGGGGG

101364 101387 99655 101916 CDS product capsid portal protein 101364-101387 4:01:01 GGCAAAGCTGGCGGTGCCGTCGGG

101645 101659 99655 101916 CDS product capsid portal protein 101645-101659 4:01:01 CCGCCTCCGTTGACC

101783 101800 99655 101916 CDS product capsid portal protein 101783-101800 4:01:01 CCTCCTCACCCGGACTCC

101977 101991 101900 104545 CDS product helicase-primase helicase subunit 101977-101991 4:01:01 CCGCCTCGCCCCGCC

102323 102343 101900 104545 CDS product helicase-primase helicase subunit 102323-102343 4:01:01 CCTCGGCCTTCACAGCCCGCC

102425 102440 101900 104545 CDS product helicase-primase helicase subunit 102425-102440 4:01:01 CCTCGAGCCCGCCTCC

102750 102769 101900 104545 CDS product helicase-primase helicase subunit 102750-102769 4:01:01 GGATGGGAAGGTTCCCGTGG

103065 103080 101900 104545 CDS product helicase-primase helicase subunit 103065-103080 4:01:01 CCCCGCCAACCTTCCC

103603 103645 101900 104545 CDS product helicase-primase helicase subunit 103603-103645 8:05:02 GGGTTGGACGAGGGGAGGGTTCGGGAGGCGTACAGGCGCATGG

103750 103780 101900 104545 CDS product helicase-primase helicase subunit 103750-103780 5:02:01 GGATCTACGGGCGGGGCTACAGGCGACTGGG

104247 104287 101900 104545 CDS product helicase-primase helicase subunit 104247-104287 7:04:01 GGAGTCGGTGGACGACGTGGAGCTGGACATGGCCACCGCGG

104797 104817 104595 105269 CDS product nuclear protein UL4 104797-104817 4:01:01 GGCATTGGTGCTGGACTGCGG

104866 104878 104595 105269 CDS product nuclear protein UL4 104866-104878 4:01:01 CCCCCTGACCACC

104881 104900 104595 105269 CDS product nuclear protein UL4 104881-104900 5:02:01 GGACGGGTCGGTGCTGGTGG

105068 105091 104595 105269 CDS product nuclear protein UL4 105068-105091 5:02:01 CCTACCGTATGTTCCACCGCTGCC

105105 105131 104595 105269 CDS product nuclear protein UL4 105105-105131 5:02:01 CCGAGTCCGAGCCCTTATACGCCGACC

105239 105259 104595 105269 CDS product nuclear protein UL4 105239-105259 4:01:01 CCGTGTTTCCCCTGCCTCTCC

105461 105477 105399 105947 CDS product protein V57 105461-105477 4:01:01 GGTGGATGTGGGCTGGG

105508 105535 105399 105947 CDS product protein V57 105508-105535 5:02:01 CCTCGCCGCGGCTGCCGCGTCCGTCGCC

105667 105736 105399 105947 CDS product protein V57 105667-105736 11:08:02 CCATGGCCAGCGCCCCGAGCGTTCCAGAAGGCCTCTCCAAGCTAGCCAGCGGTCCGTCGCCACCCGCTCC

106299 106318 106009 106647 CDS product nuclear protein UL3 106299-106318 4:01:01 GGAGAACGAGGAGGTCTTGG

106475 106496 106009 106647 CDS product nuclear protein UL3 106475-106496 4:01:01 GGGATTCTACGGATAGGTCGGG

106507 106525 106009 106647 CDS product nuclear protein UL3 106507-106525 4:01:01 GGGGTTGGGGTGGAAGTGG

106608 106619 106009 106647 CDS product nuclear protein UL3 106608-106619 4:01:01 CCACCCCCACCC

106881 106905 106737 107678 CDS product uracil-DNA glycosylase 106881-106905 4:01:01 GGTGAGCAGGACTTCTGGGCGTGGG

106960 106985 106737 107678 CDS product uracil-DNA glycosylase 106960-106985 5:02:01 CCGCCCTGACTAGCCTGTGCCACCCC

107047 107077 106737 107678 CDS product uracil-DNA glycosylase 107047-107077 5:02:01 CCCCCTGTTCTGCCCACTTTTCCAGAAACCC

107448 107466 106737 107678 CDS product uracil-DNA glycosylase 107448-107466 4:01:01 GGGTGGGTGGACACGCTGG

107565 107589 106737 107678 CDS product uracil-DNA glycosylase 107565-107589 5:02:01 GGCCGGCCTGGTTCGCTGGTTGGGG

110080  110111  110076  111677  CDS product ubiquitin E3 ligase ICP0  110080-110111  5:02:01
GGTTCCTCCGGTATCTTTGGCAGTTGTGGGGG
110315  110336  110076  111677  CDS product ubiquitin E3 ligase ICP0  110315-110336  4:01:01
CCCCCCGAAGATCCCTGCACCC
110394  110417  110076  111677  CDS product ubiquitin E3 ligase ICP0  110394-110417  4:01:01
CCCATACCCCAGCGACCGGAGCCC
110529  110554  110076  111677  CDS product ubiquitin E3 ligase ICP0  110529-110554  5:02:01
GGTGCAACAGGTTGGTCCCTGGGGGG
110612  110629  110076  111677  CDS product ubiquitin E3 ligase ICP0  110612-110629  4:01:01 GGCAGGTGGGGATGAGGG
110630  110645  110076  111677  CDS product ubiquitin E3 ligase ICP0  110630-110645  4:01:01 CCCCGCCTCCTCTTCC
110667  110679  110076  111677  CDS product ubiquitin E3 ligase ICP0  110667-110679  4:01:01 GGCTGGTCGGTGG
110767  110795  110076  111677  CDS product ubiquitin E3 ligase ICP0  110767-110795  6:03:01
GGGTGCGGGGGTTGGTTCTTCTGGGCTGG
110870  110884  110076  111677  CDS product ubiquitin E3 ligase ICP0  110870-110884  4:01:01 GGTTGGTGGCCCGGG
110914  110932  110076  111677  CDS product ubiquitin E3 ligase ICP0  110914-110932  4:01:01 GGCGGGGTGCGGGTGCTGG
111304  111327  110076  111677  CDS product ubiquitin E3 ligase ICP0  111304-111327  4:01:01
GGGGTTGCAGGCGGGGAGCCCCGG
113516  113551  113498  117925  CDS product transcriptional regulator ICP4  113516-113551  7:04:01
CCCCAGTCTCCACCGTCCTCGTCGTCCGACACCACC
113563  113615  113498  117925  CDS product transcriptional regulator ICP4  113563-113615  10:07:02
GGGAGCGCGGCGGAGGGCCTGGGCCGCGCCGGGGCTAGGGCCGGGGTGGTCGG
113616  113640  113498  117925  CDS product transcriptional regulator ICP4  113616-113640  5:02:01
CCACCAGCTCCACGTCGCCGGCCCC

113747 113778 113498 117925 CDS product transcriptional regulator ICP4 113747-113778 6:03:01
GGGGCCCGGCGGCGGGGAGCCTCGGTGATGGG
113787 113808 113498 117925 CDS product transcriptional regulator ICP4 113787-113808 4:01:01
GGGGTCCGGCCTCGGCGTCGGG
113862 113895 113498 117925 CDS product transcriptional regulator ICP4 113862-113895 6:03:01
CCGAGGGCTCCACGGCCCCGGCTCCGCGCCGGCC
113892 113914 113498 117925 CDS product transcriptional regulator ICP4 113892-113914 4:01:01
GGCCGCAGGCGAGGTACACGGGG
113923 113956 113498 117925 CDS product transcriptional regulator ICP4 113923-113956 6:03:01
GGCCCCGAGGCCCCAGCGGTTGGCCGCGCGGTGG
114071 114089 113498 117925 CDS product transcriptional regulator ICP4 114071-114089 5:02:01
GGAGGCAGGGGGACGCGGG
114243 114264 113498 117925 CDS product transcriptional regulator ICP4 114243-114264 4:01:01
GGCTGAGGTGGCGCTCCCGGGG
114338 114355 113498 117925 CDS product transcriptional regulator ICP4 114338-114355 4:01:01
CCGTCCTGGGGCCAGTCC
114394 114418 113498 117925 CDS product transcriptional regulator ICP4 114394-114418 6:03:01
GGCGGAGCCCAGGCGGAGGCAGAGG
114433 114485 113498 117925 CDS product transcriptional regulator ICP4 114433-114485 9:06:02
GGTGAAGGCCAGGTCCCCGGTGGAGAGGAGCAGGACCCCCTGGGCGTTGAGGG
114486 114521 113498 117925 CDS product transcriptional regulator ICP4 114486-114521 6:03:01
CCGAGACGTCCGGGGCTCCGGTCCAGTTGCCGGCCC
114517 114560 113498 117925 CDS product transcriptional regulator ICP4 114517-114560 7:04:01

GGCCCAGGCGTGGGACCGCTTGGTGAGGACGCGGTTCCCCAGGG
114553 114586 113498 117925 CDS product transcriptional regulator ICP4 114553-114586 6:03:01
CCCCAGGGCCGCCAGCAGCGCCGAGAGTCCCCCC
114625 114640 113498 117925 CDS product transcriptional regulator ICP4 114625-114640 4:01:01 GGGGCGGGCGGCCGGG
114684 114700 113498 117925 CDS product transcriptional regulator ICP4 114684-114700 4:01:01
CCACCACCTTCACGTCC
114779 114798 113498 117925 CDS product transcriptional regulator ICP4 114779-114798 4:01:01
CCGTCCCTGGCCGGGGGTCC
114807 114842 113498 117925 CDS product transcriptional regulator ICP4 114807-114842 6:03:01
GGGCCGCGATGGTGGCTAGGGCCTGGGGATCGAAGG
114851 114880 113498 117925 CDS product transcriptional regulator ICP4 114851-114880 5:02:01
GGGCGCCAGGCCTCGGGGAACAGCGGGTGG
114904 114926 113498 117925 CDS product transcriptional regulator ICP4 114904-114926 4:01:01
GGGGGGACAGTAGGCTGCGCAGG
114990 115049 113498 117925 CDS product transcriptional regulator ICP4 114990-115049 11:08:02
GGTCGGGGCCCTCGGCGGGCATGGGTCCCAGGGCCCGGGGAGCCTGGTGGCCCTGGGTGG
115016 115055 113498 117925 CDS product transcriptional regulator ICP4 115016-115055 6:03:01
CCCAGGGCCCGGGGAGCCTGGTGGCCCTGGGTGGCCACCC
115063 115075 113498 117925 CDS product transcriptional regulator ICP4 115063-115075 4:01:01 GGGGGCAGGAGGG
115110 115133 113498 117925 CDS product transcriptional regulator ICP4 115110-115133 5:02:01
GGGCGGACGAGGAGAAGGCCGGGG
115128 115150 113498 117925 CDS product transcriptional regulator ICP4 115128-115150 5:02:01
CCGGGGCTCCAGCCTGGGCCGCC

115143 115166 113498 117925 CDS product transcriptional regulator ICP4 115143-115166 4:01:01
GGGCCGCCGGCTCTAGGGGCTCGG
115171 115200 113498 117925 CDS product transcriptional regulator ICP4 115171-115200 4:01:01
CCGCTTGCCGCTCTTGCCCCGGGGGCGCCC
115251 115274 113498 117925 CDS product transcriptional regulator ICP4 115251-115274 6:03:01
GGCTGAGGTGGGCCGGGGAGGCGG
115311 115358 113498 117925 CDS product transcriptional regulator ICP4 115311-115358 9:06:02
GGGAGCGGGCGCTGGTCTGGGTGGCCCGGGAGCAGGTGGCTGTCGCGG
115377 115397 113498 117925 CDS product transcriptional regulator ICP4 115377-115397 4:01:01
GGGAGTGCTGGGGGGACTGGG
115433 115459 113498 117925 CDS product transcriptional regulator ICP4 115433-115459 6:03:01
GGGACGGTGGGCCACAGGGGCGGAAGG
115462 115482 113498 117925 CDS product transcriptional regulator ICP4 115462-115482 4:01:01
CCCCTCCGCGGCCGAGGAGCC
115471 115498 113498 117925 CDS product transcriptional regulator ICP4 115471-115498 5:02:01
GGCCGAGGAGCCGGAGAAGGGCTGCTGG
115513 115535 113498 117925 CDS product transcriptional regulator ICP4 115513-115535 5:02:01
GGGGGAAGACGAGGAGGGCTGGG
115544 115565 113498 117925 CDS product transcriptional regulator ICP4 115544-115565 4:01:01
GGTGGGGCTAGCAGGGACACGG
115566 115588 113498 117925 CDS product transcriptional regulator ICP4 115566-115588 6:03:01
CCTCCCCCAACATCCCCCCGACC
115609 115655 113498 117925 CDS product transcriptional regulator ICP4 115609-115655 8:05:02

GGCCTGGGTGACGGTCCAGGCCGAGGCCCGGGCCCGGGCCCCCTCGG
115624 115651 113498 117925 CDS product transcriptional regulator ICP4 115624-115651 6:03:01
CCAGGCCGAGGCCCGGGCCCGGGCCCCC
115729 115750 113498 117925 CDS product transcriptional regulator ICP4 115729-115750 5:02:01
GGCGCCCAGGCGGTGGGTAGGG
115752 115790 113498 117925 CDS product transcriptional regulator ICP4 115752-115790 6:03:01
CCGAGTCCCCGAGGAGCCTGGCCCGCTCGACCAGGTCCC
115812 115831 113498 117925 CDS product transcriptional regulator ICP4 115812-115831 5:02:01
CCGCCGTCTCCAGCCCGGCC
115852 115873 113498 117925 CDS product transcriptional regulator ICP4 115852-115873 5:02:01
GGGAGGGCAGGCGGGAGAGGGG
115917 115931 113498 117925 CDS product transcriptional regulator ICP4 115917-115931 4:01:01 GGAGGCGCAGGAGGG
115990 116039 113498 117925 CDS product transcriptional regulator ICP4 115990-116039 10:07:02
GGCGGACGGCCTGGGTGCCCTGGTACCCGGAGCGGCAGCGGCCGCGGCGG
116088 116118 113498 117925 CDS product transcriptional regulator ICP4 116088-116118 6:03:01
GGACGCGGACCGAGGCCGCCGGGGGCTCGGG
116129 116179 113498 117925 CDS product transcriptional regulator ICP4 116129-116179 9:06:02
CCGGCCGCTGCCGCGGCGGCCTGGGCCGGGTAGCCGGCCGAGACCCCGGCC
116143 116165 113498 117925 CDS product transcriptional regulator ICP4 116143-116165 5:02:01
GGCGGCCTGGGCCGGGTAGCCGG
116196 116209 113498 117925 CDS product transcriptional regulator ICP4 116196-116209 4:01:01 CCGGCCTCCCCGCC
116247 116260 113498 117925 CDS product transcriptional regulator ICP4 116247-116260 4:01:01 GGAGGCTCTGGAGG
116280 116312 113498 117925 CDS product transcriptional regulator ICP4 116280-116312 6:03:01

GGTCGTAGCGGCGGCTCATGGCCACCGAGGCGG

116342 116361 113498 117925 CDS product transcriptional regulator ICP4 116342-116361 4:01:01 CCGGCCGCCATGGCATCCCC

116367 116381 113498 117925 CDS product transcriptional regulator ICP4 116367-116381 4:01:01 GGGGCAGGGGGTTGG

116634 116667 113498 117925 CDS product transcriptional regulator ICP4 116634-116667 6:03:01 CCCACAGCCCGTCCCGCGAGTCCCCGGTCCCCCC

116697 116718 113498 117925 CDS product transcriptional regulator ICP4 116697-116718 4:01:01 CCGACCCGGGCCAGGGGTCCCC

116704 116726 113498 117925 CDS product transcriptional regulator ICP4 116704-116726 4:01:01 GGGCCAGGGGTCCCCGGACGGGG

116769 116782 113498 117925 CDS product transcriptional regulator ICP4 116769-116782 4:01:01 CCACCGGGGCCGCC

116791 116804 113498 117925 CDS product transcriptional regulator ICP4 116791-116804 4:01:01 GGAGGCGGCCTGGG

116817 116851 113498 117925 CDS product transcriptional regulator ICP4 116817-116851 6:03:01 CCAGCAAGTCCCAAGAGGCCGTCCGCGGGGCCTCC

116858 116891 113498 117925 CDS product transcriptional regulator ICP4 116858-116891 6:03:01 GGTGCCTGGGTCTGGGGTATGGGTCTGGGGTTGG

116957 116973 113498 117925 CDS product transcriptional regulator ICP4 116957-116973 4:01:01 GGGCTCCGGCCCGGCGG

117068 117079 113498 117925 CDS product transcriptional regulator ICP4 117068-117079 4:01:01 GGAGGGAGGAGG

117089 117145 113498 117925 CDS product transcriptional regulator ICP4 117089-117145 10:07:02 GGGCGGCTGGGCGCGGGCGCGGGCGCGGGCGCACCCGGAGAAGCGGCGCCGGAGCGG

117155 117178 113498 117925 CDS product transcriptional regulator ICP4 117155-117178 5:02:01 GGGGTGGCGGCCCCGGCCGCCGGG

117190 117240 113498 117925 CDS product transcriptional regulator ICP4 117190-117240 9:06:02
GGAGGACGGCCCGGGAGAGCCGGCGGCCGACGGGGTCTTCGCGGCGTCCGG
117264 117276 113498 117925 CDS product transcriptional regulator ICP4 117264-117276 4:01:01 CCTCCTCGCCGCC
117324 117355 113498 117925 CDS product transcriptional regulator ICP4 117324-117355 6:03:01
CCCCGGCACCATCCGCCTGGTCGTCCGCGTCC
117397 117426 113498 117925 CDS product transcriptional regulator ICP4 117397-117426 6:03:01
CCGGACCCGGGGCGCCGGGGACCCTCCACC
117428 117479 113498 117925 CDS product transcriptional regulator ICP4 117428-117479 9:06:02
GGGGAGGCCGAGGGTGGAAACTCGGGCTGCGGGGACCCGGGGCAGGTCTCGG
117498 117522 113498 117925 CDS product transcriptional regulator ICP4 117498-117522 5:02:01
CCGGGTCGGCCGCGTCCCCGCCGCC
117523 117547 113498 117925 CDS product transcriptional regulator ICP4 117523-117547 4:01:01
GGATGATGAGGAGCCCGTGGCCCGG
117536 117565 113498 117925 CDS product transcriptional regulator ICP4 117536-117565 5:02:01
CCCGTGGCCCGGCGACCGTTCCCCGGGGCC
117560 117577 113498 117925 CDS product transcriptional regulator ICP4 117560-117577 4:01:01
GGGGCCACGGAGGAGTGG
117605 117672 113498 117925 CDS product transcriptional regulator ICP4 117605-117672 12:09:03
GGAGCCGGGCTGGGTGCCGGGGACGCCGGCTGGGCGGCCGCCGGGGTAGGAGGACCTCCGGCAGCGGG
117676 117693 113498 117925 CDS product transcriptional regulator ICP4 117676-117693 4:01:01
CCGCTTCGCCTTCCCTCC
117696 117724 113498 117925 CDS product transcriptional regulator ICP4 117696-117724 6:03:01
GGGGCTCGGGAGCCGGGGACGGCGGGGGG

117759  117783  113498  117925  CDS product transcriptional regulator ICP4  117759-117783  4:01:01
CCACCCCGAACATGCCCTGGCTGCC
117792  117812  113498  117925  CDS product transcriptional regulator ICP4  117792-117812  5:02:01
GGTCGGGCGCGGCGGGCTGGG
117826  117853  113498  117925  CDS product transcriptional regulator ICP4  117826-117853  5:02:01
GGCCGCGCTGGCTGCGCGGATGAGGGGG
120696  120728  120623  121498  CDS product regulatory protein ICP22  120696-120728  7:04:01
CCCCATCATCCCGTCCCTGACCCCCTCCCCCCC
120738  120757  120623  121498  CDS product regulatory protein ICP22  120738-120757  4:01:01 CCCGTCCCCACGCTCCAGCC
120788  120821  120623  121498  CDS product regulatory protein ICP22  120788-120821  6:03:01
GGCTCCCAGGCGGCTCGGACCATCCGGAATACGG
120826  120870  120623  121498  CDS product regulatory protein ICP22  120826-120870  7:04:01
CCGCTCTCCCCACGGGCCCTGCGCCCGTACCTGGCCCGGGGGCCC
120858  120874  120623  121498  CDS product regulatory protein ICP22  120858-120874  4:01:01 GGCCCGGGGGCCCGGGG
120950  120972  120623  121498  CDS product regulatory protein ICP22  120950-120972  5:02:01
CCACCTCCTCGATCCACGTGACC
121149  121180  120623  121498  CDS product regulatory protein ICP22  121149-121180  6:03:01
CCTGGATCCACCGAGCGACCCCCCGAATACCC
121227  121252  120623  121498  CDS product regulatory protein ICP22  121227-121252  5:02:01
GGAGGAGGAAGACGAGGCCAGCGGGG
121317  121330  120623  121498  CDS product regulatory protein ICP22  121317-121330  4:01:01 GGACGTGGGGGAGG
121407  121434  120623  121498  CDS product regulatory protein ICP22  121407-121434  5:02:01
CCACCCCACAACCCGCGCCAGCGCTGCC

123295 123316 123290 124099 CDS product virion protein V67 123295-123316 4:01:01 GGCCACGGGGGCGTCTTCGGGG
123339 123388 123290 124099 CDS product virion protein V67 123339-123388 8:05:02 GGCTGCGCGGCTGCCTGGACCCTCTGGAGTGGGGGTCCGGGGAGCTCTGG
123532 123554 123290 124099 CDS product virion protein V67 123532-123554 5:02:01 GGCTATGGCGGGGGGAACCTTGG
123595 123611 123290 124099 CDS product virion protein V67 123595-123611 4:01:01 GGCATCGGCGGCGTCGG
123633 123652 123290 124099 CDS product virion protein V67 123633-123652 4:01:01 CCGCGGGTTCCTCCTCGCCC
123668 123695 123290 124099 CDS product virion protein V67 123668-123695 6:03:01 GGGTAGAGGAACGGCAGGCGGACGAAGG
123736 123752 123290 124099 CDS product virion protein V67 123736-123752 4:01:01 GGGCAGCGGCAGGGCGG
123765 123790 123290 124099 CDS product virion protein V67 123765-123790 7:04:01 CCTCTCCGGCCACCACCCCCTCGACC
123939 123963 123290 124099 CDS product virion protein V67 123939-123963 6:03:01 CCCCCTTGGGCGCCCCCTCGCCGCC
124008 124033 123290 124099 CDS product virion protein V67 124008-124033 6:03:01 GGTGGGGCGGGTTGGCATTGCGGCGG
124060 124076 123290 124099 CDS product virion protein V67 124060-124076 5:02:01 GGTGCCGGCGGTGGCGG
124340 124406 124273 125163 CDS product virion protein US2 124340-124406 13:10:03 GGCGGGCGGCCGCGGCCCGCGGGGCCCCGGGGTGGGGCGTCTCGGTGGCGGATCGAGGGGGCGTGGG
124437 124469 124273 125163 CDS product virion protein US2 124437-124469 8:05:02 CCCCCCCTCCGACGGCCGCCGCCGCTGCCGCCC
125374 125396 125299 126450 CDS product serine/threonine protein kinase US3 125374-125396 6:03:01 CCACACCCCCGCTGCCACCTACC
126675 126708 126572 127807 CDS product envelope glycoprotein G 126675-126708 7:04:01 CCACCCACCAAACACCCAGCCCGAACGTCCACCC
126907 126921 126572 127807 CDS product envelope glycoprotein G 126907-126921 4:01:01 CCCTCCCCCGAGACC
127172 127191 126572 127807 CDS product envelope glycoprotein G 127172-127191 4:01:01 CCAGCCCGCACCCGCGTGCC
127357 127370 126572 127807 CDS product envelope glycoprotein G 127357-127370 4:01:01 CCGGCCACCGGACC

127510 127529 126572 127807 CDS product envelope glycoprotein G 127510-127529 4:01:01 CCTCCCGAAACTCCATCGCC
128146 128166 127991 130483 CDS product envelope glycoprotein J 128146-128166 5:02:01 CCCACCACGATCCCACCTACC
128179 128207 127991 130483 CDS product envelope glycoprotein J 128179-128207 5:02:01
CCCCCCACATCAACCCACACATCCTCCCC
128282 128307 127991 130483 CDS product envelope glycoprotein J 128282-128307 5:02:01
CCAGCACCACCTCTGTTCCAACATCC
128321 128340 127991 130483 CDS product envelope glycoprotein J 128321-128340 4:01:01 CCACCACAACAACCCCAACC
128504 128529 127991 130483 CDS product envelope glycoprotein J 128504-128529 5:02:01
CCACCACTGCTCCAACAACCGCTTCC
128900 128943 127991 130483 CDS product envelope glycoprotein J 128900-128943 8:05:02
CCACGTCCACCCCCTCAGCTTCCACTACCACATCTGCCACTCCC
129302 129320 127991 130483 CDS product envelope glycoprotein J 129302-129320 4:01:01 CCGACTCTTCCACCGTGCC
129452 129479 127991 130483 CDS product envelope glycoprotein J 129452-129479 6:03:01
CCACCCCATCACCTCCCCCGCGTACCCC
129577 129627 127991 130483 CDS product envelope glycoprotein J 129577-129627 9:06:02
GGGCGGCACAAAGGTCGCGCAGGGGGTCGTCGGGATGGCCATCAGGGGGGG
129736 129756 127991 130483 CDS product envelope glycoprotein J 129736-129756 6:03:01 CCGCCCCCACCAGACCGACCC
130058 130078 127991 130483 CDS product envelope glycoprotein J 130058-130078 4:01:01 CCACAATACCGTGTCCAGGCC
130680 130708 130574 131782 CDS product envelope glycoprotein D 130680-130708 5:02:01
GGTTCGAGGACGCCAGGATCGGCCAAAGG
131404 131426 130574 131782 CDS product envelope glycoprotein D 131404-131426 5:02:01 CCCGTGCCTCCGGATAACCACCC
132283 132305 131887 133164 CDS product envelope glycoprotein I 132283-132305 4:01:01 CCAAACCACAGCCCACAGATTCC
132397 132419 131887 133164 CDS product envelope glycoprotein I 132397-132419 5:02:01 CCGTCCCCGATCCAATGCCCACC

| | | | | | | | |
|---|---|---|---|---|---|---|---|
| 132538 | 132551 | 131887 | 133164 | CDS product envelope glycoprotein I | 132538-132551 | 4:01:01 | CCACCCAAACCCCC |
| 132605 | 132621 | 131887 | 133164 | CDS product envelope glycoprotein I | 132605-132621 | 4:01:01 | GGTTGTTGGTCGGGAGG |
| 132745 | 132766 | 131887 | 133164 | CDS product envelope glycoprotein I | 132745-132766 | 4:01:01 | CCCCATCGCCTGAACCGAGGCC |
| 132966 | 133001 | 131887 | 133164 | CDS product envelope glycoprotein I | 132966-133001 | 7:04:01 | GGACAGGGGGGCACATCGGCGGCCGAGCGGAGACGG |
| 133065 | 133081 | 131887 | 133164 | CDS product envelope glycoprotein I | 133065-133081 | 5:02:01 | CCTCCAACCCCCAAACC |
| 133776 | 133812 | 133399 | 135051 | CDS product envelope glycoprotein E | 133776-133812 | 6:03:01 | CCAATCCTTGCCGAGACCCGCAGCGTCCTACAGATCC |
| 133908 | 133927 | 133399 | 135051 | CDS product envelope glycoprotein E | 133908-133927 | 4:01:01 | CCTCCCAAACAACCGCAACC |
| 133945 | 133975 | 133399 | 135051 | CDS product envelope glycoprotein E | 133945-133975 | 6:03:01 | CCCCACCACCCGTAACCGTTCCTCAGGTTCC |
| 134174 | 134200 | 133399 | 135051 | CDS product envelope glycoprotein E | 134174-134200 | 6:03:01 | CCCCACCGCCATGGCCTGCCTGCACCC |
| 134430 | 134447 | 133399 | 135051 | CDS product envelope glycoprotein E | 134430-134447 | 4:01:01 | GGACATCCGGAGGCGTGG |
| 134852 | 134892 | 133399 | 135051 | CDS product envelope glycoprotein E | 134852-134892 | 9:06:02 | CCCACCACCTCCAAAACCAGCTCCACAGCTCCCACCATACC |
| 134922 | 134945 | 133399 | 135051 | CDS product envelope glycoprotein E | 134922-134945 | 5:02:01 | GGCAGGTCCGGATTCAAGGTTTGG |
| 135179 | 135199 | 135048 | 135440 | CDS product membrane protein US8A | 135179-135199 | 4:01:01 | CCCAGATCCGGCTCAACCTCC |
| 135777 | 135790 | 135776 | 136435 | CDS product membrane protein US9 | 135777-135790 | 4:01:01 | GGAGAAGGCGGAGG |
| 135935 | 135948 | 135776 | 136435 | CDS product membrane protein US9 | 135935-135948 | 4:01:01 | CCGCTACCTCCGCC |
| 136059 | 136076 | 135776 | 136435 | CDS product membrane protein US9 | 136059-136076 | 4:01:01 | CCCGTCTCCACCATCACC |
| 136317 | 136361 | 135776 | 136435 | CDS product membrane protein US9 | 136317-136361 | 8:05:02 | GGCCCGCAGGCACAGGAGGCGGCGCGTGGCCCTTACGGTTGCAGG |

137007 137023 136983 137795 CDS product virion protein V67 137007-137023 5:02:01 CCGCCACCGCCGGCACC
137050 137075 136983 137795 CDS product virion protein V67 137050-137075 6:03:01 CCGCCGCAATGCCAACCCGCCCCACC
137120 137144 136983 137795 CDS product virion protein V67 137120-137144 6:03:01 GGCGGCGAGGGGGCGCCCAAGGGGG
137293 137318 136983 137795 CDS product virion protein V67 137293-137318 7:04:01 GGTCGAGGGGGTGGTGGCCGGAGAGG
137331 137347 136983 137795 CDS product virion protein V67 137331-137347 4:01:01 CCGCCCTGCCGCTGCCC
137388 137415 136983 137795 CDS product virion protein V67 137388-137415 6:03:01 CCTTCGTCCGCCTGCCGTTCCTCTACCC
137431 137450 136983 137795 CDS product virion protein V67 137431-137450 4:01:01 GGGCGAGGAGGAACCCGCGG
137472 137488 136983 137795 CDS product virion protein V67 137472-137488 4:01:01 CCGACGCCGCCGATGCC
137529 137551 136983 137795 CDS product virion protein V67 137529-137551 5:02:01 CCAAGGTTCCCCCCGCCATAGCC
137695 137744 136983 137795 CDS product virion protein V67 137695-137744 8:05:02
CCAGAGCTCCCCGGACCCCCACTCCAGAGGGTCCAGGCAGCCGCGCAGCC
137767 137788 136983 137795 CDS product virion protein V67 137767-137788 4:01:01 CCCCGAAGACGCCCCCGTGGCC
138345 138365 138307 139011 CDS product virion protein 138345-138365 5:02:01 GGCGGTGCGGCGGGCCGCGGG
138376 138402 138307 139011 CDS product virion protein 138376-138402 5:02:01 GGGGGCTTGCGGCCGCGGGTGCGCAGG
138414 138429 138307 139011 CDS product virion protein 138414-138429 5:02:01 GGCGGCGTGGCGGGGG
138641 138663 138307 139011 CDS product virion protein 138641-138663 4:01:01 CCACAGGCGCCGCGCCGAGATCC
138707 138727 138307 139011 CDS product virion protein 138707-138727 4:01:01 GGAAGACGGGCATGGGGCTGG
138772 138796 138307 139011 CDS product virion protein 138772-138796 5:02:01 GGCCAGGCCTCCGCGGGCGGCCCGG
138815 138828 138307 139011 CDS product virion protein 138815-138828 4:01:01 CCGCTGCCTCCGCC
138918 138959 138307 139011 CDS product virion protein 138918-138959 8:05:02
CCCGTCGGCGCCCGCCCCCGTCCCCGCTCCGGACTCCTCGCC
139649 139676 139587 140462 CDS product regulatory protein ICP22 139649-139676 5:02:01
GGCAGCGCTGGCGCGGGTTGTGGGGTGG

139753 139766 139587 140462 CDS product regulatory protein ICP22 139753-139766 4:01:01 CCTCCCCCACGTCC
139831 139856 139587 140462 CDS product regulatory protein ICP22 139831-139856 5:02:01
CCCCGCTGGCCTCGTCTTCCTCCTCC
139903 139934 139587 140462 CDS product regulatory protein ICP22 139903-139934 6:03:01
GGGTATTCGGGGGGTCGCTCGGTGGATCCAGG
140111 140133 139587 140462 CDS product regulatory protein ICP22 140111-140133 5:02:01
GGTCACGTGGATCGAGGAGGTGG
140209 140225 139587 140462 CDS product regulatory protein ICP22 140209-140225 4:01:01 CCCCGGGCCCCCGGGCC
140213 140257 139587 140462 CDS product regulatory protein ICP22 140213-140257 7:04:01
GGGCCCCCGGGCCAGGTACGGGCGCAGGGCCCGTGGGGAGAGCGG
140262 140295 139587 140462 CDS product regulatory protein ICP22 140262-140295 6:03:01
CCGTATTCCGGATGGTCCGAGCCGCCTGGGAGCC
140326 140345 139587 140462 CDS product regulatory protein ICP22 140326-140345 4:01:01 GGCTGGAGCGTGGGGACGGG
140355 140387 139587 140462 CDS product regulatory protein ICP22 140355-140387 7:04:01
GGGGGGGAGGGGGTCAGGGACGGGATGATGGGG
143230 143257 143160 147587 CDS product transcriptional regulator ICP4 143230-143257 5:02:01
CCCCCTCATCCGCGCAGCCAGCGCGGCC
143271 143291 143160 147587 CDS product transcriptional regulator ICP4 143271-143291 5:02:01
CCCAGCCCGCCGCGCCCGACC
143300 143324 143160 147587 CDS product transcriptional regulator ICP4 143300-143324 4:01:01
GGCAGCCAGGGCATGTTCGGGGTGG
143359 143387 143160 147587 CDS product transcriptional regulator ICP4 143359-143387 6:03:01
CCCCCCGCCGTCCCCGGCTCCCGAGCCCC

143390 143407 143160 147587 CDS product transcriptional regulator ICP4 143390-143407 4:01:01
GGAGGGAAGGCGAAGCGG
143411 143478 143160 147587 CDS product transcriptional regulator ICP4 143411-143478 12:09:03
CCCGCTGCCGGAGGTCCTCCTACCCCGGCGGCCGCCCAGCCGGCGTCCCCGGCACCCAGCCCGGCTCC
143506 143523 143160 147587 CDS product transcriptional regulator ICP4 143506-143523 4:01:01
CCACTCCTCCGTGGCCCC
143518 143547 143160 147587 CDS product transcriptional regulator ICP4 143518-143547 5:02:01
GGCCCCGGGGAACGGTCGCCGGGCCACGGG
143536 143560 143160 147587 CDS product transcriptional regulator ICP4 143536-143560 4:01:01
CCGGGCCACGGGCTCCTCATCATCC
143561 143585 143160 147587 CDS product transcriptional regulator ICP4 143561-143585 5:02:01
GGCGGCGGGGACGCGGCCGACCCGG
143604 143655 143160 147587 CDS product transcriptional regulator ICP4 143604-143655 9:06:02
CCGAGACCTGCCCCGGGTCCCCGCAGCCCGAGTTTCCACCCTCGGCCTCCCC
143657 143686 143160 147587 CDS product transcriptional regulator ICP4 143657-143686 6:03:01
GGTGGAGGGTCCCCGGCGCCCCGGGTCCGG
143728 143759 143160 147587 CDS product transcriptional regulator ICP4 143728-143759 6:03:01
GGACGCGGACGACCAGGCGGATGGTGCCGGGG
143807 143819 143160 147587 CDS product transcriptional regulator ICP4 143807-143819 4:01:01 GGCGGCGAGGAGG
143843 143893 143160 147587 CDS product transcriptional regulator ICP4 143843-143893 9:06:02
CCGGACGCCGCGAAGACCCCGTCGGCCGCCGGCTCTCCCGGGCCGTCCTCC
143905 143928 143160 147587 CDS product transcriptional regulator ICP4 143905-143928 5:02:01
CCCGGCGGCCGGGGCCGCCACCCC

143938 143994 143160 147587 CDS product transcriptional regulator ICP4 143938-143994 10:07:02
CCGCTCCGGCGCCGCTTCTCCGGGTGCGCCCGCGCCCGCGCCCGCGCCCAGCCGCCC
144004 144015 143160 147587 CDS product transcriptional regulator ICP4 144004-144015 4:01:01 CCTCCTCCCTCC
144110 144126 143160 147587 CDS product transcriptional regulator ICP4 144110-144126 4:01:01
CCGCCGGGCCGGAGCCC
144192 144225 143160 147587 CDS product transcriptional regulator ICP4 144192-144225 6:03:01
CCAACCCCAGACCCATACCCCAGACCCAGGCACC
144232 144266 143160 147587 CDS product transcriptional regulator ICP4 144232-144266 6:03:01
GGAGGCCCCGCGGACGGCCTCTTGGGACTTGCTGG
144279 144292 143160 147587 CDS product transcriptional regulator ICP4 144279-144292 4:01:01 CCCAGGCCGCCTCC
144301 144314 143160 147587 CDS product transcriptional regulator ICP4 144301-144314 4:01:01 GGCGGCCCCGGTGG
144357 144379 143160 147587 CDS product transcriptional regulator ICP4 144357-144379 4:01:01
CCCCGTCCGGGGACCCCTGGCCC
144365 144386 143160 147587 CDS product transcriptional regulator ICP4 144365-144386 4:01:01
GGGGACCCCTGGCCCGGGTCGG
144416 144449 143160 147587 CDS product transcriptional regulator ICP4 144416-144449 6:03:01
GGGGGGACCGGGGACTCGCGGGACGGGCTGTGGG
144702 144716 143160 147587 CDS product transcriptional regulator ICP4 144702-144716 4:01:01 CCAACCCCCTGCCCC
144722 144741 143160 147587 CDS product transcriptional regulator ICP4 144722-144741 4:01:01
GGGGATGCCATGGCGGCCGG
144771 144803 143160 147587 CDS product transcriptional regulator ICP4 144771-144803 6:03:01
CCGCCTCGGTGGCCATGAGCCGCCGCTACGACC
144823 144836 143160 147587 CDS product transcriptional regulator ICP4 144823-144836 4:01:01 CCTCCAGAGCCTCC

144874 144887 143160 147587 CDS product transcriptional regulator ICP4 144874-144887 4:01:01 GGCGGGGAGGCCGG
144904 144954 143160 147587 CDS product transcriptional regulator ICP4 144904-144954 9:06:02
GGCCGGGGTCTCGGCCGGCTACCCGGCCCAGGCCGCCGCGGCAGCGGCCGG
144918 144940 143160 147587 CDS product transcriptional regulator ICP4 144918-144940 5:02:01
CCGGCTACCCGGCCCAGGCCGCC
144965 144995 143160 147587 CDS product transcriptional regulator ICP4 144965-144995 6:03:01
CCCGAGCCCCCGGCGGCCTCGGTCCGCGTCC
145044 145093 143160 147587 CDS product transcriptional regulator ICP4 145044-145093 10:07:02
CCGCCGCGGCCGCTGCCGCTCCGGGTACCAGGGCACCCAGGCCGTCCGCC
145152 145166 143160 147587 CDS product transcriptional regulator ICP4 145152-145166 4:01:01 CCCTCCTGCGCCTCC
145210 145231 143160 147587 CDS product transcriptional regulator ICP4 145210-145231 5:02:01
CCCCTCTCCCGCCTGCCCTCCC
145252 145271 143160 147587 CDS product transcriptional regulator ICP4 145252-145271 5:02:01
GGCCGGGCTGGAGACGGCGG
145293 145331 143160 147587 CDS product transcriptional regulator ICP4 145293-145331 6:03:01
GGGACCTGGTCGAGCGGGCCAGGCTCCTCGGGGACTCGG
145333 145354 143160 147587 CDS product transcriptional regulator ICP4 145333-145354 5:02:01
CCCTACCCACCGCCTGGGCGCC
145428 145474 143160 147587 CDS product transcriptional regulator ICP4 145428-145474 8:05:02
CCGAGGGGGCCCGGGCCCGGGCCTCGGCCTGGACCGTCACCCAGGCC
145432 145459 143160 147587 CDS product transcriptional regulator ICP4 145432-145459 6:03:01
GGGGGCCCGGGCCCGGGCCTCGGCCTGG
145495 145517 143160 147587 CDS product transcriptional regulator ICP4 145495-145517 6:03:01

GGTCGGGGGGATGTTGGGGGAGG

| | | | | | | |
|---|---|---|---|---|---|---|
| 145518 | 145539 | 143160 | 147587 | CDS product transcriptional regulator ICP4 | 145518-145539 | 4:01:01 |
| CCGTGTCCCTGCTAGCCCCACC | | | | | | |
| 145548 | 145570 | 143160 | 147587 | CDS product transcriptional regulator ICP4 | 145548-145570 | 5:02:01 |
| CCCAGCCCTCCTCGTCTTCCCCC | | | | | | |
| 145585 | 145612 | 143160 | 147587 | CDS product transcriptional regulator ICP4 | 145585-145612 | 5:02:01 |
| CCAGCAGCCCTTCTCCGGCTCCTCGGCC | | | | | | |
| 145601 | 145621 | 143160 | 147587 | CDS product transcriptional regulator ICP4 | 145601-145621 | 4:01:01 |
| GGCTCCTCGGCCGCGGAGGGG | | | | | | |
| 145624 | 145650 | 143160 | 147587 | CDS product transcriptional regulator ICP4 | 145624-145650 | 6:03:01 |
| CCTTCCGCCCCTGTGGCCCACCGTCCC | | | | | | |
| 145686 | 145706 | 143160 | 147587 | CDS product transcriptional regulator ICP4 | 145686-145706 | 4:01:01 |
| CCCAGTCCCCCCAGCACTCCC | | | | | | |
| 145725 | 145772 | 143160 | 147587 | CDS product transcriptional regulator ICP4 | 145725-145772 | 9:06:02 |
| CCGCGACAGCCACCTGCTCCCGGGCCACCCAGACCAGCGCCCGCTCCC | | | | | | |
| 145809 | 145832 | 143160 | 147587 | CDS product transcriptional regulator ICP4 | 145809-145832 | 6:03:01 |
| CCGCCTCCCCGGCCCACCTCAGCC | | | | | | |
| 145883 | 145912 | 143160 | 147587 | CDS product transcriptional regulator ICP4 | 145883-145912 | 4:01:01 |
| GGGCGCCCCCGGGGCAAGAGCGGCAAGCGG | | | | | | |
| 145917 | 145940 | 143160 | 147587 | CDS product transcriptional regulator ICP4 | 145917-145940 | 4:01:01 |
| CCGAGCCCCTAGAGCCGGCGGCCC | | | | | | |
| 145933 | 145955 | 143160 | 147587 | CDS product transcriptional regulator ICP4 | 145933-145955 | 5:02:01 |
| GGCGGCCCAGGCTGGAGCCCCGG | | | | | | |

145950 145973 143160 147587 CDS product transcriptional regulator ICP4 145950-145973 5:02:01 CCCCGGCCTTCTCCTCGTCCGCCC

146008 146020 143160 147587 CDS product transcriptional regulator ICP4 146008-146020 4:01:01 CCCTCCTGCCCCC

146028 146067 143160 147587 CDS product transcriptional regulator ICP4 146028-146067 6:03:01 GGGTGGCCACCCAGGGCCACCAGGCTCCCCGGGCCCTGGG

146034 146093 143160 147587 CDS product transcriptional regulator ICP4 146034-146093 11:08:02 CCACCCAGGGCCACCAGGCTCCCCGGGCCCTGGGACCCATGCCCGCCGAGGGCCCCGACC

146157 146179 143160 147587 CDS product transcriptional regulator ICP4 146157-146179 4:01:01 CCTGCGCAGCCTACTGTCCCCCC

146203 146232 143160 147587 CDS product transcriptional regulator ICP4 146203-146232 5:02:01 CCACCCGCTGTTCCCCGAGGCCTGGCGCCC

146241 146276 143160 147587 CDS product transcriptional regulator ICP4 146241-146276 6:03:01 CCTTCGATCCCCAGGCCCTAGCCACCATCGCGGCCC

146285 146304 143160 147587 CDS product transcriptional regulator ICP4 146285-146304 4:01:01 GGACCCCCGGCCAGGGACGG

146383 146399 143160 147587 CDS product transcriptional regulator ICP4 146383-146399 4:01:01 GGACGTGAAGGTGGTGG

146443 146458 143160 147587 CDS product transcriptional regulator ICP4 146443-146458 4:01:01 CCCGGCCGCCCGCCCC

146497 146530 143160 147587 CDS product transcriptional regulator ICP4 146497-146530 6:03:01 GGGGGGACTCTCGGCGCTGCTGGCGGCCCTGGGG

146523 146566 143160 147587 CDS product transcriptional regulator ICP4 146523-146566 7:04:01 CCCTGGGGAACCGCGTCCTCACCAAGCGGTCCCACGCCTGGGCC

146562 146597 143160 147587 CDS product transcriptional regulator ICP4 146562-146597 6:03:01

GGGCCGGCAACTGGACCGGAGCCCCGGACGTCTCGG

146598 146650 143160 147587 CDS product transcriptional regulator ICP4 146598-146650 9:06:02
CCCTCAACGCCCAGGGGGTCCTGCTCCTCTCCACCGGGGACCTGGCCTTCACC

146665 146689 143160 147587 CDS product transcriptional regulator ICP4 146665-146689 6:03:01
CCTCTGCCTCCGCCTGGGCTCCGCC

146728 146745 143160 147587 CDS product transcriptional regulator ICP4 146728-146745 4:01:01
GGACTGGCCCCAGGACGG

146819 146840 143160 147587 CDS product transcriptional regulator ICP4 146819-146840 4:01:01
CCCCGGGAGCGCCACCTCAGCC

146994 147012 143160 147587 CDS product transcriptional regulator ICP4 146994-147012 5:02:01
CCCGCGTCCCCCTGCCTCC

147127 147160 143160 147587 CDS product transcriptional regulator ICP4 147127-147160 6:03:01
CCACCGCGCGGCCAACCGCTGGGGCCTCGGGGCC

147169 147191 143160 147587 CDS product transcriptional regulator ICP4 147169-147191 4:01:01
CCCCGTGTACCTCGCCTGCGGCC

147188 147221 143160 147587 CDS product transcriptional regulator ICP4 147188-147221 6:03:01
GGCCGGCGCGGAGCCGGGGCCGTGGAGCCCTCGG

147275 147296 143160 147587 CDS product transcriptional regulator ICP4 147275-147296 4:01:01
CCCGACGCCGAGGCCGGACCCC

147305 147336 143160 147587 CDS product transcriptional regulator ICP4 147305-147336 6:03:01
CCCATCACCGAGGCTCCCCGCCGCCGGGCCCC

147443 147467 143160 147587 CDS product transcriptional regulator ICP4 147443-147467 5:02:01
GGGGCCGGCGACGTGGAGCTGGTGG

147468  147520  143160  147587  CDS product transcriptional regulator ICP4  147468-147520  10:07:02
CCGACCACCCCGGCCCTAGCCCCGGCGCGGCCCAGGCCCTCCGCCGCGCTCCC
147532  147567  143160  147587  CDS product transcriptional regulator ICP4  147532-147567  7:04:01
GGTGGTGTCGGACGACGAGGACGGTGGAGACTGGGG

gi|281190771|ref|NC_013590.2| Felid herpesvirus 1, complete genome
4446  4464  3482  4798  CDS product multifunctional expression regulator  4446-4464  4:01:01
GGCGCATGTGGATTGGTGG
4664  4695  3482  4798  CDS product multifunctional expression regulator  4664-4695  6:03:01
GGTTCTGGAGGTGTCGGGGAGGTCTCATCGGG
5248  5269  4919  5953  CDS product envelope glycoprotein K  5248-5269  4:01:01 CCCCATCAAGACCACCTTCGCC
5685  5702  4919  5953  CDS product envelope glycoprotein K  5685-5702  4:01:01 CCAGGGCCAAATCCTTCC
6598  6613  5963  9151  CDS product helicase-primase primase subunit  6598-6613  4:01:01 CCCACCATCACCAACC
6783  6801  5963  9151  CDS product helicase-primase primase subunit  6783-6801  5:02:01
GGTATGGAGGTGGTATTGG
7043  7061  5963  9151  CDS product helicase-primase primase subunit  7043-7061  4:01:01
GGTTGAGGGGGGCATGAGG
7170  7188  5963  9151  CDS product helicase-primase primase subunit  7170-7188  4:01:01
GGACTGGAAGTGGGATAGG
7810  7826  5963  9151  CDS product helicase-primase primase subunit  7810-7826  4:01:01
GGGATAGGTTTTGGAGG
8563  8580  5963  9151  CDS product helicase-primase primase subunit  8563-8580  4:01:01
GGAGGTCTCGGGGTTTGG

8886 8905 5963 9151 CDS product helicase-primase primase subunit 8886-8905 5:02:01
GGACGGCGGCTAGATGGTGG
9166 9191 9150 9878 CDS product tegument protein UL51 9166-9191 5:02:01 GGCAGGTCTGTGTGGAAATCCGGTGG
9531 9547 9150 9878 CDS product tegument protein UL51 9531-9547 4:01:01 CCGTAGATACCACCACC
9927 9956 9923 10900 CDS product deoxyuridine triphosphatase 9927-9956 5:02:01
CCCCGGTTGACCCAAATCCTTTATCCCCCC
10031 10059 9923 10900 CDS product deoxyuridine triphosphatase 10031-10059 5:02:01
GGTGCAGGTTCTGGGGTTTCTGGAAATGG
11302 11327 11185 12222 CDS product tegument protein VP22 11302-11327 4:01:01 CCACAGAAACCCCTCCGCGAGTCCCC
11742 11786 11185 12222 CDS product tegument protein VP22 11742-11786 8:05:02
CCTCCCTCACCCACACCAGTTAAACCAAGAGCCGCGCCGGGTACC
11829 11848 11185 12222 CDS product tegument protein VP22 11829-11848 4:01:01 CCAAGAACCTCATCCACGCC
12138 12158 11185 12222 CDS product tegument protein VP22 12138-12158 5:02:01 CCTAAGACCAGATCCTCCTCC
12504 12523 12355 13698 CDS product transactivating tegument protein VP16 12504-12523 4:01:01
CCACCAAAACCTGCTAGCCC
12906 12948 12355 13698 CDS product transactivating tegument protein VP16 12906-12948 8:05:02
GGTGCGCGGCGAGGCCCAAGGGATAAAGGTAAGGGGGTAACGG
13496 13538 12355 13698 CDS product transactivating tegument protein VP16 13496-13538 7:04:01
CCCACCATCACCAAGTGCCGTGCTTCCAGGGGATCCAGATCCC
14430 14446 14066 16642 CDS product tegument protein VP13/14 14430-14446 4:01:01 GGCGGAAGGGGATTCGG
14547 14563 14066 16642 CDS product tegument protein VP13/14 14547-14563 4:01:01 GGTTGGATATGGAGAGG
14638 14662 14066 16642 CDS product tegument protein VP13/14 14638-14662 5:02:01
GGGGATGGGGGGGAAGAGGATGTGG

14800 14818 14066 16642 CDS product tegument protein VP13/14 14800-14818 4:01:01 GGTGGTCGTGGAGCAGAGG
14929 14947 14066 16642 CDS product tegument protein VP13/14 14929-14947 5:02:01 GGAGAATGGCGGAGGCTGG
15222 15241 14066 16642 CDS product tegument protein VP13/14 15222-15241 4:01:01 GGGTTGTTGGTTTGAGGCGG
15524 15541 14066 16642 CDS product tegument protein VP13/14 15524-15541 4:01:01 GGATGGTAGGAAGAACGG
15542 15563 14066 16642 CDS product tegument protein VP13/14 15542-15563 4:01:01 CCATACGACCACTTCCAACACC
15840 15855 14066 16642 CDS product tegument protein VP13/14 15840-15855 4:01:01 GGTGGCGGTGCAGTGG
16372 16389 14066 16642 CDS product tegument protein VP13/14 16372-16389 4:01:01 CCAATGCCGGGTCCACCC
16566 16582 14066 16642 CDS product tegument protein VP13/14 16566-16582 4:01:01 GGGACGGCAAGGGCTGG
16607 16625 14066 16642 CDS product tegument protein VP13/14 16607-16625 4:01:01 GGAGACGAGGGAGGCGGGG
17425 17445 16720 18882 CDS product tegument protein VP11/12 17425-17445 4:01:01 GGTGGCTCTTATGGTTCATGG
17782 17799 16720 18882 CDS product tegument protein VP11/12 17782-17799 4:01:01 GGGCTGAGGGAGGTATGG
18089 18115 16720 18882 CDS product tegument protein VP11/12 18089-18115 6:03:01
CCTCCCACTACCATCCCCACCCACTCC
19536 19554 19053 19634 CDS product membrane protein UL45 19536-19554 4:01:01 GGTGGGTTAGGCTCGTTGG
20670 20691 19884 21488 CDS product envelope glycoprotein C 20670-20691 5:02:01 CCATCTCCACCATAGACCCGCC
21161 21181 19884 21488 CDS product envelope glycoprotein C 21161-21181 4:01:01 GGCTGCCTGGGTGGTACTCGG
21234 21252 19884 21488 CDS product envelope glycoprotein C 21234-21252 4:01:01 GGCTGGGATGCCTGGGTGG
21303 21345 19884 21488 CDS product envelope glycoprotein C 21303-21345 7:04:01
GGAGATGTTGGCTGGGATATCGGGGTGGTAGCGGACATGCTGG
22536 22558 21674 22921 CDS product envelope protein UL43 22536-22558 4:01:01 CCCATCCAACTGCCGCTATGGCC
24942 24970 24698 26059 CDS product tegument host shutoff protein 24942-24970 5:02:01
GGATAGAGGTATCTATGGTGATGGACAGG
25017 25039 24698 26059 CDS product tegument host shutoff protein 25017-25039 5:02:01

GGGTGGATCTGGACGCTTGGGGG

26275 26305 26195 27190 CDS product ribonucleotide reductase subunit 2 26275-26305 6:03:01 GGCTAAGGGAAAATCGGGATGAGGAGGCGGG

26757 26779 26195 27190 CDS product ribonucleotide reductase subunit 2 26757-26779 4:01:01 CCACATAGGCCGTACGCGCCTCC

27127 27170 26195 27190 CDS product ribonucleotide reductase subunit 2 27127-27170 8:05:02 GGATACCGGCATTTTGGTATTGGAGTGGAGACCGGTATTGGGGG

28407 28424 27204 29564 CDS product ribonucleotide reductase subunit 1 28407-28424 5:02:01 CCCATACCAGCCGCCTCC

28513 28539 27204 29564 CDS product ribonucleotide reductase subunit 1 28513-28539 5:02:01 CCTCTCCGCCTAGGTGCCGTTGATACC

29091 29126 27204 29564 CDS product ribonucleotide reductase subunit 1 29091-29126 5:02:01 CCCATCGATTCCAAAGCTCCATTTTCCAGGTTTGCC

29960 29980 29793 31184 CDS product capsid triplex subunit 1 29960-29980 4:01:01 GGCACGGCCGCGCAGGTCAGG

29995 30014 29793 31184 CDS product capsid triplex subunit 1 29995-30014 5:02:01 GGGCTGCGGTGGCTTGGGGG

30075 30105 29793 31184 CDS product capsid triplex subunit 1 30075-30105 6:03:01 GGAAATCTGGCTCGTGGGTCCGTGGCGGGGG

30660 30682 29793 31184 CDS product capsid triplex subunit 1 30660-30682 5:02:01 CCTTCTCCTGAATCCCTACCACC

30773 30808 29793 31184 CDS product capsid triplex subunit 1 30773-30808 7:04:01 GGCTGGATGGCGTAAGGTGAGGATTGGAAGACCCGG

32781 32804 31532 34615 CDS product tegument protein UL37 32781-32804 4:01:01 GGATGAGGCGCAACAGGCAATTGG

33582 33602 31532 34615 CDS product tegument protein UL37 33582-33602 4:01:01 GGTCATGCTGGTCCCGGAGGG

34538 34566 31532 34615 CDS product tegument protein UL37 34538-34566 5:02:01

CCTGGGTTACCCCAGAAGACCTCCTCGCC

36620 36637 34835 43936 CDS product large tegument protein 36620-36637 4:01:01 CCCACCTCGCCGAACGCC
37483 37503 34835 43936 CDS product large tegument protein 37483-37503 4:01:01 GGTGCGGATGAGGTTATGCGG
37686 37708 34835 43936 CDS product large tegument protein 37686-37708 4:01:01 GGCGGGATTTGTTGGCCTGAGGG
37701 37722 34835 43936 CDS product large tegument protein 37701-37722 4:01:01 CCTGAGGGCCGCACCCGGAGCC
38745 38767 34835 43936 CDS product large tegument protein 38745-38767 4:01:01 CCTTACTATCCATCCAGTGATCC
39453 39474 34835 43936 CDS product large tegument protein 39453-39474 5:02:01 CCCCCCACCACCCTCAATAGCC
40682 40705 34835 43936 CDS product large tegument protein 40682-40705 5:02:01 GGCAATATGGCAGGGTCTGGAAGG
41838 41858 34835 43936 CDS product large tegument protein 41838-41858 5:02:01 CCCGGTACCCCCAAAACCACC
42438 42460 34835 43936 CDS product large tegument protein 42438-42460 4:01:01 GGGGGCTTTATCGGATACCTGGG
42755 42779 34835 43936 CDS product large tegument protein 42755-42779 4:01:01 GGGACGGTGACGACGGACCTGATGG
42845 42861 34835 43936 CDS product large tegument protein 42845-42861 4:01:01 CCATATCCGTACCCCCC
44819 44836 44482 45297 CDS product nuclear egress membrane protein 44819-44836 4:01:01 CCTCCGGGCCCATGAACC
45195 45214 44482 45297 CDS product nuclear egress membrane protein 45195-45214 4:01:01 GGGTGGATTTTTTGGGTTGG
46137 46154 45831 47579 CDS product DNA packaging protein UL32 46137-46154 4:01:01 GGCGGAAGTTTGGATTGG
47210 47224 45831 47579 CDS product DNA packaging protein UL32 47210-47224 4:01:01 GGGGATGGTGGGCGG
47468 47494 45831 47579 CDS product DNA packaging protein UL32 47468-47494 5:02:01
CCTCCCAAGCCAACGGCCGGGGCAGCC
49110 49130 48509 52126 CDS product DNA polymerase catalytic subunit 49110-49130 4:01:01
CCAGTACCCTTCCAAATTCCC
49679 49701 48509 52126 CDS product DNA polymerase catalytic subunit 49679-49701 4:01:01
CCATTAGCCACCCCGGTAAATCC
49777 49796 48509 52126 CDS product DNA polymerase catalytic subunit 49777-49796 4:01:01

GGGAATACGGGCCCGGATGG

50232 50251 48509 52126 CDS product DNA polymerase catalytic subunit 50232-50251 4:01:01 GGTTGCGGTTATCAGGTAGG

50432 50457 48509 52126 CDS product DNA polymerase catalytic subunit 50432-50457 5:02:01 CCTATTATCCCCCGTTGCCTGGCACC

50508 50524 48509 52126 CDS product DNA polymerase catalytic subunit 50508-50524 4:01:01 CCCCTAAGGCCTCCTCC

51549 51571 48509 52126 CDS product DNA polymerase catalytic subunit 51549-51571 4:01:01 CCCTATCCACTAGATCCTTATCC

53294 53314 52392 55994 CDS product single-stranded DNA-binding protein 53294-53314 4:01:01 GGGGGAGATGGAGCCTCTGGG

53509 53523 52392 55994 CDS product single-stranded DNA-binding protein 53509-53523 4:01:01 GGCTGGATTGGTAGG

53576 53601 52392 55994 CDS product single-stranded DNA-binding protein 53576-53601 5:02:01 GGTGGGGCCGTGGATGGTAAAGATGG

53620 53643 52392 55994 CDS product single-stranded DNA-binding protein 53620-53643 4:01:01 CCGTTTTTACCTAATTCCAGCACC

54908 54935 52392 55994 CDS product single-stranded DNA-binding protein 54908-54935 6:03:01 CCACCCGGGAGCCAGACTCCAAACCCCC

54964 54983 52392 55994 CDS product single-stranded DNA-binding protein 54964-54983 4:01:01 CCAGATGCCGGCCCGTCTCC

55424 55449 52392 55994 CDS product single-stranded DNA-binding protein 55424-55449 5:02:01 GGAAATTGGAGTGGGTTGAATGGAGG

55538 55553 52392 55994 CDS product single-stranded DNA-binding protein 55538-55553 5:02:01 GGGGGTGGTGGGGGGG

55601 55622 52392 55994 CDS product single-stranded DNA-binding protein 55601-55622 4:01:01 GGTGGACCCATGGTTCAAACGG
57019 57043 56050 58389 CDS product DNA packaging terminase subunit 2 57019-57043 5:02:01 GGCTGGCCTCAGCGGGATTGGACGG
57303 57328 56050 58389 CDS product DNA packaging terminase subunit 2 57303-57328 5:02:01 CCACTACTCCGCCAACTGACCAACCC
57436 57464 56050 58389 CDS product DNA packaging terminase subunit 2 57436-57464 5:02:01 CCCAATCGGACCGGGCCATAGACTCCCCC
58000 58014 56050 58389 CDS product DNA packaging terminase subunit 2 58000-58014 4:01:01 GGGGTGGGTGGACGG
58253 58302 56050 58389 CDS product DNA packaging terminase subunit 2 58253-58302 7:04:01 GGCGATCTTGGGAAGCGGCGACGAGGGAGTCGTTGGCAGGGACACAGTGG
58661 58677 58242 61088 CDS product envelope glycoprotein B 58661-58677 4:01:01 CCACCACGGGCCTGTCC
58809 58829 58242 61088 CDS product envelope glycoprotein B 58809-58829 4:01:01 CCTATGCCGTTACAACCAACC
59589 59609 58242 61088 CDS product envelope glycoprotein B 59589-59609 4:01:01 CCCTGGAGACCTACCTAGCCC
59996 60014 58242 61088 CDS product envelope glycoprotein B 59996-60014 4:01:01 GGTGGGGTGGTCTCGATGG
60475 60493 58242 61088 CDS product envelope glycoprotein B 60475-60493 4:01:01 CCGCAACCAACTCCACGCC
60574 60620 58242 61088 CDS product envelope glycoprotein B 60574-60620 9:06:02 GGGACTCGGGGATGTGGGGGCTGGTTTCGGCAAGGTGGTCTTAGGGG
61008 61040 58242 61088 CDS product envelope glycoprotein B 61008-61040 6:03:01 CCCTCCGTCGCCGTGGACCTAAATACCAACGCC
62292 62306 61769 62653 CDS product capsid scaffold protein 62292-62306 4:01:01 GGGACGGGGAGGTGG
62435 62467 61769 62653 CDS product capsid scaffold protein 62435-62467 8:05:02 GGATGGAAATGGTTTGGAGGGAGGGGTGGATGG

| | | | | | |
|---|---|---|---|---|---|
| 62509 | 62529 | 61769 | 62653 | CDS product capsid scaffold protein | 62509-62529 5:02:01 GGCCGGGGGGTATTGGATAGG |
| 62609 | 62630 | 61769 | 62653 | CDS product capsid scaffold protein | 62609-62630 5:02:01 GGGGGTTGTGGATGGCTTATGG |
| 63254 | 63277 | 61769 | 63565 | CDS product capsid maturation protease | 63254-63277 4:01:01 CCCTCCTTTACTCCAAAAAACCCC |
| 64161 | 64183 | 63696 | 65567 | CDS product DNA packaging tegument protein UL25 | 64161-64183 4:01:01 CCCAAATCCCCCCCGGTTGCCCC |
| 65131 | 65159 | 63696 | 65567 | CDS product DNA packaging tegument protein UL25 | 65131-65159 6:03:01 CCGCCGTTTCCGCTTCCCGTGCTACCTCC |
| 66100 | 66114 | 65590 | 66372 | CDS product nuclear protein UL24 | 66100-66114 4:01:01 CCACCCCCTTCTCCC |
| 66322 | 66341 | 65590 | 66372 | CDS product nuclear protein UL24 | 66322-66341 4:01:01 CCAGCCCGGAGCCTCAATCC |
| 67065 | 67081 | 66387 | 67418 | CDS product thymidine kinase | 67065-67081 4:01:01 GGCGTGATGGGTGGGGG |
| 68200 | 68215 | 67658 | 70123 | CDS product envelope glycoprotein H | 68200-68215 5:02:01 CCGACCACCTCCGGCC |
| 68340 | 68358 | 67658 | 70123 | CDS product envelope glycoprotein H | 68340-68358 4:01:01 GGAGGGGATGTGGATATGG |
| 69858 | 69872 | 67658 | 70123 | CDS product envelope glycoprotein H | 69858-69872 4:01:01 GGAGTTGGTGGCTGG |
| 70423 | 70439 | 70246 | 71829 | CDS product tegument protein UL21 | 70423-70439 4:01:01 CCATCTATTCCGCCACC |
| 71029 | 71053 | 70246 | 71829 | CDS product tegument protein UL21 | 71029-71053 4:01:01 CCAGATGCCCGATCCCTAGGATCCC |
| 71648 | 71663 | 70246 | 71829 | CDS product tegument protein UL21 | 71648-71663 4:01:01 CCCCTTCCCCCACACC |
| 73047 | 73069 | 73018 | 77151 | CDS product major capsid protein | 73047-73069 4:01:01 CCAATAGCCGATTTCACCACCCC |
| 73338 | 73357 | 73018 | 77151 | CDS product major capsid protein | 73338-73357 4:01:01 CCCCACCCTGTAAACCAACC |
| 73860 | 73885 | 73018 | 77151 | CDS product major capsid protein | 73860-73885 4:01:01 GGCATACGGCAACGGCTCTTAAGGGG |
| 74286 | 74323 | 73018 | 77151 | CDS product major capsid protein | 74286-74323 6:03:01 CCAGCTCCCGGACAACCCGATTCCCGTGTTTTCCCACC |
| 74684 | 74707 | 73018 | 77151 | CDS product major capsid protein | 74684-74707 6:03:01 CCAGCCAGGTCCACCAGAACCACC |
| 74834 | 74852 | 73018 | 77151 | CDS product major capsid protein | 74834-74852 5:02:01 CCCAGCCACCGTTGCCGCC |

75158 75177 73018 77151 CDS product major capsid protein 75158-75177 4:01:01 GGCTGGTCGGGATCACGAGG
75200 75217 73018 77151 CDS product major capsid protein 75200-75217 4:01:01 CCATGCCCTTCTCCCCCC
75311 75336 73018 77151 CDS product major capsid protein 75311-75336 4:01:01 GGTACCGTGGGTCGAGATGGCCCAGG
75397 75427 73018 77151 CDS product major capsid protein 75397-75427 5:02:01
CCAGGGCCCCAATCATCCCACACCACGGTCC
75554 75576 73018 77151 CDS product major capsid protein 75554-75576 5:02:01 GGATCTGGGAAGGGATGAGGAGG
75579 75616 73018 77151 CDS product major capsid protein 75579-75616 7:04:01
CCTGCATCACCCGATGACCCCCGCCACCCTCTAAACCC
75735 75758 73018 77151 CDS product major capsid protein 75735-75758 5:02:01 CCCATTCTCACCGCCGCCGGACCC
75772 75786 73018 77151 CDS product major capsid protein 75772-75786 4:01:01 CCACGGCCACCACCC
75978 75996 73018 77151 CDS product major capsid protein 75978-75996 5:02:01 CCACCCGTACCGCCTTTCC
76268 76279 73018 77151 CDS product major capsid protein 76268-76279 4:01:01 GGCGGTAGGTGG
76306 76330 73018 77151 CDS product major capsid protein 76306-76330 4:01:01 GGGCCAACGTGGATCTGGGTGTAGG
76745 76764 73018 77151 CDS product major capsid protein 76745-76764 4:01:01 CCCATGGGCCTCCCAACGCC
76903 76931 73018 77151 CDS product major capsid protein 76903-76931 5:02:01
CCGGGTCCAGTTTAGCCCCAAGCACCGCC
77595 77619 77323 78261 CDS product capsid triplex subunit 2 77595-77619 5:02:01
CCACTTCCTCCTGGTGCCTTTATCC
77733 77754 77323 78261 CDS product capsid triplex subunit 2 77733-77754 4:01:01 CCATCCACTGTACCAGTAGCCC
77805 77829 77323 78261 CDS product capsid triplex subunit 2 77805-77829 6:03:01
GGTGCCGGTGGGAGACGGGGGATGG
77965 77988 77323 78261 CDS product capsid triplex subunit 2 77965-77988 4:01:01 CCTTGATACCGAACCTACTCACCC
78052 78087 77323 78261 CDS product capsid triplex subunit 2 78052-78087 7:04:01

CCCAACTAATCCATCGACCACCAGTCCACCCTCCCC
78406 78425 78345 83970 CDS product DNA packaging terminase subunit 1 78406-78425 4:01:01 CCGCCATTACCAGAGCTACC
78704 78718 78345 83970 CDS product DNA packaging terminase subunit 1 78704-78718 4:01:01 GGTCGGAGGAATGGG
79504 79524 78345 83970 CDS product DNA packaging terminase subunit 1 79504-79524 4:01:01 GGCCTCGGATACCCTGGAGGG
79930 79954 79666 81741 CDS product DNA packaging tegument protein UL17 79930-79954 4:01:01 CCAATACCCTTGTGCCAACATGTCC
80284 80301 79666 81741 CDS product DNA packaging tegument protein UL17 80284-80301 4:01:01 CCAACCACACCCAATCCC
80339 80353 79666 81741 CDS product DNA packaging tegument protein UL17 80339-80353 4:01:01 GGTGAATGGAGGGGG
80425 80452 79666 81741 CDS product DNA packaging tegument protein UL17 80425-80452 5:02:01 CCTCGGAGGCCATAACCACCAGACACCC
80480 80503 79666 81741 CDS product DNA packaging tegument protein UL17 80480-80503 5:02:01 GGATGGTTGTGGAGTGGCATCTGG
80866 80880 79666 81741 CDS product DNA packaging tegument protein UL17 80866-80880 4:01:01 GGGGTGGGAGGAGGG
81029 81040 79666 81741 CDS product DNA packaging tegument protein UL17 81029-81040 4:01:01 CCTGCCACCACC
81455 81471 79666 81741 CDS product DNA packaging tegument protein UL17 81455-81471 4:01:01 GGCGGAACCTTTGGCGG
82174 82186 81772 82863 CDS product tegument protein UL16 82174-82186 4:01:01 CCATCCCCCTCCC
82313 82325 81772 82863 CDS product tegument protein UL16 82313-82325 4:01:01 GGGGGTATGGTGG
82366 82388 81772 82863 CDS product tegument protein UL16 82366-82388 4:01:01 CCACGCTCCTCGCCCTATGCCCC
83055 83079 78345 83970 CDS product DNA packaging terminase subunit 1 83055-83079 4:01:01

CCTCGCTCCAATTTCCTCAAACACC
83648 83672 78345 83970 CDS product DNA packaging terminase subunit 1 83648-83672 6:03:01
CCGAACCGGACACCGCCTCCTCCCC
83784 83810 78345 83970 CDS product DNA packaging terminase subunit 1 83784-83810 5:02:01
GGGTACAACGGTCTGGTGGCGCTGGGG
84529 84549 84007 84975 CDS product tegument protein UL14 84529-84549 6:03:01 GGGGGTCCGGGAAGGGGGGGG
84748 84765 84007 84975 CDS product tegument protein UL14 84748-84765 4:01:01 GGCTGGTAACGGAGAAGG
85139 85158 84417 86237 CDS product tegument serine/threonine protein kinase 85139-85158 5:02:01
GGGGGGGCTGGTGGGTATGG
85445 85463 84417 86237 CDS product tegument serine/threonine protein kinase 85445-85463 4:01:01
GGGTTGGGTAAGGCTGTGG
85855 85879 84417 86237 CDS product tegument serine/threonine protein kinase 85855-85879 4:01:01
CCTTTCCCCTCGGTTACCGGTCCCC
86229 86248 86222 87856 CDS product deoxyribonuclease 86229-86248 4:01:01 CCAATTAACCAACCCACGCC
86746 86770 86222 87856 CDS product deoxyribonuclease 86746-86770 4:01:01 CCCTATGGACCTGTATACCCACCCC
87139 87158 86222 87856 CDS product deoxyribonuclease 87139-87158 4:01:01 CCCGAATCCTCTTCCCCGCC
87476 87497 86222 87856 CDS product deoxyribonuclease 87476-87497 4:01:01 CCACGCCCATCTTCGCCAATCC
88684 88706 88143 89411 CDS product envelope glycoprotein M 88684-88706 4:01:01 GGGATGTAGTGGCGATGGAGAGG
89511 89532 89398 91980 CDS product DNA replication origin-binding helicase 89511-89532 4:01:01
GGAGGTGATTTGGGAGAGTGGG
89592 89612 89398 91980 CDS product DNA replication origin-binding helicase 89592-89612 5:02:01
CCATCACCAGTCTCCACCACC
90425 90444 89398 91980 CDS product DNA replication origin-binding helicase 90425-90444 4:01:01

GGAGGAAGTTGGGGTCTGGG

92278 92294 91995 94343 CDS product helicase-primase subunit 92278-92294 4:01:01 GGCAGCGGCTGGAGAGG

92513 92530 91995 94343 CDS product helicase-primase subunit 92513-92530 4:01:01 CCGGTGCCGGTCCCAGCC

92599 92618 91995 94343 CDS product helicase-primase subunit 92599-92618 5:02:01 GGGGGGAGGATTGGCAAGGG

93128 93147 91995 94343 CDS product helicase-primase subunit 93128-93147 4:01:01 GGTATGTGGCCGGCAATGGG

93426 93455 91995 94343 CDS product helicase-primase subunit 93426-93455 5:02:01 GGGAAAAGGGAGGGCTGAAGGCAGCATTGG

94175 94196 91995 94343 CDS product helicase-primase subunit 94175-94196 4:01:01 CCACATCCGGCCATTATTCCCC

94545 94559 94402 95292 CDS product tegument protein UL7 94545-94559 4:01:01 CCGCCTCTCCGATCC

96909 96939 95177 97330 CDS product capsid portal protein 96909-96939 6:03:01 CCCTTACCGCTTCCGCCTCACCGTGGTTACC

97864 97880 97406 99997 CDS product helicase-primase helicase subunit 97864-97880 4:01:01 CCTTCTAACCCACCACC

99048 99070 97406 99997 CDS product helicase-primase helicase subunit 99048-99070 5:02:01 GGATGGAATACTGGTCTCGGAGG

99573 99592 97406 99997 CDS product helicase-primase helicase subunit 99573-99592 4:01:01 CCTTAATCGCCGCCGAATCC

100072 100092 100065 100775 CDS product nuclear protein UL4 100072-100092 5:02:01 CCACCAGACAACCTTCCACCC

100394 100415 100065 100775 CDS product nuclear protein UL4 100394-100415 5:02:01 GGTTGGTCCGGTGTTGGGATGG

101322 101346 100841 101386 CDS product protein V57 101322-101346 5:02:01 CCATGGAAGCCGCCCTCCCAACACC

101943 101961 101390 101995 CDS product nuclear protein UL3 101943-101961 4:01:01 CCGGCCATCCCCATTCCCC

102543 102573 102101 102982 CDS product uracil-DNA glycosylase 102543-102573 5:02:01 GGCCATTGGCTTGACCGGGTCCGTGGTATGG

103079 103089 103037 103480 CDS product envelope glycoprotein L 103079-103089 4:01:01 GGAGGCGGTGG
104207 104229 104175 105671 CDS product ubiquitin E3 ligase ICP0 104207-104229 4:01:01 CCCAGATACCCTCCTACGACCCC
104441 104460 104175 105671 CDS product ubiquitin E3 ligase ICP0 104441-104460 4:01:01 GGTGTTGGGAGGTCCATCGG
105629 105653 104175 105671 CDS product ubiquitin E3 ligase ICP0 105629-105653 4:01:01 GGGATCAAGGCATATTGGACAGTGG
107245 107263 107219 111415 CDS product transcriptional regulator ICP4 107245-107263 4:01:01 CCATCCACTCTCCGCGTCC
107430 107453 107219 111415 CDS product transcriptional regulator ICP4 107430-107453 4:01:01 CCTGTTTTCCAAATCCCGAGTCCC
107668 107687 107219 111415 CDS product transcriptional regulator ICP4 107668-107687 4:01:01 GGCACGATGGCTGTGGGCGG
107736 107754 107219 111415 CDS product transcriptional regulator ICP4 107736-107754 4:01:01 CCTCCATATCCTTACACCC
107790 107815 107219 111415 CDS product transcriptional regulator ICP4 107790-107815 4:01:01 GGGGTAACATGGGAACGGGTGTGCGG
107965 107985 107219 111415 CDS product transcriptional regulator ICP4 107965-107985 4:01:01 GGAGAGGTCCTCTAGGGCCGG
107973 107995 107219 111415 CDS product transcriptional regulator ICP4 107973-107995 4:01:01 CCTCTAGGGCCGGCCAGCGCACC
108007 108058 107219 111415 CDS product transcriptional regulator ICP4 108007-108058 9:06:02 GGTCGGGGTGACCGTGGCGCGGATGTATAGGTGGTAATGGCTCACGGCCGGG
108054 108071 107219 111415 CDS product transcriptional regulator ICP4 108054-108071 4:01:01

CCGGGCCCGTCGCCGGCC
108216 108242 107219 111415 CDS product transcriptional regulator ICP4 108216-108242 5:02:01
CCGGGGGACCACTCCAATTCCCTGCCC
108256 108271 107219 111415 CDS product transcriptional regulator ICP4 108256-108271 5:02:01 GGGGGAGAGGAGGCGG
108468 108500 107219 111415 CDS product transcriptional regulator ICP4 108468-108500 8:05:02
CCACCAGCCCCCCGAGACCTGTACCCTCCTGCC
108646 108659 107219 111415 CDS product transcriptional regulator ICP4 108646-108659 4:01:01 GGTGGGTCTGGGGG
108719 108747 107219 111415 CDS product transcriptional regulator ICP4 108719-108747 5:02:01
GGCATGGGTCCAAGGGGACCCCTAGGGGG
108763 108774 107219 111415 CDS product transcriptional regulator ICP4 108763-108774 4:01:01 GGTGGGTGGTGG
108817 108861 107219 111415 CDS product transcriptional regulator ICP4 108817-108861 7:04:01
GGCATCGATGGGGAGGTTGCCGGATGCGTGGCCCCGGGGAAGCGG
108889 108921 107219 111415 CDS product transcriptional regulator ICP4 108889-108921 7:04:01
GGATTGGGGGCGGGTGAGGTGGGGTGAAAGGGG
109029 109047 107219 111415 CDS product transcriptional regulator ICP4 109029-109047 5:02:01
CCTCCACCGTGACTCCCCC
109066 109106 107219 111415 CDS product transcriptional regulator ICP4 109066-109106 9:06:02
GGTCGCCCGGGGGGTGGGAGTATGGGGGTCGGGGGTACCGG
109103 109117 107219 111415 CDS product transcriptional regulator ICP4 109103-109117 4:01:01 CCGGCGACCTCCTCC
109215 109232 107219 111415 CDS product transcriptional regulator ICP4 109215-109232 4:01:01
GGGGGGCCAGGGTCCTGG
109397 109435 107219 111415 CDS product transcriptional regulator ICP4 109397-109435 6:03:01
GGGGGGCAGACCGGCGAGGGGTACTTGGCCACCGAGGGG

109579 109599 107219 111415 CDS product transcriptional regulator ICP4 109579-109599 4:01:01
GGGAGTTGGTATCTGGGTGGG
109602 109630 107219 111415 CDS product transcriptional regulator ICP4 109602-109630 6:03:01
CCCCCGTTCCCCTGGTCGTCCCCCCCGCC
109687 109716 107219 111415 CDS product transcriptional regulator ICP4 109687-109716 6:03:01
GGATGTGGGGGTTTTTGGGGGGGCGTCGGG
109881 109916 107219 111415 CDS product transcriptional regulator ICP4 109881-109916 6:03:01
GGGGGAGAGCCAGGAGAGGGTCCATGGTCGCCATGG
109929 109943 107219 111415 CDS product transcriptional regulator ICP4 109929-109943 4:01:01 GGGGGAGGGGATTGG
109977 109991 107219 111415 CDS product transcriptional regulator ICP4 109977-109991 4:01:01 GGGGGCCGTGGATGG
110140 110162 107219 111415 CDS product transcriptional regulator ICP4 110140-110162 4:01:01
GGGAACCGGGCCGGGGTCCGCGG
110190 110232 107219 111415 CDS product transcriptional regulator ICP4 110190-110232 7:04:01
CCGGAGCGTCCCACAGACCTTCCCTCGAGTCCCCGATCCCCCC
110268 110283 107219 111415 CDS product transcriptional regulator ICP4 110268-110283 4:01:01 CCGGCCAGACCTCTCC
110300 110316 107219 111415 CDS product transcriptional regulator ICP4 110300-110316 4:01:01
GGTCCTGTGGTTGGGGG
110341 110363 107219 111415 CDS product transcriptional regulator ICP4 110341-110363 4:01:01
CCTTTCCGAGATCCTCTCGGCCC
110383 110421 107219 111415 CDS product transcriptional regulator ICP4 110383-110421 7:04:01
GGCCCGCGGAAGTCTGGAACCTGGGGGGCGGGATAGCGG
110584 110613 107219 111415 CDS product transcriptional regulator ICP4 110584-110613 6:03:01
GGCAGGCCGAGGGGGAGACGGGGTCCCCGG

110630 110666 107219 111415 CDS product transcriptional regulator ICP4 110630-110666 7:04:01
GGTGGTGCTGGTGGCGATGGGGACCGTGAGGTGCTGG
110878 110933 107219 111415 CDS product transcriptional regulator ICP4 110878-110933 9:06:02
GGGGCTATGGGCGGATGAATCGGTGATGGAGAGGGCGTTGCTGGGATGGGAACAGG
111056 111089 107219 111415 CDS product transcriptional regulator ICP4 111056-111089 8:05:02
GGGGGTGGTGGTGGTTTAGTGGTGGGTCCCGAGG
114453 114474 114384 115388 CDS product regulatory protein ICP22 114453-114474 4:01:01
GGAGCGGTCGATTGGGTCATGG
114539 114556 114384 115388 CDS product regulatory protein ICP22 114539-114556 4:01:01 CCCAGTCCCATCCTCCCC
114916 114934 114384 115388 CDS product regulatory protein ICP22 114916-114934 4:01:01 CCAACGTCCGACCAGAGCC
114977 115017 114384 115388 CDS product regulatory protein ICP22 114977-115017 7:04:01
CCCCTGCGAGCCACCTTACGCCACCTCAATACCACTGAACC
115112 115124 114384 115388 CDS product regulatory protein ICP22 115112-115124 4:01:01 GGTGAGGGGGTGG
115243 115261 114384 115388 CDS product regulatory protein ICP22 115243-115261 4:01:01 GGATGAAACGGAGGATCGG
115707 115730 115629 116270 CDS product virion protein US10 115707-115730 4:01:01 GGGGGGACTCCGCGGATCACGCGG
115828 115852 115629 116270 CDS product virion protein US10 115828-115852 4:01:01 GGACATATGGCCCGGGGTATATCGG
115891 115905 115629 116270 CDS product virion protein US10 115891-115905 4:01:01 GGAGGGTAGCGGGGG
115970 115992 115629 116270 CDS product virion protein US10 115970-115992 4:01:01 CCCGCAAAACCAGAATCCCATCC
118694 118713 117698 118753 CDS product serine/threonine protein kinase US3 118694-118713 4:01:01
CCAAGTTCCGACCATCGGCC
119461 119485 118880 120184 CDS product envelope glycoprotein G 119461-119485 4:01:01
CCATTTGACCATCATCCCCAAAGCC
119911 119927 118880 120184 CDS product envelope glycoprotein G 119911-119927 5:02:01 CCACCACCAACCCATCC

121287 121316 120583 121707 CDS product envelope glycoprotein D 121287-121316 5:02:01
CCCTATAACCCACAACCTCACCATAAAGCC
122129 122150 121834 122988 CDS product envelope glycoprotein I 122129-122150 4:01:01 CCGGTCCTGTCTCCACAAGACC
123559 123577 123167 124765 CDS product envelope glycoprotein E 123559-123577 4:01:01 GGCCTTGGTATTCAGGGGG
123915 123930 123167 124765 CDS product envelope glycoprotein E 123915-123930 4:01:01 CCCCCATGCCGCATCC
125548 125577 125212 125670 CDS product membrane protein US9 125548-125577 7:04:01
GGCGACGGAGGCGGTGTCTGGTGGCACTGG
126239 126261 125963 126604 CDS product virion protein US10 126239-126261 4:01:01 GGATGGGATTCTGGTTTTGCGGG
126326 126340 125963 126604 CDS product virion protein US10 126326-126340 4:01:01 CCCCCGCTACCCTCC
126379 126403 125963 126604 CDS product virion protein US10 126379-126403 4:01:01 CCGATATACCCCGGGCCATATGTCC
126501 126524 125963 126604 CDS product virion protein US10 126501-126524 4:01:01 CCGCGTGATCCGCGGAGTCCCCCC
126970 126988 126845 127849 CDS product regulatory protein ICP22 126970-126988 4:01:01 CCGATCCTCCGTTTCATCC
127107 127119 126845 127849 CDS product regulatory protein ICP22 127107-127119 4:01:01 CCACCCCCTCACC
127214 127254 126845 127849 CDS product regulatory protein ICP22 127214-127254 7:04:01
GGTTCAGTGGTATTGAGGTGGCGTAAGGTGGCTCGCAGGGG
127297 127315 126845 127849 CDS product regulatory protein ICP22 127297-127315 4:01:01 GGCTCTGGTCGGACGTTGG
127675 127692 126845 127849 CDS product regulatory protein ICP22 127675-127692 4:01:01 GGGGAGGATGGGACTGGG
127757 127778 126845 127849 CDS product regulatory protein ICP22 127757-127778 4:01:01
CCATGACCCAATCGACCGCTCC
131142 131175 130818 135014 CDS product transcriptional regulator ICP4 131142-131175 8:05:02
CCTCGGGACCCACCACTAAACCACCACCACCCCC
131298 131353 130818 135014 CDS product transcriptional regulator ICP4 131298-131353 9:06:02
CCTGTTCCCATCCCAGCAACGCCCTCTCCATCACCGATTCATCCGCCCATAGCCCC

131565 131601 130818 135014 CDS product transcriptional regulator ICP4 131565-131601 7:04:01 CCAGCACCTCACGGTCCCCATCGCCACCAGCACCACC
131618 131647 130818 135014 CDS product transcriptional regulator ICP4 131618-131647 6:03:01 CCGGGGACCCCGTCTCCCCCTCGGCCTGCC
131810 131848 130818 135014 CDS product transcriptional regulator ICP4 131810-131848 7:04:01 CCGCTATCCCGCCCCCCAGGTTCCAGACTTCCGCGGGCC
131868 131890 130818 135014 CDS product transcriptional regulator ICP4 131868-131890 4:01:01 GGGCCGAGAGGATCTCGGAAAGG
131915 131931 130818 135014 CDS product transcriptional regulator ICP4 131915-131931 4:01:01 CCCCCAACCACAGGACC
131948 131963 130818 135014 CDS product transcriptional regulator ICP4 131948-131963 4:01:01 GGAGAGGTCTGGCCGG
131999 132041 130818 135014 CDS product transcriptional regulator ICP4 131999-132041 7:04:01 GGGGGGATCGGGGACTCGAGGGAAGGTCTGTGGGACGCTCCGG
132069 132091 130818 135014 CDS product transcriptional regulator ICP4 132069-132091 4:01:01 CCGCGGACCCCGGCCCGGTTCCC
132240 132254 130818 135014 CDS product transcriptional regulator ICP4 132240-132254 4:01:01 CCATCCACGGCCCCC
132288 132302 130818 135014 CDS product transcriptional regulator ICP4 132288-132302 4:01:01 CCAATCCCCTCCCCC
132315 132350 130818 135014 CDS product transcriptional regulator ICP4 132315-132350 6:03:01 CCATGGCGACCATGGACCCTCTCCTGGCTCTCCCCC
132515 132544 130818 135014 CDS product transcriptional regulator ICP4 132515-132544 6:03:01 CCCGACGCCCCCCCAAAAACCCCCACATCC
132601 132629 130818 135014 CDS product transcriptional regulator ICP4 132601-132629 6:03:01 GGCGGGGGGGACGACCAGGGGAACGGGGG

132632 132652 130818 135014 CDS product transcriptional regulator ICP4 132632-132652 4:01:01
CCCACCCAGATACCAACTCCC
132796 132834 130818 135014 CDS product transcriptional regulator ICP4 132796-132834 6:03:01
CCCCTCGGTGGCCAAGTACCCCTCGCCGGTCTGCCCCCC
132999 133016 130818 135014 CDS product transcriptional regulator ICP4 132999-133016 4:01:01
CCAGGACCCTGGCCCCCC
133114 133128 130818 135014 CDS product transcriptional regulator ICP4 133114-133128 4:01:01 GGAGGAGGTCGCCGG
133125 133165 130818 135014 CDS product transcriptional regulator ICP4 133125-133165 9:06:02
CCGGTACCCCCGACCCCCATACTCCCACCCCCCGGGCGACC
133184 133202 130818 135014 CDS product transcriptional regulator ICP4 133184-133202 5:02:01
GGGGGAGTCACGGTGGAGG
133310 133342 130818 135014 CDS product transcriptional regulator ICP4 133310-133342 7:04:01
CCCCTTTCACCCCACCTCACCCGCCCCCAATCC
133370 133414 130818 135014 CDS product transcriptional regulator ICP4 133370-133414 7:04:01
CCGCTTCCCCGGGGCCACGCATCCGGCAACCTCCCCATCGATGCC
133457 133468 130818 135014 CDS product transcriptional regulator ICP4 133457-133468 4:01:01 CCACCACCCACC
133484 133512 130818 135014 CDS product transcriptional regulator ICP4 133484-133512 5:02:01
CCCCCTAGGGGTCCCCTTGGACCCATGCC
133572 133585 130818 135014 CDS product transcriptional regulator ICP4 133572-133585 4:01:01 CCCCCAGACCCACC
133731 133763 130818 135014 CDS product transcriptional regulator ICP4 133731-133763 8:05:02
GGCAGGAGGGTACAGGTCTCGGGGGGCTGGTGG
133960 133975 130818 135014 CDS product transcriptional regulator ICP4 133960-133975 5:02:01 CCGCCTCCTCTCCCCC
133989 134015 130818 135014 CDS product transcriptional regulator ICP4 133989-134015 5:02:01

GGGCAGGGAATTGGAGTGGTCCCCCGG

134160 134177 130818 135014 CDS product transcriptional regulator ICP4 134160-134177 4:01:01
GGCCGGCGACGGGCCCGG

134173 134224 130818 135014 CDS product transcriptional regulator ICP4 134173-134224 9:06:02
CCCGGCCGTGAGCCATTACCACCTATACATCCGCGCCACGGTCACCCCGACC

134236 134258 130818 135014 CDS product transcriptional regulator ICP4 134236-134258 4:01:01
GGTGCGCTGGCCGGCCCTAGAGG

134246 134266 130818 135014 CDS product transcriptional regulator ICP4 134246-134266 4:01:01
CCGGCCCTAGAGGACCTCTCC

134416 134441 130818 135014 CDS product transcriptional regulator ICP4 134416-134441 4:01:01
CCGCACACCCGTTCCCATGTTACCCC

134477 134495 130818 135014 CDS product transcriptional regulator ICP4 134477-134495 4:01:01
GGGTGTAAGGATATGGAGG

134544 134563 130818 135014 CDS product transcriptional regulator ICP4 134544-134563 4:01:01
CCGCCCACAGCCATCGTGCC

134778 134801 130818 135014 CDS product transcriptional regulator ICP4 134778-134801 4:01:01
GGGACTCGGGATTTGGAAAACAGG

134968 134986 130818 135014 CDS product transcriptional regulator ICP4 134968-134986 4:01:01
GGACGCGGAGAGTGGATGG

gi|386522723|ref|NC_017826.1| Equid herpesvirus 8, complete genome

450 487 420 1028 CDS product membrane protein UL56 450-487 8:05:02 CCTCCTCGGTTTCCATTTCCATGTGTCCACCACCGCCC

680 693 420 1028 CDS product membrane protein UL56 680-693 4:01:01 CCATCCAGGCCGCC

1332 1356 1069 1683 CDS product membrane protein V1 1332-1356 4:01:01 GGCATCGGTACAGCGGTCTGTGGGG
1369 1383 1069 1683 CDS product membrane protein V1 1369-1383 4:01:01 GGTGGTGAGGCGGGG
1518 1536 1069 1683 CDS product membrane protein V1 1518-1536 4:01:01 GGTGGAGGGGCGCGCGTGG
2237 2263 1960 2733 CDS product myristylated tegument protein CIRC 2237-2263 5:02:01
GGTCTTTGGCTACGAGGATGGTACCGG
2456 2491 1960 2733 CDS product myristylated tegument protein CIRC 2456-2491 6:03:01
CCTACTGGGCCAGACCAAGAGCTCCACGCACCGTCC
2557 2575 1960 2733 CDS product myristylated tegument protein CIRC 2557-2575 4:01:01
GGCTAAGAGGCAAGGGAGG
3176 3192 2766 3368 CDS product nuclear protein UL55 3176-3192 4:01:01 GGCTGGCTGGGATGTGG
3795 3810 3588 5000 CDS product multifunctional expression regulator 3795-3810 4:01:01
CCGCTCCCAACCGCCC
4515 4531 3588 5000 CDS product multifunctional expression regulator 4515-4531 4:01:01
CCTGCGCCGCCATGTCC
4595 4628 3588 5000 CDS product multifunctional expression regulator 4595-4628 5:02:01
CCGCTTTGGCCAGCTCCGCGCTGCCTGGTGCGCC
4717 4734 3588 5000 CDS product multifunctional expression regulator 4717-4734 4:01:01
CCTGGCCAAACCCCGGCC
6798 6824 6180 9422 CDS product helicase-primase primase subunit 6798-6824 5:02:01
CCTCCGTAAACCTCGCCAGAGTCGCCC
6839 6857 6180 9422 CDS product helicase-primase primase subunit 6839-6857 4:01:01
GGGAAGGCGAAGGCTGCGG
6994 7015 6180 9422 CDS product helicase-primase primase subunit 6994-7015 6:03:01

GGAGGTAAGGGGGTGGCACTGG

7744 7761 6180 9422 CDS product helicase-primase primase subunit 7744-7761 4:01:01 GGCGCTTGGAAGGCAGGG

7935 7969 6180 9422 CDS product helicase-primase primase subunit 7935-7969 5:02:01 GGTGACGAGGAATGGACACCCTGGGGTTTGTTGGG

7988 8003 6180 9422 CDS product helicase-primase primase subunit 7988-8003 4:01:01 CCAACCGCGGCCCGCC

8573 8590 6180 9422 CDS product helicase-primase primase subunit 8573-8590 4:01:01 GGGATTTGGTGCGGCCGG

8810 8832 6180 9422 CDS product helicase-primase primase subunit 8810-8832 5:02:01 GGAGGTTAAACCGGAGCGGCCGG

8885 8898 6180 9422 CDS product helicase-primase primase subunit 8885-8898 4:01:01 GGCAGCGGCGGCGG

9043 9063 6180 9422 CDS product helicase-primase primase subunit 9043-9063 4:01:01 CCAACGCCCGGGCTCCCGTCC

9123 9136 6180 9422 CDS product helicase-primase primase subunit 9123-9136 4:01:01 CCTCCCACCATACC

9221 9239 6180 9422 CDS product helicase-primase primase subunit 9221-9239 4:01:01 CCAACCGTCACCAGTGTCC

9506 9525 9421 10158 CDS product tegument protein UL51 9506-9525 4:01:01 CCCATCTACCATGCTCCGCC

9697 9719 9421 10158 CDS product tegument protein UL51 9697-9719 4:01:01 CCGACAACCCAAACCTAAACGCC

10040 10053 9421 10158 CDS product tegument protein UL51 10040-10053 4:01:01 CCCTCTCCTCCCCC

11992 12003 11676 12593 CDS product tegument protein VP22 11992-12003 4:01:01 GGTTGGTGGTGG

12473 12498 11676 12593 CDS product tegument protein VP22 12473-12498 4:01:01 GGATATGCGGCAGCTGGGCACAACGG

20103 20132 19564 20247 CDS product membrane protein UL45 20103-20132 7:04:01 GGTGGGTGTAGGGGGAGTTGGCAACGGGGG

23784 23799 23577 24797 CDS product DNA polymerase processivity subunit 23784-23799 4:01:01 GGCTGGGTCGGGTAGG
48100 48115 47924 49789 CDS product DNA packaging protein UL32 48100-48115 4:01:01 CCCACGCCACCGCCCC
48322 48339 47924 49789 CDS product DNA packaging protein UL32 48322-48339 4:01:01 CCACCATGTGCCGTGGCC
48550 48565 47924 49789 CDS product DNA packaging protein UL32 48550-48565 4:01:01 GGACCCGGTATGGCGG
48672 48695 47924 49789 CDS product DNA packaging protein UL32 48672-48695 4:01:01 GGCTGGGCTACACGGTAGACATGG
48708 48734 47924 49789 CDS product DNA packaging protein UL32 48708-48734 6:03:01
GGCAGATGGGGGCGGTGGAGTGTTTGG
49149 49168 47924 49789 CDS product DNA packaging protein UL32 49149-49168 4:01:01 GGACACGGCCACTGGGCCGG
49393 49407 47924 49789 CDS product DNA packaging protein UL32 49393-49407 5:02:01 GGGGGTGGCGGTGGG
49543 49565 47924 49789 CDS product DNA packaging protein UL32 49543-49565 5:02:01 CCCATAGGCCCCCCTCCAGAACC
50814 50837 50686 54348 CDS product DNA polymerase catalytic subunit 50814-50837 5:02:01
GGGGGTCTTGTGGGGGGTTTCCGG
50977 50995 50686 54348 CDS product DNA polymerase catalytic subunit 50977-50995 4:01:01
GGGTCTTCGGCGAGGTCGG
51036 51055 50686 54348 CDS product DNA polymerase catalytic subunit 51036-51055 4:01:01
GGTGGCCACGGGGTTTGGGG
51186 51201 50686 54348 CDS product DNA polymerase catalytic subunit 51186-51201 4:01:01 GGTGAGGTGTGGCAGG
51377 51396 50686 54348 CDS product DNA polymerase catalytic subunit 51377-51396 4:01:01
CCTCTGCGGCCGCCGTGGCC
51628 51645 50686 54348 CDS product DNA polymerase catalytic subunit 51628-51645 4:01:01
CCCAGCTCCGATACCCCC
51708 51729 50686 54348 CDS product DNA polymerase catalytic subunit 51708-51729 4:01:01
GGAGTAAGGCTTGTGGTTTTGG

51868 51890 50686 54348 CDS product DNA polymerase catalytic subunit 51868-51890 5:02:01 CCGTTTGCCACCCCCGTGAATCC
52244 52314 50686 54348 CDS product DNA polymerase catalytic subunit 52244-52314 12:09:03 CCTTTGCTCCCTGATAGCCCACGGCCCTTCCGGCGCCGGGCTTTCCGCCTCCATTTTCCGAAGCCATGACC
52639 52667 50686 54348 CDS product DNA polymerase catalytic subunit 52639-52667 5:02:01 CCTATTACGCCCCGCCGGTCGGGCCCGCC
53178 53205 50686 54348 CDS product DNA polymerase catalytic subunit 53178-53205 4:01:01 GGAGATCTGGATGACCAGGTCCTCTTGG
53301 53324 50686 54348 CDS product DNA polymerase catalytic subunit 53301-53324 4:01:01 GGGGATCGGCTCTAGGTTATCTGG
53396 53415 50686 54348 CDS product DNA polymerase catalytic subunit 53396-53415 4:01:01 GGGTGCCTGGCCGCAGGCGG
53531 53545 50686 54348 CDS product DNA polymerase catalytic subunit 53531-53545 4:01:01 GGCGGCTGCTGGAGG
53644 53672 50686 54348 CDS product DNA polymerase catalytic subunit 53644-53672 8:05:02 CCGCCCCACCATCCCCCGCCACTGCCGCC
53683 53706 50686 54348 CDS product DNA polymerase catalytic subunit 53683-53706 5:02:01 CCGCCGTTGCCATTTCCCAGGGCC
53995 54028 50686 54348 CDS product DNA polymerase catalytic subunit 53995-54028 6:03:01 CCATAGCCCCGAAAATCCTGGCCGTTCCAAACCC
54143 54154 50686 54348 CDS product DNA polymerase catalytic subunit 54143-54154 4:01:01 GGTAGGCGGGGG
54221 54250 50686 54348 CDS product DNA polymerase catalytic subunit 54221-54250 5:02:01 GGGGGGGCCTGGGGCGCTCGGTCTCTGCGG
58873 58910 58386 60713 CDS product DNA packaging terminase subunit 2 58873-58910 6:03:01

GGGGTTCGAGGCCGGCCTCGAGGCCTTGGACAGCGTGG
58933 58958 58386 60713 CDS product DNA packaging terminase subunit 2 58933-58958 4:01:01
GGGCAGCGGCATTGTGGTACCCCCGG
58969 58987 58386 60713 CDS product DNA packaging terminase subunit 2 58969-58987 4:01:01
CCCCACCCATCCCTGCTCC
59170 59194 58386 60713 CDS product DNA packaging terminase subunit 2 59170-59194 4:01:01
GGCTCTCCGGGCCCTCGAGGCAAGG
60989 61014 60872 63514 CDS product envelope glycoprotein B 60989-61014 7:04:01 CCTGCCCACCGCCTACCGGATCCACC
61369 61395 60872 63514 CDS product envelope glycoprotein B 61369-61395 5:02:01 CCAGGGGCCAGAGCCTGGCAGACCACC
61416 61434 60872 63514 CDS product envelope glycoprotein B 61416-61434 4:01:01 GGGGTGGATGCCATGGAGG
61470 61485 60872 63514 CDS product envelope glycoprotein B 61470-61485 5:02:01 GGAGGTGGAGGCGCGG
61505 61521 60872 63514 CDS product envelope glycoprotein B 61505-61521 4:01:01 CCTTCGCCCTGTCCACC
62060 62077 60872 63514 CDS product envelope glycoprotein B 62060-62077 4:01:01 CCAACCGCACCTACGACC
62144 62167 60872 63514 CDS product envelope glycoprotein B 62144-62167 5:02:01 CCATACCAGAACCTCAGCCAACCC
62220 62234 60872 63514 CDS product envelope glycoprotein B 62220-62234 4:01:01 GGAGGCAACGGAAGG
63015 63044 60872 63514 CDS product envelope glycoprotein B 63015-63044 5:02:01
GGGCCTGGGTAAAGTGGGGGAAGCAGTGGG
63354 63376 60872 63514 CDS product envelope glycoprotein B 63354-63376 4:01:01 GGTTTCGGCCCTGGAAAAGCAGG
69088 69105 69044 70102 CDS product thymidine kinase 69088-69105 4:01:01 GGGGCGCCGGCCAGGCGG
69359 69379 69044 70102 CDS product thymidine kinase 69359-69379 6:03:01 CCACCCCCTACCTTATCCTCC
69428 69444 69044 70102 CDS product thymidine kinase 69428-69444 4:01:01 CCAGACCAGACCTAACC
69456 69477 69044 70102 CDS product thymidine kinase 69456-69477 5:02:01 CCGCCACCCGGTCGCCTCTGCC
69536 69563 69044 70102 CDS product thymidine kinase 69536-69563 5:02:01 CCATGGTCGCCACCCTACCCAGGGAACC

69724 69751 69044 70102 CDS product thymidine kinase 69724-69751 5:02:01 GGGAGAGTCTGGCGCGACGGCTGGGGGG
70616 70633 70329 72884 CDS product envelope glycoprotein H 70616-70633 4:01:01 CCTCCGCAACACCAACCC
70649 70674 70329 72884 CDS product envelope glycoprotein H 70649-70674 4:01:01 CCAGAACCTGGGGACCCACGTGGACC
70892 70905 70329 72884 CDS product envelope glycoprotein H 70892-70905 4:01:01 GGTGAGGAAGGCGG
71286 71303 70329 72884 CDS product envelope glycoprotein H 71286-71303 4:01:01 GGATTTCCCGGGAGGTGG
71527 71546 70329 72884 CDS product envelope glycoprotein H 71527-71546 5:02:01 CCTCCGCCTTATTGTCCCCC
72639 72658 70329 72884 CDS product envelope glycoprotein H 72639-72658 4:01:01 CCATCCCGGCCTTTAATCCC
87321 87351 86725 87795 CDS product hypothetical protein 87321-87351 5:02:01
GGCTACTTGGTGAAAGGCGTCTGAGGAGCGG
87471 87489 86725 87795 CDS product hypothetical protein 87471-87489 4:01:01 GGCATCCACGGAGGAGGGG
87610 87641 86725 87795 CDS product hypothetical protein 87610-87641 6:03:01
GGAACAACGGTCTGGTGGCGCTGGGGAGTAGG
87770 87788 86725 87795 CDS product hypothetical protein 87770-87788 4:01:01 CCCTGCCCAGCACCCTACC
87834 87855 87824 88777 CDS product tegument protein UL14 87834-87855 4:01:01 GGCAAGGTCTAGGCGCCAAAGG
88084 88110 87824 88777 CDS product tegument protein UL14 88084-88110 4:01:01 GGGGAGATACTGGACAGGCATCGCAGG
88213 88232 87824 88777 CDS product tegument protein UL14 88213-88232 6:03:01 GGAGGCGGTGGTGAGGTCGG
88329 88361 87824 88777 CDS product tegument protein UL14 88329-88361 4:01:01
CCCTCATTCTCCCCGCTCAACCTGTCTCGCCCC
88650 88682 87824 88777 CDS product tegument protein UL14 88650-88682 7:04:01
GGTCGGCCCGGTCGATCAGGAATGGCTGGGTGG
88929 88953 88246 90030 CDS product tegument serine/threonine protein kinase 88929-88953 5:02:01
GGAGGCGCCGGTAGCTACGGGGAGG
89760 89782 88246 90030 CDS product tegument serine/threonine protein kinase 89760-89782 5:02:01

CCTGCCCTATTTCCTACCACCCC
89928 89942 88246 90030 CDS product tegument serine/threonine protein kinase 89928-89942 4:01:01 CCTTCCGCCTTTACC
91999 92015 91995 93347 CDS product envelope glycoprotein M 91999-92015 4:01:01 GGTACTCTCGGTGGTGG
92327 92343 91995 93347 CDS product envelope glycoprotein M 92327-92343 4:01:01 GGCTTTGGACGCGGTGG
92398 92418 91995 93347 CDS product envelope glycoprotein M 92398-92418 4:01:01 GGGTGGCTACGGCCAGCGTGG
92526 92550 91995 93347 CDS product envelope glycoprotein M 92526-92550 4:01:01 CCCACGGCAACCGTCACCGTTTGCC
92650 92669 91995 93347 CDS product envelope glycoprotein M 92650-92669 4:01:01 CCGCGCGTCCGGGTCCGGCC
92800 92816 91995 93347 CDS product envelope glycoprotein M 92800-92816 4:01:01 CCGCCCCCAAAACTGCC
96178 96213 95940 98195 CDS product helicase-primase subunit 96178-96213 5:02:01 GGCTGTGCCGGGGTGTTGGCCCCTGGCTCCCCTGGG
96242 96257 95940 98195 CDS product helicase-primase subunit 96242-96257 4:01:01 GGTGGCATAACGGCGG
96339 96352 95940 98195 CDS product helicase-primase subunit 96339-96352 4:01:01 CCCTCCGACCCGCC
96361 96378 95940 98195 CDS product helicase-primase subunit 96361-96378 6:03:01 GGATGGAGGCGGTGGCGG
96415 96441 95940 98195 CDS product helicase-primase subunit 96415-96441 5:02:01 GGTGGAGGTAGACTTGGACCCAGCGGG
96983 97002 95940 98195 CDS product helicase-primase subunit 96983-97002 4:01:01 GGGCTGTGGCCGGCTCTAGG
97024 97064 95940 98195 CDS product helicase-primase subunit 97024-97064 7:04:01 GGCTCCATGGGAACCGGAAGATAGGTGGCCCGGTGAATCGG
97074 97096 95940 98195 CDS product helicase-primase subunit 97074-97096 4:01:01 GGGTAGATGGGGTGGCACACAGG
97114 97136 95940 98195 CDS product helicase-primase subunit 97114-97136 6:03:01 GGATGGTTGGGGGGCCGGAAGGG
97342 97375 95940 98195 CDS product helicase-primase subunit 97342-97375 5:02:01 CCCCGTGGCCTATAGGTCCATAATAGCCCTCGCC

97448 97467 95940 98195 CDS product helicase-primase subunit 97448-97467 4:01:01 GGCTTTTGGGGGGCAGCTGG
97715 97736 95940 98195 CDS product helicase-primase subunit 97715-97736 4:01:01 GGGGCCGGCAGGGCCGCAAAGG
99628 99647 98997 101072 CDS product capsid portal protein 99628-99647 4:01:01 CCACGTAGCCGTCCTCTCCC
99670 99706 98997 101072 CDS product capsid portal protein 99670-99706 7:04:01
CCTCGTGCCCTAGTCCTCCTGCGCCGGCCTTGGCGCC
99749 99764 98997 101072 CDS product capsid portal protein 99749-99764 4:01:01 CCCGTCGGCCGCCGCC
99829 99860 98997 101072 CDS product capsid portal protein 99829-99860 5:02:01
CCAGAAGCCCGCTATTCCCGGCCCTCAGGTCC
99891 99913 98997 101072 CDS product capsid portal protein 99891-99913 4:01:01 CCCTCCAACATTCCGTTTATGCC
100495 100520 98997 101072 CDS product capsid portal protein 100495-100520 5:02:01
CCAGTTCCACCACTGCCTCAAGCCCC
100626 100647 98997 101072 CDS product capsid portal protein 100626-100647 5:02:01 GGGTGGACGGTTGCGCCGGGGG
100706 100737 98997 101072 CDS product capsid portal protein 100706-100737 5:02:01
GGCAAAGCTGGCGGTGCCGTCGGGCTCGCTGG
104139 104159 103937 104605 CDS product nuclear protein UL4 104139-104159 4:01:01 GGCGTTGGTGCTGGACTGCGG
104208 104220 103937 104605 CDS product nuclear protein UL4 104208-104220 4:01:01 CCCCCTGACCACC
104414 104433 103937 104605 CDS product nuclear protein UL4 104414-104433 4:01:01 CCGCGTGTTCCACCGCTGCC
104575 104595 103937 104605 CDS product nuclear protein UL4 104575-104595 4:01:01 CCGTGTTTCCCATGCCTTTCC
120061 120093 119988 120854 CDS product regulatory protein ICP22 120061-120093 7:04:01
CCCCATCATCCCGTCCCTGACCCCCTCCCCCCC
120103 120122 119988 120854 CDS product regulatory protein ICP22 120103-120122 4:01:01 CCCGTCCCCACGCTCCAGCC
120161 120186 119988 120854 CDS product regulatory protein ICP22 120161-120186 5:02:01
GGTGGCTCGGACCATCCGGAATACGG

120191  120235  119988  120854  CDS product regulatory protein ICP22  120191-120235  7:04:01
CCGCTTTCCCCGAGGGCCCTGCGCCCGTACCTGGCCCGGGGGCCC
120223  120239  119988  120854  CDS product regulatory protein ICP22  120223-120239  4:01:01 GGCCCGGGGGCCCGGGG
120315  120337  119988  120854  CDS product regulatory protein ICP22  120315-120337  5:02:01
CCACCTCCTCGATCCACGTGACC
120521  120537  119988  120854  CDS product regulatory protein ICP22  120521-120537  4:01:01 CCGCCGAGCGACCCTCC
120592  120614  119988  120854  CDS product regulatory protein ICP22  120592-120614  4:01:01
GGAGGAAGACGAGGCCAGCGTGG
120744  120790  119988  120854  CDS product regulatory protein ICP22  120744-120790  8:05:02
CCGATACGCCCACCAACAACCACCCTACAACCCGCGCCAGCGCTGCC
120799  120816  119988  120854  CDS product regulatory protein ICP22  120799-120816  4:01:01 CCGCAGGCGCCAGCCCCC
121459  121490  121409  122113  CDS product virion protein US10  121459-121490  6:03:01
GGCGAGGAGTCCGGAGCGGGGATGGGGACGGG
121590  121603  121409  122113  CDS product virion protein US10  121590-121603  4:01:01 GGCGGAGGCCGCGG
121762  121777  121409  122113  CDS product virion protein US10  121762-121777  4:01:01 GGCGCGGCGCCGGTGG
121990  122004  121409  122113  CDS product virion protein US10  121990-122004  4:01:01 CCCCGCCACGCCGCC
122016  122054  121409  122113  CDS product virion protein US10  122016-122054  6:03:01
CCTGCGCGCCCGTGGCCGCAAGCTCCCGCTCCAGCTTCC
122079  122093  121409  122113  CDS product virion protein US10  122079-122093  4:01:01 GGTGGCTGTGGCCGG
127658  127702  127508  129142  CDS product envelope glycoprotein J  127658-127702  9:06:02
CCCCCACCACAACCCACACATCCTCCCCATCTCCAACCTCTACCC
127760  127779  127508  129142  CDS product envelope glycoprotein J  127760-127779  4:01:01 CCAGCACCACCTCTTCTCCC
127793  127808  127508  129142  CDS product envelope glycoprotein J  127793-127808  4:01:01 CCACCACAACCACTCC

127826  127869  127508  129142  CDS product envelope glycoprotein J  127826-127869  8:05:02
CCTCCCCAACACCTACCGCCGCACCCACAACAGCCGCTACAACC
127982  128001  127508  129142  CDS product envelope glycoprotein J  127982-128001  4:01:01 CCACCACTGTTCCAACAACC
128174  128346  127508  129142  CDS product envelope glycoprotein J  128174-128346  34:31:08
CCTCTGCAACCACCGCGGCTACCACCACCGCGGCTACCACCACCGCGGCTACCACCACCGCGGCTACCACCACCGCGGCTACCACCACCGCGGCTACCACCACCGCGGCTACCACCACCGCGGCTACC
ACCACCGCGGCTACCACCACCGCGGCTACCACCACCGCGGCTACC
128393  128436  127508  129142  CDS product envelope glycoprotein J  128393-128436  9:06:02
CCACGTCCACCCCCTCAGCTTCCACTGCCACACCTGCCACTCCC
128789  128808  127508  129142  CDS product envelope glycoprotein J  128789-128808  4:01:01 CCGACTCTTCCACCGTGCCC
128927  128963  127508  129142  CDS product envelope glycoprotein J  128927-128963  6:03:01
CCGGAAATACCACCCCATCACCTTCCCCGCGTACCCC
129030  129042  127508  129142  CDS product envelope glycoprotein J  129030-129042  4:01:01 CCACACCACCGCC
129073  129095  127508  129142  CDS product envelope glycoprotein J  129073-129095  4:01:01 GGTCGCGCAGGGGGTCGTCGGGG
133255  133291  132878  134530  CDS product envelope glycoprotein E  133255-133291  6:03:01
CCAATCCTTGCCGAGACCCGCAGTGTCCTACAGATCC
133387  133406  132878  134530  CDS product envelope glycoprotein E  133387-133406  4:01:01 CCGCCCAAACAACCGCAACC
133424  133445  132878  134530  CDS product envelope glycoprotein E  133424-133445  5:02:01 CCCCGCCACCCGTAACCGTTCC
133653  133679  132878  134530  CDS product envelope glycoprotein E  133653-133679  6:03:01
CCCTACCGCCATGGCCTGCCTGCACCC
133909  133926  132878  134530  CDS product envelope glycoprotein E  133909-133926  4:01:01 GGACATCCGGAGGCGTGG
134331  134371  132878  134530  CDS product envelope glycoprotein E  134331-134371  9:06:02
CCCACCACCTCCCAAACCAGCTCCACAGCTACCACCCTACC
134401  134424  132878  134530  CDS product envelope glycoprotein E  134401-134424  4:01:01 GGCAGATCCGGATTCAAGGTTTGG

139250  139267  139214  140080  CDS product regulatory protein ICP22  139250-139267  4:01:01 GGGGGCTGGCGCCTGCGG

139276  139322  139214  140080  CDS product regulatory protein ICP22  139276-139322  8:05:02 GGCAGCGCTGGCGCGGGTTGTAGGGTGGTTGTTGGTGGGCGTATCGG

139452  139474  139214  140080  CDS product regulatory protein ICP22  139452-139474  4:01:01 CCACGCTGGCCTCGTCTTCCTCC

139529  139545  139214  140080  CDS product regulatory protein ICP22  139529-139545  4:01:01 GGAGGGTCGCTCGGCGG

139729  139751  139214  140080  CDS product regulatory protein ICP22  139729-139751  5:02:01 GGTCACGTGGATCGAGGAGGTGG

139827  139843  139214  140080  CDS product regulatory protein ICP22  139827-139843  4:01:01 CCCCGGGCCCCCGGGCC

139831  139875  139214  140080  CDS product regulatory protein ICP22  139831-139875  7:04:01 GGGCCCCCGGGCCAGGTACGGGCGCAGGGCCCTCGGGGAAAGCGG

139880  139905  139214  140080  CDS product regulatory protein ICP22  139880-139905  5:02:01 CCGTATTCCGGATGGTCCGAGCCACC

139944  139963  139214  140080  CDS product regulatory protein ICP22  139944-139963  4:01:01 GGCTGGAGCGTGGGGACGGG

139973  140005  139214  140080  CDS product regulatory protein ICP22  139973-140005  7:04:01 GGGGGGGAGGGGGTCAGGGACGGGATGATGGGG

gi|675510705|ref|NC_024771.1| Equid herpesvirus 3 strain AR/2007/C3A, complete genome

190 221 146 766 CDS product membrane protein UL56  190-221 7:04:01 CCTCGCCGCGCTTCCTTCTCCGCCGCCATGCC

230 264 146 766 CDS product membrane protein UL56  230-264 8:05:02 CCGCCACCGCGCGCCAGCCTCTCCGCAGGCCCGCC

302 318 146 766 CDS product membrane protein UL56  302-318 4:01:01 CCTCCGGCCCTCTGGCC

387 408 146 766 CDS product membrane protein UL56  387-408 6:03:01 CCTGCCCTCCAGACCCCCGTCC

429 456 146 766 CDS product membrane protein UL56 429-456 5:02:01 GGCCGTCCTGGAGCGCGGCGTGGAGCGG
477 531 146 766 CDS product membrane protein UL56 477-531 12:09:03
CCTCCGCGTTCCGCCGCCATCCTACCTAGAGGCCATGCGCCTGGCGCCCCCGGCC
519 541 146 766 CDS product membrane protein UL56 519-541 4:01:01 GGCGCCCCCGGCCTACGAGGTGG
594 612 146 766 CDS product membrane protein UL56 594-612 4:01:01 CCCCAACCCCTACACCACC
644 660 146 766 CDS product membrane protein UL56 644-660 4:01:01 CCGGCTACCCCCACCCC
707 751 146 766 CDS product membrane protein UL56 707-751 6:03:01 CCCTCGTTATCCTTTTGTCCATTACCCTCAACCCCTGGGTCACCC
923 935 808 1500 CDS product membrane protein V1 923-935 4:01:01 CCCCCGCCCCGCC
944 958 808 1500 CDS product membrane protein V1 944-958 4:01:01 GGACGGGGCGGGGGG
1049 1072 808 1500 CDS product membrane protein V1 1049-1072 5:02:01 GGGGGAGCACGGCGGGGCTCCCGG
1068 1083 808 1500 CDS product membrane protein V1 1068-1083 4:01:01 CCCGGCCTCCACGCCC
1099 1116 808 1500 CDS product membrane protein V1 1099-1116 4:01:01 CCCGCTGCCCCCGAGGCC
1191 1209 808 1500 CDS product membrane protein V1 1191-1209 4:01:01 GGGGCTGGTTCTGGCGGGG
1218 1234 808 1500 CDS product membrane protein V1 1218-1234 4:01:01 GGCCGACGGGGGGAAGG
1235 1277 808 1500 CDS product membrane protein V1 1235-1277 8:05:02
CCGTTCCGCCCTCGTACCACACGCTCCGCGACCTCGCACCCCC
1301 1326 808 1500 CDS product membrane protein V1 1301-1326 6:03:01 CCATTCCTCCAATTGCCGGAACCGCC
1342 1365 808 1500 CDS product membrane protein V1 1342-1365 4:01:01 GGGCGCTGGCTCTGGGGCCGCGGG
1434 1491 808 1500 CDS product membrane protein V1 1434-1491 11:08:02
GGGCTCGGGTGGGAGGGGTTTGGGAGGAGAGGTGCGAGCGGCACACCGGCCCGGGCGG
1665 1711 1656 2435 CDS product myristylated tegument protein CIRC 1665-1711 8:05:02
CCGGCTCCAGCCCCGAGAAGCCAGCTCCCGCCCTCGAGGCCTTGGCC
1765 1783 1656 2435 CDS product myristylated tegument protein CIRC 1765-1783 4:01:01

GGGGGGCGACGAGGCGCGG

1932 1959 1656 2435 CDS product myristylated tegument protein CIRC 1932-1959 5:02:01 GGGTGTTCGGGTACGAGGAGGGCACTGG

2170 2196 1656 2435 CDS product myristylated tegument protein CIRC 2170-2196 6:03:01 CCACTCTCCCGCCCAATGCCGCCGCCC

2278 2294 1656 2435 CDS product myristylated tegument protein CIRC 2278-2294 4:01:01 CCTTCGCCCGCCCCACC

2370 2383 1656 2435 CDS product myristylated tegument protein CIRC 2370-2383 4:01:01 CCCCCTCCGAGCCC

2394 2422 1656 2435 CDS product myristylated tegument protein CIRC 2394-2422 6:03:01 CCCGCGCCCCCGGCTCCTCCGTTTCTTCC

2693 2715 2472 3059 CDS product nuclear protein UL55 2693-2715 4:01:01 GGGGAGGTCTTGCAGGATCCGGG

2883 2898 2472 3059 CDS product nuclear protein UL55 2883-2898 4:01:01 GGCTGGCTGGTAGTGG

3609 3632 3283 4662 CDS product multifunctional expression regulator 3609-3632 5:02:01 GGCCAGGTGGCACCGCAGGAAGGG

3752 3768 3283 4662 CDS product multifunctional expression regulator 3752-3768 4:01:01 CCGAGGCCAGCGCCTCC

3797 3816 3283 4662 CDS product multifunctional expression regulator 3797-3816 4:01:01 CCCGGAGGGCCCGCGCCGCC

3936 3955 3283 4662 CDS product multifunctional expression regulator 3936-3955 5:02:01 GGCGGCCCAGGGGGACGTGG

4047 4068 3283 4662 CDS product multifunctional expression regulator 4047-4068 4:01:01 CCGGCTCACCTCGGCCACGGCC

4059 4081 3283 4662 CDS product multifunctional expression regulator 4059-4081 5:02:01

GGCCACGGCCGCGGCAGCGGCGG

4129 4147 3283 4662 CDS product multifunctional expression regulator 4129-4147 4:01:01 CCGCGGCCGCGCAGCCTCC

4238 4254 3283 4662 CDS product multifunctional expression regulator 4238-4254 4:01:01 CCGCGGGCCGCCGGCCC

4298 4338 3283 4662 CDS product multifunctional expression regulator 4298-4338 6:03:01 CCGCGTCCCGAGAGCTCCTCTCTCGCCCCGCGCCAGGAGCC

4350 4376 3283 4662 CDS product multifunctional expression regulator 4350-4376 5:02:01 CCGCTGTCGCCGGTTGCCCCCGCGACC

4419 4435 3283 4662 CDS product multifunctional expression regulator 4419-4435 4:01:01 GGGGGCGGCCGCCGGGG

4455 4487 3283 4662 CDS product multifunctional expression regulator 4455-4487 8:05:02 GGCGGCGGCCGGAGCTGTGGCGGGAGCGGCCGG

4802 4825 4799 5836 CDS product envelope glycoprotein K 4802-4825 4:01:01 CCCGGAACCGGCAGCCTTTGGCCC

5022 5051 4799 5836 CDS product envelope glycoprotein K 5022-5051 5:02:01 GGATGGCCACGGCTTCCAGGGCTGTCATGG

5163 5188 4799 5836 CDS product envelope glycoprotein K 5163-5188 4:01:01 CCGCGTACTCCAGGCCCATGAGGACC

5175 5200 4799 5836 CDS product envelope glycoprotein K 5175-5200 4:01:01 GGCCCATGAGGACCGCGGCGCAGGGG

5393 5414 4799 5836 CDS product envelope glycoprotein K 5393-5414 5:02:01 CCCAGCAGCCGCCGCTCCTGCC

5683 5701 4799 5836 CDS product envelope glycoprotein K 5683-5701 4:01:01 GGCGGTCGTGGCATTGGGG

5736 5756 4799 5836 CDS product envelope glycoprotein K 5736-5756 4:01:01 GGTTAAGGCCGGAGCTGAGGG

5946 5969 5849 9106 CDS product helicase-primase primase subunit 5946-5969 4:01:01 GGCCGTGGGCGTCGGCCGCTATGG

5986 6007 5849 9106 CDS product helicase-primase primase subunit 5986-6007 4:01:01
GGCGTCCCGGGTGGAGCGCAGG
6036 6050 5849 9106 CDS product helicase-primase primase subunit 6036-6050 4:01:01 GGGGGCGGTATGTGG
6189 6214 5849 9106 CDS product helicase-primase primase subunit 6189-6214 5:02:01
CCGCCACAAACGCCTCTACCTCGTCC
6636 6664 5849 9106 CDS product helicase-primase primase subunit 6636-6664 5:02:01
CCAGGCCCCGCACGGCCTCGGTGCCCGCC
6666 6692 5849 9106 CDS product helicase-primase primase subunit 6666-6692 7:04:01
GGAGGTAGGGTGGCGGCACAGGCACGG
6722 6741 5849 9106 CDS product helicase-primase primase subunit 6722-6741 4:01:01
CCGCACGCCTCCGTGTCGCC
6810 6823 5849 9106 CDS product helicase-primase primase subunit 6810-6823 4:01:01 CCTCCCAGCCCGCC
6946 6976 5849 9106 CDS product helicase-primase primase subunit 6946-6976 6:03:01
GGTCGCGCGGGCGTTTCGGGCGGGGGGCGGG
7009 7027 5849 9106 CDS product helicase-primase primase subunit 7009-7027 4:01:01
CCCCCAGTTCACCTCGGCC
7053 7074 5849 9106 CDS product helicase-primase primase subunit 7053-7074 4:01:01
CCACGACTCCCAGCCGGAAGCC
7201 7217 5849 9106 CDS product helicase-primase primase subunit 7201-7217 4:01:01
GGGGGGGCAGGCCGAGG
7240 7256 5849 9106 CDS product helicase-primase primase subunit 7240-7256 4:01:01
GGCCTCCGGCGCGGCGG
7275 7311 5849 9106 CDS product helicase-primase primase subunit 7275-7311 6:03:01

GGTCGCGCCGGGCCAGGGCCGGCTCCCCGGCGTCGGG
7282 7302 5849 9106 CDS product helicase-primase primase subunit 7282-7302 4:01:01
CCGGGCCAGGGCCGGCTCCCC
7415 7444 5849 9106 CDS product helicase-primase primase subunit 7415-7444 5:02:01
GGGAGGGCCACGCGGTAGACGGGCACCGGG
7440 7464 5849 9106 CDS product helicase-primase primase subunit 7440-7464 4:01:01
CCGGGCCGCGAACGCCCGTCTTTCC
7501 7525 5849 9106 CDS product helicase-primase primase subunit 7501-7525 7:04:01
GGCGGCCGGCGGTGCGGCGGCCAGG
7554 7579 5849 9106 CDS product helicase-primase primase subunit 7554-7579 5:02:01
GGGGGGCGGCGCCAGGGGCCGCGGGG
7595 7615 5849 9106 CDS product helicase-primase primase subunit 7595-7615 4:01:01
GGCTGGGACCGCGGCGACTGG
7627 7642 5849 9106 CDS product helicase-primase primase subunit 7627-7642 4:01:01 GGCCGCGGACGGGCGG
7646 7659 5849 9106 CDS product helicase-primase primase subunit 7646-7659 4:01:01 CCGCCCGCCTGCCC
7692 7705 5849 9106 CDS product helicase-primase primase subunit 7692-7705 4:01:01 CCTCCACCCGGTCC
7750 7769 5849 9106 CDS product helicase-primase primase subunit 7750-7769 4:01:01
GGGCACGGCCAGAGCGGGGG
7780 7827 5849 9106 CDS product helicase-primase primase subunit 7780-7827 9:06:02
GGGGGGGTAGGTCCTGGCCTCGGCGTACAGGCGGCCCACGGCCGCCGG
7933 7943 5849 9106 CDS product helicase-primase primase subunit 7933-7943 4:01:01 GGAGGCGGTGG
8130 8144 5849 9106 CDS product helicase-primase primase subunit 8130-8144 4:01:01 CCTGCACCCGCCGCC
8156 8176 5849 9106 CDS product helicase-primase primase subunit 8156-8176 4:01:01

GGGAGGGCTAGGGCGCCGGGG

8251 8265 5849 9106 CDS product helicase-primase primase subunit 8251-8265 4:01:01 GGTGGGGTTGGGGGG

8349 8369 5849 9106 CDS product helicase-primase primase subunit 8349-8369 4:01:01
GGAAGAGGTAGTACCGGGTGG

8501 8536 5849 9106 CDS product helicase-primase primase subunit 8501-8536 7:04:01
CCCGAGCGCCCGCCCCGGCCGCCGCACACCGCGGCC

8571 8596 5849 9106 CDS product helicase-primase primase subunit 8571-8596 5:02:01
CCGAGGCCGTCCGCCCCGCGCGCGCC

8658 8687 5849 9106 CDS product helicase-primase primase subunit 8658-8687 4:01:01
CCTCTTCGTCCAGCGCGTCCAACAGCGCCC

8692 8711 5849 9106 CDS product helicase-primase primase subunit 8692-8711 4:01:01
GGACAGCGGCTGGCCGCGGG

8718 8739 5849 9106 CDS product helicase-primase primase subunit 8718-8739 4:01:01
CCGCGGCCAGCGCCCGCGCGCC

8802 8817 5849 9106 CDS product helicase-primase primase subunit 8802-8817 4:01:01 CCCCGCCCACCATACC

9190 9212 9105 9860 CDS product tegument protein UL51 9190-9212 6:03:01 CCCGGCCACCATGCTCCGCCTCC

9217 9230 9105 9860 CDS product tegument protein UL51 9217-9230 4:01:01 GGCGCTGGCGGCGG

9241 9259 9105 9860 CDS product tegument protein UL51 9241-9259 4:01:01 CCTGCCCGCGACCCTGACC

9294 9308 9105 9860 CDS product tegument protein UL51 9294-9308 4:01:01 GGCGGCTGGTGAAGG

9364 9379 9105 9860 CDS product tegument protein UL51 9364-9379 5:02:01 CCGCCACCAGACCTCC

9388 9419 9105 9860 CDS product tegument protein UL51 9388-9419 6:03:01
CCCCAGCCTAAACGCCGTGGTGGCCACCCACC

9614 9628 9105 9860 CDS product tegument protein UL51 9614-9628 4:01:01 CCGGTACCCGCCACC

9666 9684 9105 9860 CDS product tegument protein UL51 9666-9684 4:01:01 CCTCGCCAGTCCAGACCCC
9713 9734 9105 9860 CDS product tegument protein UL51 9713-9734 5:02:01 CCCACGAGCCCCCTCCTTCCCC
9743 9800 9105 9860 CDS product tegument protein UL51 9743-9800 12:09:03 CCCGCCGGCGAGCCTCCCACACCCCCCCAGCCCGCCCGTGCCCCCGTCAAGTCCAGCC
10110 10128 9977 10954 CDS product deoxyuridine triphosphatase 10110-10128 4:01:01 GGCCGTAGGTGGGAAAAGG
10145 10171 9977 10954 CDS product deoxyuridine triphosphatase 10145-10171 6:03:01 CCCCGCCCCAACCACCAGGCGTCCTCC
10626 10647 9977 10954 CDS product deoxyuridine triphosphatase 10626-10647 4:01:01 CCTTTACCGGTCCGCGATAGCC
11103 11119 10920 11228 CDS product envelope glycoprotein N 11103-11119 4:01:01 CCCCCGGCACGGCCGCC
11163 11182 10920 11228 CDS product envelope glycoprotein N 11163-11182 4:01:01 CCTGCCAGGCGTACCGGGCC
11555 11601 11473 12420 CDS product tegument protein VP22 11555-11601 8:05:02 CCGTCGCGGCCCCCGGCGGCACCAACCCGCGACTCCCGCCGACGGCC
11709 11724 11473 12420 CDS product tegument protein VP22 11709-11724 4:01:01 CCCAACCGCCAGCGCC
11811 11833 11473 12420 CDS product tegument protein VP22 11811-11833 4:01:01 GGTGTATACGGAGGGGCGAACGG
11889 11972 11473 12420 CDS product tegument protein VP22 11889-11972 14:11:03 CCTCCCCCAAAGCCTGCGCCGCAGGCCACGCGGCCGGCCACTCGCGCCAGCGCCGGCGGGGCCGTTCCCAAGAGACCCGGCGCC
12046 12070 11473 12420 CDS product tegument protein VP22 12046-12070 5:02:01 CCACCCCCAAAACGCCTAAGGCCCC
12311 12323 11473 12420 CDS product tegument protein VP22 12311-12323 4:01:01 GGTGGGCGGCGGG
12332 12347 11473 12420 CDS product tegument protein VP22 12332-12347 4:01:01 CCGCCGGCACCGGTCC
12355 12380 11473 12420 CDS product tegument protein VP22 12355-12380 4:01:01 GGCGCCGGTCCTCTAGGTGCGCCAGG
12669 12697 12567 13919 CDS product transactivating tegument protein VP16 12669-12697 5:02:01 CCGGCGAGTCCGTCTTTGCCGGCCCGGCC
12689 12706 12567 13919 CDS product transactivating tegument protein VP16 12689-12706 4:01:01

GGCCCGGCCAAGAGGAGG

12707 12746 12567 13919 CDS product transactivating tegument protein VP16 12707-12746 7:04:01 CCTGCGCTGCCGCCCCCCAAGGCGGCCAGCCCCTCGGCCC

12756 12786 12567 13919 CDS product transactivating tegument protein VP16 12756-12786 5:02:01 GGTTGCAGGGCGAGCTCGGGTTCCCGGAGGG

12778 12802 12567 13919 CDS product transactivating tegument protein VP16 12778-12802 5:02:01 CCCGGAGGGCCCCACCCTCCTCTCC

12991 13019 12567 13919 CDS product transactivating tegument protein VP16 12991-13019 5:02:01 GGCGCCGGTCGTCGAGGACGACCTGGAGG

13140 13163 12567 13919 CDS product transactivating tegument protein VP16 13140-13163 5:02:01 GGCGACAGGCGCGCGGCGGCGCGG

13456 13466 12567 13919 CDS product transactivating tegument protein VP16 13456-13466 4:01:01 CCACCGCCGCC

13507 13519 12567 13919 CDS product transactivating tegument protein VP16 13507-13519 4:01:01 GGAGGAGGGGCGG

13600 13634 12567 13919 CDS product transactivating tegument protein VP16 13600-13634 7:04:01 GGAGGCCTACTCGGAGCGGCACCCGGCGGCGCCGG

13621 13648 12567 13919 CDS product transactivating tegument protein VP16 13621-13648 4:01:01 CCCGGCGGCGCCGGCATGCCCGCTGGCC

13691 13715 12567 13919 CDS product transactivating tegument protein VP16 13691-13715 4:01:01 GGCACGACGGCCGAGGCCATGATGG

13741 13762 12567 13919 CDS product transactivating tegument protein VP16 13741-13762 4:01:01 CCCTGGAGACCCCGTTCCGCCC

13850 13883 12567 13919 CDS product transactivating tegument protein VP16 13850-13883 6:03:01

CCGGGGGCCCACGACCTGAGCTCCGACGCCACCC
14348 14363 14334 16991 CDS product tegument protein VP13/14 14348-14363 4:01:01 GGAGAGCGGGGGTGGG
14366 14384 14334 16991 CDS product tegument protein VP13/14 14366-14384 4:01:01 CCGATACGCCGGCCCCGCC
14376 14398 14334 16991 CDS product tegument protein VP13/14 14376-14398 4:01:01 GGCCCCGCCGGTCTGTGGAAGGG
14460 14509 14334 16991 CDS product tegument protein VP13/14 14460-14509 10:07:02
CCGTTGCGCCCCGTGAGCCCTCCTCTCCCCCCGGGCCGCCCCCCGGCGCC
14504 14529 14334 16991 CDS product tegument protein VP13/14 14504-14529 7:04:01
GGCGCCGGGGGGCGGCCCGGGGGGGG
14571 14608 14334 16991 CDS product tegument protein VP13/14 14571-14608 7:04:01
CCACGCCAGCAGCCCCCCCGACCCGGCCGCGCGGGCCC
14595 14615 14334 16991 CDS product tegument protein VP13/14 14595-14615 4:01:01 GGCCGCGCGGGCCCAGGCTGG
14664 14681 14334 16991 CDS product tegument protein VP13/14 14664-14681 4:01:01 CCCCTGCCCCCGTGCCCC
14743 14770 14334 16991 CDS product tegument protein VP13/14 14743-14770 6:03:01
GGAGGGGGAAACCAGGGCGGAGCATTGG
14787 14806 14334 16991 CDS product tegument protein VP13/14 14787-14806 4:01:01 GGCGCGGTTCTTACGGCGGG
14840 14854 14334 16991 CDS product tegument protein VP13/14 14840-14854 4:01:01 CCGCCCGGGCCATCC
14902 14921 14334 16991 CDS product tegument protein VP13/14 14902-14921 4:01:01 GGGGTTGGACGGGGACGAGG
14932 14946 14334 16991 CDS product tegument protein VP13/14 14932-14946 4:01:01 GGAGGAAGAGGACGG
14962 14993 14334 16991 CDS product tegument protein VP13/14 14962-14993 6:03:01
GGAAAACGGCGCGGGCGAGGAGGGCTTTGGGG
15002 15029 14334 16991 CDS product tegument protein VP13/14 15002-15029 5:02:01
GGCCCGCGGCCGAGCCGGCCTTGGCTGG
15004 15021 14334 16991 CDS product tegument protein VP13/14 15004-15021 4:01:01 CCCGCGGCCGAGCCGGCC

15042 15059 14334 16991 CDS product tegument protein VP13/14 15042-15059 4:01:01 GGCAAATGGAGGCCGCGG
15079 15098 14334 16991 CDS product tegument protein VP13/14 15079-15098 4:01:01 GGCCAGGGTGATGGCGCGGG
15197 15212 14334 16991 CDS product tegument protein VP13/14 15197-15212 4:01:01 GGCATGTGGGAGGGGG
15286 15316 14334 16991 CDS product tegument protein VP13/14 15286-15316 5:02:01
CCTGCTCACCGCCCCGGTGGCCATGAACCCC
15321 15335 14334 16991 CDS product tegument protein VP13/14 15321-15335 4:01:01 GGGAGGAGGCCGTGG
15340 15358 14334 16991 CDS product tegument protein VP13/14 15340-15358 4:01:01 CCACCGCGCCTCGTTTCCC
15686 15698 14334 16991 CDS product tegument protein VP13/14 15686-15698 4:01:01 GGGGGTCTGGCGG
15833 15850 14334 16991 CDS product tegument protein VP13/14 15833-15850 4:01:01 CCCCCCGAGCACCGCCCC
15862 15877 14334 16991 CDS product tegument protein VP13/14 15862-15877 4:01:01 GGTGGCCGCGGCCAGG
15986 16022 14334 16991 CDS product tegument protein VP13/14 15986-16022 6:03:01
GGCCCTCTGGTCTACTGGGCGGCGCTCAGGCGCGCGG
16024 16039 14334 16991 CDS product tegument protein VP13/14 16024-16039 4:01:01 CCGAGACCCGGCCACC
16101 16129 14334 16991 CDS product tegument protein VP13/14 16101-16129 5:02:01
CCCATACGACCAGCCCCGGCTTTACCGCC
16521 16550 14334 16991 CDS product tegument protein VP13/14 16521-16550 6:03:01
CCGGCCACCTCAACCTGCTCCTAAACTGCC
16571 16586 14334 16991 CDS product tegument protein VP13/14 16571-16586 4:01:01 GGGGGGCGCCGGGTGG
16715 16731 14334 16991 CDS product tegument protein VP13/14 16715-16731 4:01:01 CCGATGCCCGGCCCCCC
16829 16850 14334 16991 CDS product tegument protein VP13/14 16829-16850 4:01:01 GGTAACCCGGTTGACCTGGCGG
16915 16932 14334 16991 CDS product tegument protein VP13/14 16915-16932 4:01:01 GGTGCGGGCGCACGGGGG
16944 16969 14334 16991 CDS product tegument protein VP13/14 16944-16969 5:02:01
GGCGCGGCGGGCGGGGAAGTAGAGGG

16972 16986 14334 16991 CDS product tegument protein VP13/14 16972-16986 4:01:01 CCCGCCCCCGAGGCC
17135 17159 17121 19349 CDS product tegument protein VP11/12 17135-17159 4:01:01 CCGTGGCCCCACGTCTCCCGGACCC
17230 17258 17121 19349 CDS product tegument protein VP11/12 17230-17258 6:03:01 GGCGCTAAAGGAGGCCGCGGAGGGGCTGG
17278 17304 17121 19349 CDS product tegument protein VP11/12 17278-17304 5:02:01 GGCCGAGCGGACGGCGGCTCTGTCGGG
17335 17396 17121 19349 CDS product tegument protein VP11/12 17335-17396 10:07:02 GGTCACCTCGGCCGTCGCGGGGGACGTTTCGGGGGAGTACCGGAGGGAATACGAGGCCGCGG
17431 17467 17121 19349 CDS product tegument protein VP11/12 17431-17467 7:04:01 GGATGGGATCTGGCAGGTGGTGATCAGGAGCTACTGG
17772 17810 17121 19349 CDS product tegument protein VP11/12 17772-17810 6:03:01 GGTGGCTGCTGTGGTTTATGGACCTGACGGACGCGAGGG
17903 17933 17121 19349 CDS product tegument protein VP11/12 17903-17933 7:04:01 GGGCCCGGGGTCTCGGCCGGAACGGGGGCGG
18076 18104 17121 19349 CDS product tegument protein VP11/12 18076-18104 6:03:01 CCACCACCACCTGCAGTACCTCATCAACC
18166 18187 17121 19349 CDS product tegument protein VP11/12 18166-18187 4:01:01 GGCGCTGCGGGCCCAGGGCCGG
18229 18274 17121 19349 CDS product tegument protein VP11/12 18229-18274 7:04:01 GGCAAGCTGGGCCAACATGGAGCGGGGCGTTCCGGCCTGGTTCCGG
18361 18387 17121 19349 CDS product tegument protein VP11/12 18361-18387 6:03:01 GGAGGCCGGCGAGCGCGGCGAGGACGG
18388 18406 17121 19349 CDS product tegument protein VP11/12 18388-18406 5:02:01 CCTCTCCCGCCGCACCGCC

18418 18459 17121 19349 CDS product tegument protein VP11/12 18418-18459 8:05:02 CCCCTTCCGTGGCCCCCCGCAAACCCCGCTCCCGAATCCCCC

18724 18741 17121 19349 CDS product tegument protein VP11/12 18724-18741 4:01:01 GGCGGAGGGGTCCAGCGG

18820 18857 17121 19349 CDS product tegument protein VP11/12 18820-18857 8:05:02 GGGCCCGGACGAGGACAGGTTGGTTGACGGGGGGGCGG

19011 19031 17121 19349 CDS product tegument protein VP11/12 19011-19031 4:01:01 GGAGCGGGGCGGCCGTTGGGG

19096 19122 17121 19349 CDS product tegument protein VP11/12 19096-19122 5:02:01 GGGCGGCGCGAGGGCCGAGGCCCCGGG

19110 19135 17121 19349 CDS product tegument protein VP11/12 19110-19135 4:01:01 CCGAGGCCCCGGGCCCCGCGTACGCC

19144 19160 17121 19349 CDS product tegument protein VP11/12 19144-19160 4:01:01 CCCGCGCTCCCGCCACC

19206 19242 17121 19349 CDS product tegument protein VP11/12 19206-19242 7:04:01 CCCGCACCAAGGCCATAAACGCCTCCGGACCCCGGCC

19313 19334 17121 19349 CDS product tegument protein VP11/12 19313-19334 4:01:01 CCCGCGCGCCCGGAGTCCCACC

19552 19577 19494 20105 CDS product membrane protein UL45 19552-19577 5:02:01 GGGGGCTGAGTGGGCGCGCGGCCTGG

19731 19749 19494 20105 CDS product membrane protein UL45 19731-19749 4:01:01 GGGGCGGCCGGGAGCTCGG

19835 19853 19494 20105 CDS product membrane protein UL45 19835-19853 4:01:01 GGTGTGCGGTGGGCAGCGG

20017 20035 19494 20105 CDS product membrane protein UL45 20017-20035 4:01:01 CCTTATTACCCCCGCTCCC

20046 20060 19494 20105 CDS product membrane protein UL45 20046-20060 4:01:01 CCTATCCGCCGCTCC

20071 20093 19494 20105 CDS product membrane protein UL45 20071-20093 4:01:01 GGGGCGGCAGAGAGGCAGTGGGG

20440 20476 20391 21809 CDS product envelope glycoprotein C 20440-20476 6:03:01 CCAGCAGGCCGAGGGCCAGCACCCCCCCGACGATCCC

20591 20606 20391 21809 CDS product envelope glycoprotein C 20591-20606 4:01:01 CCTTTCGCCGCCCTCC

| | | | | | | | |
|---|---|---|---|---|---|---|---|
| 20854 | 20870 | 20391 | 21809 | CDS product envelope glycoprotein C | 20854-20870 | 4:01:01 | CCTTGTACCAGTCCACC |
| 20881 | 20895 | 20391 | 21809 | CDS product envelope glycoprotein C | 20881-20895 | 4:01:01 | GGTTGGGCGGGTAGG |
| 21014 | 21036 | 20391 | 21809 | CDS product envelope glycoprotein C | 21014-21036 | 5:02:01 | CCACCGAAGCCGCACCGAGCGCC |
| 21117 | 21138 | 20391 | 21809 | CDS product envelope glycoprotein C | 21117-21138 | 4:01:01 | GGGGGCATGTAGGCCCCGATGG |
| 21312 | 21338 | 20391 | 21809 | CDS product envelope glycoprotein C | 21312-21338 | 5:02:01 | CCGAGCGGCCCCGCGCCCCCCGTGTCC |
| 21590 | 21616 | 20391 | 21809 | CDS product envelope glycoprotein C | 21590-21616 | 4:01:01 | GGGGACCTTCGGTTTAAGGCCCTTTGG |
| 21609 | 21635 | 20391 | 21809 | CDS product envelope glycoprotein C | 21609-21635 | 5:02:01 | CCCTTTGGCCCGCGCCTAGTCCTAGCC |
| 21668 | 21689 | 20391 | 21809 | CDS product envelope glycoprotein C | 21668-21689 | 6:03:01 | GGCAGCGGTGGCCGGCGGGGGG |
| 21772 | 21791 | 20391 | 21809 | CDS product envelope glycoprotein C | 21772-21791 | 5:02:01 | GGAGCCCGGCGGCGGCCAGG |
| 22177 | 22205 | 22005 | 23237 | CDS product envelope protein UL43 | 22177-22205 | 5:02:01 | CCGCGCCAAACCCGACGGCCGACAGCACC |
| 22220 | 22239 | 22005 | 23237 | CDS product envelope protein UL43 | 22220-22239 | 5:02:01 | GGCGGCGGCGTGGAGCGCGG |
| 22248 | 22279 | 22005 | 23237 | CDS product envelope protein UL43 | 22248-22279 | 5:02:01 | GGGCCGGCGCGGCGCACGCGGACCACCAGGGG |
| 22303 | 22317 | 22005 | 23237 | CDS product envelope protein UL43 | 22303-22317 | 4:01:01 | GGCCGGCGGCCAGGG |
| 22455 | 22484 | 22005 | 23237 | CDS product envelope protein UL43 | 22455-22484 | 5:02:01 | CCGAGGCACCCCACGCGCTCCTCGGCCACC |
| 22540 | 22557 | 22005 | 23237 | CDS product envelope protein UL43 | 22540-22557 | 4:01:01 | GGCCGAGGTCGGTGAAGG |
| 22789 | 22809 | 22005 | 23237 | CDS product envelope protein UL43 | 22789-22809 | 4:01:01 | GGTGTATTTGGACGGCGCAGG |
| 22879 | 22918 | 22005 | 23237 | CDS product envelope protein UL43 | 22879-22918 | 7:04:01 | CCCAGGCCAGGATAGCCACCATGGCAGCCAGGGCCTGGCC |
| 22901 | 22925 | 22005 | 23237 | CDS product envelope protein UL43 | 22901-22925 | 4:01:01 | GGCAGCCAGGGCCTGGCCGAAGCGG |
| 22948 | 22967 | 22005 | 23237 | CDS product envelope protein UL43 | 22948-22967 | 4:01:01 | GGCGGGCCACGGAGACGCGG |

23076 23105 22005 23237 CDS product envelope protein UL43 23076-23105 5:02:01 CCCACACACGCCAGCGCCTTGGTGGCCGCC

23096 23116 22005 23237 CDS product envelope protein UL43 23096-23116 4:01:01 GGTGGCCGCCAGGGTGCAGGG

23614 23643 23412 24695 CDS product DNA polymerase processivity subunit 23614-23643 5:02:01 CCGCGAGGGCCGCATCTACCGCCGCGGCCC

23981 23999 23412 24695 CDS product DNA polymerase processivity subunit 23981-23999 4:01:01 GGGGAAGGAGGCCGTTCGG

24189 24209 23412 24695 CDS product DNA polymerase processivity subunit 24189-24209 4:01:01 GGCGCGGGCGCAGCGGGGGGG

24349 24380 23412 24695 CDS product DNA polymerase processivity subunit 24349-24380 5:02:01 CCAGCACGCCGCGGCGGCCGTCCACGTTGGCC

24428 24442 23412 24695 CDS product DNA polymerase processivity subunit 24428-24442 4:01:01 GGCGGCTTGGATGGG

24603 24637 23412 24695 CDS product DNA polymerase processivity subunit 24603-24637 8:05:02 CCGCCCTCCGGGTCCGCCGCTACCGGAGTCCAGCC

25242 25258 25185 26711 CDS product tegument host shut-off protein 25242-25258 4:01:01 CCATCACCACCCCGCCC

25470 25502 25185 26711 CDS product tegument host shut-off protein 25470-25502 6:03:01 CCAAGGCCATCGTCACCCAGACCATGAGCCGCC

25512 25543 25185 26711 CDS product tegument host shut-off protein 25512-25543 6:03:01 CCGGGCGCCTGCCCGGCGGCCCCTGCCTCGCC

25605 25623 25185 26711 CDS product tegument host shut-off protein 25605-25623 4:01:01 CCGTCCCGGGCGCCGCGCC

25800 25816 25185 26711 CDS product tegument host shut-off protein 25800-25816 4:01:01 CCAACCTGTACCACACC

25892 25913 25185 26711 CDS product tegument host shut-off protein 25892-25913 4:01:01 CCCCTGTTTCCCCCGACGCTCC

25981 26004 25185 26711 CDS product tegument host shut-off protein 25981-26004 5:02:01
CCACACAGACCTCCACCAGCAGCC
26198 26212 25185 26711 CDS product tegument host shut-off protein 26198-26212 4:01:01 CCGGCGCCCCCGCCC
26251 26282 25185 26711 CDS product tegument host shut-off protein 26251-26282 7:04:01
CCGCCCCGAGGACCGCCGCGCACCACGCCGCC
26291 26306 25185 26711 CDS product tegument host shut-off protein 26291-26306 4:01:01 CCCCCCGAGGACCGCC
26309 26327 25185 26711 CDS product tegument host shut-off protein 26309-26327 4:01:01 GGCGCGCGGCGGTCTTTGG
26329 26351 25185 26711 CDS product tegument host shut-off protein 26329-26351 4:01:01
CCCGACTACCCGCGTTCTCCACC
26361 26377 25185 26711 CDS product tegument host shut-off protein 26361-26377 4:01:01 CCTCCCGCTACCCGCCC
26565 26596 25185 26711 CDS product tegument host shut-off protein 26565-26596 5:02:01
CCCTGAAGAACCTCGTCCCGGACCCCGACACC
26626 26643 25185 26711 CDS product tegument host shut-off protein 26626-26643 4:01:01 CCACGTTCCCACCCCTCC
27000 27047 26920 27867 CDS product ribonucleotide reductase subunit 2 27000-27047 10:07:02
GGCAAGCGGGAAGCTGGGGGCGGGGGGCGGTGCGTGGAACATGGGGGG
27332 27358 26920 27867 CDS product ribonucleotide reductase subunit 2 27332-27358 6:03:01
GGTAGGCGATGGCGGCGAATGAGGAGG
27419 27447 26920 27867 CDS product ribonucleotide reductase subunit 2 27419-27447 7:04:01
CCCGCACCTTCGCCTCCAGCCACGCCACC
27452 27465 26920 27867 CDS product ribonucleotide reductase subunit 2 27452-27465 4:01:01 GGCGGATGGCCGGG
27477 27493 26920 27867 CDS product ribonucleotide reductase subunit 2 27477-27493 4:01:01
GGAGGCCACGTAGGCGG
27605 27639 26920 27867 CDS product ribonucleotide reductase subunit 2 27605-27639 5:02:01

GGTCGAAGAGGGCCGACAGGTCGCCAAGGTTCAGG

27700 27723 26920 27867 CDS product ribonucleotide reductase subunit 2 27700-27723 5:02:01 CCCTCCGAGAGCCGGGCCACGTCC

27825 27850 26920 27867 CDS product ribonucleotide reductase subunit 2 27825-27850 6:03:01 GGCGGGGGCCGCGGCGCTCTCGGGGG

28230 28247 27954 30317 CDS product ribonucleotide reductase subunit 1 28230-28247 4:01:01 GGGTGGCCGTCGGGGAGG

28251 28272 27954 30317 CDS product ribonucleotide reductase subunit 1 28251-28272 5:02:01 CCGAACGCCCCCCGCACCGACC

28323 28341 27954 30317 CDS product ribonucleotide reductase subunit 1 28323-28341 4:01:01 GGAAAGAGGTCGCGGATGG

28511 28545 27954 30317 CDS product ribonucleotide reductase subunit 1 28511-28545 7:04:01 GGCGGTGCGGAGCCGGCGCCAGGCGTTGCGGTTGG

28619 28637 27954 30317 CDS product ribonucleotide reductase subunit 1 28619-28637 4:01:01 GGGCGGCAGGCCGTTTTGG

28758 28782 27954 30317 CDS product ribonucleotide reductase subunit 1 28758-28782 4:01:01 CCCTGGACGCCTAGCCCCAGCGACC

28797 28814 27954 30317 CDS product ribonucleotide reductase subunit 1 28797-28814 4:01:01 CCGCGCTCCGACCGGGCC

28903 28918 27954 30317 CDS product ribonucleotide reductase subunit 1 28903-28918 4:01:01 CCCCCCCGGCCGTCCC

28910 28949 27954 30317 CDS product ribonucleotide reductase subunit 1 28910-28949 7:04:01 GGCCGTCCCGGGCGGCGGGACCGCGAGGCACCGGGGCAGG

29025 29042 27954 30317 CDS product ribonucleotide reductase subunit 1 29025-29042 4:01:01

CCCGTCAGGGCCTCCTCC
29265 29295 27954 30317 CDS product ribonucleotide reductase subunit 1 29265-29295 5:02:01 CCGGGCTCGCCGTCCAGGTGCCGCTGGTACC
29585 29621 27954 30317 CDS product ribonucleotide reductase subunit 1 29585-29621 7:04:01 CCCCACCTCCTCCATGAGGGCCCGGACGGCCGAGTCC
29659 29683 27954 30317 CDS product ribonucleotide reductase subunit 1 29659-29683 5:02:01 CCAGAGACCCTCTTTTCCGGCCGCC
29716 29732 27954 30317 CDS product ribonucleotide reductase subunit 1 29716-29732 4:01:01 CCTGACCGGCCAGCGCC
29791 29807 27954 30317 CDS product ribonucleotide reductase subunit 1 29791-29807 4:01:01 CCATGGCCGGCTCCTCC
29822 29832 27954 30317 CDS product ribonucleotide reductase subunit 1 29822-29832 4:01:01 GGCGGTGGCGG
29852 29876 27954 30317 CDS product ribonucleotide reductase subunit 1 29852-29876 4:01:01 CCCCATGGACTCCAGGTGCCCGCCC
30037 30065 27954 30317 CDS product ribonucleotide reductase subunit 1 30037-30065 5:02:01 CCATCGACTCCAGCCACACCCGGAGGTCC
30178 30189 27954 30317 CDS product ribonucleotide reductase subunit 1 30178-30189 4:01:01 CCACCACCTCCC
30190 30223 27954 30317 CDS product ribonucleotide reductase subunit 1 30190-30223 7:04:01 GGCGGGATGGCCGCGGGGGCAGCGTGGCCCCCGG
30217 30244 27954 30317 CDS product ribonucleotide reductase subunit 1 30217-30244 6:03:01 CCCCCGGCCCGTCCTCGTACCCGTACCC
30706 30726 30693 32090 CDS product capsid triplex subunit 1 30706-30726 4:01:01 CCACCCAGCTTCCCGGCTCCC
30760 30781 30693 32090 CDS product capsid triplex subunit 1 30760-30781 4:01:01 CCACGTTGCCGAACCGCTCGCC

30860 30896 30693 32090 CDS product capsid triplex subunit 1 30860-30896 6:03:01 GGCTCGGCCGCGCAGGTCTGGGGTCCACTGGTACAGG

30973 31011 30693 32090 CDS product capsid triplex subunit 1 30973-31011 8:05:02 GGGGGAAGGCCGCGTTGGGGGCGGGGTCCGGCGCATCGG

31012 31024 30693 32090 CDS product capsid triplex subunit 1 31012-31024 4:01:01 CCCCCTCCGTACC

31069 31088 30693 32090 CDS product capsid triplex subunit 1 31069-31088 4:01:01 CCGCATCCTCGAAGGCCACC

31249 31269 30693 32090 CDS product capsid triplex subunit 1 31249-31269 5:02:01 GGGGGGCCCCGGTGCGGTCGG

31471 31490 30693 32090 CDS product capsid triplex subunit 1 31471-31490 4:01:01 CCCTGACGGCCTCGGCCGCC

31509 31526 30693 32090 CDS product capsid triplex subunit 1 31509-31526 4:01:01 CCGGCCCTGGCCGCGACC

31585 31605 30693 32090 CDS product capsid triplex subunit 1 31585-31605 4:01:01 GGTCAGCGAGGGCGGCCCAGG

31600 31619 30693 32090 CDS product capsid triplex subunit 1 31600-31619 5:02:01 CCCAGGCCTCCTCCAGCTCC

31635 31665 30693 32090 CDS product capsid triplex subunit 1 31635-31665 6:03:01 GGGGGCGTGCTGGCCGTGGCCATCCCGGTGG

31695 31721 30693 32090 CDS product capsid triplex subunit 1 31695-31721 4:01:01 GGGACCCCGGGGCGCTCGGCCTGCGGG

31744 31763 30693 32090 CDS product capsid triplex subunit 1 31744-31763 4:01:01 CCTGCCGGGTGAGCCGCACC

31806 31828 30693 32090 CDS product capsid triplex subunit 1 31806-31828 4:01:01 CCGATTGCCGCGCCCACGATCCC

31829 31848 30693 32090 CDS product capsid triplex subunit 1 31829-31848 4:01:01 GGAGGTTATCTGGATTCCGG

31917 31953 30693 32090 CDS product capsid triplex subunit 1 31917-31953 7:04:01 GGGGGGGCAAACGCGGTGGGGGTCGTTGGCCCCCTGG

31999 32020 30693 32090 CDS product capsid triplex subunit 1 31999-32020 4:01:01 CCCACCACGTCACCCCGGTGCC

32781 32802 32538 35609 CDS product tegument protein UL37 32781-32802 4:01:01 CCATGCTGGCCGAGAACCTGCC

32803 32821 32538 35609 CDS product tegument protein UL37 32803-32821 4:01:01 GGGGCTGGTGCTGTGGCGG

32994 33041 32538 35609 CDS product tegument protein UL37 32994-33041 8:05:02
CCTTTCGCCAGACCCTGGCGGCCGTGGCCGAGCGCACCCCCCGGGCCC
33136 33160 32538 35609 CDS product tegument protein UL37 33136-33160 5:02:01 GGAAGGCCTCCGGGTCGTGGCCCGG
33276 33297 32538 35609 CDS product tegument protein UL37 33276-33297 4:01:01 GGGCGGGCGCGGGCGCCCACGG
33414 33439 32538 35609 CDS product tegument protein UL37 33414-33439 4:01:01 CCTCGGCCCTAGCGCACCAGGACCCC
33525 33553 32538 35609 CDS product tegument protein UL37 33525-33553 6:03:01
CCGGGGCCCTGGCCATCCACGCCACGGCC
33645 33666 32538 35609 CDS product tegument protein UL37 33645-33666 5:02:01 CCATCCAGGCCAGCACCTCCCC
33673 33707 32538 35609 CDS product tegument protein UL37 33673-33707 7:04:01
GGTGGCGGACGCTCTGGTGGGCGCCGGCTTTACGG
33764 33799 32538 35609 CDS product tegument protein UL37 33764-33799 7:04:01
GGCTCCGGCGAGGGCGACGCGGGCGGGGAGGGCGGG
33817 33833 32538 35609 CDS product tegument protein UL37 33817-33833 4:01:01 GGCGGTGGGATGCGTGG
33867 33887 32538 35609 CDS product tegument protein UL37 33867-33887 4:01:01 GGTCCTACGGGTACGGCCTGG
33906 33934 32538 35609 CDS product tegument protein UL37 33906-33934 6:03:01
CCACCACCCTCTCAACCCTCGAGCCCGCC
33949 33980 32538 35609 CDS product tegument protein UL37 33949-33980 6:03:01
GGCGGCGCTGGGCCTGCCGGACAAGGGGCTGG
34309 34339 32538 35609 CDS product tegument protein UL37 34309-34339 6:03:01
CCCGTCCTTCGCCCCGTACCTGCTGGCCACC
34422 34443 32538 35609 CDS product tegument protein UL37 34422-34443 4:01:01 GGGCGCGGGACTACGGGACGGG
34524 34537 32538 35609 CDS product tegument protein UL37 34524-34537 4:01:01 CCCCCACGCCGACC
34584 34614 32538 35609 CDS product tegument protein UL37 34584-34614 6:03:01

CCGCCGTGGCCGGCGCCATCGCCCTCATCCC
34678 34706 32538 35609 CDS product tegument protein UL37 34678-34706 5:02:01
CCTCTTCCTGACGGCCGTCCACAAGCGCC
34816 34847 32538 35609 CDS product tegument protein UL37 34816-34847 5:02:01
GGCGGCCGTGCTGGGGCACCAGGCCGTGTCGG
34880 34917 32538 35609 CDS product tegument protein UL37 34880-34917 8:05:02
GGGACGTGGCGGCTGGTCGACCTGGTGGACGCGGTCGG
34995 35030 32538 35609 CDS product tegument protein UL37 34995-35030 5:02:01
GGGCCACTGAGGCCCTGCAGGAGTGCGAGGCGCTGG
35070 35087 32538 35609 CDS product tegument protein UL37 35070-35087 4:01:01 CCCCCCTCCTCGCGCGCC
35113 35134 32538 35609 CDS product tegument protein UL37 35113-35134 4:01:01 GGCGCTGGCCATCAAGGCGGGG
35143 35163 32538 35609 CDS product tegument protein UL37 35143-35163 4:01:01 GGGGGGCGCCGAGGCGCCCGG
35343 35360 32538 35609 CDS product tegument protein UL37 35343-35360 4:01:01 GGCGGGAGCTCGAGGCGG
35422 35455 32538 35609 CDS product tegument protein UL37 35422-35455 6:03:01
CCTCAGCCGCCGCGCCAACATCGCCTCGTGGGCC
35841 35867 35836 45897 CDS product large tegument protein 35841-35867 6:03:01 CCCAAGCCCTCGCCGCGCCCCCCCGCC
35868 35903 35836 45897 CDS product large tegument protein 35868-35903 7:04:01
GGGGGAAGCCGCGGGGCGGACGTGGTGGTCGTGGGG
36017 36063 35836 45897 CDS product large tegument protein 36017-36063 10:07:02
GGCAGACGCGGTGGACGGGTCGCTGGTGGAGGGCCAGGCGTGGACGG
36064 36079 35836 45897 CDS product large tegument protein 36064-36079 4:01:01 CCGCCAGCGTGCCCCC
36295 36324 35836 45897 CDS product large tegument protein 36295-36324 5:02:01
CCATAGGCGCCATCGGCCTCGCCGTGTACC

| | | | | | | |
|---|---|---|---|---|---|---|
| 36681 | 36699 | 35836 | 45897 | CDS product large tegument protein | 36681-36699 4:01:01 | GGGGCGCAGGGCGAGGAGG |
| 36823 | 36844 | 35836 | 45897 | CDS product large tegument protein | 36823-36844 4:01:01 | CCCTGCCCAAGCGCCGGCGCCC |
| 36875 | 36916 | 35836 | 45897 | CDS product large tegument protein | 36875-36916 7:04:01 | CCTGGCTGCCGTGGACGCCACCCACACGCCCGCCGGGCGCCC |
| 36971 | 37014 | 35836 | 45897 | CDS product large tegument protein | 36971-37014 8:05:02 | GGGGGAGGGCGCCGCGGGCGCGTGGGCCGAGGCGCTCGAGGAGG |
| 37037 | 37064 | 35836 | 45897 | CDS product large tegument protein | 37037-37064 5:02:01 | GGTCGAGGGCGACGGCGGAGAGCCAGGG |
| 37168 | 37187 | 35836 | 45897 | CDS product large tegument protein | 37168-37187 4:01:01 | CCGCCGAGGGCCTCGGCGCC |
| 37337 | 37357 | 35836 | 45897 | CDS product large tegument protein | 37337-37357 4:01:01 | GGCCTTTCTGGTGGAGAACGG |
| 37366 | 37388 | 35836 | 45897 | CDS product large tegument protein | 37366-37388 4:01:01 | CCCGCTCCGACGCCCCGTGCGCC |
| 37417 | 37449 | 35836 | 45897 | CDS product large tegument protein | 37417-37449 6:03:01 | CCGCCGTGCCCCACCCCACGGCGGCCGTGGACC |
| 37456 | 37485 | 35836 | 45897 | CDS product large tegument protein | 37456-37485 5:02:01 | GGGAGACGGGCATGGCCCTTGGCGACGTGG |
| 37638 | 37650 | 35836 | 45897 | CDS product large tegument protein | 37638-37650 4:01:01 | GGGGGTGGCGTGG |
| 37652 | 37674 | 35836 | 45897 | CDS product large tegument protein | 37652-37674 4:01:01 | CCCACTCGGCCTCTACACCCACC |
| 37679 | 37717 | 35836 | 45897 | CDS product large tegument protein | 37679-37717 9:06:02 | GGAGCGGCTGGTGGCCGCCATGGACCGGGCAGGCGGCGG |
| 37841 | 37862 | 35836 | 45897 | CDS product large tegument protein | 37841-37862 5:02:01 | CCTCCTCGCCCTGGAGGCCGCC |
| 37901 | 37931 | 35836 | 45897 | CDS product large tegument protein | 37901-37931 6:03:01 | CCAGCCAGCGGACCCCCCGGGGGCCGACGCC |
| 37934 | 37962 | 35836 | 45897 | CDS product large tegument protein | 37934-37962 6:03:01 | GGCGGAGCTGGCCAGGTCGCTGGACCTGG |

37958 37972 35836 45897 CDS product large tegument protein 37958-37972 4:01:01 CCTGGCCGCCGTGCC
37973 38017 35836 45897 CDS product large tegument protein 37973-38017 8:05:02
GGCGCGGCTGGCCAAGGTGGCAGAGAAGGCCGAGGCGCTCGTGGG
38062 38082 35836 45897 CDS product large tegument protein 38062-38082 4:01:01 CCCGAGCCATCGCCATGGACC
38091 38133 35836 45897 CDS product large tegument protein 38091-38133 7:04:01
GGTGCGCGGTTCCAGGTGGCCAGCGCGGCCGTGGCCAACCTGG
38102 38139 35836 45897 CDS product large tegument protein 38102-38139 6:03:01
CCAGGTGGCCAGCGCGGCCGTGGCCAACCTGGAGCGCC
38162 38187 35836 45897 CDS product large tegument protein 38162-38187 5:02:01 GGACCGGGCCGCGCGGGCTGCGGCGG
38188 38202 35836 45897 CDS product large tegument protein 38188-38202 4:01:01 CCGCCGCCGGCGTCC
38294 38310 35836 45897 CDS product large tegument protein 38294-38310 4:01:01 GGCGCTGGGGGCGTGGG
38339 38367 35836 45897 CDS product large tegument protein 38339-38367 5:02:01
GGGACACCTGGAGAAGCGGGAGCTGGAGG
38458 38474 35836 45897 CDS product large tegument protein 38458-38474 4:01:01 CCGCCGTCGACCAGGCC
38471 38496 35836 45897 CDS product large tegument protein 38471-38496 5:02:01 GGCCGCGGCGGACGCGTCGGACTCGG
38594 38616 35836 45897 CDS product large tegument protein 38594-38616 7:04:01 GGAGGTGGCGGCGCAGGTGGGGG
38644 38666 35836 45897 CDS product large tegument protein 38644-38666 4:01:01 CCCACGCCGCGCGCCAGCGGGCC
38777 38808 35836 45897 CDS product large tegument protein 38777-38808 5:02:01
GGCGCGGGACGCGCGGGTTGCCGCGGCCGAGG
38797 38819 35836 45897 CDS product large tegument protein 38797-38819 4:01:01 CCGCGGCCGAGGACCTGCGCGCC
38883 38904 35836 45897 CDS product large tegument protein 38883-38904 4:01:01 GGGCAGTATCGGGAGGCGCTGG
38909 38927 35836 45897 CDS product large tegument protein 38909-38927 4:01:01 CCCCAACGCCACCACGGCC
39050 39063 35836 45897 CDS product large tegument protein 39050-39063 5:02:01 GGGGGCGGTGGAGG

39091 39102 35836 45897 CDS product large tegument protein 39091-39102 4:01:01 GGGAGGCGGTGG
39191 39204 35836 45897 CDS product large tegument protein 39191-39204 4:01:01 CCAGGCCCGCCTCC
39256 39272 35836 45897 CDS product large tegument protein 39256-39272 4:01:01 CCGCCGTGTCGGCCGCC
39302 39315 35836 45897 CDS product large tegument protein 39302-39315 4:01:01 GGCGGCTCCGGGGG
39326 39345 35836 45897 CDS product large tegument protein 39326-39345 4:01:01 GGCGGACGCGCTGGACGGGG
39356 39390 35836 45897 CDS product large tegument protein 39356-39390 6:03:01
GGCGCGGCACGTGGCATCCGAGGTGACGGCCGTGG
39385 39398 35836 45897 CDS product large tegument protein 39385-39398 4:01:01 CCGTGGCCGCCGCC
39418 39439 35836 45897 CDS product large tegument protein 39418-39439 4:01:01 GGCTGGACGGGGACGCCTTGGG
39541 39561 35836 45897 CDS product large tegument protein 39541-39561 4:01:01 GGCAGCGGCGCGAGCGGGAGG
39742 39756 35836 45897 CDS product large tegument protein 39742-39756 4:01:01 CCGTCACCACCGTCC
39770 39800 35836 45897 CDS product large tegument protein 39770-39800 5:02:01
CCGCTTCAACCCCCACGCGCCCGAGAACGCC
39811 39828 35836 45897 CDS product large tegument protein 39811-39828 5:02:01 CCAACCCCACCATCCACC
39862 39884 35836 45897 CDS product large tegument protein 39862-39884 5:02:01 CCCTGGCCGCGCCGATCCTCGCC
39894 39918 35836 45897 CDS product large tegument protein 39894-39918 6:03:01 GGCGGCGTGGACGTGGAGGAGCTGG
40064 40095 35836 45897 CDS product large tegument protein 40064-40095 5:02:01
GGGGCACTTGGACTTCGAGGCCGAGATGGAGG
40127 40147 35836 45897 CDS product large tegument protein 40127-40147 4:01:01 GGCCTCGGGGGTCCGCGCGGG
40189 40229 35836 45897 CDS product large tegument protein 40189-40229 9:06:02
GGGCGGAGGAGGCCGAACGGGCGCTGGAGGCGGGCGTGCGG
40274 40310 35836 45897 CDS product large tegument protein 40274-40310 6:03:01
CCTGGAGGCCTTCGACCAGACCAGGTTCGCCGGGTCC

| | | | | | | | |
|---|---|---|---|---|---|---|---|
| 40324 | 40350 | 35836 | 45897 | CDS product large tegument protein | 40324-40350 | 6:03:01 | CCGCGGCCCTTGCCGTCCGCCAGGACC |
| 40387 | 40415 | 35836 | 45897 | CDS product large tegument protein | 40387-40415 | 7:04:01 | CCGTCCGCCGCGAGGCCACCGACCGGGCC |
| 40433 | 40443 | 35836 | 45897 | CDS product large tegument protein | 40433-40443 | 4:01:01 | GGTGGTGGAGG |
| 40478 | 40497 | 35836 | 45897 | CDS product large tegument protein | 40478-40497 | 4:01:01 | CCTAGCCAACCTCAAGAACC |
| 40547 | 40563 | 35836 | 45897 | CDS product large tegument protein | 40547-40563 | 4:01:01 | GGCGGCCTCGGCCGAGG |
| 40628 | 40640 | 35836 | 45897 | CDS product large tegument protein | 40628-40640 | 4:01:01 | GGCGGTGGAGTGG |
| 41231 | 41261 | 35836 | 45897 | CDS product large tegument protein | 41231-41261 | 7:04:01 | GGCGATGGGCTCGGCGGCGTCGGGCGGCCGG |
| 41258 | 41276 | 35836 | 45897 | CDS product large tegument protein | 41258-41276 | 4:01:01 | CCGGCCCCACCTCGAGGCC |
| 41342 | 41355 | 35836 | 45897 | CDS product large tegument protein | 41342-41355 | 4:01:01 | GGGGGACAAGGAGG |
| 41493 | 41516 | 35836 | 45897 | CDS product large tegument protein | 41493-41516 | 4:01:01 | GGCCGAACAGGGCGGTCGATGCGG |
| 41597 | 41618 | 35836 | 45897 | CDS product large tegument protein | 41597-41618 | 5:02:01 | CCTTGGCCTGGACCGGGCCGCC |
| 41783 | 41804 | 35836 | 45897 | CDS product large tegument protein | 41783-41804 | 5:02:01 | GGCGGGCGGCTTCGCGGTCGGG |
| 41826 | 41842 | 35836 | 45897 | CDS product large tegument protein | 41826-41842 | 4:01:01 | GGCGGGAGCGGAAGTGG |
| 42033 | 42055 | 35836 | 45897 | CDS product large tegument protein | 42033-42055 | 4:01:01 | GGGCCCGCGGAGCCCGGGGCGGG |
| 42059 | 42112 | 35836 | 45897 | CDS product large tegument protein | 42059-42112 | 9:06:02 | CCCCTCTCGGTCCGCCACCGGCAGCGCCCCGGGCCGGTGGCGCCCGCTGCCGCC |
| 42209 | 42227 | 35836 | 45897 | CDS product large tegument protein | 42209-42227 | 4:01:01 | CCTCTGGCTCCACTCCCCC |
| 42228 | 42277 | 35836 | 45897 | CDS product large tegument protein | 42228-42277 | 7:04:01 | GGGGAGCGGGGCCGCGCGGCCGCGGCCAAAGTGGCCGCGGCCATCCCGGG |
| 42239 | 42283 | 35836 | 45897 | CDS product large tegument protein | 42239-42283 | 7:04:01 | CCGCGCGGCCGCGGCCAAAGTGGCCGCGGCCATCCCGGGCAACCC |

42448 42473 35836 45897 CDS product large tegument protein 42448-42473 5:02:01 CCGCGGCCGCCGAAACGGCCCCACCC
42713 42740 35836 45897 CDS product large tegument protein 42713-42740 5:02:01 CCCGGCCGTTTCCTCGGACCTCAGCTCC
42820 42861 35836 45897 CDS product large tegument protein 42820-42861 8:05:02
CCATGATAGCCTCCAAGCCGCTGGCCGACGCGCCCCCTTGCC
42907 42935 35836 45897 CDS product large tegument protein 42907-42935 7:04:01
CCCCGCCCGCCCCGCCGCCGGAGCCGACC
43063 43079 35836 45897 CDS product large tegument protein 43063-43079 4:01:01 GGGCAACGGCGGAGGGG
43134 43163 35836 45897 CDS product large tegument protein 43134-43163 7:04:01
CCGTACGCCACCATCTCGCCCCCGCCACCC
43179 43202 35836 45897 CDS product large tegument protein 43179-43202 5:02:01 CCGGGGCACCCACCGAGCGCCCCC
43236 43261 35836 45897 CDS product large tegument protein 43236-43261 5:02:01 GGCGGCGGCGAGCCGGGGCGAAACGG
43274 43295 35836 45897 CDS product large tegument protein 43274-43295 4:01:01 CCTCGACAACCAGCCGTATGCC
43345 43367 35836 45897 CDS product large tegument protein 43345-43367 5:02:01 GGCAGGAGTGGCTCGAGGACGGG
43440 43457 35836 45897 CDS product large tegument protein 43440-43457 4:01:01 CCGCCCAAGTCGCCACCC
43473 43529 35836 45897 CDS product large tegument protein 43473-43529 12:09:03
CCTCTCGCCGGGGCCCGGCCGCCGCCCAAGTCGCCGCCGCTGACCCCGGCCGCCGCC
43576 43601 35836 45897 CDS product large tegument protein 43576-43601 5:02:01 CCTTGCCCAACAAGCCCCCCGCGCCC
43611 43634 35836 45897 CDS product large tegument protein 43611-43634 6:03:01 CCCGCCGCCACCCCCGAGGCGCCC
43628 43652 35836 45897 CDS product large tegument protein 43628-43652 5:02:01 GGCGCCCTCGGTGGCGGGGCCGAGG
43657 43681 35836 45897 CDS product large tegument protein 43657-43681 6:03:01 CCGCCGCCCCAACAACGCCGCCCCC
43696 43730 35836 45897 CDS product large tegument protein 43696-43730 7:04:01
CCGCGGCCGCCCCCAAAACCCCGCCTGGAATACCC
43761 43793 35836 45897 CDS product large tegument protein 43761-43793 6:03:01

CCCGATGCCTCCCGGGCGCCGAGCGCCGGGCCC

43828 43851 35836 45897 CDS product large tegument protein 43828-43851 4:01:01 CCAAGACGCCCAGCGGGCCCGCCC

43891 43910 35836 45897 CDS product large tegument protein 43891-43910 5:02:01 CCTCCTCGGCCGCCACTGCC

43953 43987 35836 45897 CDS product large tegument protein 43953-43987 7:04:01 CCGGCCAGACTGCCTCCCGAGGCCAGCCCCAAGCC

44069 44122 35836 45897 CDS product large tegument protein 44069-44122 9:06:02 CCCGCTTCCCCCTCCGCCGTCAGCCCCGTCGCAGCCCGCGCCCGCGTCGTCCCC

44138 44171 35836 45897 CDS product large tegument protein 44138-44171 8:05:02 CCCGCTCCCCCCTCCGCCACCCCTGAAGCCGCCC

44186 44218 35836 45897 CDS product large tegument protein 44186-44218 7:04:01 CCCGCCCCCCGCGCCCAGTGCCGGCGCCCTCCC

44219 44271 35836 45897 CDS product large tegument protein 44219-44271 11:08:02 GGCGGCGTCTGGGGCCAAACGGCAGGACGGGCGGCTCCGGCGGTCCGGCGAGG

44277 44305 35836 45897 CDS product large tegument protein 44277-44305 6:03:01 CCTCCGTCCGGCCCACAATCCACTGCGCC

44397 44441 35836 45897 CDS product large tegument protein 44397-44441 8:05:02 CCGATACCCGAGTCCCCGACTGAGCCGGCGCCGCCCCCGCTCGCC

44458 44494 35836 45897 CDS product large tegument protein 44458-44494 6:03:01 CCGTCTCGGCCGCCGACGTTCCTATCCCGGGCTCGCC

44516 44552 35836 45897 CDS product large tegument protein 44516-44552 8:05:02 CCTCCACCCGGCCCGGATCCCCCTGCCAGACTCGCCC

44729 44747 35836 45897 CDS product large tegument protein 44729-44747 4:01:01 CCCTCCTCCGGGACGACCC

44766 44797 35836 45897 CDS product large tegument protein 44766-44797 6:03:01

CCTCAGCCTGGTAAACCGCCAGTTTCCGGTCC
44831 44893 35836 45897 CDS product large tegument protein 44831-44893 11:08:02 GGCAGCGGCTGATGGGCCGGCGAAGAAGGGTAAGGGTGGTGCTCCGGATAAGAAGGCGGCCGG
45038 45066 35836 45897 CDS product large tegument protein 45038-45066 6:03:01 GGAGGCCGCGGACGGCCCCGGCCGCGAGG
45083 45099 35836 45897 CDS product large tegument protein 45083-45099 5:02:01 GGAGGAGGAAGGAGGGG
45100 45129 35836 45897 CDS product large tegument protein 45100-45129 6:03:01 CCACCACCGCAACTGCCGCGCCCCGGCGCC
45182 45231 35836 45897 CDS product large tegument protein 45182-45231 11:08:02 CCCCCGAGAACCGCCCTCTCCGCCGCCTGCGTCGCCGCCTGCGCGCCCCC
45264 45285 35836 45897 CDS product large tegument protein 45264-45285 4:01:01 CCGCTGCTTCCGCAGCCCCTCC
45292 45316 35836 45897 CDS product large tegument protein 45292-45316 5:02:01 GGACGGCGCCGGCGCCGGGCGCCGG
45359 45378 35836 45897 CDS product large tegument protein 45359-45378 5:02:01 CCCGCCCGCGCCCCCGGCCC
45400 45436 35836 45897 CDS product large tegument protein 45400-45436 7:04:01 CCGAAGACCTGGAGGACCTTCCGCTGTCCCCCGAGCC
45652 45675 35836 45897 CDS product large tegument protein 45652-45675 5:02:01 GGCGGCCCTCGGCGGGCGAAGTGG
46314 46344 46036 46404 CDS product small capsid protein 46314-46344 7:04:01 GGTCGCCCGGATGGTGGTGGGGTCGGACGGG
46510 46527 46500 47330 CDS product nuclear egress membrane protein 46510-46527 5:02:01 GGCGGGGGAGAAGGGCGG
46568 46596 46500 47330 CDS product nuclear egress membrane protein 46568-46596 6:03:01 GGCCATCAGGCGCCACGGGCGGCGGGCGG
46708 46738 46500 47330 CDS product nuclear egress membrane protein 46708-46738 5:02:01 GGGGCCCGAGGTCCTCGGGGCGGTCGCCGGG

46912 46924 46500 47330 CDS product nuclear egress membrane protein 46912-46924 4:01:01 CCAGCCCGCCGCC
46964 46982 46500 47330 CDS product nuclear egress membrane protein 46964-46982 4:01:01 GGCCTGGGTCGACCGGAGG
47000 47022 46500 47330 CDS product nuclear egress membrane protein 47000-47022 4:01:01
GGACGTGATGGGCGCTGCGGCGG
47077 47101 46500 47330 CDS product nuclear egress membrane protein 47077-47101 4:01:01
GGATTCGGACGTACGGGTCGCACGG
47198 47213 46500 47330 CDS product nuclear egress membrane protein 47198-47213 5:02:01 GGGGAGGTGGCGGGGG
47544 47574 47542 47922 CDS product DNA packaging protein UL33 47544-47574 6:03:01
GGCATCGTGGAGGATCTGGTGCAGGTCGAGG
47603 47625 47542 47922 CDS product DNA packaging protein UL33 47603-47625 5:02:01 CCTTGCGCGCCTCCAGGGCCGCC
47807 47827 47542 47922 CDS product DNA packaging protein UL33 47807-47827 4:01:01 GGTCGCCGGGGCGGTACTTGG
47922 47959 47921 49786 CDS product DNA packaging protein UL32 47922-47959 9:06:02
GGCCTCTGGCGCGGGGGGCGGCGGGTGGGCGCCGGGGG
48088 48126 47921 49786 CDS product DNA packaging protein UL32 48088-48126 7:04:01
GGCGACGGGGAGGACGGGGACGCGGATGAAGCGGGCGGG
48152 48172 47921 49786 CDS product DNA packaging protein UL32 48152-48172 4:01:01 CCGTGGCCGACTCCCTGGCCC
48269 48290 47921 49786 CDS product DNA packaging protein UL32 48269-48290 4:01:01 CCAAGTGCCTGGCAGCCCCGCC
48466 48497 47921 49786 CDS product DNA packaging protein UL32 48466-48497 6:03:01
CCGCGAGGCCCGCCCGCCGGCGCCGCGGCGCC
48536 48564 47921 49786 CDS product DNA packaging protein UL32 48536-48564 5:02:01
CCAACTACTCCTTCCTGGTCCAGTCATCC
48603 48620 47921 49786 CDS product DNA packaging protein UL32 48603-48620 5:02:01 GGCGGAGGCGGCGTCGGG
48647 48664 47921 49786 CDS product DNA packaging protein UL32 48647-48664 4:01:01 GGGAGCAGGGCGGCGCGG

48665 48717 47921 49786 CDS product DNA packaging protein UL32 48665-48717 9:06:02
CCGCCGCTGGAGCCGCGCCTGGCCCAGCCCCGCGCCAGCAGTCCCTCGCGGCC
48753 48788 47921 49786 CDS product DNA packaging protein UL32 48753-48788 6:03:01
GGACTGCTCGGGCCCGGAGCTGCGGCGCGCCGGGGG
48855 48882 47921 49786 CDS product DNA packaging protein UL32 48855-48882 6:03:01
GGCCAAGGCCGAGGGCGGGGACGGGGGG
48894 48925 47921 49786 CDS product DNA packaging protein UL32 48894-48925 6:03:01
GGCGCGGCGGACATGGGGCTACGCGGACCTGG
48948 48972 47921 49786 CDS product DNA packaging protein UL32 48948-48972 5:02:01
GGCCACGTGGGAGGACGGAGAAAGG
49067 49101 47921 49786 CDS product DNA packaging protein UL32 49067-49101 6:03:01
CCCGGTTTGCCGAGCCCGACGCCGAGCCCGACGCC
49110 49128 47921 49786 CDS product DNA packaging protein UL32 49110-49128 4:01:01 CCCTGTCCTAGCGACCACC
49290 49345 47921 49786 CDS product DNA packaging protein UL32 49290-49345 11:08:02
GGAGGCGCTGGCCGCCGGCGAGGGCAGGGAAGGGGCTGAGGCGGCGCGGACCGAGG
49359 49375 47921 49786 CDS product DNA packaging protein UL32 49359-49375 4:01:01 GGATAGAGGGGGCGAGG
49386 49407 47921 49786 CDS product DNA packaging protein UL32 49386-49407 4:01:01 GGGCGATGGGCTCAGCGGCCGG
49449 49462 47921 49786 CDS product DNA packaging protein UL32 49449-49462 4:01:01 GGCTGGGACGGAGG
49512 49531 47921 49786 CDS product DNA packaging protein UL32 49512-49531 4:01:01 CCACACCAACCCCGGTGTCC
49540 49562 47921 49786 CDS product DNA packaging protein UL32 49540-49562 6:03:01 CCGCCGGGCCCGCCGCCGGAGCC
49674 49698 47921 49786 CDS product DNA packaging protein UL32 49674-49698 5:02:01
CCCCCCCAAGCCAACCGCAGGAGCC
49715 49745 47921 49786 CDS product DNA packaging protein UL32 49715-49745 6:03:01

CCGGCCTGCTGCTCCGGCGCCACAACCTCCC

49801 49828 49779 50774 CDS product nuclear egress lamina protein 49801-49828 6:03:01

CCGCCGGGCCACGCCCGCAGCCGACCCC

49924 49945 49779 50774 CDS product nuclear egress lamina protein 49924-49945 4:01:01

CCGCGCGCGCCTCGGCTCCGCC

50088 50131 49779 50774 CDS product nuclear egress lamina protein 50088-50131 8:05:02

CCGCGCCCGTGACCCTCCCCTTTGACCTCACCCAGACCGTGGCC

50988 51007 50698 54363 CDS product DNA polymerase catalytic subunit 50988-51007 4:01:01

GGGGTCCTCGGCCAGGTCGG

51048 51069 50698 54363 CDS product DNA polymerase catalytic subunit 51048-51069 6:03:01

GGAGGGGGGCTGGGGCGGGGGG

51386 51417 50698 54363 CDS product DNA polymerase catalytic subunit 51386-51417 6:03:01

CCGTCGCTTCCGCCGCGGCCGCGGCCACGGCC

51428 51447 50698 54363 CDS product DNA polymerase catalytic subunit 51428-51447 4:01:01

GGAGCAGGTCCACCAGGTGG

51883 51912 50698 54363 CDS product DNA polymerase catalytic subunit 51883-51912 5:02:01

CCGTTGGCCACGCCCGTAAAACCGTACACC

51923 51939 50698 54363 CDS product DNA polymerase catalytic subunit 51923-51939 4:01:01

CCACCTTGATGGCCGCC

51950 51966 50698 54363 CDS product DNA polymerase catalytic subunit 51950-51966 4:01:01

CCAGCAGCACCGCCTCC

51969 52000 50698 54363 CDS product DNA polymerase catalytic subunit 51969-52000 6:03:01

GGGGGTGCTGGAGGGAATGCGGGCCCGCACGG

52125 52152 50698 54363 CDS product DNA polymerase catalytic subunit 52125-52152 7:04:01 CCCGGCCACGTCCGCCGCCTCCGCGGCC
52211 52224 50698 54363 CDS product DNA polymerase catalytic subunit 52211-52224 4:01:01 CCAGCACCACCACC
52293 52306 50698 54363 CDS product DNA polymerase catalytic subunit 52293-52306 4:01:01 GGCCCGGGAGGCGG
52334 52352 50698 54363 CDS product DNA polymerase catalytic subunit 52334-52352 4:01:01 CCCCGCCGCCCTCGCTCCC
52364 52380 50698 54363 CDS product DNA polymerase catalytic subunit 52364-52380 4:01:01 CCGAACCCTCCCGCTCC
52389 52415 50698 54363 CDS product DNA polymerase catalytic subunit 52389-52415 5:02:01 GGCCTCGGCGTCGAGGTCCTCCGGCGG
52406 52450 50698 54363 CDS product DNA polymerase catalytic subunit 52406-52450 9:06:02 CCTCCGGCGGCGCCGCATCCTCGTCCGCCCCGCCAAACCGGCGCC
52445 52464 50698 54363 CDS product DNA polymerase catalytic subunit 52445-52464 4:01:01 GGCGCCGGTTGTCGGGGAGG
52556 52581 50698 54363 CDS product DNA polymerase catalytic subunit 52556-52581 5:02:01 CCAGCCTGGCCACGGCCGAGAGCTCC
52583 52593 50698 54363 CDS product DNA polymerase catalytic subunit 52583-52593 4:01:01 GGTGGGGGAGG
52612 52629 50698 54363 CDS product DNA polymerase catalytic subunit 52612-52629 4:01:01 CCCACCAGCCGGGAGTCC
52721 52749 50698 54363 CDS product DNA polymerase catalytic subunit 52721-52749 5:02:01 CCTTGAGCACCTCGCCCACCACGGCGTCC
52897 52919 50698 54363 CDS product DNA polymerase catalytic subunit 52897-52919 5:02:01 CCGTAGCCGTCCAGCCGGAGGCC

52980 52996 50698 54363 CDS product DNA polymerase catalytic subunit 52980-52996 4:01:01
GGTGGCGAACTCGGGGG
53009 53031 50698 54363 CDS product DNA polymerase catalytic subunit 53009-53031 5:02:01
GGAAGGTGAGGAAGGCGAGAAGG
53358 53378 50698 54363 CDS product DNA polymerase catalytic subunit 53358-53378 4:01:01
GGTCGCGTGGCGGTCAACCGG
53403 53421 50698 54363 CDS product DNA polymerase catalytic subunit 53403-53421 4:01:01
GGGGCCGGGGCGCAGGCGG
53650 53681 50698 54363 CDS product DNA polymerase catalytic subunit 53650-53681 9:06:02
CCGCCCCACCAGCCCCCGCCGCCCCCCTCGCC
53684 53706 50698 54363 CDS product DNA polymerase catalytic subunit 53684-53706 6:03:01
GGCGATCGGCGGGGGTGGAGGGG
53954 53967 50698 54363 CDS product DNA polymerase catalytic subunit 53954-53967 4:01:01 CCACCGTCTCCACC
54013 54027 50698 54363 CDS product DNA polymerase catalytic subunit 54013-54027 4:01:01 GGCCGGAAGGCCCGG
54015 54034 50698 54363 CDS product DNA polymerase catalytic subunit 54015-54034 4:01:01
CCGGAAGGCCCGGCCGTTCC
54044 54066 50698 54363 CDS product DNA polymerase catalytic subunit 54044-54066 4:01:01
CCCGGCGGGGCCAGCAGCCCTCC
54149 54169 50698 54363 CDS product DNA polymerase catalytic subunit 54149-54169 7:04:01
GGCGGCGGTCGGTGGGGGCGG
54225 54246 50698 54363 CDS product DNA polymerase catalytic subunit 54225-54246 5:02:01
GGAGTGGGGCGGGGGGCGCGGG
54690 54725 54684 58328 CDS product single-stranded DNA-binding protein 54690-54725 7:04:01

CCGCACCCAAAACCGTCAGCCTCCCAGCGGCCCCCC
54895 54913 54684 58328 CDS product single-stranded DNA-binding protein 54895-54913 4:01:01 GGGACTCGGCGGCAGCGGG
54932 54948 54684 58328 CDS product single-stranded DNA-binding protein 54932-54948 4:01:01 CCCAGCCACTACCACCC
54975 54988 54684 58328 CDS product single-stranded DNA-binding protein 54975-54988 4:01:01 CCGCCATCCGAGCC
54999 55030 54684 58328 CDS product single-stranded DNA-binding protein 54999-55030 7:04:01 CCCCCAACCTATCCCGGGCCTGCGACGCCGCC
55032 55052 54684 58328 CDS product single-stranded DNA-binding protein 55032-55052 4:01:01 GGAAGCGGTTTGGCTTCTCGG
55208 55223 54684 58328 CDS product single-stranded DNA-binding protein 55208-55223 4:01:01 GGCGGCGCGGGCACGG
55310 55333 54684 58328 CDS product single-stranded DNA-binding protein 55310-55333 4:01:01 CCCTTCAACGCCAAGCACCGCTCC
55552 55601 54684 58328 CDS product single-stranded DNA-binding protein 55552-55601 9:06:02 GGGCAGGGGCGGCGGCCGCGGGCCCCAGCGGGGCGGGGACGGCACCTCGG
55596 55615 54684 58328 CDS product single-stranded DNA-binding protein 55596-55615 4:01:01 CCTCGGCCAAGAGCACCCCC
55622 55664 54684 58328 CDS product single-stranded DNA-binding protein 55622-55664 9:06:02 GGGACGGAGCGGCGCCTGGCCTCGGTGATGGCGGCGGACACGG
55687 55697 54684 58328 CDS product single-stranded DNA-binding protein 55687-55697 4:01:01 GGGGGCGGGGG
55768 55781 54684 58328 CDS product single-stranded DNA-binding protein 55768-55781 4:01:01 GGAGGCGCTGGGGG
55794 55820 54684 58328 CDS product single-stranded DNA-binding protein 55794-55820 5:02:01 GGGTCTCGGGCCTGGTAGGGGCCATGG

55852 55869 54684 58328 CDS product single-stranded DNA-binding protein 55852-55869 5:02:01 GGAGGTGGACGACGGCGG
55897 55919 54684 58328 CDS product single-stranded DNA-binding protein 55897-55919 4:01:01 CCCCTCATACCACCGCTTCTACC
56116 56130 54684 58328 CDS product single-stranded DNA-binding protein 56116-56130 4:01:01 GGGGGCCTTTGGCGG
56154 56183 54684 58328 CDS product single-stranded DNA-binding protein 56154-56183 6:03:01 GGTATCTGGCCGGTACGCTGGAGTCGGAGG
56186 56218 54684 58328 CDS product single-stranded DNA-binding protein 56186-56218 5:02:01 CCCTGCGGCCTTTGCGACCGAACCACGCGCCCC
56242 56252 54684 58328 CDS product single-stranded DNA-binding protein 56242-56252 4:01:01 CCGCCTCCGCC
56452 56471 54684 58328 CDS product single-stranded DNA-binding protein 56452-56471 5:02:01 GGAGCAGGCCAGGCTGGTGG
56491 56517 54684 58328 CDS product single-stranded DNA-binding protein 56491-56517 5:02:01 GGCCGGCGTCGGCCCCGGCGCAGCGGG
56566 56585 54684 58328 CDS product single-stranded DNA-binding protein 56566-56585 4:01:01 GGAGACGGTGGAGCAAGCGG
56828 56849 54684 58328 CDS product single-stranded DNA-binding protein 56828-56849 5:02:01 CCAGTGCTCCGCCGCCGCTTCC
56878 56921 54684 58328 CDS product single-stranded DNA-binding protein 56878-56921 9:06:02 GGCCAAGACGGTGACGGTGACGGTGGCCGAGTCGGGGGTGGTGG
56924 56959 54684 58328 CDS product single-stranded DNA-binding protein 56924-56959 7:04:01 CCTGACCTGACGCTCCCCGCCACGGAGCCCCCCACC
57061 57092 54684 58328 CDS product single-stranded DNA-binding protein 57061-57092 7:04:01

GGAGGCGGCCAAGGCGCGCGTGGCCGGGATGG

57084 57112 54684 58328 CDS product single-stranded DNA-binding protein 57084-57112 6:03:01

CCGGGATGGCCAGCGCCTACCGCCGCCCC

57181 57198 54684 58328 CDS product single-stranded DNA-binding protein 57181-57198 4:01:01

CCCCAAGGGCCACCCTCC

57437 57459 54684 58328 CDS product single-stranded DNA-binding protein 57437-57459 4:01:01

GGCCTCACGGCCATTGTGGTGGG

57462 57484 54684 58328 CDS product single-stranded DNA-binding protein 57462-57484 4:01:01

CCAAACGGCCCCGCGACCCGGCC

57481 57508 54684 58328 CDS product single-stranded DNA-binding protein 57481-57508 5:02:01

GGCCTCGGTGCTGGCCTGGATCGACAGG

57517 57539 54684 58328 CDS product single-stranded DNA-binding protein 57517-57539 5:02:01

GGGTCAGGCGGACGTGGAGCCGG

57706 57735 54684 58328 CDS product single-stranded DNA-binding protein 57706-57735 6:03:01

GGCGGGCAACTGGAACGGCCTGAACGGCGG

57817 57845 54684 58328 CDS product single-stranded DNA-binding protein 57817-57845 6:03:01

GGCCGGCGGCTTTGGCGCCGGCGTGCGGG

57865 57891 54684 58328 CDS product single-stranded DNA-binding protein 57865-57891 6:03:01

GGTGCGTGGCATAGTGGCGGAGGGCGG

57940 57961 54684 58328 CDS product single-stranded DNA-binding protein 57940-57961 5:02:01

CCCCCGCACCCAGCACCTGGCC

57972 57989 54684 58328 CDS product single-stranded DNA-binding protein 57972-57989 5:02:01

GGGTCGGGCTGGTGGAGG

58002 58027 54684 58328 CDS product single-stranded DNA-binding protein 58002-58027 4:01:01
CCGTCAGCCTGGATGCCATCAACGCC
58046 58079 54684 58328 CDS product single-stranded DNA-binding protein 58046-58079 7:04:01
GGCGAGTGGACCGTGGAGGCGGCGCAGGAGCTGG
58171 58192 54684 58328 CDS product single-stranded DNA-binding protein 58171-58192 5:02:01
GGCGGCCGGACCCGGGTTTGGG
58223 58240 54684 58328 CDS product single-stranded DNA-binding protein 58223-58240 4:01:01
GGCGTCGGCGGCGCCGGG
58445 58485 58444 60738 CDS product DNA packaging terminase subunit 2 58445-58485 8:05:02
GGAGCGGGAGGAGGGCGCCGGAACGATCGGGAGGGCGCAGG
58502 58524 58444 60738 CDS product DNA packaging terminase subunit 2 58502-58524 5:02:01
GGCGGTCTTCGGGCAGGTGCAGG
58627 58642 58444 60738 CDS product DNA packaging terminase subunit 2 58627-58642 4:01:01 GGCGGAAGCTGGCGGG
58664 58685 58444 60738 CDS product DNA packaging terminase subunit 2 58664-58685 4:01:01
CCGACACCAGACGCCGCTCGCC
58702 58721 58444 60738 CDS product DNA packaging terminase subunit 2 58702-58721 4:01:01
CCATGGCCTACGCCGAGGCC
58718 58743 58444 60738 CDS product DNA packaging terminase subunit 2 58718-58743 5:02:01
GGCCGAGGGGGAGCGGGTGCTCAAGG
58988 59007 58444 60738 CDS product DNA packaging terminase subunit 2 58988-59007 4:01:01
GGCGGGAAGCGGCCTCGTGG
59072 59110 58444 60738 CDS product DNA packaging terminase subunit 2 59072-59110 8:05:02
GGCCAACCAGGGGGAGGCGGTGCACCGGCGGCTGCTGGG

59134 59169 58444 60738 CDS product DNA packaging terminase subunit 2 59134-59169 8:05:02
GGCAGGCGGCGGTGCGGGTGGCCGACGCGGACATGG
59228 59247 58444 60738 CDS product DNA packaging terminase subunit 2 59228-59247 4:01:01
GGCGCTCCGGGCGCTGGAGG
59252 59279 58444 60738 CDS product DNA packaging terminase subunit 2 59252-59279 5:02:01
CCGTGCACCCGGGCGCCCCCCGGCGCCC
59389 59421 58444 60738 CDS product DNA packaging terminase subunit 2 59389-59421 6:03:01
GGCTGGCCTCGGCCGAGCGGGGCGAGGGCGGGG
59449 59472 58444 60738 CDS product DNA packaging terminase subunit 2 59449-59472 4:01:01
CCAACCTGGAGACCCTGGGGCGCC
59486 59501 58444 60738 CDS product DNA packaging terminase subunit 2 59486-59501 4:01:01 CCACCTCCGAGCGGCC
59548 59577 58444 60738 CDS product DNA packaging terminase subunit 2 59548-59577 5:02:01
GGGTCTTCGCGGACGGGCTGCTGGACCTGG
59660 59677 58444 60738 CDS product DNA packaging terminase subunit 2 59660-59677 4:01:01
CCACCACATGTCCGCACC
59738 59757 58444 60738 CDS product DNA packaging terminase subunit 2 59738-59757 4:01:01
GGGGCGCGTAGGCATGGGGG
60077 60105 58444 60738 CDS product DNA packaging terminase subunit 2 60077-60105 6:03:01
GGCGGTGGGGCGCGCCGGAACGGTTGTGG
60124 60141 58444 60738 CDS product DNA packaging terminase subunit 2 60124-60141 4:01:01
CCGCCTTCGACTCCCACC
60347 60371 58444 60738 CDS product DNA packaging terminase subunit 2 60347-60371 4:01:01
GGTGACAGCGGCCCGCGGCGATTGG

60424 60443 58444 60738 CDS product DNA packaging terminase subunit 2 60424-60443 5:02:01
CCGCCACCCAGCGCCTGGCC
60626 60653 58444 60738 CDS product DNA packaging terminase subunit 2 60626-60653 6:03:01
GGGCCCGGAGGGTACGGTCCGGGAGGGG
60713 60747 60600 63581 CDS product envelope glycoprotein B 60713-60747 5:02:01
GGCTCCGGGGTCTGGGGCGAGCTAGGCGCCATCGG
60751 60792 60600 63581 CDS product envelope glycoprotein B 60751-60792 8:05:02
CCGCTGCTCCCCGGGCGCCGTTGCCGGCGCCCGGCCACCGCC
60835 60861 60600 63581 CDS product envelope glycoprotein B 60835-60861 5:02:01 GGGGCTGCTGGCCGCGGGGCGGTGCGG
60864 60918 60600 63581 CDS product envelope glycoprotein B 60864-60918 11:08:02
CCACTCCGACGTCGCCCCCGGCCACGCCAGCGCCCCCCACGACCCCTAGCCCGCC
60930 60961 60600 63581 CDS product envelope glycoprotein B 60930-60961 7:04:01
CCTCCGAGCCGGCCGAACCGTCGCCGCTGCCC
60965 60987 60600 63581 CDS product envelope glycoprotein B 60965-60987 5:02:01 GGCGGCGAGGAAGACGGCGACGG
61006 61031 60600 63581 CDS product envelope glycoprotein B 61006-61031 5:02:01 CCTCCGGGGCGCCGTCCGCGACGCCC
61062 61084 60600 63581 CDS product envelope glycoprotein B 61062-61084 6:03:01 CCTGCCCGCCGCCCACCGGATCC
61096 61119 60600 63581 CDS product envelope glycoprotein B 61096-61119 5:02:01 CCTCGAGCCCCCCCGCGCCTGCCC
61260 61297 60600 63581 CDS product envelope glycoprotein B 61260-61297 7:04:01
CCATTACCAACCGCTACACCGACCGCGTGCCCGTCTCC
61341 61361 60600 63581 CDS product envelope glycoprotein B 61341-61361 5:02:01 CCTCCAAGGCCGAGTACCTCC
61393 61409 60600 63581 CDS product envelope glycoprotein B 61393-61409 4:01:01 GGACGCGGACGAGGAGG
61417 61444 60600 63581 CDS product envelope glycoprotein B 61417-61444 6:03:01 CCTGAGGGCCTCCAAGTTCACCACCCCC
61543 61562 60600 63581 CDS product envelope glycoprotein B 61543-61562 5:02:01 GGAGGTGGACGCCCGGTCGG

61774 61789 60600 63581 CDS product envelope glycoprotein B 61774-61789 4:01:01 GGTCGGCTGGGACTGG
61885 61903 60600 63581 CDS product envelope glycoprotein B 61885-61903 4:01:01 CCGGGCCCTTTCCACGACC
62033 62054 60600 63581 CDS product envelope glycoprotein B 62033-62054 4:01:01 CCCATGGAAACCTACCTTGCCC
62073 62092 60600 63581 CDS product envelope glycoprotein B 62073-62092 4:01:01 CCTTCCGGCCCATGCTCTCC
62133 62152 60600 63581 CDS product envelope glycoprotein B 62133-62152 4:01:01 CCAACCGCACCTACGACCCC
62232 62257 60600 63581 CDS product envelope glycoprotein B 62232-62257 5:02:01 CCGAGGCCGTGCCCGCCGCGCAGCCC
62281 62317 60600 63581 CDS product envelope glycoprotein B 62281-62317 8:05:02 CCTGCGCCGCCGGGCCGCGGATGCCGCCACCCAGGCC
62314 62333 60600 63581 CDS product envelope glycoprotein B 62314-62333 4:01:01 GGCCGGCAAAGACGGCAAGG
62506 62531 60600 63581 CDS product envelope glycoprotein B 62506-62531 4:01:01 CCCCAGCGCCATCGCCTCGTCTTCCC
62659 62677 60600 63581 CDS product envelope glycoprotein B 62659-62677 4:01:01 CCCCCCCGTGACCTTCACC
63088 63141 60600 63581 CDS product envelope glycoprotein B 63088-63141 8:05:02 GGGCCTGGGCAAAGCGGGCCAGGCCATCGGCAAGCTGGTGATCGGGGCCGCGGG
63135 63163 60600 63581 CDS product envelope glycoprotein B 63135-63163 5:02:01 CCGCGGGCGCCGTCGTCTCCACCGTGTCC
63225 63241 60600 63581 CDS product envelope glycoprotein B 63225-63241 4:01:01 CCGGCCTCGTGGCCGCC
63276 63301 60600 63581 CDS product envelope glycoprotein B 63276-63301 4:01:01 CCAACCCCATGAAGGCCCTGTACCCC
63346 63360 60600 63581 CDS product envelope glycoprotein B 63346-63360 4:01:01 GGGCGGAGGCGACGG
63376 63398 60600 63581 CDS product envelope glycoprotein B 63376-63398 4:01:01 GGACAAGCTGGAGCAGGCCCGGG
63472 63509 60600 63581 CDS product envelope glycoprotein B 63472-63509 7:04:01 CCCCGCCCTAATCGCCAACCACGTCTCCAACCTCGCCC
63832 63857 63814 64314 CDS product protein V32 63832-63857 6:03:01 CCGCTCCCGCCGAGTCCCCCCTGGCC
63854 63870 63814 64314 CDS product protein V32 63854-63870 4:01:01 GGCCGAGGAGGTTTCGG

63892 63911 63814 64314 CDS product protein V32 63892-63911 4:01:01 CCTCCGAAGCGCCCGCGGCC
63946 63974 63814 64314 CDS product protein V32 63946-63974 5:02:01 CCGGCGCTGCCAGCACCCCCGCGAGCGCC
63995 64020 63814 64314 CDS product protein V32 63995-64020 4:01:01 GGACACCGAGGAGCTGCTGGAGATGG
64054 64083 63814 64314 CDS product protein V32 64054-64083 5:02:01 CCCCCCAGCCGGCACAGCCCGCATTCACCC
64246 64268 63814 64314 CDS product protein V32 64246-64268 4:01:01 CCCCCGTGTGCCCCTGGATGGCC
64467 64503 64409 65437 CDS product capsid scaffold protein 64467-64503 8:05:02 GGGTGGTGGCGGCGCTGGCATCGACGGTCTGGGTGGG
64555 64586 64409 65437 CDS product capsid scaffold protein 64555-64586 5:02:01 GGGCCTCGCGGCTTCCGCGGACGGCTGCTGGG
64606 64635 64409 65437 CDS product capsid scaffold protein 64606-64635 7:04:01 GGCTGGCGGCGGGCAGGGCTGGGAGTGGGG
64669 64684 64409 65437 CDS product capsid scaffold protein 64669-64684 4:01:01 GGGCGGGTGAGGATGG
64716 64734 64409 65437 CDS product capsid scaffold protein 64716-64734 4:01:01 CCGACACGACCGCCTGCCC
64739 64763 64409 65437 CDS product capsid scaffold protein 64739-64763 5:02:01 GGATACTGGGGGTAGACTGGCGGGG
64773 64821 64409 65437 CDS product capsid scaffold protein 64773-64821 9:06:02 GGTGGCCCGCAAGGGCGGTCCCCGGGTAAACGGCCGGCGCGCCGGCGGG
64845 64862 64409 65437 CDS product capsid scaffold protein 64845-64862 4:01:01 GGACGGCCCGGAGCTGGG
64903 64929 64409 65437 CDS product capsid scaffold protein 64903-64929 5:02:01 GGCTATGGTCGTCGAGGGGCGGTGGGG
65069 65094 64409 65437 CDS product capsid scaffold protein 65069-65094 6:03:01 GGGGACGGGGGGGTGGGCTGGCATGG
65120 65138 64409 65437 CDS product capsid scaffold protein 65120-65138 4:01:01 CCGCCGCCCCTTGCGGCCC
65134 65150 64409 65437 CDS product capsid scaffold protein 65134-65150 4:01:01 GGCCCTGCGGTCGGCGG
65239 65281 64409 65437 CDS product capsid scaffold protein 65239-65281 7:04:01 GGCCACTGCGGGCGCGTGGGGCGGGAGCGGGGCGTACGGGTGG

65346 65371 64409 65437 CDS product capsid scaffold protein 65346-65371 4:01:01 GGTTGTACTGGGCCGTAGGAACGAGG
65383 65409 64409 65437 CDS product capsid scaffold protein 65383-65409 5:02:01 GGAAACAAGGGGGGTTCCCGGAGCCGG
65418 65432 64409 65437 CDS product capsid scaffold protein 65418-65432 4:01:01 GGGAGCCGGAGGAGG
65445 65470 64409 66394 CDS product capsid maturation protease 65445-65470 5:02:01
GGTGCTCGGGGCGAGGGAGGTTGCGG
65494 65522 64409 66394 CDS product capsid maturation protease 65494-65522 6:03:01
GGGGGCGGAAGGCGTAGGCGCTAGAGGGG
65600 65619 64409 66394 CDS product capsid maturation protease 65600-65619 4:01:01 CCTTCCCGCGACTCCGCCCC
65687 65714 64409 66394 CDS product capsid maturation protease 65687-65714 6:03:01
CCCGCGATGCCGGCCTCGCGCCGCCGCC
65802 65820 64409 66394 CDS product capsid maturation protease 65802-65820 4:01:01 CCCACCGCGCGGCCTCCCC
65862 65885 64409 66394 CDS product capsid maturation protease 65862-65885 5:02:01 CCCGCACCCCCGGCGCCAAGCGCC
65895 65905 64409 66394 CDS product capsid maturation protease 65895-65905 4:01:01 CCACCGCCGCC
66075 66108 64409 66394 CDS product capsid maturation protease 66075-66108 5:02:01
CCGTCAGCCCCTCCCCGAGGTCCCCGAAAAATCC
66244 66281 64409 66394 CDS product capsid maturation protease 66244-66281 8:05:02
GGGGAGCTGGGAGGCCGGCGGAAGGGCGGCCTCGACGG
66760 66783 66507 68294 CDS product DNA packaging tegument protein UL25 66760-66783 5:02:01
GGCTGGCTTGGTGCCGGCCCGAGG
66948 66971 66507 68294 CDS product DNA packaging tegument protein UL25 66948-66971 5:02:01
CCACTCCGGCTGGCCATGGCCGCC
66988 67015 66507 68294 CDS product DNA packaging tegument protein UL25 66988-67015 4:01:01
CCCCGCGGGCTCCCGGCGCCAGCGCGCC

67189 67223 66507 68294 CDS product DNA packaging tegument protein UL25 67189-67223 6:03:01
CCAAAAACGCCGCCGCGGCCCTGTTAACCTCGTCC
67246 67288 66507 68294 CDS product DNA packaging tegument protein UL25 67246-67288 8:05:02
CCAGCGCCGCACCCGCCGGCGCCGCACCGGCGCCGAGGCTTCC
67274 67300 66507 68294 CDS product DNA packaging tegument protein UL25 67274-67300 5:02:01
GGCGCCGAGGCTTCCAGGAACGGGGGG
67364 67378 66507 68294 CDS product DNA packaging tegument protein UL25 67364-67378 4:01:01 CCGCCCCCCGTTTCC
67393 67422 66507 68294 CDS product DNA packaging tegument protein UL25 67393-67422 6:03:01
GGTGCTTGGGGAGGCGGGCGTGGTCGAAGG
67436 67454 66507 68294 CDS product DNA packaging tegument protein UL25 67436-67454 4:01:01
CCCGGTGCCGCCCTCGCCC
67460 67487 66507 68294 CDS product DNA packaging tegument protein UL25 67460-67487 6:03:01
GGCGGCGAGGGCGTCCAGGCAGGCCGGG
67518 67562 66507 68294 CDS product DNA packaging tegument protein UL25 67518-67562 7:04:01
GGGTACGACTGGCTAGGGGCCAGGGAGCGGTGCGCAAGGTGCAGG
67658 67677 66507 68294 CDS product DNA packaging tegument protein UL25 67658-67677 4:01:01
CCGAGACATCCGCCCGTCCC
67714 67796 66507 68294 CDS product DNA packaging tegument protein UL25 67714-67796 14:11:03
CCACCAAACGGTCCTGCAGGGCCCGGTACCACGTTCCGAACACCACCCCGTTGGATCCCCCCGCCGCCCGCCCCACGAACACC
67907 67930 66507 68294 CDS product DNA packaging tegument protein UL25 67907-67930 6:03:01
CCCGCCGCCCCCGCAATTACCGCC
67934 67956 66507 68294 CDS product DNA packaging tegument protein UL25 67934-67956 6:03:01
GGGGGGGGAGGACGCGGCGTCGG

67957 67994 66507 68294 CDS product DNA packaging tegument protein UL25 67957-67994 9:06:02 CCTCCTCCGCGGCCCGCGCCGCGTCCTCCAGCGCCGCC
68008 68027 66507 68294 CDS product DNA packaging tegument protein UL25 68008-68027 5:02:01 CCTTCGCCACCTGCCGCTCC
68141 68160 66507 68294 CDS product DNA packaging tegument protein UL25 68141-68160 4:01:01 GGCGCGGTGGGCATTGTCGG
68161 68187 66507 68294 CDS product DNA packaging tegument protein UL25 68161-68187 5:02:01 CCGAAAACGCCGGGCCGCTCCAGTACC
68390 68415 68361 69104 CDS product nuclear protein UL24 68390-68415 6:03:01 GGCCAGCGGGTCGGGCGGGTGGGCGG
68416 68452 68361 69104 CDS product nuclear protein UL24 68416-68452 7:04:01 CCGCGCCGCTCGGGCCAGCCGCCGGCGCGCCGGAACC
68439 68504 68361 69104 CDS product nuclear protein UL24 68439-68504 14:11:03 GGCGCGCCGGAACCGGGGGCGGGGCTGGGGGCTGGGGGCCGCCGGGCGAGAGGCGGAGCCCGGCGG
68497 68534 68361 69104 CDS product nuclear protein UL24 68497-68534 6:03:01 CCCGGCGGCCCCTTTTCGGCCGCGCCCGCCGGGCGGCC
68527 68542 68361 69104 CDS product nuclear protein UL24 68527-68542 4:01:01 GGGCGGCCACGGGCGG
68571 68585 68361 69104 CDS product nuclear protein UL24 68571-68585 4:01:01 CCGGCCAGGACCGCC
68639 68660 68361 69104 CDS product nuclear protein UL24 68639-68660 4:01:01 CCCCCGCTGCGCCACAAACACC
68671 68693 68361 69104 CDS product nuclear protein UL24 68671-68693 5:02:01 CCAGGACGACCTCTCCGCCGCCC
68694 68709 68361 69104 CDS product nuclear protein UL24 68694-68709 4:01:01 GGCGGGAGGGTTCTGG
68877 68894 68361 69104 CDS product nuclear protein UL24 68877-68894 4:01:01 GGGCGGCGCTGGCCCAGG
68991 69011 68361 69104 CDS product nuclear protein UL24 68991-69011 5:02:01 CCGCTGCGCCTGGCCGCCGCC
69280 69298 69148 70200 CDS product thymidine kinase 69280-69298 4:01:01 CCGCCGGCCGGGGGAGCCC

69385 69411 69148 70200 CDS product thymidine kinase 69385-69411 6:03:01 GGAAACGGGCCGGGGGGCTGACGGGGG
69454 69474 69148 70200 CDS product thymidine kinase 69454-69474 4:01:01 CCACGCCCTACCTGCTGCTCC
69523 69543 69148 70200 CDS product thymidine kinase 69523-69543 5:02:01 CCGCCGCGCCCGACCTCGTCC
69625 69675 69148 70200 CDS product thymidine kinase 69625-69675 11:08:02
CCCTGCTCGCCATGGCCGCCACCCTCCCCCCTGAAGCCCCCGGGGCCAACC
69713 69732 69148 70200 CDS product thymidine kinase 69713-69732 4:01:01 CCGCCTGCGCGCCCGCGCCC
69822 69848 69148 70200 CDS product thymidine kinase 69822-69848 5:02:01 GGGCGCGTCTGGCGCGACGGCTGGGGG
69855 69876 69148 70200 CDS product thymidine kinase 69855-69876 5:02:01 CCCCCCTTCACTGCCGCCCGCC
69971 69996 69148 70200 CDS product thymidine kinase 69971-69996 5:02:01 GGATGAGCGGGGGGTGCTCCTGGAGG
70072 70094 69148 70200 CDS product thymidine kinase 70072-70094 5:02:01 CCCCGCGCCAGTGCGCCGCCGCC
70132 70155 69148 70200 CDS product thymidine kinase 70132-70155 6:03:01 CCGGGGCCGCCGCAGCCGCCGCCC
70515 70604 70439 73075 CDS product envelope glycoprotein H 70515-70604 16:13:04
CCCGGTTCCCACCCCGGCGCCGCCGTCTCCGTCGCCGGCGACCCCGTCCCCCACAACGCCCCCAACGACTCCGTCTCCCCCGCTGTTTCC
70644 70663 70439 73075 CDS product envelope glycoprotein H 70644-70663 4:01:01 GGAAGCCGGCTTCGGCTTGG
70842 70860 70439 73075 CDS product envelope glycoprotein H 70842-70860 4:01:01 CCCCCGCCTAGGCGACCCC
71056 71079 70439 73075 CDS product envelope glycoprotein H 71056-71079 4:01:01 CCAAAACCCTCGGGCCGCAAGACC
71133 71153 70439 73075 CDS product envelope glycoprotein H 71133-71153 4:01:01 CCCTTTCGCCTTCCGGGCCCC
71243 71260 70439 73075 CDS product envelope glycoprotein H 71243-71260 4:01:01 CCGTCGCCACCATCGGCC
71288 71303 70439 73075 CDS product envelope glycoprotein H 71288-71303 4:01:01 CCGTGAACCACCCCCC
71352 71368 70439 73075 CDS product envelope glycoprotein H 71352-71368 4:01:01 CCGTCTCGCCCCCAGCC
71380 71404 70439 73075 CDS product envelope glycoprotein H 71380-71404 6:03:01 CCCCCAGGGCCTCCGGCCGGGCCCC
71466 71479 70439 73075 CDS product envelope glycoprotein H 71466-71479 4:01:01 GGCCCGGGCGGTGG
71499 71518 70439 73075 CDS product envelope glycoprotein H 71499-71518 4:01:01 CCTGGACTACCGCTACCACC

71556 71575 70439 73075 CDS product envelope glycoprotein H 71556-71575 5:02:01 GGAGGCCGGCAGAAAGGGGG
71781 71815 70439 73075 CDS product envelope glycoprotein H 71781-71815 6:03:01 CCTGGCCTTCGACCGCTCCGACGTCGCCGGTCGCC
71822 71836 70439 73075 CDS product envelope glycoprotein H 71822-71836 4:01:01 GGCGCAGGGTGGCGG
71847 71872 70439 73075 CDS product envelope glycoprotein H 71847-71872 6:03:01 GGAGGCGGCCTCGCGGCGGCCCCTGG
71912 71928 70439 73075 CDS product envelope glycoprotein H 71912-71928 4:01:01 CCGACCGGCGCCTGCCC
71934 71961 70439 73075 CDS product envelope glycoprotein H 71934-71961 6:03:01 GGTGCGGCTGGCCGGCGACCTGGCAAGG
72044 72063 70439 73075 CDS product envelope glycoprotein H 72044-72063 4:01:01 CCGCCGGGCGCCAGTCGCCC
72083 72101 70439 73075 CDS product envelope glycoprotein H 72083-72101 5:02:01 CCGAGCCCACCGGCCCCCC
72210 72227 70439 73075 CDS product envelope glycoprotein H 72210-72227 4:01:01 GGAAACGGCCCTGGCCGG
72218 72256 70439 73075 CDS product envelope glycoprotein H 72218-72256 7:04:01 CCCTGGCCGGCCTGTCTGCCGGCGGCCCCCAGTTTAGCC
72378 72400 70439 73075 CDS product envelope glycoprotein H 72378-72400 5:02:01 GGAGGCCAGCGGCGAGGCTTTGG
72491 72517 70439 73075 CDS product envelope glycoprotein H 72491-72517 5:02:01 CCTGCACCAAGCCGCTGCCCAAGCTCC
72675 72699 70439 73075 CDS product envelope glycoprotein H 72675-72699 5:02:01 CCGGGCGGCCACCCTGTTCCACCCC
72827 72849 70439 73075 CDS product envelope glycoprotein H 72827-72849 5:02:01 CCACCATCCCAGCCTTTAACCCC
74561 74583 74386 75978 CDS product tegument protein UL21 74561-74583 5:02:01 CCGCTGCCGCCCAAATGCCCTCC
74595 74613 74386 75978 CDS product tegument protein UL21 74595-74613 4:01:01 CCGGCCGAGAACCTTGCCC
74656 74686 74386 75978 CDS product tegument protein UL21 74656-74686 5:02:01 GGAACTCTCGGCGCGCCGGCGAGGCTCACGG
74693 74709 74386 75978 CDS product tegument protein UL21 74693-74709 4:01:01 CCGCCAGCCACACTGCC
74752 74769 74386 75978 CDS product tegument protein UL21 74752-74769 5:02:01 GGCGGGGGGTCGAGGCGG
74844 74859 74386 75978 CDS product tegument protein UL21 74844-74859 4:01:01 GGCCTCGGGAAGGCGG

75092 75111 74386 75978 CDS product tegument protein UL21 75092-75111 4:01:01 CCAGGCCGTCCCACGTTTCC
75402 75426 74386 75978 CDS product tegument protein UL21 75402-75426 5:02:01 CCCGTCCACCAGCGCCTCGAGCTCC
75678 75696 74386 75978 CDS product tegument protein UL21 75678-75696 4:01:01 CCCCTGCGCCAAGACCTCC
75791 75822 74386 75978 CDS product tegument protein UL21 75791-75822 5:02:01
GGTCTCCAGGCGTTAGGCCGCGGACCGTCAGG
76562 76587 76498 77223 CDS product envelope protein UL20 76562-76587 6:03:01 GGAGGCGCAGGCGGCGGACGACGGGG
76661 76683 76498 77223 CDS product envelope protein UL20 76661-76683 4:01:01 GGTGAGCTCGGCCGGCGCGCGGG
76697 76712 76498 77223 CDS product envelope protein UL20 76697-76712 4:01:01 CCAGCCCGCCTTTTCC
76817 76839 76498 77223 CDS product envelope protein UL20 76817-76839 5:02:01 GGCAGGGGGTGCGGCAACGCTGG
76938 76952 76498 77223 CDS product envelope protein UL20 76938-76952 4:01:01 GGGCTGGCGGTGGGG
77066 77085 76498 77223 CDS product envelope protein UL20 77066-77085 5:02:01 CCACCGGCTCCTGCCGCTCC
77128 77147 76498 77223 CDS product envelope protein UL20 77128-77147 4:01:01 CCGTTTACGCCGCCGACGCC
77477 77495 77440 81570 CDS product major capsid protein 77477-77495 5:02:01 GGAGGTGGTTCCGGCCGGG
77757 77781 77440 81570 CDS product major capsid protein 77757-77781 5:02:01 CCCCACCCGGTCGACCAGCCCATCC
78236 78262 77440 81570 CDS product major capsid protein 78236-78262 5:02:01 GGACACCAAGGGCCGCGCGGTGGACGG
78395 78416 77440 81570 CDS product major capsid protein 78395-78416 4:01:01 GGTTATGGGCAAGGCCGTCCGG
78590 78615 77440 81570 CDS product major capsid protein 78590-78615 6:03:01 CCAGGCCACCAACGTTCCCTACCCCC
78706 78752 77440 81570 CDS product major capsid protein 78706-78752 9:06:02
CCCCGAGTCCCGGCCAGCCGGACCCGCGCACCTACCCCCCCAAGACC
78805 78830 77440 81570 CDS product major capsid protein 78805-78830 4:01:01 CCGCGGGCACCCTCTGCCACAGCTCC
78918 78936 77440 81570 CDS product major capsid protein 78918-78936 4:01:01 GGCGCGGGCGGGCTGCTGG
79241 79286 77440 81570 CDS product major capsid protein 79241-79286 10:07:02
CCGGCACCGCCTGAGCCCCGCCACGGTGGCCGCCGTCCGCGCCACC

79553 79591 77440 81570 CDS product major capsid protein 79553-79591 8:05:02 GGACTACACGGTCCCGGGGGAGGCTCTCGGCGGCCAGGG

79613 79641 77440 81570 CDS product major capsid protein 79613-79641 6:03:01 CCTGGTAGACCCCGCCTTCCTCCCGCCCC

79658 79680 77440 81570 CDS product major capsid protein 79658-79680 5:02:01 CCCCCTCATCTACCGGGCCGACC

79741 79758 77440 81570 CDS product major capsid protein 79741-79758 4:01:01 GGGTGGAGATGGCCGAGG

79826 79849 77440 81570 CDS product major capsid protein 79826-79849 4:01:01 CCCCGTCGTTCCCCACCACGACCC

79885 79904 77440 81570 CDS product major capsid protein 79885-79904 4:01:01 CCGTGGTTCCCGCCTTCTCC

80001 80053 77440 81570 CDS product major capsid protein 80001-80053 8:05:02 CCCACGAGCCCGAGCGACCCGCGCCACCCGCTCAACCCGCGGCACCTCGTCCC

80157 80208 77440 81570 CDS product major capsid protein 80157-80208 9:06:02 CCCATCCTGGCGGCCGTGGCCCCCGACGCCGGAACAGCCACGGCCGTGACCC

80237 80250 77440 81570 CDS product major capsid protein 80237-80250 4:01:01 CCACCACGGCCTCC

80437 80460 77440 81570 CDS product major capsid protein 80437-80460 5:02:01 CCACCGTCCGACAGCCCGTGGTCC

80714 80732 77440 81570 CDS product major capsid protein 80714-80732 4:01:01 CCTTACCCAGCCCCGCGCC

80810 80829 77440 81570 CDS product major capsid protein 80810-80829 5:02:01 CCTCCCCCAGAACCTGTACC

80898 80925 77440 81570 CDS product major capsid protein 80898-80925 5:02:01 GGCAACCGGCTCGGGCCCCAGGGCCCGG

80949 80963 77440 81570 CDS product major capsid protein 80949-80963 4:01:01 CCAGCCGCCCCGGCC

81137 81153 77440 81570 CDS product major capsid protein 81137-81153 4:01:01 CCCAGCCTACCCAAGCC

81278 81296 77440 81570 CDS product major capsid protein 81278-81296 4:01:01 GGAGGTGGAGACGAAGGGG

81319 81359 77440 81570 CDS product major capsid protein 81319-81359 7:04:01 CCGAGGCCGGCGCCTCCGTGGCGCCCAGCACGGCCAACACC

81384 81405 77440 81570 CDS product major capsid protein 81384-81405 4:01:01 GGGTCCGCGGACCTTGTGGAGG

81925 81943 81813 82763 CDS product capsid triplex subunit 2 81925-81943 4:01:01 CCGCCGGGCCATGCTAGCC
81980 82027 81813 82763 CDS product capsid triplex subunit 2 81980-82027 10:07:02 CCTCCGGACACCCTCTCCCTCATGGCCGCCTACCGCCGCCGGTTTCCC
82108 82130 81813 82763 CDS product capsid triplex subunit 2 82108-82130 4:01:01 CCAAAACACCGGCCCCTTTGACC
82160 82193 81813 82763 CDS product capsid triplex subunit 2 82160-82193 6:03:01 CCGCCGCTCCTGGACCTGGAAGACCGGCTGCGCC
82370 82385 81813 82763 CDS product capsid triplex subunit 2 82370-82385 4:01:01 CCGGACCTCCAGCACC
82567 82589 81813 82763 CDS product capsid triplex subunit 2 82567-82589 5:02:01 CCAACAGCCGCCGCCGCGGCCCC
82621 82646 81813 82763 CDS product capsid triplex subunit 2 82621-82646 5:02:01 GGCGCTGATGGCGTGGATTGGAACGG
82706 82736 81813 82763 CDS product capsid triplex subunit 2 82706-82736 6:03:01 GGCCCATCGGTGGTGGCCCGAGGTGAGAAGG
82909 82930 82877 88735 CDS product DNA packaging terminase subunit 1 82909-82930 5:02:01 GGACGGGGGGAGGCTGCTCAGG
82938 82954 82877 88735 CDS product DNA packaging terminase subunit 1 82938-82954 4:01:01 CCATCACCAGGGCCACC
82965 82986 82877 88735 CDS product DNA packaging terminase subunit 1 82965-82986 4:01:01 CCGACGCGCCGCCTCGCTTCCC
83330 83350 82877 88735 CDS product DNA packaging terminase subunit 1 83330-83350 4:01:01 CCCTCGACGGCCACCCGCACC
83359 83375 82877 88735 CDS product DNA packaging terminase subunit 1 83359-83375 4:01:01 GGGTCGCGGGTGGATGG
83495 83524 82877 88735 CDS product DNA packaging terminase subunit 1 83495-83524 6:03:01

CCCACGACCGCCACGCCCGTCCCGGACGCC
83518 83548 82877 88735 CDS product DNA packaging terminase subunit 1 83518-83548 6:03:01
GGACGCCAGGGAGTTGGTGGTAAAGGCCGGG
83580 83596 82877 88735 CDS product DNA packaging terminase subunit 1 83580-83596 4:01:01
CCGCCTGGTTCGCCACC
83664 83685 82877 88735 CDS product DNA packaging terminase subunit 1 83664-83685 6:03:01
CCCCCACTACGCCCCCACCGCC
83868 83878 82877 88735 CDS product DNA packaging terminase subunit 1 83868-83878 4:01:01 CCGCCGCCCCC
84125 84144 82877 88735 CDS product DNA packaging terminase subunit 1 84125-84144 5:02:01
CCCCCGCGCGCCCCACCACC
84158 84215 82877 88735 CDS product DNA packaging terminase subunit 1 84158-84215 11:08:02
CCGCCTTTTCCGCTCTTGCCGCCCGTCGGCCGCACGCCCGCCCCCGCCATGAACGCCC
84252 84278 84207 86327 CDS product DNA packaging tegument protein UL17 84252-84278 5:02:01
CCCGTCGCCGAGACCGGGGCCTGGTCC
84290 84313 84207 86327 CDS product DNA packaging tegument protein UL17 84290-84313 5:02:01
CCCGACGCCTGCCTCGCCAAGGCC
84745 84759 84207 86327 CDS product DNA packaging tegument protein UL17 84745-84759 4:01:01 CCGCCCTGACCTGCC
84793 84821 84207 86327 CDS product DNA packaging tegument protein UL17 84793-84821 5:02:01
GGCCGGCCTCCGGGACGCGGCCGCTTCGG
84835 84851 84207 86327 CDS product DNA packaging tegument protein UL17 84835-84851 4:01:01
GGAGGTGGTCGACGGGG
84948 84964 84207 86327 CDS product DNA packaging tegument protein UL17 84948-84964 4:01:01
CCCTTATCCGCCGCGCC

85018 85039 84207 86327 CDS product DNA packaging tegument protein UL17 85018-85039 5:02:01 GGGCGGCGGGCCGGTTCGAAGG
85048 85077 84207 86327 CDS product DNA packaging tegument protein UL17 85048-85077 6:03:01 GGCCTCGGGGCTGCGGCAGGGGGCGCGTGG
85080 85107 84207 86327 CDS product DNA packaging tegument protein UL17 85080-85107 5:02:01 CCCGCCCGCCGGACGCCCCGGCTGCCCC
85090 85123 84207 86327 CDS product DNA packaging tegument protein UL17 85090-85123 6:03:01 GGACGCCCCGGCTGCCCCGGGCGCGGGGGGCGGG
85186 85198 84207 86327 CDS product DNA packaging tegument protein UL17 85186-85198 4:01:01 GGGGGGCGGCCGG
85195 85214 84207 86327 CDS product DNA packaging tegument protein UL17 85195-85214 5:02:01 CCGGCGCCACCTGCCCCGCC
85229 85259 84207 86327 CDS product DNA packaging tegument protein UL17 85229-85259 5:02:01 CCCGCGGACCGCGAAAGCCCCGCCGGCGCCC
85283 85306 84207 86327 CDS product DNA packaging tegument protein UL17 85283-85306 5:02:01 CCCGAGAACGCCAACCCCCTCACC
85471 85513 84207 86327 CDS product DNA packaging tegument protein UL17 85471-85513 8:05:02 CCGCGCGCCCACGGGCCACCTCGCCGGCTCGGCCCACCCGGCC
85521 85544 84207 86327 CDS product DNA packaging tegument protein UL17 85521-85544 4:01:01 GGGCGTGCCTGGTCGCCGGCGTGG
85546 85574 84207 86327 CDS product DNA packaging tegument protein UL17 85546-85574 6:03:01 CCACCCGCGCAACCTGCCTCCCGAGGCCC
85613 85635 84207 86327 CDS product DNA packaging tegument protein UL17 85613-85635 5:02:01 CCGCTCGCCAACACGCCGCCGCC

85722 85743 84207 86327 CDS product DNA packaging tegument protein UL17 85722-85743 4:01:01 CCGAAAGCCTGCGCGCCAGCCC
85774 85787 84207 86327 CDS product DNA packaging tegument protein UL17 85774-85787 4:01:01 CCACGACCGCCACC
85831 85847 84207 86327 CDS product DNA packaging tegument protein UL17 85831-85847 4:01:01 GGAGGCCGTGGCCTTGG
85836 85870 84207 86327 CDS product DNA packaging tegument protein UL17 85836-85870 8:05:02 CCGTGGCCTTGGCCGTCCGCCACGCCTCCGTCGCC
85912 85936 84207 86327 CDS product DNA packaging tegument protein UL17 85912-85936 4:01:01 CCTCAGCTCCCGGGCCGGGGCTCCC
86020 86033 84207 86327 CDS product DNA packaging tegument protein UL17 86020-86033 4:01:01 GGGCGGCGAGGTGG
86417 86439 86344 87510 CDS product tegument protein UL16 86417-86439 5:02:01 GGCGGCGCGGCGCCTGGCCGAGG
86429 86447 86344 87510 CDS product tegument protein UL16 86429-86447 4:01:01 CCTGGCCGAGGCCCTCACC
86458 86485 86344 87510 CDS product tegument protein UL16 86458-86485 5:02:01 CCGCCCTGCGCCTCGTCCGCGGGGACCC
86516 86557 86344 87510 CDS product tegument protein UL16 86516-86557 10:07:02 CCTCAGCCCCCGCCTGGCGCCGTTCGTACCGCCCCCGCCCCC
86567 86585 86344 87510 CDS product tegument protein UL16 86567-86585 4:01:01 CCACGCCTCCAAGTGTGCC
86781 86810 86344 87510 CDS product tegument protein UL16 86781-86810 6:03:01 CCCGACCCGGCAGCCGAGACCGTCCCCGCC
86891 86909 86344 87510 CDS product tegument protein UL16 86891-86909 4:01:01 GGCGCCGGGCGTTTGGTGG
86976 86998 86344 87510 CDS product tegument protein UL16 86976-86998 5:02:01 GGGTGGCGGGCGCGCGGCCTTGG
87239 87264 86344 87510 CDS product tegument protein UL16 87239-87264 5:02:01 CCGCAGCCCCAAGATCACCCCCGACC
87266 87298 86344 87510 CDS product tegument protein UL16 87266-87298 6:03:01 GGCGGACGTGCTGGTGGGCCTGGGCCCCCACGG

87284 87300 86344 87510 CDS product tegument protein UL16 87284-87300 4:01:01 CCTGGGCCCCCACGGCC
87324 87351 86344 87510 CDS product tegument protein UL16 87324-87351 6:03:01 GGCTGGAAGCTGGCGGTGCTGGACCCGG
87408 87431 86344 87510 CDS product tegument protein UL16 87408-87431 4:01:01 GGCCCTCGCGGCGAGGAACACCGG
87442 87458 86344 87510 CDS product tegument protein UL16 87442-87458 4:01:01 GGCGGGCCTCGGGCAGG
87461 87483 86344 87510 CDS product tegument protein UL16 87461-87483 4:01:01 CCCGTCCGGGTCCGGACGGTCCC
87468 87498 86344 87510 CDS product tegument protein UL16 87468-87498 7:04:01 GGGTCCGGACGGTCCCGGTGCGGCGAGGCGG
87593 87638 82877 88735 CDS product DNA packaging terminase subunit 1 87593-87638 9:06:02 GGCGCGGTGTGGTCTTTGGGGACTGGGCAGGTTGGCGAGCGGCTGG
87647 87670 82877 88735 CDS product DNA packaging terminase subunit 1 87647-87670 6:03:01 GGTGAAGGGGGGCGGCGAGGGAGG
87942 87965 82877 88735 CDS product DNA packaging terminase subunit 1 87942-87965 4:01:01 GGCGACGCGGAACCAGGAAAACGG
88051 88073 82877 88735 CDS product DNA packaging terminase subunit 1 88051-88073 4:01:01 GGCGTGGTCGCCTAGGATGACGG
88175 88194 82877 88735 CDS product DNA packaging terminase subunit 1 88175-88194 4:01:01 CCCGCGCGCCGGCCGTCCCC
88370 88393 82877 88735 CDS product DNA packaging terminase subunit 1 88370-88393 4:01:01 CCCCTCAGAGCCTGGGTCACCTCC
88428 88468 82877 88735 CDS product DNA packaging terminase subunit 1 88428-88468 8:05:02 CCCGCCCTCGCGCCTCGCCGCAGCCGGCCTGCTTCCGGGCC
88486 88501 82877 88735 CDS product DNA packaging terminase subunit 1 88486-88501 5:02:01 CCGCCTCGCCACCGCC
88541 88558 82877 88735 CDS product DNA packaging terminase subunit 1 88541-88558 5:02:01

CCAACCCCCGGCACCACC
88674 88690 82877 88735 CDS product DNA packaging terminase subunit 1 88674-88690 4:01:01
CCTCCCGCCGGAGGTCC
88772 88800 88766 89689 CDS product tegument protein UL14 88772-88800 5:02:01
GGGGGTCCAGGTCAGCGCGGCGCCAGCGG
89049 89066 88766 89689 CDS product tegument protein UL14 89049-89066 4:01:01 CCGCCGCTTCCTGCATCC
89115 89131 88766 89689 CDS product tegument protein UL14 89115-89131 4:01:01 GGAGGACCGGCTAACGG
89228 89242 88766 89689 CDS product tegument protein UL14 89228-89242 4:01:01 GGATGTTGGGGGAGG
89301 89320 88766 89689 CDS product tegument protein UL14 89301-89320 4:01:01 GGGCGGGACCGGCGAGCTGG
89330 89354 88766 89689 CDS product tegument protein UL14 89330-89354 4:01:01 CCGCTGGGGCCAGGAGCCCGGACCC
89411 89452 88766 89689 CDS product tegument protein UL14 89411-89452 7:04:01
GGCCCGACGGCGGCTCGGGTCAGAGGCCCGCCCGGCCTCGGG
89458 89499 88766 89689 CDS product tegument protein UL14 89458-89499 7:04:01
CCTCCTCAGGGCCCATCGGATCCAGACAGTCCATGGCCGCCC
89492 89511 88766 89689 CDS product tegument protein UL14 89492-89511 4:01:01 GGCCGCCCGGTGTCGGGCGG
89521 89541 88766 89689 CDS product tegument protein UL14 89521-89541 4:01:01 GGCTCGTCGGACGGACCCGGG
89549 89579 88766 89689 CDS product tegument protein UL14 89549-89579 6:03:01
CCCCGAGTCCGCCCACCAGTCCGGGGAGCCC
89841 89865 89191 90939 CDS product tegument serine/threonine protein kinase 89841-89865 5:02:01
GGCGGCGCGGGGAGCTACGGCGAGG
90395 90410 89191 90939 CDS product tegument serine/threonine protein kinase 90395-90410 4:01:01
GGTGGTTGGCCACGGG
90476 90492 89191 90939 CDS product tegument serine/threonine protein kinase 90476-90492 4:01:01

CCCCCTCCGGCACGACC

90495 90507 89191 90939 CDS product tegument serine/threonine protein kinase 90495-90507 4:01:01 GGGCTGGCGGTGG

90557 90580 89191 90939 CDS product tegument serine/threonine protein kinase 90557-90580 4:01:01 CCTGTCGCCGTGCTTGCCGGTTCC

90683 90701 89191 90939 CDS product tegument serine/threonine protein kinase 90683-90701 4:01:01 CCCATCCACGCCGCTCACC

90800 90819 89191 90939 CDS product tegument serine/threonine protein kinase 90800-90819 4:01:01 CCGCCTGACGCACCGGCGCC

90840 90856 89191 90939 CDS product tegument serine/threonine protein kinase 90840-90856 4:01:01 CCCACCACCCTCTCTCC

90978 90994 90924 92609 CDS product deoxyribonuclease 90978-90994 4:01:01 CCTCCTACCCCTACGCC

91010 91043 90924 92609 CDS product deoxyribonuclease 91010-91043 7:04:01 CCCGCCTCCCTCGAGGACCCCCCCGCGTTCCCCC

91103 91123 90924 92609 CDS product deoxyribonuclease 91103-91123 4:01:01 CCCGCGCGCCTGTACCTGCCC

91174 91191 90924 92609 CDS product deoxyribonuclease 91174-91191 4:01:01 CCGGAGCAGCCCCCTGCC

91225 91256 90924 92609 CDS product deoxyribonuclease 91225-91256 6:03:01 CCGCCTGGCCTACATCCACGACCTGGTGGGCC

91249 91292 90924 92609 CDS product deoxyribonuclease 91249-91292 9:06:02 GGTGGGCCGGATGGAGCTGGAGGGGCTAGTGGGCCGTGGAACGG

91296 91319 90924 92609 CDS product deoxyribonuclease 91296-91319 5:02:01 CCGCCCTGGTGGGCCTCTCCGGCC

91303 91324 90924 92609 CDS product deoxyribonuclease 91303-91324 4:01:01 GGTGGGCCTCTCCGGCCGCGGG

91461 91478 90924 92609 CDS product deoxyribonuclease 91461-91478 4:01:01 GGCGCGGGCTGGCTACGG

91491 91507 90924 92609 CDS product deoxyribonuclease 91491-91507 4:01:01 GGTGGGGGGCTTGCGGG

91603 91622 90924 92609 CDS product deoxyribonuclease 91603-91622 4:01:01 GGCCAGGTCGCTGGTGTCGG
91669 91687 90924 92609 CDS product deoxyribonuclease 91669-91687 4:01:01 GGACCCGCGGGGTGGCCGG
91870 91893 90924 92609 CDS product deoxyribonuclease 91870-91893 4:01:01 CCGGGCCAAGTACCTCTTCGACCC
91926 91958 90924 92609 CDS product deoxyribonuclease 91926-91958 6:03:01
CCAACCTCCAAAAGCACCGCAGCGCCGCGTGCC
92009 92029 90924 92609 CDS product deoxyribonuclease 92009-92029 4:01:01 CCAGGCCGCGTCCCCGGCGCC
92024 92042 90924 92609 CDS product deoxyribonuclease 92024-92042 4:01:01 GGCGCCGCGGAGGCGCTGG
92205 92237 90924 92609 CDS product deoxyribonuclease 92205-92237 5:02:01
GGGACACTGGCGAGTTGGCGCTGTCGGTCCCGG
92361 92377 90924 92609 CDS product deoxyribonuclease 92361-92377 4:01:01 GGCGGTGCGAGGAGGGG
92437 92457 90924 92609 CDS product deoxyribonuclease 92437-92457 4:01:01 CCACCCCAGCTGCGCCGTTCC
92479 92510 90924 92609 CDS product deoxyribonuclease 92479-92510 5:02:01 GGTTATTGTGGACCGCGAGGGTTGCTGGGAGG
92676 92696 92561 92785 CDS product myristylated tegument protein 92676-92696 5:02:01
CCTTCCGGCAGACCCCCGGCC
92754 92777 92561 92785 CDS product myristylated tegument protein 92754-92777 5:02:01
CCCCCGACCCAAGCGCCGCGACCC
93062 93088 92993 94357 CDS product envelope glycoprotein M 93062-93088 5:02:01 CCCCTGACCGGGTACCGGTCTTCCGCC
93115 93136 92993 94357 CDS product envelope glycoprotein M 93115-93136 4:01:01 GGTCTCGTAGGCGTCGGAGAGG
93324 93344 92993 94357 CDS product envelope glycoprotein M 93324-93344 4:01:01 CCACGGCCCCCATGACGGCCC
93651 93675 92993 94357 CDS product envelope glycoprotein M 93651-93675 4:01:01 GGTTGGCGACAACGGCGCGCCAGGG
93687 93707 92993 94357 CDS product envelope glycoprotein M 93687-93707 4:01:01 GGTGCATGGCGCTGCTGGTGG
93781 93812 92993 94357 CDS product envelope glycoprotein M 93781-93812 6:03:01
GGCAAGGGCGAGGAAGTGGGCGATGTAGGCGG

| | | | | | | |
|---|---|---|---|---|---|---|
| 93871 | 93896 | 92993 | 94357 | CDS product envelope glycoprotein M | 93871-93896 5:02:01 | GGCGGACAGGGTGCCCAGGGTGATGG |
| 93949 | 93967 | 92993 | 94357 | CDS product envelope glycoprotein M | 93949-93967 4:01:01 | GGAGGAGCCGCCGGTTTGG |
| 94044 | 94064 | 92993 | 94357 | CDS product envelope glycoprotein M | 94044-94064 5:02:01 | GGAAGGCTGCCGTGGTGGCGG |
| 94086 | 94123 | 92993 | 94357 | CDS product envelope glycoprotein M | 94086-94123 7:04:01 | CCACTCCGCCAGTTATCCGATGCATCCACACCCCGTCC |
| 94292 | 94315 | 92993 | 94357 | CDS product envelope glycoprotein M | 94292-94315 5:02:01 | CCCTCTCCAGCCGCGCCCGGCACC |
| 94547 | 94567 | 94278 | 96938 | CDS product DNA replication origin-binding helicase | 94547-94567 4:01:01 | CCTCGGCCTCGGGATCCCACC |
| 94980 | 95003 | 94278 | 96938 | CDS product DNA replication origin-binding helicase | 94980-95003 4:01:01 | CCATGGACGCCACGGCCAACGCCC |
| 95507 | 95525 | 94278 | 96938 | CDS product DNA replication origin-binding helicase | 95507-95525 4:01:01 | GGCCCGGACATGGTGTCGG |
| 95592 | 95610 | 94278 | 96938 | CDS product DNA replication origin-binding helicase | 95592-95610 4:01:01 | CCGGGGCCAGGCCCGAGCC |
| 95830 | 95861 | 94278 | 96938 | CDS product DNA replication origin-binding helicase | 95830-95861 6:03:01 | CCTGCACACCCTCCTCGAGTCCAACCGCGTCC |
| 95965 | 96004 | 94278 | 96938 | CDS product DNA replication origin-binding helicase | 95965-96004 8:05:02 | CCAGCTCCGGGAGCCCGGCCCGCCCGCGGCCACCACTACC |
| 96055 | 96092 | 94278 | 96938 | CDS product DNA replication origin-binding helicase | 96055-96092 6:03:01 | GGCCGACGTGGCCTGCGAGGACATCAGGGGGCTCTTGG |
| 96093 | 96116 | 94278 | 96938 | CDS product DNA replication origin-binding helicase | 96093-96116 4:01:01 | CCGAGCTCCACAGCCCCGTCGTCC |
| 96359 | 96374 | 94278 | 96938 | CDS product DNA replication origin-binding helicase | 96359-96374 4:01:01 | |

GGGGGATCGGCCTCGG

96667 96701 94278 96938 CDS product DNA replication origin-binding helicase 96667-96701 7:04:01 GGAGGAGATAGTGGGGCTTTTGGAGGCGGCAGGGG

96892 96914 94278 96938 CDS product DNA replication origin-binding helicase 96892-96914 5:02:01 GGCGTGGCCACAGGCCCAGGGGG

97046 97067 96983 99238 CDS product helicase-primase subunit 97046-97067 4:01:01 GGACGGACCCGGCCGAGCCGGG

97053 97102 96983 99238 CDS product helicase-primase subunit 97053-97102 10:07:02 CCCGGCCGAGCCGGGCGCCCTCCAGGCCCTGGTCCACCTGCTCTGCCGCC

97195 97215 96983 99238 CDS product helicase-primase subunit 97195-97215 4:01:01 CCCGCCCGCGCGGCCGCGGCC

97206 97225 96983 99238 CDS product helicase-primase subunit 97206-97225 4:01:01 GGCCGCGGCCGGGCACAGGG

97246 97269 96983 99238 CDS product helicase-primase subunit 97246-97269 5:02:01 CCGCTCGCCGCCCTGGGCCAGGCC

97313 97328 96983 99238 CDS product helicase-primase subunit 97313-97328 4:01:01 GGCGGACCCTGGGCGG

97350 97399 96983 99238 CDS product helicase-primase subunit 97350-97399 8:05:02 GGCGCTTGGGGTAGACCCGGCCACCGGGCTTTTGGTGGCGGCCGCGCCGG

97391 97413 96983 99238 CDS product helicase-primase subunit 97391-97413 4:01:01 CCGCGCCGGCGCCCGCGCGGGCC

97409 97440 96983 99238 CDS product helicase-primase subunit 97409-97440 6:03:01 GGGCCGGCGCGGCAGAGGAGCCGGGCCCGCGG

97429 97446 96983 99238 CDS product helicase-primase subunit 97429-97446 4:01:01 CCGGGCCCGCGGCCCGCC

97460 97481 96983 99238 CDS product helicase-primase subunit 97460-97481 4:01:01 CCGCCGAGGTGCCCCTGGACCC

97467 97504 96983 99238 CDS product helicase-primase subunit 97467-97504 7:04:01 GGTGCCCCTGGACCCGGCGGCGGTTAGGGCCCACGCGG

97778 97806 96983 99238 CDS product helicase-primase subunit 97778-97806 6:03:01 CCAGCTCCCAGGCCGCGTCCGGCCTGGCC

97868 97896 96983 99238 CDS product helicase-primase subunit 97868-97896 5:02:01 CCGTGCCCGTCTTCGCCTACCTCGGCCCC
97970 97992 96983 99238 CDS product helicase-primase subunit 97970-97992 5:02:01 CCGCCTCGACCACTCCCGAGGCC
98018 98037 96983 99238 CDS product helicase-primase subunit 98018-98037 4:01:01 GGTTTGCGGACGGCCTCTGG
98072 98099 96983 99238 CDS product helicase-primase subunit 98072-98099 4:01:01 CCCCGTGGGGGCCGGGGGACCGCTGGCC
98078 98108 96983 99238 CDS product helicase-primase subunit 98078-98108 7:04:01 GGGGGCCGGGGGACCGCTGGCCTGGCGAGGG
98117 98142 96983 99238 CDS product helicase-primase subunit 98117-98142 5:02:01 GGCGGCTGGCTGCGACGGCGTCCCGG
98160 98178 96983 99238 CDS product helicase-primase subunit 98160-98178 4:01:01 GGACGCGTGGCCGGCAGGG
98196 98226 96983 99238 CDS product helicase-primase subunit 98196-98226 5:02:01 GGAGTCGCCGGCGGCGCTGCAGGGGCCCTGG
98268 98301 96983 99238 CDS product helicase-primase subunit 98268-98301 6:03:01 CCTGGCCCTGTTTCCGAACCACGCGCGCCTGGCC
98336 98353 96983 99238 CDS product helicase-primase subunit 98336-98353 4:01:01 CCGCCCTGAAGCCTGCCC
98382 98406 96983 99238 CDS product helicase-primase subunit 98382-98406 5:02:01 CCTGGACCCCGCGGCCTACCGGGCC
98413 98459 96983 99238 CDS product helicase-primase subunit 98413-98459 7:04:01 GGCCTGGCCAACGGGATAAGCCGGCGCCTGGAGCGCGAGGCAAACGG
98643 98665 96983 99238 CDS product helicase-primase subunit 98643-98665 4:01:01 CCTGCGCCTGCGCCTCGAGGGCC
98678 98695 96983 99238 CDS product helicase-primase subunit 98678-98695 4:01:01 CCGTCTCCTGGTCCACCC
98717 98744 96983 99238 CDS product helicase-primase subunit 98717-98744 4:01:01

GGGAGACTGGCATTGTGGACTTTGTGGG
98749 98814 96983 99238 CDS product helicase-primase subunit 98749-98814 13:10:03
CCCGCCAAGAGCCGCGCCGGAAAGGCCGCCAAGTCCAGCCTGGCGAGCCTCCTTGCCGCCGCTGCC
98850 98876 96983 99238 CDS product helicase-primase subunit 98850-98876 5:02:01
GGCGGCGGCGCGCGAGGCGTGCGAGGG
98889 98922 96983 99238 CDS product helicase-primase subunit 98889-98922 5:02:01
GGCGTTTGCGGAGCGGGGCAACCCGGAGTTTTGG
98948 98964 96983 99238 CDS product helicase-primase subunit 98948-98964 5:02:01 CCCCCCTCCCCCCGGCC
98974 99002 96983 99238 CDS product helicase-primase subunit 98974-99002 5:02:01
GGAGGGGCCGTGCTGGACCTGGACCGCGG
99110 99123 96983 99238 CDS product helicase-primase subunit 99110-99123 4:01:01 CCCACCTAACCCCC
99606 99632 99332 100267 CDS product tegument protein UL7 99606-99632 5:02:01 GGCTCTGGTCAAAGGGGCTGTGGCTGG
99825 99838 99332 100267 CDS product tegument protein UL7 99825-99838 4:01:01 GGTTGGTGGGCCGG
99933 99952 99332 100267 CDS product tegument protein UL7 99933-99952 5:02:01 GGAAGGCGGGCAGGTCCAGG
100079 100096 99332 100267 CDS product tegument protein UL7 100079-100096 4:01:01 CCGTCGACCTCCCGGACC
100209 100228 99332 100267 CDS product tegument protein UL7 100209-100228 4:01:01 CCAGGGGATCCAGCCCCTCC
100489 100505 100044 102290 CDS product capsid portal protein 100489-100505 4:01:01 GGTGGCGGGCCCGCAGG
100651 100669 100044 102290 CDS product capsid portal protein 100651-100669 4:01:01 GGTCGGCAGCGTAGGGGGG
100738 100766 100044 102290 CDS product capsid portal protein 100738-100766 6:03:01
CCTCGTGTCCCAAGCCGTCCGCGGCCGCC
100795 100843 100044 102290 CDS product capsid portal protein 100795-100843 8:05:02
CCGCCTGCGAAGCCTGGACCAGGCGCTCCCTGGCCCGGGCCAGCTCACC
100978 100995 100044 102290 CDS product capsid portal protein 100978-100995 4:01:01 GGACCGGCTGGGCGCCGG

100992 101026 100044 102290 CDS product capsid portal protein 100992-101026 5:02:01 CCGGCAGCCCGTGGGCGCCCGCTCTGTCCGAAGCC
101041 101057 100044 102290 CDS product capsid portal protein 101041-101057 4:01:01 CCACCTGCGATCCGCCC
101209 101243 100044 102290 CDS product capsid portal protein 101209-101243 8:05:02 CCACCGCCTTGGACGCCCACCCAGGCCCCCTGTCC
101621 101644 100044 102290 CDS product capsid portal protein 101621-101644 6:03:01 GGGGGGTGGGGCGTCTGGGGGGGG
101661 101680 100044 102290 CDS product capsid portal protein 101661-101680 5:02:01 GGGTGGACGGCGGATCCCGG
101741 101772 100044 102290 CDS product capsid portal protein 101741-101772 5:02:01 GGCGAAGCTGGCGGTGCCGTCGGGCTCGCTGG
101860 101876 100044 102290 CDS product capsid portal protein 101860-101876 4:01:01 CCCGCACCGCCTCGGCC
101926 101940 100044 102290 CDS product capsid portal protein 101926-101940 4:01:01 GGCCGGACAGGTGGG
101983 102021 100044 102290 CDS product capsid portal protein 101983-102021 8:05:02 GGGTGTTGGTTAGGATGGTCGTCTGGATCTGGCGGATGG
102022 102036 100044 102290 CDS product capsid portal protein 102022-102036 4:01:01 CCGCCTCCGTGGACC
102133 102146 100044 102290 CDS product capsid portal protein 102133-102146 4:01:01 CCCATCCCCCGTCC
102160 102184 100044 102290 CDS product capsid portal protein 102160-102184 4:01:01 CCTGGTCATCCGCGTCCGCGCGGCC
102209 102234 100044 102290 CDS product capsid portal protein 102209-102234 5:02:01 CCGCTTCCGCTCCCGCCCGGGCACCC
102262 102279 100044 102290 CDS product capsid portal protein 102262-102279 4:01:01 GGGGGGGAGGCCGCATGG
102294 102319 102277 104922 CDS product helicase-primase helicase subunit 102294-102319 5:02:01 GGCGAGGGGCGCGACGGCCGCGGCGG
102372 102396 102277 104922 CDS product helicase-primase helicase subunit 102372-102396 4:01:01

GGCGCGGAGTCTGAGGATTACTCGG

102458 102476 102277 104922 CDS product helicase-primase helicase subunit 102458-102476 4:01:01

CCGCATCCGGGCCCTGGCC

102466 102486 102277 104922 CDS product helicase-primase helicase subunit 102466-102486 4:01:01

GGGCCCTGGCCGGAAAGACGG

102489 102508 102277 104922 CDS product helicase-primase helicase subunit 102489-102508 5:02:01

CCCGCCGCCCGCGTGCCGCC

102666 102684 102277 104922 CDS product helicase-primase helicase subunit 102666-102684 4:01:01

GGGGCGACGCGGGTGGCGG

102761 102783 102277 104922 CDS product helicase-primase helicase subunit 102761-102783 4:01:01

CCGCGGAAACCACGTCCAGGCCC

102814 102827 102277 104922 CDS product helicase-primase helicase subunit 102814-102827 4:01:01

CCAACCCCCCTCCC

102969 102987 102277 104922 CDS product helicase-primase helicase subunit 102969-102987 4:01:01

GGGTCGCTGGCCGGGCTGG

103051 103074 102277 104922 CDS product helicase-primase helicase subunit 103051-103074 5:02:01

CCGGCCTGCTGGGCCGACACCTCC

103451 103466 102277 104922 CDS product helicase-primase helicase subunit 103451-103466 4:01:01

CCCCGCCAACCTACCC

103538 103553 102277 104922 CDS product helicase-primase helicase subunit 103538-103553 4:01:01

GGTATCCGGGGGGGGG

103748 103770 102277 104922 CDS product helicase-primase helicase subunit 103748-103770 6:03:01

GGGGGTGGTGGTGGCGCGAACGG

104092 104103 102277 104922 CDS product helicase-primase helicase subunit 104092-104103 4:01:01 GGGAGGAGGGGG
104141 104175 102277 104922 CDS product helicase-primase helicase subunit 104141-104175 6:03:01 GGCGGCCGGGGACGACTGGGGCGAGGACGACGTGG
104522 104546 102277 104922 CDS product helicase-primase helicase subunit 104522-104546 4:01:01 GGGTATCGCGGAGCTCCTGGGCGGG
104966 104985 104945 105661 CDS product nuclear protein UL4 104966-104985 5:02:01 CCGCGGCCTCCCCCGTAACC
105277 105293 104945 105661 CDS product nuclear protein UL4 105277-105293 4:01:01 GGCTGGGTGGCCGTGGG
105412 105433 104945 105661 CDS product nuclear protein UL4 105412-105433 4:01:01 GGGTCCAGCGGCGAGGAACCGG
105430 105447 104945 105661 CDS product nuclear protein UL4 105430-105447 4:01:01 CCGGAGCCCGCCTGCCCC
105492 105514 104945 105661 CDS product nuclear protein UL4 105492-105514 5:02:01 CCGTGCCCCCGAGACCGAGGCCC
105556 105592 104945 105661 CDS product nuclear protein UL4 105556-105592 7:04:01 GGGTTTGGGGGTTCTGCGGACTGGCCCGCGGCAGCGG
105809 105858 105782 106288 CDS product protein V57 105809-105858 9:06:02 GGCCTATGGTGGGTTCCGGGGTTGGGGTTAGAGGCGGGGTAGGGGCGCGG
105894 105909 105782 106288 CDS product protein V57 105894-105909 4:01:01 CCTCCTCTGCCGCCCC
105975 106006 105782 106288 CDS product protein V57 105975-106006 6:03:01 GGTCGCCGGCTCGGGTTGTGGACTTTCGGTGG
106084 106102 105782 106288 CDS product protein V57 106084-106102 4:01:01 CCTATCGCCGCCGGCTGCC
106135 106155 105782 106288 CDS product protein V57 106135-106155 4:01:01 CCGTGCCGGGCGCCCGGGGCC
106167 106183 105782 106288 CDS product protein V57 106167-106183 4:01:01 GGGCGTGCGGCGGCTGG
106631 106656 106335 106964 CDS product nuclear protein UL3 106631-106656 6:03:01 GGAGGAGGTTTTGGTGCAGGCAATGG
106839 106876 106335 106964 CDS product nuclear protein UL3 106839-106876 7:04:01 GGGGAAGCGCCGGGCGGGCCGGCAAAAGCGGACGGCGG
106898 106914 106335 106964 CDS product nuclear protein UL3 106898-106914 4:01:01 GGCCGCGGCCTGGGCGG

106921 106939 106335 106964 CDS product nuclear protein UL3 106921-106939 5:02:01 CCGCCCACCCCCACTCCCC

107091 107108 107048 107995 CDS product uracil-DNA glycosylase 107091-107108 4:01:01 GGTAGGCGTTGGCGTCGG

107145 107165 107048 107995 CDS product uracil-DNA glycosylase 107145-107165 4:01:01 GGGGCGACGGGTGGCCGTAGG

107311 107332 107048 107995 CDS product uracil-DNA glycosylase 107311-107332 4:01:01 CCCCGGCTGTCCGCGCCGCACC

107358 107388 107048 107995 CDS product uracil-DNA glycosylase 107358-107388 5:02:01 CCCCCTGGAGGGCCCACTTTTCCAGGTAGCC

107382 107401 107048 107995 CDS product uracil-DNA glycosylase 107382-107401 4:01:01 GGTAGCCGTGGGCGGGGAGG

107448 107463 107048 107995 CDS product uracil-DNA glycosylase 107448-107463 4:01:01 GGCTTGGGGGAACTGG

107487 107511 107048 107995 CDS product uracil-DNA glycosylase 107487-107511 5:02:01 CCAGCCCGTGCGCCTGGCCGGGGCC

107502 107527 107048 107995 CDS product uracil-DNA glycosylase 107502-107527 5:02:01 GGCCGGGGCCGTGGTAGGGGTCCTGG

107589 107611 107048 107995 CDS product uracil-DNA glycosylase 107589-107611 5:02:01 CCTTTCCCGGCAGAACCTCCTCC

107679 107699 107048 107995 CDS product uracil-DNA glycosylase 107679-107699 5:02:01 GGATGGAGCGCCAGGCGGGGG

107718 107736 107048 107995 CDS product uracil-DNA glycosylase 107718-107736 5:02:01 CCAGCTCCTCCCAGCCCCC

107876 107985 107048 107995 CDS product uracil-DNA glycosylase 107876-107985 22:19:05 GGCGGGCGCTTGGGGGGCGGGGGCGGCGCGGCGGGCGTTGCGGTTCCAGGCGGGGAAGGCGGGGCGGCCGCCGGCGGCTCAGGGCTTTTTTGGGGCGGGCGCGGCGTCGG

108311 108327 107955 108593 CDS product envelope glycoprotein L 108311-108327 4:01:01 GGGGGGGGCGCAGTTGG

108430 108444 107955 108593 CDS product envelope glycoprotein L 108430-108444 4:01:01 CCGCCCTGGGCCCCC

108479 108494 107955 108593 CDS product envelope glycoprotein L 108479-108494 4:01:01 GGAGGCCGGCCAAGGG

108484 108503 107955 108593 CDS product envelope glycoprotein L 108484-108503 4:01:01 CCGGCCAAGGGCCCGCGACC

108550 108564 107955 108593 CDS product envelope glycoprotein L 108550-108564 4:01:01 CCCCCAGCTCCCCCC

113705 113718 113644 115263 CDS product ubiquitin E3 ligase ICP0 113705-113718 4:01:01 GGAAAGGCGGCCGG

113752 113782 113644 115263 CDS product ubiquitin E3 ligase ICP0 113752-113782 5:02:01 GGGCGCCGGGCGACCGGGTAAGGGGTTGGGG
113790 113851 113644 115263 CDS product ubiquitin E3 ligase ICP0 113790-113851 12:09:03 CCTCCCGGCACCACGCACCACCCCGCCGCGTAGCCTGGCCGCCCTCTCCTCGGTCCAGCGCC
113860 113889 113644 115263 CDS product ubiquitin E3 ligase ICP0 113860-113889 6:03:01 CCCTTCAACCGGCGCCTGGCGCCCCCCGCC
113892 113921 113644 115263 CDS product ubiquitin E3 ligase ICP0 113892-113921 6:03:01 GGCGGGGGGCGCGGTCCCGCCGGGCTGAGG
113922 113938 113644 115263 CDS product ubiquitin E3 ligase ICP0 113922-113938 4:01:01 CCGAAAACCCTCCACCC
114019 114054 113644 115263 CDS product ubiquitin E3 ligase ICP0 114019-114054 6:03:01 CCGACGCCGTACCTCGGCCCGCGTCCGGGGGAACCC
114060 114107 113644 115263 CDS product ubiquitin E3 ligase ICP0 114060-114107 10:07:02 GGGGGGGGCAGGGGCTGCGGGGGCAGAGGTTTTGGGCGCGCCGGTGGG
114117 114138 113644 115263 CDS product ubiquitin E3 ligase ICP0 114117-114138 4:01:01 GGAAGTGCGGGCAAGGGGCGGG
114167 114189 113644 115263 CDS product ubiquitin E3 ligase ICP0 114167-114189 4:01:01 CCGTAAACGCCTGCCCGGGGGCC
114183 114248 113644 115263 CDS product ubiquitin E3 ligase ICP0 114183-114248 14:11:03 GGGGGCCGGGGTGGCTCGCGGGCCCGGGCTGGGTAGGCGGCCACCCCGGGCCGGGCAGGCGGCCGG
114414 114431 113644 115263 CDS product ubiquitin E3 ligase ICP0 114414-114431 5:02:01 GGCCCTGGCCGGCGGGGG
114500 114520 113644 115263 CDS product ubiquitin E3 ligase ICP0 114500-114520 4:01:01 GGTGGACGAGCGGCGAGTGGG
114650 114668 113644 115263 CDS product ubiquitin E3 ligase ICP0 114650-114668 4:01:01 CCACGCCGCGCGCCCGGCC

114665 114694 113644 115263 CDS product ubiquitin E3 ligase ICP0 114665-114694 6:03:01
GGCCGGGGTGCGGCACAGCGGCGGGGCCGG
114705 114725 113644 115263 CDS product ubiquitin E3 ligase ICP0 114705-114725 5:02:01
CCGGGCCTCGGCCACCAGCCC
114909 114931 113644 115263 CDS product ubiquitin E3 ligase ICP0 114909-114931 4:01:01
GGGCGCCCCGGCGGTTCCGTTGG
114997 115015 113644 115263 CDS product ubiquitin E3 ligase ICP0 114997-115015 4:01:01 GGGGGAGTAGGCGTGAGGG
115043 115071 113644 115263 CDS product ubiquitin E3 ligase ICP0 115043-115071 5:02:01
CCACGGGCCCTCCGTTTACCGCGGTTTCC
115166 115196 113644 115263 CDS product ubiquitin E3 ligase ICP0 115166-115196 5:02:01
GGCATACGTGGCAGAAGCGGTGGAGACACGG
115236 115257 113644 115263 CDS product ubiquitin E3 ligase ICP0 115236-115257 4:01:01
GGCACACGGGTTTTCCTGGGGG
118353 118377 118285 122433 CDS product transcriptional regulator ICP4 118353-118377 4:01:01
GGGCCCGCGGGCCCCGGCGCCGGGG
118356 118400 118285 122433 CDS product transcriptional regulator ICP4 118356-118400 7:04:01
CCCGCGGGCCCCGGCGCCGGGGCGCCCGCTGCCGCCCGCGGGCCC
118437 118458 118285 122433 CDS product transcriptional regulator ICP4 118437-118458 5:02:01
CCCGCCGGCGCCGTCCGTCTCC
118505 118527 118285 122433 CDS product transcriptional regulator ICP4 118505-118527 5:02:01
CCACGGCCCCGGCCGCCAGCACC
118607 118644 118285 122433 CDS product transcriptional regulator ICP4 118607-118644 6:03:01
CCGTCGGGCCGGCCTCGAGGTCCGGCTCCAGCAGCGCC

118612 118631 118285 122433 CDS product transcriptional regulator ICP4 118612-118631 4:01:01
GGGCCGGCCTCGAGGTCCGG
118668 118696 118285 122433 CDS product transcriptional regulator ICP4 118668-118696 6:03:01
GGGCGGCAGCAGGTCCGCGGGCGCGGCGG
118703 118728 118285 122433 CDS product transcriptional regulator ICP4 118703-118728 4:01:01
CCAGCGCGCCGCGGCGGCCGGCGGCC
118715 118748 118285 122433 CDS product transcriptional regulator ICP4 118715-118748 7:04:01
GGCGGCCGGCGGCCAGGTAGAGCGGACGCAGCGG
118766 118782 118285 122433 CDS product transcriptional regulator ICP4 118766-118782 4:01:01
GGTTGGCCGCGCGGTGG
118821 118840 118285 122433 CDS product transcriptional regulator ICP4 118821-118840 4:01:01
GGCCAGGCCCCGGCTCTGGG
118883 118907 118285 122433 CDS product transcriptional regulator ICP4 118883-118907 5:02:01
GGCGGTAGTCCCGGGGCGGCATGGG
118937 118959 118285 122433 CDS product transcriptional regulator ICP4 118937-118959 4:01:01
CCACCACGTAGGCCACGTTGCCC
119157 119174 118285 122433 CDS product transcriptional regulator ICP4 119157-119174 4:01:01
GGCCGGGCCGTCGGCGGG
119211 119229 118285 122433 CDS product transcriptional regulator ICP4 119211-119229 4:01:01
CCGGCGCCGGGCCGAGCCC
119249 119276 118285 122433 CDS product transcriptional regulator ICP4 119249-119276 4:01:01
CCACGCAGCCCGCGAAGGCCAGGTCCCC
119265 119281 118285 122433 CDS product transcriptional regulator ICP4 119265-119281 4:01:01

GGCCAGGTCCCCGGTGG
119329 119347 118285 122433 CDS product transcriptional regulator ICP4 119329-119347 4:01:01
CCGGTCCAGTTGCCGGCCC
119434 119459 118285 122433 CDS product transcriptional regulator ICP4 119434-119459 6:03:01
CCGCCCTCGCGGCCCGCCGCGCCGCC
119470 119489 118285 122433 CDS product transcriptional regulator ICP4 119470-119489 4:01:01
CCGCCCAGCAGGTCCTCGCC
119490 119508 118285 122433 CDS product transcriptional regulator ICP4 119490-119508 5:02:01
GGGCAGGGGGTGGTAGAGG
119553 119584 118285 122433 CDS product transcriptional regulator ICP4 119553-119584 7:04:01
GGCGGCGCGGCGGCGCAGCGGGCCGCTGGGGG
119605 119632 118285 122433 CDS product transcriptional regulator ICP4 119605-119632 4:01:01
CCCTCGCGGGCCGGGGGCCCCGTGCGCC
119633 119678 118285 122433 CDS product transcriptional regulator ICP4 119633-119678 8:05:02
GGGCGGCGACGGTCGCCAGGGCGGCCGGGTCGAAGGTGAGCGCGGG
119679 119691 118285 122433 CDS product transcriptional regulator ICP4 119679-119691 4:01:01 CCGCCATGCCTCC
119718 119778 118285 122433 CDS product transcriptional regulator ICP4 119718-119778 10:07:02
GGCCACGGTCTCGGGGGAGCAGTAGGCCGCGCGGGCGGCCTCGCCGGGCGCCGGCGTGTGG
119813 119905 118285 122433 CDS product transcriptional regulator ICP4 119813-119905 18:15:04
GGGGGTCGGGGCCGCCGGCGGGCATGGGGCCCAGGGCGCGAGGCGGGGGGGCGCCGGGCCGGCGCTTGGGTGCCGGCGGGGTGGCGGCCCCGG
119937 119958 118285 122433 CDS product transcriptional regulator ICP4 119937-119958 5:02:01
GGGGCGCGGCGGCTCGGAGCGG
119964 119983 118285 122433 CDS product transcriptional regulator ICP4 119964-119983 4:01:01

CCCCCGCTGCCTGGGGCGCC

119988 120046 118285 122433 CDS product transcriptional regulator ICP4 119988-120046 10:07:02

GGGAAGGCGTCCCAGGCAGCGGGGGGACGCGGCCTCCGAGGCCTCGGGGAGGCCCCGGG

120055 120084 118285 122433 CDS product transcriptional regulator ICP4 120055-120084 8:05:02

GGGGAGGCGCAGGGGGCGGCGGGGGCCGGG

120094 120177 118285 122433 CDS product transcriptional regulator ICP4 120094-120177 17:14:04

GGGGACGGGGCCCGGTGCGCGGCCGGGCTCGGAGGGCCCGGCGGCTGGGGCTGCGGCGGGTGGGCCCACAGGGGCGGCAGGTGG

120208 120244 118285 122433 CDS product transcriptional regulator ICP4 120208-120244 6:03:01

GGCCGGGGCGCGCCGGGGAGCGCGGCGACGGCCTCGG

120239 120261 118285 122433 CDS product transcriptional regulator ICP4 120239-120261 5:02:01

CCTCGGCCATCTGCCGGGCCGCC

120293 120313 118285 122433 CDS product transcriptional regulator ICP4 120293-120313 4:01:01

GGGTCCAGGCCGAGGCGCGGG

120328 120355 118285 122433 CDS product transcriptional regulator ICP4 120328-120355 4:01:01

GGGTTGTACCGGACGAGCGGGGCGACGG

120418 120442 118285 122433 CDS product transcriptional regulator ICP4 120418-120442 4:01:01

CCGGGGTCCTGCGGGCCCAGGAGCC

120477 120507 118285 122433 CDS product transcriptional regulator ICP4 120477-120507 7:04:01

GGCCGCGGCGGCGGCCCGGAGGCCGCCGGGG

120491 120512 118285 122433 CDS product transcriptional regulator ICP4 120491-120512 4:01:01

CCCGGAGGCCGCCGGGGTTGCC

120518 120546 118285 122433 CDS product transcriptional regulator ICP4 120518-120546 6:03:01

GGGCCTCCGGGGGGCAGGCGGGCGTCGGG

120555 120580 118285 122433 CDS product transcriptional regulator ICP4 120555-120580 6:03:01
GGGCTGGAGGCCGGGGACGGCCGCGG
120575 120595 118285 122433 CDS product transcriptional regulator ICP4 120575-120595 4:01:01
CCGCGGCGCCTCCGCGGAGCC
120615 120637 118285 122433 CDS product transcriptional regulator ICP4 120615-120637 5:02:01
GGCGTGGCAGGCGAGGACGCAGG
120641 120668 118285 122433 CDS product transcriptional regulator ICP4 120641-120668 6:03:01
CCCCCAGCTCCTTGACCCGGGCGCCCCC
120678 120715 118285 122433 CDS product transcriptional regulator ICP4 120678-120715 6:03:01
CCGCGCCGCCCCGGGCTCCCCGGGCTCCCCGGGCGCCC
120690 120717 118285 122433 CDS product transcriptional regulator ICP4 120690-120717 4:01:01
GGGCTCCCCGGGCTCCCCGGGCGCCCGG
120764 120820 118285 122433 CDS product transcriptional regulator ICP4 120764-120820 11:08:02
GGGCCCGGGCCTGCGGCGGGGAGCGCGGCGCCCCGGCCGTCGGGGAGGAGGAGGAGG
120830 120886 118285 122433 CDS product transcriptional regulator ICP4 120830-120886 13:10:03
GGGGCGAGGGGGCGGCGGGTCCGGGGCCCGGCGGCGAGCCGGGCTCGGCGGCGGGGG
120900 120913 118285 122433 CDS product transcriptional regulator ICP4 120900-120913 4:01:01 GGGCCGGGGGAAGG
120927 120948 118285 122433 CDS product transcriptional regulator ICP4 120927-120948 4:01:01
GGCCCGGCGCAGGCTCTGCAGG
120968 120997 118285 122433 CDS product transcriptional regulator ICP4 120968-120997 5:02:01
GGTCGTATCGGCGGCTCATGGCCACCGAGG
120989 121002 118285 122433 CDS product transcriptional regulator ICP4 120989-121002 4:01:01 CCACCGAGGCCGCC
121234 121251 118285 122433 CDS product transcriptional regulator ICP4 121234-121251 4:01:01

CCCGAGTCCCCCATCTCC

121263 121282 118285 122433 CDS product transcriptional regulator ICP4 121263-121282 4:01:01

GGGCACGGGCCCGCCGGCGG

121322 121355 118285 122433 CDS product transcriptional regulator ICP4 121322-121355 6:03:01

CCCAGAGCCCCTCCCGCGAGTCCCCGGTGCCCCC

121367 121412 118285 122433 CDS product transcriptional regulator ICP4 121367-121412 9:06:02

GGCCCTTGGGCGGCGGGTCGGAGCCCGGCCAGGGGTCCCCGGCCGG

121390 121410 118285 122433 CDS product transcriptional regulator ICP4 121390-121410 4:01:01

CCCGGCCAGGGGTCCCCGGCC

121421 121492 118285 122433 CDS product transcriptional regulator ICP4 121421-121492 12:09:03

GGGGCTCCGCGGGGCCGGCCGCGGGCTCCCGGGCGGCGTCGGGGCCCGGGCCCGAGGGGCGGCCGGCGCCGG

121427 121450 118285 122433 CDS product transcriptional regulator ICP4 121427-121450 4:01:01

CCGCGGGGCCGGCCGCGGGCTCCC

121516 121553 118285 122433 CDS product transcriptional regulator ICP4 121516-121553 8:05:02

GGCTGCTGGGGCAGGGGCGGCGAGGAGGGCTGCGGCGG

121568 121638 118285 122433 CDS product transcriptional regulator ICP4 121568-121638 13:10:03

GGGGCGAGGACCGATCGGCGGGCGGGGACGGGGTTCGCGGGGCGGCCTGGAGGAACGCGGGCACCGGCCGG

121653 121723 118285 122433 CDS product transcriptional regulator ICP4 121653-121723 14:11:03

GGAGGCCCCCGAGGGCGGCGGCGGGCCCCCGAGGGCGGCGGGCGGCGGGGCCCGCGGGGAGGGCGAAGGGG

121796 121817 118285 122433 CDS product transcriptional regulator ICP4 121796-121817 4:01:01

GGTCTCCGGGCGGGAGAGCCGG

121827 121846 118285 122433 CDS product transcriptional regulator ICP4 121827-121846 6:03:01

GGGCGGCGGGGGACGGCGGG

121857  121879  118285  122433  CDS product transcriptional regulator ICP4  121857-121879  5:02:01
GGACGGGGCGGGCGCGGCGTCGG
121893  122014  118285  122433  CDS product transcriptional regulator ICP4  121893-122014  24:21:06
GGCGGAGTCCGAGGCGGAGGAGCCAGAGGAGGAGGACGCGGACGAGGACGAGGAGCAGGAGGCGCTCCGGGCGCGCGGGGGCCTCGACGGCGGGGAGGCGGACGCGGGGAGCGGAGGGGTGG
122050  122091  118285  122433  CDS product transcriptional regulator ICP4  122050-122091  7:04:01
CCGTCCCCTTCGACCTCCGTGGTCTCCATCTCGGCCCGGACC
122113  122136  118285  122433  CDS product transcriptional regulator ICP4  122113-122136  5:02:01
CCCGCCGGCTCCCCGCCGGGGGCC
122130  122192  118285  122433  CDS product transcriptional regulator ICP4  122130-122192  13:10:03
GGGGGCCGGCGAGGGCACCGTCGGCGGGGCGGGCGGCTTGGTGGGCGGCGCGGCCTGCTTGGG
122201  122254  118285  122433  CDS product transcriptional regulator ICP4  122201-122254  13:10:03
GGGGCTCCGGCGGCGGCGGAGCCGGGGAGGCGGGGATGGCGGGGATGGCGGGGG
122276  122300  118285  122433  CDS product transcriptional regulator ICP4  122276-122300  4:01:01
CCACCCCGAAGAGCCCCTGGCTCCC
122306  122326  118285  122433  CDS product transcriptional regulator ICP4  122306-122326  4:01:01
GGAGGTCGGGGTGCTGCTGGG
122337  122361  118285  122433  CDS product transcriptional regulator ICP4  122337-122361  6:03:01
GGCGGCACCGGCGGCCCGGATGAGG
126116  126133  126077  127144  CDS product regulatory protein ICP22  126116-126133  5:02:01 CCTGCCGCCTGCGCCCCC
126142  126247  126077  127144  CDS product regulatory protein ICP22  126142-126247  23:20:05
CCCGCCAGCCCCCTCATCCCGCCTCTGTCGCCGTCGCCCCCCCGCTCCGGCGCCACCGCCCCCTCCTGGCGCGCCTCCCTCTCCCCCACGCCCGGCTCCGCCCCCC
126277  126304  126077  127144  CDS product regulatory protein ICP22  126277-126304  5:02:01
GGGGGGGCGGACCACCCGGAGTACGGGG

126307 126351 126077 127144 CDS product regulatory protein ICP22 126307-126351 7:04:01
CCCGTCTCGCCCCGCGCCCTCGCCCCGCGCCTCGCCGGCGGGCCC

126390 126421 126077 127144 CDS product regulatory protein ICP22 126390-126421 7:04:01
CCGCCTCGCCGCGCGCCTGAACCGCCTCTTCC

126630 126649 126077 127144 CDS product regulatory protein ICP22 126630-126649 4:01:01 CCTCGACCCCCCCGAGCCCC

126703 126741 126077 127144 CDS product regulatory protein ICP22 126703-126741 10:07:02
CCCTCCCCGTCCTCCGCCTCGTCGTCCTCCGCCCCCTCC

126772 126784 126077 127144 CDS product regulatory protein ICP22 126772-126784 4:01:01 GGCGAGGAGGAGG

126825 126842 126077 127144 CDS product regulatory protein ICP22 126825-126842 5:02:01 GGACGGGGGCTTTGGCGG

126860 126876 126077 127144 CDS product regulatory protein ICP22 126860-126876 4:01:01 CCACCGGCCGCTGGACC

126897 126931 126077 127144 CDS product regulatory protein ICP22 126897-126931 8:05:02
CCCGCCCTCCTCCCCCGAGACGCCGCCCGTGCTCC

126964 126997 126077 127144 CDS product regulatory protein ICP22 126964-126997 8:05:02
CCCCCCTCCTCCCCCGAGACGCCGCCCGTGCTCC

127007 127021 126077 127144 CDS product regulatory protein ICP22 127007-127021 4:01:01 CCCGCCGCGCCGCCC

127030 127074 126077 127144 CDS product regulatory protein ICP22 127030-127074 10:07:02
CCCCCCTTCTCCCCGTTGCCCTCCTCCTCCCCCGAGACGCCGCCC

127097 127115 126077 127144 CDS product regulatory protein ICP22 127097-127115 4:01:01 GGAAGGCCCGCGGCCGCGG

127103 127125 126077 127144 CDS product regulatory protein ICP22 127103-127125 5:02:01
CCCGCGGCCGCGGCCGTCCCGCC

127339 127359 127330 127995 CDS product virion protein US10 127339-127359 4:01:01 CCTCGGTCTCCCCCATGGCCC

127462 127494 127330 127995 CDS product virion protein US10 127462-127494 8:05:02
CCGCCTCCGCCGAGGCCGCCGAGGCCCTGCGCC

127503  127525  127330  127995  CDS product virion protein US10    127503-127525   5:02:01 CCCCCGCCCCAGGGCCTGTGGCC
127567  127616  127330  127995  CDS product virion protein US10    127567-127616   9:06:02
CCGCCGGGCCCTCGCCCGTCTTCCACTCGGCCGACCCGCTGCGCCGCGCC
127645  127667  127330  127995  CDS product virion protein US10    127645-127667   5:02:01 CCGCGCCGCCCGCCGCGCACGCC
127775  127794  127330  127995  CDS product virion protein US10    127775-127794   4:01:01 GGCCCGGCTCGGCATCGGGG
127838  127854  127330  127995  CDS product virion protein US10    127838-127854   4:01:01 GGAGCTGGCCGGGGCGG
127869  127905  127330  127995  CDS product virion protein US10    127869-127905   8:05:02
CCGCGCCACGCGGCCGCCGCCGCCTACCTGCGCGCCC
127906  127917  127330  127995  CDS product virion protein US10    127906-127917   4:01:01 GGGGGCGGCGGG
127920  127936  127330  127995  CDS product virion protein US10    127920-127936   5:02:01 CCGCTCCCGCCGCCCCC
127948  127968  127330  127995  CDS product virion protein US10    127948-127968   4:01:01 GGCCCCGCGGCGGGGACCCGG
127964  127988  127330  127995  CDS product virion protein US10    127964-127988   5:02:01 CCCGGCCGCGCTCCGGGAGACCCCC
128370  128391  128351  128707  CDS product membrane protein US9    128370-128391   4:01:01 CCAGGAACGCCCCGGCCACGCC
128409  128434  128351  128707  CDS product membrane protein US9    128409-128434   5:02:01
CCACGACCAGCGCCACGCCGGCGACC
128439  128455  128351  128707  CDS product membrane protein US9    128439-128455   4:01:01 GGGCGCACTGGCGGCGG
128555  128568  128351  128707  CDS product membrane protein US9    128555-128568   4:01:01 CCGCCCGCCCCGCC
128591  128629  128351  128707  CDS product membrane protein US9    128591-128629   8:05:02
CCCCCCGGGGGCGCCGCCACGGTCGCCTCCAGGCCCACC
128836  128879  128805  129599  CDS product virion protein V67    128836-128879   9:06:02
GGTTGGCGGGCGTGGGCGGCGGGGTTTGGCCAAACGGGCACGGG
128974  128995  128805  129599  CDS product virion protein V67    128974-128995   5:02:01 GGGGGAGCGGCAGGGGCCTGGG
129228  129240  128805  129599  CDS product virion protein V67    129228-129240   4:01:01 GGGGGCAGGAAGG

129258 129285 128805 129599 CDS product virion protein V67 129258-129285 6:03:01 CCCTCCACCTTCACCTCGCCGTACAGCC
129452 129485 128805 129599 CDS product virion protein V67 129452-129485 7:04:01 CCTTCGCGCCCGCCCGGCGCCGGCCTCCGCGGCC
129467 129487 128805 129599 CDS product virion protein V67 129467-129487 4:01:01 GGCGCCGGCCTCCGCGGCCGG
129500 129537 128805 129599 CDS product virion protein V67 129500-129537 6:03:01 GGCGGGCTCTCTGGAGTCAGCGGCGTCTTGGCGCTGGG
129563 129573 128805 129599 CDS product virion protein V67 129563-129573 4:01:01 GGCGGCGGAGG
129764 129792 129705 130700 CDS product virion protein US2 129764-129792 6:03:01 CCGCGCCCCGACGCCCCCTTCACTCCCCC
129838 129863 129705 130700 CDS product virion protein US2 129838-129863 4:01:01 CCGGGGGGGCCCGGGGCGCCGCAGCC
129890 129918 129705 130700 CDS product virion protein US2 129890-129918 6:03:01 GGAGGCCGGGGTGGGCGCGGAGCCCGGGG
129956 129997 129705 130700 CDS product virion protein US2 129956-129997 9:06:02 GGGGGTGGCTGGCGTGGCGCGCCCGGGGACGTGGCGGAGGGG
130087 130113 129705 130700 CDS product virion protein US2 130087-130113 5:02:01 CCACCGCGTAGACCATGCGCGCCACCC
130129 130140 129705 130700 CDS product virion protein US2 130129-130140 4:01:01 GGTCGGCGGGGG
130179 130196 129705 130700 CDS product virion protein US2 130179-130196 4:01:01 GGCACGGCCGGAAACCGG
130186 130207 129705 130700 CDS product virion protein US2 130186-130207 4:01:01 CCGGAAACCGGCGACCGCATCC
130230 130244 129705 130700 CDS product virion protein US2 130230-130244 4:01:01 CCGGGCCACTCCTCC
130271 130286 129705 130700 CDS product virion protein US2 130271-130286 4:01:01 GGGCCCCGGGAGGAGG
130330 130343 129705 130700 CDS product virion protein US2 130330-130343 4:01:01 GGTGGTAGGGGCGG
130485 130506 129705 130700 CDS product virion protein US2 130485-130506 4:01:01 GGCCTGCAGGGCTTCGGTAGGG
130595 130631 129705 130700 CDS product virion protein US2 130595-130631 6:03:01

CCGCGCCAAAAACTCCCACAGGTGCCCGTCCACGTCC

130940 130957 130842 132023 CDS product serine/threonine protein kinase US3 130940-130957 4:01:01
CCCGATGCCCCCCCAGCC

130959 130982 130842 132023 CDS product serine/threonine protein kinase US3 130959-130982 6:03:01
GGGGTGGGCCCGGTCGGGGGGCGG

131092 131116 130842 132023 CDS product serine/threonine protein kinase US3 131092-131116 5:02:01
CCTCGTCCCCTCCAGGTCCAAGGCC

131200 131219 130842 132023 CDS product serine/threonine protein kinase US3 131200-131219 5:02:01
GGAGGGGGAGGACGAGACGG

131348 131366 130842 132023 CDS product serine/threonine protein kinase US3 131348-131366 4:01:01
CCCCACTACCGGTCCGACC

131397 131417 130842 132023 CDS product serine/threonine protein kinase US3 131397-131417 4:01:01
CCATCCCGCTCGACCGGGCCC

131547 131575 130842 132023 CDS product serine/threonine protein kinase US3 131547-131575 5:02:01
CCGCCAGGTACCCCGTCGACGCCCCGGCC

131572 131600 130842 132023 CDS product serine/threonine protein kinase US3 131572-131600 5:02:01
GGCCGAGCTGGGAATAGCCGGGACGGTGG

131850 131870 130842 132023 CDS product serine/threonine protein kinase US3 131850-131870 4:01:01
CCACCCGAGAGCGCCGGCCCC

131968 131984 130842 132023 CDS product serine/threonine protein kinase US3 131968-131984 4:01:01
GGCGGCGGAGCTGCTGG

132258 132288 132158 133504 CDS product envelope glycoprotein G 132258-132288 5:02:01
CCCTGAGCCATGGCCCCTGCTCCCGGGCCCC

| | | | | | | | |
|---|---|---|---|---|---|---|---|
| 132716 | 132736 | 132158 | 133504 | CDS product envelope glycoprotein G | 132716-132736 | 4:01:01 | CCCGCGACCACCTGCGAGACC |
| 132960 | 132982 | 132158 | 133504 | CDS product envelope glycoprotein G | 132960-132982 | 4:01:01 | CCTTGCCAGGGGCCCTCAATCCC |
| 133148 | 133164 | 132158 | 133504 | CDS product envelope glycoprotein G | 133148-133164 | 4:01:01 | CCCCCGCTTCTCCCACC |
| 133207 | 133229 | 132158 | 133504 | CDS product envelope glycoprotein G | 133207-133229 | 5:02:01 | CCAACCCCAGCGCCACCGGCCCC |
| 133758 | 133781 | 133704 | 135005 | CDS product envelope glycoprotein J | 133758-133781 | 5:02:01 | GGTACGGCCTTCCCGGCGCGGAGG |
| 133822 | 133836 | 133704 | 135005 | CDS product envelope glycoprotein J | 133822-133836 | 4:01:01 | GGACGGAGGGGAGGG |
| 133913 | 133927 | 133704 | 135005 | CDS product envelope glycoprotein J | 133913-133927 | 4:01:01 | CCGCCCACCCTGTCC |
| 133973 | 134008 | 133704 | 135005 | CDS product envelope glycoprotein J | 133973-134008 | 7:04:01 | CCCGCCTTCACCGCCCTCGCAACCCGGGAACCGGCC |
| 134055 | 134083 | 133704 | 135005 | CDS product envelope glycoprotein J | 134055-134083 | 5:02:01 | CCGCCGAGGAAACCCCAACGCATCCGCCC |
| 134124 | 134155 | 133704 | 135005 | CDS product envelope glycoprotein J | 134124-134155 | 7:04:01 | CCACCACGCCCTTCGCCGCAGCCGGCTCCACC |
| 134165 | 134212 | 133704 | 135005 | CDS product envelope glycoprotein J | 134165-134212 | 8:05:02 | CCCGCCCTCTCTACCACCAAGCGGCCCGGTGCCGCCCCGGCAGCGCCC |
| 134237 | 134262 | 133704 | 135005 | CDS product envelope glycoprotein J | 134237-134262 | 5:02:01 | CCCGCTCCGATCACGCCCCCCGGCCC |
| 134393 | 134413 | 133704 | 135005 | CDS product envelope glycoprotein J | 134393-134413 | 4:01:01 | GGCATGGGAACCCGGCGCAGG |
| 134427 | 134459 | 133704 | 135005 | CDS product envelope glycoprotein J | 134427-134459 | 7:04:01 | GGGGCGTTATGGTGGACGTGGTGGTTGGAACGG |
| 134622 | 134638 | 133704 | 135005 | CDS product envelope glycoprotein J | 134622-134638 | 4:01:01 | CCTCCGGGGACCGCGCC |
| 134667 | 134699 | 133704 | 135005 | CDS product envelope glycoprotein J | 134667-134699 | 7:04:01 | GGCCCGTGGTCAGGTGGACAGTGGCGGCGCGGG |

134741 134757 133704 135005 CDS product envelope glycoprotein J 134741-134757 4:01:01 GGCCCGGGGGAGTGCGG

134782 134825 133704 135005 CDS product envelope glycoprotein J 134782-134825 7:04:01 CCCCGCTATGCCCCGCGCCGACCCGACGGCCTCTGCGTCCTACC

134839 134860 133704 135005 CDS product envelope glycoprotein J 134839-134860 5:02:01 CCCCCCGGGCGGCCAGACCACC

135129 135187 135101 136321 CDS product envelope glycoprotein D 135129-135187 10:07:02 CCTCGGCCTCCTGCTGGCCCTGCTGGCCCGGCCCGTCGCCGGGGCCACTGGCCCCGGCC

135244 135268 135101 136321 CDS product envelope glycoprotein D 135244-135268 5:02:01 CCGCTGCCAAAGTTTCCACCTCCCC

135594 135616 135101 136321 CDS product envelope glycoprotein D 135594-135616 4:01:01 GGACTACGTGGTGGACACGTCGG

135643 135660 135101 136321 CDS product envelope glycoprotein D 135643-135660 5:02:01 CCGCCGCCGGGCGCCGCC

135849 135867 135101 136321 CDS product envelope glycoprotein D 135849-135867 4:01:01 CCAGCCGCACCACATCGCC

135938 135954 135101 136321 CDS product envelope glycoprotein D 135938-135954 4:01:01 CCAAACCCGCGCCCCCC

136008 136034 135101 136321 CDS product envelope glycoprotein D 136008-136034 6:03:01 CCGTCCACAATCCGGACCTCCGCATCC

136044 136062 135101 136321 CDS product envelope glycoprotein D 136044-136062 4:01:01 CCCGCCACCAGACGCACCC

136125 136151 135101 136321 CDS product envelope glycoprotein D 136125-136151 5:02:01 GGGCGGAAAGTCCGGGGGAAGACCGGG

136188 136206 135101 136321 CDS product envelope glycoprotein D 136188-136206 4:01:01 CCTAGCCCTGGCCGGTGCC

136260 136276 135101 136321 CDS product envelope glycoprotein D 136260-136276 4:01:01 GGTGGGAGGCATTGGGG

136469 136489 136427 137770 CDS product envelope glycoprotein I 136469-136489 4:01:01 CCCTCGCGGCCGGCCGCGCCC

136760 136781 136427 137770 CDS product envelope glycoprotein I 136760-136781 4:01:01 CCAGGCACCACCACGGCGACCC

136945 136964 136427 137770 CDS product envelope glycoprotein I 136945-136964 4:01:01 GGGGAGGGAGAAGGCAACGG

136975 137028 136427 137770 CDS product envelope glycoprotein I 136975-137028 10:07:02

CCAACGCCCGCCCCCCCGAGCGACCCCCCGTCTGCTCCCGCCACACCGACGCCC
137038 137061 136427 137770 CDS product envelope glycoprotein I 137038-137061 5:02:01 CCAACCGCGGCCGCAACCGAAGCC
137072 137105 136427 137770 CDS product envelope glycoprotein I 137072-137105 7:04:01
CCGCGGCGACCTTTATCGCCACCCCCGCCCCACC
137151 137165 136427 137770 CDS product envelope glycoprotein I 137151-137165 4:01:01 GGTGCAGGTCGGCGG
137268 137289 136427 137770 CDS product envelope glycoprotein I 137268-137289 5:02:01 GGAGGAAGACGGCGCCGGTGGG
137315 137366 136427 137770 CDS product envelope glycoprotein I 137315-137366 11:08:02
CCACCGGCGGAGCCGCCACGACCACAACAACCGCCCCACCAACCACACCCCC
137514 137538 136427 137770 CDS product envelope glycoprotein I 137514-137538 5:02:01
CCGCCGCATTTACCGCCCCGGCTCC
137533 137569 136427 137770 CDS product envelope glycoprotein I 137533-137569 7:04:01
GGCTCCGGGCTCGGGCCTGGTACGGCCGCGGAGCTGG
137570 137592 136427 137770 CDS product envelope glycoprotein I 137570-137592 4:01:01 CCACGCTCGCCCGCTCGCCCGCC
137603 137634 136427 137770 CDS product envelope glycoprotein I 137603-137634 6:03:01
CCTCGGAGTCCCTCGCATCCGAGGCCGCCGCC
137645 137661 136427 137770 CDS product envelope glycoprotein I 137645-137661 4:01:01 CCGCGTCCGCGCCCCCC
137690 137717 136427 137770 CDS product envelope glycoprotein I 137690-137717 5:02:01
CCTCGAAGCCCGGGCAGCCCCCCGATCC
138089 138110 138046 139713 CDS product envelope glycoprotein E 138089-138110 4:01:01 CCTGGCGCCAGCCTTTGGGACC
138162 138215 138046 139713 CDS product envelope glycoprotein E 138162-138215 8:05:02
GGGGGCACGCTCGGCGTTCCGGACAACGGGACCTCGCGGGTGCTCAAGGCCTGG
138211 138232 138046 139713 CDS product envelope glycoprotein E 138211-138232 4:01:01 CCTGGACCTTCCTGGAAACCCC
138341 138374 138046 139713 CDS product envelope glycoprotein E 138341-138374 7:04:01

CCCCCTGGCCCTGGCCGAGTTCACCGCCGCAGCC
138426 138453 138046 139713 CDS product envelope glycoprotein E 138426-138453 4:01:01
CCCATTTTGACCGAAACCCGAAGCGGCC
138472 138485 138046 139713 CDS product envelope glycoprotein E 138472-138485 4:01:01 CCACCAACCTCACC
138567 138616 138046 139713 CDS product envelope glycoprotein E 138567-138616 10:07:02
CCCAAGCCGTCCGCGGCACCCGCGCCCACGCCGACCACGACCGCCGCGCC
138940 138962 138046 139713 CDS product envelope glycoprotein E 138940-138962 5:02:01 CCATCCAGTGCCACAGCACCCCC
139067 139091 138046 139713 CDS product envelope glycoprotein E 139067-139091 5:02:01
GGTCAACGGACACCCGGAGGCGTGG
139138 139160 138046 139713 CDS product envelope glycoprotein E 139138-139160 5:02:01 CCGACACCACCCGCCCACGGCCC
139209 139232 138046 139713 CDS product envelope glycoprotein E 139209-139232 5:02:01 CCCGCCGAGGTTCCGCCAGCTCCC
139233 139250 138046 139713 CDS product envelope glycoprotein E 139233-139250 4:01:01 GGACGCGGCTGGGCTCGG
139505 139553 138046 139713 CDS product envelope glycoprotein E 139505-139553 10:07:02
CCCGGGACTGCCGCCGGCGCCCGACTCTCCTCCGCTCCCCCGCCCCCCC
139585 139604 138046 139713 CDS product envelope glycoprotein E 139585-139604 4:01:01 GGTCGGGATACAAGGTCTGG
139616 139633 138046 139713 CDS product envelope glycoprotein E 139616-139633 4:01:01 CCCCGAGCCCTCGCCCCC
139647 139661 138046 139713 CDS product envelope glycoprotein E 139647-139661 4:01:01 CCGCCCCGCCAAGCC
139833 139864 139710 140135 CDS product putative membrane protein US8A 139833-139864 6:03:01
CCATCAGCCGCTACCGCCGCGCGTCCGAGTCC
139937 139950 139710 140135 CDS product putative membrane protein US8A 139937-139950 4:01:01 CCCGACCGCCAACC
139967 139986 139710 140135 CDS product putative membrane protein US8A 139967-139986 4:01:01
CCCAACTCTCCGGCCGCGCC
139997 140020 139710 140135 CDS product putative membrane protein US8A 139997-140020 4:01:01

CCCAAAGCCCCGCCCGAGAAACCC

140451 140489 140351 140731 CDS product membrane protein US9 140451-140489 8:05:02

GGTGGGCCTGGAGGCGACCGTGGCGGCGCCCCCGGGGGG

140512 140525 140351 140731 CDS product membrane protein US9 140512-140525 4:01:01 GGCGGGGCGGGCGG

140625 140641 140351 140731 CDS product membrane protein US9 140625-140641 4:01:01 CCGCCGCCAGTGCGCCC

140646 140671 140351 140731 CDS product membrane protein US9 140646-140671 5:02:01

GGTCGCCGGCGTGGCGCTGGTCGTGG

140689 140710 140351 140731 CDS product membrane protein US9 140689-140710 4:01:01 GGCGTGGCCGGGGCGTTCCTGG

141092 141116 141087 141752 CDS product virion protein US10 141092-141116 5:02:01 GGGGGTCTCCCGGAGCGCGGCCGGG

141112 141132 141087 141752 CDS product virion protein US10 141112-141132 4:01:01 CCGGGTCCCCGCCGCGGGGCC

141144 141160 141087 141752 CDS product virion protein US10 141144-141160 5:02:01 GGGGGCGGCGGGAGCGG

141163 141174 141087 141752 CDS product virion protein US10 141163-141174 4:01:01 CCCGCCGCCCCC

141175 141211 141087 141752 CDS product virion protein US10 141175-141211 8:05:02

GGGCGCGCAGGTAGGCGGCGGCGGCCGCGTGGCGCGG

141226 141242 141087 141752 CDS product virion protein US10 141226-141242 4:01:01 CCGCCCCGGCCAGCTCC

141286 141305 141087 141752 CDS product virion protein US10 141286-141305 4:01:01 CCCCGATGCCGAGCCGGGCC

141413 141435 141087 141752 CDS product virion protein US10 141413-141435 5:02:01 GGCGTGCGCGGCGGGCGGCGCGG

141464 141513 141087 141752 CDS product virion protein US10 141464-141513 9:06:02

GGCGCGGCGCAGCGGGTCGGCCGAGTGGAAGACGGGCGAGGGCCCGGCGG

141555 141577 141087 141752 CDS product virion protein US10 141555-141577 5:02:01 GGCCACAGGCCCTGGGGCGGGGG

141586 141618 141087 141752 CDS product virion protein US10 141586-141618 8:05:02

GGCGCAGGGCCTCGGCGGCCTCGGCGGAGGCGG

141721 141741 141087 141752 CDS product virion protein US10 141721-141741 4:01:01 GGGCCATGGGGGAGACCGAGG

141955 141977 141938 143005 CDS product regulatory protein ICP22 141955-141977 5:02:01
GGCGGGACGGCCGCGGCCGCGGG
141965 141983 141938 143005 CDS product regulatory protein ICP22 141965-141983 4:01:01 CCGCGGCCGCGGGCCTTCC
142006 142050 141938 143005 CDS product regulatory protein ICP22 142006-142050 10:07:02
GGGCGGCGTCTCGGGGGAGGAGGAGGGCAACGGGGAGAAGGGGGG
142059 142073 141938 143005 CDS product regulatory protein ICP22 142059-142073 4:01:01 GGGCGGCGCGGCGGG
142083 142116 141938 143005 CDS product regulatory protein ICP22 142083-142116 8:05:02
GGAGCACGGGCGGCGTCTCGGGGGAGGAGGGGGG
142149 142183 141938 143005 CDS product regulatory protein ICP22 142149-142183 8:05:02
GGAGCACGGGCGGCGTCTCGGGGGAGGAGGGCGGG
142204 142220 141938 143005 CDS product regulatory protein ICP22 142204-142220 4:01:01 GGTCCAGCGGCCGGTGG
142238 142255 141938 143005 CDS product regulatory protein ICP22 142238-142255 5:02:01 CCGCCAAAGCCCCCGTCC
142296 142308 141938 143005 CDS product regulatory protein ICP22 142296-142308 4:01:01 CCTCCTCCTCGCC
142339 142377 141938 143005 CDS product regulatory protein ICP22 142339-142377 10:07:02
GGAGGGGGCGGAGGACGACGAGGCGGAGGACGGGGAGGG
142431 142450 141938 143005 CDS product regulatory protein ICP22 142431-142450 4:01:01 GGGGCTCGGGGGGGTCGAGG
142659 142690 141938 143005 CDS product regulatory protein ICP22 142659-142690 7:04:01
GGAAGAGGCGGTTCAGGCGCGCGGCGAGGCGG
142729 142773 141938 143005 CDS product regulatory protein ICP22 142729-142773 7:04:01
GGGCCCGCCGGCGAGGCGCGGGGCGAGGGCGCGGGGCGAGACGGG
142776 142803 141938 143005 CDS product regulatory protein ICP22 142776-142803 5:02:01
CCCCGTACTCCGGGTGGTCCGCCCCCCC
142833 142938 141938 143005 CDS product regulatory protein ICP22 142833-142938 23:20:05

GGGGGGCGGAGCCGGGCGTGGGGGAGAGGGAGGCGCGCCAGGAGGGGGCGGTGGCGCCGGAGCGGGGGGGCGACGGCGACAGAGGCGGGATGAGGGGGCTGGCGGG

142947 142964 141938 143005 CDS product regulatory protein ICP22 142947-142964 5:02:01 GGGGGCGCAGGCGGCAGG

146719 146743 146649 150797 CDS product transcriptional regulator ICP4 146719-146743 6:03:01
CCTCATCCGGGCCGCCGGTGCCGCC

146754 146774 146649 150797 CDS product transcriptional regulator ICP4 146754-146774 4:01:01
CCCAGCAGCACCCCGACCTCC

146780 146804 146649 150797 CDS product transcriptional regulator ICP4 146780-146804 4:01:01
GGGAGCCAGGGGCTCTTCGGGGTGG

146826 146879 146649 150797 CDS product transcriptional regulator ICP4 146826-146879 13:10:03
CCCCCGCCATCCCCGCCATCCCCGCCTCCCCGGCTCCGCCGCCGCCGGAGCCCC

146888 146950 146649 150797 CDS product transcriptional regulator ICP4 146888-146950 13:10:03
CCCAAGCAGGCCGCGCCGCCCACCAAGCCGCCCGCCCCGCCGACGGTGCCCTCGCCGGCCCCC

146944 146967 146649 150797 CDS product transcriptional regulator ICP4 146944-146967 5:02:01
GGCCCCCGGCGGGGAGCCGGCGGG

146989 147030 146649 150797 CDS product transcriptional regulator ICP4 146989-147030 7:04:01
GGTCCGGGCCGAGATGGAGACCACGGAGGTCGAAGGGGACGG

147066 147187 146649 150797 CDS product transcriptional regulator ICP4 147066-147187 24:21:06
CCACCCCTCCGCTCCCCGCGTCCGCCTCCCCGCCGTCGAGGCCCCCGCGCGCCCGGAGCGCCTCCTGCTCCTCGTCCTCGTCCGCGTCCTCCTCCTCTGGCTCCTCCGCCTCGGACTCCGCC

147201 147223 146649 150797 CDS product transcriptional regulator ICP4 147201-147223 5:02:01
CCGACGCCGCGCCCGCCCCGTCC

147234 147253 146649 150797 CDS product transcriptional regulator ICP4 147234-147253 6:03:01
CCCGCCGTCCCCCGCCGCCC

147263 147284 146649 150797 CDS product transcriptional regulator ICP4 147263-147284 4:01:01

CCGGCTCTCCCGCCCGGAGACC

147357 147427 146649 150797 CDS product transcriptional regulator ICP4 147357-147427 14:11:03

CCCCTTCGCCCTCCCCGCGGGCCCCGCCGCCCGCCGCCCTCGGGGGCCCGCCGCCGCCCTCGGGGGCCTCC

147442 147512 146649 150797 CDS product transcriptional regulator ICP4 147442-147512 13:10:03

CCGGCCGGTGCCCGCGTTCCTCCAGGCCGCCCCGCGAACCCCGTCCCCGCCCGCCGATCGGTCCTCGCCCC

147527 147564 146649 150797 CDS product transcriptional regulator ICP4 147527-147564 8:05:02

CCGCCGCAGCCCTCCTCGCCGCCCCTGCCCCAGCAGCC

147588 147659 146649 150797 CDS product transcriptional regulator ICP4 147588-147659 12:09:03

CCGGCGCCGGCCGCCCCTCGGGCCCGGGCCCCGACGCCGCCCGGGAGCCCGCGGCCGGCCCCGCGGAGCCCC

147630 147653 146649 150797 CDS product transcriptional regulator ICP4 147630-147653 4:01:01

GGGAGCCCGCGGCCGGCCCCGCGG

147668 147713 146649 150797 CDS product transcriptional regulator ICP4 147668-147713 9:06:02

CCGGCCGGGGACCCCTGGCCGGGCTCCGACCCGCCGCCCAAGGGCC

147670 147690 146649 150797 CDS product transcriptional regulator ICP4 147670-147690 4:01:01

GGCCGGGGACCCCTGGCCGGG

147725 147758 146649 150797 CDS product transcriptional regulator ICP4 147725-147758 6:03:01

GGGGGCACCGGGGACTCGCGGGAGGGGCTCTGGG

147798 147817 146649 150797 CDS product transcriptional regulator ICP4 147798-147817 4:01:01

CCGCCGGCGGGCCCGTGCCC

147829 147846 146649 150797 CDS product transcriptional regulator ICP4 147829-147846 4:01:01

GGAGATGGGGGACTCGGG

148078 148091 146649 150797 CDS product transcriptional regulator ICP4 148078-148091 4:01:01 GGCGGCCTCGGTGG

148083 148112 146649 150797 CDS product transcriptional regulator ICP4 148083-148112 5:02:01

CCTCGGTGGCCATGAGCCGCCGATACGACC
148132 148153 146649 150797 CDS product transcriptional regulator ICP4 148132-148153 4:01:01
CCTGCAGAGCCTGCGCCGGGCC
148167 148180 146649 150797 CDS product transcriptional regulator ICP4 148167-148180 4:01:01 CCTTCCCCCGGCCC
148194 148250 146649 150797 CDS product transcriptional regulator ICP4 148194-148250 13:10:03
CCCCCGCCGCCGAGCCCGGCTCGCCGCCGGGCCCCGGACCCGCCGCCCCCTCGCCCC
148260 148316 146649 150797 CDS product transcriptional regulator ICP4 148260-148316 11:08:02
CCTCCTCCTCCTCCCCGACGGCCGGGGCGCCGCGCTCCCCGCCGCAGGCCCGGGCCC
148363 148390 146649 150797 CDS product transcriptional regulator ICP4 148363-148390 4:01:01
CCGGGCGCCCGGGGAGCCCGGGGAGCCC
148365 148402 146649 150797 CDS product transcriptional regulator ICP4 148365-148402 6:03:01
GGGCGCCCGGGGAGCCCGGGGAGCCCGGGGCGGCGCGG
148412 148439 146649 150797 CDS product transcriptional regulator ICP4 148412-148439 6:03:01
GGGGGCGCCCGGGTCAAGGAGCTGGGGG
148443 148465 146649 150797 CDS product transcriptional regulator ICP4 148443-148465 5:02:01
CCTGCGTCCTCGCCTGCCACGCC
148485 148505 146649 150797 CDS product transcriptional regulator ICP4 148485-148505 4:01:01
GGCTCCGCGGAGGCGCCGCGG
148500 148525 146649 150797 CDS product transcriptional regulator ICP4 148500-148525 6:03:01
CCGCGGCCGTCCCCGGCCTCCAGCCC
148534 148562 146649 150797 CDS product transcriptional regulator ICP4 148534-148562 6:03:01
CCCGACGCCCGCCTGCCCCCCGGAGGCCC
148568 148589 146649 150797 CDS product transcriptional regulator ICP4 148568-148589 4:01:01

GGCAACCCCGGCGGCCTCCGGG

148573 148603 146649 150797 CDS product transcriptional regulator ICP4 148573-148603 7:04:01

CCCCGGCGGCCTCCGGGCCGCCGCCGCGGCC

148638 148662 146649 150797 CDS product transcriptional regulator ICP4 148638-148662 4:01:01

GGCTCCTGGGCCCGCAGGACCCCGG

148725 148752 146649 150797 CDS product transcriptional regulator ICP4 148725-148752 4:01:01

CCGTCGCCCCGCTCGTCCGGTACAACCC

148767 148787 146649 150797 CDS product transcriptional regulator ICP4 148767-148787 4:01:01

CCCGCGCCTCGGCCTGGACCC

148819 148841 146649 150797 CDS product transcriptional regulator ICP4 148819-148841 5:02:01

GGCGGCCCGGCAGATGGCCGAGG

148836 148872 146649 150797 CDS product transcriptional regulator ICP4 148836-148872 6:03:01

CCGAGGCCGTCGCCGCGCTCCCCGGCGCGCCCCGGCC

148903 148986 146649 150797 CDS product transcriptional regulator ICP4 148903-148986 17:14:04

CCACCTGCCGCCCCTGTGGGCCCACCCGCCGCAGCCCCAGCCGCCGGGCCCTCCGAGCCCGGCCGCGCACCGGGCCCCGTCCCC

148996 149025 146649 150797 CDS product transcriptional regulator ICP4 148996-149025 8:05:02

CCCGGCCCCCGCCGCCCCCTGCGCCTCCCC

149034 149092 146649 150797 CDS product transcriptional regulator ICP4 149034-149092 10:07:02

CCCGGGGCCTCCCCGAGGCCTCGGAGGCCGCGTCCCCCCGCTGCCTGGGACGCCTTCCC

149097 149116 146649 150797 CDS product transcriptional regulator ICP4 149097-149116 4:01:01

GGCGCCCCAGGCAGCGGGGG

149122 149143 146649 150797 CDS product transcriptional regulator ICP4 149122-149143 5:02:01

CCGCTCCGAGCCGCCGCGCCCC

149175  149267  146649  150797  CDS product transcriptional regulator ICP4  149175-149267  18:15:04
CCGGGGCCGCCACCCCGCCGGCACCCAAGCGCCGGCCCGGCGCCCCCCCGCCTCGCGCCCTGGGCCCCATGCCCGCCGGCGGCCCCGACCCCC
149302  149362  146649  150797  CDS product transcriptional regulator ICP4  149302-149362  10:07:02
CCACACGCCGGCGCCCGGCGAGGCCGCCCGCGCGGCCTACTGCTCCCCCGAGACCGTGGCC
149389  149401  146649  150797  CDS product transcriptional regulator ICP4  149389-149401  4:01:01 GGAGGCATGGCGG
149402  149447  146649  150797  CDS product transcriptional regulator ICP4  149402-149447  8:05:02
CCCGCGCTCACCTTCGACCCGGCCGCCCTGGCGACCGTCGCCGCCC
149448  149475  146649  150797  CDS product transcriptional regulator ICP4  149448-149475  4:01:01
GGCGCACGGGGCCCCCGGCCCGCGAGGG
149496  149527  146649  150797  CDS product transcriptional regulator ICP4  149496-149527  7:04:01
CCCCCAGCGGCCCGCTGCGCCGCCGCGCCGCC
149572  149590  146649  150797  CDS product transcriptional regulator ICP4  149572-149590  5:02:01
CCTCTACCACCCCCTGCCC
149591  149610  146649  150797  CDS product transcriptional regulator ICP4  149591-149610  4:01:01
GGCGAGGACCTGCTGGGCGG
149621  149646  146649  150797  CDS product transcriptional regulator ICP4  149621-149646  6:03:01
GGCGGCGCGGCGGGCCGCGAGGGCGG
149733  149751  146649  150797  CDS product transcriptional regulator ICP4  149733-149751  4:01:01
GGGCCGGCAACTGGACCGG
149799  149815  146649  150797  CDS product transcriptional regulator ICP4  149799-149815  4:01:01
CCACCGGGGACCTGGCC
149804  149831  146649  150797  CDS product transcriptional regulator ICP4  149804-149831  4:01:01
GGGGACCTGGCCTTCGCGGGCTGCGTGG

149851 149869 146649 150797 CDS product transcriptional regulator ICP4 149851-149869 4:01:01
GGGCTCGGCCCGGCGCCGG
149906 149923 146649 150797 CDS product transcriptional regulator ICP4 149906-149923 4:01:01
CCCGCCGACGGCCCGGCC
150121 150143 146649 150797 CDS product transcriptional regulator ICP4 150121-150143 4:01:01
GGGCAACGTGGCCTACGTGGTGG
150173 150197 146649 150797 CDS product transcriptional regulator ICP4 150173-150197 5:02:01
CCCATGCCGCCCCGGGACTACCGCC
150240 150259 146649 150797 CDS product transcriptional regulator ICP4 150240-150259 4:01:01
CCCAGAGCCGGGGCCTGGCC
150298 150314 146649 150797 CDS product transcriptional regulator ICP4 150298-150314 4:01:01
CCACCGCGCGGCCAACC
150332 150365 146649 150797 CDS product transcriptional regulator ICP4 150332-150365 7:04:01
CCGCTGCGTCCGCTCTACCTGGCCGCCGGCCGCC
150352 150377 146649 150797 CDS product transcriptional regulator ICP4 150352-150377 4:01:01
GGCCGCCGGCCGCCGCGGCGCGCTGG
150384 150412 146649 150797 CDS product transcriptional regulator ICP4 150384-150412 6:03:01
CCGCCGCGCCCGCGGACCTGCTGCCGCCC
150436 150473 146649 150797 CDS product transcriptional regulator ICP4 150436-150473 6:03:01
GGCGCTGCTGGAGCCGGACCTCGAGGCCGGCCCGACGG
150449 150468 146649 150797 CDS product transcriptional regulator ICP4 150449-150468 4:01:01
CCGGACCTCGAGGCCGGCCC
150553 150575 146649 150797 CDS product transcriptional regulator ICP4 150553-150575 5:02:01

GGTGCTGGCGGCCGGGGCCGTGG

150622 150643 146649 150797 CDS product transcriptional regulator ICP4 150622-150643 5:02:01

GGAGACGGACGGCGCCGGCGGG

150680 150724 146649 150797 CDS product transcriptional regulator ICP4 150680-150724 7:04:01

GGGCCCGCGGGCGGCAGCGGGCGCCCCGGCGCCGGGGCCCGCGGG

150703 150727 146649 150797 CDS product transcriptional regulator ICP4 150703-150727 4:01:01

CCCCGGCGCCGGGGCCCGCGGGCCC
