# Supplementary material for: Intensive Distribution of G2-Quaduplexes in the Pseudorabies Virus Genome and Their Sensitivity to Cations and G-Quadruplex Ligands
Source: Molecules. 2019 Feb 21;24(4):774. doi: 10.3390/molecules24040774 (PMC6412908; doi:10.3390/molecules24040774)
Supplement: Supplementary file 1 [file molecules-24-00774-s001.zip › Supporting information/File S4.pdf]

capsid maturation protease gi|9625875|ref|NC_001348.1| Human herpesvirus 3, complete genome
capsid maturation protease gi|50313241|ref|NC_001491.2| Equid herpesvirus 1, complete genome
capsid maturation protease gi|9629732|ref|NC_001844.1| Equid herpesvirus 4, complete genome
capsid maturation protease gi|9629818|ref|NC_001847.1| Bovine herpesvirus 1, complete genome
capsid maturation protease gi|126882977|ref|NC_002686.2| Cercopithecine herpesvirus 9, complete genome
capsid maturation protease gi|123318702|ref|NC_005261.2| Bovine herpesvirus 5, complete genome
capsid maturation protease gi|51557483|ref|NC_006151.1| Suid herpesvirus 1, complete genome
capsid maturation protease gi|216905852|ref|NC_011644.1| Equid herpesvirus 9, complete genome
capsid maturation protease gi|281190771|ref|NC_013590.2| Felid herpesvirus 1, complete genome
capsid maturation protease gi|675510705|ref|NC_024771.1| Equid herpesvirus 3 strain AR/2007/C3A, complete genome
capsid portal protein   gi|9625875|ref|NC_001348.1| Human herpesvirus 3, complete genome
capsid portal protein   gi|50313241|ref|NC_001491.2| Equid herpesvirus 1, complete genome
capsid portal protein   gi|9629732|ref|NC_001844.1| Equid herpesvirus 4, complete genome
capsid portal protein   gi|9629818|ref|NC_001847.1| Bovine herpesvirus 1, complete genome
capsid portal protein   gi|126882977|ref|NC_002686.2| Cercopithecine herpesvirus 9, complete genome
capsid portal protein   gi|123318702|ref|NC_005261.2| Bovine herpesvirus 5, complete genome
capsid portal protein   gi|51557483|ref|NC_006151.1| Suid herpesvirus 1, complete genome
capsid portal protein   gi|216905852|ref|NC_011644.1| Equid herpesvirus 9, complete genome
capsid portal protein   gi|281190771|ref|NC_013590.2| Felid herpesvirus 1, complete genome
capsid portal protein   gi|386522723|ref|NC_017826.1| Equid herpesvirus 8, complete genome
capsid portal protein   gi|675510705|ref|NC_024771.1| Equid herpesvirus 3 strain AR/2007/C3A, complete genome
capsid scaffold protein gi|9625875|ref|NC_001348.1| Human herpesvirus 3, complete genome
capsid scaffold protein gi|50313241|ref|NC_001491.2| Equid herpesvirus 1, complete genome

capsid scaffold protein gi|9629732|ref|NC_001844.1| Equid herpesvirus 4, complete genome
capsid scaffold protein gi|9629818|ref|NC_001847.1| Bovine herpesvirus 1, complete genome
capsid scaffold protein gi|123318702|ref|NC_005261.2| Bovine herpesvirus 5, complete genome
capsid scaffold protein gi|51557483|ref|NC_006151.1| Suid herpesvirus 1, complete genome
capsid scaffold protein gi|216905852|ref|NC_011644.1| Equid herpesvirus 9, complete genome
capsid scaffold protein gi|281190771|ref|NC_013590.2| Felid herpesvirus 1, complete genome
capsid scaffold protein gi|675510705|ref|NC_024771.1| Equid herpesvirus 3 strain AR/2007/C3A, complete genome
capsid triplex subunit 1 gi|9625875|ref|NC_001348.1| Human herpesvirus 3, complete genome
capsid triplex subunit 1 gi|50313241|ref|NC_001491.2| Equid herpesvirus 1, complete genome
capsid triplex subunit 1 gi|9629732|ref|NC_001844.1| Equid herpesvirus 4, complete genome
capsid triplex subunit 1 gi|9629818|ref|NC_001847.1| Bovine herpesvirus 1, complete genome
capsid triplex subunit 1 gi|126882977|ref|NC_002686.2| Cercopithecine herpesvirus 9, complete genome
capsid triplex subunit 1 gi|123318702|ref|NC_005261.2| Bovine herpesvirus 5, complete genome
capsid triplex subunit 1 gi|51557483|ref|NC_006151.1| Suid herpesvirus 1, complete genome
capsid triplex subunit 1 gi|216905852|ref|NC_011644.1| Equid herpesvirus 9, complete genome
capsid triplex subunit 1 gi|281190771|ref|NC_013590.2| Felid herpesvirus 1, complete genome
capsid triplex subunit 1 gi|675510705|ref|NC_024771.1| Equid herpesvirus 3 strain AR/2007/C3A, complete genome
capsid triplex subunit 2 gi|9625875|ref|NC_001348.1| Human herpesvirus 3, complete genome
capsid triplex subunit 2 gi|50313241|ref|NC_001491.2| Equid herpesvirus 1, complete genome
capsid triplex subunit 2 gi|9629732|ref|NC_001844.1| Equid herpesvirus 4, complete genome
capsid triplex subunit 2 gi|9629818|ref|NC_001847.1| Bovine herpesvirus 1, complete genome
capsid triplex subunit 2 gi|123318702|ref|NC_005261.2| Bovine herpesvirus 5, complete genome
capsid triplex subunit 2 gi|51557483|ref|NC_006151.1| Suid herpesvirus 1, complete genome

capsid triplex subunit 2   gi|216905852|ref|NC_011644.1| Equid herpesvirus 9, complete genome
capsid triplex subunit 2   gi|281190771|ref|NC_013590.2| Felid herpesvirus 1, complete genome
capsid triplex subunit 2   gi|675510705|ref|NC_024771.1| Equid herpesvirus 3 strain AR/2007/C3A, complete genome
deoxyribonuclease   gi|9625875|ref|NC_001348.1| Human herpesvirus 3, complete genome
deoxyribonuclease   gi|50313241|ref|NC_001491.2| Equid herpesvirus 1, complete genome
deoxyribonuclease   gi|9629732|ref|NC_001844.1| Equid herpesvirus 4, complete genome
deoxyribonuclease   gi|9629818|ref|NC_001847.1| Bovine herpesvirus 1, complete genome
deoxyribonuclease   gi|126882977|ref|NC_002686.2| Cercopithecine herpesvirus 9, complete genome
deoxyribonuclease   gi|123318702|ref|NC_005261.2| Bovine herpesvirus 5, complete genome
deoxyribonuclease   gi|51557483|ref|NC_006151.1| Suid herpesvirus 1, complete genome
deoxyribonuclease   gi|216905852|ref|NC_011644.1| Equid herpesvirus 9, complete genome
deoxyribonuclease   gi|281190771|ref|NC_013590.2| Felid herpesvirus 1, complete genome
deoxyribonuclease   gi|675510705|ref|NC_024771.1| Equid herpesvirus 3 strain AR/2007/C3A, complete genome
deoxyuridine triphosphatase gi|50313241|ref|NC_001491.2| Equid herpesvirus 1, complete genome
deoxyuridine triphosphatase gi|9629732|ref|NC_001844.1| Equid herpesvirus 4, complete genome
deoxyuridine triphosphatase gi|9629818|ref|NC_001847.1| Bovine herpesvirus 1, complete genome
deoxyuridine triphosphatase gi|123318702|ref|NC_005261.2| Bovine herpesvirus 5, complete genome
deoxyuridine triphosphatase gi|51557483|ref|NC_006151.1| Suid herpesvirus 1, complete genome
deoxyuridine triphosphatase gi|216905852|ref|NC_011644.1| Equid herpesvirus 9, complete genome
deoxyuridine triphosphatase gi|281190771|ref|NC_013590.2| Felid herpesvirus 1, complete genome
deoxyuridine triphosphatase gi|675510705|ref|NC_024771.1| Equid herpesvirus 3 strain AR/2007/C3A, complete genome
DNA packaging protein UL32 gi|9625875|ref|NC_001348.1| Human herpesvirus 3, complete genome
DNA packaging protein UL32 gi|50313241|ref|NC_001491.2| Equid herpesvirus 1, complete genome

DNA packaging protein UL32 gi|9629732|ref|NC_001844.1| Equid herpesvirus 4, complete genome
DNA packaging protein UL32 gi|51557483|ref|NC_006151.1| Suid herpesvirus 1, complete genome
DNA packaging protein UL32 gi|216905852|ref|NC_011644.1| Equid herpesvirus 9, complete genome
DNA packaging protein UL32 gi|281190771|ref|NC_013590.2| Felid herpesvirus 1, complete genome
DNA packaging protein UL32 gi|386522723|ref|NC_017826.1| Equid herpesvirus 8, complete genome
DNA packaging protein UL33 gi|9629818|ref|NC_001847.1| Bovine herpesvirus 1, complete genome
DNA packaging protein UL33 gi|123318702|ref|NC_005261.2| Bovine herpesvirus 5, complete genome
DNA packaging protein UL33 gi|675510705|ref|NC_024771.1| Equid herpesvirus 3 strain AR/2007/C3A, complete genome
DNA packaging tegument protein UL17 gi|9625875|ref|NC_001348.1| Human herpesvirus 3, complete genome
DNA packaging tegument protein UL17 gi|50313241|ref|NC_001491.2| Equid herpesvirus 1, complete genome
DNA packaging tegument protein UL17 gi|9629732|ref|NC_001844.1| Equid herpesvirus 4, complete genome
DNA packaging tegument protein UL17 gi|9629818|ref|NC_001847.1| Bovine herpesvirus 1, complete genome
DNA packaging tegument protein UL17 gi|123318702|ref|NC_005261.2| Bovine herpesvirus 5, complete genome
DNA packaging tegument protein UL17 gi|51557483|ref|NC_006151.1| Suid herpesvirus 1, complete genome
DNA packaging tegument protein UL17 gi|216905852|ref|NC_011644.1| Equid herpesvirus 9, complete genome
DNA packaging tegument protein UL17 gi|281190771|ref|NC_013590.2| Felid herpesvirus 1, complete genome
DNA packaging tegument protein UL17 gi|675510705|ref|NC_024771.1| Equid herpesvirus 3 strain AR/2007/C3A, complete genome
DNA packaging tegument protein UL25 gi|9625875|ref|NC_001348.1| Human herpesvirus 3, complete genome
DNA packaging tegument protein UL25 gi|50313241|ref|NC_001491.2| Equid herpesvirus 1, complete genome
DNA packaging tegument protein UL25 gi|9629732|ref|NC_001844.1| Equid herpesvirus 4, complete genome
DNA packaging tegument protein UL25 gi|9629818|ref|NC_001847.1| Bovine herpesvirus 1, complete genome
DNA packaging tegument protein UL25 gi|126882977|ref|NC_002686.2| Cercopithecine herpesvirus 9, complete genome
DNA packaging tegument protein UL25 gi|123318702|ref|NC_005261.2| Bovine herpesvirus 5, complete genome

DNA packaging tegument protein UL25 gi|51557483|ref|NC_006151.1| Suid herpesvirus 1, complete genome
DNA packaging tegument protein UL25 gi|216905852|ref|NC_011644.1| Equid herpesvirus 9, complete genome
DNA packaging tegument protein UL25 gi|281190771|ref|NC_013590.2| Felid herpesvirus 1, complete genome
DNA packaging tegument protein UL25 gi|675510705|ref|NC_024771.1| Equid herpesvirus 3 strain AR/2007/C3A, complete genome
DNA packaging terminase subunit 1 gi|9625875|ref|NC_001348.1| Human herpesvirus 3, complete genome
DNA packaging terminase subunit 1 gi|50313241|ref|NC_001491.2| Equid herpesvirus 1, complete genome
DNA packaging terminase subunit 1 gi|9629732|ref|NC_001844.1| Equid herpesvirus 4, complete genome
DNA packaging terminase subunit 1 gi|9629818|ref|NC_001847.1| Bovine herpesvirus 1, complete genome
DNA packaging terminase subunit 1 gi|123318702|ref|NC_005261.2| Bovine herpesvirus 5, complete genome
DNA packaging terminase subunit 1 gi|51557483|ref|NC_006151.1| Suid herpesvirus 1, complete genome
DNA packaging terminase subunit 1 gi|216905852|ref|NC_011644.1| Equid herpesvirus 9, complete genome
DNA packaging terminase subunit 1 gi|281190771|ref|NC_013590.2| Felid herpesvirus 1, complete genome
DNA packaging terminase subunit 1 gi|675510705|ref|NC_024771.1| Equid herpesvirus 3 strain AR/2007/C3A, complete genome
DNA packaging terminase subunit 2 gi|9625875|ref|NC_001348.1| Human herpesvirus 3, complete genome
DNA packaging terminase subunit 2 gi|50313241|ref|NC_001491.2| Equid herpesvirus 1, complete genome
DNA packaging terminase subunit 2 gi|9629732|ref|NC_001844.1| Equid herpesvirus 4, complete genome
DNA packaging terminase subunit 2 gi|9629818|ref|NC_001847.1| Bovine herpesvirus 1, complete genome
DNA packaging terminase subunit 2 gi|126882977|ref|NC_002686.2| Cercopithecine herpesvirus 9, complete genome
DNA packaging terminase subunit 2 gi|123318702|ref|NC_005261.2| Bovine herpesvirus 5, complete genome
DNA packaging terminase subunit 2 gi|51557483|ref|NC_006151.1| Suid herpesvirus 1, complete genome
DNA packaging terminase subunit 2 gi|216905852|ref|NC_011644.1| Equid herpesvirus 9, complete genome
DNA packaging terminase subunit 2 gi|281190771|ref|NC_013590.2| Felid herpesvirus 1, complete genome
DNA packaging terminase subunit 2 gi|386522723|ref|NC_017826.1| Equid herpesvirus 8, complete genome

DNA packaging terminase subunit 2 gi|675510705|ref|NC_024771.1| Equid herpesvirus 3 strain AR/2007/C3A, complete genome
DNA packaging tsingle-stranded DNA-binding protein gi|9625875|ref|NC_001348.1| Human herpesvirus 3, complete genome
DNA packaging tsingle-stranded DNA-binding protein gi|50313241|ref|NC_001491.2| Equid herpesvirus 1, complete genome
DNA packaging tsingle-stranded DNA-binding protein gi|9629732|ref|NC_001844.1| Equid herpesvirus 4, complete genome
DNA packaging tsingle-stranded DNA-binding protein gi|9629818|ref|NC_001847.1| Bovine herpesvirus 1, complete genome
DNA packaging tsingle-stranded DNA-binding protein gi|126882977|ref|NC_002686.2| Cercopithecine herpesvirus 9, complete genome
DNA packaging tsingle-stranded DNA-binding protein gi|123318702|ref|NC_005261.2| Bovine herpesvirus 5, complete genome
DNA packaging tsingle-stranded DNA-binding protein gi|51557483|ref|NC_006151.1| Suid herpesvirus 1, complete genome
DNA packaging tsingle-stranded DNA-binding protein gi|216905852|ref|NC_011644.1| Equid herpesvirus 9, complete genome
DNA packaging tsingle-stranded DNA-binding protein gi|281190771|ref|NC_013590.2| Felid herpesvirus 1, complete genome
DNA packaging tsingle-stranded DNA-binding protein gi|675510705|ref|NC_024771.1| Equid herpesvirus 3 strain AR/2007/C3A, complete genome
DNA polymerase catalytic subunit gi|9625875|ref|NC_001348.1| Human herpesvirus 3, complete genome
DNA polymerase catalytic subunit gi|50313241|ref|NC_001491.2| Equid herpesvirus 1, complete genome
DNA polymerase catalytic subunit gi|9629732|ref|NC_001844.1| Equid herpesvirus 4, complete genome
DNA polymerase catalytic subunit gi|9629818|ref|NC_001847.1| Bovine herpesvirus 1, complete genome
DNA polymerase catalytic subunit gi|126882977|ref|NC_002686.2| Cercopithecine herpesvirus 9, complete genome
DNA polymerase catalytic subunit gi|123318702|ref|NC_005261.2| Bovine herpesvirus 5, complete genome
DNA polymerase catalytic subunit gi|51557483|ref|NC_006151.1| Suid herpesvirus 1, complete genome
DNA polymerase catalytic subunit gi|216905852|ref|NC_011644.1| Equid herpesvirus 9, complete genome
DNA polymerase catalytic subunit gi|281190771|ref|NC_013590.2| Felid herpesvirus 1, complete genome
DNA polymerase catalytic subunit gi|386522723|ref|NC_017826.1| Equid herpesvirus 8, complete genome
DNA polymerase catalytic subunit gi|675510705|ref|NC_024771.1| Equid herpesvirus 3 strain AR/2007/C3A, complete genome

DNA polymerase processivity subunit gi|9625875|ref|NC_001348.1| Human herpesvirus 3, complete genome
DNA polymerase processivity subunit gi|50313241|ref|NC_001491.2| Equid herpesvirus 1, complete genome
DNA polymerase processivity subunit gi|9629732|ref|NC_001844.1| Equid herpesvirus 4, complete genome
DNA polymerase processivity subunit gi|9629818|ref|NC_001847.1| Bovine herpesvirus 1, complete genome
DNA polymerase processivity subunit gi|51557483|ref|NC_006151.1| Suid herpesvirus 1, complete genome
DNA polymerase processivity subunit gi|216905852|ref|NC_011644.1| Equid herpesvirus 9, complete genome
DNA polymerase processivity subunit gi|386522723|ref|NC_017826.1| Equid herpesvirus 8, complete genome
DNA polymerase processivity subunit gi|675510705|ref|NC_024771.1| Equid herpesvirus 3 strain AR/2007/C3A, complete genome
DNA replication origin-binding helicase gi|9625875|ref|NC_001348.1| Human herpesvirus 3, complete genome
DNA replication origin-binding helicase gi|50313241|ref|NC_001491.2| Equid herpesvirus 1, complete genome
DNA replication origin-binding helicase gi|9629732|ref|NC_001844.1| Equid herpesvirus 4, complete genome
DNA replication origin-binding helicase gi|9629818|ref|NC_001847.1| Bovine herpesvirus 1, complete genome
DNA replication origin-binding helicase gi|126882977|ref|NC_002686.2| Cercopithecine herpesvirus 9, complete genome
DNA replication origin-binding helicase gi|123318702|ref|NC_005261.2| Bovine herpesvirus 5, complete genome
DNA replication origin-binding helicase gi|51557483|ref|NC_006151.1| Suid herpesvirus 1, complete genome
DNA replication origin-binding helicase gi|216905852|ref|NC_011644.1| Equid herpesvirus 9, complete genome
DNA replication origin-binding helicase gi|281190771|ref|NC_013590.2| Felid herpesvirus 1, complete genome
DNA replication origin-binding helicase gi|675510705|ref|NC_024771.1| Equid herpesvirus 3 strain AR/2007/C3A, complete genome
envelope glycoprotein B gi|9625875|ref|NC_001348.1| Human herpesvirus 3, complete genome
envelope glycoprotein B gi|50313241|ref|NC_001491.2| Equid herpesvirus 1, complete genome
envelope glycoprotein B gi|9629732|ref|NC_001844.1| Equid herpesvirus 4, complete genome
envelope glycoprotein B gi|9629818|ref|NC_001847.1| Bovine herpesvirus 1, complete genome
envelope glycoprotein B gi|126882977|ref|NC_002686.2| Cercopithecine herpesvirus 9, complete genome

envelope glycoprotein B gi|123318702|ref|NC_005261.2| Bovine herpesvirus 5, complete genome
envelope glycoprotein B gi|51557483|ref|NC_006151.1| Suid herpesvirus 1, complete genome
envelope glycoprotein B gi|216905852|ref|NC_011644.1| Equid herpesvirus 9, complete genome
envelope glycoprotein B gi|281190771|ref|NC_013590.2| Felid herpesvirus 1, complete genome
envelope glycoprotein B gi|386522723|ref|NC_017826.1| Equid herpesvirus 8, complete genome
envelope glycoprotein B gi|675510705|ref|NC_024771.1| Equid herpesvirus 3 strain AR/2007/C3A, complete genome
envelope glycoprotein C gi|9625875|ref|NC_001348.1| Human herpesvirus 3, complete genome
envelope glycoprotein C gi|50313241|ref|NC_001491.2| Equid herpesvirus 1, complete genome
envelope glycoprotein C gi|9629732|ref|NC_001844.1| Equid herpesvirus 4, complete genome
envelope glycoprotein C gi|9629818|ref|NC_001847.1| Bovine herpesvirus 1, complete genome
envelope glycoprotein C gi|126882977|ref|NC_002686.2| Cercopithecine herpesvirus 9, complete genome
envelope glycoprotein C gi|123318702|ref|NC_005261.2| Bovine herpesvirus 5, complete genome
envelope glycoprotein C gi|51557483|ref|NC_006151.1| Suid herpesvirus 1, complete genome
envelope glycoprotein C gi|216905852|ref|NC_011644.1| Equid herpesvirus 9, complete genome
envelope glycoprotein C gi|281190771|ref|NC_013590.2| Felid herpesvirus 1, complete genome
envelope glycoprotein C gi|675510705|ref|NC_024771.1| Equid herpesvirus 3 strain AR/2007/C3A, complete genome
envelope glycoprotein D gi|50313241|ref|NC_001491.2| Equid herpesvirus 1, complete genome
envelope glycoprotein D gi|9629732|ref|NC_001844.1| Equid herpesvirus 4, complete genome
envelope glycoprotein D gi|9629818|ref|NC_001847.1| Bovine herpesvirus 1, complete genome
envelope glycoprotein D gi|123318702|ref|NC_005261.2| Bovine herpesvirus 5, complete genome
envelope glycoprotein D gi|51557483|ref|NC_006151.1| Suid herpesvirus 1, complete genome
envelope glycoprotein D gi|216905852|ref|NC_011644.1| Equid herpesvirus 9, complete genome
envelope glycoprotein D gi|281190771|ref|NC_013590.2| Felid herpesvirus 1, complete genome

envelope glycoprotein D gi|675510705|ref|NC_024771.1| Equid herpesvirus 3 strain AR/2007/C3A, complete genome
envelope glycoprotein E gi|9625875|ref|NC_001348.1| Human herpesvirus 3, complete genome
envelope glycoprotein E gi|50313241|ref|NC_001491.2| Equid herpesvirus 1, complete genome
envelope glycoprotein E gi|9629732|ref|NC_001844.1| Equid herpesvirus 4, complete genome
envelope glycoprotein E gi|9629818|ref|NC_001847.1| Bovine herpesvirus 1, complete genome
envelope glycoprotein E gi|126882977|ref|NC_002686.2| Cercopithecine herpesvirus 9, complete genome
envelope glycoprotein E gi|123318702|ref|NC_005261.2| Bovine herpesvirus 5, complete genome
envelope glycoprotein E gi|51557483|ref|NC_006151.1| Suid herpesvirus 1, complete genome
envelope glycoprotein E gi|216905852|ref|NC_011644.1| Equid herpesvirus 9, complete genome
envelope glycoprotein E gi|281190771|ref|NC_013590.2| Felid herpesvirus 1, complete genome
envelope glycoprotein E gi|386522723|ref|NC_017826.1| Equid herpesvirus 8, complete genome
envelope glycoprotein E gi|675510705|ref|NC_024771.1| Equid herpesvirus 3 strain AR/2007/C3A, complete genome
envelope glycoprotein G gi|50313241|ref|NC_001491.2| Equid herpesvirus 1, complete genome
envelope glycoprotein G gi|9629732|ref|NC_001844.1| Equid herpesvirus 4, complete genome
envelope glycoprotein G gi|9629818|ref|NC_001847.1| Bovine herpesvirus 1, complete genome
envelope glycoprotein G gi|123318702|ref|NC_005261.2| Bovine herpesvirus 5, complete genome
envelope glycoprotein G gi|51557483|ref|NC_006151.1| Suid herpesvirus 1, complete genome
envelope glycoprotein G gi|216905852|ref|NC_011644.1| Equid herpesvirus 9, complete genome
envelope glycoprotein G gi|281190771|ref|NC_013590.2| Felid herpesvirus 1, complete genome
envelope glycoprotein G gi|675510705|ref|NC_024771.1| Equid herpesvirus 3 strain AR/2007/C3A, complete genome
envelope glycoprotein H gi|9625875|ref|NC_001348.1| Human herpesvirus 3, complete genome
envelope glycoprotein H gi|50313241|ref|NC_001491.2| Equid herpesvirus 1, complete genome
envelope glycoprotein H gi|9629732|ref|NC_001844.1| Equid herpesvirus 4, complete genome

envelope glycoprotein H gi|9629818|ref|NC_001847.1| Bovine herpesvirus 1, complete genome
envelope glycoprotein H gi|126882977|ref|NC_002686.2| Cercopithecine herpesvirus 9, complete genome
envelope glycoprotein H gi|123318702|ref|NC_005261.2| Bovine herpesvirus 5, complete genome
envelope glycoprotein H gi|51557483|ref|NC_006151.1| Suid herpesvirus 1, complete genome
envelope glycoprotein H gi|216905852|ref|NC_011644.1| Equid herpesvirus 9, complete genome
envelope glycoprotein H gi|281190771|ref|NC_013590.2| Felid herpesvirus 1, complete genome
envelope glycoprotein H gi|386522723|ref|NC_017826.1| Equid herpesvirus 8, complete genome
envelope glycoprotein H gi|675510705|ref|NC_024771.1| Equid herpesvirus 3 strain AR/2007/C3A, complete genome
envelope glycoprotein I gi|9625875|ref|NC_001348.1| Human herpesvirus 3, complete genome
envelope glycoprotein I gi|50313241|ref|NC_001491.2| Equid herpesvirus 1, complete genome
envelope glycoprotein I gi|9629732|ref|NC_001844.1| Equid herpesvirus 4, complete genome
envelope glycoprotein I gi|9629818|ref|NC_001847.1| Bovine herpesvirus 1, complete genome
envelope glycoprotein I gi|123318702|ref|NC_005261.2| Bovine herpesvirus 5, complete genome
envelope glycoprotein I gi|51557483|ref|NC_006151.1| Suid herpesvirus 1, complete genome
envelope glycoprotein I gi|216905852|ref|NC_011644.1| Equid herpesvirus 9, complete genome
envelope glycoprotein I gi|281190771|ref|NC_013590.2| Felid herpesvirus 1, complete genome
envelope glycoprotein I gi|675510705|ref|NC_024771.1| Equid herpesvirus 3 strain AR/2007/C3A, complete genome
envelope glycoprotein J gi|50313241|ref|NC_001491.2| Equid herpesvirus 1, complete genome
envelope glycoprotein J gi|9629732|ref|NC_001844.1| Equid herpesvirus 4, complete genome
envelope glycoprotein J gi|216905852|ref|NC_011644.1| Equid herpesvirus 9, complete genome
envelope glycoprotein J gi|386522723|ref|NC_017826.1| Equid herpesvirus 8, complete genome
envelope glycoprotein J gi|675510705|ref|NC_024771.1| Equid herpesvirus 3 strain AR/2007/C3A, complete genome
envelope glycoprotein K gi|9625875|ref|NC_001348.1| Human herpesvirus 3, complete genome

envelope glycoprotein K gi|50313241|ref|NC_001491.2| Equid herpesvirus 1, complete genome
envelope glycoprotein K gi|9629732|ref|NC_001844.1| Equid herpesvirus 4, complete genome
envelope glycoprotein K gi|9629818|ref|NC_001847.1| Bovine herpesvirus 1, complete genome
envelope glycoprotein K gi|126882977|ref|NC_002686.2| Cercopithecine herpesvirus 9, complete genome
envelope glycoprotein K gi|123318702|ref|NC_005261.2| Bovine herpesvirus 5, complete genome
envelope glycoprotein K gi|51557483|ref|NC_006151.1| Suid herpesvirus 1, complete genome
envelope glycoprotein K gi|216905852|ref|NC_011644.1| Equid herpesvirus 9, complete genome
envelope glycoprotein K gi|281190771|ref|NC_013590.2| Felid herpesvirus 1, complete genome
envelope glycoprotein K gi|675510705|ref|NC_024771.1| Equid herpesvirus 3 strain AR/2007/C3A, complete genome
envelope glycoprotein L gi|9625875|ref|NC_001348.1| Human herpesvirus 3, complete genome
envelope glycoprotein L gi|9629818|ref|NC_001847.1| Bovine herpesvirus 1, complete genome
envelope glycoprotein L gi|123318702|ref|NC_005261.2| Bovine herpesvirus 5, complete genome
envelope glycoprotein L gi|51557483|ref|NC_006151.1| Suid herpesvirus 1, complete genome
envelope glycoprotein L gi|281190771|ref|NC_013590.2| Felid herpesvirus 1, complete genome
envelope glycoprotein L gi|675510705|ref|NC_024771.1| Equid herpesvirus 3 strain AR/2007/C3A, complete genome
envelope glycoprotein M gi|9625875|ref|NC_001348.1| Human herpesvirus 3, complete genome
envelope glycoprotein M gi|50313241|ref|NC_001491.2| Equid herpesvirus 1, complete genome
envelope glycoprotein M gi|9629732|ref|NC_001844.1| Equid herpesvirus 4, complete genome
envelope glycoprotein M gi|9629818|ref|NC_001847.1| Bovine herpesvirus 1, complete genome
envelope glycoprotein M gi|126882977|ref|NC_002686.2| Cercopithecine herpesvirus 9, complete genome
envelope glycoprotein M gi|123318702|ref|NC_005261.2| Bovine herpesvirus 5, complete genome
envelope glycoprotein M gi|51557483|ref|NC_006151.1| Suid herpesvirus 1, complete genome
envelope glycoprotein M gi|216905852|ref|NC_011644.1| Equid herpesvirus 9, complete genome

envelope glycoprotein M gi|281190771|ref|NC_013590.2| Felid herpesvirus 1, complete genome
envelope glycoprotein M gi|386522723|ref|NC_017826.1| Equid herpesvirus 8, complete genome
envelope glycoprotein M gi|675510705|ref|NC_024771.1| Equid herpesvirus 3 strain AR/2007/C3A, complete genome
envelope glycoprotein N gi|50313241|ref|NC_001491.2| Equid herpesvirus 1, complete genome
envelope glycoprotein N gi|9629732|ref|NC_001844.1| Equid herpesvirus 4, complete genome
envelope glycoprotein N gi|9629818|ref|NC_001847.1| Bovine herpesvirus 1, complete genome
envelope glycoprotein N gi|123318702|ref|NC_005261.2| Bovine herpesvirus 5, complete genome
envelope glycoprotein N gi|51557483|ref|NC_006151.1| Suid herpesvirus 1, complete genome
envelope glycoprotein N gi|216905852|ref|NC_011644.1| Equid herpesvirus 9, complete genome
envelope glycoprotein N gi|675510705|ref|NC_024771.1| Equid herpesvirus 3 strain AR/2007/C3A, complete genome
envelope protein UL20 gi|9625875|ref|NC_001348.1| Human herpesvirus 3, complete genome
envelope protein UL20 gi|50313241|ref|NC_001491.2| Equid herpesvirus 1, complete genome
envelope protein UL20 gi|9629818|ref|NC_001847.1| Bovine herpesvirus 1, complete genome
envelope protein UL20 gi|123318702|ref|NC_005261.2| Bovine herpesvirus 5, complete genome
envelope protein UL20 gi|51557483|ref|NC_006151.1| Suid herpesvirus 1, complete genome
envelope protein UL20 gi|675510705|ref|NC_024771.1| Equid herpesvirus 3 strain AR/2007/C3A, complete genome
envelope protein UL43 gi|9625875|ref|NC_001348.1| Human herpesvirus 3, complete genome
envelope protein UL43 gi|50313241|ref|NC_001491.2| Equid herpesvirus 1, complete genome
envelope protein UL43 gi|9629732|ref|NC_001844.1| Equid herpesvirus 4, complete genome
envelope protein UL43 gi|9629818|ref|NC_001847.1| Bovine herpesvirus 1, complete genome
envelope protein UL43 gi|126882977|ref|NC_002686.2| Cercopithecine herpesvirus 9, complete genome
envelope protein UL43 gi|123318702|ref|NC_005261.2| Bovine herpesvirus 5, complete genome
envelope protein UL43 gi|51557483|ref|NC_006151.1| Suid herpesvirus 1, complete genome

envelope protein UL43 gi|216905852|ref|NC_011644.1| Equid herpesvirus 9, complete genome
envelope protein UL43 gi|281190771|ref|NC_013590.2| Felid herpesvirus 1, complete genome
envelope protein UL43 gi|675510705|ref|NC_024771.1| Equid herpesvirus 3 strain AR/2007/C3A, complete genome
helicase-primase helicase subunit gi|9625875|ref|NC_001348.1| Human herpesvirus 3, complete genome
helicase-primase helicase subunit gi|50313241|ref|NC_001491.2| Equid herpesvirus 1, complete genome
helicase-primase helicase subunit gi|9629732|ref|NC_001844.1| Equid herpesvirus 4, complete genome
helicase-primase helicase subunit gi|9629818|ref|NC_001847.1| Bovine herpesvirus 1, complete genome
helicase-primase helicase subunit gi|123318702|ref|NC_005261.2| Bovine herpesvirus 5, complete genome
helicase-primase helicase subunit gi|51557483|ref|NC_006151.1| Suid herpesvirus 1, complete genome
helicase-primase helicase subunit gi|216905852|ref|NC_011644.1| Equid herpesvirus 9, complete genome
helicase-primase helicase subunit gi|281190771|ref|NC_013590.2| Felid herpesvirus 1, complete genome
helicase-primase helicase subunit gi|675510705|ref|NC_024771.1| Equid herpesvirus 3 strain AR/2007/C3A, complete genome
helicase-primase primase subunit gi|9625875|ref|NC_001348.1| Human herpesvirus 3, complete genome
helicase-primase primase subunit gi|50313241|ref|NC_001491.2| Equid herpesvirus 1, complete genome
helicase-primase primase subunit gi|9629732|ref|NC_001844.1| Equid herpesvirus 4, complete genome
helicase-primase primase subunit gi|9629818|ref|NC_001847.1| Bovine herpesvirus 1, complete genome
helicase-primase primase subunit gi|126882977|ref|NC_002686.2| Cercopithecine herpesvirus 9, complete genome
helicase-primase primase subunit gi|123318702|ref|NC_005261.2| Bovine herpesvirus 5, complete genome
helicase-primase primase subunit gi|51557483|ref|NC_006151.1| Suid herpesvirus 1, complete genome
helicase-primase primase subunit gi|216905852|ref|NC_011644.1| Equid herpesvirus 9, complete genome
helicase-primase primase subunit gi|281190771|ref|NC_013590.2| Felid herpesvirus 1, complete genome
helicase-primase primase subunit gi|386522723|ref|NC_017826.1| Equid herpesvirus 8, complete genome
helicase-primase primase subunit gi|675510705|ref|NC_024771.1| Equid herpesvirus 3 strain AR/2007/C3A, complete genome

helicase-primase subunit	gi|9625875|ref|NC_001348.1| Human herpesvirus 3, complete genome
helicase-primase subunit	gi|50313241|ref|NC_001491.2| Equid herpesvirus 1, complete genome
helicase-primase subunit	gi|9629732|ref|NC_001844.1| Equid herpesvirus 4, complete genome
helicase-primase subunit	gi|9629818|ref|NC_001847.1| Bovine herpesvirus 1, complete genome
helicase-primase subunit	gi|126882977|ref|NC_002686.2| Cercopithecine herpesvirus 9, complete genome
helicase-primase subunit	gi|123318702|ref|NC_005261.2| Bovine herpesvirus 5, complete genome
helicase-primase subunit	gi|51557483|ref|NC_006151.1| Suid herpesvirus 1, complete genome
helicase-primase subunit	gi|216905852|ref|NC_011644.1| Equid herpesvirus 9, complete genome
helicase-primase subunit	gi|281190771|ref|NC_013590.2| Felid herpesvirus 1, complete genome
helicase-primase subunit	gi|386522723|ref|NC_017826.1| Equid herpesvirus 8, complete genome
helicase-primase subunit	gi|675510705|ref|NC_024771.1| Equid herpesvirus 3 strain AR/2007/C3A, complete genome
hypothetical protein	gi|386522723|ref|NC_017826.1| Equid herpesvirus 8, complete genome
large latency transcript	gi|51557483|ref|NC_006151.1| Suid herpesvirus 1, complete genome
large tegument protein	gi|9625875|ref|NC_001348.1| Human herpesvirus 3, complete genome
large tegument protein	gi|50313241|ref|NC_001491.2| Equid herpesvirus 1, complete genome
large tegument protein	gi|9629732|ref|NC_001844.1| Equid herpesvirus 4, complete genome
large tegument protein	gi|9629818|ref|NC_001847.1| Bovine herpesvirus 1, complete genome
large tegument protein	gi|126882977|ref|NC_002686.2| Cercopithecine herpesvirus 9, complete genome
large tegument protein	gi|123318702|ref|NC_005261.2| Bovine herpesvirus 5, complete genome
large tegument protein	gi|51557483|ref|NC_006151.1| Suid herpesvirus 1, complete genome
large tegument protein	gi|216905852|ref|NC_011644.1| Equid herpesvirus 9, complete genome
large tegument protein	gi|281190771|ref|NC_013590.2| Felid herpesvirus 1, complete genome
large tegument protein	gi|675510705|ref|NC_024771.1| Equid herpesvirus 3 strain AR/2007/C3A, complete genome

major capsid protein	gi|9625875|ref|NC_001348.1| Human herpesvirus 3, complete genome
major capsid protein	gi|50313241|ref|NC_001491.2| Equid herpesvirus 1, complete genome
major capsid protein	gi|9629732|ref|NC_001844.1| Equid herpesvirus 4, complete genome
major capsid protein	gi|9629818|ref|NC_001847.1| Bovine herpesvirus 1, complete genome
major capsid protein	gi|126882977|ref|NC_002686.2| Cercopithecine herpesvirus 9, complete genome
major capsid protein	gi|123318702|ref|NC_005261.2| Bovine herpesvirus 5, complete genome
major capsid protein	gi|51557483|ref|NC_006151.1| Suid herpesvirus 1, complete genome
major capsid protein	gi|216905852|ref|NC_011644.1| Equid herpesvirus 9, complete genome
major capsid protein	gi|281190771|ref|NC_013590.2| Felid herpesvirus 1, complete genome
major capsid protein	gi|675510705|ref|NC_024771.1| Equid herpesvirus 3 strain AR/2007/C3A, complete genome
membrane protein UL45	gi|9629732|ref|NC_001844.1| Equid herpesvirus 4, complete genome
membrane protein UL45	gi|216905852|ref|NC_011644.1| Equid herpesvirus 9, complete genome
membrane protein UL45	gi|281190771|ref|NC_013590.2| Felid herpesvirus 1, complete genome
membrane protein UL45	gi|386522723|ref|NC_017826.1| Equid herpesvirus 8, complete genome
membrane protein UL45	gi|675510705|ref|NC_024771.1| Equid herpesvirus 3 strain AR/2007/C3A, complete genome
membrane protein UL56	gi|9625875|ref|NC_001348.1| Human herpesvirus 3, complete genome
membrane protein UL56	gi|50313241|ref|NC_001491.2| Equid herpesvirus 1, complete genome
membrane protein UL56	gi|51557483|ref|NC_006151.1| Suid herpesvirus 1, complete genome
membrane protein UL56	gi|216905852|ref|NC_011644.1| Equid herpesvirus 9, complete genome
membrane protein UL56	gi|386522723|ref|NC_017826.1| Equid herpesvirus 8, complete genome
membrane protein UL56	gi|675510705|ref|NC_024771.1| Equid herpesvirus 3 strain AR/2007/C3A, complete genome
membrane protein UL56A	gi|126882977|ref|NC_002686.2| Cercopithecine herpesvirus 9, complete genome
membrane protein US8A	gi|50313241|ref|NC_001491.2| Equid herpesvirus 1, complete genome

membrane protein US8A   gi|9629732|ref|NC_001844.1| Equid herpesvirus 4, complete genome
membrane protein US8A   gi|216905852|ref|NC_011644.1| Equid herpesvirus 9, complete genome
membrane protein US9   gi|9625875|ref|NC_001348.1| Human herpesvirus 3, complete genome
membrane protein US9   gi|50313241|ref|NC_001491.2| Equid herpesvirus 1, complete genome
membrane protein US9   gi|9629732|ref|NC_001844.1| Equid herpesvirus 4, complete genome
membrane protein US9   gi|9629818|ref|NC_001847.1| Bovine herpesvirus 1, complete genome
membrane protein US9   gi|123318702|ref|NC_005261.2| Bovine herpesvirus 5, complete genome
membrane protein US9   gi|51557483|ref|NC_006151.1| Suid herpesvirus 1, complete genome
membrane protein US9   gi|216905852|ref|NC_011644.1| Equid herpesvirus 9, complete genome
membrane protein US9   gi|281190771|ref|NC_013590.2| Felid herpesvirus 1, complete genome
membrane protein US9   gi|675510705|ref|NC_024771.1| Equid herpesvirus 3 strain AR/2007/C3A, complete genome
membrane protein V1 gi|9625875|ref|NC_001348.1| Human herpesvirus 3, complete genome
membrane protein V1 gi|50313241|ref|NC_001491.2| Equid herpesvirus 1, complete genome
membrane protein V1 gi|216905852|ref|NC_011644.1| Equid herpesvirus 9, complete genome
membrane protein V1 gi|386522723|ref|NC_017826.1| Equid herpesvirus 8, complete genome
membrane protein V1 gi|675510705|ref|NC_024771.1| Equid herpesvirus 3 strain AR/2007/C3A, complete genome
multifunctional expression regulator   gi|9625875|ref|NC_001348.1| Human herpesvirus 3, complete genome
multifunctional expression regulator   gi|50313241|ref|NC_001491.2| Equid herpesvirus 1, complete genome
multifunctional expression regulator   gi|9629732|ref|NC_001844.1| Equid herpesvirus 4, complete genome
multifunctional expression regulator   gi|9629818|ref|NC_001847.1| Bovine herpesvirus 1, complete genome
multifunctional expression regulator   gi|123318702|ref|NC_005261.2| Bovine herpesvirus 5, complete genome
multifunctional expression regulator   gi|51557483|ref|NC_006151.1| Suid herpesvirus 1, complete genome
multifunctional expression regulator   gi|216905852|ref|NC_011644.1| Equid herpesvirus 9, complete genome

multifunctional expression regulator gi|281190771|ref|NC_013590.2| Felid herpesvirus 1, complete genome
multifunctional expression regulator gi|386522723|ref|NC_017826.1| Equid herpesvirus 8, complete genome
multifunctional expression regulator gi|675510705|ref|NC_024771.1| Equid herpesvirus 3 strain AR/2007/C3A, complete genome
multifunctional expression regulator-related protein gi|126882977|ref|NC_002686.2| Cercopithecine herpesvirus 9, complete genome
myristylated tegument protein gi|9625875|ref|NC_001348.1| Human herpesvirus 3, complete genome
myristylated tegument protein gi|50313241|ref|NC_001491.2| Equid herpesvirus 1, complete genome
myristylated tegument protein gi|9629732|ref|NC_001844.1| Equid herpesvirus 4, complete genome
myristylated tegument protein gi|9629818|ref|NC_001847.1| Bovine herpesvirus 1, complete genome
myristylated tegument protein gi|126882977|ref|NC_002686.2| Cercopithecine herpesvirus 9, complete genome
myristylated tegument protein gi|123318702|ref|NC_005261.2| Bovine herpesvirus 5, complete genome
myristylated tegument protein gi|51557483|ref|NC_006151.1| Suid herpesvirus 1, complete genome
myristylated tegument protein gi|216905852|ref|NC_011644.1| Equid herpesvirus 9, complete genome
myristylated tegument protein gi|675510705|ref|NC_024771.1| Equid herpesvirus 3 strain AR/2007/C3A, complete genome
myristylated tegument protein CIRC gi|9625875|ref|NC_001348.1| Human herpesvirus 3, complete genome
myristylated tegument protein CIRC gi|50313241|ref|NC_001491.2| Equid herpesvirus 1, complete genome
myristylated tegument protein CIRC gi|9629732|ref|NC_001844.1| Equid herpesvirus 4, complete genome
myristylated tegument protein CIRC gi|9629818|ref|NC_001847.1| Bovine herpesvirus 1, complete genome
myristylated tegument protein CIRC gi|123318702|ref|NC_005261.2| Bovine herpesvirus 5, complete genome
myristylated tegument protein CIRC gi|216905852|ref|NC_011644.1| Equid herpesvirus 9, complete genome
myristylated tegument protein CIRC gi|386522723|ref|NC_017826.1| Equid herpesvirus 8, complete genome
myristylated tegument protein CIRC gi|675510705|ref|NC_024771.1| Equid herpesvirus 3 strain AR/2007/C3A, complete genome
nuclear egress lamina protein gi|9625875|ref|NC_001348.1| Human herpesvirus 3, complete genome
nuclear egress lamina protein gi|50313241|ref|NC_001491.2| Equid herpesvirus 1, complete genome

nuclear egress lamina protein gi|9629818|ref|NC_001847.1| Bovine herpesvirus 1, complete genome
nuclear egress lamina protein gi|123318702|ref|NC_005261.2| Bovine herpesvirus 5, complete genome
nuclear egress lamina protein gi|51557483|ref|NC_006151.1| Suid herpesvirus 1, complete genome
nuclear egress lamina protein gi|216905852|ref|NC_011644.1| Equid herpesvirus 9, complete genome
nuclear egress lamina protein gi|675510705|ref|NC_024771.1| Equid herpesvirus 3 strain AR/2007/C3A, complete genome
nuclear egress membrane protein gi|9625875|ref|NC_001348.1| Human herpesvirus 3, complete genome
nuclear egress membrane protein gi|50313241|ref|NC_001491.2| Equid herpesvirus 1, complete genome
nuclear egress membrane protein gi|9629732|ref|NC_001844.1| Equid herpesvirus 4, complete genome
nuclear egress membrane protein gi|9629818|ref|NC_001847.1| Bovine herpesvirus 1, complete genome
nuclear egress membrane protein gi|126882977|ref|NC_002686.2| Cercopithecine herpesvirus 9, complete genome
nuclear egress membrane protein gi|123318702|ref|NC_005261.2| Bovine herpesvirus 5, complete genome
nuclear egress membrane protein gi|51557483|ref|NC_006151.1| Suid herpesvirus 1, complete genome
nuclear egress membrane protein gi|216905852|ref|NC_011644.1| Equid herpesvirus 9, complete genome
nuclear egress membrane protein gi|281190771|ref|NC_013590.2| Felid herpesvirus 1, complete genome
nuclear egress membrane protein gi|675510705|ref|NC_024771.1| Equid herpesvirus 3 strain AR/2007/C3A, complete genome
nuclear protein UL24 gi|50313241|ref|NC_001491.2| Equid herpesvirus 1, complete genome
nuclear protein UL24 gi|9629732|ref|NC_001844.1| Equid herpesvirus 4, complete genome
nuclear protein UL24 gi|9629818|ref|NC_001847.1| Bovine herpesvirus 1, complete genome
nuclear protein UL24 gi|126882977|ref|NC_002686.2| Cercopithecine herpesvirus 9, complete genome
nuclear protein UL24 gi|123318702|ref|NC_005261.2| Bovine herpesvirus 5, complete genome
nuclear protein UL24 gi|51557483|ref|NC_006151.1| Suid herpesvirus 1, complete genome
nuclear protein UL24 gi|216905852|ref|NC_011644.1| Equid herpesvirus 9, complete genome
nuclear protein UL24 gi|281190771|ref|NC_013590.2| Felid herpesvirus 1, complete genome

nuclear protein UL24 gi|675510705|ref|NC_024771.1| Equid herpesvirus 3 strain AR/2007/C3A, complete genome
nuclear protein UL3 gi|9625875|ref|NC_001348.1| Human herpesvirus 3, complete genome
nuclear protein UL3 gi|50313241|ref|NC_001491.2| Equid herpesvirus 1, complete genome
nuclear protein UL3 gi|9629732|ref|NC_001844.1| Equid herpesvirus 4, complete genome
nuclear protein UL3 gi|9629818|ref|NC_001847.1| Bovine herpesvirus 1, complete genome
nuclear protein UL3 gi|123318702|ref|NC_005261.2| Bovine herpesvirus 5, complete genome
nuclear protein UL3 gi|51557483|ref|NC_006151.1| Suid herpesvirus 1, complete genome
nuclear protein UL3 gi|216905852|ref|NC_011644.1| Equid herpesvirus 9, complete genome
nuclear protein UL3 gi|281190771|ref|NC_013590.2| Felid herpesvirus 1, complete genome
nuclear protein UL3 gi|675510705|ref|NC_024771.1| Equid herpesvirus 3 strain AR/2007/C3A, complete genome
nuclear protein UL4 gi|9625875|ref|NC_001348.1| Human herpesvirus 3, complete genome
nuclear protein UL4 gi|50313241|ref|NC_001491.2| Equid herpesvirus 1, complete genome
nuclear protein UL4 gi|9629732|ref|NC_001844.1| Equid herpesvirus 4, complete genome
nuclear protein UL4 gi|9629818|ref|NC_001847.1| Bovine herpesvirus 1, complete genome
nuclear protein UL4 gi|123318702|ref|NC_005261.2| Bovine herpesvirus 5, complete genome
nuclear protein UL4 gi|51557483|ref|NC_006151.1| Suid herpesvirus 1, complete genome
nuclear protein UL4 gi|216905852|ref|NC_011644.1| Equid herpesvirus 9, complete genome
nuclear protein UL4 gi|281190771|ref|NC_013590.2| Felid herpesvirus 1, complete genome
nuclear protein UL4 gi|386522723|ref|NC_017826.1| Equid herpesvirus 8, complete genome
nuclear protein UL4 gi|675510705|ref|NC_024771.1| Equid herpesvirus 3 strain AR/2007/C3A, complete genome
nuclear protein UL55 gi|9625875|ref|NC_001348.1| Human herpesvirus 3, complete genome
nuclear protein UL55 gi|50313241|ref|NC_001491.2| Equid herpesvirus 1, complete genome
nuclear protein UL55 gi|9629732|ref|NC_001844.1| Equid herpesvirus 4, complete genome

nuclear protein UL55	gi|126882977|ref|NC_002686.2| Cercopithecine herpesvirus 9, complete genome
nuclear protein UL55	gi|386522723|ref|NC_017826.1| Equid herpesvirus 8, complete genome
nuclear protein UL55	gi|675510705|ref|NC_024771.1| Equid herpesvirus 3 strain AR/2007/C3A, complete genome
protein V32 gi|9625875|ref|NC_001348.1| Human herpesvirus 3, complete genome
protein V32 gi|50313241|ref|NC_001491.2| Equid herpesvirus 1, complete genome
protein V32 gi|9629732|ref|NC_001844.1| Equid herpesvirus 4, complete genome
protein V32 gi|216905852|ref|NC_011644.1| Equid herpesvirus 9, complete genome
protein V32 gi|675510705|ref|NC_024771.1| Equid herpesvirus 3 strain AR/2007/C3A, complete genome
protein V57 gi|50313241|ref|NC_001491.2| Equid herpesvirus 1, complete genome
protein V57 gi|9629818|ref|NC_001847.1| Bovine herpesvirus 1, complete genome
protein V57 gi|123318702|ref|NC_005261.2| Bovine herpesvirus 5, complete genome
protein V57 gi|51557483|ref|NC_006151.1| Suid herpesvirus 1, complete genome
protein V57 gi|216905852|ref|NC_011644.1| Equid herpesvirus 9, complete genome
protein V57 gi|281190771|ref|NC_013590.2| Felid herpesvirus 1, complete genome
protein V57 gi|675510705|ref|NC_024771.1| Equid herpesvirus 3 strain AR/2007/C3A, complete genome
putative membrane protein US8A	gi|675510705|ref|NC_024771.1| Equid herpesvirus 3 strain AR/2007/C3A, complete genome
regulatory protein ICP22	gi|9625875|ref|NC_001348.1| Human herpesvirus 3, complete genome
regulatory protein ICP22	gi|50313241|ref|NC_001491.2| Equid herpesvirus 1, complete genome
regulatory protein ICP22	gi|9629732|ref|NC_001844.1| Equid herpesvirus 4, complete genome
regulatory protein ICP22	gi|9629818|ref|NC_001847.1| Bovine herpesvirus 1, complete genome
regulatory protein ICP22	gi|126882977|ref|NC_002686.2| Cercopithecine herpesvirus 9, complete genome
regulatory protein ICP22	gi|123318702|ref|NC_005261.2| Bovine herpesvirus 5, complete genome
regulatory protein ICP22	gi|51557483|ref|NC_006151.1| Suid herpesvirus 1, complete genome

regulatory protein ICP22   gi|216905852|ref|NC_011644.1| Equid herpesvirus 9, complete genome
regulatory protein ICP22   gi|281190771|ref|NC_013590.2| Felid herpesvirus 1, complete genome
regulatory protein ICP22   gi|386522723|ref|NC_017826.1| Equid herpesvirus 8, complete genome
regulatory protein ICP22   gi|675510705|ref|NC_024771.1| Equid herpesvirus 3 strain AR/2007/C3A, complete genome
ribonucleotide reductase subunit 1 gi|9625875|ref|NC_001348.1| Human herpesvirus 3, complete genome
ribonucleotide reductase subunit 1 gi|50313241|ref|NC_001491.2| Equid herpesvirus 1, complete genome
ribonucleotide reductase subunit 1 gi|9629732|ref|NC_001844.1| Equid herpesvirus 4, complete genome
ribonucleotide reductase subunit 1 gi|9629818|ref|NC_001847.1| Bovine herpesvirus 1, complete genome
ribonucleotide reductase subunit 1 gi|123318702|ref|NC_005261.2| Bovine herpesvirus 5, complete genome
ribonucleotide reductase subunit 1 gi|51557483|ref|NC_006151.1| Suid herpesvirus 1, complete genome
ribonucleotide reductase subunit 1 gi|216905852|ref|NC_011644.1| Equid herpesvirus 9, complete genome
ribonucleotide reductase subunit 1 gi|281190771|ref|NC_013590.2| Felid herpesvirus 1, complete genome
ribonucleotide reductase subunit 1 gi|675510705|ref|NC_024771.1| Equid herpesvirus 3 strain AR/2007/C3A, complete genome
ribonucleotide reductase subunit 2 gi|9625875|ref|NC_001348.1| Human herpesvirus 3, complete genome
ribonucleotide reductase subunit 2 gi|50313241|ref|NC_001491.2| Equid herpesvirus 1, complete genome
ribonucleotide reductase subunit 2 gi|9629732|ref|NC_001844.1| Equid herpesvirus 4, complete genome
ribonucleotide reductase subunit 2 gi|9629818|ref|NC_001847.1| Bovine herpesvirus 1, complete genome
ribonucleotide reductase subunit 2 gi|126882977|ref|NC_002686.2| Cercopithecine herpesvirus 9, complete genome
ribonucleotide reductase subunit 2 gi|123318702|ref|NC_005261.2| Bovine herpesvirus 5, complete genome
ribonucleotide reductase subunit 2 gi|51557483|ref|NC_006151.1| Suid herpesvirus 1, complete genome
ribonucleotide reductase subunit 2 gi|216905852|ref|NC_011644.1| Equid herpesvirus 9, complete genome
ribonucleotide reductase subunit 2 gi|281190771|ref|NC_013590.2| Felid herpesvirus 1, complete genome
ribonucleotide reductase subunit 2 gi|675510705|ref|NC_024771.1| Equid herpesvirus 3 strain AR/2007/C3A, complete genome

serine/threonine protein kinase US3 gi|9625875|ref|NC_001348.1| Human herpesvirus 3, complete genome
serine/threonine protein kinase US3 gi|50313241|ref|NC_001491.2| Equid herpesvirus 1, complete genome
serine/threonine protein kinase US3 gi|9629732|ref|NC_001844.1| Equid herpesvirus 4, complete genome
serine/threonine protein kinase US3 gi|9629818|ref|NC_001847.1| Bovine herpesvirus 1, complete genome
serine/threonine protein kinase US3 gi|126882977|ref|NC_002686.2| Cercopithecine herpesvirus 9, complete genome
serine/threonine protein kinase US3 gi|123318702|ref|NC_005261.2| Bovine herpesvirus 5, complete genome
serine/threonine protein kinase US3 gi|51557483|ref|NC_006151.1| Suid herpesvirus 1, complete genome
serine/threonine protein kinase US3 gi|216905852|ref|NC_011644.1| Equid herpesvirus 9, complete genome
serine/threonine protein kinase US3 gi|281190771|ref|NC_013590.2| Felid herpesvirus 1, complete genome
serine/threonine protein kinase US3 gi|675510705|ref|NC_024771.1| Equid herpesvirus 3 strain AR/2007/C3A, complete genome
small capsid protein gi|9625875|ref|NC_001348.1| Human herpesvirus 3, complete genome
small capsid protein gi|9629818|ref|NC_001847.1| Bovine herpesvirus 1, complete genome
small capsid protein gi|123318702|ref|NC_005261.2| Bovine herpesvirus 5, complete genome
small capsid protein gi|51557483|ref|NC_006151.1| Suid herpesvirus 1, complete genome
small capsid protein gi|675510705|ref|NC_024771.1| Equid herpesvirus 3 strain AR/2007/C3A, complete genome
tegument host shutoff protein gi|9625875|ref|NC_001348.1| Human herpesvirus 3, complete genome
tegument host shutoff protein gi|50313241|ref|NC_001491.2| Equid herpesvirus 1, complete genome
tegument host shutoff protein gi|9629732|ref|NC_001844.1| Equid herpesvirus 4, complete genome
tegument host shutoff protein gi|9629818|ref|NC_001847.1| Bovine herpesvirus 1, complete genome
tegument host shutoff protein gi|123318702|ref|NC_005261.2| Bovine herpesvirus 5, complete genome
tegument host shutoff protein gi|51557483|ref|NC_006151.1| Suid herpesvirus 1, complete genome
tegument host shutoff protein gi|216905852|ref|NC_011644.1| Equid herpesvirus 9, complete genome
tegument host shutoff protein gi|281190771|ref|NC_013590.2| Felid herpesvirus 1, complete genome

tegument host shut-off protein gi|675510705|ref|NC_024771.1| Equid herpesvirus 3 strain AR/2007/C3A, complete genome
tegument protein UL14   gi|9625875|ref|NC_001348.1| Human herpesvirus 3, complete genome
tegument protein UL14   gi|50313241|ref|NC_001491.2| Equid herpesvirus 1, complete genome
tegument protein UL14   gi|9629732|ref|NC_001844.1| Equid herpesvirus 4, complete genome
tegument protein UL14   gi|9629818|ref|NC_001847.1| Bovine herpesvirus 1, complete genome
tegument protein UL14   gi|126882977|ref|NC_002686.2| Cercopithecine herpesvirus 9, complete genome
tegument protein UL14   gi|123318702|ref|NC_005261.2| Bovine herpesvirus 5, complete genome
tegument protein UL14   gi|51557483|ref|NC_006151.1| Suid herpesvirus 1, complete genome
tegument protein UL14   gi|216905852|ref|NC_011644.1| Equid herpesvirus 9, complete genome
tegument protein UL14   gi|281190771|ref|NC_013590.2| Felid herpesvirus 1, complete genome
tegument protein UL14   gi|386522723|ref|NC_017826.1| Equid herpesvirus 8, complete genome
tegument protein UL14   gi|675510705|ref|NC_024771.1| Equid herpesvirus 3 strain AR/2007/C3A, complete genome
tegument protein UL16   gi|9625875|ref|NC_001348.1| Human herpesvirus 3, complete genome
tegument protein UL16   gi|50313241|ref|NC_001491.2| Equid herpesvirus 1, complete genome
tegument protein UL16   gi|9629732|ref|NC_001844.1| Equid herpesvirus 4, complete genome
tegument protein UL16   gi|9629818|ref|NC_001847.1| Bovine herpesvirus 1, complete genome
tegument protein UL16   gi|126882977|ref|NC_002686.2| Cercopithecine herpesvirus 9, complete genome
tegument protein UL16   gi|123318702|ref|NC_005261.2| Bovine herpesvirus 5, complete genome
tegument protein UL16   gi|51557483|ref|NC_006151.1| Suid herpesvirus 1, complete genome
tegument protein UL16   gi|216905852|ref|NC_011644.1| Equid herpesvirus 9, complete genome
tegument protein UL16   gi|281190771|ref|NC_013590.2| Felid herpesvirus 1, complete genome
tegument protein UL16   gi|675510705|ref|NC_024771.1| Equid herpesvirus 3 strain AR/2007/C3A, complete genome
tegument protein UL21   gi|9625875|ref|NC_001348.1| Human herpesvirus 3, complete genome

tegument protein UL21	gi|50313241|ref|NC_001491.2| Equid herpesvirus 1, complete genome
tegument protein UL21	gi|9629732|ref|NC_001844.1| Equid herpesvirus 4, complete genome
tegument protein UL21	gi|9629818|ref|NC_001847.1| Bovine herpesvirus 1, complete genome
tegument protein UL21	gi|126882977|ref|NC_002686.2| Cercopithecine herpesvirus 9, complete genome
tegument protein UL21	gi|123318702|ref|NC_005261.2| Bovine herpesvirus 5, complete genome
tegument protein UL21	gi|51557483|ref|NC_006151.1| Suid herpesvirus 1, complete genome
tegument protein UL21	gi|216905852|ref|NC_011644.1| Equid herpesvirus 9, complete genome
tegument protein UL21	gi|281190771|ref|NC_013590.2| Felid herpesvirus 1, complete genome
tegument protein UL21	gi|675510705|ref|NC_024771.1| Equid herpesvirus 3 strain AR/2007/C3A, complete genome
tegument protein UL37	gi|9625875|ref|NC_001348.1| Human herpesvirus 3, complete genome
tegument protein UL37	gi|50313241|ref|NC_001491.2| Equid herpesvirus 1, complete genome
tegument protein UL37	gi|9629732|ref|NC_001844.1| Equid herpesvirus 4, complete genome
tegument protein UL37	gi|9629818|ref|NC_001847.1| Bovine herpesvirus 1, complete genome
tegument protein UL37	gi|123318702|ref|NC_005261.2| Bovine herpesvirus 5, complete genome
tegument protein UL37	gi|51557483|ref|NC_006151.1| Suid herpesvirus 1, complete genome
tegument protein UL37	gi|216905852|ref|NC_011644.1| Equid herpesvirus 9, complete genome
tegument protein UL37	gi|281190771|ref|NC_013590.2| Felid herpesvirus 1, complete genome
tegument protein UL37	gi|675510705|ref|NC_024771.1| Equid herpesvirus 3 strain AR/2007/C3A, complete genome
tegument protein UL51	gi|9625875|ref|NC_001348.1| Human herpesvirus 3, complete genome
tegument protein UL51	gi|50313241|ref|NC_001491.2| Equid herpesvirus 1, complete genome
tegument protein UL51	gi|9629732|ref|NC_001844.1| Equid herpesvirus 4, complete genome
tegument protein UL51	gi|9629818|ref|NC_001847.1| Bovine herpesvirus 1, complete genome
tegument protein UL51	gi|126882977|ref|NC_002686.2| Cercopithecine herpesvirus 9, complete genome

tegument protein UL51   gi|123318702|ref|NC_005261.2| Bovine herpesvirus 5, complete genome
tegument protein UL51   gi|51557483|ref|NC_006151.1| Suid herpesvirus 1, complete genome
tegument protein UL51   gi|216905852|ref|NC_011644.1| Equid herpesvirus 9, complete genome
tegument protein UL51   gi|281190771|ref|NC_013590.2| Felid herpesvirus 1, complete genome
tegument protein UL51   gi|386522723|ref|NC_017826.1| Equid herpesvirus 8, complete genome
tegument protein UL51   gi|675510705|ref|NC_024771.1| Equid herpesvirus 3 strain AR/2007/C3A, complete genome
tegument protein UL7   gi|9625875|ref|NC_001348.1| Human herpesvirus 3, complete genome
tegument protein UL7   gi|50313241|ref|NC_001491.2| Equid herpesvirus 1, complete genome
tegument protein UL7   gi|9629732|ref|NC_001844.1| Equid herpesvirus 4, complete genome
tegument protein UL7   gi|9629818|ref|NC_001847.1| Bovine herpesvirus 1, complete genome
tegument protein UL7   gi|126882977|ref|NC_002686.2| Cercopithecine herpesvirus 9, complete genome
tegument protein UL7   gi|123318702|ref|NC_005261.2| Bovine herpesvirus 5, complete genome
tegument protein UL7   gi|51557483|ref|NC_006151.1| Suid herpesvirus 1, complete genome
tegument protein UL7   gi|216905852|ref|NC_011644.1| Equid herpesvirus 9, complete genome
tegument protein UL7   gi|281190771|ref|NC_013590.2| Felid herpesvirus 1, complete genome
tegument protein UL7   gi|675510705|ref|NC_024771.1| Equid herpesvirus 3 strain AR/2007/C3A, complete genome
tegument protein VP11/12   gi|9625875|ref|NC_001348.1| Human herpesvirus 3, complete genome
tegument protein VP11/12   gi|50313241|ref|NC_001491.2| Equid herpesvirus 1, complete genome
tegument protein VP11/12   gi|9629732|ref|NC_001844.1| Equid herpesvirus 4, complete genome
tegument protein VP11/12   gi|9629818|ref|NC_001847.1| Bovine herpesvirus 1, complete genome
tegument protein VP11/12   gi|126882977|ref|NC_002686.2| Cercopithecine herpesvirus 9, complete genome
tegument protein VP11/12   gi|123318702|ref|NC_005261.2| Bovine herpesvirus 5, complete genome
tegument protein VP11/12   gi|51557483|ref|NC_006151.1| Suid herpesvirus 1, complete genome

tegument protein VP11/12   gi|216905852|ref|NC_011644.1| Equid herpesvirus 9, complete genome
tegument protein VP11/12   gi|281190771|ref|NC_013590.2| Felid herpesvirus 1, complete genome
tegument protein VP11/12   gi|675510705|ref|NC_024771.1| Equid herpesvirus 3 strain AR/2007/C3A, complete genome
tegument protein VP13/14   gi|50313241|ref|NC_001491.2| Equid herpesvirus 1, complete genome
tegument protein VP13/14   gi|9629732|ref|NC_001844.1| Equid herpesvirus 4, complete genome
tegument protein VP13/14   gi|9629818|ref|NC_001847.1| Bovine herpesvirus 1, complete genome
tegument protein VP13/14   gi|126882977|ref|NC_002686.2| Cercopithecine herpesvirus 9, complete genome
tegument protein VP13/14   gi|123318702|ref|NC_005261.2| Bovine herpesvirus 5, complete genome
tegument protein VP13/14   gi|51557483|ref|NC_006151.1| Suid herpesvirus 1, complete genome
tegument protein VP13/14   gi|216905852|ref|NC_011644.1| Equid herpesvirus 9, complete genome
tegument protein VP13/14   gi|281190771|ref|NC_013590.2| Felid herpesvirus 1, complete genome
tegument protein VP13/14   gi|675510705|ref|NC_024771.1| Equid herpesvirus 3 strain AR/2007/C3A, complete genome
tegument protein VP22   gi|50313241|ref|NC_001491.2| Equid herpesvirus 1, complete genome
tegument protein VP22   gi|9629732|ref|NC_001844.1| Equid herpesvirus 4, complete genome
tegument protein VP22   gi|9629818|ref|NC_001847.1| Bovine herpesvirus 1, complete genome
tegument protein VP22   gi|123318702|ref|NC_005261.2| Bovine herpesvirus 5, complete genome
tegument protein VP22   gi|51557483|ref|NC_006151.1| Suid herpesvirus 1, complete genome
tegument protein VP22   gi|216905852|ref|NC_011644.1| Equid herpesvirus 9, complete genome
tegument protein VP22   gi|281190771|ref|NC_013590.2| Felid herpesvirus 1, complete genome
tegument protein VP22   gi|386522723|ref|NC_017826.1| Equid herpesvirus 8, complete genome
tegument protein VP22   gi|675510705|ref|NC_024771.1| Equid herpesvirus 3 strain AR/2007/C3A, complete genome
tegument serine/threonine protein kinase   gi|9625875|ref|NC_001348.1| Human herpesvirus 3, complete genome
tegument serine/threonine protein kinase   gi|50313241|ref|NC_001491.2| Equid herpesvirus 1, complete genome

tegument serine/threonine protein kinase gi|9629732|ref|NC_001844.1| Equid herpesvirus 4, complete genome
tegument serine/threonine protein kinase gi|9629818|ref|NC_001847.1| Bovine herpesvirus 1, complete genome
tegument serine/threonine protein kinase gi|123318702|ref|NC_005261.2| Bovine herpesvirus 5, complete genome
tegument serine/threonine protein kinase gi|51557483|ref|NC_006151.1| Suid herpesvirus 1, complete genome
tegument serine/threonine protein kinase gi|216905852|ref|NC_011644.1| Equid herpesvirus 9, complete genome
tegument serine/threonine protein kinase gi|281190771|ref|NC_013590.2| Felid herpesvirus 1, complete genome
tegument serine/threonine protein kinase gi|386522723|ref|NC_017826.1| Equid herpesvirus 8, complete genome
tegument serine/threonine protein kinase gi|675510705|ref|NC_024771.1| Equid herpesvirus 3 strain AR/2007/C3A, complete genome
thymidine kinase gi|9625875|ref|NC_001348.1| Human herpesvirus 3, complete genome
thymidine kinase gi|50313241|ref|NC_001491.2| Equid herpesvirus 1, complete genome
thymidine kinase gi|9629732|ref|NC_001844.1| Equid herpesvirus 4, complete genome
thymidine kinase gi|9629818|ref|NC_001847.1| Bovine herpesvirus 1, complete genome
thymidine kinase gi|126882977|ref|NC_002686.2| Cercopithecine herpesvirus 9, complete genome
thymidine kinase gi|123318702|ref|NC_005261.2| Bovine herpesvirus 5, complete genome
thymidine kinase gi|51557483|ref|NC_006151.1| Suid herpesvirus 1, complete genome
thymidine kinase gi|216905852|ref|NC_011644.1| Equid herpesvirus 9, complete genome
thymidine kinase gi|281190771|ref|NC_013590.2| Felid herpesvirus 1, complete genome
thymidine kinase gi|386522723|ref|NC_017826.1| Equid herpesvirus 8, complete genome
thymidine kinase gi|675510705|ref|NC_024771.1| Equid herpesvirus 3 strain AR/2007/C3A, complete genome
thymidylate synthase gi|9625875|ref|NC_001348.1| Human herpesvirus 3, complete genome
transactivating tegument protein VP16 gi|9625875|ref|NC_001348.1| Human herpesvirus 3, complete genome
transactivating tegument protein VP16 gi|50313241|ref|NC_001491.2| Equid herpesvirus 1, complete genome
transactivating tegument protein VP16 gi|9629732|ref|NC_001844.1| Equid herpesvirus 4, complete genome

transactivating tegument protein VP16 gi|9629818|ref|NC_001847.1| Bovine herpesvirus 1, complete genome
transactivating tegument protein VP16 gi|126882977|ref|NC_002686.2| Cercopithecine herpesvirus 9, complete genome
transactivating tegument protein VP16 gi|123318702|ref|NC_005261.2| Bovine herpesvirus 5, complete genome
transactivating tegument protein VP16 gi|51557483|ref|NC_006151.1| Suid herpesvirus 1, complete genome
transactivating tegument protein VP16 gi|216905852|ref|NC_011644.1| Equid herpesvirus 9, complete genome
transactivating tegument protein VP16 gi|281190771|ref|NC_013590.2| Felid herpesvirus 1, complete genome
transactivating tegument protein VP16 gi|675510705|ref|NC_024771.1| Equid herpesvirus 3 strain AR/2007/C3A, complete genome
transcriptional regulator ICP4 gi|9625875|ref|NC_001348.1| Human herpesvirus 3, complete genome
transcriptional regulator ICP4 gi|50313241|ref|NC_001491.2| Equid herpesvirus 1, complete genome
transcriptional regulator ICP4 gi|9629732|ref|NC_001844.1| Equid herpesvirus 4, complete genome
transcriptional regulator ICP4 gi|9629818|ref|NC_001847.1| Bovine herpesvirus 1, complete genome
transcriptional regulator ICP4 gi|126882977|ref|NC_002686.2| Cercopithecine herpesvirus 9, complete genome
transcriptional regulator ICP4 gi|123318702|ref|NC_005261.2| Bovine herpesvirus 5, complete genome
transcriptional regulator ICP4 gi|51557483|ref|NC_006151.1| Suid herpesvirus 1, complete genome
transcriptional regulator ICP4 gi|216905852|ref|NC_011644.1| Equid herpesvirus 9, complete genome
transcriptional regulator ICP4 gi|281190771|ref|NC_013590.2| Felid herpesvirus 1, complete genome
transcriptional regulator ICP4 gi|675510705|ref|NC_024771.1| Equid herpesvirus 3 strain AR/2007/C3A, complete genome
ubiquitin E3 ligase ICP0 gi|9625875|ref|NC_001348.1| Human herpesvirus 3, complete genome
ubiquitin E3 ligase ICP0 gi|50313241|ref|NC_001491.2| Equid herpesvirus 1, complete genome
ubiquitin E3 ligase ICP0 gi|9629732|ref|NC_001844.1| Equid herpesvirus 4, complete genome
ubiquitin E3 ligase ICP0 gi|9629818|ref|NC_001847.1| Bovine herpesvirus 1, complete genome
ubiquitin E3 ligase ICP0 gi|126882977|ref|NC_002686.2| Cercopithecine herpesvirus 9, complete genome
ubiquitin E3 ligase ICP0 gi|123318702|ref|NC_005261.2| Bovine herpesvirus 5, complete genome

ubiquitin E3 ligase ICP0   gi|51557483|ref|NC_006151.1| Suid herpesvirus 1, complete genome
ubiquitin E3 ligase ICP0   gi|216905852|ref|NC_011644.1| Equid herpesvirus 9, complete genome
ubiquitin E3 ligase ICP0   gi|281190771|ref|NC_013590.2| Felid herpesvirus 1, complete genome
ubiquitin E3 ligase ICP0   gi|675510705|ref|NC_024771.1| Equid herpesvirus 3 strain AR/2007/C3A, complete genome
uracil DNA glycosylase gi|9629818|ref|NC_001847.1| Bovine herpesvirus 1, complete genome
uracil DNA glycosylase gi|126882977|ref|NC_002686.2| Cercopithecine herpesvirus 9, complete genome
uracil-DNA glycosylase gi|9625875|ref|NC_001348.1| Human herpesvirus 3, complete genome
uracil-DNA glycosylase gi|50313241|ref|NC_001491.2| Equid herpesvirus 1, complete genome
uracil-DNA glycosylase gi|9629732|ref|NC_001844.1| Equid herpesvirus 4, complete genome
uracil-DNA glycosylase gi|123318702|ref|NC_005261.2| Bovine herpesvirus 5, complete genome
uracil-DNA glycosylase gi|51557483|ref|NC_006151.1| Suid herpesvirus 1, complete genome
uracil-DNA glycosylase gi|216905852|ref|NC_011644.1| Equid herpesvirus 9, complete genome
uracil-DNA glycosylase gi|281190771|ref|NC_013590.2| Felid herpesvirus 1, complete genome
uracil-DNA glycosylase gi|675510705|ref|NC_024771.1| Equid herpesvirus 3 strain AR/2007/C3A, complete genome
virion protein gi|216905852|ref|NC_011644.1| Equid herpesvirus 9, complete genome
virion protein US10 gi|9625875|ref|NC_001348.1| Human herpesvirus 3, complete genome
virion protein US10 gi|50313241|ref|NC_001491.2| Equid herpesvirus 1, complete genome
virion protein US10 gi|9629732|ref|NC_001844.1| Equid herpesvirus 4, complete genome
virion protein US10 gi|126882977|ref|NC_002686.2| Cercopithecine herpesvirus 9, complete genome
virion protein US10 gi|281190771|ref|NC_013590.2| Felid herpesvirus 1, complete genome
virion protein US10 gi|386522723|ref|NC_017826.1| Equid herpesvirus 8, complete genome
virion protein US10 gi|675510705|ref|NC_024771.1| Equid herpesvirus 3 strain AR/2007/C3A, complete genome
virion protein US2 gi|50313241|ref|NC_001491.2| Equid herpesvirus 1, complete genome

virion protein US2 gi|9629732|ref|NC_001844.1| Equid herpesvirus 4, complete genome
virion protein US2 gi|9629818|ref|NC_001847.1| Bovine herpesvirus 1, complete genome
virion protein US2 gi|123318702|ref|NC_005261.2| Bovine herpesvirus 5, complete genome
virion protein US2 gi|51557483|ref|NC_006151.1| Suid herpesvirus 1, complete genome
virion protein US2 gi|216905852|ref|NC_011644.1| Equid herpesvirus 9, complete genome
virion protein US2 gi|675510705|ref|NC_024771.1| Equid herpesvirus 3 strain AR/2007/C3A, complete genome
virion protein V67 gi|50313241|ref|NC_001491.2| Equid herpesvirus 1, complete genome
virion protein V67 gi|9629732|ref|NC_001844.1| Equid herpesvirus 4, complete genome
virion protein V67 gi|9629818|ref|NC_001847.1| Bovine herpesvirus 1, complete genome
virion protein V67 gi|123318702|ref|NC_005261.2| Bovine herpesvirus 5, complete genome
virion protein V67 gi|216905852|ref|NC_011644.1| Equid herpesvirus 9, complete genome
virion protein V67 gi|675510705|ref|NC_024771.1| Equid herpesvirus 3 strain AR/2007/C3A, complete genome
