# Supplementary material for: Intensive Distribution of G2-Quaduplexes in the Pseudorabies Virus Genome and Their Sensitivity to Cations and G-Quadruplex Ligands
Source: Molecules. 2019 Feb 21;24(4):774. doi: 10.3390/molecules24040774 (PMC6412908; doi:10.3390/molecules24040774)
Supplement: Supplementary file 1 [file molecules-24-00774-s001.zip › Supporting information/Table S1.pdf]

Table S1. G_2-PQSs with 100% conservation in repeat regions among twenty-five PRV genome sequences.

Sequence 5′-3′	Nucleotide position	Genome Location
GGGGAAGGGTCGGGCGATGG	110127-110146	IRS
GGCACCACAGGCGGGAGCGGGGTCGCTCGGGGGAAGG	110470-110506	IRS
GGCGCGGCCGGCTCGGCGGCCCGG	111721-111744	IRS
GGCGGCGGGGGAGCGCGG	111813-111830	IRS
GGGCGGGGCGGGGG	131926-131939	TRS
GGAGAAGGCGAGGGAGACGGAGAGGGAGG	132667-132695	TRS
GGACCGAGGCGACGGCGGAGACGGAGACGG	134715-134744	TRS
GGGGGCGGGGG	135068-135078	TRS
GGTTCGGGTCCGGTCGCCGGTCCCCGGTCCGACGGCGG	135706-135743	TRS
GGCCGCCGCGGGGGCCGG	136420-136437	TRS
GGGGGAAGCGGAAGGGGCCGG	142824-142844	TRS
