# Supplementary material for: Intensive Distribution of G2-Quaduplexes in the Pseudorabies Virus Genome and Their Sensitivity to Cations and G-Quadruplex Ligands
Source: Molecules. 2019 Feb 21;24(4):774. doi: 10.3390/molecules24040774 (PMC6412908; doi:10.3390/molecules24040774)
Supplement: Supplementary file 1 [file molecules-24-00774-s001.zip › Supporting information/Table S2.pdf]

Table S2. G_2-PQSs with 100% conservation in the large latency transcripts among twenty-five PRV genome sequences.

Sequence 5′-3′	Nucleotide position
GGGGGCAGGAGCGGCGGCGG	96413-96432
GGTAGCGGAGCGGGGGG	96514-96530
GGGGGGCGGCGGTCTCGGGGGCAGAGGCAGAGGGCGACGGG	96564-96604
GGGCCGGCCCCGGCCCCCGAGG	96674-96695
GGGGAGCGCCTGGGCGCCGGCCTCTGG	96839-96865
GGTGGAGGATGGAGGTGACGCGGG	97434-97457
GGCGCTGGAAGCCTCGGCGGTGG	97660-97682
GGGGGGTTGATGGGGATGGGCGCTCGGGGG	99290-99319
GGGGGCGAGGCAGGG	99533-99547
GGTGGTGGGGATGAGTGGATGGATGGAGGCGAGGGACGCGGTGG	99899-99942
GGATGGCGAGGATGTGGGGGGAGAGGTCTCCCGGCGTTAGTGGG	100455-100498
GGGGGCGGGGCTTAAAGGGGGGGGG	101174-101198
GGGGGTCCGCGGGCGGGGG	101863-101881
GGAGCAGCAGGTAGGGGTTGCCGGCGTCCTCGG	103176-103208
GGGCCCGAGCGGGCCGCGGGGCCGG	103243-103267
GGGGCGGCCTCGGCGTCGGG	103472-103491
GGCGGTAGGCGCGCGGCGGCAGCGGGACCGGGGTCCGGGGCCCGGCGCGGG	103737-103787
GGCGGCAGAGGCGCAGCGGCTCGGCCCCGGGGTGCAGGCGGGCGAAGGAGG	103815-103865
GGCGGTGGGCGAAGGCGG	104220-104237
GGCCGCCGCGGCGCGGGTCCCAGGCCGGGCGCGGGGCGCCCTCGGCGGGCTCGG	104253-104306
GGGCGGCCGGGTCGAAGG	104436-104453
GGGCGCGGGGTGCTCGGGCATGGGCCCGAGCGGGCGCCGGAGCCGG	104599-104644
GGGAGCGGGGTCCGGAGCGGG	104683-104703
GGGGCGGCGGGCCCCGGGCGCGCGG	104824-104848
GGCGCCACGGTGCGGGCGACGAGGG	105230-105254
GGGGCACGCGGCCGGGCTGCGCGGCGGCGAAGGCGGCGCGGACGCGGGCGCAGAGG GCCTCGACGG	105462-105527
GGCGGCTCATGGCCACGG	105633-105650
GGGTCCTGGGCGGCCATGG	105680-105698
GGTGATGAAGGAGCCGTGGCCGTGGGGCGCGTGGACCCGGCGCTGGCAGAACTGG	105736-105790
GGACATGGCCTCGCCGGCGG	105835-105854
GGGCACGGGCCCGGCCGCGGCGCGGTAGCGGGCCGCGGCCTGGCGG	105925-105970
GGACGCGGCCGTCGGCCGGAGGGTCGGAGCCGGGCCAGGG	106023-106062
GGCCCGTCGGTCGGCGGGGGCCCGTCGG	106085-106112
GGCGCGGCGCCGGCGGGG	106507-106524
GGGGCTCGCGGTGCTGGTGATGG	106719-106741
GGCGGAGGGGG	106762-106772
GGCTGTGCTGGTGGCGCCGGGGTCCGAGG	106865-106893
GGGGTCGGCGGCCGGGGCCCGGAGCCGGCCCGGGACCGGGGCCCGAGGACGAGG	107063-107116
GGGGGACCCGGCGGCCGGGGACCCGGG	107152-107178
GGCGGCCGCCGGCCAATGGG	107939-107958
