# Supplementary material for: Intensive Distribution of G2-Quaduplexes in the Pseudorabies Virus Genome and Their Sensitivity to Cations and G-Quadruplex Ligands
Source: Molecules. 2019 Feb 21;24(4):774. doi: 10.3390/molecules24040774 (PMC6412908; doi:10.3390/molecules24040774)
Supplement: Supplementary file 1 [file molecules-24-00774-s001.zip › Supporting information/Table S3.pdf]

Table S3. Oligonucleotide sequences for Circular dichroism (CD) spectroscopy.

| PQS | Site | x:y:z | Label | Location |
|---|---|---|---|---|
| GGTGGAGGATGGAGGTGACGCGGG | 97434-97457 | 6:03:01 | LLT-PQS1 | Start-Prv-miR-1-5′ |
| GGCGCTGCGGCGGTGGCGAGGGGCTTCTGGGGCGG | 97605-97639 | 7:04:01 | LLT-PQS2 | Start-Prv-miR-1-5′ |
| GGAGGAGGAGGAGGAGGAGG | 104653-104672 | 13:10:03 | LLT-PQS3 | Prv-miR-11-1-End |
| GGCGGCGGCGGGGGCCCGGGGGGCGGAGGGCGAGCGGGCCGGGGAGAGG | 106393-106441 | 12:09:03 | LLT-PQS4 | Prv-miR-11-1-End |
| GGACCGGGGACCGGCGACCGG | 108866-108886 | 4:01:01 | LLT-PQS5 | Prv-miR-11-1-End |
| GGACCCGGAAGGAAGGAGCCGG | 108952-108973 | 5:02:01 | LLT-PQS6 | Prv-miR-11-1-End |
| GGAGGGTCGGGAGGACGGATGGTCGGTCGGACGGGGCGGGCGGGGAGGG | 109610-109658 | 12:09:03 | IRS-PQS1 | Between *IE180* and *US1* |
| GGCGGAGGAGGAGGAAGAAGGAGAGGAAGG | 111118-111147 | 8:05:02 | IRS-PQS2 | Between *IE180* and *US1* |
| GGGGGGCGTGGGGGTCGAGGCGGGGGACGGGGGG | 128470-128503 | 9:06:02 | TRS-PQS1 | Complementary to *US1* CDS |
| GGGTGGGGGTGGCTGGATGGATTGGATGGATTGAATGGGTGG | 128810-128851 | 10:07:02 | TRS-PQS2 | Complementary to *US1* CDS |
| GGGGGAGGGGAGGGGGGAGG | 128984-129003 | 6:03:01 | TRS-PQS3 | Complementary to *US1* CDS |
| GGGGTGGGGAGGGAGGGGGGACGGGGAGG | 129122-129150 | 7:04:01 | TRS-PQS4 | Complementary to *US1* CDS |
| GGGTGGGTGGGGGAGGGGGTTGATGGCCGCGCTGGTTCCGGG | 131809-131850 | 9:06:02 | TRS-PQS5 | Between *IE180* and *US1* |
| GGAGTGAGAGGCGGAGAGGGGCGGGCGAGCGGAGGAGG | 92293-92330 | 8:05:02 | UL5-3UTR | *UL5* 3′UTR |
| GGGGATCGACCGGGAGAGGCGCGG | 115268-115291 | 4:01:01 | US1-5UTR | *US1* 5′UTR |
