# Supplementary material for: Intensive Distribution of G2-Quaduplexes in the Pseudorabies Virus Genome and Their Sensitivity to Cations and G-Quadruplex Ligands
Source: Molecules. 2019 Feb 21;24(4):774. doi: 10.3390/molecules24040774 (PMC6412908; doi:10.3390/molecules24040774)
Supplement: Supplementary file 1 [file molecules-24-00774-s001.zip › Supporting information/Table S4.pdf]

Table S4. PQSs from *UL5* 3’UTR and *UL9* 3’UTR, and mutant sequence.

Name	Sequence (5’-3’)
UL5-3’UTR-2G	GGCGAGCGGAGGAGG
UL9-3’UTR-2G	GGCGGGCGGCGG
UL9-3’UTR-3G-mut	GGGCGGGGCGGGCGGG
