# Supplementary material for: Intensive Distribution of G2-Quaduplexes in the Pseudorabies Virus Genome and Their Sensitivity to Cations and G-Quadruplex Ligands
Source: Molecules. 2019 Feb 21;24(4):774. doi: 10.3390/molecules24040774 (PMC6412908; doi:10.3390/molecules24040774)
Supplement: Supplementary file 1 [file molecules-24-00774-s001.zip › Supporting information/Table S5.pdf]

Table S5. Oligonucleotide sequences for plasmids construction.

Primer	Sequence (5′-3′)
UL5-3′UTR-F	GGCGAGCGGAGGAGGAATAAAATATCTTTATTTTC
UL5-3′UTR-R	CCTCCTCCGCTCGCCTTACTGCTCGTTCTTCAGCA
UL9-3′UTR-F	GGCGGGCGGCGGAATAAAATATCTTTATTTTC
UL9-3′UTR-R	CCGCCGCCCGCCTTACTGCTCGTTCTTCAGCA
