# Supplementary figures and images for: Intensive Distribution of G2-Quaduplexes in the Pseudorabies Virus Genome and Their Sensitivity to Cations and G-Quadruplex Ligands
Source: Molecules. 2019 Feb 21;24(4):774. doi: 10.3390/molecules24040774 (PMC6412908; doi:10.3390/molecules24040774)

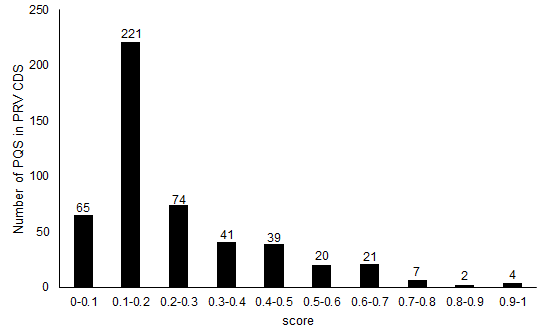

Supplement: Supplementary file 1 [file molecules-24-00774-s001.zip › Supporting information/Figure S1.tif]

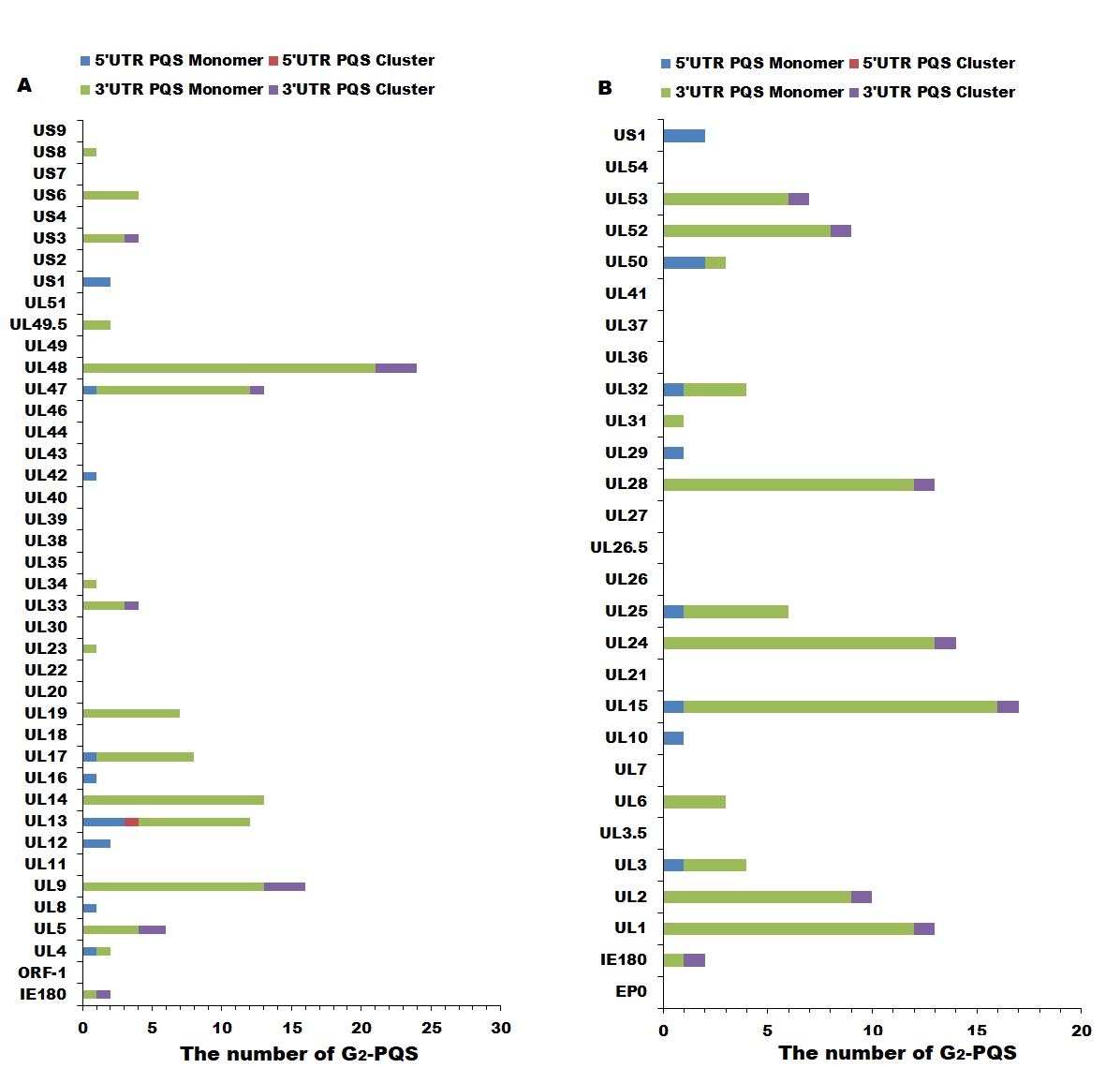

Supplement: Supplementary file 1 [file molecules-24-00774-s001.zip › Supporting information/Figure S2.tif]

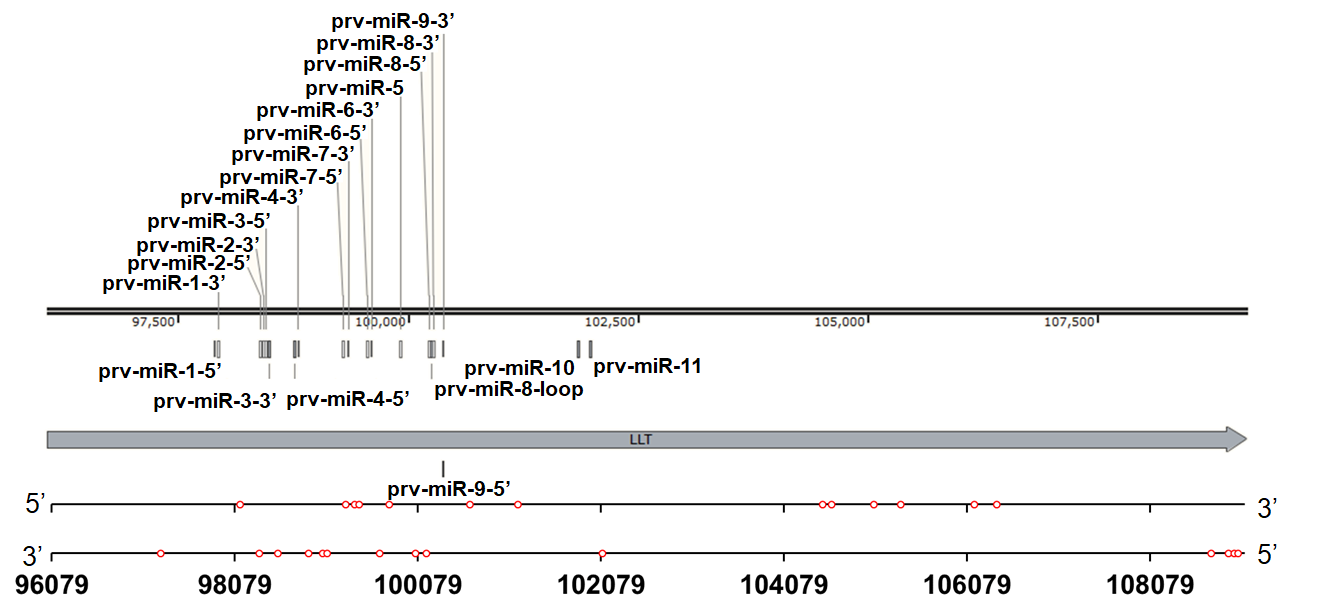

Supplement: Supplementary file 1 [file molecules-24-00774-s001.zip › Supporting information/Figure S3.tif]

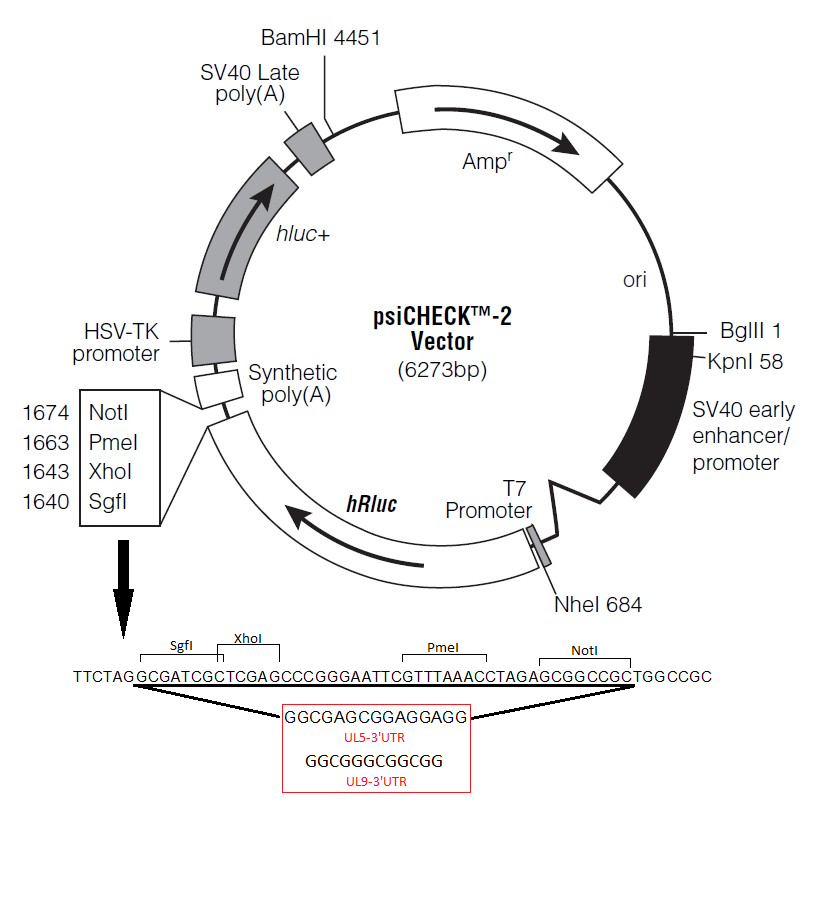

Supplement: Supplementary file 1 [file molecules-24-00774-s001.zip › Supporting information/Figure S4.tif]

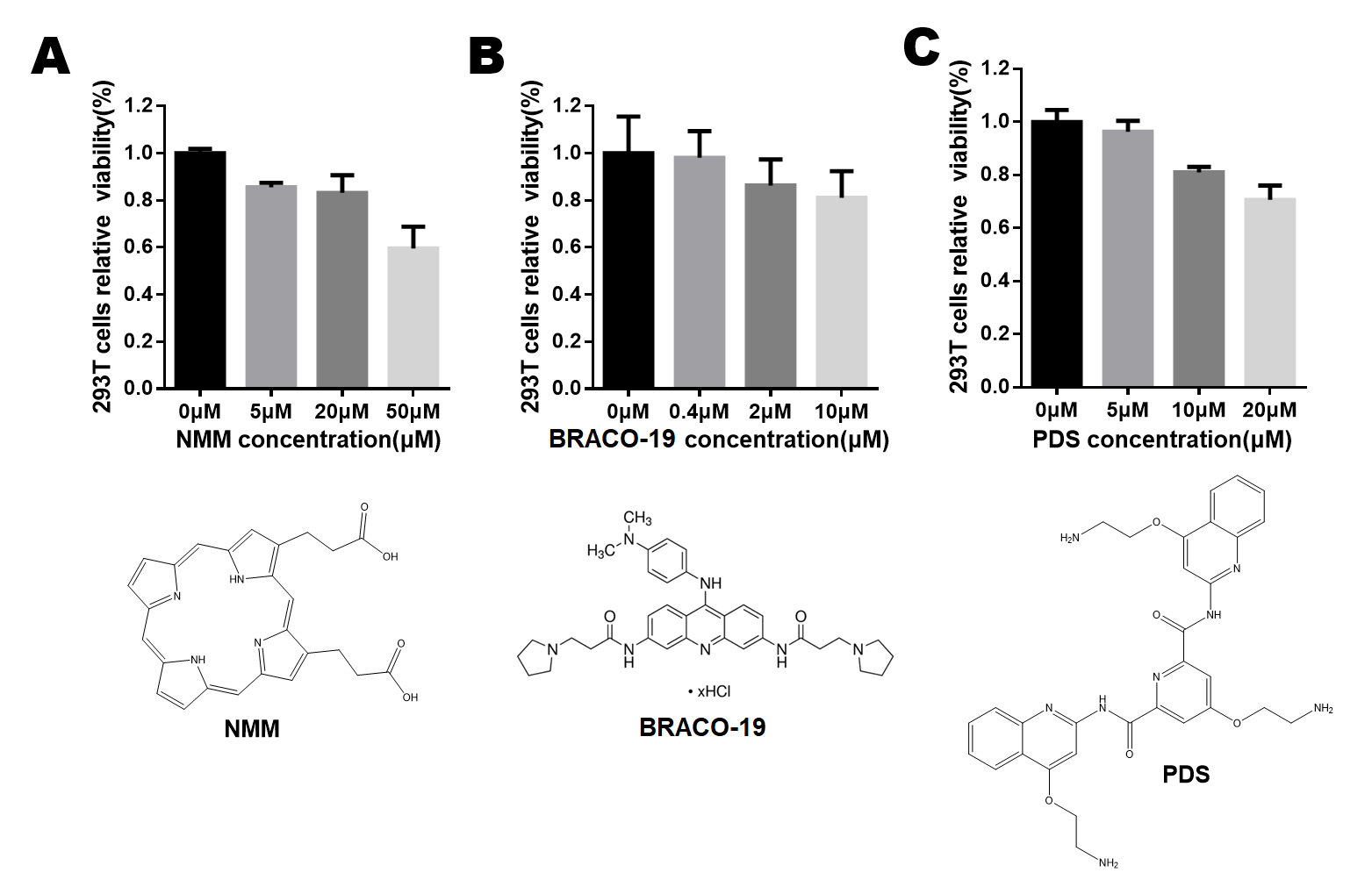

Supplement: Supplementary file 1 [file molecules-24-00774-s001.zip › Supporting information/Figure S5.tif]

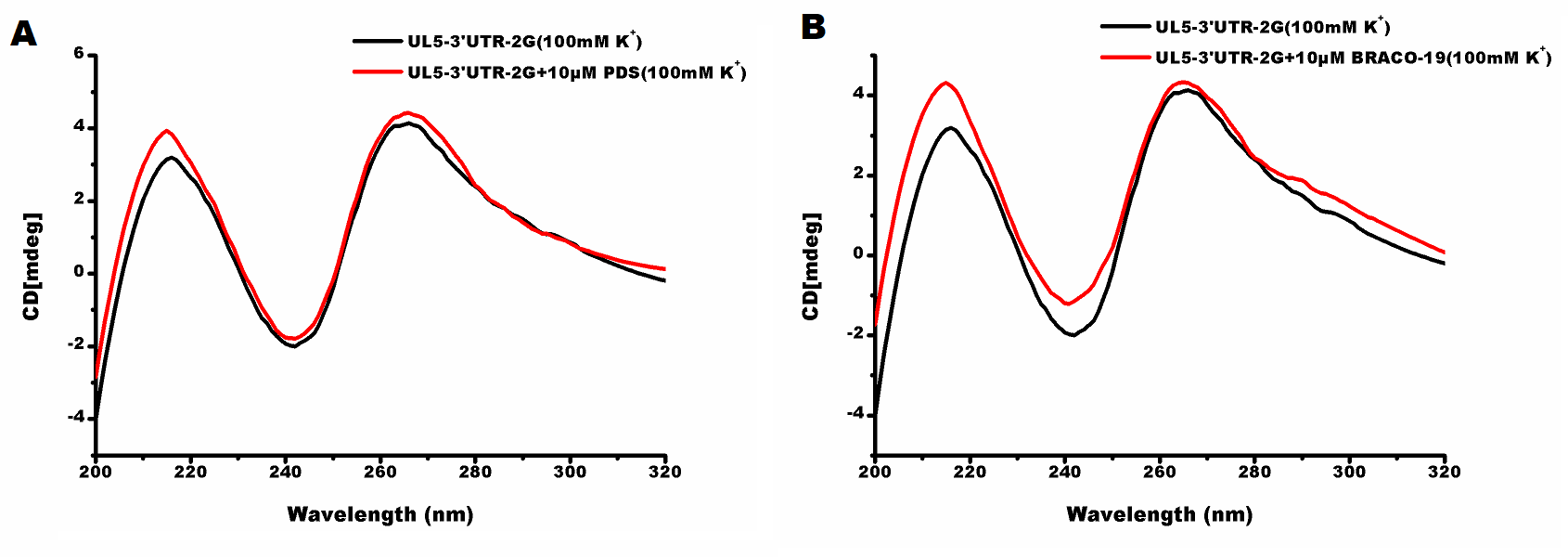

Supplement: Supplementary file 1 [file molecules-24-00774-s001.zip › Supporting information/Figure S6.tif]

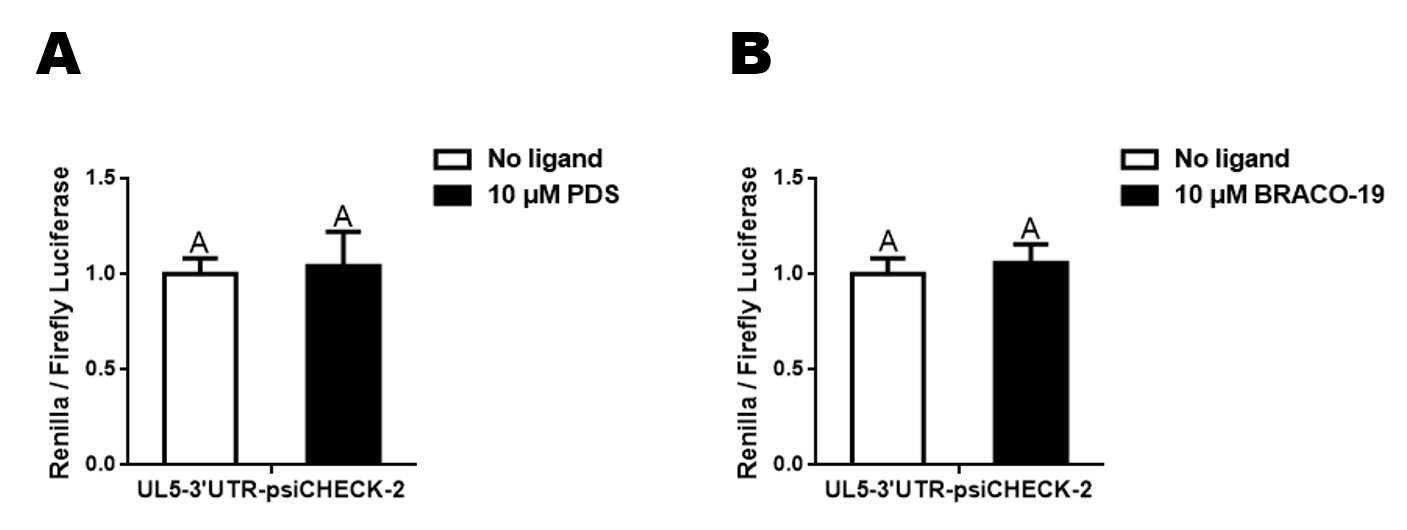

Supplement: Supplementary file 1 [file molecules-24-00774-s001.zip › Supporting information/Figure S7.tif]

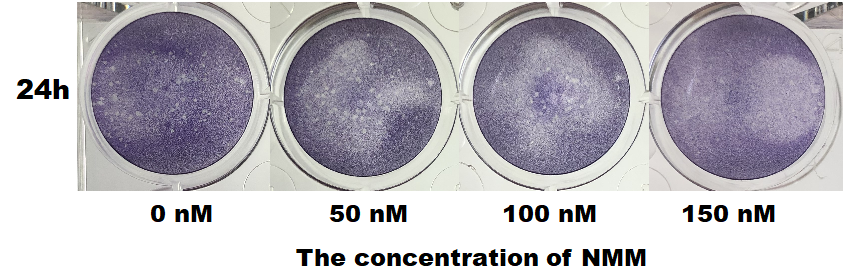

Supplement: Supplementary file 1 [file molecules-24-00774-s001.zip › Supporting information/Figure S8.tif]
